# Effects of Lycium Barbarum Polysaccharides on the Metabolism of Dendritic Cells: An *In Vitro* Study

**DOI:** 10.1155/2022/5882136

**Published:** 2022-10-19

**Authors:** Baochen Zhang, Kengyu Chen, Li Liu, Xiuyun Li, Enhui Wu, Liang Han, Zhongfeng Shi, Xiangliang Deng

**Affiliations:** ^1^School of Chinese Medicine, Guangdong Pharmaceutical University, Guangzhou, Guangzhou 510006, China; ^2^School of Pharmacy (The Center for Drug Research and Development), Guangdong Pharmaceutical University, Guangzhou, Guangzhou 510006, China; ^3^School of Health (Guangdong Light and Health Engineering R&D Center), Guangdong Pharmaceutical University, Guangzhou, Guangzhou 510006, China

## Abstract

Targeting dendritic cells (DCs) metabolism-related pathways and in-situ activation of DCs have become a new trend in DC-based immunotherapy. Studies have shown that *Lycium barbarum* polysaccharide can promote DCs function. This study is aimed at exploring the mechanism of LBP affecting DCs function from the perspective of metabolomics. MTT method was used to detect the activity of DC2.4 cells. ELISA kit method was used to detect the contents of IL-6, IL-12, and TNF-*α* in the supernatant of cells. Ultra-performance liquid chromatography-quadrupole-time-of-flight mass spectrometry (UPLC-Q-TOF/MS) was used to detect general changes in DC2.4 cell metabolism. And then multidistance covariates and bioinformatics, partial least squares-discriminant analysis (PLS-DA) were used to analyze differential metabolites. Finally, metabolic pathway analysis was performed by MetaboAnalyst v5.0. The results showed that LBP had no significant inhibitory effect on the activity of DC2.4 cells at the experimental dose of 50-200 *μ*g/ml. LBP (100 *μ*g/ml) could significantly stimulate DC2.4 cells to secrete IL-6, TNF-*α*, and IL-12. Moreover, 20 differential metabolites could be identified, including betaine, hypoxanthine, L-carnitine, 5'-methylthioadenosine, orotic acid, sphingomyelin, and L-glutamine. These metabolites were involved 28 metabolic pathways and the top 5 metabolic pathways were aspartate metabolism, pyrimidine metabolism, phenylacetate metabolism, methionine metabolism, and fatty acid metabolism. These results suggest that the effect of LBP on DCs function is related to the regulation of cell metabolism.

## 1. Introduction

Dendritic cells (DCs) were discovered and identified as highly effective antigen-presenting cells that can differentiate and capture antigens, before they migrate to the lymph node to present antigens and effectively activate T cells [1]. Currently, among the five types of DCs, conventional DCs (cDCs) including cDCs1 and cDCs2 can, respectively, polarize naive CD8^+^ and CD4^+^ T cells [2]. Also, DCs can induce cytotoxicity and proliferation of natural killer (NK) cells through direct cell-cell interaction and the secretion of some cytokines (e.g., IL-12, IL-15, etc.) [3]. The effects of DCs on the function of T cell and NK cell contribute to promoting the antitumor immunity. For example, when DCs recognize tumor antigens, they will be activated and migrate to the tumor draining lymph nodes to present tumor antigen information to T cells, in which T cells are then activated and differentiated into antitumor effector T cells [4]. Due to the influence of tumor microenvironment on DCs metabolism, DCs in tumor tissues are mostly immature [5]. Thus, targeting dendritic cells (DCs) metabolism-related pathways and in-situ activation of DCs have become a new trend in DC-based immunotherapy [6].

As traditional Chinese medicine, the fruits of *Lycium barbarum* are officially listed in the Chinese pharmacopeia. Polysaccharides are bioactive constituents of *Lycium barbarum* and have many biological activities, including antitumor and immunomodulatory effects. Promoting the functional maturation of dendritic cells is one of the important immunomodulatory effects of *Lycium barbarum* polysaccharides (LBP). Studies have indicated that LBP could promote the maturity of murine DCs maybe through TLR4-Erk1/2-Blimp1 and TLR2/TLR4-MyD88-NF-*κ*B/p38 and Notch signaling [7–10]. Furthermore, LBP could strengthen DC mediated T lymphocyte cytotoxicity [10] and enhance the antitumor immune response in tumor-bearing mice [11]. However, it is not clear now which metabolic pathways are involved in the promotion of DCs maturation by LBP.

Based on the complexity of the multitarget and multipathway of traditional Chinese medicine, traditional research methods and analysis techniques are not enough to explain the specific mechanism of the detected biomarkers. Metabolomics as a cutting-edge analysis technology is widely used in the pharmacological research of traditional Chinese medicine by detecting and screening out statistically significant metabolites from biological samples [12]. In this way, metabolomics was used to investigate the effects of *Lycium barbarum* polysaccharides on the metabolism of dendritic cells in the present study.

## 2. Materials and Methods

### 2.1. Reagents

LBP, also named LBP3, were prepared as be described in our previous study with molecular weights range from 40 kDa to 350 kDa and the level of lipopolysaccharide was under the detection limit [13, 14]. DMEM were obtained from Gibco (USA, Lot No. 8119929). PBS buffer 1× was purchased form Wuhan Boshide Biological Engineering Co., Ltd. (Wuhan, China, Lot No. 14I17B30). Fetal bovine Serum (FBS) was obtained from Biological Industries (Israel, Lot No. 1924622). Methanol (AR) was purchased from Shanghai Anpu Experimental Technology Co., Ltd. (Shanghai, China, Lot No. Z5400144). Acetonitrile (AR) was purchased from Shanghai Anpu Experimental Technology Co., Ltd. (Shanghai, China, Lot No. C3010432). Ammonium acetate (AR) was purchased from Tianjin Zhiyuan Chemical Reagent Co., Ltd. (Tianjin, China, Lot No. 201840182077). LK16-HF90 CO_2_ Incubator was purchased from Beijing Zhongxin Huada Technology Co., Ltd. (Beijing, China). SW-CF-2FD Ultra-clean table was purchased from Jiangsu Sujing Antai Co., Ltd. (Jiangsu, China). Fresco17 high speed refrigerated centrifuge was obtained from Thermo (USA). Pipettes were obtained from Thermo (USA, range 1000, 200, 100, 10 *μ*L). DHP-9272 electronic analytical balance was purchased from Sartorius Scientific Instrument (Beijing) Co., LTD. (Beijing, China). Spectramax Plus 384-plus Microplate reader was obtained from MD (USA).

### 2.2. Cell Culture

Mouse bone marrow derived dendritic cell line DC2.4 was donated by Professor Zhou Lian of Guangzhou University of Chinese Medicine and purchased from Qingqi (Shanghai) Biotechnology Development Co., Ltd. (Shanghai, China). Cells were cultured in DMEM medium containing 10% FBS, 100 *μ*g/mL streptomycin and 100 U/mL penicillin in a cell incubator where the condition is maintained at 37°C with 5% CO_2_.

### 2.3. Cell Activity Assay

DC2.4 cells were seeded into 96-well plates at a density of 3 × 10^5^ cells/mL (100 *μ*L for each well) and cultured for 24 h before discarding the medium. Complete culture medium containing different concentrations of LBP (0, 50, 100, 200 *μ*g/mL) was added, respectively, and cultured for 24 h. The supernatant was discarded. 100 *μ*L basal medium and 10 *μ*L MTT solution (5 mg/mL) were added into each well and continued to culture for 4 h. 150 *μ*L dimethyl sulfoxide were added to each well. Plates were shaken on a shaking table for 10 min to fully dissolve the crystals. The absorbance (Ab) at 450 nm and 630 nm of each well was measured using the microplate reader. The following formula was used to calculate cell activity: relative cell relative activity (%) = mean Ab (450 nm − 630 nm) value of polysaccharide group/mean Ab (450 nm − 630 nm) value of control group × 100%.

### 2.4. Cytokine Detection

DC2.4 cells were treated as previously mentioned in the present study. After supernatant was collected, the levels of IL-6, IL-12, and TNF-*α* were assayed by enzyme-linked immunosorbent assay (ELISA) kits according to user instructions.

### 2.5. Sample Processing for Metabolomic

DC2.4 cells were seeded into 6-well plates and treated with LBP concentration of 100 *μ*g/mL. The cell culture medium was discarded. The plates were washed with precooled PBS for two times, and then washed with precooled normal saline (0.9% sodium chloride solution) for one time. The supernatant was completely discarded after each cleaning, and 1 mL ultrapure water was finally added. 100 *μ*L sample was collected into a 1.5 mL centrifuge tube, 1000 *μ*L extract (methanol: acetonitrile; water=2 : 2 : 1, V/V) was added, and mixed by shock. The samples were ultrasonically treated with ice water bath for 10 min, quick-frozen with liquid nitrogen for 1 min, repeated three times, and placed at -20°C for 1 h. After centrifugation at 13000 *r* and 4°C for 15 min, the supernatant was taken and dried with a nitrogen blower. 100 *μ*L extract (acetonitrile: water=1 : 1, V/V) was added and the sample was shaken for 30 s. The sample was treated with ice water bath ultrasound for 10 min, and centrifuged at 13000 r under the condition of 4°C for 15 min. The supernatant was taken and prepared for testing.

### 2.6. UPLC-Q-TOF MS Conditions

Liquid phase conditions were shown as follows: UPLC BEH Amide Column (2.1 mm ×100 mm, 1.7 *μ*m, Waters, USA); injection volume 5 *μ*L; column temperature 55°C; mobile phase A-100% H_2_O, B-100% ACN. Gradient elution conditions were as follows: at 0-1 min, 85% B; at 1-12 min, 65% B; at 12-12.1 min, 40% B; at 12.1-15 min, 40% B; at 15-15.1 min, 85% B; at 15.1-20 min, 85% B. The flow rate was 0.3 mL/min. Mass spectrometry conditions were as follows: UPLC-Q-TOF/MS (ESI) electrospray ionization source (X500R, AB SCIEX, USA); ion source temperature 600°C; ion source voltage -4500 V or 5500 V; curtain gas 20 psi, atomized gas and auxiliary gas both 60 psi. Multiple responses monitoring (MRM) was used for scanning.

### 2.7. Statistical Processing

UPLC-Q-TOF/MS technology was used to collect atlas information, and peak recognition, peak matching, peak retention time (RT) and quality (M/Z) data compared in XCMS and VGDB. The 3D data information was imported into R software. The clustering information and important variables were obtained by principal component analysis (PCA) and partial least squares discriminant analysis (PCA-DA). The PLS-DA model was used to calculate the VIP values of each variable in the samples. The differential metabolites between groups at different time points were screened according to VIP> 1.0. The data were normalized and log transformed, and the *P* value was calculated by *t*-test. When there was no biological duplication, only the fold change was calculated. Metabolites with log2 fold change ≥1 and *P* ≤ 0.05 were selected as the final differential metabolites. Qualitative analysis was performed on the primary and secondary spectrum data of mass spectrometry detection based on the Very Genome Database (VGDB), and material analysis was also performed by referring to other public mass spectrometry databases, including MassBank, METLIN, HMDB, and MONA. Finally, metabolic pathway analysis was performed by MetaboAnalyst 5.0 (https://www.metaboanalyst.ca/).

## 3. Results

### 3.1. Effect of LBP on DC2.4 Cell Activity and Cytokine Secretion

**Figure 1:**
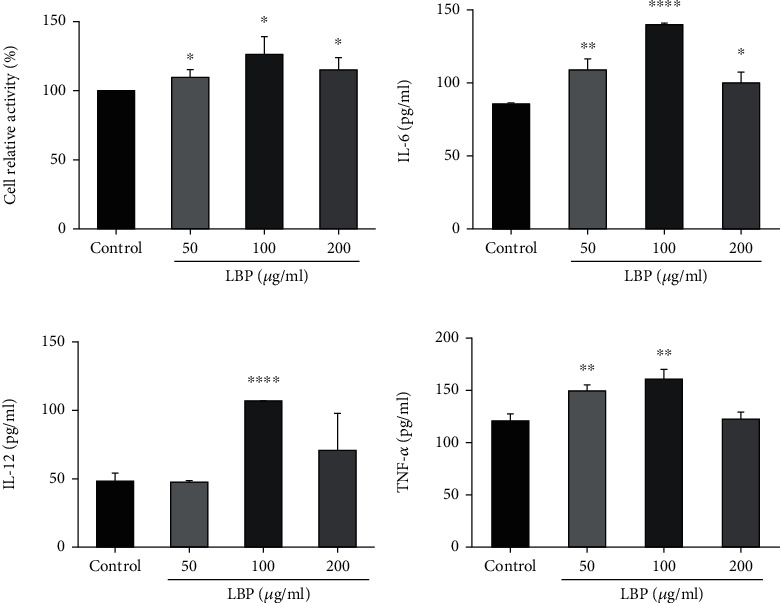
The effect of *Lycium barbarum* polysaccharide (LBP) on activity and cytokine production of DCs. (a) LBP had no significant inhibitory effect on DC2.4 cells. The cells were inoculated into 96-well plates and cultured in complete medium with different concentrations of LBP (0, 50,100, 200 *μ*g/mL) for 24 h. (B-D) LBP induced the production of IL-6, IL-12, and TNF-*α* in DCs. ELISA kits were used to detect the levels of IL-6, IL-12, and TNF-*α*. Data were expressed as mean ± SD, *n* = 3 for each group. ^∗^*P* < 0.05, ^∗∗^*P* < 0.01, ^∗∗∗∗^*P* < 0.0001 versus Control group.

The effect of LBP on DC2.4 cell activity was measured by MTT assay. When the concentration of LBP was 50, 100 and 200 *μ*g/mL, there was no significant inhibitory effect on DC2.4 cell (Figure 1(a)). On the contrary, LBP increased the cell activity significantly. Dendritic cells will secrete some cytokines during maturation, such as IL-6, IL-12, and TNF-*α*. In the present study, the results showed that LBP could promote the secretion of IL-6, IL-12, and TNF-*α*. In particular, LBP with the concentration of 100 *μ*g/mL had the greatest promotion effect on the secretion of IL-6, IL-12, and TNF-*α* (Figure 1(b)–1(d)). Therefore, the concentration of LBP used in the subsequent metabolomics study was 100 *μ*g/mL.

### 3.2. Method Stability Analysis

**Figure 2:**
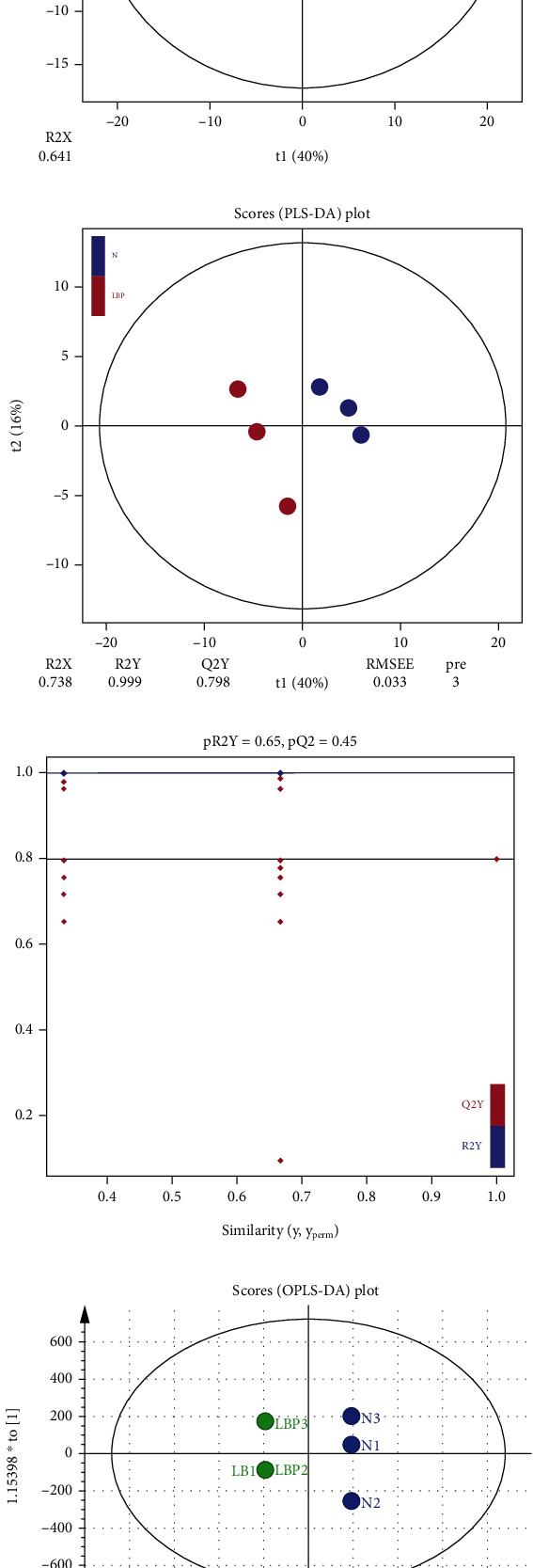
The scores plot of PCA, PLSDA and OPLS-DA. (a) PCA analysis. (b) PLS-DA model. (c) OPLS-DA score diagram and (d) load diagram.

The intracellular metabolites were analyzed by UPLC-Q-TOF-MS in both positive and negative ion modes in the present study. The total ions chromatogram is shown in Figure S1 and Table S1 and S2. The further detailed of global metabolic differences between the control group and LBP group were performed by multivariate statistical analysis. Firstly, principal component analysis (PCA) method and partial least-squares discriminant analysis (PLS-DA) model was used. The PCA scores plot (Figure 2(a)) showed good separation between the control group and LBP group, which provided a visual overview of the raw data without human influence. Then, PLS-DA was used to determine whether the model was reliable and over-fitting according to R^2^ and Q^2^ in the results. The two groups of samples in the score chart of PLS-DA model (Figure 2(b)) could be distinguished, with R^2^Y (cum) = 0.999 and Q^2^ (cum) = 0.798. It is considered that the model was reliable and had good interpretation ability of original data without over-fitting. Furtherly, in order to improve the analytical ability and effectiveness of the PLS-DA model, orthogonal PLS-DA (OPLS-DA) model was established to obtain score graph (Figure 2(c)) and draw load graph (Figure 2(d)) according to VIP, both which showed that there were significant differences in potential biomarkers between control group and LBP group.

### 3.3. The Identification of Significantly Differential Metabolites

**Figure 3:**
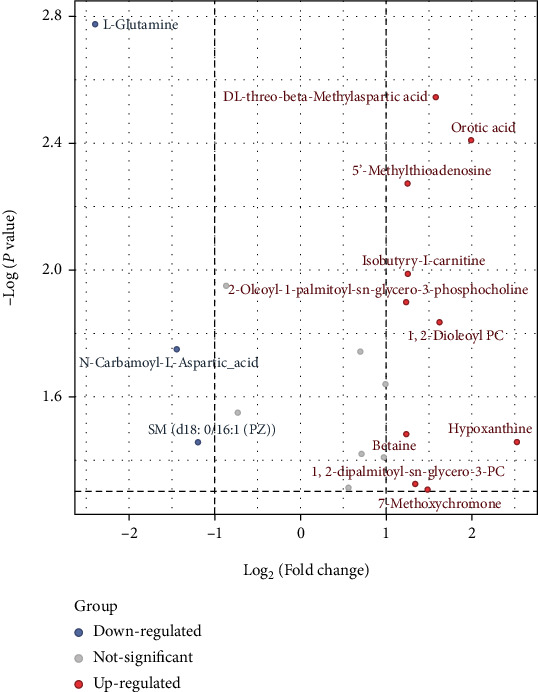
The volcanograms for identifying potential metabolites in LBP-induced DCs. The red dot is the upregulated metabolites with FC > 1.0 and *P* < 0.05, and the blue dot is the downregulated metabolites with FC> 1.0 and *P* < 0.05.

**Figure 4:**
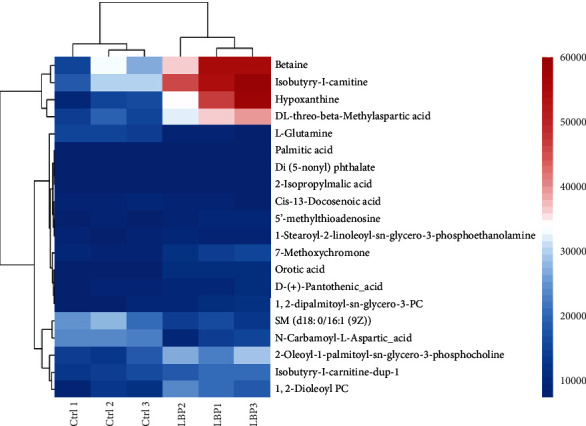
The heat map of the distribution of metabolites in LBP-induced DCs. Each small square represents a potential metabolite, and its color indicates the expression level of this metabolite. The greater the expression level, the darker the color (red represents upregulation, blue represents downregulation).

For the samples of control group and LBP group, the metabolites with log2 fold change ≥1 and *P* value ≤0.05 were selected as the final differential metabolites. A total of 20 potential metabolites were screened from the raw pool as shown in Table 1. Among them, 10 metabolites were upregulated and 3 were downregulated. According to the variation of differential metabolites, volcanograms was further used to screen the differential metabolites as shown in Figure 3. The red dot is the upregulated metabolite with FC> 1.0 and *P* < 0.05, and the blue dot is the down-regulated metabolite with FC> 1.0 and *P* < 0.05. To help quickly and intuitively identify potential biomarkers that vary widely and were statistically significant, the samples of each group were hierarchically clustered by using the expression of qualitative significant difference metabolites, and were represented by heat map (Figure 4). Each small square represents a potential metabolite, and its color varies with the concentration of metabolite. In this way, the changed of metabolite concentrations of LBP group versus control group could be found clearly, such as betaine, hypoxanthine, and DL threonine-beta-methylaspartic acid increased significantly in LBP group while L-glutamine decreased significantly. These results indicate that LBP has significant effects on dendritic cell metabolism.

### 3.4. KEGG Path Analysis

**Figure 5:**
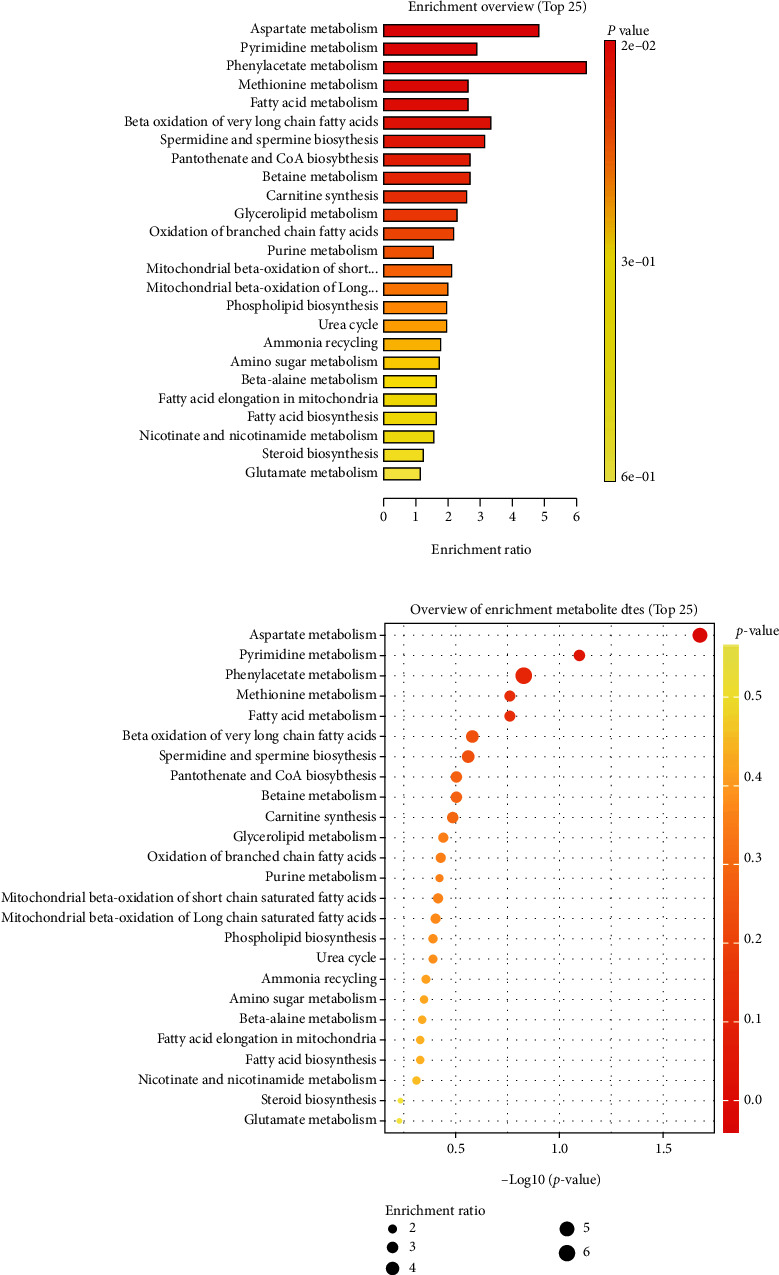
Metabolic pathways enrichment analysis of differential metabolites was conducted based on KEGG database. (a) Bar chart and (b) Bubble chart.

To identify metabolic pathways affected by LBP in DCs, the significantly different metabolites which were found between LBP group and control group were analyzed using MetaboAnalyst. In the present study, the critical value of the influence value of metabolic pathway is set to 0.10. If it is higher than this threshold, this path will be selected as a potential target path. In this way, there were 28 metabolic pathways were found and summarized in Table 2. The top 25 metabolic pathways with *P* < 0.6 as shown in Figure 5, among which the top 5 metabolic pathways were aspartate metabolism (*P* = 0.021), pyrimidine metabolism (*P* = 0.08), phenylacetate metabolism (*P* = 0.148), methionine metabolism (*P* = 0.173) and fatty acid metabolism (*P* = 0.173).

## 4. Discussion

Current studies suggest that polysaccharides from traditional Chinese medicine may directly target DCs, activate and promote cell maturation, thereby affecting the immune response [15]. DCs are known to be the most effective and powerful antigen presenting cells in the body, and they are also the only full-time antigen presenting cells that can activate initial T cells [16, 17]. DCs release cytokines during activation and maturation, such as TNF-*α*, IL-6, IL-12, and chemokine ligands [18, 19]. Our study showed that LBP could significantly increase the activity of DC2.4 cells and stimulate the cells to secrete IL-6, TNF-*α*, and IL-12. Consistent with the previous studies [7–10], the results indicated that LBP promoted DCs maturation in the present study. However, we found it strange that the effect of LBP on TNF-*α* release of DCs showed little effect at the higher dose of 200 *μ*g/mL. We speculate that this may be due to the existence of a negative feedback regulation mechanism on TNF-*α*.

To further investigate which metabolic pathways are involved in the promotion of DCs maturation by LBP, UPLC-Q-TOF-MS technology was used to determine DC2.4 cells metabolites in the present study and then the underlying metabolic pathways were analyzed. Our study showed that LBP could change 20 metabolites in DC2.4 cells, such as betaine, hypoxanthine, DL threonine-beta-methylaspartic acid, and L-glutamine. Among these metabolites, hypoxanthine, and L-glutamine had been shown to be involved in DCs activation and functional maturation [20]. The results suggest that LBP can affect DCs metabolism. Further pathway analysis suggested that these metabolites were involved in 28 metabolic pathways, such as aspartate metabolism, pyrimidine metabolism, phenylacetate metabolism, methionine metabolism, and fatty acid metabolism. Most of these pathways have been confirmed to be closely related to DCs activation, maturation, and T cell priming. For example, the aspartate metabolism is involved in mitochondrial respiration [21], and also plays a role in maturation and T cell priming of DCs by the IMP-S-AMP-MP cycle [20]. Fatty acid metabolism is involved in the glucose metabolism which is crucial for toll-like receptor (TLR)-induced activation of DCs [22], while inhibition of fatty acid metabolism could suppress DCs activation [23]. The inhibition of antigen-presenting activity of DCs resulting from UV irradiation of murine skin is restored by *in vitro* photorepair of cyclobutane pyrimidine dimers [24], this indicated that pyrimidine metabolism may involve in DCs metabolism.

In summary, the present study indicated that LBP promoted DCs maturation by the regulation of cell metabolism. Our previous study found that LBP could induce antitumor immune response and inhibit tumor growth in H22 tumor-bearing mice [11], so we speculated that these might be related to the regulation of DCs metabolism by LBP.

## Figures and Tables

**Table 1:** Cell significantly different metabolites.

NO	Compound name	Chemical formula	M/Z	Trend
1	Betaine	C_5_H_12_NO_2_	118.1543	↑
2	Hypoxanthine	C_5_H_4_N_4_O	136.1115	↑
3	DL-threo-beta-Methylaspartic acid	C_5_H_9_NO_4_	147.13	↑
4	L-carnitine	C_7_H_16_NO_3_	162.2068	↑
5	7-Methoxychromone	C_10_H_9_O_3_	177.17	↑
6	D-(+)-Pantothenic_acid	C_9_H_17_NO_5_	219.235	—
7	Isobutyry-l-carnitine	C_11_H_22_NO_4_	232.299	—
8	5′-Methylthioadenosine	C_11_H_15_N_5_O_3_S	297.334	↑
9	Cis-13-Docosenoic acid	C_22_H_42_O_2_	338.5677	—
10	Di(5-nonyl) phthalate	C_28_H_46_O_4_	446.662	—
11	SM(d18:0/16 : 1(9Z))	C_39_H_79_N_2_O_6_P	703.0281	↓
12	1,2-dipalmitoyl-sn-glycero-3-PC(DHPE)	C_37_H_74_NO_8_P	691.972	↑
13	1-Stearoyl-2-linoleoyl-sn-glycero-3-phosphoethanolamine(1,2-dop/1,2-dielaidoyl)	C_41_H_78_NO_8_P	744.034	—
14	2-Oleoyl-1-palmitoyl-sn-glycero-3-phosphocholine(POPC)	C_42_H_82_NO_8_P	760.0761	↑
15	1,2-Dioleoyl PC(DOPC)	C_44_H_84_NO_8_P	786.1134	↑
16	L-glutamine	C_5_H_10_N_2_O_3_	146.1445	↓
17	Orotic acid	C_5_H_4_N_2_O_4_	156.0963	↑
18	N-carbamoyl-L-Aspartic_acid	C_5_H_8_N_2_O_5_	176.1274	↓
19	2-Isopropylmalic acid	C_7_H_12_O_5_	176.1672	—
20	Palmitic acid	C_16_H_32_O_2_	256.424	—

**Table 2:** Analysis of cellular metabolic pathway based on metaboanalyst.

NO	Pathway name	Total	Expected	Hits	Raw p
1	Aspartate metabolism	35	0.615	3	0.021
2	Pyrimidine metabolism	59	1.04	3	0.08
3	Phenylacetate metabolism	9	0.158	1	0.148
4	Methionine metabolism	43	0.756	2	0.173
5	Fatty acid metabolism	43	0.756	2	0.173
6	Beta oxidation of very long chain fatty acids	17	0.299	1	0.262
7	Spermidine and Spermine biosynthesis	18	0.316	1	0.275
8	Pantothenate and CoA biosynthesis	21	0.369	1	0.313
9	Betaine metabolism	21	0.369	1	0.313
10	Carnitine synthesis	22	0.387	1	0.326
11	Glycerolipid metabolism	25	0.439	1	0.362
12	Oxidation of branched chain fatty acids	26	0.457	1	0.373
13	Purine metabolism	74	1.3	2	0.378
14	Mitochondrial Beta-oxidation of short chain saturated fatty acids	27	0.475	1	0.384
15	Mitochondrial Beta-oxidation of long chain saturated fatty acids	28	0.492	1	0.395
16	Phospholipid biosynthesis	29	0.51	1	0.406
17	Urea cycle	29	0.51	1	0.406
18	Ammonia recycling	32	0.562	1	0.438
19	Amino sugar metabolism	33	0.58	1	0.448
20	Beta-alanine metabolism	34	0.598	1	0.458
21	Fatty acid elongation in mitochondria	35	0.615	1	0.468
22	Fatty acid biosynthesis	35	0.615	1	0.468
23	Nicotinate and nicotinamide metabolism	37	0.65	1	0.487
24	Steroid biosynthesis	48	0.844	1	0.582
25	Glutamate metabolism	49	0.861	1	0.589
26	Warburg effect	58	1.02	1	0.653
27	Glycine and serine metabolism	59	1.04	1	0.66
28	Bile acid biosynthesis	65	1.14	1	0.696

## Data Availability

The underlying data supporting the results of our study were available on request. The corresponding author (Deng) is contacted to request the data.

## Supplementary Materials

Supplementary Materials Figure S1: the total ions chromatogram in negative ion and positive ion mode. Table Sl: the raw data of negative ion mode. Table S2: the raw data of positive ion mode. (Supplementary Materials).
