# Supplementary material for: Effects of Lycium Barbarum Polysaccharides on the Metabolism of Dendritic Cells: An In Vitro Study
Source: J Immunol Res. 2022 Oct 19;2022:5882136. doi: 10.1155/2022/5882136 (PMC9605842; doi:10.1155/2022/5882136)
Supplement: Supplementary Materials — Figure S1: the total ions chromatogram in negative ion and positive ion mode. Table Sl: the raw data of negative ion mode. Table S2: the raw data of positive ion mode. (Supplementary Materials). [file 5882136.f1.zip › Supplementary data (Table S1).pdf]

Supplementary data (Table S1): Raw data of negative ion mode

ID	mz	RT	LBP1	LBP2	LBP3	Ctrl1	Ctrl2	Ctrl3	QC1	QC2	QC3
1	57.9748	59	194.092	587.02	157.705	506.113	621.392	181.5543	1093.94	217.957	102.564
2	59.0131	835	1403.21	2068.7	1833.38	2092.06	2197.6	1986.446	2994.25	1903.49	2049.89
3	59.0131	112	1617.25	1412.3	1057.85	2362.13	2228.52	1625.776	3331.37	1642.83	1294.62
4	59.0132	990	6757.71	6085.7	6173.05	10255.1	9042.53	8288.777	13051.5	7106.55	5937.99
5	59.0132	955	10445.8	6087.6	9190.53	14733.3	12964.3	11387.86	18777.5	10128.6	9269.26
6	59.0132	73	1465.68	1945.5	1243.95	2800.27	2203.94	1403.584	5031.64	1323.08	1148.32
7	59.0133	1018	21975.2	33457	25737.1	29356.6	58158.2	36308.46	40919	20228.9	26749.8
8	59.0133	923	28778	6324.2	18882.3	11336.1	9745.37	7930.751	10150.3	7127.28	6257.85
9	59.0133	356	669.843	702.3	643.791	1338.11	958.754	827.1091	1669.64	741.49	552.254
10	59.0133	243	1637.21	1519.6	1341.71	1738.78	1649.17	1374.23	4162.19	1193.26	1289.86
11	59.0133	6	1682.36	1533.1	1010.54	1792.55	1817.09	1765.167	2939.53	1226.67	1127.23
12	59.0134	771	2668.56	1324.9	1850.93	2087.01	2485.13	2213.704	3536.84	1632.46	1717.93
13	59.0134	137	2253.94	2161.7	1993.63	615.969	3780.56	2234.48	6323.04	1782.98	1848.29
14	59.0134	46	999.692	1426.8	605.007	1377.87	1594.48	828.4266	2413.94	803.85	1106.41
15	59.0135	101	2133.39	1483.7	1167.78	3101.64	2437.74	1785.821	4872.82	1585.94	1399.47
16	59.0136	1183	1186.37	577.44	871.105	1229.02	634.684	869.04	569.736	726.257	730.586
17	59.0136	338	750.388	700.91	710.488	881.632	967.368	930.1421	1709.75	772.254	569.083
18	59.0136	227	579.036	715.7	457.403	724.953	666.996	347.8885	1493.38	552.538	592.249
19	59.9846	802	4064.4	2451.5	1309.27	2604.68	2014.17	1647.019	1604.66	1779.94	3549.1
20	59.9846	681	390.166	495.41	422.856	519.502	453.787	457.5008	748.748	713.532	469.041
21	59.9846	528	421.276	652.47	1183.41	459.057	332.994	348.0059	612.24	368.652	361.504
22	59.9846	497	2502.51	1270.8	961.111	1236.44	1242.52	834.4601	1519.7	701.897	1024.7
23	59.9846	98	6070.56	5207.1	2955.63	8278.9	6158.34	4756.822	6072.47	3789.87	4265.81
24	59.9847	1179	5340.27	2158.4	2554.9	3383.86	1773.47	1625.07	2023.39	2701.55	4176.05
25	59.9847	1093	3969.91	1707.4	3120.27	1943.74	3982.74	2700.062	2542.03	2033.11	1544.72
26	59.9847	862	2874.42	2903.4	1884.36	2157.74	1522.24	3892.794	3173.4	3276.74	2334.1
27	59.9847	835	2987.86	4131.6	3572	2349.42	2546.93	3315.181	2825.72	3828.69	4615.58
28	59.9847	744	2979.82	1184.3	2754.06	7548.06	1362.97	1490.607	2399.61	1842.68	1411.46
29	59.9847	601	1539.19	1083.9	1256.17	1770.9	993.377	1058.747	1156.98	2567.54	1295.33
30	59.9847	559	2900.23	974.12	1203.48	1688.39	1186.74	1182.008	1933.21	1233.87	1001.2
31	59.9847	261	2326.56	2062.9	2145.11	2650.16	2134.31	2892.784	6048.29	3140.52	1582.55
32	59.9847	226	4894.9	4514.3	3615.3	6205.33	3452.99	3340.446	8314.56	3239.63	4263.1
33	59.9847	161	7473.25	1990.9	2248.16	3631.48	4228.2	4534.691	7060.93	4193.54	2576.06
34	59.9847	137	9779.66	6689.3	5102.02	20155.1	10592.6	7993.477	13693.5	7028.9	4624.33
35	59.9847	118	5354.39	5840.1	3065.17	6979.12	5415.59	5005.09	6041.93	4622.25	4146.88
36	59.9847	49	2264.13	5260.5	1037.91	2430.68	4641.08	2443.826	4015.53	2327.04	4181.59
37	59.9848	1196	2553.94	1834.8	1382.21	1477.52	0	2838.369	4095.63	2744.82	2809.3
38	59.9848	1057	7792.33	5008.5	11483.6	6290.05	7028.73	3566.271	7317.03	6159.73	8699.8
39	59.9848	820	2569.24	1616.7	1782.28	1874.57	1691.56	281.8725	2640.77	1744.02	2444
40	59.9848	782	3277.49	1287.1	2858.45	2199.43	6374.2	2106.091	2988.39	3641.2	2329.94
41	59.9848	768	2795.8	3596.6	3149.76	1572.19	3402.88	2124.677	2036.68	2562.93	2610.54
42	59.9848	437	2182.76	1072	1587	1955.1	1285.15	1565.688	2573.95	1482.15	1423.14
43	59.9848	331	2476.01	1288.2	1969.6	2990.85	2721.28	2164.842	5095.06	1802.55	208.315
44	59.9848	277	2608.52	1948.4	2536.32	3454.33	2692.63	2670.213	2632.75	2240.3	2094.86
45	59.9848	181	5632.88	3948.8	4564.7	2849.44	4306.26	3217.424	6966.22	3732.11	3205.45
46	59.9848	72	4609.13	16669	2410.99	6037.66	7488.89	3930.007	8398.16	4535.37	4530.61
47	59.9848	34	2408.94	1268.8	2715.92	3947.94	1280.76	2550.83	2307.99	1292.74	990.074
48	59.9848	9	6177.44	4267.5	2261.63	7267.55	3126.38	6122.714	4103.4	2791.29	3996.07
49	59.9849	867	1821.46	888.84	2020.61	2323.25	1956.24	1688.153	2621.07	1869.57	1341.42
50	59.9849	638	1064.92	1121.5	1288.39	1751.57	850.904	1015.077	1416.25	1189.95	1080.33
51	59.9849	361	866.109	870.87	860.092	3272.04	1164.9	1328.175	2628.17	977.15	816.728
52	59.985	1167	1460.89	1670.6	1179.07	1254.56	1302	1818.91	1561.86	2255.85	2138.46
53	59.985	710	879.907	689.3	875.279	750.052	792.26	837.1176	961.679	909.667	750.584
54	61.9878	125	16297.7	7555.1	23057.5	33227.7	23623.7	19823.15	68753.3	23091.3	12593.1
55	61.9879	53	14771.2	13798	12141.4	39138	38185.7	14125.53	47667.8	27835.5	10779.5
56	61.988	5	5896.69	4926.7	6007.94	13207.5	9104.24	6463.914	12312.8	10765.6	3904.45
57	61.9881	148	13928.7	10020	13275.2	22828.9	16722.9	14993.05	59924	19158.7	9155.83
58	68.9948	2	48.7153	22.713	64.2143	119.539	42.112	37.80367	270.433	90.298	9.7845
59	75.0079	240	241.993	190.7	265.208	380.582	408.253	271.414	1689.15	406.452	174.086
60	75.0083	341	167.164	231.55	174.736	394.31	342.057	262.0334	872.685	257.691	150.799
61	75.0083	277	236.493	205.54	316.796	493.272	425.924	400.4115	1512.96	391.181	209.231
62	75.0084	319	432.88	449.53	452.204	929.272	692.507	674.228	2057.19	525.286	309.555
63	75.0084	297	258.257	240.19	299.758	483.492	389.427	316.1763	1375.79	320.227	197.508

64	75.0084	224	332.23	316.81	395.189	643.323	512.45	438.7413	2240.61	583.488	243.083
65	75.0086	86	757.704	733.22	784.668	2245.69	1365.13	910.0332	5872.09	661.761	448.544
66	82.9717	72	69.6267	92.458	109.532	175.719	214.14	116.5193	919.558	131.658	53.03
67	84.0083	243	130.891	113.55	173.476	196.017	211.467	172.1886	1063.89	216.946	84.5938
68	84.0087	218	34.128	30.959	50.4432	70.598	42.7342	48.77127	353.094	61.744	18.7
69	84.0087	197	245.261	203.02	235.859	375.49	289.924	260.8198	1694.19	389.508	149.156
70	84.0087	183	12.6267	6.08	14.036	0	0	16.50133	113.174	25.5733	5.12
71	84.0089	256	106.843	95.756	128.251	181.522	154.512	159.3856	740.75	147.442	55.14
72	84.0094	1	0	100.19	0	0	282.567	0	1271.39	0	91.0865
73	85.0035	1136	373.662	280.25	326.646	679.76	543.813	449.4277	773.651	395.762	297.737
74	85.0035	13	1534.6	1017.5	1233.95	3230.53	1986.82	1462.195	8361.61	1389.4	799.483
75	85.0036	1147	428.825	330.14	373.455	702.266	709.266	614.7273	1090.37	488.565	410.091
76	85.0037	1125	462.138	383.67	381.337	726.594	811.045	603.225	1244.82	533.193	485.444
77	85.0037	377	555.227	526.78	549.273	1374.46	1009.99	814.7104	3130.56	739.555	468.677
78	85.0037	338	354.883	361.19	350.769	719.959	766.147	605.002	2094.05	427.292	275.412
79	85.0037	272	418.416	309.16	476.518	950.962	632.4	683.8623	3094.11	653.776	282.142
80	85.0038	394	230.24	217.2	184.442	514.113	389.211	335.6893	1163.1	282.552	128.666
81	85.0038	233	696.6	684.84	781.94	1161.53	1099.38	847.3634	6218.71	1223.46	549.646
82	85.0038	175	819.78	554.36	837.253	1738.81	1010.73	1081.627	5800.71	1296.39	478.213
83	85.0038	122	5685.22	4305	5103.78	12874.6	9295.26	6594.477	34291.4	8288.97	3621.7
84	85.0038	100	2929.99	2852.2	3186.22	8764.81	6366.91	4236.09	20894.1	4448.95	2139.17
85	85.0038	30	1284.09	891.86	1095.86	2794.84	1627.65	1178.335	7501.65	1118.91	662.155
86	85.0039	357	170.06	183.77	200.196	451.5	342.442	307.4206	1083.49	270.37	103.698
87	85.0039	320	439.828	431.59	461.978	1066.76	850.125	775.8248	2947.99	675.524	337.874
88	85.0039	254	784.962	719.03	864.664	1661.2	1355.76	1216.82	7054.82	1361.58	567.455
89	85.0039	204	1017.13	797.37	1103.85	1958.79	1467.32	1188.276	8093	1865.08	663.238
90	85.0039	146	1000.69	532.38	727.622	1857.58	1078.8	1092.518	5753.83	1475.91	558.076
91	85.004	296	606.504	524.88	683.256	1346.96	1018.79	928.0495	3982.22	781.943	443.155
92	85.0041	2	0	655.66	0	0	1714.8	0	5588.76	0	510.349
93	87.0081	2	0	61.039	0	0	147.64	0	932.792	0	40.3442
94	87.0089	87	94.6734	94.309	152.732	492.445	356.762	150.9795	1398.73	171.663	40.3008
95	88.0395	75	164.235	104.06	273.868	518.526	342.44	204.253	2079.57	195.725	53.5756
96	88.0399	105	426.676	239.05	588.996	644.202	549.874	351.3896	2663.79	614.948	174.673
97	89.0239	65	1777.94	774.2	2583.39	3510.82	2816.7	1757.843	20698	2529.05	783.455
98	91.0032	146	1247.48	746.42	1172.01	3066.72	1812.75	1424.382	10689.7	2089.81	694.409
99	91.0032	98	1712.58	1577	2512.99	6274.37	4551.55	2739.481	24913.4	3290.17	966.99
100	91.0038	3	342.771	415.09	521.293	1278.76	847.504	506.2446	3126.71	1106.56	189.428
101	92.9274	71	9145.25	9026.1	10920	19162.7	18434.3	11458.46	113244	13934.3	5434.7
102	94.9244	71	5573.96	5646.7	6993.53	12499.9	12526.3	7422.399	81678.1	9964.42	3805.44
103	96.9214	69	950.056	929.05	1186.44	2016.97	2131.4	1302.898	14588.1	1806.98	548.815
104	98.9483	56	50.7739	277.31	89.0625	775.554	255.573	29.67714	1368.19	171.115	25.422
105	102.9558	145	213.439	108.36	138.349	419.793	263.459	204.4241	1573.31	423.04	101.277
106	102.9559	69	2680.43	1501.5	2949.74	6962.61	3496.29	2392.404	18267.6	3041.84	1273.44
107	104.0345	126	383.985	153.57	447.509	422.406	229.094	166.5451	1634.88	360.958	144.559
108	104.9533	70	1840.38	1067.7	1682.36	2981.4	2220.77	1099.515	7353.96	1479.44	866.249
109	108.9007	70	858.303	618.99	1254.47	925.365	825.202	683.9308	2397.49	896.145	598.923
110	110.898	69	989.521	825.86	1184.09	742.445	1010.92	700.149	2368.97	980.046	692.693
111	111.0136	70	2414.38	2485	3275.42	1831.95	3398.58	1152.635	5076.78	1566.93	1555.57
112	112.6328	88	1118.22	950.72	1070.09	1298.82	1223.07	912.0801	2063.77	1708.47	826.025
113	112.9852	84	126900	71657	121593	146820	120148	113190.8	438457	132353	80139.3
114	113.0348	72	1155.73	2404.2	1753.48	73857.7	54032.9	65387.93	1010.8	62359.2	1225.03
115	113.9881	84	4170.5	2359.9	3294.41	2984.07	2916.21	3434.601	14070.2	4166.7	2430.43
116	114.9877	968	1000	1042.4	982.603	1110.33	1095.62	1079.54	1089.39	571.246	620.718
117	114.9878	982	341.404	299.48	307.224	338.73	296.471	285.7092	345.388	876.621	74.812
118	114.9879	892	2844.11	4221.6	10051	5229.89	4271.64	5836.015	5329.13	2088.7	7162.99
119	114.9879	825	1801.69	1622.6	1163.72	1867.59	2091.59	2094.191	2253.92	1697.08	1525.26
120	114.9879	806	1489.31	1186.8	1530.81	1607.42	1770.21	1745.017	1931.79	1392.28	3318.1
121	114.988	930	1201.99	1111.1	1112.54	1272.03	1267.49	1199.533	1026.88	859.656	1191.62
122	114.988	845	812.748	669.24	769.092	930.953	895.517	854.6831	1054.76	1294.89	420.032
123	114.988	791	2370.27	765.12	1138.55	1042.67	1122	1573.123	1387.76	257.909	1609.27
124	114.988	774	985.433	803.96	1223.99	976.867	1166.32	1078.201	1395.1	1283.49	830.263
125	114.988	750	1064.88	545.97	655.153	571.215	571.2	653.0974	751.537	358.592	638.817
126	114.9881	947	512.148	491.37	456.803	587.838	526.906	522.836	264.767	727.61	1086.16
127	114.9893	84	1975.57	1773.6	2182.68	2115.51	4133.91	1923.846	5065.91	1883.86	1834.56
128	115.0031	217	657.207	570.34	558.677	765.406	714.145	543.462	1472.35	729.971	484.401

129	115.0031	74	2477.96	2190	2336.88	3555.85	3488.78	2660.064	9085.66	2677.97	2104.28
130	115.0032	191	725.363	689.84	698.674	811.294	738.824	790.8345	1788.61	860.48	581.376
131	115.9198	64	320.727	626.62	409.101	482.91	537.724	349.2284	1232.52	915.441	385.883
132	115.9199	976	187.059	218.16	188.133	339.743	289.873	265.9665	402.037	220.233	184.274
133	115.9199	933	259.941	284.72	261.301	401.54	328.374	329.4829	430.25	272.737	257.461
134	115.9202	952	394.528	374.71	318.65	589.412	505.532	464.3849	745.664	399.086	348.118
135	116.0344	67	1448.8	1324.2	1903.54	1269.77	1379.01	1196.488	2850.17	1826.08	1240.46
136	116.9274	919	250.051	299.1	287.984	364.25	381.427	355.2443	429.235	271.713	294.404
137	116.9277	993	485.231	421.19	437.515	666.922	639.424	618.1996	867.045	470.124	419.699
138	116.9278	1010	413.515	329.97	314.513	516.227	459.681	423.8139	628.16	338.555	303.683
139	116.9279	937	315.959	313.94	266.051	145.53	404.291	382.0421	997.295	333.529	306.589
140	116.928	953	547.944	565.21	509.906	896.959	769.953	654.5181	1017.43	577.284	517.938
141	117.0205	2	85.8536	80.955	188.589	246.424	141.134	158.6068	412.278	153.094	82.8302
142	118.0498	117	2623.03	1335.1	4091.01	1387.67	1747.15	1154.537	6839.46	2997.59	1692.23
143	118.93	76	490.432	177.87	483.723	396.248	199.307	248.2755	710.489	269.943	214.117
144	119.9463	64	2072.82	1783.5	2052.26	2574.32	4347.67	1856.53	7997.2	3154.33	2009.29
145	119.9465	118	676.687	976.43	824.035	1224.7	1123.42	994.627	2139.06	1198.07	800.818
146	121.0292	1	0	700.35	0	0	1329.74	0	3450.38	0	501.948
147	121.9433	63	657.726	668.61	830.672	1110.3	1512.66	813.2389	2594.74	1015.05	768.179
148	123.008	1	0	182.35	0	0	175.713	0	581.248	0	115.267
149	123.9011	22	700.015	608.18	717.474	836.444	706.933	673.7397	1323.78	682.682	591.854
150	123.9016	52	1219.08	988.5	1216.77	1432.91	1353.37	1335.209	1995.07	1364.39	1033.61
151	124.0067	70	40579.1	16328	49179.5	25066.5	26554	25722.89	57366.3	28243.8	25626.6
152	125.0097	63	1340.03	746.84	1659.61	994.982	967.949	966.8407	744.523	1134.32	854.004
153	126.0025	71	2237.93	1502.7	2312.16	1831.62	1784.15	1595.117	3124.51	2273.18	1767.32
154	126.9041	58	561.432	732.71	508.482	588.086	683.401	391.755	2221.57	1069.29	983.885
155	127.0006	84	3573.72	1007.3	3540.73	3546.35	1767.93	3141.178	8711.75	3479.39	1765.78
156	127.001	59	657.152	725.93	562.879	742.036	920.938	666.9306	1419.74	900.365	573.666
157	127.0499	92	2748.2	1851.9	2456.9	2839.77	2478.87	2415.449	7799	2628.29	1892.29
158	127.0506	160	313.059	321.23	354.304	387.951	425.082	398.4454	636.357	498.049	435.066
159	127.0509	144	549.289	446.29	557.055	787.003	831.099	770.5189	1330.38	938.205	741.545
160	127.051	118	1102.85	991.65	1256.44	1533.44	1794.37	2605.664	3758.21	1603.1	1438.04
161	128.0345	127	925.553	852.35	772.348	1509.26	1191.67	1236.178	2458.22	1290.58	1038.95
162	128.0347	69	14152.1	10359	13185	12672.4	13385.2	12506.22	25367.6	13136.1	11194
163	128.0348	279	2888.14	1289	3352.55	2749.93	1337.41	2604.785	3620.82	3011.8	213.035
164	128.0348	149	475.43	402.91	408.865	607.36	546.425	549.5968	1177.37	615.472	430.794
165	128.0349	250	7133.09	2670.2	4392.66	2998.4	2539.69	3044.801	7075.08	3724.29	2927.4
166	128.0349	227	3710.73	1906.6	1993.03	2582.23	3011.55	2644.677	4469.19	2565.69	2927.52
167	128.9586	81	3098.87	1365.9	4708.54	3464.65	2233.76	2603.148	9351.37	2475.38	2219.91
168	128.9588	60	1728.48	1549.8	1853.61	2135.09	998.165	1727.184	4276.29	1617.08	1224.9
169	129.0187	71	3104.77	3366.3	4524.94	5206.66	3555.15	2484.597	5766.03	3305.41	3097.28
170	129.0384	68	4111.98	3295.4	4847.16	884.294	4328.19	3903.308	1863.36	3451.86	2816.62
171	129.0551	63	1712.52	1299.4	1753.26	1702.55	1447.6	1321.074	3255.37	1766.1	1148.06
172	129.9749	61	907.884	751.68	944.229	1032.63	2133.39	853.4936	3534.2	1222.56	710.022
173	129.9751	92	2823.35	2460.5	2977.38	4024.28	1702.09	2932.629	9033.61	4084	2370.52
174	130.0616	97	4251.93	2453.7	3282.48	3012.6	1709.23	1674.841	6172.41	3127.34	2919.14
175	130.0867	62	2786.06	1790.4	3058.09	1488.74	1482.43	1418.932	1787.14	830.61	1547.59
176	130.9655	989	595.035	7542.3	8524.32	9149.81	8374.47	8885.243	8983.23	6832.96	8343.62
177	130.9656	343	1203.46	1229.4	1123.15	1696.33	1590.19	1583.099	1374.54	1280.5	1141.74
178	130.9656	214	1107.8	1006.2	924.105	1466.26	1277.74	1045.048	1498.1	968.051	973.005
179	130.9656	125	1549.77	1254.7	1377.03	2523.93	2078.27	3380.84	2282.52	1465.07	1420.79
180	130.9657	1165	2524.21	2248.5	2403.91	3632.5	2617.57	2612.107	2580.89	2263.84	2120.51
181	130.9657	1125	3984.19	5395.4	3755.06	4731.42	4893.36	4158.174	6386.09	5558.83	5606.39
182	130.9657	1084	1956.85	3501.2	3324.81	3322.75	3139	3981.876	3324.4	3436.56	2998.54
183	130.9657	892	7510.42	7536.9	6554.7	9036.27	8395.45	8078.685	8962.91	7431.08	6839.24
184	130.9657	237	1122.85	1051.5	938.69	1345.22	1196.13	1348.504	1975.37	1103.92	940.753
185	130.9657	156	1378.81	1363.6	893.574	1624.51	1505.89	1426.653	2070.72	1420.12	1085.21
186	130.9657	54	1848.21	1720.5	1311.63	1459.29	1980.52	2912.015	2895.44	1613.2	899.615
187	130.9659	1185	1376.02	1593.4	1684.22	2113.53	2469.83	2208.944	2050.05	2823.69	2122.16
188	130.9659	188	498.676	869.54	468.522	655.236	1011.96	615.7556	970.283	725.918	759.741
189	130.9659	14	2676.88	2780	1480.79	2673.72	2205.81	2300.582	2247.27	1283.85	2093.02
190	130.966	959	7539.88	39101	7081.97	7645.59	7579.31	8972.328	7224.14	7515.83	7644.08
191	130.966	282	1164.63	1174.5	1149.94	1532.63	1467.4	1542.594	1594.4	1212.22	1036.82
192	130.9661	324	915.598	1020.8	823.618	1171.48	1215.79	1211.165	1145.66	888.086	883.917
193	131.0348	64	2890.48	3025.8	3012.67	4171.8	4163.17	3802.074	6110.1	3665.23	2753.9

194	131.0351	1	0	1378.8	0	0	1705.74	0	1316.51	0	1095
195	132.0295	424	723.607	327.22	523.268	354.089	314.965	399.6963	600.766	449.839	341.713
196	132.0296	408	1290.04	675.46	960.812	700.658	709.561	831.5652	1306.59	941.779	711.129
197	132.0296	393	797.425	414.67	572.908	488.608	512.82	538.8794	920.509	586.895	431.806
198	132.0299	111	7017.77	3681.5	7617.1	747.583	737.115	574.5091	5281.31	3370.11	2260.09
199	132.03	374	663.916	332.66	486.809	481.19	434.782	452.9508	907.328	448.318	365.036
200	132.8669	167	6343.42	5070.5	7230.09	10188.6	5976.43	7994.256	10671.6	7342.67	3741.12
201	132.867	385	4981.16	6432.6	4980.6	6809.23	7676.53	6233.066	8162.75	6699.12	4917.65
202	132.867	231	7275.04	6296.6	5675.26	7576.44	8031.06	5542.066	12597.2	10500.9	6012.22
203	132.8671	458	6935.23	7257.9	6478.04	9555.42	8891.89	7811.791	9279.54	9114.35	6499.55
204	132.8671	339	8742.68	9476	9324.63	11131	13874.9	10291.29	10573.4	10691.8	8866.96
205	132.8671	313	7061.16	9595.5	7949.2	10761.2	8023.36	10231.61	864.389	8927.88	5537.16
206	132.8671	290	7171.94	7079.9	6131.93	9471.12	10579.7	7639.463	15217.5	9454.34	6501.58
207	132.8671	261	6657.26	6032.9	7238.34	9139.71	8635.22	7785.368	17618	8836.65	6962.08
208	132.8671	206	8619.88	7133	10472.1	10520.8	8852.65	10002.7	22515.3	11207.3	8493.97
209	132.8671	150	17428.3	7430.9	8329.63	13352.9	10984.3	10011.66	16665.1	12727.2	7577.59
210	132.8672	1194	7487.83	4094.2	5880.28	10769.6	0	4659.853	6642.84	2925.81	4145.77
211	132.8672	1173	7631.51	7841.8	5198.22	15208.1	15543.9	10112.09	9916.15	6164.66	10081.9
212	132.8672	1119	8279.58	8264.5	5667.73	11316.7	12233.8	7795.079	12210.9	10331.7	11133.7
213	132.8672	1081	8808.6	6835.6	7971.71	8425.46	7629.72	17350.51	8488.36	9577.1	8667.24
214	132.8672	1060	6356.72	5477.6	6230.58	8516.73	5935.64	5768.192	7559.93	6150.04	5547.45
215	132.8672	1034	13757	14332	16442.4	15445	8034.57	5994.037	6739.9	13054.2	21757
216	132.8672	602	3920.59	3999.5	3838.34	4949.75	4550.72	4387.171	4232.34	5141.21	3561.17
217	132.8672	547	7863.2	8436.8	8317.98	10328.9	9707.01	9406.545	10686.5	9505.42	6944.89
218	132.8672	474	5537.53	6103.8	4856.96	7467.88	7701.15	6863.27	9059.33	6541.41	5507.36
219	132.8672	182	4992.22	7502	5114.05	8841.39	12240.5	10178.13	11499.2	8757.13	5928.77
220	132.8672	126	6585.32	7842.8	6152.94	11484.2	12423.9	7815.458	17794.3	9521.05	6266.64
221	132.8672	54	10121.2	13691	11829.7	18245.9	20640.2	9520.643	36738	12261.6	15929.5
222	132.8672	25	9777.62	8870.7	10230.6	10747.6	8905.53	9431.932	24321.7	9504.83	7326.74
223	132.8673	1148	5705.11	5104.6	5831.86	6468.95	6181.15	6109.075	6769.9	8353.63	6152.69
224	132.8673	3	0	8393.7	0	0	8726.18	0	6308.05	5410.17	6442.93
225	133.0135	74	1677.67	2351.1	2390.1	6227.99	12867.8	3805.957	19050.9	2871.6	1525.25
226	133.0499	63	1634.9	2796.8	2227.25	5752.22	11946	3088.904	4750.03	2413.59	1605.18
227	133.0506	3	231.784	185.56	298.432	379.796	330.32	266.7203	862.945	292.135	148.551
228	134.0165	72	1107.6	901.27	1478.05	833.856	1893.46	1172.659	1141.73	1339.32	1135
229	134.0467	62	1099.8	928.06	1718.06	878.875	1298.65	1128.832	2118.08	1228.83	1123.96
230	134.8645	548	810.18	274.5	660.192	772.928	710.71	714	790.764	450.3	469.224
231	134.8645	534	5120.96	5503.6	4974.81	6671.23	6252.34	6168.026	6842.36	6032.51	4397.46
232	134.8645	337	7455.34	10280	9729.95	10575.4	10993.9	11005.32	12349.9	10885.3	9416.29
233	134.8645	314	9420.47	10855	11074.3	11413.1	11910.8	13419.31	15440	12918.4	8986.98
234	134.8646	1197	8039.19	3444.9	6885.38	12752.2	0	5235.074	6688.7	2543.74	3463.84
235	134.8646	1123	5169.95	11076	8595.03	4635.49	13001.1	6802.786	9635.34	11187	9671.44
236	134.8646	1099	5452.24	7653.9	5587.54	6299.86	9201.26	10454.65	9284.37	7908.8	7746.81
237	134.8646	1079	7682.95	8053.8	7592.38	10978.2	9341.65	8488.978	8666.52	8015.06	8128.69
238	134.8646	457	6812.6	6880	6699.3	9192.61	8814.29	8163.274	10094.3	8447.51	6513.09
239	134.8646	398	4971.16	6577.4	4816.63	7635.7	7992.25	5991.093	7967.38	6504.38	4825.51
240	134.8646	287	7228.62	7428.9	8852.29	8538.98	9317.92	8390.668	13210.8	10786.4	6655.46
241	134.8646	150	9904.17	22613	9137.1	16429.7	9588.18	11043.32	18205.1	10379.3	8033.24
242	134.8646	126	7513.13	9621.1	6669.61	11652.2	11662.4	8395.518	19027.1	10925.7	6636.98
243	134.8646	53	8665.63	16874	13323.3	9820.69	22004.9	10327.99	40929.4	13487.9	6393.88
244	134.8647	1034	5735.33	8224.9	9272.62	9883.61	3630.23	12678.81	13849	21592.5	23156.4
245	134.8647	437	7850.63	8197.4	7242.53	9843.35	9992.15	8940.414	11679.4	9036.89	7241.9
246	134.8647	356	8367.99	8899.3	8581.17	11552.7	10537.3	9870.485	11137.6	10984.4	6657.14
247	134.8647	265	6807.46	8131.5	8318.32	7859.32	10185.1	9149.789	21158.5	9499.72	9070.77
248	134.8647	234	7818.46	7054	7775.3	8115.53	11361.4	8980.457	20178.9	11134.8	6570.7
249	134.8647	216	9069.11	8743.3	11337	11666.3	10195.3	9088.495	21517	12469.8	7469.08
250	134.8647	202	9348.4	7638.6	8968.64	9686.44	9577.09	11585.75	22690.1	12977.6	7398.74
251	134.8647	175	7760.69	6750.5	7349.36	12047.8	13431.2	11071.02	18057.2	10157.6	6736.78
252	134.8648	1178	7640.07	6772.7	6582.41	9100.84	17910	8096.127	10105.1	6815.37	6694.08
253	134.8648	1151	8830.72	803.99	5956.66	6976.61	8280.78	7407.924	8262.83	8577.35	6108.5
254	134.8648	480	6800.21	7709.9	6963.19	9924.54	10208	8782.418	11102.3	8887.69	7037.25
255	134.8649	1064	7959.42	5984.4	5909.47	11470.6	6214.1	6447.262	9370.69	7104.78	5600.84
256	134.8649	17	10358.4	10093	13556.9	11583.5	10041.8	11777.97	25975	10354.5	8794.37
257	134.8651	1109	6995.52	5271.5	6856.13	7759.13	7276.92	6289.984	7746.69	7022.56	6128.75
258	134.8939	70	9156.8	2047.6	10941.6	731.794	1757.85	8631.043	3787.77	2430.55	1271.32

259	135.03	65	4180.5	2367.9	5278.51	1852.07	2129.84	1915.237	5618.47	2634.71	1913.7
260	135.9703	1084	874.477	166.08	858.613	951.274	1484.8	1129.161	3005.14	1131.31	2545.24
261	135.9703	1041	4432.94	4562.6	2813.57	6387.29	3132.81	3669.86	4068.43	3460.55	2145.44
262	135.9703	129	2923.92	1184.1	2716.81	3771.97	3177.44	2509.918	14981.9	2910.05	2190.52
263	135.9703	110	1968.86	3804.3	2003.11	3705.81	7390.1	2349.99	4714.95	2920.16	3252.32
264	135.9704	218	2186.44	2286.5	1962.31	2549.83	2441.63	2193.353	3923.33	2421.17	1881.36
265	135.9704	41	1944.27	3032.3	1770.77	3105.79	3089.87	3338.424	6368.11	3107.31	4111.76
266	135.9705	1164	1076.31	1271.6	949.764	2597.87	2367.15	1205.652	2093.87	1295.07	582.75
267	135.9705	1141	1297.93	1200.3	1128.82	1451.84	1308.28	1217.522	1265.06	1200.94	1118.83
268	135.9706	1187	2776.05	2530.1	1619.24	2200.47	2644.43	3412.702	3655.62	2005.54	2676.63
269	135.9706	1062	2783.86	2346.1	2842.58	3254.04	2922.3	2652.962	2764.64	6475.88	1922.31
270	135.9706	150	2522.83	1834.3	2306.23	2399.63	2151.7	2319.212	4076.77	2332.61	1875.14
271	135.9706	6	1337.5	1360.2	1352.27	1522.7	1604.51	1675.39	1176.62	1775.24	1316.2
272	135.9707	1120	1549.51	1327.9	1430.74	1518.92	1637.42	1381.355	1516.46	1273.65	590.717
273	135.9709	1102	909.889	691.93	780.684	842.217	891.954	1130.026	1098.26	888.984	763.8
274	136.8619	611	1843.88	1775.9	1878.39	2151.97	2059.68	1764.739	2083.96	1708.54	1613.22
275	136.862	1194	2295.78	1140.9	3683.92	4490.14	0	1824.968	2766.65	1187.13	1120.66
276	136.862	124	2541.01	3042.8	2783.68	4405	4383.9	3197.58	7282.64	4054.13	2453.73
277	136.862	48	1152.74	1955.6	3175.47	4217.1	2677.99	3543.867	13797.6	8045.01	1926.73
278	136.8621	712	1570.77	1417.7	1668.57	1812.41	1817.68	1786.578	1847.71	1598.87	1476.35
279	136.8621	347	2974.99	5273.8	3340.57	4120.22	4473.43	3650.908	4398.06	4558.77	2422.06
280	136.8621	292	2177.75	2168.5	2516.75	2411.69	4345.47	228	4089.67	3902.82	3143.97
281	136.8621	264	2568.96	3254.8	3197.46	3902.97	3698.07	2934.72	5317.56	3079.65	2590.11
282	136.8621	248	1532.86	1999.4	3030.86	3016.29	4250.87	2680.435	6662.69	2956.35	2216.09
283	136.8621	205	3483.53	3488.4	3803.2	423.108	3513.49	3558.614	8366.56	4841.31	2687.75
284	136.8621	175	2288.23	2898.1	2805.41	4276.99	3315.34	4058.688	6627.61	3898.49	2505.22
285	136.8621	150	6780.1	2688.1	3331.11	10396.8	3232.38	3954.964	6259.26	3743.08	3117.21
286	136.8622	1180	2481.92	2463.2	2155.44	3143.81	2996.54	2902.062	3484.09	2306.89	2648.92
287	136.8622	1157	2495.03	2224.5	2301.21	1439.06	2376.92	3184.14	2712.7	3381.06	2418.12
288	136.8622	1116	2867.07	3982.1	4095.29	3790.65	4509.55	2869.368	3284.1	3840.23	2655.53
289	136.8622	456	2446.89	2201.8	2878.65	3192.17	3111.34	2044.738	3398	2806.6	2156.22
290	136.8622	229	2159.85	2458.3	2186.34	3096.79	3273.45	4307.568	7123.29	4703.78	2442.51
291	136.8622	21	3919.37	3128.2	4147.93	4129.02	3813.48	3797.845	9297.06	3480.96	2756.14
292	136.8623	1070	2806.69	2173.6	2892.04	4031.67	3611.21	3246.283	2861.53	3573.23	3457.42
293	136.8623	1034	2490.84	5894.7	4511.99	3669.27	5891.33	4681.423	4944.57	7922.39	7288.94
294	136.8623	309	3401.09	2748.1	2786.9	4910.79	3304.41	2420.272	4277.8	3139.42	3668.83
295	136.8623	69	4509.04	5349.1	4834.97	4930.75	3807.82	4023.716	8818.32	6379.42	4933.9
296	136.8624	526	1633.54	1941.7	1824.62	2190.56	2248.38	2203.252	2473.99	2137.3	1647.79
297	136.8624	474	2041.77	2525.9	2283.3	3228.31	3308.95	3009.927	3468.5	2761.94	2257.1
298	136.8624	2	1619.83	3286.1	1449.36	2397.22	3333.24	0	2590.06	2309.1	2624.04
299	136.8906	67	6333.04	5077.8	6645.27	6253.92	6526.03	7573.747	1231.08	7682.38	4928.07
300	136.9367	976	431.139	473.86	481.998	472.528	466.714	469.1192	417.468	463.354	528.184
301	137.0246	2	0	7581.1	0	0	10319.4	0	23772.7	0	6121.69
302	137.035	62	3673.53	3355.6	3186.18	5127.46	5332.83	4063.06	8001.25	4259.4	3031.96
303	138.019	66	2672.57	2497.7	3210	2902.64	4422.72	3044.506	5253.55	3099.75	2247.77
304	138.8595	248	498.218	504.28	590.549	615.969	598.941	547.6125	1060.26	604.827	494.306
305	138.8597	175	402.458	448.49	348.345	585.407	540.325	531.4014	885.967	611.46	394.096
306	138.8597	150	553.129	537.24	492.454	711.849	557.529	558.6602	901.815	576.284	464.515
307	138.86	201	574.852	512.22	593.081	746.592	633.017	685.645	1265.43	743.433	506.695
308	140.0687	84	265.844	234.48	1334.63	541.18	248.533	283.54	2537.89	1641.99	333.769
309	140.0716	2	34.3715	22.681	527.809	74.9051	61.0853	67.30812	1040.93	601.717	45.9188
310	140.9853	45	1125.9	1072.7	1102.98	1434.56	1402.89	1149.478	2642.1	1415.97	937.581
311	140.986	116	1343.39	1146.2	1262.37	2147.72	1799.3	1453.733	3317.5	1582.7	964.846
312	142.0115	101	1134.98	586.94	1289.62	803.528	788.285	683.0472	1616.5	817.74	509.89
313	143.0716	2	174.644	251.06	531.74	306.84	354.935	221.7096	2669.33	657.294	167.123
314	143.0723	139	790.759	640.52	1194.24	1115.35	841.954	757.8568	2763.7	1408.84	761.959
315	144.9813	990	1384.99	1933	1360.35	1461.78	1384.73	1444.556	1389.38	1477.71	1365.52
316	144.9814	964	649.433	678.85	1053.25	693.745	646.943	693.2258	690.343	807.049	659.155
317	144.9815	900	198.503	217.91	218.685	263.01	226.084	230.3379	259.658	247.145	224.31
318	144.9816	1004	685.731	613.66	645.977	115.107	699.615	768.2005	676.465	753.124	639.321
319	145.0497	63	2417.62	2124.1	2182.73	5194.63	3953.73	2752.668	4759	2681.56	3046.15
320	145.0615	119	2078.34	1470.8	1449.06	9157.42	9331.04	7759.509	40040.8	19201.6	15981.5
321	146.0453	69	4914.31	2756.3	6842.3	3378.47	3891.45	4249.08	9548.21	4711.21	2676.08
322	146.0454	300	9367.36	4830.8	6456.83	2201.12	4083.46	5802.022	11663.2	7275.68	5763.35
323	146.0645	115	497.955	332.44	354.8	1531.88	1438.4	1390.938	2166.94	1425.57	964.478

324	146.965	255	7380.63	6887.6	6376.43	7977.52	8337.24	8010.496	12910.2	8209.32	4657.42
325	146.9652	1131	6339.86	5623.9	4864.29	5448.95	5513.98	6384.086	6362.5	6468.62	7229.95
326	146.9652	321	9244.24	4203.3	3861.26	3156.79	4943.23	4838.586	5915.57	4704.75	5864.7
327	146.9653	600	4866.68	4960.8	4373.89	5819.36	5139.37	5065.211	5068.14	4559.62	4297.04
328	146.9653	457	5149.11	4528.7	3450.6	5285.94	4819.76	5510.46	4536.68	3621.86	3192.59
329	146.9653	183	5668.42	9353.5	5900.16	6711.5	7482.82	5724.267	16722.6	10126.3	6203.46
330	146.9654	1161	11983.9	10828	12533	12019.5	12094.1	10976.66	11531.2	10461.3	11374.1
331	146.9654	1112	3545.12	3841.3	6239.37	3353.64	4599.28	3321.949	3752.76	3594.64	4123.05
332	146.9654	1079	7793	10148	6976.64	10140	11478.2	7941.583	12607.7	9196.12	8924.64
333	146.9654	342	4221.76	5210.6	4415.03	3983.51	6180.46	5346.839	10866.4	4960.77	542.923
334	146.9654	157	7053.45	8552.6	5699.83	33675.5	9460.11	8922.945	15749	9068.59	16845
335	146.9655	230	8036.87	6141.1	8122.38	11213.6	7997.07	8934.701	18500.9	9858.73	3817.24
336	146.9655	214	10093.3	10243	9592.28	8352.18	10686.9	7342.091	20151	5177.34	6765.99
337	146.9655	48	5168.89	20058	13688.6	19247.3	24213.8	8675.964	13818.5	7232.03	19733.9
338	146.9656	1189	11246.7	8321.8	3882.44	11120.3	5850.87	9190.544	12853.8	9124.99	3540.72
339	146.9656	1035	4290.23	3537.1	4072.69	4470.26	4225.1	4051.764	3310.48	3013.55	3200.41
340	146.9656	278	6691.67	8370	7724.14	9926.48	9418.36	10227.45	11757	8936.09	7128.62
341	146.9656	127	6123.02	13334	6481.75	8921.29	9143.61	8322.751	11965.3	7450.06	6177.82
342	146.9656	5	2218.02	8677.3	8622.77	10619.4	11303.9	8212.78	16764.3	8193.28	8020.53
343	146.9658	731	7348.38	6858.2	7081.97	8818.25	8185.9	8751.62	8192.86	6514.76	6227.72
344	146.9825	64	5590.82	2106.6	3765.84	3057.16	3476.59	2625.333	8991.29	4852.44	3456.16
345	147.0293	71	2959.78	3279.6	3183.62	2759.42	3971.29	2138.063	9283.32	3081.53	3072.76
346	147.0489	243	2128.7	8028.8	7532.81	8249.43	8923.79	8637.709	2087.5	811.322	6971.85
347	148.9798	63	1653.39	810.07	1585.59	776.345	1003.57	745.0104	2840.68	1557.63	823.46
348	149.9936	1	965.712	804.75	1008.64	854.572	2103.56	1610.002	270.86	1667.66	1564.49
349	149.9937	1139	957.776	1010.2	915.744	1061.24	1193.79	907.3325	1017.12	935.912	1023.93
350	149.9939	1163	1181.04	1326.7	1995.4	1251.21	2150.66	2369.365	1753.38	2210.34	1426.51
351	149.9939	1061	1080.93	1486.9	1160.37	1799.61	1917.47	1321.791	1753.77	1606	1534.49
352	149.9939	190	955.537	1153.1	872.263	1075	563.144	1045.319	8125.26	1354.99	1141.65
353	149.9939	47	1805.4	2222	2169.39	2530.23	3863.7	3203.658	3747.06	1692.33	1776.74
354	149.994	1084	1981.65	2288	2360.68	2686.05	2401.33	2514.967	2454.35	1938.13	1895.87
355	149.994	220	1733.97	1847.5	1563.21	2235.91	2286.26	1768.81	3770.34	2052.88	1575.35
356	149.994	132	2671.93	1960	2426.85	2803.73	2154.84	2659.871	5290.68	2634.88	1862.41
357	149.994	108	2215.46	2250.3	2332.76	2284.88	2842.91	2448.286	4989.9	2433.73	1893.63
358	149.994	24	2978.27	1321.8	1001.25	2391.28	1519.84	1881.68	3364.16	1718.04	1197.26
359	149.9941	1186	1876.74	1955.5	1504.9	2961.47	2413.68	1739.294	3067.16	1361.83	847.933
360	149.9941	1109	1435.52	1294.9	1330.89	1540.79	1728.82	1697.957	1743.82	1543.7	1424.85
361	149.9941	287	1234.17	1381.7	1448.34	1506.58	1611.45	1611.5	2154.01	1453.04	1185.53
362	149.9941	157	1801.5	1492.3	1809.82	2257.1	2460.7	2190.72	4274.57	2412.53	1439.86
363	149.9942	1040	2208.95	1898.4	2059.84	2611.43	2377.25	1556.554	4242.72	1901.38	1930.5
364	149.9942	260	1489.09	1405.8	1588.77	1676.71	1931.84	1843.547	2840.66	1744.9	1290.15
365	149.9946	307	1385.67	1365.7	1327.8	1727.52	1632.97	1642.854	1998.53	1456.47	1279.61
366	150.8862	71	10871.1	9646.6	10117.6	8134.72	10025.6	7949.839	26423	11107	9164.9
367	151.0256	64	3856.97	1273.2	5546.59	1153.13	1415.9	1649.819	2747.77	1466.1	1082.31
368	152.0349	66	1775.05	1449.8	1636.06	1900.92	1816.31	1706.143	2950.83	1735.38	1451.02
369	152.8832	70	11454	10162	10902.5	8168.76	9589.61	8013.455	26801.6	11806.9	9653.44
370	154.8802	71	3864.31	3364.5	3537.63	2730.84	3375.28	2722.073	9586.47	3715.52	3468.69
371	154.9463	73	4074.67	913.78	3657.14	2825.1	698.832	2648.572	9687.78	3721.75	3311.31
372	154.9468	999	1955.13	1411.3	2352.73	1780.95	2344.05	1936.362	1753.53	1858.09	1792.88
373	154.9469	964	907.753	1987.9	1836.98	1874.44	1608.45	1987.966	1198.87	2034.88	1845.15
374	154.9469	924	2293.6	5007.5	5331.51	3863.05	7591.05	6510.9	4350.15	4763.71	2674.95
375	154.947	1018	2756.45	152.75	1706.3	10030.8	4911.42	2750.004	4143.94	9872.77	7663.66
376	155.0094	73	4237.24	4823.6	4092.26	1041.92	1160.87	1109.215	3983.15	2258.36	2399.36
377	155.8735	55	629.454	1411.3	878.806	2148.33	1523.45	2102.207	1772.61	1079.61	2552.68
378	155.8739	30	455.592	346.34	431.664	447.545	351.2	389.5794	695.703	459.734	300.213
379	155.874	3	280.694	390.41	312.018	322.709	438.975	318.0392	596.716	422.379	300.893
380	156.0282	95	3817.56	1911.6	3602.71	3260.52	2981.59	2408.703	4717.05	1818.75	2132.82
381	156.0426	69	1665.42	1067	1771.33	1204.65	1124.91	1572.706	3056.32	1351.83	1314.65
382	156.8781	74	422.063	373.45	363.343	276.493	393.68	297.5139	870.638	461.766	261.982
383	157.0112	63	6661.68	2552.9	4840.91	4287.76	4211.85	3215.787	12999.2	5175.47	3150.14
384	157.0249	73	11685	5297.3	4884.05	4958.38	5475.97	4041.926	17405.8	8401.73	2629.68
385	157.0605	103	2150.96	1277.6	1629.21	772.255	494.547	572.2727	1481.38	1038.37	770.607
386	157.0616	70	2676.32	1475.1	1869.2	914.589	750.272	703.3111	1587.66	1134.6	836.37
387	157.8713	55	1349.99	930.31	1251.36	1163	2597.91	1656.187	1566.88	1937.29	2344.71
388	158.8461	59	1631.19	1571.6	1943.77	1739.89	1011.57	1513.167	2638.5	1455.61	1916.42

389	158.9749	1	0	0	0	0	0	0	0	0	92.8
390	158.9778	730	1900.31	1680.8	3336.21	1828.33	1666.28	1894.728	2861.63	2139.88	1682.64
391	158.9779	1100	1399	960.31	781.11	1029.84	1831.29	1274.55	1168.68	1103.51	1553.68
392	158.9779	1078	693.279	1213.2	681.211	1016.72	1387.69	1428.177	1621.22	1024.88	1069.85
393	158.978	1119	1582.39	1137.8	1163.93	1334.14	2281.33	1740.784	2058.99	1459.05	1673.72
394	158.9781	959	2246.8	1957.6	2006.61	2520.23	2459.21	2287.069	2566.93	2263.63	1961.63
395	158.9782	821	3548.94	2123.6	3628.58	4369.6	4019.78	4549.354	4078.98	3816.97	2919.52
396	158.9782	795	2953.03	4540.3	3471.7	6429.15	7454.4	3955.665	7545.63	13024.8	2829.44
397	158.9783	1178	772.491	667.36	556.026	900.623	793.682	929.5449	1160.11	831.897	666.204
398	158.9783	887	7880.52	6452.5	35225.2	7134.33	15596	6016.185	47061.2	36508.9	5908.5
399	158.9783	842	3373.49	2593.4	4305.89	4414.49	3841.14	3829.936	4351.49	3937.59	3538.38
400	158.9783	772	2113.85	1486.4	3216.02	2360.18	2623.2	2710.791	3241.39	4731.48	1691.5
401	158.9783	751	3656.2	3516.3	5633.1	3828.44	3974.07	4310.339	7620.93	4423.52	3302.01
402	158.9784	122	1855.98	1622.7	1587.51	2448.45	2510.17	2188.135	4518.42	2138.84	1441.7
403	159.9793	821	413.226	344.79	345.245	397.082	490.862	484.8664	540.701	480.194	350.684
404	159.9796	886	1757.66	4614.3	4516.65	3966.27	5595.11	5150.454	6294.94	5181.94	5226.28
405	159.9796	808	523.594	375.74	507.156	641.82	659.977	590.8346	745.669	669.317	507.515
406	160.0609	68	3397.64	1567.4	4059.13	2098.6	1585.33	2255.202	4522.5	2786.24	2024.7
407	160.8409	1044	619.144	652.73	725.995	544.907	554.893	717.4023	1008.57	1042.04	805.776
408	160.8413	59	24562.4	23813	30839.3	21554.6	29622.3	19394.33	42363.6	23582.5	24467
409	160.9152	67	2029.23	1224.9	1651.08	1624.97	1301.95	926.0226	1818.96	1492.35	1125.74
410	160.9756	811	424.812	308.44	476.899	825.802	539.343	525.6277	571.082	487.954	376.147
411	160.9756	795	674.007	447.12	539.47	499.149	667.887	637.5953	848.414	348.232	524.628
412	160.9757	906	603.375	754.65	915.568	1172.77	928.793	940.0671	1077.98	793.012	738.914
413	160.9757	879	1100.44	837.67	966.646	781.986	2728.66	5900.721	1526.76	730.389	963.415
414	160.9757	850	612.523	498.7	472.408	652.743	709.758	637.0728	710.357	878.193	445.056
415	160.9757	832	463.331	327.68	367.006	631.539	523.596	503.0569	613.912	826.943	360.87
416	160.9758	922	808.387	606.42	543.945	730.76	794.712	82.34667	664.321	571.265	504.205
417	160.9759	944	644.133	515.35	441.961	468.481	888.639	530.4176	668.967	638.381	522.427
418	160.9763	765	410.888	373.73	533.766	542.572	500.999	490.3697	545.994	458.573	324.222
419	160.9764	978	638.423	634.65	644.604	785.953	750.899	694.1952	825.525	713.078	629.411
420	160.9766	744	275.88	282.46	396.789	343.894	330.057	347.2328	412.82	390.89	283.297
421	161.0454	65	1390.74	1354	1473.48	1799.62	1910.44	1488.367	4337.95	1619.32	1090.21
422	161.948	962	483.266	507.01	571.479	609.602	537.651	574.2109	730.795	537.278	480.239
423	162.8383	59	33328.3	25793	33820	28706.1	25689.1	23981.05	39210.5	25728.2	27395.6
424	162.9122	67	1551	753.24	1033.54	1036.41	976.563	600.9262	1949.55	1185.82	827.029
425	163.0608	63	702.322	325.62	534.81	738.167	662.474	540.522	1084.2	763.023	647.401
426	163.8383	59	644.124	808.27	876.499	1085.49	564.008	685.1905	1199.19	563.598	745.243
427	164.0717	62	1781.33	976.31	1793.03	1265.44	1415.47	1119.968	1193.94	1540.39	1197.38
428	164.9273	57	745.661	877.45	589.147	1005.95	1547.65	984.7714	2032.67	837.266	834.307
429	165.0186	61	1170.1	2217	2089.76	2443.26	2526.41	1732.26	4485.64	2035.86	1737.57
430	165.0189	1051	2156.61	1839.3	2156.51	2201.11	2854.51	1364.949	3412.2	1871.87	1823.69
431	165.0193	142	1906.27	1452.8	1736.81	2061.62	1805.08	1603.174	3674.77	1970.29	1324.67
432	166.017	65	2279.87	1518.5	2091.6	1582.22	1601.38	1431.865	2151.99	1258.43	1401.45
433	166.0503	64	1443.72	1948.7	3376.49	2410.88	2196.65	2222.497	2629.41	2404.1	1032.99
434	166.8322	58	1262.72	987.28	1277.91	1149.53	658.964	901.3781	1539.01	892.738	939.675
435	166.8602	70	879.252	607.17	906.168	878.777	676.026	596.9569	1053.85	611.425	773.06
436	167.0205	71	2958.05	1606	4085.47	1016.27	3017.86	1429.167	2706.95	1966.06	1763.23
437	167.8361	1184	745.676	39.389	579.588	753.664	594.585	664.4929	659	576.333	628.583
438	167.8362	1049	2717.5	2297	2716.46	2542.51	2381.7	2238.465	2941.64	3074.99	2411.18
439	167.8362	68	1683.97	4290.5	2386.81	2229.9	3709.9	1689.748	4194.11	2365.42	2154.4
440	167.8363	46	242.097	1078.2	554.025	112.253	2015.41	430.3267	1657.39	682.042	1597.44
441	168.0276	266	1094.83	473.64	1089.43	755.285	632.854	864.5361	1337.96	942.144	557.366
442	168.0279	242	1598.22	640.78	899.778	836.854	850.456	861.6569	1957	834.995	700.524
443	168.8354	56	2399.63	2278.5	1486.66	1117.43	1547.15	928.5558	3284.88	705.281	2190.57
444	168.8569	71	664.8	511.42	769.621	528.655	574.278	545.1803	1414.04	604.169	563.4
445	168.9891	59	721.327	418.35	941.585	355.261	483.322	373.1829	939.339	506.154	380.525
446	169.8332	42	458.805	180.9	720.323	146.68	186.761	592.1301	2247.18	853.326	184.101
447	169.8334	1049	2992.61	3545.4	2183.91	3716.45	3275.24	3193.846	4562.82	3426.77	2872.28
448	169.8334	68	1093.71	6240	3428.88	2875.78	4592.91	3208.884	6243.53	2783.12	2912.52
449	169.8336	1191	1023.16	137.17	763.397	999.003	445.251	905.5063	959.21	822.375	336.47
450	169.8336	1173	22.8185	628.94	32.5951	101.203	783.363	32.55429	333.487	230.155	837.214
451	169.8339	144	191.797	46.837	241.78	217.28	65.0284	413.4395	780.673	467.93	19.3132
452	169.9817	35	1893.8	1664.1	1552.63	1991.32	1880.22	1836.045	2777.26	1720.6	1469.4
453	169.9817	3	713.246	949.33	716.137	1003.01	1108.19	734.9239	1607.81	638.713	775.859

454	170.8326	1055	1041.67	1240.1	857.192	1422.64	929.832	1135.705	2164.42	1060.43	1207.07
455	170.8327	57	3300.03	3488.5	3487.91	2224.63	2349.75	2087.053	4946.22	1342.92	3175.76
456	170.9435	76	5261.17	3851.1	4510.12	7028.74	4217.84	3960.505	10539	3424.93	3994.89
457	170.9442	62	2987.1	2034.2	2161.78	3060.14	2618.3	1337.198	4707.82	1997.94	2030.66
458	171.0267	63	1045.3	1634.1	1889.35	1961.47	1984.32	1097.67	835.255	1068.08	686.426
459	171.0421	62	1113.77	1115.9	1659.14	1621.05	1563.93	758.7268	1278.71	909.241	501.362
460	171.0654	3	138.331	108.17	205.6	237.582	129.929	173.6415	359.117	181.558	104.891
461	171.0655	95	754.728	709.96	797.258	961.39	991.591	758.1199	1906.62	739.428	648.737
462	171.066	67	1553.3	1510.6	1676.67	1639.66	1798.87	1340.008	2059.33	1708.61	1125.54
463	171.8308	1050	1729.54	1716	2044.6	2273.34	841.891	1710.748	2376.37	2038.22	1637.69
464	171.8309	1184	513.329	27.1	442.887	593.809	19.1862	226.5039	60.345	92.8963	19.3154
465	171.8311	66	1268.68	3636.2	1914.14	1640.42	2837.68	1915.381	2841.94	1940.32	2805.68
466	172.0221	98	1764.2	813.02	916.691	1194.79	1038.81	657.9258	1889.76	816.19	848.061
467	172.0229	156	799.322	309.78	621.303	397.799	323.758	350.3415	1212.07	661.012	380.206
468	172.023	127	962.936	417.17	949.885	520.953	503.854	461.5023	1647	751.566	496.821
469	172.0982	2	489.703	435.02	1202.43	680.397	807.209	604.4255	3197.4	2162.43	521.644
470	172.8301	1057	701.528	276.06	588.681	658.006	290.758	726.5573	580.389	434.046	282.791
471	172.8303	56	2576.3	2539	2709.19	1318.34	1778.97	1378.264	3910.57	837.65	2574.01
472	172.9409	75	2696.4	1304.4	1904.17	2412.94	2017.26	1508.366	2479.08	1602.57	1314.2
473	172.9573	51	1490.89	1000.6	1418.79	1674.14	1302.83	1265.463	2137.73	1327.02	810.148
474	172.9574	211	1139.67	1204.9	1250.53	1418.32	1321.06	1227.715	3982.95	1657.2	992.103
475	172.9574	73	1845.48	2344.2	1883.14	3150.92	2190.02	3105.195	6106.33	2607.49	1500.19
476	172.9574	2	0	894.82	0	0	1021.17	0	1839.14	0	539.693
477	172.9575	1168	148.833	42.78	78.12	94.72	62.075	80.275	57.75	56.73	71.36
478	172.9575	1016	14139.6	3676.9	4571.72	19483	21124.5	20157.27	12680.6	38431.4	32169.1
479	172.9575	896	2028.56	1974.9	1855.92	2519.03	2405.4	2093.405	2508.46	2009.15	1946.58
480	172.9576	993	4105.81	4033.7	3797.86	4528.1	4630.78	4277.739	5711.47	4402.82	4130.79
481	172.9576	956	3633.14	3331.1	3166.21	3851.19	3619.25	3885.668	3916.61	3606.11	3370.61
482	172.9577	946	2450.85	2265.1	2316.05	2650.15	2673.73	2448.15	2578.36	2493.92	2222.44
483	172.9577	924	15948.7	14426	6387.71	9868.45	20550.9	16129.03	2971.47	15804	6150.03
484	172.9577	119	1699.24	1482.4	1770.9	1928.89	2194.35	1821.147	3555.21	2223.92	1269.81
485	172.9578	29	1463.43	1244.6	1345.63	1541.86	1451.96	1463.63	3297.71	1422.99	1042.52
486	172.9934	875	858.495	828.9	721.272	928.154	1069.39	925.6475	923.871	778.141	670.418
487	172.9935	963	5051.95	4835	4462.65	5491.09	5404.98	5283.112	1340.67	5064.18	4788.45
488	172.9936	929	4183.65	3644.8	3700.56	4086.28	4092	4242.942	3695.21	3695.32	708.633
489	172.9936	856	468.837	402.92	374.163	392.785	365.144	449.563	460.443	381.665	366.057
490	172.9937	998	3795.49	3559.3	514.811	4020.44	3533.41	3973.255	3702.23	3734.47	3348.99
491	172.9938	984	5804.79	5703.6	5292.85	6572.09	6560.16	6286.75	6337.51	6362.34	5707.58
492	172.9938	903	1517.79	2528.1	1195.65	3482.29	5045.32	2706.535	3123.13	2715.66	1376.98
493	172.9944	942	3834.88	3577.8	3603.35	3985.58	963.63	3714.989	3698.6	3939.66	3380.87
494	173.0089	70	1452.35	2872.4	1467.88	2303.52	3218.44	1683.974	4611.41	1336.36	2188.43
495	173.8287	54	256.152	556.72	296.631	328.351	818.588	323.314	579.917	243.095	608.044
496	174.0017	62	2020.48	962.71	2774.47	1811.89	2160.61	1681.009	4810	1812.12	1223.35
497	174.8279	55	598.627	956.42	728.68	467.026	939.971	470.9239	1582.54	473.512	1229.29
498	174.9549	1199	5506.58	1668.8	3838.14	6795.25	2059.06	2860.699	2330.07	1493.85	1859.26
499	174.9555	1178	11263.5	10091	8216.74	13573.4	12912.5	13728.77	19286	13936.7	10277.3
500	174.9555	944	19490.6	17918	22270.4	22903.9	20941.1	19964.87	27956.7	23573	19271.9
501	174.9555	228	5996.77	5749.7	5034.5	9577.87	8248.12	8437.756	18141.3	7758.9	5082.09
502	174.9555	126	9599.68	9192	7500.84	14840.7	12792.9	10308.5	21939.7	10194.5	7675.26
503	174.9556	1003	54419.4	51884	52431.8	67320.8	62819.7	59836.79	72037.9	61707.1	50907.3
504	174.9556	955	111167	103543	106605	128960	122537	115925.4	141965	119088	102308
505	174.9556	899	10482.7	9872.2	9718.61	13377.7	13026.6	11720.86	15033.7	11700.7	9462
506	174.9556	203	8658.11	6005.8	6538.14	11277	8563.54	10329.29	21577.4	9728.69	5205.08
507	174.9557	1123	22138.8	27829	23530.8	27459.2	32863	24417.91	39684.1	26252.9	51527.8
508	174.9557	176	4232.63	1853.4	3106.44	5563.5	3076.07	6789.3	10134.1	5685.46	1603.02
509	174.9557	154	8568.75	7436.1	5842.17	13060.3	8564.45	8920.39	17582.6	8393.91	5756.48
510	174.9558	1083	17971.6	17325	12232.6	25119.4	22513	22569.19	25616.3	17985	14003
511	174.9558	1024	28532.9	27619	25929.4	36520.7	35767.6	31431.47	41917.2	34798	29445.1
512	174.9558	337	5189.69	5504.4	5260.98	8764.31	8235.87	7946.714	8240.55	5622.11	4884.93
513	174.9558	53	10494.7	7892.2	7654.07	10887.2	11409.5	16849.64	30755.7	12816	5096.29
514	174.9559	354	4528.75	4673.8	4157.88	6990.31	7214.11	7095.692	7276.35	5177.23	3737.18
515	174.9559	272	5576.83	5319.7	5609.62	9069.83	7766.77	9151.272	11778.8	6927.72	4799.27
516	174.9559	30	10374.6	14738	8089.99	14229.3	17062.7	13361.83	22871.6	7503.52	14440
517	174.9559	5	18284.3	13016	11936.3	22479.5	17535.2	13357.41	18522	8332.64	10962
518	175.0357	111	7559.63	3322.9	8860.16	18282.1	18204.8	17212.04	6940.64	3374.07	9754.86

519	175.0612	63	1107.34	908.58	1403	748.806	482.955	507.7914	1062.31	698.412	551.783
520	175.9586	2	232.643	540.52	148.32	457.621	580.026	203.3141	741.645	363.754	446.667
521	175.9589	980	1505.1	1189.5	1268.44	1383.69	1340.98	1997.791	1544.55	1251.32	1146.26
522	175.959	1081	787.383	733.38	814.412	1104.37	834.667	877.8946	1080.91	756.787	776.16
523	175.959	959	3513.32	814.24	1245.08	984.333	924.013	294.4548	2653.3	1882.95	1395.23
524	175.9591	1158	179.934	438.66	171.057	242.826	519.358	229.7439	354.027	300.808	439.981
525	175.9591	1102	559.924	658.98	476.622	675.255	939.679	705.448	812.631	858.522	790.518
526	175.9591	999	163.499	144	204.788	168.84	164.628	171.2	172.839	160.68	151.204
527	175.9593	1147	395.508	393.3	370.326	399.277	517.182	630.1746	719.037	645.272	426.492
528	175.9593	1121	1042.72	1044.8	1000.56	1238.73	2588.08	1153.052	1511.31	922.029	1139.53
529	175.9595	1063	708.266	481.45	608.654	916.933	604.756	551.0976	860.391	653.859	436.849
530	175.9997	98	2382.41	1040.6	2352.71	1539.88	1417.3	1273.018	2631.61	1532.75	1038.5
531	176.0086	71	1087.63	600.84	1351.85	1103.43	660.926	806.0089	2071.91	678.965	652.019
532	176.0566	70	652.68	522.03	723.486	373.913	379.635	408.2119	633.181	524.161	460.222
533	178.9486	998	886.105	899.59	916.675	238.809	1020.84	1025.006	1078.21	1452.67	863.575
534	178.9486	962	932.103	718.76	676.362	1128.81	825.568	932.8768	813.789	840.718	646.161
535	179.0554	63	2653	1616.4	4216.45	2490.91	2381.86	1356.482	7120.59	2906.43	1622.09
536	180.0657	95	1084.72	863.68	2989.95	892.1	951.333	869.5211	2578.22	1034.54	830.516
537	180.9723	175	177.181	179.34	203.966	219.311	232.375	250.2844	620.139	320.652	142.337
538	180.9726	84	58101.9	28622	49424.4	52533.4	41976.6	45907.48	172382	61539.2	27940.4
539	180.9728	34	310.186	326.41	326.655	393.871	320.652	316.9565	811.27	388.215	263.395
540	180.9729	58	2446.96	3179.2	1507.06	2960.21	5611.72	1588.546	10729.2	4123.22	2774.9
541	180.9735	19	448.668	398.92	461.845	503.017	476.509	419.997	1154.22	444.197	340.969
542	181.0716	62	15197.7	4280.2	4528.69	9831.57	16146.2	3157.853	52368.9	30623	27536.9
543	181.9655	69	8573.04	4666.2	8519.05	5360.9	5519.83	5724.31	13124.6	6764.71	5159.38
544	181.9765	87	1954.76	1075	1442.64	1841.34	1964.08	1328.841	3337.3	2070.76	1261.4
545	182.0747	62	1425.08	492	437.397	799.534	1406.65	403.5705	3595.97	2685.06	2088.21
546	182.9775	84	1213.85	688.52	1017.82	1140.15	1235.72	952.654	4460.98	1421.11	756.771
547	183.0046	62	643.475	511.59	671.259	647.045	1053.42	676.0658	1518.62	794.791	545.868
548	183.0755	63	1205.19	1092.1	982.717	1384.64	1443.53	1159.113	2811.77	1477.7	1116.83
549	183.9624	70	3262.79	1937.5	3830.42	2086.98	2375.83	2029.561	4732.69	2844	2171.78
550	185.9923	66	3351.53	1247.5	4711.05	1622.38	1484.58	1906.696	1594.65	1961.7	1589.72
551	186.0372	105	7854.01	2668.4	8864	2436.05	3014.04	3708.136	14035.8	6791.77	3049.63
552	186.0372	92	15210.4	4280.3	15629.2	6066.7	4980.04	6666.699	18063.4	8495.61	4827.31
553	186.0382	96	13369.4	2622	16106	6682.34	2455.09	5263.874	22500.1	9225.99	5519.23
554	186.0386	146	480.881	279.7	457.328	254.662	259.902	248.6003	754.835	412.619	275.093
555	186.0455	67	1852.47	1189.4	2916.44	1014.74	1342.57	736.8367	1821.23	1269.11	1477.59
556	186.113	58	690.649	844.18	791.301	1048.34	1137.28	866.712	1474.13	1073.08	748.101
557	187.0415	66	6736.98	1916.5	7835.83	2437.5	2981.38	3397.253	8409.71	5443.63	4122.17
558	187.0719	71	1174.36	1203.8	1428.68	1095.69	1177.01	1601.713	3156.48	1712.91	1408.03
559	187.0976	65	2003.25	1707.4	2190.27	1916.12	3025.7	1515.131	4415.25	2475.93	1858.05
560	188.0561	71	1965.24	2829.4	2626.93	1314.94	2355.39	1451.151	4726.47	2293.09	2190.77
561	188.9706	1182	375.547	301.34	251.079	411.05	395.605	463.6084	729.564	525.835	308.66
562	188.9714	1121	800.69	657.13	688.673	773.616	951.555	947.478	1116.08	915.505	779.486
563	188.9715	954	9542.86	6346.3	29233.7	29442.4	8138.24	18724.52	48117.5	25571.8	9636.01
564	188.9716	1005	24900.1	12492	6537.52	5970.84	8186.38	34579.02	841.28	6557.87	10127.6
565	188.9716	980	7775.27	6884	6964.7	8313.06	8026.54	2151.83	20929.4	9332.29	5593.64
566	188.9716	900	2382.49	2122.3	2111.6	3135.55	2585.75	2580.669	3212.14	1989.74	2165.81
567	188.9717	916	4173.92	3986	4014.22	556.578	5879.16	2823.757	5661.49	4381.28	4091.17
568	189.0124	151	1628.68	689.63	1240.6	646.976	680.361	682.7741	1706.31	3191.14	816.294
569	189.0125	120	2283.69	835.88	1889.51	1109.11	1233.29	1102.402	2225.36	1572.18	939.695
570	189.0127	170	685.558	321.97	819.959	459.036	279.024	391.7595	648.201	610.549	300.108
571	189.0347	62	1459.81	1329.7	1082.3	1413.14	1132.13	1376.157	2386.97	1122.57	751.746
572	189.975	952	980.501	908.16	1016.86	1135.76	1016.69	1008.677	1226.27	1024.42	903.921
573	190.072	69	1112.18	757.97	1727.22	757.752	888.934	809.6634	2180.52	1073.73	851.816
574	191.0192	1048	1144.94	3497.8	1110	3885.85	7461.69	1467.511	6999.18	2316.66	3185.24
575	191.0531	71	795.264	675.9	764.099	1386.23	1081.95	849.4382	1077.13	895.841	529.198
576	191.056	990	596.584	528.18	524.621	1248.05	937.874	818.6634	1456.24	663.175	510.405
577	191.9943	82	5419.79	1736.8	5804.04	4298.26	2446.85	4080.4	13898.4	5746.31	1812.33
578	191.9947	60	1689.9	1044	1574.12	2930.99	3050.13	2056.582	7546.3	1906.05	1218.03
579	192.0229	70	3587.07	2014.7	3824.36	2139.22	3634.86	1186.412	8469.95	1224.78	2066.9
580	193.0242	70	482.776	420.55	584.152	743.58	910.445	643.0794	2093.14	665.22	410.276
581	193.0863	75	966.559	547.68	854.653	958.396	887.416	761.7088	944.072	650.68	619.043
582	193.8151	59	960.621	1335.5	1476.54	1957.53	1184.77	1124.064	2078.84	1538.08	1291.88
583	194.0819	1044	1565.58	1532.7	1419.3	1946.32	1992.55	1660.147	2307.03	1743.63	1764.35

584	194.9054	951	197.269	199.42	178.029	348.344	283.528	249.9033	382.766	216.759	180.785
585	194.9056	975	202.962	165.18	147.314	329.254	295.278	247.1833	361.39	225.576	158.838
586	194.9057	1013	1064.47	916.72	874.191	1533.97	1424.79	1296.729	1687.24	1082.78	840.842
587	194.9058	930	275.765	265.57	262.567	424.654	400.707	370.8221	468.947	303.549	247.328
588	194.9882	84	1428.6	758.93	1310.64	1471.57	1449.01	1200.431	4052.25	1730.25	821.167
589	195.0504	246	1052.61	352.74	1218.99	423.234	437.56	532.1633	1481.56	678.267	504.41
590	195.0659	66	343.029	197.73	351.058	361.812	1119.3	301.7617	764.969	486.802	305.768
591	195.8104	59	14579.3	27645	20630.7	41465.1	35351.2	20421.8	36377.8	27809.7	25260.8
592	196.024	63	913.978	557.02	777.095	876.041	820.022	617.9665	1852.31	866.276	484.28
593	196.9464	80	990.437	627.47	1268.11	1023.63	553.461	997.9856	3964.46	901.843	453.658
594	197.8075	63	7219.09	593.02	26770.2	1460.94	539.455	5550.193	933.733	471.846	381.451
595	197.9629	35	141.939	373.03	209.99	212.17	426.785	236.2093	714.539	396.178	354.097
596	197.9633	3	516.603	625.02	604.754	588.762	643.933	478.0305	1152.27	728.942	435.859
597	198.0492	96	6412.32	1292.1	9371.49	2493.44	1641.82	3795.996	10574.1	4078.78	2722.62
598	198.0746	76	1854.58	881.62	2491	986.961	1323.38	902.0808	785.829	1144.72	744.192
599	199.0376	99	20553.6	591.66	674.761	8414.18	16979.3	634.9128	10389.7	5009.44	4525
600	199.8051	58	8505.59	13866	11691.7	22675.1	14438.8	10951.28	21360.9	14168.6	13777.1
601	200.0563	68	1224.09	3184.9	1068.81	452.393	1275.23	348.0994	1335.04	749.239	555.214
602	201.0378	63	3294.4	1310.7	2707.32	1229.37	1113.87	1041.595	4064.58	1837.61	1235.56
603	201.8014	58	1584.96	2966	2498.07	4193.29	2322.98	2557.147	4382.12	3125.46	3088.96
604	202.9872	962	832.858	773.61	806.173	1275.44	683.499	1107.082	689.765	886.422	751.836
605	202.9873	1002	476.419	454.91	450.509	502.4	795.494	442.5867	553.029	475.067	411.378
606	202.9874	925	308.656	264.44	265.925	347.739	334.455	329.9833	394.157	323.739	255.36
607	203.0279	111	1940.54	2122.1	5323.55	2112.55	2002.35	1519.471	4276.48	2261.89	2176.01
608	205.0117	99	758.324	752.18	838.981	1297.88	1086.55	996.0195	1846.12	930.113	698.542
609	207.0506	96	478.461	465.1	494.174	932.463	748.936	709.1866	2149.52	704.311	447.117
610	208.0718	124	1023.13	778.62	896.493	853.563	956.736	945.6887	2644.2	1404.93	725.727
611	208.0725	102	1883.88	2128.3	3961.72	3346.58	2013.43	2176.705	4046.7	1688.21	1721.48
612	208.8451	70	15386.3	11700	13106.5	10013.4	11630.9	9237.851	34269.2	14421.2	10973.8
613	208.9338	673	1812.1	1838.9	1901.54	2219.48	2099.33	2154.735	2669.63	1885.46	1633.38
614	208.9338	2	0	233.63	0	0	273.24	0	1213.79	0	133.059
615	208.9339	847	1090.53	766.31	802.625	1205.94	1249.41	1005.78	1462.13	919.578	697.838
616	208.9339	750	1036.33	993.37	1179.48	1675.83	1357.06	1459.395	2091.02	1301.73	799.508
617	208.934	390	938.603	838.29	752.765	1139.17	904.899	1035.975	1517.76	1232.79	686.356
618	208.934	144	1937.06	2258.6	3916.5	3960.43	2258.85	1717.748	8232.91	4416.35	2139.72
619	208.9341	1061	2056.47	3320.2	2706.83	3335.92	3617.65	2316.556	3800.02	3021.2	2856.97
620	208.9341	415	841.128	674.14	692.686	991.44	894.354	910.2	1238.74	959.506	539.094
621	208.9341	172	984.617	578.5	575.346	610.628	1137.46	872.9348	2312.28	939.744	540.36
622	208.9342	1172	1049.01	2296.1	1407.94	2362.68	2979.67	2770.009	2436.25	1479.72	1552.3
623	208.9342	1139	819.178	1086.2	1421.54	1214.29	1277.02	1425.65	1833.22	2408.81	1046.78
624	208.9342	967	1788.19	1734.6	1620.39	2251.12	2843.41	1990.731	1878.66	1897.02	1569.45
625	208.9342	795	2853.22	1654.2	1979.32	2885.41	3271.58	2832.714	1223.93	815.735	1902.01
626	208.9342	712	436.043	898.02	1274.74	1599.74	1749.84	1393.94	1713.47	1674.48	1295.39
627	208.9342	658	1075.89	895.6	1072.49	1205.86	1134.12	1152.027	1600.58	908.336	797.16
628	208.9342	563	1124.17	900.14	1056.06	1719.44	1346.41	1377.471	1854.95	1132.3	784.115
629	208.9342	523	2396.47	1563.8	1074.12	2091.91	2196.04	1917.608	2622.08	829.983	1118.43
630	208.9342	191	1282.23	1021	1487.03	1367	1165.07	1301.173	2861.08	2052.62	818.602
631	208.9343	1188	1771.6	1155.8	865.53	2164.98	1429.6	2083.22	1868.31	748.372	1314.88
632	208.9343	607	1165.78	1028.1	1399.16	1639.35	1178.52	1220.884	1846.65	1068.05	952.772
633	208.9343	287	953.027	601.33	1037.69	897.427	819.657	994.0084	1613.1	1135.47	621.764
634	208.9344	1089	740.448	605.99	346.794	650.563	1661.63	820.9067	908.833	678.324	634.795
635	208.9344	1031	3366.81	5602.3	3080.16	3719.9	1635.51	6406.444	15554.7	10466.7	6728.61
636	208.9344	917	7189.62	10831	11560.6	22564.5	5846.22	12607.03	3514.5	7767.9	11798.4
637	208.9344	894	5192.15	1905.3	2942.05	3757.21	4722.84	2948.486	1459.12	566.685	3455.24
638	208.9344	638	1214.39	1215.5	1185.32	1801.82	1849.9	1567.293	1967.45	1038.02	926.957
639	208.9344	626	1638.1	1245.8	1262.15	1905.8	1280.65	1464.195	1826.58	1062.81	978.86
640	208.9344	368	821.195	685.75	758.004	985.71	983.249	852.3012	1330.44	874.669	724.395
641	208.9344	219	1177.2	906.09	936.87	1490.3	1096.74	1121.58	3479.58	1412.13	718.914
642	208.9345	1112	921.893	632.54	503.853	643.95	813.829	861.5867	874.947	926.562	762.067
643	208.9345	1007	1891.46	1608.9	1889.9	2180.08	3194.31	2114.245	2387.34	2184.52	1664.43
644	208.9345	949	2889.18	1937.3	1985.82	2368.34	3073.03	2381.618	2224.05	2217.35	1830.82
645	208.9345	455	747.799	420.39	570.63	808.063	604.625	651.9203	918.896	552.528	444.119
646	208.9345	247	1600.31	746.8	1610.34	1296.59	1156.12	1266.903	5055.05	1008.71	790.025
647	208.9346	985	1638.99	1477	1578.54	1993.09	2106.65	1996.975	2293.14	1871.9	1425.76
648	208.9346	733	1700.86	1466.6	2303.33	2628.51	3283.83	2327.556	3577.52	995.433	1396.3

649	208.9346	272	1032.41	760.87	1093.95	1171.01	842.037	1180.234	3182.22	1019.25	893.146
650	208.9346	71	879.674	3966.5	1354.35	1820.84	5294.48	2234.461	4884.37	2200.07	2300.31
651	208.9349	688	1062.61	916.4	1105.58	1222.39	1191.69	1292.444	1682.44	1024.42	974.086
652	208.9349	38	1256	685.37	1426.43	1447.85	870.653	1192.411	2635.78	1036.48	622.42
653	208.9351	335	573.258	592.33	637.541	626.502	943.828	753.5534	1115.83	696.859	511.193
654	208.9353	940	1783.15	1661.9	1686.47	2159.96	1989.92	1922.852	2320.42	1911.34	1583.06
655	208.9353	19	919.07	1000.7	1000.04	1206.36	1271.51	960.9393	4637.6	1328.25	747.004
656	208.9844	62	904.587	462.5	1446.23	1080.16	1108.71	737.0891	1686.11	916.005	503.616
657	209.029	70	970.574	445.4	1333.37	1086.66	1043.08	740.37	1252.88	861.562	502.814
658	209.9342	72	86.5583	307.95	117.015	156.195	455.618	273.6408	844.971	221.925	253.68
659	209.9484	57	923.875	280.79	1695.43	2879.95	4041.97	1913.888	971.015	925.1	1386.31
660	210.0765	78	886.355	274.67	343.736	740.148	832.2	510.1841	686.86	323.555	224.833
661	210.842	71	19082.5	14475	17971.4	13269.8	15305.7	11683.44	42567.1	17483.2	14188.5
662	210.9311	1058	845.56	1110.9	955.591	1276.9	1292.06	889.84	1359.83	1079.63	1030.98
663	210.9311	898	3037.6	2589.1	2551.36	3308.56	3238.12	2566.994	3465.22	2863.38	2441.85
664	210.9311	144	1296.48	1130	1481.74	2076.97	1536.33	1519.596	3705.2	1942.49	1070.95
665	210.9313	1031	1407.31	4161	4034.95	5737.32	5376.44	4879.44	5335.54	4803.66	4671.05
666	210.9313	917	3353.02	3003.1	4413.7	4337.96	3861.59	3864.237	4197.27	2531.23	3049.12
667	210.9317	72	452.742	1825.1	647.955	1031.4	2430.4	1174.085	1893.88	1008.47	966.844
668	212.8391	70	9608.26	7374.4	7887.86	6504.13	7492.81	6081.059	19909.4	8773.99	6947.21
669	212.9681	70	804.65	1054.1	1058.97	412.687	587.374	360.9449	913.333	622.993	558.138
670	213.0489	143	355.469	201.08	257.299	830.249	923.826	713.4517	2387.51	1116	635.768
671	213.0489	128	475.99	269.99	338.168	1159.35	1537.26	1340.136	4597.77	2001.79	1052.37
672	213.049	102	1595.19	697.41	752.719	6803.19	2930.43	3884.025	8279.77	3519.96	3604.36
673	213.0492	158	255.959	176.42	220.861	501.757	495.948	519.144	1841.71	908.726	395.366
674	214.0331	250	4687.35	1994.9	5177.8	2642.75	2616.22	2836.096	6635.12	3846.66	2019.41
675	214.0482	222	927.679	384.16	1135.54	557.085	333.912	393.4435	927.013	562.67	323.687
676	214.0482	198	1435.45	436.47	2356.67	282.709	272.232	336.5877	2303.83	916.563	442.509
677	214.0483	151	2349.06	1007.8	1922.49	633.114	637.148	626.4725	2637.41	1423.36	955.123
678	214.0487	175	3109.43	1059.4	4595.34	640.389	542.024	773.3099	1898.81	1817.1	1020.71
679	214.8356	70	2009.23	1570.3	1907.45	1577.91	1875.44	1312.167	4572.14	1891.92	1579.16
680	214.9882	63	2673.47	8326.9	20082.4	1329.08	2311.74	6765.617	2084.28	16476.6	7347.51
681	215.0319	152	300.533	174.74	281.217	416.381	292.149	285.5063	1244.18	377.842	195.838
682	216.0357	70	1526.29	989.06	1764.36	894.149	972.248	843.732	2969.58	1530.62	1152.6
683	216.9844	63	1287.96	5534.3	10555.7	15702.5	27723.2	7244.214	168215	82032.9	42553
684	217.0298	70	2882.87	4042.1	7626.88	16925.9	29185.5	7075.799	182495	86118.4	44850.4
685	217.0486	69	21080.4	5737.6	7401.81	15741.8	28870	6043.497	176034	86055.3	44854.3
686	217.0824	69	935.785	953.4	910.921	1480.91	2376.72	1817.809	1312.07	1261.48	1152.14
687	218.0516	68	1955.03	4652.5	9936.44	1249.64	1627.37	3464.401	13900.9	5094.47	3443.86
688	218.0666	72	5735.14	3189.4	7436.89	2987.36	2999.24	2738.381	16074.1	5505.71	2993.42
689	218.1035	64	6989.23	1919.9	10145.3	3320.8	1995.3	3118.499	7921.14	3693.24	2021.92
690	218.874	67	5995.01	3398.3	4893.72	4961.22	4001.26	2871.911	8992.49	4272.89	3255.96
691	218.9628	1019	87.04	297.35	326.39	361.76	394.018	938.4332	361.485	89.5467	65.205
692	218.963	923	3204.34	1031.5	1047.09	1051.92	1439.07	2345.929	1175.52	99.3293	953.962
693	219.0452	69	7979.16	2538.1	3175.37	5556.18	9725.95	2656.134	62838.6	25601.2	14961.5
694	219.1069	62	856.508	410.42	812.953	567.608	394.431	481.5089	855.615	534.548	261.07
695	219.8446	58	2320.85	4447.7	2374.69	2636.41	2812.74	2839.798	2654.34	2272.42	2779.72
696	220.0489	70	926.65	363.59	492.231	698.955	1052.84	342.0698	4867.16	1931.27	850.843
697	220.8706	67	5765.12	3082.3	4798.28	5182.35	3815.36	2723.949	7144.45	3084.48	3145
698	221.0121	66	3066.64	549.02	3617.37	867.674	1031.95	1273.442	2607.66	1722.87	1311.77
699	221.0483	68	2202.95	674.81	2483.46	1111.44	1422.62	1238.08	1382.46	1792.08	1184.45
700	221.1549	91	365.175	194.37	254.987	489.784	581.504	216.5747	845.17	336.382	211.558
701	221.8415	58	1551.09	2475.6	1636.38	2073.16	2003.53	1937.886	1970.47	1399.82	1700.4
702	222.0015	37	795.825	767.21	827.755	1382.78	1203.47	494.421	4351.6	660.289	622.154
703	222.0017	1126	889.963	982.16	775.429	966.8	1292.45	1558.22	1008.51	1019.86	944.429
704	222.0017	5	1160.23	1014.3	548.055	1777.16	1035.33	663.8248	1725.54	1065.84	743.396
705	222.0018	1103	694.256	720.23	663.601	811.73	1119.19	745.8462	1190.58	927.004	783.285
706	222.0018	1078	558.501	662	532.716	944.093	957.451	756.15	834.869	649.628	750.59
707	222.0018	204	735.889	807.45	792.818	919.984	826.355	909.7503	2120.11	1303.84	597.493
708	222.0019	1152	274.354	404.81	263.432	241.362	595.307	584.3127	509.317	646.254	429.444
709	222.0019	227	358.953	555.5	490.611	643.061	638.995	645.9224	1290.06	799.94	387.18
710	222.0019	112	1957.23	1742.6	1663.08	3371.01	1701.94	2108.291	4346.28	2363.95	1351.48
711	222.0026	156	854.868	685.15	617.285	974.817	841.051	757.61	1796.37	1047.47	543.963
712	222.0027	133	863.512	676.57	797.686	1140.18	1055.1	958.8659	1621.03	954.469	614.272
713	222.8673	67	2214.87	1222.3	1630.66	1519.21	1372.46	1060.944	2382.24	1402.19	1152.8

714	222.9822	88	1049.86	691.31	910.602	1824.61	1703.43	1272.408	2447.61	1066.08	629.876
715	224.8187	70	1266.96	863.47	1558.76	897.093	1082.47	747.0397	3092.54	1080.33	849.522
716	224.9984	62	723.468	346.03	946.523	1338.04	3439.02	458.8118	2087.84	1532.28	933.181
717	225.0162	62	3112.75	350.64	798.52	1121.35	3252.52	425.4437	2323.02	1353.22	722.796
718	225.0612	96	7125.06	2800.6	8133.42	5176.85	5385.98	4718.262	9888.82	3189.8	2873.7
719	225.0612	63	1651.11	1296.7	2211.01	1676.56	1563.91	1050.986	3497.56	1863.43	814.209
720	225.0615	33	256.312	209.71	221.212	495.043	367.793	353.8506	1166.06	301.041	178.183
721	225.062	18	313.823	267.59	281.527	595.81	471.842	372.7373	1739.02	370.217	220.769
722	225.0623	3	142.225	82.592	57.0662	206.309	152.085	81.481	497.003	86.6097	43.7374
723	226.816	69	1783.25	1320.6	1948.24	1052.28	1596.77	1049.998	4637.92	1574.34	1276.99
724	226.9685	70	1423.81	971.2	1498.39	895.552	1111.51	1401.734	1845.56	1158.54	1193.95
725	227.0769	62	1219.03	677.38	608.016	663.092	1252.07	482.6597	7905.39	3358.18	2104.27
726	227.2012	1115	488.624	481.96	399.093	714.56	801.182	489.0914	677.319	646.336	581.691
727	227.2018	148	401.733	303.14	307.85	648.487	442.864	216.3507	678.014	261.712	217.408
728	228.0817	60	361.92	256.61	346.303	310.846	351.769	245.2596	957.204	451.993	398.419
729	228.1605	66	261.251	99.141	242.089	753.527	1118.28	246.2592	594.05	391.351	200.225
730	228.8134	69	1088.12	773.51	1076.76	697.331	901.784	602.6975	2163.15	930.161	679.124
731	228.9022	66	10435.6	5698.6	7868.02	9733.75	6232.98	4584.431	12621.2	6391.94	5823.46
732	230.0388	156	211.938	118.88	92.5829	574.93	469.25	581.5717	1244.3	951.688	451.389
733	230.039	145	234.337	89.654	95.7792	689.897	498.589	511.9781	911.388	659.019	344.78
734	230.0394	119	382.17	211.9	176.645	1337.48	1264.1	1508.601	3281.89	2273.33	920.073
735	230.8997	66	7004.12	3592	4958.57	5832.19	2706	3257.163	6918.8	3734.13	3358.28
736	231.023	311	450.364	189.26	318.558	154.277	186.598	260.2286	352.806	293.271	223.083
737	231.023	289	1139.41	310.83	702.074	288.36	356.812	454.0822	762.598	451.483	416.298
738	231.0232	248	2229.18	390.66	1206.04	635.837	670.918	777.6123	4034.06	1086.79	657.454
739	231.0233	268	1565.05	311.45	1717.48	500.322	478.55	627.9	1207.77	1345.48	442.953
740	231.0233	223	763.906	181.78	267.42	414.773	276.816	336.491	866.975	298.489	196.759
741	232.0577	80	596.091	394.04	634.776	237.999	505.809	371.9803	1726.53	535.101	222.631
742	232.8961	66	1270.41	1733.3	1141.29	1278.4	1860.49	1117.787	1243.15	1338.55	1285.49
743	232.9239	147	802.52	471.77	714.672	679.11	574.094	802.1376	1548.99	1072.03	511.488
744	232.924	1142	1102.97	638.57	969.456	1071.56	1487.48	1108.155	1261.83	1303.93	1249.54
745	232.9241	1061	857.827	575.67	701.095	1173.79	1333.05	992.609	1086.31	1342.8	1038.64
746	232.9243	1031	1290.62	2047	1844.71	3148.15	2174.35	1290.102	1328.98	1572.62	1831.12
747	232.9244	917	2050.46	3317.7	2840.15	1519.2	2945.29	5224.364	2995.16	2046.25	1685.43
748	232.9245	2	182.866	107.97	268.895	289.08	177.683	140.8669	584.655	304.406	95.3335
749	232.9246	1175	665.865	882.14	485.228	1197.44	922.21	1148.388	666.859	793.445	1009.96
750	232.9246	73	1233.75	1372.1	1016.29	1225.41	1179.74	1067.243	1328.59	1144.96	921.227
751	232.9247	49	875.38	756.94	821.435	882.268	988.779	780.2777	1765.01	960.221	643.607
752	232.925	1200	0	0	124.631	0	0	0	0	0	0
753	232.925	18	802.427	770.29	828.897	879.3	884.178	739.1772	2088.43	882.304	594.873
754	233.1535	93	1112.07	830.28	1164.13	1351.86	1288.32	1104.632	3162.34	1518.66	816.919
755	233.1545	1	0	290.88	0	0	390.717	0	1925.45	0	232.594
756	235.9525	2	0	437.27	0	0	635.722	0	2383.3	0	337.636
757	235.9527	1057	608.004	602.36	577.486	827.86	925.806	716.0818	1019.38	769.854	670.006
758	235.9532	918	3447.69	3146	2994.94	4388.66	4183.84	3508.064	4223.47	3359.17	3010.05
759	235.9534	1071	589.141	529.03	549.108	989.847	954.729	647.221	965.036	565.888	602.276
760	235.9534	1033	1727.82	1425	1430	1019.14	1161.92	2538.855	1920.74	1265.83	1236.29
761	236.0155	252	3054.68	2059.9	3219.16	3019.14	2925.59	2847.969	4819.54	3302.47	1993.79
762	238.9312	79	17267.5	7257.2	15249.8	24442.4	10822.2	13088.31	53304.9	18095.3	7055.45
763	238.9315	60	4632.41	4878.5	2509.83	4946.27	7232.38	2233.01	15383.2	5181.15	4559.27
764	239.0663	83	537.515	305.31	437.138	692.354	629.651	627.1341	1909.26	861.98	435.126
765	239.9243	71	856.238	339.17	959.787	786.769	479.182	716.1453	1791.51	700.19	348.755
766	240.9283	61	2077.42	1552.2	1018.08	2071.12	2247.29	990.7448	5142.26	2092.36	1770.39
767	240.9285	80	6171.97	2548.7	4750.07	8269.93	4468.83	4913.732	17882.3	5625.75	2021.78
768	241.9214	70	844.391	390.04	759.959	678.015	547.371	605.3293	1847	788.472	494.475
769	242.0789	98	1711.41	857.79	2257.42	1070.79	1180.2	1164.331	1749	1414.5	711.923
770	242.1759	68	1557.47	710.5	1765.5	4769.57	7845.86	2255.627	5624.53	2379.65	1938.2
771	242.943	204	379.884	262.13	297.706	521.612	363.954	419.9378	1453.93	471.654	219.591
772	242.943	153	456.127	307.17	252.817	611.565	426.067	424.2844	1275.01	496.155	255.936
773	242.9431	250	252.531	208.03	250.139	370.578	370.036	279.072	992.142	279.528	177.752
774	242.9433	96	1672.89	1279.8	1057.42	2243.75	3417.13	1565.461	4782.91	1540.77	966.258
775	242.9434	1116	759.389	687.06	705.698	937.428	1206.13	991.3898	1400.52	936.905	869.574
776	242.9434	35	74.0939	282.09	60.7434	148.712	337.841	110.161	533.051	187.872	231.495
777	242.9436	3	310.959	577.31	207.285	871.373	528.147	267.4022	1326.27	302.252	344.456
778	242.9437	227	187.871	205.29	179.767	345.202	307.729	335.2591	1112.75	329.496	177.437

779	242.9439	25	577.753	324.3	434.13	776.872	477.853	526.7117	1493.77	390.96	282.548
780	243.0622	62	3901.42	1198.5	5442.46	1026.21	1395.04	1142.609	5225.2	2484.48	1411.21
781	243.1797	68	340.63	195.4	412.094	907.825	1342.75	415.3853	989.488	450.955	346.66
782	244.0665	62	553.571	263.81	548.688	405.44	728.853	280.0182	3342.97	968.271	399.663
783	244.874	78	508.022	138.11	640.696	519.704	389.695	304.6857	1162.85	408.776	159.588
784	245.0421	166	302.189	221.71	266.185	275.311	278.109	229.2423	648.092	336.668	241.456
785	245.0427	145	1050.21	344.86	1164.58	480.144	495.778	370.7822	1516.21	911.221	483.356
786	245.0428	120	2011.66	646.51	1877.27	1202.78	832.912	730.2208	2699.89	1192.87	728.358
787	248.0531	99	972.717	434.17	858.139	405.252	433.848	502.3554	1629.54	1007.45	419.125
788	248.1662	84	1262.89	511.87	885.935	965.772	870.75	870.1461	2744.23	1386.78	596.228
789	248.9602	85	121625	55704	101392	114735	86742.5	95909.66	494500	140446	54785.9
790	248.9786	70	6372.6	4957.5	3632.19	7711.64	8099.13	4681.736	1178.74	6094.86	4288.38
791	249.9633	84	6517.43	3059.4	5225.11	6013.42	4756.39	5548.071	27680.6	6899.84	3557.4
792	250.9641	85	2755.27	1513.6	2070.52	2362.62	2146.78	2020.136	10024.2	2751.54	1249.85
793	252.946	80	418.829	304.66	350.347	428.389	306.952	233.496	1075.89	552.217	203.533
794	253.0504	63	5781.21	4099.1	3187.09	5388.32	6351.82	4074.378	11557.4	5613.02	5275.22
795	254.0251	100	1880.72	530.22	1687.44	911.169	770.891	847.0706	3266.61	1336.25	618.155
796	254.0534	63	1114.99	1057.6	820.13	611.502	1229.4	739.5877	2036.21	1137.95	988.597
797	254.0788	67	1329.41	1028.1	1157.81	996.291	1133.86	774.0778	812.901	1210.52	905.34
798	254.9049	75	1597.54	681.24	1717.87	1617.63	989.363	952.3704	3066.64	1512.47	599.442
799	255.2329	211	1607.34	780.12	1958.29	2182.82	1296.89	1172.709	6308.21	2092.31	777.605
800	255.2331	1191	434.174	410.07	256.887	605.172	679.68	541.8	732.192	0	527.175
801	255.8219	58	6152.85	10116	5736.84	7997.4	10130.4	6888.922	9416.86	5834.02	6319.66
802	255.9224	62	78.81	77.042	64.0822	143.289	388.08	58.59	692.567	257.282	146.72
803	256.2358	210	306.605	161.97	336.072	354.732	247.439	244.0854	1296.5	442.477	160.138
804	256.9034	79	431.824	158.39	577.757	647.589	421.418	303.9093	1793.28	277.32	133.803
805	257.818	58	5791.82	8587.4	5628.76	7954.77	9665.36	6746.23	8726.41	5864.72	5742.1
806	258.9216	72	273.656	956.39	262.256	289.679	871.268	367.344	739.204	350.64	501.191
807	259.0909	98	1042.69	274.52	1850.89	301.153	344.417	435.6527	1298.97	799.687	404.853
808	259.8166	58	1799.26	3136.5	2338.1	2866.36	2824.76	2442.626	3548.7	2119.39	2109.87
809	259.9812	83	795.22	357.95	839.563	532.311	687.757	652.3331	2906.01	912.753	346.642
810	262.9754	85	3730.13	1668.1	2752.16	2972.24	2995.04	2668.852	13782.9	3924.74	1410.88
811	264.9338	83	4596.2	1423.2	4976.68	4545.53	3137.24	2993.358	15291.3	4372.12	1782.63
812	265.1464	1068	221.364	297.92	272.333	362.706	392.124	281.3812	361.784	273.879	291.75
813	265.9491	89	1288.06	850.95	1501.09	1178.82	1318.1	1049.152	3244.2	1515.13	767.473
814	266.037	100	905.375	377.16	945.108	343.024	343.951	442.0675	1676.24	584.322	279.591
815	266.8037	71	25648.4	18322	21897.4	17283.8	20398	15240.09	66749	23599.1	16862.9
816	266.9316	82	296.387	216.34	379.262	426.042	265.403	330.0532	1569.88	385.908	151.378
817	267.0251	97	6467.99	182.55	412.069	3191.91	4590.48	253.2915	4829.03	1674.58	1320.33
818	267.0733	66	10176.9	1662.5	12631	1535.72	2167.95	1924.032	13246	5142.21	1257.1
819	268.0768	61	1477.96	483.36	2033.84	340.565	400.801	406.0742	2080.4	782.2	498.669
820	268.8007	71	42196.3	29564	37323.4	29542.7	32109.3	24837.89	109758	40661.4	27403.8
821	269.0422	62	748.773	73.5	160.095	149.207	355.111	101.985	592.68	187.688	147.56
822	269.0874	95	1096.42	613.61	638.52	2216.84	1807.44	1043.303	4479.13	1178.73	485.44
823	269.1321	81	947.411	398.62	606.918	796.898	1190.91	661.2554	2567.41	870.409	386.481
824	270.7976	71	27135.5	18817	22577	18099.3	20757.7	16204.81	71445.7	25432.5	17145.8
825	271.103	62	1417.29	215.92	626.941	750.166	881.092	279.27	7204.73	2157.48	1283.63
826	272.0848	112	224.604	107.77	185.217	335.451	210.265	222.5545	878.299	334.966	201.625
827	272.1071	62	198.69	79.654	146.474	82.2942	94.08	85.932	933.517	355.232	174.23
828	272.7945	71	8396.58	6178.5	7060.64	5779.24	7184.15	4874.013	23571.4	8067.03	5679.04
829	272.929	63	482.044	182.67	377.888	191.837	285.722	225.0743	1216.7	343.029	202.84
830	274.7915	69	1346.53	1055.4	1159.02	1039.77	1408.37	824.7098	3895.83	1402.58	992.908
831	275.0547	71	934.727	351.96	960.578	448.827	393.869	335.563	1578.41	561.507	341.517
832	275.1675	88	192.761	57.936	34.3496	289.214	733.128	297.942	469.195	133.895	126.133
833	276.8321	67	5114.16	2518.9	3644.24	3771.5	2595.47	2091.708	7460.82	3018.56	2280.01
834	278.094	68	370.047	165.86	370.592	315.229	251.935	186.7376	912.214	577.282	276.982
835	278.1522	68	364.571	177.55	320.529	893.408	1162.45	341.1354	1282.24	605.823	314.618
836	278.829	67	6071.66	3039.8	4601.15	5047.89	3254.6	2516.971	12337.8	4143.28	3052.51
837	279.0383	63	5925.1	1000.9	16398.1	967.74	839.055	1551.419	5414.69	2007.12	1112.02
838	279.0887	68	1308.96	145.96	613.652	489.094	893.944	295.7956	4070.53	1580.13	999.949
839	280.0416	59	749.978	157.07	1858.35	146.549	118.985	269.2214	778.01	326.414	140.598
840	280.8258	67	3238.43	1702.8	2171.04	2573.61	1628.86	1189.144	6386.98	2035.18	1282.26
841	281.0349	101	199.788	107.81	178.684	894.062	531.47	527.0268	1801.61	638.895	344.355
842	281.0365	59	1391.23	340.68	3546.66	396.877	484.713	475.6573	2266.6	710.379	310.71
843	281.2485	221	524.027	351.62	698.212	524.774	720.209	595.7488	2956.87	802.916	495.833

844	282.7773	71	3743.67	2959.5	3932.66	2407.17	3480.18	2434.742	11168.4	3627.16	2925.35
845	282.9569	62	333.2	194.99	425	347.698	276.267	270.135	1590.48	587.44	304.5
846	283.2638	336	974.544	740.87	1154.65	1906.84	1474.13	1319.337	1664.64	1010.68	728.514
847	284.051	71	119.34	91.494	127.49	114.38	222.778	89.0966	973.668	448.052	209.824
848	284.7744	71	6273.58	4927.3	6701.46	4221.93	5162.13	3638.953	18223.8	5922.66	4435.63
849	285.0448	71	312.55	204.12	223.179	323.853	980.528	288.7875	3728.2	1236.12	955.329
850	286.7719	71	4506.32	3687.4	4563.02	2888.63	3738.32	2476.036	12054.4	4282.43	3146.33
851	286.8615	67	5278.07	2674.4	3928.66	4915.18	3703.24	2340.124	6106.14	3715.98	2703.45
852	286.9109	1063	665.714	888.16	752.933	1238.55	951.192	694.0736	3512.36	947.642	1061.83
853	286.9109	145	973.97	783.36	1020.91	1717.85	1144.18	1148.804	3747.07	1567.64	738.523
854	286.9111	1036	1445.22	760.14	1339.06	2127.32	1898.97	898.2887	1670.8	1489.69	1273.95
855	286.9112	916	2170.5	2277.9	3289.38	1696.58	4560.82	1305.15	2410.63	2133.13	2357.13
856	286.9113	220	466.836	422.57	449.808	692.321	543.995	470.9158	1936.82	652.897	352.401
857	286.9115	1187	543.578	313.64	351.218	888.742	417.049	622.0215	597.956	429.169	378.82
858	286.9118	1170	703.081	721.24	425.361	768.29	1041.22	1045.8	1276.87	919.253	929.323
859	286.9119	1146	945.993	915.08	824.014	1104.89	1282.99	976.1409	1267.59	990.36	935.865
860	286.912	193	438.066	370.17	454.408	515.154	371.735	467.088	1473.13	615.493	291.103
861	286.912	73	4587.61	2508.8	3728.31	4727.05	1044.13	2421.111	12314.2	3746.68	2196.61
862	286.9121	1157	891.126	320.27	547.2	963.875	460.567	851.1935	771.318	622.01	326.071
863	286.9124	252	750.035	478.48	762.976	656.637	646.399	658.5106	2794.26	802.713	445.65
864	288.7685	71	1443.05	1289.7	1711.16	1304.53	1298.8	950.6703	4662.42	1538.55	1223.13
865	288.8577	67	5517.36	2708.5	3947.36	4812.22	3269.31	2354.658	10306	3066.48	2125.27
866	288.9074	47	282.46	234.58	243.982	301.266	313.107	275.6767	1013.55	344.235	178.484
867	288.9086	71	5217.63	1858.8	3667.18	4371.84	2239.45	2320.325	891.759	1964.99	1529.56
868	288.9096	18	274.294	226.71	301.585	294.993	347.197	265.8519	1166.11	362.314	188.071
869	289.1672	69	211.47	168.2	371.531	561.333	794.214	228.7409	688.94	356.07	212.782
870	290.7616	72	372.6	359.71	387.679	226.135	274.591	202.0659	902.325	504.487	218.168
871	290.8548	67	1979.51	803.28	1250.97	1843.81	1227.34	786.7279	4203.17	1346.38	972.91
872	290.9703	87	258.446	186.11	92.3618	754.67	652.657	524.2841	2020.89	449.034	122.089
873	292.9858	62	354.32	170.28	387.051	222.329	387.179	258.39	1538.14	490.563	219.327
874	292.9869	87	638.079	288.4	582.067	479.893	311.05	397.2564	1820.22	582.704	183.056
875	293.1761	1042	5465.01	5003.5	4190.6	7331.41	7265.6	5684.52	9809.95	7286.84	6214.42
876	293.1765	139	1002.2	704.56	904.974	1239.42	1256.27	964.5076	5242.7	1223.02	687.873
877	293.1786	82	1062.63	1032.3	1259.21	2354.68	3214.49	1441.335	2346.77	1476.08	1044.24
878	294.1791	1042	1169.64	1135.1	1051.41	1535.13	1377.39	1250.271	1949.79	1468.84	1320
879	294.8019	64	1098.13	319.35	1089.8	934.515	340.973	553.47	1490.75	635.043	302.1
880	294.9938	70	469.245	169.41	289.635	879.748	734.191	284.2416	1229.13	366.029	209.657
881	296.8899	67	6877.9	2158.9	4912.82	7558.77	4354.91	3040.079	14567.8	5589.45	2217.89
882	297.1334	63	2572.12	324.53	526.733	1174.47	4173.73	326.3752	3351.35	1073.36	696.732
883	297.8829	71	978.059	516.49	943.426	721.047	659.451	541.6099	2037.53	838.414	444.575
884	298.1368	62	423.992	100.12	206.137	325.069	899.789	134.5465	742.678	275.958	160.012
885	298.752	72	369.74	123.16	256.057	290.667	287.863	147.8643	1015.55	251.674	93.0922
886	298.887	64	4433.54	1950.4	3147.09	4921.68	1980.64	2207.455	6511.96	2754.53	2172.94
887	298.9887	67	713.83	117.89	714.371	347.515	210.826	294.7313	994.526	388.475	232.98
888	299.2021	118	318.409	101.78	242.034	332.52	513.542	117.1966	1305.52	360.167	152.853
889	299.8792	71	799.223	395.29	793.174	691.145	602.108	551.3761	2583.94	914.418	382.394
890	300.7475	71	728.867	527.69	887.068	425.62	661.803	382.394	1496.56	605.723	489.448
891	300.8839	78	270.871	164.33	287.616	355.904	515.592	107.7867	1372.78	331.17	161.414
892	302.0985	58	395.924	227.76	370.995	218.31	476.7	187.6643	760.842	256.195	170.064
893	302.1002	123	432.641	258.57	689.839	183.601	250.813	182.925	1223.51	463.21	207.181
894	302.1002	95	1533.3	679.49	2096.57	590.729	985.254	953.8117	3254.3	1129.9	472.381
895	302.8342	76	627.717	192.59	790.849	580.923	529.284	292.817	1577.87	388.065	196.827
896	303.0486	65	1710.79	602.39	2761.66	454.317	545.129	538.0871	3435.4	982.982	563.492
897	303.2324	153	471.824	181.72	530.967	371.114	298.679	553.7343	1222.4	318.698	305.167
898	304.8328	63	975.724	398.8	968.257	817.556	553.829	421.5482	1484.25	739.346	374.685
899	304.9124	1015	278.901	268.24	288.951	401.486	364.323	372.0224	429.831	358.369	269.461
900	304.9133	975	586.677	541.73	593.093	743.322	735.095	651.178	855.495	714.019	528.262
901	304.9136	956	714.939	719.84	698.916	2910.87	922.795	579.0725	160.758	776.55	719.04
902	304.9137	994	453.791	408.78	554.011	590.085	545.965	539.9936	698.797	81.3827	410.85
903	305.0475	68	726.517	242.37	1374.59	293.532	249.804	315.7401	1681.38	497.313	298.378
904	306.0747	449	1402.91	443.49	957.589	994.047	595.543	1138.223	1201.88	792.45	586.981
905	306.9178	60	2896.46	2642	1256.12	2683.53	4358.28	1400.392	11022	3518.72	3018.67
906	306.9184	81	16137.7	5516.5	9571.99	16877.2	7447.68	7982.857	42756.2	11794.8	5223
907	307.923	80	817.444	290.96	559.107	703.878	583.225	440.0449	3074.74	678.571	327.473
908	308.9154	60	1153.72	1027.7	587.172	1193.18	1549.02	520.8936	3700.9	1312.31	1119.83

909	308.9155	81	5512.7	1972	4783.22	6424.1	2706.15	3718.724	16608.7	5539.55	2029.63
910	309.1718	74	422.215	223.5	293.052	451.365	1236.47	444.5833	989.963	404.235	264.546
911	310.9302	94	698.225	621.35	481.074	1233.43	877.671	875.7124	2281.04	728.07	569.35
912	311.1679	1042	1622.29	1373.4	1329.61	2072.97	1873.84	1607.414	2684.87	1562.63	1536.89
913	311.1687	87	726.529	529.7	742.802	1003.47	2010.27	962.2884	2464.05	736.793	631.278
914	312.8634	71	1198.65	1092.3	1417.02	1293.93	1438.11	954.3951	2418.87	1272.38	900.85
915	313.9296	167	122.4	168.23	122.866	172.068	215.949	220.806	814.543	264.329	131.5
916	313.9303	12	768.795	550.83	959.528	1259.54	792.622	737.1994	2880.91	922.909	418.226
917	313.9304	39	667.03	555.49	665.642	873.213	957.306	703.1534	2021.59	760.781	469.938
918	313.9305	1036	1839.48	1673.2	1570.99	1019.65	2949.98	1722.054	2106.21	1735.68	1530.16
919	313.9305	915	1100.36	926.49	970.103	892.15	1390.39	1099.763	1079.23	1009.94	958.523
920	313.9305	192	428.069	384.92	435.071	615.213	494.072	500.9037	1797.35	618.3	276.943
921	313.9313	236	1133.44	691.31	1084.81	1188.46	1092.25	1073.166	4207.93	1145.35	672.447
922	314.1466	60	1016.63	245.39	1127.18	186.246	190.931	146.8837	462.225	321.497	234.275
923	314.8626	62	563.934	314.16	404.282	462.746	346.792	283.5313	1002.6	345.48	368.315
924	315.1961	89	812.841	345.2	703.74	834.041	829.254	432.7845	732.914	314.097	538.926
925	315.9252	83	719.509	299.57	563.251	565.439	727.436	558.6633	1137.96	823.312	444.317
926	315.9473	87	641.883	299.66	469.305	486.665	500.957	461.0563	1050.8	971.866	365.307
927	316.0416	95	326.522	192.55	145.524	129.611	249.811	202.098	1081.58	322.486	86.7613
928	316.9473	85	114955	53445	93857.6	67387.5	76341.5	85742.4	426892	138897	57642.9
929	317.9503	85	7608.91	3619.3	6422.9	6594.91	5274.14	6073.885	29651.3	8614.62	4233.88
930	318.9285	1008	409.683	205.43	190.762	204.1	210.91	215.1545	213.486	530.429	206.623
931	318.9288	982	639.236	873.31	1087.61	686.297	917.426	702.0213	1040.64	1468.62	565.034
932	318.9289	959	1247.23	1780.9	607.787	2141.92	3378.78	624.9416	251.962	663.662	649.899
933	318.951	86	3229.95	1546.4	2604.71	2421.96	2437.08	2327.55	11321	3793.74	1630.45
934	320.9335	80	1342.68	383.06	710.508	861.09	530.971	453.402	2165.88	800.823	366.241
935	321.0742	68	322.607	203.06	322.785	461.524	468.993	257.4828	1420.64	264.734	162.229
936	321.2094	99	669.318	228.77	684.885	864.268	866.722	709.9459	1323.87	488.665	440.38
937	322.0122	91	3554.41	1033.9	3266.79	1522.32	1043.61	1734.45	3553.7	2595.79	1331.66
938	322.8549	63	864.396	478.35	626.289	454.686	663.13	300.9714	892.688	736	596.666
939	322.8921	79	3142.43	936.13	3456.35	2969.29	1735.88	1673.556	6232.12	1748.66	1270.42
940	323.028	316	790.713	288.18	671.327	645.988	486.513	497.3507	5023.59	669.688	351.547
941	323.0282	430	447.222	149.19	421.605	157.929	169.214	205.459	370.63	333.469	237.262
942	323.1065	70	338.271	661.74	335.273	454.464	1166.55	292.6224	965.011	545.214	674.807
943	323.909	62	130.98	129.27	91.546	293.355	535.08	149.73	911.133	372.21	269.86
944	324.7616	70	5405.82	4260.5	4744.38	4036.53	4605.98	3170.359	14630.2	5392.49	4445.56
945	324.8893	77	1414.85	633.5	1403.97	1379.24	964.22	712.2017	3308.83	1336.38	821.772
946	325.1837	1046	3769.93	4310.8	3688.43	6397.77	4804.2	4299.75	7834.42	5621.4	5636.82
947	326.1867	1046	845.733	961.16	930.561	1408.39	1043.07	1032.492	1757.21	1396.09	1197.8
948	326.1867	93	0	104.24	91.7056	171.806	1230.14	109.6953	483.834	429.709	376.13
949	326.7589	70	10881.7	8900.8	9087.26	8987.14	8270.35	7031.032	27849.3	11151.9	8899.05
950	327.2317	140	742.835	256.83	1039.52	362.236	685.837	1078.055	3776.47	1041.21	555.186
951	327.9686	83	3269.5	1187.7	3392.54	2017.52	1870.56	2046.157	8187.59	3861.1	1416.67
952	328.0576	407	983.946	421.19	1318.21	777.281	645.522	697.4464	525.494	807.608	428.28
953	328.0582	451	505.63	199.28	363.703	364.699	254.329	407.5783	441.775	332.007	230.054
954	328.0582	385	1053.72	510.9	633.016	1519.59	756.859	1134.495	937.971	907.767	565.126
955	328.0591	427	371.496	127.32	318.259	272.611	166.212	301.9236	353.362	255.51	174.22
956	328.2364	139	216.263	73.16	258.986	88.8939	206.871	227.9481	1162.79	264.35	155.049
957	328.7559	70	9437.72	7760.1	7389.37	6607.04	7244.94	5643.689	23129.7	9848.3	7108.97
958	329.2479	158	495.943	99.042	711.776	380.8	188.641	456.1324	999.87	418.267	153.29
959	330.753	70	4091.12	3450.9	3658.62	3067.03	3402.01	2460.87	10348.7	4194.73	3739.42
960	330.8865	63	859.919	1157.6	716.562	764.075	1452.46	649.4356	1104.41	995.769	878.956
961	330.9192	73	795.311	1203.8	632.422	765.779	1189.65	612.6214	2099.13	1084.46	794.208
962	330.9627	85	7468.62	3200.3	5968.55	5792.94	4375.57	6151.882	21122.2	8460.11	3933.1
963	331.102	139	823.79	504.97	472.338	1907.01	1346.31	1059.568	5449.1	1062.19	438.401
964	331.1023	2	219.666	182.46	192.836	1211.62	689.488	342.1179	1737.56	297.714	89.5141
965	331.1025	543	155.63	151.05	113.083	453.791	279.491	297.9125	476.783	226.707	73.075
966	331.1025	158	317.048	248.76	230.707	709.338	650.326	542.0007	2500.92	472.385	135.64
967	331.1026	1192	421.626	284.81	223.929	995.69	795.6	729.28	1363.39	0	315.758
968	331.1026	1045	426.55	407.28	367.84	986.018	907.129	712.1483	1733.58	705.499	385.2
969	331.1028	54	636.61	486.24	463.183	1792.87	1400.37	893.827	3852.71	831.749	399.585
970	331.1029	1060	732.951	694.06	733.371	2645.57	1706.72	1403.196	1924.98	1131.82	620.591
971	331.103	220	554.084	445.92	447.426	1465.6	1192.1	906.8692	4228.64	876.735	305.853
972	331.1032	307	337.661	260.57	245.308	1004.44	651.33	619.0552	1654.64	424.142	192.758
973	331.1032	36	538.436	375.43	408.025	1376.62	933.576	820.5603	3296.46	530.905	257.48

974	331.1032	14	795.487	631.67	532.964	1948.34	911.266	1158.422	7460.77	811.545	403.035
975	331.1035	1160	252.904	178.21	135.478	584.47	433.896	396.825	588.612	271.713	143.724
976	331.1035	338	262.888	259.53	224.323	988.759	786.254	606.2515	1407.37	379.056	184.795
977	331.1035	88	1323.23	1002.3	865.339	2711.79	3431.96	1934.699	15048.6	1877.34	941.334
978	331.1041	108	875.766	743.46	675.407	2793.4	2072.59	1501.143	6227.06	1215.54	510.857
979	332.7506	69	1270.47	730.3	861.562	895.158	629.548	614.4852	2585.96	1230.39	788.886
980	332.8827	64	4149.2	655.68	4729.51	2870.56	1252.29	5293.227	1177.93	3123.24	893.627
981	332.9207	83	10146.9	4270.2	12401.3	9344.76	7857.61	8999.295	32400.7	11226	6247.88
982	333.0585	454	5866.15	1338.3	9012.96	1281.93	1655.59	879.3675	4101.31	6677.81	1731.9
983	333.11	66	10776.5	1210.9	2972.32	5247.27	14631.9	1074.313	10346	4319.05	3131.05
984	333.9367	87	1214.44	1108	1187.01	1317.45	1519.56	1112.531	4468.11	1697.4	1271.6
985	334.0231	91	2825.59	881.98	3261.94	1086	1254.1	1476.191	4252.76	2028.61	1232.96
986	334.1136	66	1921.7	244.07	629.156	1087.75	2806.77	258.3579	1662.38	826.705	533.042
987	334.79	67	1479.62	694.21	1012.5	1136.56	527.442	614.5858	1890.36	1016.99	706.389
988	334.9204	83	1377.52	736.65	1253.63	1345.12	989.852	832.3604	3394.14	1097.56	612.068
989	335.0116	96	3300.12	178.05	387.73	1478.46	2584.27	299.3477	2755.02	1030.58	689.377
990	335.0597	60	1637.44	446.21	616.896	966.667	1665.36	296.555	899.329	866.163	556.093
991	335.1071	66	3670.81	730.46	1125.14	1930.22	5067.86	600.1311	4161.48	1398.65	1085.71
992	336.7873	66	2042.45	1216.7	1589.54	1505.33	1113.68	833.391	3334.68	1470.8	1249.17
993	336.8981	73	204.294	882.05	97.9417	203.363	566.765	226.9048	236.725	222.446	407.975
994	337.1406	114	459.564	198.25	446.225	508.805	225.36	371.079	1201.34	417.366	189.382
995	337.2036	93	317.567	181.11	283.387	471.434	767.265	528.456	627.706	319.76	306.971
996	337.2042	1050	1385.32	1627.6	1065.35	1416.02	2061.59	1406.858	2265.4	1718.12	2295.93
997	338.7841	66	1569.83	828.5	1184.81	996.702	832.871	585.4584	2374.16	830.171	933.285
998	338.8647	79	97.8465	129.98	211.72	101.833	320.483	69.804	832.455	208.26	105.663
999	339.199	1049	2388.98	2159	2196.45	2583.56	2368.33	1694.919	2735.7	2163.65	2778.64
1000	340.2025	99	447.288	176.3	502.241	520.222	979.791	534.8839	899.247	493.434	314.339
1001	340.7358	71	1768.29	1373.4	1513.45	1135.35	1212.37	1027.856	4285.13	1525.48	1335.14
1002	340.9147	63	895.985	426.03	679.373	528.578	712.988	449.6958	1583.19	917.125	663.91
1003	341.1073	115	517.403	436.32	793.966	652.674	632.682	374.4471	1054.57	687.615	446.294
1004	341.1082	96	2100.62	1228.4	2066.49	1231.83	1405.87	1168.049	2954.45	1403.41	1328.3
1005	342.7325	71	3497	2729.6	3400.82	2292.04	3074.97	1787.313	9954.97	3670.42	2849.54
1006	342.9134	63	436.73	172.74	352.407	317.274	398.086	259.695	1162.48	555.97	527.625
1007	343.1387	63	5144.97	931.18	989.324	2667.96	10570.7	674.4772	6332.78	2698.58	1914.3
1008	343.9934	132	15.241	31.292	11.4193	776.628	821.598	141.9356	290.372	175.677	82.1701
1009	343.9939	112	33.2128	89.036	88.7575	5001.21	2580.67	468.423	883.457	458.951	249.378
1010	344.1423	62	758.282	180.68	250.415	510.484	2029.53	169.9643	1493.39	526.904	337.813
1011	344.7297	70	2952.09	2377.9	2843.81	1852.91	2429.54	1677.103	6950.23	2929.3	2293.95
1012	344.8187	64	2260.45	1185.9	1608.88	1852.42	1537.79	982.5951	2977.58	837.942	1636.89
1013	345.9741	93	1307.06	702.21	928.641	1051.67	936.271	744.65	2170.11	912.485	911.888
1014	346.0547	546	878.47	408.24	1000.84	393.583	403.906	494.1186	601.167	635.703	373.309
1015	346.0548	522	2041.23	991.28	2544.91	1016.08	1171.03	1225.32	1881.8	1703.94	988.013
1016	346.0548	433	2253.96	557.06	848.86	841.737	1017.99	1303.209	2719.59	1329.2	804.755
1017	346.055	500	855.252	472.18	1360.32	575.768	677.379	723.3563	1098.19	925.559	617.333
1018	346.7272	70	1225.13	998.66	1304.21	1047.92	1252.88	893.6566	3722.18	1628.77	950.913
1019	346.8162	64	2822.69	1941.4	2347.73	2326.64	1847.01	1187.865	3593.6	2023.46	2140.37
1020	346.9347	83	616.35	189.28	691.168	513.796	240.51	503.5669	1658.76	689.904	456.402
1021	347.0745	68	980.494	515.18	1051.2	629.351	751.347	828.9368	2042.83	997.367	588.551
1022	347.1708	115	920.707	403.31	864.743	1492.43	977.969	1042.67	4046.49	1051.76	392.059
1023	348.0935	12	22.8933	26.502	22.72	68.22	58.4743	39.77633	658.417	26.63	13.7392
1024	348.1716	114	135.815	151.01	228.477	449.943	182.435	306.8093	1043.9	306.203	157.523
1025	348.813	64	1643.75	958.31	889.288	1037.53	1100.47	580.734	1746.88	934.049	1075.82
1026	348.8951	81	753.732	530.99	1169.28	907.498	1201.96	701.2344	2262.42	559.085	455.899
1027	349.0227	90	335.248	137.46	124.333	1730.7	1605.74	1149.435	3067.26	1207.34	1166.77
1028	349.105	62	1223.01	502.47	677.79	658.503	1030.16	408.7345	1596.12	635.247	524.239
1029	350.1235	59	1716.76	400.74	2412.32	337.703	404.408	227.9764	1099.04	601.817	292.329
1030	350.1247	1040	1087.77	941.89	961.311	1313.72	1081.12	980.5435	896.24	738.344	803.493
1031	350.9431	63	608.713	327.22	363.354	426.07	613.022	395.1886	2113.36	849.69	662.523
1032	351.217	69	1125.73	834.75	2460.76	944	1717.48	2024.31	2335.48	1363.28	1444.79
1033	352.9399	62	197.58	112.3	156.936	138.448	251.86	157.542	761.033	436.204	272.48
1034	353.049	65	754.172	364.9	660.66	604.512	589.606	299.7395	1224.11	788.689	568.736
1035	353.1995	1043	1331.17	1512.8	1414.87	2066.42	1324.14	1516.167	2816.27	2237.91	1318.32
1036	354.8479	64	3548.45	2302.9	2264.59	3723.11	1878.51	1515.812	3486.95	2943.5	3025.01
1037	355.841	68	1620.97	1238.7	1768.53	1188.97	1212.88	1194.998	3374.72	1540.52	1324.72
1038	356.1714	1039	480.894	554.14	411.523	660.8	652.978	663.5219	1289.42	724.755	547.596

1039	356.1848	121	518.13	365.65	660.119	518.534	416.989	438.1328	1197.19	615.799	415.289
1040	356.845	64	3628.66	2374.9	2379.72	3304.84	1883.26	1269.01	3239.09	3022.52	3324.96
1041	357.838	70	2563.96	1546.5	2320.2	1627.91	1843.42	1545.21	4925.94	2352.23	1849.94
1042	358.7052	71	621.605	436.32	771.773	406.775	412.009	251.6971	1543.71	493.339	408.956
1043	358.841	64	1178.06	997.5	808.972	1248.66	813.712	616.7947	1409.49	1202.42	1127.43
1044	358.9575	87	574.79	513.06	430.804	1199.16	1017.04	997.6436	2521.77	1010.45	395.985
1045	359.043	270	960.166	236.18	880.96	445.664	398.707	514.9191	940.234	781.492	286.046
1046	359.0433	228	434.981	81.339	195.996	236.993	191.888	224.4058	438.793	242.911	164.555
1047	359.0442	248	895.912	131.92	655.97	363.712	251.897	345.4914	593.527	402.408	192.257
1048	359.0443	291	341.532	206.82	343.639	689.828	583.177	585.424	999.208	326.471	146.506
1049	359.0973	1068	1902.32	1706	1753.09	4385.29	4224.19	3456.723	5388.5	2701.44	1659.38
1050	359.0973	1057	984.9	886.43	1013.97	2532.63	2239.08	1771.187	2952.68	1467.53	854.244
1051	359.0974	43	1617.24	1281.5	1145.62	3380.55	2972.3	2410.779	6490.3	1902.22	969.044
1052	359.0976	87	1830.52	1522.7	1318.51	6042.29	4655.43	2442.766	6125.3	2050.55	1277.7
1053	359.0978	244	1001.85	731.37	865.095	2244.92	2011.57	1842.937	4872	1620.29	516.737
1054	359.0978	3	0	243.76	0	1101.77	807.255	0	612.9	0	134.364
1055	359.098	222	1033.04	813.08	850.463	2401.72	3187.64	1843.922	3015.03	1775.47	600.887
1056	359.0985	139	1574.33	998.71	1060.03	3185.33	2412.42	2144.666	6733.57	2104.32	829.138
1057	359.835	67	1157.06	813.96	1067.18	828.454	974.221	787.5289	2338.51	1278.46	1049.72
1058	359.9881	90	1962.77	631.05	1716.81	949.473	669.335	1164.595	2730.9	1692.35	917.353
1059	360.9725	64	1688.31	839.8	1314.28	887.027	1134.55	937.2935	1978.5	1554.7	1086.34
1060	360.9726	83	1506.43	622.32	1384.24	730.105	779.648	773.975	1996.36	1338.59	759.651
1061	361.1652	82	719.512	464.64	939.355	1167.14	1194.82	1260.439	1558.13	747.189	847.21
1062	363.0216	70	7.95	10.58	10.72	65.0475	186.55	45.2115	643.464	354.983	200.075
1063	364.8764	62	6883.32	4022.2	5276.75	6612.95	3065.85	4082.712	14535	7040.84	4822.7
1064	364.8767	75	6668.09	3929.9	5288.1	6250.25	5353.05	3893.687	8118.71	3743.75	4575.89
1065	366.8738	64	3424.25	2553.4	3412.89	4597.5	3696.21	2081.105	4968.68	4057.91	3982.64
1066	367.1889	118	329.495	156	301.975	217.029	540.662	214.9982	694.739	543.307	224.13
1067	367.8699	62	613.661	364.47	492.799	471.313	430.712	312.2348	653.476	339.574	299.702
1068	368.8697	60	389.877	495.24	193.609	360.528	481.44	210.33	1005.1	379.27	634.23
1069	370.8221	62	584.05	381.24	555.765	591.705	334.191	273.2916	911.786	477.092	429.326
1070	370.9573	89	268.934	284.89	374.656	1137.34	670.45	744.1298	2090.66	966.618	562.161
1071	371.136	66	536.175	122.29	174.222	212.364	595.623	160.6511	1927.36	913.357	423.299
1072	372.8176	64	1172.88	723.19	909.295	861.584	829.302	554.2722	778.321	697.075	807.906
1073	373.1114	96	916.689	894.16	713.162	2147.55	1661.52	1374.901	3410.53	1215.5	620.367
1074	373.1141	2	57.2032	56.525	48.384	184.964	122.474	73.7538	415.569	74.2684	25.3823
1075	373.1836	75	683.869	148.85	786.998	915.04	476.392	299.3097	561.702	301.393	194.457
1076	373.8131	71	668.547	423.73	824.467	529.634	794.163	390.3056	1330.86	823.836	651.718
1077	374.2442	79	2297.91	1245.4	1646.51	5836.86	1929.55	3020.242	2242.83	2013.27	1725.91
1078	374.9048	59	2429.25	3001.6	1075.9	2489.37	4297.92	1374.313	6514.87	3585.65	3989.63
1079	374.9057	82	18833	8850.1	19309.9	19656.3	10297.8	14195.33	38448.4	19156.6	9739.57
1080	375.2467	79	711.18	285.95	570.045	1629.54	792.103	847.5436	336.597	542.567	406.161
1081	375.8991	62	405.042	375.83	255.11	486.176	667.368	296.628	948.485	451.845	550.293
1082	375.908	79	1369	735	1166.63	1311.29	1125.2	1062.526	3112.33	1013.09	679.213
1083	376.9026	82	6922.84	3123.1	5795.13	6371.42	4289.17	4920.497	14784.9	8133.89	3309.43
1084	376.903	59	885.36	1340	511.338	797.532	1544.84	556.1429	2498.73	1606.93	1701
1085	377.0847	121	1206.57	923.16	1361.1	848.547	949.295	743.7146	2992.49	1561.76	748.509
1086	377.0849	70	6606.56	4684.9	4704.94	5234.79	5139.54	3567.232	11666.7	6095.58	4238.04
1087	377.1637	114	429.013	223.79	510.939	588.149	327.872	329.5624	953.31	476.407	237.07
1088	377.2708	1194	263.796	240.55	136.76	516.95	563.779	461.7357	1013.82	0	315.531
1089	377.2718	1084	820.145	757.9	701.58	1438.85	1657.97	1233.501	1848.72	907.912	760.654
1090	377.2718	1050	1877.24	2076.6	2182.79	4710.86	3892.84	1365.819	2390.67	2524.44	2072.88
1091	377.2718	138	2604.48	2158.4	2985.17	4380.92	3765.68	3379.058	9375.37	3792.09	1813.78
1092	377.2719	1120	337.804	301.77	292.998	374.842	1045.38	399.2229	525.78	356.732	337.077
1093	377.272	1136	994.342	875.68	815.422	1425.03	750.632	1281.166	1811.87	1115.18	867.483
1094	377.2721	1158	349.791	238.95	171.079	732.211	1266.65	2043.657	488.644	435.63	258.105
1095	377.2722	1176	833.179	667.41	532.904	2082.11	1355.49	1166.401	1365.64	791.51	801.27
1096	377.2724	1042	1184.36	1049.1	1069.58	1484.93	1714.95	1399.138	2175.6	1320.29	1177.84
1097	378.088	70	784.823	680.3	753.881	706.194	874.942	482.2986	1349.22	962.408	553.262
1098	378.9165	90	2371.87	1715.5	1277.6	2594.89	1787.91	2475.83	4843.4	2216.62	1770.67
1099	379.0822	70	2114.37	1489.1	1513.29	1652.71	1741.78	1365.908	4577.76	2329.56	1547.7
1100	379.1542	86	601.064	163.58	261.319	350.437	1112.89	800.6468	999.216	447.11	413.706
1101	379.9611	96	1171.38	474.77	920.673	638.171	608.22	764.947	1665.21	807.005	655.04
1102	380.8505	62	830.677	558.51	539.991	615.273	708.972	380.2263	1103.11	659.35	727.873
1103	380.8509	74	2001.75	1111.1	1532.82	1727.93	1998.69	1021.919	3543.82	1695.28	1430.19

1104	381.2297	1053	647.672	702.61	609.404	798.46	770.86	769.2363	807.46	659.124	954.756
1105	382.7194	70	2666.81	2404.4	2421.22	2502.55	2551.84	1669.308	7039.44	3233.07	2340.12
1106	382.8467	60	478.56	541.76	349.424	452.189	526.083	259.5556	882.167	612.827	587.45
1107	382.8477	75	1168	951.88	1223.6	1272.65	1068.69	706.3701	2001.52	1321.26	888.829
1108	383.707	86	728.099	307.48	617.232	465.193	344.071	374.5719	1658.88	1203.46	459.262
1109	384.029	96	435.084	159.23	256.363	154.445	190.161	257.8412	1078.15	623.36	243.51
1110	384.7173	70	6019.2	4895.4	5194.01	4757.31	5242.29	3829.533	17099.3	7048.8	4940.26
1111	384.934	85	130204	66313	109297	98752.3	78291.4	102667.9	372359	171809	73337.7
1112	385.9287	62	428.46	309.47	286.408	330.145	518.42	264.306	816.333	492.362	493.87
1113	385.9373	86	10013.4	5484.8	8847.27	7272.99	6843.63	7981.958	28777.1	12962.8	5401.74
1114	386.7141	70	5880.94	4637.2	4892.85	4498.96	5192.93	3655.046	16498.4	6689.23	5263.95
1115	386.9382	86	4331.55	2283.5	3223.63	3481.33	2807.03	3058.978	12703.8	5804.66	2534.3
1116	387.1133	104	9169.13	3558.7	7785.41	1679.95	6235.78	4576.473	11819.5	5621.46	3181.98
1117	387.1141	60	1610.07	1359.4	1607.98	1592.5	2212.26	1210.953	3015.25	1211.38	1271.05
1118	387.1656	63	1596.01	355.89	1388.93	752.129	1289.76	768.2513	1472.92	889.819	657.017
1119	387.1961	67	1577.1	310.87	1243.42	707.367	1663.12	714.3429	992.483	683.686	520.744
1120	387.9414	92	342.363	140.18	335.729	282.278	274.031	270.6146	1310.3	530.513	243.518
1121	388.1161	97	1539.47	819.75	2177.22	1012.23	1055.8	1010.012	2171.55	1461.55	908.167
1122	388.1532	84	1741.2	650.46	1824.29	1346.81	1075.25	1481.239	2458.11	791.216	1026.72
1123	388.711	70	3175.56	2725.6	2678.46	2529.67	3036.38	1992.828	8883.92	3591.75	2989.69
1124	388.9194	82	1253.42	455.06	936.219	1108.21	884.846	776.3482	2577.93	1300.62	687.212
1125	389.9991	90	5268.28	1564.8	4675.91	2105.88	1980.29	2431.176	4882.74	3356.94	2056.24
1126	390.2384	91	1081.94	696.13	459.988	1929.04	920.386	997.8472	1268.71	646.361	713.718
1127	390.7077	69	1045.64	886.23	778.56	946.552	689.189	691.4617	2216.88	1160.2	897.789
1128	390.8786	80	4458.2	1208.8	4698.85	3774.28	2206.98	2778.566	8897.72	3085.83	1613.06
1129	390.8803	60	604.02	546.62	370.673	393.565	763.925	355.025	1503.59	861.21	644.431
1130	391.8959	62	171.513	226.08	202.848	435.911	567.53	218.6297	518.446	554.539	303.107
1131	392.7474	66	766.973	554.09	567.488	537.93	461.246	380.871	1581	703.42	630.562
1132	392.8768	80	2039.46	925.56	2108.63	1796.47	1142.99	1322.118	4096.53	1973.62	967.672
1133	393.1697	90	541.733	233.33	367.413	499.811	1569.52	1021.447	1301.67	833.781	662.205
1134	393.2264	77	7150.31	1261.3	2034.55	4516.21	1865.36	1057.479	2298.99	1714.78	920.059
1135	394.2304	77	1934.47	184.48	368.75	1072.91	586.315	265.6863	201.317	170.268	54.6381
1136	394.7462	67	1449.52	922.13	1012.13	1012.33	800.608	673.8046	1970.1	960.419	1043.01
1137	395.1494	63	1280.7	572.29	1611.92	1477.98	854.061	396.0041	1095.83	568.444	546.245
1138	395.243	77	6125.37	1986.6	2800.92	4615.6	3671.7	965.3236	875.093	866.466	646.477
1139	395.2432	112	570.968	519.69	670.591	498.553	914.269	241.9035	1387	740.481	617.339
1140	395.9552	83	3468.18	1636.6	3835.09	2203.07	2174.32	2676.373	8436.28	4478.3	1670.94
1141	396.246	76	1922.3	374.93	545.889	1283.84	739.431	164.91	244.694	217.183	84.105
1142	396.7428	66	1111.28	605.52	946.785	947.934	592.779	500.7944	1850.7	824.908	643.849
1143	396.9579	82	322.996	97.756	284.726	178.202	408.37	323.9147	1147.17	477.07	312.467
1144	397.2255	1045	695.162	916.98	657.186	1152.95	985.13	784.6054	1659.29	1166.56	1067.05
1145	398.6936	70	1191.69	1185.1	1315.27	1032.02	1222.68	793.7952	3769.06	1580.72	1159.78
1146	398.8741	63	349.733	133.6	252.768	166.237	307.073	190.53	861.571	511.55	426.833
1147	398.9498	85	7710.32	3920.1	6222.09	5789.12	5145.09	6368.865	20930.4	9656.83	4766.26
1148	399.9484	87	729.266	551.74	607.015	664.627	588.529	576.2709	1449.74	1227.71	705.136
1149	400.6913	71	3233.1	2553.9	2912.29	2187.34	2761.1	1746.283	8159.63	3397.5	2631.49
1150	400.8733	63	436.303	383.73	351.462	411.232	594.898	346.7714	867.56	868.743	605.886
1151	400.9077	83	16014.1	7108.9	16713.3	13066.1	9927.58	11577.85	39655.9	16340.1	8703.98
1152	401.9185	83	1593.83	1181.6	1004.19	1520.97	1467.42	1396.436	4711.56	2082.45	1089.88
1153	402.0101	90	4808.26	1334	4200.97	1535.31	1678.27	2211.615	5214.99	2552.42	1742.58
1154	402.6881	70	3139.54	2779.1	3071.72	2141.26	2911.92	1816.483	7696.22	3102.2	2953.01
1155	402.7771	64	968.262	667.93	676.928	911.834	514.918	494.6563	827.885	427.077	838.228
1156	402.9075	84	1795.72	899.54	1966.28	1536.03	1740.68	1346.617	3068.58	1772.63	1004.89
1157	402.999	93	6396.37	277.3	648.596	2602.15	3169.52	465.8723	3725.67	2232.48	1188.98
1158	403.1879	70	1312.04	270.23	410.709	676.579	3040.74	241.9093	1166.04	691.425	646.027
1159	404.1048	125	446.46	393.11	519.684	494.376	450.782	334.6359	1792.1	818.248	345.099
1160	404.6848	71	1903.28	1604.1	1752.5	1318.08	1811.52	1132.319	5473.71	2186.39	1797.54
1161	404.7736	64	1942.75	861.94	1370.48	1423.94	1223.73	638.556	2366.77	1282.67	1247.85
1162	406.0095	97	926.361	369.55	765.367	298.247	311.053	327.436	782.85	709.469	471.36
1163	406.6832	71	717.409	621.85	671.254	481.574	741.178	386.251	2000.42	818.338	680.397
1164	406.7711	65	1296	800.79	875.719	916.426	955.153	486.459	1887.65	1028.79	915.992
1165	407.1856	95	574.24	227.78	498.536	471.516	1237.6	984.9902	967.233	677.276	480.739
1166	408.9028	63	540.687	219.46	264.257	277.687	388.07	256.48	946.91	638.743	476.806
1167	409.176	67	189.833	148.68	365.173	182.21	278.723	238.07	529.598	221.233	247.799
1168	409.3094	83	950.628	692.97	378.319	1416.14	1198.09	1010.055	1037.06	612.212	670.559

1169	409.3098	114	964.497	787.25	481.5	1360.26	1189.37	939.1042	882.5	440.802	712.388
1170	409.3103	152	1101.25	610.66	340.156	1167.67	1003.49	864.7809	702.886	411.32	615.021
1171	410.2209	77	203.698	915.04	123.28	1090.57	1353.3	46.84333	1051.62	599.28	797.46
1172	410.7187	67	819.497	360.34	571.738	451.863	295.921	311.857	997.57	396.467	510.785
1173	410.9	62	358.625	169.47	245.648	209.062	283.222	200.2	828.192	435.814	366.45
1174	412.8059	64	1963.23	1278	1348.29	1060.88	1264.59	790.2456	1738.4	1462.73	1580.06
1175	413.8007	68	716.659	358.78	739.837	463.7	500.804	428.2804	1711.85	704.582	434.022
1176	413.9606	93	1747.17	740.49	977.128	1015.19	852.947	880.1441	1832.1	957.785	903.39
1177	414.8033	64	2699.91	1799.3	1690.53	2116.9	2281.16	1040.998	2390.18	1916.24	2475.85
1178	414.9241	83	899.398	572.14	1052.02	948.917	847.054	996.8121	2848.06	1440.15	716.599
1179	415.7957	67	1389.42	595.34	996.652	884.453	699.705	734.0066	2606.06	1182.03	806.04
1180	416.6641	70	862.538	776.08	1020.51	506.448	622.248	445.0733	1914.38	861.833	747.06
1181	416.8002	63	1373.13	963.59	895.964	941.396	806.769	568.164	1944.89	764.342	1056.89
1182	416.8811	82	1453.61	1070.9	2328.94	1575.04	1737.84	1322.118	4404.59	1412.17	884.276
1183	417.0096	90	637.581	143.54	158.673	1663.9	1498.26	1261.385	3223.65	1309.57	981.749
1184	417.7942	72	792.52	471.85	720.374	599.686	688.275	514.027	1923.8	797.875	554.864
1185	418.1124	72	472.056	302.9	649.856	331.773	339.293	392.8458	837.848	521.604	423.661
1186	418.6616	69	1024.34	894.16	1248.93	630.368	845.582	495.0388	2553.07	1071.76	900.446
1187	418.8793	82	156.104	249.63	210.834	250.33	329.728	146.415	744.705	176.85	121.275
1188	418.9325	62	527.345	212.75	254.993	420.171	458.222	319.1198	1424.04	810.387	584.76
1189	420.2491	79	2487.68	1669.8	2910.66	8248.96	2127.69	3592.428	5835.7	2482.04	1830.7
1190	420.6576	72	551.54	529.34	760.147	434.056	477.56	306.3856	1525.91	663.142	756.075
1191	420.7473	66	1086.65	836.38	1177.48	600.243	588.027	551.0059	2511	1035.41	1074.84
1192	421.1534	77	2430.64	3218.6	672.175	3342.52	43840.8	478.555	9385.48	5038.73	7237.04
1193	421.2254	1045	264.586	385.12	284.647	343.348	447.068	286.74	920.496	653.539	727.318
1194	421.252	79	906.026	3142.9	850.461	2040.26	43725.8	1161.509	10021.6	5270.03	7004.11
1195	422.1578	77	525.517	563.3	72.81	591.822	11592.2	201.5357	1941.75	1074.19	1478.96
1196	422.8348	64	3404.11	1653.8	2037.71	2641.96	1564.88	1526.882	3035.32	2651.46	3027.66
1197	423.1517	77	799.314	1194.4	217.647	1009.38	13587.2	241.6498	2925.46	1821.48	2257.93
1198	424.8324	63	3502.32	1837.7	2163.24	2829.54	1561.8	1526.382	3367.09	2185.9	2501.42
1199	426.0202	891	160.461	163.51	133.95	157.337	161.456	154.3597	131.326	185.9	140.519
1200	426.0205	1002	249.057	339.78	361.006	261.348	268.443	307.1476	278.563	396.322	364.931
1201	426.0209	959	70.4	445.48	471.224	317.876	405.376	492.3514	262.542	494.155	495.01
1202	426.0212	918	2511.43	1952.6	1926.04	1612.22	2822.2	3004.799	2649.81	545.498	1755.28
1203	426.828	63	1135.78	1045.1	796.958	1108.12	1463.19	655.8164	1834.78	1104.96	1197.69
1204	426.9387	89	447.518	306.34	227.401	701.22	512.09	638.616	894.963	796.979	203.738
1205	427.976	88	1917.06	610.77	1467.53	978.272	827.115	996.5732	1746.32	1067.25	760.642
1206	428.7792	67	1112.82	400.24	738.784	675.445	376.58	432.5021	868.421	426.767	559.687
1207	428.9607	86	946.66	437.07	982.799	573.323	526.021	911.825	2485.04	1247.81	584.926
1208	429.8074	72	187.731	266.18	298.749	251.114	361.76	232.1409	838.688	506.495	360.391
1209	430.7772	67	1292.95	596.88	1178.13	1085.19	658.115	585.3612	1555.15	930.149	928.746
1210	431.1833	79	24124.6	4852	3567.27	14332.8	11005.4	5451.5	15114.5	10112.1	5342.77
1211	431.2201	74	630.667	3995.3	3580.79	13367.7	8441.41	4915.03	15433.7	10008.7	1037.42
1212	432.1858	79	5876.83	1290.3	763.8	3513.61	3268.41	1388.985	3974.71	2142.87	1354.43
1213	432.2221	74	3640	1028.3	635.177	1854.56	1987.21	969.7114	3283.68	2008.57	991.527
1214	432.7688	66	888.633	657.47	797.767	826.392	1167.59	528.6151	1402.64	785.984	725.225
1215	432.8633	63	6318	3019	4648.61	6159.28	4388.36	3439.299	4985.45	6434.31	5198.71
1216	432.8639	76	6323.4	3975.3	4606.02	6236.69	5749.29	3158.624	7275.48	3833.37	5087.73
1217	433.1895	79	1157.31	1233.2	222.44	835.574	3221.4	317.0933	627.534	480.48	410.316
1218	433.2172	75	1004.08	1393.8	245.518	870.716	2832.22	289.3516	1060.35	516.795	454.142
1219	434.7625	72	401.497	408.59	336.885	355.05	467.634	366.8972	1037.61	526.035	503.617
1220	434.8603	76	3788.33	2571.5	2818.11	3173.11	4289.83	2117.787	7238.52	3961.2	3253.07
1221	434.8607	62	3080.51	1566.4	1990.35	2799.99	3487.16	1848.853	5202.31	2091.44	3307.77
1222	435.1277	88	1704.9	1416.6	1005.1	4186.35	3048.61	2020.402	5321.71	1951.4	1079.3
1223	435.1287	1053	670.044	595.47	591.893	1729.23	1563.63	1221.342	2067.13	985.051	567.05
1224	435.1301	2	434.181	323.37	324.12	1389.35	982.855	647.5896	1981.56	514.667	199.476
1225	436.8628	62	439.56	412.63	164.783	366.935	479.22	160.146	691.25	547.214	584.26
1226	438.8073	65	1627.16	884.5	1318.92	934.099	1245.29	753.4468	2698.99	1565.91	1107.58
1227	438.9459	91	465.937	261.9	218.358	970.103	579.219	763.4453	1796.35	483.092	497.566
1228	439.7165	71	280.63	264.8	219.056	352.98	236.01	228.9148	1051.42	502.12	269.767
1229	440.6781	70	4264.09	3856.8	3398.13	2835.65	3431.05	2564.298	9974.86	4902.87	3983.95
1230	440.8063	64	1705.22	879.22	1310.91	1323.68	1230.95	728.2479	2380.39	1486.89	835.987
1231	442.6757	70	11366.4	9740.2	8908.12	7709.12	9369.82	7057.096	24002	12432.7	10347.5
1232	442.8925	82	21854	9318.4	17542.5	20148.1	12204	14348.46	39889.4	21623.5	9876.79
1233	442.8934	59	2064.65	3062.5	868.478	1643.96	3276.42	1132.74	5847.32	3531.2	3744.56

1234	443.245	499	185.952	180.65	222.343	286.543	273.343	267.9511	463.526	186.343	123.86
1235	443.2465	471	527.957	375.58	1864.93	1778.7	983.101	726.6622	1398.67	1076.87	661.406
1236	443.8948	83	1977.75	968.74	1536.59	1860.84	1603.96	1353.444	4259.74	2030.04	1443.59
1237	444.6726	70	13184.9	11116	10353.8	8940.06	10585.8	8542.384	27330.4	14638	11447.2
1238	444.8899	59	813.106	1028.6	234.918	922.614	1363.93	364.4241	1659.48	1229.16	1187.86
1239	444.8902	82	8687.2	3250.8	5972.65	7381.77	4151.45	4434.406	16096.1	7684.75	3560.06
1240	445.8902	80	530.691	272.49	442.997	368.507	796.767	268.7029	991.709	740.959	316.183
1241	446.6695	70	8144.29	7254.3	6479.58	5469.48	6082.46	4831.802	18143	9230.01	6964.66
1242	446.9035	93	1595.41	1237.1	1028.9	1845.94	1750.59	1506.985	3562.95	1670.43	1343.02
1243	447.1326	75	1143.2	1020.3	912.912	1333.01	2695.21	1271.418	2145.42	1128.9	1325.86
1244	447.1333	98	706.694	642.39	594.027	597.934	694.584	872.7455	1684.36	1194.88	858.324
1245	447.2138	70	443.306	844.03	316.663	655.485	1137.62	249.1541	1463.09	725.244	601.104
1246	447.9502	92	702.049	442.78	808.839	601.649	392.907	440.4675	1885.42	841.492	464.516
1247	448.6666	70	2796.45	2799.1	2368.28	2134.82	2554.97	2012.122	7119.6	3948.02	3013.47
1248	448.8374	76	2958.56	1434.8	2486.16	2562.32	2207.52	1172.178	3009.34	2127.16	1510.9
1249	448.8377	62	2285.88	1435.6	2072.49	1934.42	879.495	1356.839	4302.38	2235	1038.19
1250	449.1484	79	1827.07	5097.1	1363.14	3514.7	1631.76	1619.976	1430.8	2849.35	3281.98
1251	449.1717	76	670.905	4633.7	856.841	1886.35	6855.65	483.1783	3845.38	1688.68	2363.86
1252	450.1768	75	363.813	1352.7	251.429	837.45	1906.52	216.7319	1318.96	532.795	797.492
1253	450.6628	71	1283	943.72	911.54	1062.18	979.444	660.0858	1808.71	1197.16	1038.57
1254	450.7071	67	1168.2	972.13	905.639	933.655	798.045	685.8752	2395.1	1417.96	1138.82
1255	450.8348	75	1880.3	959.82	1733.1	1825.5	1827.54	1076.614	2728	1930.77	1360.18
1256	450.8348	62	717.813	596.48	425.606	523.604	599.452	266.705	1337.06	822.052	1460.55
1257	451.17	76	395.453	1647	301.763	947.065	2284.98	383.2664	2400.99	689.029	998.615
1258	451.476	84	675.644	436.66	461.337	336.662	519.56	417.7695	1970.3	899.113	483.38
1259	452.7041	67	2541.83	1684.6	1582.86	1724.68	1326.87	866.073	3142.39	1653.17	2014.65
1260	452.8367	78	485.067	234.94	537.58	415.115	479.264	302.4654	1028.71	587	427.664
1261	452.9218	85	145632	72372	116861	99667	83133	107453.2	392289	194732	81301.4
1262	453.9248	86	12416.9	6847.8	10673.6	9079.31	6667.56	9815.958	35322.2	16778.7	6628.66
1263	454.7008	67	2620.66	1591	1626.65	1597.15	1223.94	1004.231	3341.74	1720.49	1638.19
1264	454.9257	86	4843.27	2684.9	3937.09	3495.61	3574.46	3430.486	14470	7052.15	3042.59
1265	455.1008	100	1912.44	975.87	1868.93	1323.61	1043.48	1048.592	3627.71	2036.8	753.754
1266	456.6523	71	2760.86	2378.3	2663.49	1604.92	2191.3	1554.098	5537.48	3012.03	2812.39
1267	456.698	67	1467.97	2616.5	2460.16	1698.97	2334.01	1563.056	1623.05	2654.38	3088.41
1268	456.9081	82	1652.95	814.24	1289.15	1324.86	768.696	1050.355	3076.95	1750.91	746.956
1269	457.9866	89	4392.74	1339.5	3696.22	1738.05	1429.72	1754.177	5752.42	2985.47	1662.72
1270	458.6493	70	6547.44	6369.4	6701.46	4047.64	5714.23	3785.385	14343.9	7380.3	6397.38
1271	458.8661	81	7784.14	2985.9	8036.77	6213.16	3895.29	3916.269	14296.6	5919.13	3184.84
1272	458.8687	59	698.19	758.67	459.038	453.102	925.12	436.17	2160.19	1346.49	1112.19
1273	459.1033	2	1.18829	1.1862	0	29.6788	2.6685	4.135	227.012	4.178	0
1274	459.1081	12	18.6041	9.5583	34.4181	80.3904	40.8463	40.1736	605.729	30.4679	10.384
1275	459.2005	80	5191.13	2094.8	3940.16	9163.46	4539.66	3925.463	9158.91	3090.95	1871.08
1276	460.2043	80	1634.94	614.38	1239.48	3067.49	1392.43	1187.352	2416.75	963.415	585.15
1277	460.6468	71	7950.94	7300.3	7927.93	4710.87	6632.4	4299.894	15601.9	8838.75	7870.35
1278	460.7356	65	1165.15	687.31	744.206	940.684	897.398	531.1856	6435.6	920.95	1052.93
1279	460.8632	80	3358.39	1203.9	2958.37	2925.97	1810.28	2118.076	5247.3	2592.56	1357.4
1280	462.6432	71	5534.1	4933.2	5458.2	2908.78	4817.04	3015.425	11183.7	5471.56	5214.01
1281	462.7334	64	2773.94	1326	1553.48	1629.5	1582.31	895.1477	2721.15	1654.49	1803.63
1282	463.1246	87	1130.67	733.51	689.71	1728.38	1746.14	1371.014	2686.03	1009.95	640.121
1283	463.9429	84	2801.37	1178.3	2795.76	1609.12	1816.09	2131.362	7355.73	3820.38	1779.36
1284	464.6406	70	2325.26	2205.8	2195.02	1626.33	1971.27	1229.216	4520.17	2518.39	2336.83
1285	464.73	64	2057.4	1165.3	1251.6	1420.41	1371.93	801.7088	1509.18	1287.65	1611.75
1286	465.1677	73	400.349	517.64	209.674	316.639	1262.13	185.7538	1022.76	614.941	555.28
1287	465.3032	187	1342.94	413.29	1614.88	914.444	1100.38	1084.556	1935.97	1115.98	449.952
1288	466.6376	71	667.565	636.92	593.952	414.388	557.79	363.7122	1690.94	887.743	692.912
1289	466.7258	65	970.661	701.82	746.76	724.828	604.638	430.7624	1243.54	649.928	894.949
1290	466.9371	85	10728.8	5192.6	7888.04	7444.26	5930.51	8292.178	29377.2	12954.5	5660.16
1291	467.9411	86	1502.76	777.11	1142.55	979.996	968.805	1273.58	3526.15	2079.92	742.528
1292	468.6788	67	1386.97	582.39	1152.75	864.495	405.404	495.2072	884.331	743.771	780.867
1293	468.8957	84	28889.2	14953	30538.7	22642.9	18341.4	22046.2	73541.5	30372.2	15370.6
1294	468.9385	88	15658.5	10135	17228.9	13994.5	10470.7	7829.788	1169.82	18931.5	10866
1295	469.3624	71	37.32	42.377	14.8029	156.279	926.139	90.72171	212.571	209.176	196.819
1296	469.8997	84	3692.95	1619.1	3350.36	1935.25	2206.54	2683.797	9710.51	3468.38	1984.75
1297	469.9975	90	3666.68	1148.5	3460.04	1306.58	1424.19	1712.065	5516.57	2368.64	1630.68
1298	470.6749	67	1350.11	678.91	1319.72	796.594	460.65	537.5778	2075.23	1050.63	746.397

1299	470.7644	64	1948.19	1285.2	1180.8	1307.46	1329.62	719.004	1848.05	1326.09	1347.02
1300	470.8956	84	3798.13	1618.1	3176.79	2844.1	2526.26	2434.427	8692.16	3706.6	2188.97
1301	470.9857	92	5568.58	240.86	651.314	1691.35	2920.12	472.5826	3570.97	1741.16	1366.15
1302	471.8987	83	548.371	182.98	482.453	498.213	439.484	391.0971	1031.8	628.273	296.154
1303	472.6275	72	817.195	713.73	811.154	481.126	588.735	350.438	1440.17	833.661	819.991
1304	472.6708	67	873.037	378.18	701.844	408.308	453.34	337.7573	1046.53	522.08	527.472
1305	472.7615	64	3115.38	1733.4	1979.87	2279.73	1685.92	1277.723	2085.86	1359.31	2440.1
1306	473.2816	121	4202.37	1792.4	4549.35	3642.26	6189.1	2004.3	10634.6	3403.44	3802.54
1307	473.7552	68	752.93	457.35	817.99	725.791	678.394	460.9216	1573.44	906.641	670.371
1308	474.2848	122	1343.16	553.74	1371.49	1269.62	2086.63	710.6469	3312.42	1158.33	1180.3
1309	474.6234	71	1877.29	1899.7	2128.34	1073.46	1694.77	894.9825	3311.34	1966.59	1896.7
1310	474.7594	64	1874.22	1320.1	1765.22	1332.75	1457.17	1334.934	1240.51	919.452	1815.22
1311	474.841	79	2267.72	1206.2	1556.63	1284.66	1317.63	1388.615	2814.93	1865.86	1640.67
1312	475.7522	71	790.443	525.06	825.799	551.154	459.088	420.6334	1279.17	843.545	515.833
1313	476.6202	71	2252.25	2168.7	2464.46	1079.6	2030.94	1119.766	4507.85	2372.52	2540.44
1314	476.71	66	840.599	470.34	700.381	481.558	342.204	357.4892	1094.02	538.025	589.991
1315	476.7535	64	1480.21	826.8	1387.42	1099.84	745.861	663.6074	2287.43	978.523	961.222
1316	476.8403	79	645.246	263.34	877.197	746.753	370.329	352.869	1183.16	479.047	335.692
1317	476.8893	62	222.453	172.9	186.943	214.17	323.505	184.005	911.786	470.888	450.473
1318	477.8539	71	87.0333	153.22	132.239	156.976	210.405	165.5104	1291.1	226.452	303.597
1319	478.6168	71	1780.82	1639.9	1829.62	1093.26	1425.14	825.861	3307.04	1771.31	1737.2
1320	478.706	67	1304.72	729.82	1293.01	828.59	1009.2	592.5803	1817.2	951.669	975.256
1321	479.1732	74	30.5363	4748.5	22.8367	90.5156	173.345	16.95556	189.16	130.889	131.879
1322	479.9113	84	554.419	326.49	1090.61	493.924	628.099	570.8739	1487.29	924.154	433.782
1323	480.1754	74	0	1346.6	14.8088	42.8	13.1613	26.43575	151.905	47.5155	90.3465
1324	480.3084	209	2487.8	1132.8	4192.66	1088.58	1533.24	1886.995	3746.19	2538.54	986.772
1325	480.616	71	645.369	663.68	806.37	354.594	546.977	371.6156	1517.23	711.143	587.05
1326	480.7035	67	1315.94	513.17	1030.71	792.796	580.899	504.7584	1727.14	714.394	902.323
1327	480.7934	64	2612.39	1779.5	1674.46	1688.07	2198.91	1183.239	1820.03	2049.25	1956.51
1328	481.1692	74	144.878	1563	77.9832	174.633	430.295	88.76092	326.604	175.652	174.119
1329	481.948	91	2152.22	1098.6	953.207	1050.18	911.908	1029.455	2502.32	1219.87	1252.33
1330	482.7907	64	3189.85	1994	2282.14	1893.49	1726.76	1787.383	2734.47	2578.69	3058.17
1331	482.911	83	2722.81	1152.4	2185.03	1869.63	1622.98	1846.006	5877.52	1922.88	1088.83
1332	484.7882	63	1703.49	1292.5	1001.45	1328.85	1057.99	941.493	1461.36	1318.32	1601.65
1333	484.8692	83	4136.19	2069.9	5497.22	3379.42	2901.5	3285.613	10007.6	3870.52	2063.93
1334	484.9976	90	745.85	132.59	89.9935	1642.71	1451.15	1184.904	2600.6	1177.58	1293.62
1335	485.2807	148	1544.05	1464.7	1763.38	2360.47	2128.91	624.9525	1694.75	537.976	337.237
1336	485.282	112	90844	67309	115330	168223	124440	55968.29	120442	46394	19854.1
1337	485.7698	71	306.425	340.38	304.717	392.544	396.618	318.9041	1085.63	585.184	325.846
1338	485.8635	82	341.301	317.73	647.29	512.601	483.708	380.964	1178.01	592.423	287.244
1339	485.9713	82	791.055	172.79	977.612	441.337	355.152	390.4359	731.96	330.376	276.784
1340	486.1238	7	12.4952	7.3406	19.7238	86.79	24.9221	25.45035	873.499	15.1241	5.311
1341	486.124	38	55.0275	10.707	22.1639	191.082	53.7684	59.25265	1010.03	43.117	5.13865
1342	486.2852	112	33163	21995	37710.7	49220.4	39984.3	18680.38	35694.4	14517	6037.75
1343	486.738	65	1242.42	548.79	866.276	797.551	546.449	389.8004	1167.69	747.285	694.461
1344	486.783	63	598.468	444.86	327.794	337.885	426.859	316.8743	793.793	414.878	586.357
1345	486.8676	82	730	594.23	919.124	500.645	818.216	390.7631	2073.88	975.619	575.949
1346	486.9175	62	254.129	210.42	238.327	319.636	337.876	202.6804	816.934	622.261	398.705
1347	487.2875	112	6282.86	4118.1	6822.42	9498.39	7587.96	3466.781	6691.24	2781.73	1204.57
1348	487.7642	70	331.76	376.36	309.372	333.562	605.758	323.057	1287.56	478.272	449.504
1349	488.2909	113	1032.7	654.32	1238.49	1373.59	1292.77	588.9766	1170.9	583.327	214.559
1350	488.7351	66	1808.17	999.2	1471.87	1180.22	1130.41	713.976	2016.47	1383.13	1160.66
1351	489.2772	126	2175.57	274.7	639.829	1136.68	933.762	418.2297	2586.78	763.097	551.51
1352	489.7616	71	194.18	239.7	107.813	229.333	344.904	199.8167	1276.43	358.405	260.658
1353	490.7291	67	1350.58	1029.5	1182.75	1158.98	1085.36	749.6669	2477.74	1551.43	1100.97
1354	490.8217	63	2972.08	2728.9	2289.89	2999.7	1936.55	1655.241	2864.13	2722.8	3233.59
1355	492.722	68	873.613	531.74	817.55	673.069	800.775	522.2165	2408.09	936.681	779.714
1356	492.82	63	3711.67	1775.8	1814.46	3489.9	1755.49	1618.647	2855.28	2963	3485.18
1357	494.7191	70	512.074	472.61	628.164	575.49	685.314	428.526	1829.52	715.815	484.299
1358	494.8162	62	1328.26	704.04	813.993	939.873	716.772	589.0288	1276.21	1072.16	1092.39
1359	494.9314	86	704.713	579.11	457.359	1048.86	829.213	1110.402	3216.9	1385.45	441.882
1360	495.6792	69	410.283	302.1	357.002	387.03	343.578	313.8538	977.55	514.751	464.131
1361	495.963	90	2208.14	774.8	1748.35	831.956	644.316	1142.21	3183.12	1886.81	918.509
1362	496.7169	71	630.388	296.2	601.581	340.848	380.109	321.8628	1405.63	460.116	355.182
1363	496.7667	66	1353.09	827.06	1110.48	1098.63	1131.44	709.9433	1861.95	1370.12	1148.16

1364	496.9436	88	1575.27	362.36	1026.24	527.415	434.259	646.4672	1100.28	993.358	583.946
1365	497.6755	71	510.314	497.19	458.538	448.547	515.46	370.8708	1469.33	619.641	596.388
1366	498.6374	70	3466.29	3513.9	2653.76	2370.3	2869.82	2182.431	8063.04	3976.95	3587.19
1367	498.7646	63	1919.38	1144.4	1541.53	1140.57	788.243	853.5967	1404.22	1020.93	1413.26
1368	499.672	70	470.82	317.76	395.107	386.28	404.4	307.4379	1300	528.478	461.128
1369	500.6344	70	10415.9	9609.8	8299.02	6814.76	8407.99	6958.165	22170	12479.8	10690.1
1370	500.7627	63	2791.04	1896.6	1979.71	1716.07	2258.55	1652.217	1015.57	3121.83	694.851
1371	500.8508	77	6505.26	3805.8	4582.13	5650.67	5877.34	3550.592	11649.5	3540.42	5172.02
1372	500.8515	62	6301.2	3662.1	4654.76	5981.82	2511.68	3606.454	11931.5	6341.82	4502.91
1373	501.6727	72	243.39	154.95	227.756	218.667	237.453	194.4882	810.579	349.182	252.679
1374	502.6314	70	13883.9	12578	10844.4	8641.54	10506.7	8958.216	28399	17321	13869.6
1375	502.8478	61	4312.91	2894.4	2823.73	4282.15	1796.11	2088.829	4007.88	3624.66	2164.73
1376	502.8489	77	4370.86	2898.3	3026.58	4407.41	4255.83	2351.873	7138.17	2467.86	1393.69
1377	504.6282	70	10315.1	9417.4	7999.94	6663.93	7974.32	6515.473	21487.1	12858.5	9999.36
1378	504.8419	79	387.685	161.23	329.4	404.68	463.275	242.852	680.618	306.475	139.388
1379	504.847	62	662.879	458.66	428.467	729.349	501.988	527.1143	928.596	539.261	630.584
1380	504.934	96	515.637	286.15	645.001	553.179	563.793	422.5536	834.476	717.631	215.149
1381	506.6255	70	4933.91	4702.8	3431.21	2978.2	3992.25	2982.024	10449.9	5836.64	4724.84
1382	506.7957	64	1531.87	1096.9	1686.03	1647.49	784.3	956.2978	2014.99	1875.41	1097.56
1383	508.6225	70	1367.33	1664.3	1093.06	1050.13	1418.52	1027.573	4071.25	1845.23	1651.96
1384	508.6657	67	699.077	613.46	540.783	455.264	489.924	266.484	1295.36	592.77	539.349
1385	508.7933	63	2158.33	863.69	1429.49	1442.45	757.953	851.5303	3506.43	1397.29	1577.46
1386	510.6185	71	556.32	453.31	317.291	234.56	432.069	278.141	1958.78	616.398	438.27
1387	510.6627	67	1513.46	1057	1016.83	935.162	835.159	528.5515	1881.01	1118.13	1085.42
1388	510.7944	78	530.634	172.66	538.909	568.904	571.521	233.261	959.805	325.816	215.445
1389	510.8802	83	21408	9303.4	15368.8	15333.2	12398.9	14319.29	41728.9	21441.1	10537.5
1390	511.8838	83	2302.04	1027.3	1932.22	1992.76	1202.82	1413.935	5437.52	2546.76	1117.36
1391	512.6591	67	1909.46	867.43	1142.07	1039.77	763.565	688.4271	1920.74	1176.18	1367.37
1392	512.8781	83	8816.87	3015.8	5696.42	6670.43	4266.51	4888.951	15917	7635.58	3659.43
1393	512.8781	60	545.02	792.45	131.65	614.728	838.591	349.74	1614.79	1112.29	1104.51
1394	513.6511	71	303.24	325.79	350.393	233.333	286.536	190.4919	994.257	426.924	333.802
1395	513.8796	87	727.064	322.46	404.014	493.739	859.576	355.4815	2306.85	860.682	489.796
1396	514.6117	71	2714	2641.1	2221.69	1817.63	2305.1	1242.368	5025.41	3073.46	2880.34
1397	514.6572	67	1181.81	486.2	939.223	824.45	479.65	467.5251	1248.63	968.248	646.571
1398	514.891	90	1569.76	1352.2	1187.99	1739.32	1826.13	1503.102	3575.53	1899.4	1247.31
1399	515.6498	71	162.26	190.7	250.666	280	193.677	157.1891	967.637	235.862	272.627
1400	515.938	95	1614.66	547.72	1266.34	873.265	678.042	736.2913	1458.46	1260.06	678.458
1401	516.6079	71	7502.8	7227.7	7047.79	3827.21	6222.4	3937.488	14001.9	8019.64	7544.55
1402	516.8257	61	996.03	736.91	434.239	654.106	959.322	390.195	1497.18	1185.37	1224.56
1403	516.8258	77	3323.04	1642.6	2782.58	2983.06	2754.44	1827.928	3759.84	1875.68	2067.59
1404	517.1342	82	64.3463	207.11	98.0135	92.1911	468.521	179.6051	1124.92	399.429	250.546
1405	518.6053	71	10179.6	9734.5	8914.27	4998.75	8040.5	5372.367	19432.7	10764.4	9992.52
1406	518.6951	64	884.092	559.37	596.717	447.361	424.779	372.072	852.103	594.087	675.081
1407	518.8214	61	556.133	567.04	407.849	440.33	790.61	278.3697	1272.38	790.636	936.15
1408	518.822	77	2083.59	1034.3	2218.26	2229.29	1993.52	1018.687	2789.92	1854.04	1312.93
1409	519.2234	83	577.862	413.83	732.261	505.028	498.752	474.0366	1205.78	1028.72	398.903
1410	519.2474	88	420.158	323.75	577.892	339.218	275.601	236.9775	1025.56	779.18	397.47
1411	519.2518	118	921.921	165.9	980.247	361.652	348.201	628.6064	1565.4	707.916	331.856
1412	520.6024	71	7366.84	7117.2	7191.81	3910.7	6471.34	4309.701	15085.1	8480.39	7904.62
1413	520.6914	65	1790.72	1258.9	1080.62	1177.79	1006.14	582.5365	1805.53	1095.97	1189.26
1414	520.8292	79	577.789	162.26	488.235	469.784	559.902	387.5299	1078.48	334.993	255.824
1415	520.9096	86	126760	59012	93257.9	86069.8	66066.5	89996.28	326379	170077	68620.6
1416	521.9122	86	12890.6	6003.5	9292.51	8792.15	7570.33	9756.555	32326.5	18642.5	8246.24
1417	522.5993	71	3651.86	3840.7	3357.01	2290.58	3335.28	2166.521	8157.57	4701.21	3923.41
1418	522.6887	65	1726.31	1208.8	1140.73	1132.74	990.603	652.707	1601.29	1027.52	1236.58
1419	522.9127	86	5200.79	2354.8	3737.66	3434.39	3209.5	4012.521	14105.7	7661.36	2929.23
1420	523.914	91	597.95	330.96	687.786	457.076	486.77	344.5129	1906.69	820.838	412.678
1421	524.5962	72	1322.46	1288.3	1297.54	846.042	1211.04	755.3001	3065.29	1524.5	1477.83
1422	524.6851	66	1121.54	588.43	577.518	584.649	458.881	324.5119	1086.77	407.381	653.377
1423	524.8959	82	2143.26	724.18	1493.32	1466.56	1423.21	1329.012	4341.04	2123.61	939.395
1424	525.9739	89	4295.11	1209.8	3396.09	1515.59	1281.79	1750.724	5064.78	3231.75	1424.44
1425	526.6373	67	1084.05	600.98	841.523	584.559	418.32	390.6046	1464.92	746.503	721.208
1426	526.8533	61	844.9	693.8	313.106	609.558	785.757	387.8257	1596.29	1296.79	1152.85
1427	526.8538	81	9142.99	3600.2	9591.18	7985.64	5413.41	5432.03	16194.8	8941.78	3853.23
1428	527.8601	82	789.491	494.28	713.61	863.763	843.892	571.648	1948.74	957.674	439.358

1429	528.6344	67	1280.81	484.75	874.916	660.704	285.105	386.6323	943.442	754.402	672.155
1430	528.7231	64	847.749	802.87	683.657	821.096	842.294	469.3656	1376.62	857.747	1013.3
1431	528.8518	81	3864.7	1691.5	3801.43	3193.17	2414.38	2454.553	7510.6	3374.92	2075.56
1432	529.1441	89	0	9.864	0	45.948	40.0598	217.1	509.6	165.088	157.99
1433	529.2795	119	2292.31	715.79	2879.85	1006.92	1345.28	1617.916	4509.66	2415.38	1453.44
1434	530.2833	118	1014.99	373.25	1173.79	461.152	644.336	781.5253	1526.14	1022.83	539.224
1435	530.5868	72	1200.86	975.45	902.76	463.834	636.534	482.5587	1585.06	920.583	1021.22
1436	530.6302	67	1194.47	976.26	906.165	458.858	632.122	463.2672	1587.64	920.769	1024.17
1437	530.72	65	2059.89	1289.6	1329.07	1295.57	925.446	719.004	2362.23	1400.25	1898.45
1438	531.7135	69	849.437	560.67	699.171	520.607	653.363	484.9381	1269.87	1085.41	720.967
1439	531.9305	84	2988.8	1135.8	2650.61	1750.91	1184.6	2249.386	7547.95	3734.48	1320.36
1440	532.5818	71	2418.22	2133	2297.7	1104.88	1893.57	1190.655	4567.26	2197.75	2506.88
1441	532.7176	64	1658.63	1098	1058.87	1091.43	917.055	698.892	1018.5	1126.04	1638.22
1442	533.7143	71	825.709	533.06	677.513	445.502	720.458	437.9068	1701.79	849.853	625.354
1443	534.5795	71	2986.11	3062.5	3061.82	1323.35	2526.08	1348.938	5263.81	3041.19	3366.75
1444	534.7146	66	826.65	474.19	492.722	555.007	388.052	462.0117	668.895	385.135	498.96
1445	534.7969	77	574.516	232.21	976.527	552.012	482.869	291.8147	986.712	412.895	213.69
1446	534.9247	85	10413.8	4875.3	7270.2	6830.49	5826.52	7866.519	25585.8	14407.7	5601.39
1447	535.9285	87	1669.35	565.42	1266.74	938.148	993.411	1172.306	4477.89	2055.53	1001.16
1448	536.576	71	2364.86	2256.4	2533.34	1211.63	2170.61	1103.567	4216.75	2478.93	2865.5
1449	536.6655	67	1279.8	559.99	1009.83	618.338	437.224	492.6182	1537.9	747.535	794.661
1450	536.7929	79	88.5908	124.62	198.32	186.473	574.793	18.836	796.55	118.815	58.85
1451	536.8833	84	34199.2	17975	36212.2	26722.4	19436.7	25424.33	78976.7	38918	18143.6
1452	537.8879	84	4235.7	1934.5	4097.71	2970.41	2815.38	2227.167	11104.8	4397.84	2301
1453	537.9846	89	3270.17	1240.7	3123.33	981.183	1333.13	1587.383	3862.14	2480.33	1355.72
1454	538.5733	71	1315.66	1185.1	1369.56	707.913	1178.79	680.9648	2291.73	1383.72	1460.59
1455	538.6618	66	1275.86	587.1	859.592	775.193	631.43	572.2006	1628.83	955.829	830.968
1456	538.7513	63	1211.26	965.79	1058.61	1237.17	1591.12	747.915	1161.54	1430.55	1506.07
1457	538.8832	84	4322.48	1905.3	4391.57	3040.09	2780.96	2936.949	9206.07	4852.55	2326.59
1458	538.9731	90	5558.27	309.75	676.344	1980.92	1956.14	451.8049	2943.09	2013.14	1110.46
1459	539.8895	84	693.665	357.38	599.793	636.753	367.541	592.54	1825.36	994.722	495.705
1460	539.9765	90	1219.19	171.67	508.464	476.76	304.143	285.651	832.505	430.389	348.871
1461	540.3295	177	1271.86	466.27	1648.24	744.825	1219.67	1864.331	3238.74	1275.7	496.074
1462	540.3296	202	399.668	159.31	563.201	247.728	338.384	590.6016	739.431	506.722	154.882
1463	540.5754	71	495.158	355.07	485.951	263.588	314.588	223.629	997.5	499.759	444.596
1464	540.659	67	1590.22	1116.1	1162.1	878.596	844.652	642.1804	1304.65	1154.64	544.883
1465	540.7495	64	2377.66	1625.5	1406.54	1468.7	1806.12	947.778	3448.85	1788.49	2380.58
1466	541.9479	83	1172.62	447.72	1208.21	463.341	383.009	572.4922	1490.9	961.224	643.633
1467	542.7478	63	1460.49	1249.6	2066.99	1837.83	1691.46	1513.428	4027.76	1026.14	1511.93
1468	542.8269	80	2304.58	906.99	2518.95	1757.11	1237.46	1428.349	4804.56	1708	780.856
1469	543.241	113	1061.04	624.9	1117.44	1599.74	1224.71	770.7883	894.734	560.219	350.468
1470	544.6992	64	982.89	535.9	621.049	546.608	532.658	327.0079	1217.78	611.165	738.389
1471	544.7466	64	1459.76	455.13	1151.11	920.112	564.011	751.0133	1865.31	744.654	707.512
1472	544.826	79	981.706	421.61	1137.88	914.006	896.762	666.7173	2024.77	721.043	478.769
1473	545.2756	106	288.192	406.09	401.23	347.599	511.797	338.1054	1206.51	627.829	422.445
1474	546.6946	66	1434.32	782.14	1376.09	1059.99	893.956	556.374	1569.92	1041.76	1209.48
1475	547.6916	71	439.74	414.21	488.087	369.104	392.809	388.4597	1223.97	698.882	439.123
1476	547.9027	82	1028.52	306.29	1300.02	616.076	965.305	825.3307	1590.06	1248.58	529.809
1477	548.6914	66	1284.78	703.17	1100.42	812.286	903.373	486.459	1607.16	1039.32	950.113
1478	548.7813	63	1736.82	1317.5	1079.96	1455.87	966.354	834.561	1330.68	1442.12	2072.64
1479	549.9355	90	2200.62	919.93	977.816	1385.84	1069.84	960.297	3215.44	1405.9	1201.29
1480	550.7785	63	2473.35	1530.7	1482.15	2098.14	1271.05	1085.855	2123.68	2040.03	2518.57
1481	550.8989	84	3564.82	1548.7	2717.37	2349.86	2291.75	2151.169	6722.95	3509.6	1598.2
1482	551.8967	84	393.894	170.23	269.399	332.556	230.884	259.4286	890.414	428.529	307.125
1483	552.7759	63	1175.18	835.78	701.015	1091.43	1382.18	600.846	864.214	786.706	1407.58
1484	552.8564	82	5718.81	2939.7	7045.23	4436.7	4624.72	4629.901	13402	5216.84	3328.32
1485	552.9002	89	4925.78	2682.3	6463.16	4167.96	4522.2	3852.144	1108.84	5099.96	3078.85
1486	552.985	89	527.213	204.75	85.1421	1399.92	1181.91	1054.109	2183.36	1215.84	946.77
1487	553.269	111	3016.75	3375.1	4115.38	4568.35	5477.52	1517.245	2846.41	1495.56	1289.29
1488	553.8677	83	1007.13	481.82	1203.17	685.076	617.269	815.4956	2353.19	1231.03	690.643
1489	553.9688	82	1122.71	333.18	1254.29	541.631	516.543	529.1921	1148.5	538.17	370.74
1490	554.2734	111	1534.92	1395.1	1636.26	2651.56	2596.19	1369.822	1875.64	878.329	616.359
1491	554.2834	110	1366.36	1366.3	1655.07	2482.78	2432.23	1291.323	1704.65	832.592	591.204
1492	554.729	64	1058.02	726.26	763.082	707.818	960.341	529.8767	1441.76	1049.74	894.931
1493	554.7709	62	341.872	260.33	138.675	340.408	406.377	160.528	648.158	519.894	311.669

1494	554.8559	82	1659.01	721.45	1520.75	1000.87	1017.28	807.4312	3373.37	1164.3	778.862
1495	554.9466	88	1603.12	625	1572.37	1127.25	1240.79	702.6408	3014.91	1191.17	704.315
1496	556.596	70	2585.14	2197.8	2012.32	1614.93	2102.25	1474.847	5488.67	3248.9	2610.46
1497	556.7234	65	1282.93	753.19	1171.08	1135.76	1309.65	686.322	2619.94	1329.12	1363.39
1498	558.5925	70	8267.83	7752.7	5730.25	4568.15	6095.7	4890.887	15472	9903.12	8568.51
1499	558.7205	70	1398.18	500.05	851.105	764.577	937.434	477.3332	1786.68	881.649	918.242
1500	558.8089	63	2643.58	2301.6	1656.26	2661.28	3366.18	1321.92	4349.45	2277.14	1893.94
1501	560.5903	70	12377.1	11594	9492.74	7041.92	9178.1	7495.38	22989.2	14377.1	12436
1502	560.8064	62	2660.84	1277.6	1761.17	2447.29	2582.08	1180.335	1602.8	2396.38	1992.16
1503	562.5871	70	10326.4	9991.5	7291.36	6088.09	7845.34	6447.679	19715	12744.2	10232.8
1504	562.8033	62	960.093	509.27	648.081	924.17	530.635	506.3424	1168.79	1228.53	1028.87
1505	562.9204	91	614.622	398.4	328.089	955.064	962.593	826.8661	1760.21	1097.41	392.636
1506	563.9509	89	2741.72	839.49	2064.57	1079.09	1053.9	1048.076	3559.7	1803.97	1008.13
1507	564.584	70	5567.08	5211.3	3791.61	3174.82	4329.26	3220.611	12147.1	7129.1	5417.83
1508	564.7542	63	1230.31	454	1074.87	969.601	591.449	694.9022	1839.1	932.734	959.291
1509	564.9292	87	979.076	360	1029.48	751.003	856.926	598.1217	1987.14	933.871	448.273
1510	565.047	777	201.885	55.104	240.513	206.723	243.382	235.376	645.65	224.527	72.4338
1511	565.0476	758	198.858	129.44	157.383	303.805	300.583	293.4572	458.106	233.406	94.472
1512	565.0477	795	482.758	118.66	379.19	413.215	364.617	441.6502	703.543	338.594	221.624
1513	566.3456	182	1622.01	670.74	2114.26	1108.01	1900.79	2546.152	2543.5	1910.12	751.619
1514	566.5806	70	1990.74	2102.5	1682.93	1312.49	1956.72	1279.494	4770.35	2968.48	2231.34
1515	566.7517	63	1830.43	762.36	1349.5	1133.02	783.552	839.0618	2771.41	1535.99	1306.12
1516	568.3601	291	211.175	81.357	306.03	94.8652	117.051	158.6933	196.898	159.225	60.956
1517	568.361	249	708.483	183.34	1044.1	246.03	235.157	449.7654	388.078	354.13	135.587
1518	568.3621	266	848.044	369.9	1417.36	395.119	540.386	679.1281	830.562	778.056	254.411
1519	568.5779	71	1295.42	862.14	944.714	868	715.013	724.6684	1905.99	986.932	1016.04
1520	568.6228	67	1269.68	1087	821.364	789.12	773.096	790.4778	2543.71	1517.4	880.252
1521	568.7481	62	3862.56	1420.8	2184.27	3063.45	1913.86	2075.852	5256.25	3276.95	828.528
1522	568.838	80	4602.25	1627.5	3737.97	4549.52	3248.29	2652.276	7252.54	3718.64	2194.44
1523	568.8384	61	962.71	1372.7	365.987	526.758	1583.13	497.205	2226.61	1779.66	2080.31
1524	570.26	112	3313.84	3720.1	4003.6	6330.01	6144.69	2597.978	3182.71	1773.56	1203.16
1525	570.6181	66	1644.64	944.59	887.426	972.313	787.464	550.566	1819.86	1161.83	1032.48
1526	570.8348	79	3388.38	1553.4	2262.31	2932.87	2465	1947.545	3354.03	2198	1756.22
1527	570.8357	61	749.7	946.28	211.406	528.066	1076.19	358.5486	1469.43	1163.08	1901.68
1528	571.2623	112	1164.14	1506.7	1576.65	2503.48	2204.65	878.9043	1173.71	602.773	342.896
1529	572.5697	71	2468.81	2343.9	2053.2	1318.03	1722.55	1153.189	4281.32	2686.97	2481.64
1530	572.6145	66	1254.1	2503.9	2122.93	1289.63	1691.62	1281.716	4513.32	3007.56	2731.4
1531	572.8292	79	573.735	194.18	425.933	489.775	375.682	282.701	780.661	265.85	190.567
1532	574.2925	113	774.957	675.85	996.588	1760.17	1174.65	987.0731	1719.65	772.861	201.072
1533	574.5672	70	7406.51	7271.4	6541.59	3621.46	5528.82	4066.678	13331.2	8353.83	7643.87
1534	574.6103	66	7410.65	7064.4	6582.63	3605.02	4964.37	4074.28	13327.6	8343.81	7338.23
1535	574.7837	74	1965.52	1125.3	1645.34	1629.07	2111.4	917.3965	3212.99	1880.38	1279.95
1536	574.7842	61	797.3	632.51	404.313	432.449	685.23	357.57	1193.25	832.644	897.967
1537	574.9193	89	248.529	185.16	265.226	568.271	553.364	402.3499	1517.27	653.931	262.642
1538	576.5647	71	11386.2	10885	9559.96	5206.94	8591.56	5675.433	19653.7	12989.1	12330.1
1539	576.6502	64	11595.1	10613	9589.2	5417.64	7911.68	5693.728	19745.2	13145.9	12333.6
1540	576.7811	74	1859.97	1118.7	1576.27	1524.31	1987.71	938.8783	2731.92	1623.48	1516.83
1541	576.7812	61	714	850.26	438.085	424.843	793.15	335.7686	1028.07	866.274	1164.19
1542	578.5612	71	10007.5	9675.1	8022.09	4621.84	7368.63	5207.219	17908.3	10972.1	11259.3
1543	578.6506	65	1541.93	9439	8051.67	921.765	7348.56	5235.217	1022.86	11039.6	1013.3
1544	578.8679	83	18012.8	7552.1	12987.6	13471.2	8379.91	11143.56	33569.3	17893.1	8450.83
1545	579.0265	915	1407.27	761.86	1506.83	903.644	925.754	896.7168	1250.97	1192.01	1122.31
1546	579.8709	83	2318.58	987.39	1509.61	1933.47	1470.38	1577.909	4845.09	2889.58	1085.3
1547	580.5576	71	5310.11	5271.4	4584.29	2933.31	4262.74	2711.466	10245.6	6862.19	6245.63
1548	580.6466	64	1550.22	860.77	4702.81	3159.64	4679.55	2789.283	10856.3	7230.33	1043.68
1549	580.8648	83	6974.32	3077.7	4749.31	4967.11	3940.71	4229.169	12381.1	6829.61	3046.42
1550	581.8713	88	948.845	502.9	810.341	582.796	599.576	682.9705	2209.23	1237.39	524.153
1551	582.5553	71	2119.84	2327.6	1965.65	1242.56	1824.42	1267.56	4411.47	2818.71	2557.36
1552	582.6459	67	1063.01	2238.8	2027.67	1656.45	1649.63	1505.483	5112.63	2862.25	2556.05
1553	582.8805	91	1384.88	1146.7	909.371	1417.84	1217.5	1294.001	3051.96	1590.63	1137.74
1554	583.923	90	1235.96	474.6	1028.04	665.608	740.714	733.2127	1771.59	1111.68	651.95
1555	584.5528	71	1474.48	1068.6	1061.87	694.631	822.773	607.9568	1485.33	1152.88	1185.38
1556	584.5949	67	1243.69	744.95	1011.16	610.331	642.42	606.4302	2100.52	995.337	856.435
1557	584.6419	64	1365.77	1000.9	1012.82	626.47	893.783	575.9064	2104.33	1021.34	1048.01
1558	584.8127	79	3566.78	1270.5	2836.34	2851	2136.21	1710.981	4603.09	2217.45	1151.49

1559	584.8141	60	2109.96	704.18	939.168	1570.18	1345.09	1123.93	1211.32	977.612	1179.02
1560	586.5912	67	1377	599.66	1027.19	633.761	427.035	460.781	1382.16	601.952	810.033
1561	586.6803	64	737.663	369.78	502.73	469.856	280.223	288.0559	988.45	520.318	489.166
1562	586.809	79	2888.39	1034.8	1736.48	2197.49	1505.13	1190.837	3998.79	1762.28	1095.6
1563	586.8111	60	566.44	520.35	211.18	409.631	598.217	226.4486	985.786	727.566	914.813
1564	587.0164	88	337.691	198.19	449.377	231.195	196.401	162.9824	783.9	659.854	166.091
1565	587.5713	72	207.827	175.12	39.0856	96.16	161.84	119.89	922.485	235.138	159.453
1566	588.5444	71	1116.44	978.74	965.487	716.934	770.629	684.5511	1806.17	1236.27	1071.21
1567	588.5869	66	662.463	569.28	802.786	692.334	623.905	488.5868	1307.31	958.473	763.632
1568	588.6798	64	1536.51	1059.8	831.871	874.222	1120.25	494.001	1314.23	1011.67	1108.42
1569	588.8093	79	534.126	302.27	570.802	406.89	863.334	241.0977	1266.87	788.372	234.285
1570	588.8972	86	97482.5	43553	71614	59479.2	47178.7	65284.58	244113	133878	51632.7
1571	589.5701	70	427.8	360.37	285.291	295.894	316.166	236.9689	1906.35	606.568	410.036
1572	589.6724	70	823.446	477.5	612.338	402.109	478.456	412.7535	1213.79	874.609	604.281
1573	589.8996	86	11890.6	5333.5	8163.65	7885.12	6412.61	7930.475	29505.8	16613.8	5654.06
1574	590.1543	2	0	3.5832	0	0	7.48	0	689.55	0	1.188
1575	590.5415	71	3125.93	2762.8	2764.44	1179.78	2054.62	1325.9	4666.42	3070.09	3463.75
1576	590.6756	64	1353.09	959.6	886.399	992.154	982.03	543.7864	1680.25	1002.2	1279.42
1577	590.9008	86	4934.67	2124.8	3344.2	3269.53	3342.95	3359.472	12731.3	6830.23	2380
1578	591.5724	70	442.15	263.56	347.607	303.24	157.86	271.2973	1387.19	617.188	363.06
1579	591.6727	70	811.749	602.97	733.929	466.299	671.442	502.0001	1454.79	1020.43	727.594
1580	591.9005	88	809.104	447.86	547.613	596.301	542.824	450.6023	1989.81	1129.39	322.668
1581	592.5387	71	3932.72	4044.8	4402.02	2044.17	3456.88	1891.203	7175.2	4278.68	4540.95
1582	592.6735	64	910.309	635.89	546.124	724.828	566.4	333.105	935.314	570.376	728.385
1583	592.8823	83	2298.48	898.38	1574.97	1519.55	1478.67	1253.937	4017.31	2117.67	978.895
1584	593.274	112	1135.45	1049.4	1193.16	1419.11	2345.92	489.5572	1057.25	494.476	337.38
1585	593.6659	70	468.512	426.94	486.592	386.86	510.472	278.3671	1063.52	720.665	427.906
1586	593.9613	89	3962.31	983.85	3063.19	1536.65	1047.42	1414.682	4899.23	1916.55	1518.65
1587	594.5353	71	3999.38	3716	3593.91	1671.9	3045.57	1625.355	6108.18	4014.07	4219.92
1588	594.626	66	1363.29	494.54	786.066	616.604	416.369	389.4143	817.517	642.053	864.368
1589	594.8408	82	9692.76	3171.9	9005.58	7404.1	3586.6	5468.798	16087.5	8142.3	4567.27
1590	594.8827	88	434.196	345.24	321.363	259.276	734.566	399.3634	1478.49	575.303	310.056
1591	595.8439	82	1577.14	511.48	862.491	821.546	685.316	681.0523	2269.92	855.862	438.939
1592	596.5321	71	2156.3	2355.4	2158.57	1247.97	2138.28	1086.519	3617.46	2596.26	2657
1593	596.6212	66	1350.02	518.48	898.634	661.028	963.807	492.2541	1974.08	886.905	889.661
1594	596.7101	63	877.787	616.1	568.683	587.236	931.154	471.375	1612.48	787.725	965.993
1595	596.8388	82	4197.26	1875.5	3675.75	3030	2445.18	2395.509	8183.13	3456.58	1959.04
1596	597.1082	900	120.308	72.006	89.1388	617.75	385.368	307.1578	1235.82	182.155	63.9505
1597	597.3087	113	2582.68	2198.3	2980.11	4259.19	4090.53	2121.499	3544.83	1485.43	625.453
1598	597.5574	71	784.823	562.7	424.672	418.248	485.279	503.0802	2717.9	1185.27	704.317
1599	598.3119	113	1074.56	914.79	1361.44	1766.35	1983.45	1019.998	1439.56	654.757	316.647
1600	598.5289	73	1482.43	1114.1	899.295	183.422	501.573	466.116	1852.98	858.45	1091.79
1601	598.552	70	1978.14	1203.3	1286.31	734.506	885.343	825.5071	3242.53	1509.49	1490.12
1602	598.6193	66	814.985	712.38	700.088	690.577	546.449	552.3273	2560.16	1113.03	867.279
1603	598.7079	64	1603.66	1152.1	823.86	1068.55	1437.53	692.607	1054.03	801.178	1412.27
1604	598.8411	80	824.101	612.7	815.968	813.523	546.614	468.3579	1393.76	646.508	408.493
1605	599.5546	72	468.942	659.35	540.459	419.364	630.114	440.9288	2696.61	1144.35	770.691
1606	599.918	84	2719.13	1047.9	2227.82	1532.37	1274.43	1867.501	6048.77	3777.23	1483.06
1607	600.5519	72	724.729	521.08	645.586	410.016	585.011	422.2123	2115.04	959.567	823.842
1608	600.7051	63	1277.1	884.36	667.633	928.498	1119.66	504.057	765.728	1001.12	1367.36
1609	600.7871	78	922.386	355.53	1173.18	877.367	1471.61	585.6746	1817.39	784.043	429.216
1610	601.5547	72	223.65	270.3	184.608	154.86	143.357	127.908	1202.67	294.053	218.1
1611	602.5592	72	544.342	292.93	571.393	358.504	275.676	258.1787	1556.36	450.089	296.164
1612	602.6539	64	844.391	668.06	713.721	653.37	894.069	355.7743	1808.18	787.303	837.92
1613	602.6948	62	663.872	497.36	499.39	603.824	703.818	272.769	945.913	687.613	671.542
1614	602.9121	86	9586.57	4228	6110.12	5675.18	5025.17	6795.189	24813.2	13077.8	5091.04
1615	603.9144	86	1449.1	766.13	1128.12	993.294	952.156	1148.016	4963.69	1934.57	931.915
1616	604.5444	72	690.091	518.73	702.192	332.553	424.498	323.703	1284.27	906.206	652.43
1617	604.6542	65	1194.19	761.22	872.702	854.219	897.398	493.3984	1701.9	1030.15	1107.1
1618	604.8704	84	31750.1	14260	31091.7	21624.1	17985.9	21568.16	68011	33806.9	17331
1619	604.92	89	14732.8	5385.7	14031	10877.6	6280.07	5566.076	1064.72	16570.8	6390.69
1620	605.5457	70	694.843	387.39	538.286	369.201	407.828	295.5454	1704	643.571	447.312
1621	605.8745	85	4399.75	2131.2	4257.97	3133.23	2493.81	2918.825	9148.73	5869.87	2469.19
1622	605.9708	89	3366.48	1022.2	2787.06	1023.99	960.856	1404.505	4376.68	3135.3	1425.7
1623	606.0698	774	99.5806	22.024	112.766	100.241	86.4253	94.35018	143.163	98.4372	31.928

1624	606.0717	764	103.055	48.255	136.832	106.961	124.865	127.3806	165.855	133.712	51.2563
1625	606.0736	712	1034.71	339.6	2102.14	1090.03	829.645	557.3333	2366.35	1192.37	565.77
1626	606.0754	696	786.109	416.43	769.931	581.065	673.696	700.2866	979.256	728.467	400.798
1627	606.0763	739	233.558	121.2	339.136	264.005	245.545	332.5249	434.783	292.442	158.064
1628	606.5398	72	802.593	668.25	791.217	342.838	669.58	250.3972	2185.5	1009.73	863.785
1629	606.6502	67	1377	747.09	1027.01	968.709	635.544	611.4805	1653.35	1024.74	959.131
1630	606.7399	62	1376.6	835.1	867.923	889.799	1124.44	477.66	1566.79	1022.24	992.123
1631	606.8711	85	4045.56	2156.4	4144.29	2818.39	2795.15	2769.83	9434.43	4743.03	1966.37
1632	606.9624	88	4738.82	276.39	685.976	1849.68	1682.15	429.2964	2865.46	1954.87	885.088
1633	607.5427	72	372.68	592.68	531.073	292.146	556.878	335.6388	2007.09	554.358	629.899
1634	607.8617	82	780.508	387.54	771.592	601.599	454.594	573.3759	1228.65	900.079	407.762
1635	608.5122	69	924.662	593.59	1226.86	341.696	544.835	442.5653	1635.93	963.224	1123.14
1636	608.7356	63	1522.22	945.74	660.061	1086.38	741.789	335.2995	1146.62	1319.51	1212.75
1637	608.8562	83	1127.11	398.6	1110.85	939.356	593.5	718.0735	2037.19	1206.19	471.548
1638	609.9303	83	1271.5	332.09	1285.96	616.313	511.507	461.104	1417.62	740.194	551.29
1639	610.5073	69	906.872	678.12	893.846	238.07	549.194	291.2457	1244.88	837.424	747.398
1640	610.517	74	578.088	600.19	515.518	172.602	521.039	173.42	908.58	709.56	740.912
1641	610.7342	63	1172.38	844.99	678.315	886.187	1394.16	431.6151	881.677	1068.24	1402.67
1642	610.8154	81	2449.6	916.61	3418.72	2231.82	2719.13	1545.082	4725.86	1883.71	1040.97
1643	611.1446	894	5789.68	2777.8	3250.94	2585.06	3545.76	2325.189	4841.6	4953.21	3262.2
1644	612.1469	894	1703.14	771.51	923.292	728.36	953.202	642.7726	1483.42	1345.42	968.548
1645	612.3038	111	551.833	309.53	546.238	474.159	407.07	205.3797	330.346	211.397	210.101
1646	612.5037	74	279.224	393.82	234.552	71.02	385.866	108.0378	670.667	403.308	442.884
1647	612.6861	63	812.237	412.93	528.766	554.703	703.818	281.568	948.567	704.738	687.405
1648	612.8124	80	1301.34	442.92	1363.54	1114.94	653.15	736.1644	2418.82	1172.13	509.065
1649	613.1432	893	895.538	430.27	463.183	385.307	549.195	361.3799	709.554	705.986	513.985
1650	613.3044	112	808.136	519.75	875.021	1215.1	841.675	828.6786	1060.71	610.497	183.895
1651	613.5287	72	219.656	243.8	291.06	100.125	80.9699	85.272	892	276.911	292.573
1652	614.5487	70	889.77	619.16	728.394	595.404	627.151	421.8953	1496.4	904.079	661.792
1653	614.6813	64	1418.12	817.82	1017.22	742.787	868.757	552.5977	1809.5	1091.87	1163.16
1654	615.3441	91	323.411	244.76	264.677	347.728	230.898	75.81608	621.463	276.353	267.032
1655	615.5266	72	157.015	205.55	357.038	122.49	168.526	137.352	1085.18	195.752	294.857
1656	615.8902	83	980.299	384.4	1343.09	497.724	607.307	758.6875	3148.39	1420.92	494.975
1657	616.5515	70	2071.76	2034.9	1541.58	1548.06	1967.4	1319.271	5377.81	2747.94	2419.72
1658	616.6783	64	1229.95	529	1123	688.886	957.145	425.477	1256.83	864.377	1100.49
1659	616.768	62	565.307	820.51	227.261	436.669	584.237	298.845	958.533	1173.48	1178.63
1660	616.77	62	1244.1	840.88	709.92	985.144	727.387	607.8746	2005.92	1311.31	1205.81
1661	617.5512	71	352.781	351.13	254.678	264.33	185.833	266.475	1553.33	588.107	426.89
1662	617.9228	90	2161	872.44	963.932	1027.44	1197.9	881.851	2467.28	1388.72	1000.65
1663	618.5494	70	3740.96	3238.3	2721.29	2497.56	3091.77	2324.746	9458.7	4862.55	3888.26
1664	618.7654	62	828.583	1066.1	277.782	522.163	772.027	324.945	1244.67	871.509	1516.63
1665	618.8862	84	3338.11	1413.7	2851.23	2445.66	2439.18	2401.864	8036.55	3809.82	1644.45
1666	619.5485	72	775.39	615.82	499.984	410.667	500.111	370.3269	2439.72	1078.04	461.471
1667	620.5455	70	3489.96	3435.8	2533.34	2095.57	3118.31	2178.024	9692.51	4657.33	3910.82
1668	620.7631	62	394.08	453.2	246.186	326.442	496.041	230.985	741.321	499.745	728.19
1669	620.845	84	5885.53	2971.1	7286.05	3990.17	4511.26	3878.342	15133.8	5745.37	3417.85
1670	620.9729	90	567.111	133.31	50.1564	1104.86	1018.04	890.9049	2039.08	1155.45	989.501
1671	621.2573	113	785.943	641.46	1052.85	1280.85	1101.2	410.7644	1017.08	470.498	213.633
1672	621.5459	72	519.567	357.75	301.84	243.071	299.802	359.7413	1981.77	788.538	542.141
1673	621.848	82	1358.57	713.43	1625.2	975.677	626.308	811.9755	2745.17	1218.03	732.801
1674	622.5428	70	2207.7	2179.3	1586.92	1538.81	1950.71	1457.558	6103.89	3458.16	2231.34
1675	622.7151	62	541.227	623.48	252.768	215.19	405.665	182.7	609.617	337.098	539.78
1676	622.8431	83	1142.35	673.01	1986.49	1319.32	1202.93	1040.077	3023.54	1080.11	808.789
1677	622.8882	89	1364.94	287.1	562.364	726.174	849.424	437.7398	2411.06	929.527	281.238
1678	622.9335	88	1288.62	341.31	624.302	903.94	1201.72	490.7583	3081.56	1161.22	469.647
1679	624.54	71	1234.21	1156	888.917	848.699	1071.86	700.876	2832.37	1711.91	1315.86
1680	624.7112	63	1217.78	922.65	834.336	911.088	1057.94	635.6762	2289.08	1174.95	1311.37
1681	625.5368	71	704.746	616.54	520.242	277.796	545.214	293.0644	2085.27	970.127	750.759
1682	626.322	111	881.491	849.35	1703.78	719.933	1175.52	137.2353	577.126	270.885	460.525
1683	626.5374	70	1342.13	932.1	959.274	730.667	852.554	895.9781	3686.87	1747.56	1403.59
1684	626.7077	67	963.277	443.26	636.339	761.893	426.468	508.6228	1199.7	880.165	588.437
1685	626.7962	75	1878.32	1350.1	1616.39	1655.23	1823.91	927.5749	3604.96	1741.49	1755.5
1686	626.7979	61	604.52	890.74	256.329	399.852	1006.17	286.3143	1302.93	874.126	1292.81
1687	627.5345	71	1198.73	838.3	877.331	622.978	958.626	720.6721	3555.72	1621.02	1136.21
1688	628.5344	71	1050.93	859.49	686.314	439.215	700.421	732.711	3491.03	1562.75	1247.46

1689	628.5777	64	582.089	410.44	413.421	400.412	271.148	222.5664	1004.98	425.448	468.29
1690	628.7941	77	1647.06	1144.9	1400.56	1493.68	2218.06	799.7644	1831.13	1545.21	1485.43
1691	628.7964	61	616.59	736.84	255.401	385.029	693.874	271.9986	1015.81	572.032	1172.06
1692	629.5298	71	829.92	577.41	590.278	345.333	498.785	483.5563	2321.26	1171.61	815.222
1693	630.5289	70	974.794	884.43	735.113	557.08	903.898	594.9123	2612.75	1531.09	1156.77
1694	630.576	66	609.021	359.62	429.659	278.186	447.559	213.4293	902.1	369.58	405.428
1695	630.7955	62	294.98	343.16	69.3328	129.993	285.6	135.0267	510.835	379.27	484.155
1696	630.9033	89	571.562	365.49	446.302	733.272	539.307	572.858	2259	931.777	324.865
1697	631.5311	72	246.461	206.96	284.381	181.636	232.147	62.60922	1092.03	453.103	271.071
1698	631.9376	90	2590.98	733.63	1887.92	956.036	816.394	1147.462	3384.32	2094.8	890.45
1699	632.5256	71	2428.81	2138.3	1622.87	1322.52	1965.03	1253.091	4215.29	2597.03	2736.78
1700	632.7419	62	588.774	490.18	285.398	362.477	426.45	211.9886	703	679.486	705.989
1701	632.7428	72	1159.21	596.09	782.793	776.142	1040.41	522.307	1731.66	1374.06	901.301
1702	632.93	89	1292.34	395.72	969.406	589.48	561.964	653.2644	1609.97	1084.56	649.02
1703	633.1228	894	565.214	304.92	353.508	284.427	352.039	252.9621	466.323	503.369	364.74
1704	633.5263	72	506.73	346.98	454.164	134.667	343.578	242.4442	1460.34	728.799	567.863
1705	634.5232	71	3893.95	3589.3	3344.53	2028.27	2979.05	2187.735	9221.66	4636.34	4510.44
1706	634.7398	62	1007.3	535.72	312.011	341.28	414.908	285.795	1021.73	762.078	875.42
1707	634.8602	83	664.061	397.58	604.551	682.309	497.651	596.1568	1913.04	788.25	440.265
1708	635.5217	70	1094.16	714.96	808.092	528	598.277	363.6663	2896.02	994.358	918.953
1709	636.5204	71	3873.95	3692.6	3380.88	2252.69	2965.17	2091.246	8132.25	4616.79	3917.01
1710	636.6094	64	575.79	324.38	441.518	277.763	266.126	143.3479	861.408	392.416	392.674
1711	636.735	62	266.347	328.7	155.537	195.812	347.406	128.3294	483.413	421.182	415.066
1712	636.8241	80	3754.43	1602.7	3095.09	3207.52	2561.16	2153.686	6175.46	2996.88	1617.36
1713	636.8279	60	442.26	715.58	159.297	438.539	967.616	295.554	1137.08	858.042	1075.65
1714	637.5192	71	741.074	452.92	548.5	218.667	363.476	392.9728	2218.63	787.986	742.078
1715	638.5171	70	2499.68	2588.6	1964.31	1480.16	2135.73	1280.683	5269.43	3035.79	2550.65
1716	638.6073	65	683.989	403.7	455.09	279.149	477.975	268.6699	910.071	389.911	449.151
1717	638.8232	80	2682.38	989.92	1785.65	2190.31	2099.18	993.7307	3741.54	1560.93	991.219
1718	639.5179	72	172.532	217.16	87.1488	77.0337	186.813	114.1578	862.787	383.853	281.412
1719	640.5143	71	1126.38	1244.3	991.384	711.381	907.568	646.4519	2416.23	1488.86	1300.6
1720	640.6063	64	578.939	313.52	376.205	317.005	269.908	185.4568	606.708	407.498	403.05
1721	642.5091	71	825.878	644.79	621.274	380.19	674.883	458.2462	1924.63	1141.72	1061.79
1722	642.5302	100	4.6384	0	4.42181	19.0174	0	4.015059	2505.99	0	4.63766
1723	642.7696	75	1168.44	898.49	1177.53	1128.45	1524.45	813.6618	2771.48	1418.93	1132.16
1724	642.772	61	422.45	483.33	188.26	258.61	520.67	199.665	741.321	1151.12	732.84
1725	642.909	91	131.961	84.833	153.593	373.771	236.02	342.2435	1004.01	561.868	200.812
1726	643.5118	71	597.17	411.76	546.967	235.764	253.247	223.5912	1235.08	407.231	508.969
1727	644.5078	71	783.905	567.29	700.461	274.542	498.337	262.1712	1601.67	810.424	851.306
1728	644.7654	60	435.12	472.7	200.093	303.036	410.62	233.058	992.98	525.012	793.86
1729	644.769	75	1345.57	855.03	1221.85	1436.08	1403.91	719.7623	2203.57	1314.58	1163.93
1730	646.6382	66	637.526	432.95	453.559	471.356	480.785	232.6259	868.511	519.258	581.051
1731	646.8558	83	11845.5	4674.6	7971.29	8342.01	5646.36	6184.265	21647.1	11709.2	5094.96
1732	647.528	72	538.218	504.57	366.52	341.902	508.071	386.3122	2141.58	967.487	562.073
1733	647.6333	70	437.57	364.1	447.519	325.008	298.328	369.9902	1059.99	727.481	403.986
1734	647.8575	82	1798.9	736.07	1639.98	1326.65	1262.1	1058.088	3791.88	1605.97	1072.62
1735	648.5006	71	1295.75	975.67	1204.24	541.333	875.992	674.7248	2738.29	1563.11	1041.22
1736	648.5253	72	1230.97	1116.4	1244	498.162	878.18	675.2733	2096.38	1172.07	1226.46
1737	648.6365	65	868.357	413.2	425.387	485.398	378.003	326.3752	1169.98	459.877	486.522
1738	648.853	84	4687.71	1696.9	3371.36	3278.23	2406.59	2409.804	9214.07	4666.07	2093.04
1739	649.1236	69	301.868	473.49	219.229	414.773	1119.22	191.5423	1084.03	563.558	510.251
1740	649.5255	72	653.521	652.34	442.111	384.192	514.449	400.9019	2528.18	1009.73	527.573
1741	649.6277	71	661.509	592.33	620.078	495.646	570.208	425.477	1420.86	927.528	650.459
1742	649.854	83	945.856	321.87	563.829	531.147	507.228	467.8915	1520.88	911.997	465.125
1743	650.497	72	1528.31	1653.3	1698.94	767.295	1507.52	849.062	3029.12	1779.24	1895.77
1744	650.6353	63	515.507	362.8	340.493	347.438	346.532	202.377	857.027	450.505	565.787
1745	650.8661	88	1028.13	601.87	631.191	961.863	1104.33	856.1907	1677.64	987.602	579.759
1746	651.5191	72	491.231	296.8	249.288	256.32	238.928	218.5095	1400	640.852	349.757
1747	651.8754	88	1574.97	469.34	957.672	708.057	723.708	608.6299	2256.31	1086.13	651.687
1748	651.9133	90	1751.82	482.12	1050.31	821.897	763.151	793.0811	1885.6	1131.17	682.978
1749	652.4938	71	1592.66	1739.6	1722.94	807.543	1471.78	830.4445	3005.3	1898.03	2093.37
1750	652.5817	67	477.417	247.01	369.392	190.836	200.944	174.588	457.176	246.313	306.369
1751	652.7989	78	2427.48	983.54	2368.7	2159.1	1774.58	1352.422	4145.71	1911.11	1098.66
1752	653.3015	98	2435.31	2225.3	2971.12	3416.27	2939.34	2047.636	5099.73	2603.36	3001.58
1753	653.3351	113	533.589	266.12	463.119	1229.6	725.815	250.852	604.186	368.239	241.986

1754	653.4066	135	217.342	293.54	206.403	223.398	415.907	216.8802	440.105	261.682	326.018
1755	653.5149	72	266.5	286.47	278.674	211.128	324.215	224.049	1060	668.543	451.733
1756	654.3046	98	875.084	915.86	988.353	1493.41	1350.4	790.7848	2313.32	1251.88	1188.53
1757	654.4916	71	1141.65	1023.7	1214.81	538.229	889.679	631.4207	1966.67	916.011	1614.52
1758	654.7986	79	2137.92	949.08	1697.33	1851.13	1497.24	977.165	2686.66	1573.73	896.142
1759	655.5155	71	1121.74	858.17	561.977	422.667	631.44	560.8188	3211.7	1222.37	1091.86
1760	656.5148	71	1496.99	1105.4	833.562	563.744	1020.53	838.901	3243.86	1752.84	1518.48
1761	656.667	65	813	378.18	451.854	500.621	347.605	335.1082	1142.39	644.69	596.388
1762	656.8848	86	46333.9	21686	33367.3	27752.1	23514.8	31527.66	121742	69257.2	25099.1
1763	657.382	84	92.9023	45.943	146.054	81.9138	47.8896	120.354	1290.33	188.422	21.129
1764	657.5125	71	1034.06	805.19	774.72	693.68	831.339	551.494	3399.37	1734.36	1511.83
1765	657.8876	86	6409.83	2673.5	4944.77	4293.91	3715.89	4168.5	17955	9288.81	3695.39
1766	658.5108	72	1068.96	853.88	776.39	545.606	843.272	620.6654	2870.25	1569.36	1084.38
1767	658.6646	64	877.619	486.81	731.726	650.548	587.909	424.866	1302	906.279	627.918
1768	658.8871	86	3414.62	1340.7	2250.59	1798.92	1709.24	2178.891	7375.28	3962.62	1637.78
1769	659.5106	71	590.964	617.87	427.284	308.154	387.163	386.251	2082.6	934.496	647.174
1770	659.8584	84	871.63	409.67	849.964	722.903	383.482	575.9173	1733	1045.42	419.197
1771	659.8942	89	860.992	406.73	838.785	717.487	376.71	564.8404	2140.85	1042.94	415.871
1772	660.5094	72	368.964	207.55	281.314	175.278	206.825	134.734	1385.45	491.436	338.589
1773	660.6598	63	953.087	327.89	762.945	712.778	490.184	404.2603	1343.19	946.389	435.902
1774	660.8688	86	1608.57	754.46	1049.11	1071.76	813.768	948.5431	3294.66	1983.01	760.272
1775	661.5108	71	361.76	254.27	260.1	165.333	179.085	186.4956	1095.41	461.183	364.39
1776	661.9474	90	3173.72	878.31	2286.03	1108.02	719.861	1255.049	3326.46	2234.74	1033.3
1777	662.5061	72	671.44	620.88	633.456	380.633	405.926	235.7837	1326.99	660.5	839.788
1778	662.829	82	7417.16	2439.3	6824.33	5125.81	3823.36	4216.289	12677.9	6546.93	3243.61
1779	663.503	71	742	678.06	864.32	361.514	690.794	504.8701	2180	1139.08	1054.62
1780	663.8336	82	1249.07	538.02	1033.66	902.622	620.68	836.0099	1686.9	994.32	619.826
1781	664.5018	72	1081.91	1067.3	962.232	536.893	888.353	579.3765	3055.82	1498.09	1225.83
1782	664.6071	63	644.467	430.71	562.009	266.487	298.785	210.2137	670.388	390.332	501.678
1783	664.8281	82	3367.75	1403.6	3178.76	2385.78	2321.05	1687.454	5535.4	3143.3	1629.54
1784	664.873	86	776.716	267.66	588.318	554.997	778.083	381.3928	3515.59	982.935	254.195
1785	665.501	72	1062.33	784.01	834.793	566.95	811.451	591.4573	2566.17	1314.62	1182
1786	665.6084	71	475.02	464.52	653.979	303.673	440.009	392.9428	1297.73	792.302	589.712
1787	665.8273	82	378.559	240.29	533.432	285.655	444.227	271.0496	1172.02	594.067	277.745
1788	665.8661	87	329.659	141.64	356.04	163.754	236.546	105.5344	1268.4	383.087	104.47
1789	666.0592	82	384.321	229.28	603.993	899.98	580.689	317.4568	611.124	563.281	211.721
1790	666.4969	72	1021.21	865.52	744.828	341.178	784.615	524.3424	2438.89	1203.74	1086.38
1791	666.5043	71	1018.22	849.9	713.039	341.504	784.933	524.7686	2438.89	1173.39	1077.74
1792	666.6959	64	929.495	633.25	508.737	552.267	867.557	355.731	1223.96	799.581	809.845
1793	666.826	80	341.835	189.18	250.51	357.68	225.313	247.05	604.468	325.95	171.84
1794	667.4978	72	596.288	512.09	463.636	199.064	487.195	351.6773	1517.33	512.548	636.064
1795	667.5977	71	550.774	443.6	387.477	266.962	459.746	284.0672	1232.93	606.778	549.551
1796	667.9043	84	2485.72	612.45	2426.56	1216.25	1139.93	1712.136	6226.29	3647.12	1313.43
1797	668.6951	62	736.68	416.87	457.391	445.725	370.635	325.0539	932.8	593.355	613.441
1798	668.7744	78	812.619	371.6	1040.17	811.321	728.993	434.4209	1712.26	714.084	445.439
1799	669.4	110	609.963	691.91	570.042	740.143	1300.18	370.6587	1671.51	907.577	664.046
1800	669.4499	418	11.4019	6.712	2.67591	79.9869	38.8211	26.34912	2640.18	15.724	0
1801	669.4499	344	4.85714	3.772	1.80686	63.3133	25.4107	15.6336	1534.84	13.9689	0
1802	669.4502	565	4.13925	2.7348	1.36982	24.073	8.77878	7.533333	910.98	4.99383	0
1803	669.4502	459	5.96518	3.762	5.45733	51.8829	27.7155	13.7709	1821.99	11.8551	1.23938
1804	669.4504	514	1.33864	0	3.76947	61.684	20.2279	16.03096	1719.7	6.66	0
1805	669.4506	542	5.02588	2.5241	2.5152	30.4348	11.241	12.51871	1067.74	4.99474	3.7104
1806	669.4507	501	0	2.5154	3.778	29.95	13.8893	5.038476	869.355	7.52114	0
1807	669.4507	402	13.8997	6.6833	2.51341	98.9162	27.7666	28.7942	2810.44	17.5972	2.49088
1808	669.4508	489	0	4.1336	2.52183	35.539	22.7623	12.38738	1538.61	5.45939	2.73164
1809	669.4509	368	2.52188	3.768	0	75.0844	32.823	8.768	1797.31	7.635	1.24514
1810	669.4511	306	0	0	0	5.232	2.456	1.348	108.24	5.056	1.264
1811	669.4515	439	4.62429	0	1.25543	30.464	12.2446	6.072	945.945	6.06187	0
1812	669.4515	383	7.55673	2.5135	5.39044	88.8745	35.1909	34.02	2497.61	11.2975	0
1813	669.4515	324	16.2227	3.9695	5.29214	130.982	61.9954	23.72339	2328.81	22.1677	1.46415
1814	669.452	672	0	2.5215	0	15.1593	8.79455	2.517091	353.723	2.49983	0
1815	669.4523	287	7.5624	2.5016	17.766	52.1129	40.1583	8.954824	311.312	14.94	3.7026
1816	670.4014	110	195.769	294.84	232.906	380.81	747.914	143.6425	676.038	436.953	417.756
1817	670.4541	413	0	1.2574	0	20.1962	7.256	1.258609	557.931	2.503	0
1818	670.4939	72	398.418	408.49	402.529	205.783	283.024	218.7145	1045.33	498.44	476.976

1819	670.7723	77	925.694	325.29	880.095	686.231	568.933	443.8924	1474.83	700.319	459.926
1820	670.8992	86	5895.24	2772.3	3889.17	3104.17	3043.1	3961.417	13096.1	7695.85	3012.21
1821	671.2683	137	1810.78	516.2	2047.82	595.243	468.902	349.6928	2324.36	1139.38	1064.27
1822	671.9052	87	1134.33	468.29	822.434	838.008	658.458	838.5	2996.22	1831.03	869.811
1823	672.2713	138	892.714	203.24	769.504	254.299	195.788	173.685	1109.16	426.621	326.033
1824	672.4894	71	749.03	555.95	671.608	302.78	371.302	383.8533	896.364	844.956	613.601
1825	672.6439	65	764.243	381.25	492.062	429.085	194.821	277.485	1030.97	597.302	572.457
1826	672.8587	84	21186.8	8971.3	20719.6	13169	12985.4	13637.2	43202.7	23580.2	11323.3
1827	673.2673	138	716.402	229.99	800.533	247.312	203.841	173.5291	919.6	470.817	322.455
1828	673.4905	71	695.59	468.81	566.02	185.333	374.089	327.6993	1521.33	678.598	759.367
1829	673.8619	85	3521.63	1577.1	3104.96	2017.06	1939.87	2332.12	8441.56	4459.75	1934.86
1830	673.9588	90	2699.41	599.29	2359	934.458	832.501	1078.902	2734.32	2213.85	1299.74
1831	674.5112	70	1390.52	1178.3	777.095	879.13	1036.43	741.2657	2796.06	1644.9	1142.16
1832	674.7249	60	392.94	420.47	141.242	275.928	295.96	151.032	555.44	427.062	571.16
1833	674.8578	85	2765.54	1327.6	2985.7	2164.01	1832.16	1973.186	7142.33	3635.45	1724.7
1834	674.9501	90	3549.71	1313.5	2670.58	1056.96	1269.39	1758.005	2057.48	1605.91	1861.54
1835	675.504	70	322.38	256.05	423.675	190.19	202.135	252.3517	926.674	380.592	354.665
1836	676.5073	70	2379.64	2114.1	1550.81	1429.64	1805.9	1379.095	5613.76	3015.65	2408.56
1837	676.724	62	1031.72	560.59	560.744	756.791	569.985	425.9078	534.328	856.685	790.268
1838	676.8479	83	1300.42	284.69	995.454	984.927	631.179	517.2256	2088.62	1036.06	489.327
1839	677.5073	71	654.975	410.75	384.038	329.745	402.195	390.3859	1608	827.495	356.407
1840	677.9212	86	1344.36	444.25	1215.14	500.32	377.229	558.2228	1540.43	588.056	492.4
1841	678.5046	70	2509.69	2303.5	1667.23	1487.39	1935.91	1454.898	6248.7	3157.22	2760.65
1842	678.7224	62	625.82	378.49	362.712	461.487	509.171	332.8145	939.425	495.963	565.847
1843	678.8025	81	2559.59	1072	2947.08	1892.91	1790.79	1314.749	4318.79	1975.25	1156.63
1844	679.502	70	462.343	363.21	302.786	257.241	281.627	272.9136	1501.37	501.546	443.318
1845	679.801	79	265.28	180.06	388.776	299.64	361.026	258.0233	902.4	289.504	164.574
1846	680.5017	70	1828.18	1759.6	1213.53	1158.07	1449.52	1102.244	4523.84	2768.72	1797.02
1847	680.8011	81	1413.67	599.12	1626.02	1123.97	872.125	706.7925	2625.79	1151.57	636.253
1848	681.2965	136	5674.94	2452.6	4799.5	2191.8	2573.57	2348.68	13562.5	6086.48	4276.08
1849	681.4502	1	0	0	0	0	0	0	817.073	0	0
1850	682.3	136	2441.3	881.56	2062.53	868.173	1020.98	881.5754	5482.39	2505.27	1884.4
1851	682.4524	1	0	0	0	0	0	0	463.96	0	0
1852	682.4982	70	943.244	911.94	781.431	658.755	753.621	636.8941	3232.65	1484.9	1117.15
1853	682.6703	65	900.01	385.79	544.296	503.337	586.808	372.5944	1103.33	617.146	727.877
1854	683.3027	137	692.018	218.84	554.413	333.74	274.471	203.9554	1203.31	594.023	368.155
1855	683.4652	304	1.26411	0	1.52952	34.9408	7.54982	1.26	5531.91	6.86	0
1856	683.4661	418	5.06752	1.3424	5.35183	38.089	8.62691	6.2736	2685.72	1.42945	0
1857	683.4662	400	1.26352	1.3387	0	21.5642	8.8284	1.2508	3328.55	1.25728	1.24544
1858	683.4662	366	1.32564	2.6489	0	42.63	10.576	4.194113	5429.44	3.15626	0
1859	683.4665	336	1.68711	0	0	33.488	2.53148	4.308571	3271.22	1.45621	0
1860	683.4666	268	3.82951	3.1672	5.33639	147.824	14.8452	11.12503	5804.25	15.4223	1.57789
1861	683.4667	285	3.77935	1.3235	2.53513	59.5608	11.3001	2.679385	6580.21	4.00212	0
1862	683.4673	322	1.55374	0	1.34364	33.9642	6.26625	4.017867	6493.85	0	0
1863	683.4962	72	417.073	447.09	371.565	283.285	368.064	234.4516	1261.58	817.699	572.637
1864	683.8799	83	902.93	285.93	1558	632.618	826.547	768.3036	3273.03	1343.95	637.496
1865	684.4683	265	1.90478	1.5685	3.42154	55.7649	11.6647	1.561682	2774.29	4.54493	0
1866	684.4704	304	0	0	0	14.3364	3.77358	1.260261	3061.53	0	0
1867	684.4705	323	0	0	0	12.3248	0	4.18469	2096.52	0	0
1868	684.4711	286	0	0	1.26606	11.0488	1.25745	1.396667	1838.18	0	0
1869	684.4957	72	1076.65	834.38	743.286	572.814	728.252	654.322	2714.3	1420.22	1113.26
1870	684.7549	75	1157.4	722.84	840.03	943.828	1018.8	538.3071	1746.2	1040.56	756.506
1871	684.756	62	256.41	440.05	54.9276	303.036	182.28	87.234	461.12	415.308	517.45
1872	685.4816	295	1.26135	2.5128	5.57368	2076.88	79.0769	10.55262	1205.03	6.57108	0
1873	685.4822	275	2.65673	0	1.338	2200.41	62.1289	7.995556	747.941	8.43369	0
1874	685.4826	185	8.75875	1.6259	10.5483	5333.03	109.239	15.52923	167.197	11.8388	3.05455
1875	685.4828	207	4.68821	7.0246	12.2907	5497.72	134.496	29.2092	223.521	3.65616	0
1876	685.4829	134	790.335	517.29	422.309	25392.3	1012.03	381.85	2793.84	890.195	625.51
1877	685.4833	228	11.2381	0	0	3379.59	71.1669	17.5641	162.291	0	0
1878	685.4936	71	1029.17	864.2	598.282	1039.53	817.749	448.4857	2737.42	1267.11	1082.39
1879	685.859	88	1421.81	688.41	855.998	775.184	790.493	632.5094	2543.59	1350.23	961.464
1880	685.9096	90	1497.18	701.84	933.256	848.042	827.476	715.107	1652.75	1516.64	1028.69
1881	686.4849	212	59.4882	78.421	16.9947	4082.83	124.149	59.13861	136.119	107.037	37.77
1882	686.4859	124	0	2.9909	56.0148	4348.46	71.4371	12.28373	135.214	122.787	2.67892
1883	686.486	185	6.628	4.815	23.0442	1970.95	29.4236	11.23691	50.2738	45.5117	0

1884	686.486	149	5.02	1.7624	22.5771	1405.28	19.838	3.5984	28.6373	56.9474	0
1885	686.4919	71	1179.07	1025.9	789.195	767.651	807.146	604.1915	3009.33	1552.21	1369.73
1886	686.6595	62	621.417	304.74	461.391	366.089	394.394	317.1257	926.175	531.52	273.669
1887	686.7499	62	474.032	488.75	179.059	250.465	487.308	202.3543	926.987	524.323	788.651
1888	686.7518	76	1235.85	788.88	781.098	835.438	1452.9	513.7727	1643.89	997.208	941.449
1889	686.8733	84	3041.52	1239.1	2368.74	1658.1	1305.81	1908.828	6548.38	2991.21	1460.9
1890	687.4917	72	808.686	733.22	508.158	474.904	607.262	479.484	2120.37	1182.63	916.941
1891	687.8716	82	882.253	246	554.371	317.584	311.517	447.9596	1286.67	877.854	379.325
1892	688.4884	70	953.189	853.36	826.084	549.725	619.116	531.0568	2250.32	1069.02	909.158
1893	688.8325	84	5014.88	2665.4	6383.67	3892.71	2919.16	3408.634	10833.7	5614.91	3248.33
1894	688.961	88	716.362	308.64	223.471	1107.14	909.955	714.3359	1943.69	1055.63	879.222
1895	689.837	83	1333.15	451.18	1105	1031.88	1092.48	1070.676	3420.91	1123.14	691.629
1896	689.9364	83	1091.83	341.95	1200.44	579.38	427.335	618.0708	2000.74	1010.77	498.943
1897	690.4842	71	1541.83	1471	1153.52	641.844	1019.25	739.6812	3241	1745.15	1414.22
1898	690.8316	83	1608.4	704.63	1711	870.144	964.837	879.8456	3957.47	1259.2	612.509
1899	690.8765	88	1533.17	207.12	1063.27	923.327	721.757	714.266	8410.94	2617.35	434.594
1900	690.9202	88	1329.88	208.57	1125.67	950.004	736.384	853.9117	8261.98	2632.63	438.132
1901	691.3795	91	313.81	29.956	165.923	353.695	359.86	215.0217	1819.26	901.243	136.073
1902	691.4851	72	359.438	361.73	340.918	257.655	341.135	183.8678	1117.33	714.695	509.342
1903	692.4826	70	2932.46	2640.6	2038.61	1226.7	2134.4	1341.811	5214.72	3500.4	2791.17
1904	692.6983	62	461.76	348.65	164.783	385.33	226.38	165.354	445.4	386.576	517.45
1905	692.8723	89	636.27	50.21	199.054	255.942	228.333	236.8128	1912.14	979.184	131.681
1906	693.4499	189	46.2119	36.179	25.2298	223.86	99.0634	70.02223	1414.19	51.39	19.4652
1907	693.451	105	868.181	753.27	660.142	809.541	900.324	571.5112	10833.1	1056.72	839.521
1908	693.4517	212	45.3178	31.622	31.0013	205.042	85.188	58.79374	1257.57	38.7874	33.4291
1909	693.4531	5	0	0	0	0	7.9938	0	7013.51	0	0
1910	693.4811	70	937.28	627.99	693.254	424.669	471.218	512.4831	2247.93	816.229	653.582
1911	694.4566	86	28.5987	87.084	22.7673	170.194	157.677	82.64817	2257.72	201.815	87.2076
1912	694.4569	5	4.33435	1.8276	3.6594	65.88	15.9391	7.3184	9638.74	16.7916	1.67391
1913	694.4784	71	2976.76	2929.8	2337.71	1377.22	2131.15	1624.228	6606.92	3820.07	3186.48
1914	694.7826	78	1401.14	639.95	1205.39	1097.39	1315.46	855.8349	1738.04	1160.91	822.086
1915	694.7836	61	310.05	430.24	152.714	276.377	405.552	130.5	520.083	471.807	589.687
1916	694.8575	91	1303.79	607.78	1353.81	1213.31	1197.28	926.5506	1660.35	1398.53	719.155
1917	695.4631	2	6.55453	0	1.19467	79.8709	5.94533	4.7392	864.236	12.2289	0
1918	695.4648	471	4.17021	1.2568	2.77573	30.4087	11.3433	15.13018	1433.12	6.27438	1.25164
1919	695.4657	661	0	0	3.782	19.7224	8.78748	0	440.443	0	0
1920	695.4657	384	1.2608	5.0295	1.32669	83.664	37.7455	25.17236	3368.82	10.0532	8.40864
1921	695.4657	322	4.55318	2.6705	2.67135	100.376	41.3783	22.60728	3001.44	12.7647	2.82435
1922	695.4662	500	0	3.7625	1.37094	48.3757	15.1667	16.31644	1407.14	6.26	1.35753
1923	695.4663	304	7.57234	5.2642	12.4751	164.04	48.7185	16.3696	3074.69	27.5959	2.61682
1924	695.4667	367	0	6.6766	6.69391	83.1029	29.3695	22.92986	3049.27	9.93474	1.24232
1925	695.4667	350	2.88206	5.2586	4.78484	124.233	60.628	32.97674	4372.44	15.0934	2.71612
1926	695.4668	527	0	1.2611	0	21.5061	9.75748	6.294839	1021.69	4.99148	0
1927	695.4668	432	5.05414	2.6891	2.92105	84.4047	17.668	21.59695	2487.71	8.09354	0
1928	695.4674	415	5.06514	5.3607	1.34035	81.3547	34.5667	17.60114	2821.98	6.68783	1.33009
1929	695.4685	511	1.36859	1.26	0	32.7684	13.9646	8.782919	1402.87	4.08882	0
1930	695.4782	71	756.15	572.4	699.771	312.39	447.325	319.77	2076.14	1005.38	816.543
1931	696.4695	386	0	0	1.49875	7.57263	1.25895	0	614.399	1.2584	1.25053
1932	696.4759	71	2166.73	2040.3	1817.11	1156.87	1870.17	1182.703	4302.09	2823.29	2512.19
1933	696.7799	78	1172.56	800.8	946.359	1074.06	1503.99	531.98	1633.76	1126.88	1103.9
1934	696.7807	61	313.56	468.78	47.0808	167.569	344.76	114.576	504.283	490.053	685.691
1935	697.8912	86	320.829	116.13	323.323	279.17	211.396	198.3167	1795.72	670.753	106.503
1936	698.2872	136	977.57	879.2	1299.78	525.898	930.361	455.938	4879.53	1592.69	1819.94
1937	698.4718	71	1282.37	1124.2	961.609	678.667	993.761	593.5449	2769.81	1520.9	1362.53
1938	698.8657	85	1069.21	517.18	1082.82	985.547	690.729	806.2295	4940.48	1464.08	436.049
1939	698.8896	84	1233.89	520.51	1055.95	1033.81	690.495	865.6825	2682.45	1986.72	440.191
1940	699.2899	136	485.511	412.9	566.063	256.364	333.256	146.5473	1942.84	639.644	689.194
1941	699.3656	86	220.344	114.13	263.107	299.611	243.009	229.068	2569.85	684.491	33.5458
1942	699.8743	86	1383.46	480.21	1186.3	704.381	704.378	747.23	1967.7	1491.62	596.749
1943	699.9251	88	1549.42	481.84	1151.35	706.514	715.289	762.2388	3101.76	1504.72	599.985
1944	700.4689	69	856.417	506.84	744.461	491.175	405.926	448.5723	1705.01	720.053	684.56
1945	701.4689	72	576.431	372.33	530.915	194.91	325.207	247.8218	1208	660.631	565.197
1946	701.8394	83	619	335.46	665.484	647.574	381.317	395.1925	1603.77	544.113	404.198
1947	702.4687	71	815.29	614.49	587.583	225.333	458.988	379.6517	1388.23	907.872	804.583
1948	702.7274	75	995.598	460.98	797.487	652.445	1131.05	365.8672	1395.94	935.838	703.228

1949	704.4643	71	644.796	566.49	513.503	194.991	350.211	213.1378	1455.15	701.451	682.992
1950	704.8124	81	2735.5	1119.7	2959.21	2294	1851.44	1566.736	3940.97	2561.02	1168.44
1951	704.8135	59	206.15	281.35	46.1956	235.299	381.771	96.57	617.608	486.474	603.75
1952	705.8129	79	306.762	160.8	288.1	321.368	154.403	249.3	553.197	279.015	88.275
1953	705.8762	84	704.571	124.52	482.378	303.55	316.8	199.1636	1730.49	593.94	209.496
1954	706.4574	70	843.223	701.39	643.324	334.758	572.37	365.2522	1527.05	809.383	829.819
1955	706.8118	80	1692.71	681.16	1288.87	1457.73	1074.2	761.7867	1604.66	1147.68	564.473
1956	706.8526	84	1038.48	766.1	906.731	999.337	1262.43	400.491	3356.45	1451.27	660.633
1957	707.4703	2	0	0	0	0	1.19059	0	1878.99	0	0
1958	707.8502	84	206.986	127.13	526.218	201.149	341.585	182.5	997.624	292.48	238.713
1959	708.4563	71	1455.37	1366.8	1192.19	548	1042.95	599.6675	2663.95	1486.21	1493.97
1960	708.4718	1	0	0	0	0	0	0	778.202	0	0
1961	708.8253	82	401.748	229.88	368.943	496.078	538.698	142.4	1063.22	289.51	180.5
1962	709.4815	322	1.53238	1.3383	5.35455	62.8423	25.0808	16.011	7459.92	32.8921	0
1963	709.4822	305	10.094	9.2588	8.39503	74.2023	29.0602	16.36944	10529.8	10.5511	0
1964	709.4823	266	14.0305	6.5262	18.8747	99.876	60.9273	32.75081	6803.19	53.4414	1.60916
1965	709.4825	384	1.26023	2.5131	0	32.9294	7.54491	7.5264	3116.63	10.0526	0
1966	709.4827	415	1.267	0	4.31657	17.71	5.03513	1.25728	1565.52	5.006	2.49056
1967	709.483	366	7.95386	1.3244	5.61692	48.314	34.372	11.1843	5809.78	11.0469	1.24414
1968	710.4536	71	1285.8	1479.5	1279.03	693.2	1065.1	779.366	2878.58	1562.77	1598.19
1969	710.4857	367	1.32421	2.6428	0	25.4867	6.59931	8.364655	2709.71	6.26057	0
1970	710.4858	305	3.79034	5.6142	2.80267	13.0932	9.82489	10.08107	2373.23	6.98889	0
1971	710.486	266	7.31953	5.0082	1.5567	34.714	35.8928	27.33349	2201.03	12.0976	1.41848
1972	710.4863	286	3.7822	6.591	6.338	25.6568	10.0576	20.02	4155.27	7.96667	0
1973	710.4864	320	1.56011	2.691	1.34623	22.334	7.5217	9.383818	3197.33	24.6262	0
1974	710.7569	78	980.357	419.42	704.165	845.936	506.378	498.1928	1196.93	893.176	391.588
1975	710.7582	62	264.18	184.12	56.2354	165.557	248.92	110.67	403.48	410.084	310.47
1976	712.4504	71	1050.27	975.53	924.985	538.422	935.707	519.0191	2006.51	1208.28	1129.63
1977	712.7577	78	850.821	394.33	784.078	905.241	725.291	417.5598	1275.28	578.754	432.012
1978	712.7581	60	311.91	312.09	107.24	213.965	321.44	193.998	504.35	415.308	518.76
1979	713.4697	72	199.237	137.77	123.385	50.8368	161.07	124.5693	1072.54	313.463	148.208
1980	714.4513	71	596.836	517.4	576.849	327.211	562.46	286.4039	1189.49	801.847	721.526
1981	714.8431	84	8169.64	3175.4	5238.1	4635.19	3208.71	4510.631	14133.5	8311.97	3630.68
1982	715.8454	84	1126.48	571.42	1222.45	1019.39	915.361	810.3623	3744.22	1878.06	752.467
1983	716.8402	84	3119.41	1262.1	2079.26	2094.4	1588.95	1733.333	6974.62	3359.41	1520.6
1984	718.8515	86	1026.81	658.29	574.169	758.914	606.271	530.3665	2340.69	1122.27	557.5
1985	719.4868	324	9.0632	23.334	0	26.0955	22.544	27.4497	1260.09	4.56497	20.4273
1986	719.4887	218	193.848	432.59	37.5819	514.231	224.751	205.067	18301	56.694	482.87
1987	719.4888	286	35.2524	107.33	11.3967	156.98	45.1769	76.65842	5589.85	5.51314	116.871
1988	719.4892	262	72.9867	156.25	9.21389	236.847	91.9288	86.88393	9802.88	15.3053	164.831
1989	719.8563	86	1377.6	426.54	845.213	802.057	604.689	607.6298	4362.07	1612.86	525.166
1990	720.3558	89	66.8175	14.443	102.383	92.953	157.856	90.54327	885.36	278.186	0
1991	720.4909	286	18.8762	34.134	22.7748	77.3886	43.8681	39.776	2218.68	45.8897	44.3427
1992	720.4914	263	27.5349	80.217	20.0293	132.557	70.5343	82.6956	4266.31	49.8343	84.816
1993	720.4919	217	99.1757	236.17	9.11467	253.178	111.301	81.77639	7507.11	16.9053	248.428
1994	720.7863	80	1613.45	776.39	1604.48	1754.3	1335.76	880.578	2429.92	1529.72	805.39
1995	721.2892	95	270.474	457.56	407.251	404.595	499.8	301.1377	1262.71	781.319	573.354
1996	721.4932	217	62.3056	74.235	22.3665	293.519	115.571	48.23	2446.29	30.3085	76.892
1997	721.5016	1191	32.7338	106.5	5.3131	117.126	77.4088	55.67172	425.809	0	110.914
1998	721.5028	1137	19.0235	20.141	11.34	16.3647	6.34118	15.07765	267.4	21.44	25.1556
1999	721.7915	79	236.655	179.85	234.52	220.92	332.175	71.37	754.926	205.375	138.278
2000	722.466	74	150.29	160.24	195.412	98.6667	152.554	130.5469	875.798	304.381	188.844
2001	722.7835	79	1513.03	492.26	1305.78	1280.87	1198.82	680.4129	2235.58	1184.06	455.063
2002	724.872	86	28279	11881	20070.1	15179.7	13179	17034.94	73340.1	38016.4	14007.1
2003	725.377	87	432.555	75.888	329.526	382.627	218.422	343.0465	3530.94	1313.09	232.969
2004	725.875	86	4374.58	1784.2	3416.8	2494.14	2257.89	3115.403	10772.6	6585.18	2103.82
2005	726.6463	63	388.989	173.22	212.559	189.36	163.89	147.0238	310.231	170.072	233.254
2006	726.8748	86	2228.31	901.74	1954.44	1572.23	1150.61	1449.828	6156.68	3144.23	1049.91
2007	727.8458	83	1130.87	372.95	954.125	826.199	753.74	725.8631	3387.02	1697.92	403.008
2008	728.8503	83	1607.01	606.43	1064.35	974.471	875.281	793.4034	2934.98	1854.73	654.971
2009	728.8581	82	1605.75	545.27	1257.33	1060.06	846.521	939.1429	3053.2	1617.26	603.354
2010	729.9355	88	2016.01	626.65	1530.9	612.437	435.271	790.714	2266.63	1390.21	510.65
2011	730.8171	82	5772.42	2193.3	4523.26	4120.02	3052.41	2806.03	8647.21	5217.91	2334.12
2012	731.8205	82	964.869	446.86	1386.01	703.042	788.893	708.6604	2679.04	1023.59	433.202
2013	731.8797	88	869.09	197.99	771.564	419.248	523.69	431.1438	2726.79	1287.73	355.395

2014	732.4696	70	774.306	496.13	428.128	491.501	360.645	438.8729	1253.25	823.688	605.978
2015	732.8139	83	2191.28	1015.9	2938.17	1961.57	1800.04	1657.779	4542.46	2332.7	1173.57
2016	732.8583	85	1216.25	992.18	1716.36	1238.06	902.859	960.628	7027.71	2736.73	1138.8
2017	733.364	87	753.41	264.52	916.364	620.536	373.813	362.838	2673.71	1177.06	222.292
2018	733.8589	87	641.146	172.57	567.232	243.534	438.61	199.8514	2541.02	782.097	316.636
2019	734.4671	70	1384.27	1147.7	826.77	867.334	1134.03	806.0279	3074.51	1814.1	1328.68
2020	734.8138	80	785.361	433.23	725.158	741.814	789.553	441.3136	1049.25	634.753	418.396
2021	734.8502	87	708.419	449.08	713.127	724.267	794.949	449.1529	2114.43	829.411	455.975
2022	735.4652	71	399	259.57	233.146	270.667	126.023	251.769	907.742	624.574	292.576
2023	735.8333	82	376.768	224.37	444.328	306.587	746.817	239.2681	1844.38	674.36	146.872
2024	735.8908	85	1747.66	564.8	1554.07	746.819	831.78	955.4227	4139.59	2439.21	978.782
2025	736.4639	70	1684.94	1484.8	1060.09	987.667	1454.8	1124.719	3666.91	2528.95	1560.43
2026	736.7594	77	815.064	290.92	942.184	679.778	1105.97	491.909	1375.94	671.693	364.205
2027	736.887	84	512.431	76.433	377.679	217.571	192.313	157.446	1067.57	670.469	178.961
2028	737.4635	71	358.854	330.74	220.15	205.143	300.549	249.403	1387.8	576.986	445.883
2029	738.461	69	1396.4	1154.7	882.214	933.073	1197.72	884.9775	3252	1731.71	1226.53
2030	738.7555	77	759.056	377.61	912.327	732.243	760.388	475.3234	1392.41	643.048	418.678
2031	738.887	86	3716.64	1371.2	2251.14	2361.24	1935.64	2464.45	9079.94	4889.42	1965.35
2032	739.8895	87	1004.68	366.86	864.563	517.368	779.445	505.4532	2913.12	1431.31	606.58
2033	740.4575	71	807.917	738.53	533.768	425.546	682.839	635.3163	1995.17	1190.56	786.709
2034	740.4919	700	70.1549	32.006	70.7254	85.8265	113.954	99.1608	70.0769	73.7772	14.4412
2035	740.4938	650	61.7081	26.418	52.9044	108.939	83.6752	52.696	64.6423	44.3685	11.2046
2036	740.8468	84	14878.9	6710	14545.1	8915.79	7923.44	8816.677	32156.9	17376	7502.95
2037	741.3489	86	277.207	71.953	242.868	152.282	263.69	196.5044	1703.25	425.704	130.456
2038	741.8489	84	3180.82	1455	2264.62	1650.86	1931.95	1722.156	7275.39	3325.68	1371.13
2039	741.9466	90	1937.96	1187.2	1809.21	1224.67	1177.18	1523.761	1465.04	2892.76	1037.42
2040	742.4566	71	918.327	627.66	521.648	471.785	577.859	434.0653	1300.74	865.319	792.87
2041	742.8449	85	2514.46	1101.6	2409.94	1597.21	1477.51	1546.959	4973.17	3049.55	1237.59
2042	742.9377	91	2763.22	484.34	849.577	854.853	793.319	588.068	1301.83	1335.36	885.995
2043	743.4541	72	756.77	447.51	437.992	405.333	401.946	417.9026	1772.78	898.649	578.502
2044	743.4822	1	0	27.984	0	0	21.5402	0	1031.46	0	7.34808
2045	743.4885	41	218.756	278.44	210.935	294.287	250.965	112.6217	2249.18	177.119	80.9137
2046	743.4887	9	144.228	181.73	121.173	269.09	168.926	53.03511	2926.76	99.4187	66.9786
2047	743.8504	87	890.988	184.56	650.809	420.151	404.598	185.6861	1447.4	844.916	427.018
2048	744.4506	71	866.481	617.66	532.421	533.6	633.78	565.5977	2022.53	1075.72	859.798
2049	744.4925	2	6.83264	7.71	15.4923	54.8608	7.116	7.974643	499.122	8.95286	6.19015
2050	744.8307	82	1070.23	504.79	1024.92	777.821	781.134	568.4648	2422.44	975.519	476.958
2051	745.4477	71	915.743	720.33	526.515	564.894	700.498	443.12	2375.33	1085.99	728.09
2052	745.5031	383	15.12	20.099	5.0168	53.52	25.2305	25.132	643.153	10.0389	10.2418
2053	745.5036	240	109.377	115.47	66.7503	205.935	105.247	110.8167	5150.19	85.5868	89.124
2054	745.5037	323	33.0653	56.127	11.3082	153.929	63.8906	79.35765	2400.01	22.5619	25.7886
2055	745.5037	286	104.419	140.47	44.3157	297.419	111.548	131.1669	7680.19	55.5172	106.817
2056	745.5037	215	93.6604	121.08	80.2053	148.769	83.7793	70.37262	3259.46	94.6072	51.9476
2057	745.5042	305	59.3978	91.78	25.5446	171.509	85.0567	87.05935	3818.95	28.6944	48.8491
2058	745.5048	263	111.508	149.93	66.8194	282.385	145.344	133.5537	8903.43	75.1249	100.454
2059	746.4484	71	869.449	909.5	692.473	517.988	748.104	602.889	1933.33	884.577	729.134
2060	746.5066	327	68.9359	66.096	31.4759	70.8074	152.451	118.6096	906.03	45.6702	9.75908
2061	746.5066	215	374.642	263.04	222.283	119.015	479.783	331.4921	1572.15	237.446	72.7267
2062	746.5071	190	403.471	231.68	254.816	85.6512	479.308	406.2636	2518	303.737	35.6906
2063	746.5073	285	243.303	151.41	131.805	166.015	326.594	267.7865	3464.69	149.798	61.2911
2064	746.5076	265	316.875	217.03	195.223	197.951	461.472	367.3915	4516.47	209.162	87.7764
2065	746.508	367	64.1769	33.868	32.637	41.4265	111.358	76.28163	492.393	32.54	7.44171
2066	746.5082	354	166.007	151	74.1664	149.199	307.067	229.5374	1484.69	116.321	33.8676
2067	746.5085	239	317.92	208.86	186.738	139.333	391.31	346.8404	2386.21	209.419	62.2348
2068	746.7908	81	2033.81	857.47	2630.11	1744.33	1983.68	1156.26	3168.16	1582.25	1252.39
2069	747.4471	71	780.357	558.86	442.498	665.89	938.823	585.1773	1164.76	923.5	523.361
2070	747.5067	84	705.279	485.39	414.995	944.524	1217.55	730.7594	3702.83	144.794	303.301
2071	747.5153	114	1386.31	833.42	810.801	1217.99	1638.91	1431.958	6310.51	684.285	220.06
2072	747.517	52	777.009	515.54	482.641	986.229	1217.88	698.3534	1281.93	526.387	125.726
2073	747.5176	1191	73.0326	97.988	155.179	190.436	136.48	128.0722	2529.28	0	128.76
2074	747.5196	1139	18.9882	26.46	87.285	43.8941	33.15	20.12	768.22	222.516	32.695
2075	747.5202	2	297.599	169.5	466.893	1184.33	463.211	289.773	744.262	399.55	100.637
2076	747.5206	28	441.345	198.65	337.115	770.958	464.221	428.9133	385.978	250.505	100.727
2077	747.5637	361	463.038	867.67	327.944	787.058	639.399	712.2647	67.5142	621.728	496.056
2078	747.5638	155	210.489	175.03	593.888	120.971	163.542	229.5685	248.296	1243.24	164.562

2079	747.5642	379	440.452	714.7	259.422	751.368	539.033	586.2571	41.0631	537.929	503.736
2080	747.5652	273	3.72	4.992	52.9955	0	20.368	3.786	3.936	152.292	11.079
2081	747.5653	438	469.815	513.45	169.515	810.25	588.111	731.2834	47.5955	366.32	444.704
2082	747.5654	333	323.532	788.34	444.084	434.893	343.676	503.9674	72.9778	787.26	503.723
2083	747.5661	232	62.7936	52.866	715.716	35.1384	95.41	68.0615	116.754	1607.71	16.9984
2084	747.5663	393	704.403	1143.3	348.659	1242.71	982.581	1060.053	59.0675	814.826	736.045
2085	747.5667	212	39.6037	62.276	520.384	22.7665	88.0695	53.6796	100.093	1345.7	42.6556
2086	747.5674	181	59.7969	32.476	382.326	33.4322	92.3294	75.45508	77.3185	934.895	25.4453
2087	747.8009	80	303.707	150.54	378.43	261.685	599.975	177.876	1013.61	355.1	143.648
2088	748.4436	71	1022.61	1016.7	769.114	667.256	915.694	797.9908	1875.38	1170.98	1057.03
2089	748.5201	2	195.295	183.12	170.752	581.16	176.73	274.2568	304.007	235.488	101.537
2090	748.5212	1163	746.398	449.92	455.813	363.533	1021.06	749.3231	2316.95	812.188	316.764
2091	748.5231	1084	371.021	283.77	310.308	194.903	622.366	462.8658	2647.69	521.385	176.308
2092	748.5231	1043	1099.91	693.89	874.755	404.932	1138.68	848.0513	4714.05	1129.01	456.343
2093	748.5239	1190	425.03	256.11	208.541	241.172	614.895	451.0604	1574.8	0	212.211
2094	748.7871	81	1450.63	498.97	1287.88	967.03	952.867	614.912	2338.31	796.417	539.46
2095	748.8372	83	1153.81	484.55	1032.28	869.634	977.206	520.9355	1988.72	1289.51	569.609
2096	749.8364	83	561.436	176.39	486.685	429.653	260.562	355.995	1530.32	598.745	300.329
2097	750.4413	70	1428.08	1406.1	1064.37	739.292	1034.53	857.9424	3069.13	1990.75	1664.61
2098	751.4455	71	541.352	402.19	478.968	233.165	184.3	295.6757	1145.6	774.925	489.035
2099	751.8646	84	671.677	370.21	1342.51	496.267	659.937	410.4369	1918.88	1057.92	534.488
2100	752.438	71	1916.97	1987.1	1413.9	865.82	1343.1	1027.573	3934.27	2470.64	2061.52
2101	753.4373	71	716.87	382.73	363.87	220	326.333	282.4076	1657.33	698.363	650.316
2102	753.5461	140	1012.33	666.33	1205.24	2441.15	1086.99	1007.55	4046.47	1631.26	428.345
2103	753.5467	1116	47.3511	65.229	27.3255	71.9091	137.575	63.68	118.594	84.925	45.5262
2104	753.5467	1069	362.6	481.52	291.415	709.3	1010.83	730.5035	1038.83	401.526	255.792
2105	753.5471	218	417.137	283.42	447.711	650.847	566.016	460.6748	1083.82	583.422	133.139
2106	753.5476	1098	288.673	282.09	236.795	535.436	657.908	535.3047	765.116	402.864	190.446
2107	753.5477	1077	370.523	411.85	293.617	805.783	852.239	569.4915	824.181	365.353	210.589
2108	753.5479	265	201.858	163.34	242.471	366.426	318.508	333.6231	793.871	355.523	82.3788
2109	753.5481	186	448.918	181.36	421.825	870.606	318.808	559.7713	909.328	579.06	97.309
2110	753.5484	1054	1237.06	1091.5	1037.1	2262.35	1749.31	2292.585	3376.63	1668.09	712.07
2111	753.549	167	555.641	226.15	594.894	731.577	508.212	643.3458	1342.65	628.93	163.12
2112	753.5492	44	356.818	311.72	316.436	508.488	505.127	574.6593	1604.25	569.794	157.762
2113	753.5493	116	731.48	583.8	773.7	1132.14	840.75	959.7786	2327.72	1026.63	414.048
2114	753.5494	17	447.76	411.47	516.14	800.06	639.792	502.7749	2124.9	624.733	239.863
2115	753.552	243	381.133	227.79	431.944	563.637	489.784	470.587	947.799	532.207	135.02
2116	753.8522	86	1065.33	517.14	1120.65	827.788	859.601	764.9105	3924.67	1788.3	598.598
2117	754.3603	88	420.218	114.43	229.401	219.004	27.1663	140.8279	1494.73	375.001	124.141
2118	754.4342	71	1798.64	1453.8	1181.37	868.621	1410.32	816.3613	3449.1	2022.14	1637.35
2119	754.5468	265	200.542	162.42	234.6	376.049	348.445	334.5799	585.297	322.634	62.4068
2120	754.5509	169	314.704	189.82	297.36	403.623	423.567	374.0421	879.178	419.682	106.064
2121	754.5512	144	628.321	397.74	701.421	860.315	748.801	721.7186	2420.63	945.686	285.736
2122	754.5516	116	459.077	326.42	516.327	775.093	552.523	541.375	1473.79	695.403	251.954
2123	754.5516	15	238.827	230.31	302.846	425.431	341.449	309.1473	1251.1	356.361	136.974
2124	754.5527	44	242.241	212.07	214.304	393.992	363.707	385.9127	1018.9	369.981	117.66
2125	754.5531	194	461.707	245.9	463.58	662.23	487.446	452.4279	1037.74	566.778	117.041
2126	754.8594	85	2181.55	916.6	1989.41	1263.84	1338.45	1522.058	5365.97	2841.69	1125.42
2127	755.3543	84	290.143	134.37	180.345	200.362	201.348	235.5859	1289.16	176.371	82.6924
2128	755.4627	212	735.539	281.2	568.781	877.076	555.376	580.5189	1320.37	784.349	125.232
2129	755.4634	191	299.547	172.91	278.521	355.55	298.934	336.1109	628.042	373.926	58.3956
2130	755.4636	167	458.209	276.81	372.32	539.098	513.855	421.7583	867.131	477.846	136.384
2131	755.4638	102	1643.03	1543.7	1392.29	2619.49	2424.29	2044.363	3642.46	2044.83	752.818
2132	755.4644	263	139.612	87.58	151.714	233.566	177.424	215.9318	362.246	222.836	27.4388
2133	755.5461	144	2030.97	1355.1	1840.08	2656.66	2616.75	1969.782	4151.64	2340.84	810.624
2134	755.5462	1051	691.694	598.7	630.871	1082.67	1079.58	1099.552	1338.26	880.604	407.306
2135	755.547	1094	288.177	243.46	161.369	461.612	434.073	476.7111	624.619	360.801	128.322
2136	755.547	1072	273.194	319.48	242.219	528.984	629.268	393.6926	664.491	334.231	157.817
2137	755.5474	220	755.019	347.49	641.961	991.102	646.735	719.3809	683.94	914.75	146.564
2138	755.5485	17	271.571	283.35	272.245	340.48	385.869	345.3012	1216.04	390.569	124.722
2139	755.5488	192	322.858	202.62	317.239	427.68	375.192	368.0267	692.441	470.607	73.968
2140	755.5488	43	273.375	257.86	218.359	382.964	373.404	385.2169	968.945	400.481	115.353
2141	755.8661	87	824.976	297.47	517.62	381.939	563.16	494.8051	2012.06	612.598	297.022
2142	756.4322	70	970.606	844.97	772.102	602.166	814.392	516.4626	2060.49	1268.09	962.571
2143	756.4627	140	527.323	368.71	418.543	575.165	640.148	438.6757	1765.11	600.407	194.826

2144	756.4647	113	392.053	410.35	414.002	649.961	748.941	514.998	1424.72	650.957	177.783
2145	756.8203	84	5033.47	2087.5	5656.34	2859.82	3524.48	3228.85	9574.4	4687.49	2376.18
2146	756.9495	88	538.899	126.65	288.998	633.19	564.111	623.844	1756.9	1029.69	599.481
2147	757.8257	83	1429.84	604.01	1516.61	905.186	931.88	829.6462	2876.19	1186.92	656.171
2148	757.923	83	1159.78	259.83	933.337	462.725	448.349	380.6033	1157.42	573.97	302.218
2149	758.8168	83	1317.44	595.52	1276.91	939.869	848.536	976.9667	1941.75	1139.84	628.831
2150	758.8648	86	1740.94	615.18	1602.61	1293.49	923.381	1206.418	9710.72	3779.5	653.589
2151	758.9103	88	1092.73	621.22	1602.16	1361.98	974.831	1105.03	9754.99	3712.67	693.148
2152	759.3695	87	519.242	228.18	605.376	591.228	409.287	269.8858	3512.07	1734.52	106.156
2153	759.8653	87	740.404	141.42	554.787	298.373	355.465	218.9621	2582.86	926.456	221.154
2154	760.4252	72	520.416	462.9	439.52	214.638	251.931	237.614	1203.57	745.462	732.505
2155	760.7931	80	262.628	255.82	569.951	472.928	575.303	368.9046	972.866	604.708	185.962
2156	760.8558	88	494.088	219.38	352.323	416.209	546.474	298.517	1416.32	799.067	212.304
2157	761.4404	7	4.14316	10.647	1.77682	14.2356	6.25888	5.319	1488.15	3.89853	0
2158	761.8424	84	1105.14	376.06	911.958	722.798	596.102	513.7514	3106.27	1451.13	288.057
2159	762.3496	84	305.88	114.91	483.021	169.453	391.856	120.45	1467.09	362.675	68.5533
2160	762.4155	72	542.199	528.15	493.745	288.836	483.367	221.232	1056.13	405.771	498.271
2161	762.7708	81	999.782	451.23	856.752	718.282	885.555	668.5324	2256.46	752.766	565.316
2162	762.8394	84	853.238	539.26	889.404	706.211	1148.08	501.5768	2506.2	1350.86	708.719
2163	763.5748	158	631.095	451.3	647.256	1029	902.052	807.321	1548.85	773.177	256.257
2164	763.5754	99	2435.61	1867.3	2160.13	4575.69	2366.72	2623.55	5503.85	2494.01	1276.3
2165	763.5764	178	571.088	387.94	579.686	949.034	949.207	771.1799	1342.17	796.072	252.067
2166	763.5767	147	982.758	641.36	931.826	1401.22	1254.91	1111.274	2893.67	1081.52	406.93
2167	763.5773	211	979.029	656.85	925.389	1130.75	1268.88	1134.449	2905.75	1359	349.968
2168	763.5773	35	714.641	543.25	721.458	1116.83	857.349	908.1024	1860.94	931.187	316.118
2169	763.5775	116	3525.79	2594.8	3303.32	5163.83	4671.66	3924.32	9146.74	3836.95	1756.42
2170	763.5783	263	229.712	175.47	232.612	410.721	372.06	416.6788	676.015	373.39	75.4393
2171	763.5791	2	255.882	174.44	291.898	813.732	366.458	395.5785	1068.53	415.758	125.49
2172	764.4335	72	774.266	655.65	614.744	310.225	498.932	450.5828	1221.86	836.749	653.103
2173	764.578	214	507.404	305.19	516.757	748.82	595.358	546.2135	1062.84	614.056	179.428
2174	764.5819	116	2098.78	1609.8	2261.74	3554.2	3023.42	2626.812	5508.44	2550.24	1101.51
2175	764.582	191	309.122	213.37	318.143	421.748	412.599	349.5997	735.806	429.803	102.197
2176	764.7623	79	627.598	358.66	643.334	661.957	918.862	416.999	1154.47	612.193	338.582
2177	765.8765	87	664.037	58.526	518.376	408.051	314.214	340.6149	3283.62	1088.03	256.882
2178	766.4231	72	887.43	677.5	810.219	390.02	651.678	485.5607	2134.44	968.994	937.892
2179	766.4357	68	685.458	479.74	681.626	377.012	385.135	449.9931	1332.26	884.655	685.767
2180	766.855	86	1516.75	636.64	1785.92	1250.91	1052.27	1266.428	8115.3	3048.81	660.884
2181	767.3582	85	644.448	202.97	606.94	433.322	346.417	332.6937	3260.15	1281.25	226.124
2182	767.8562	84	1297.19	475.14	1186.96	730.457	850.531	734.3651	3479.38	1191.71	683.341
2183	767.9105	86	1251.84	368.5	1116.48	668.237	516.564	608.6299	1668.22	1115.8	510.883
2184	768.4156	72	977.625	820.88	896.381	422.878	837.63	420.5644	1870.06	1096.84	990.756
2185	768.8497	84	821.776	339.19	664.289	530.961	597.181	498.4814	1679.45	1045.37	473.845
2186	768.8876	88	734.481	178.64	489.308	371.419	274.031	432.3097	1744.89	692.254	414.974
2187	769.8319	84	1150.43	353.26	956.984	602.65	606.362	465.0985	2576.58	1227.7	456.23
2188	770.4157	70	822.331	498.65	706.701	408.227	394.543	433.6907	1549	687.609	809.96
2189	770.8226	83	868.023	444.79	983.291	569.418	695.419	531.0399	2183.16	792.146	308.435
2190	771.4358	72	497.047	357.28	356.872	214.585	273.79	197.9839	968.889	738.239	446.239
2191	772.4258	71	741.367	637.76	514.898	401.534	570.137	460.8374	1822.14	929.21	833.22
2192	772.4532	43	439.724	359.09	390.076	602.997	574.28	547.2871	1262.14	680.453	169.018
2193	772.4542	12	517.691	489.14	516.473	646.141	652.75	551.691	1687.51	696.126	229.73
2194	772.7985	82	2113.99	948.87	2370.39	1508.78	1574	1261.747	4307.6	1846.37	956.677
2195	773.4296	72	762.09	591.15	528.371	358.667	432.069	456.4706	1755.59	1001.8	806.151
2196	773.7997	82	552.923	231.47	673.831	461.161	386.377	374.9321	1695.05	565.531	229.004
2197	773.8622	86	675.249	198.49	620.266	300.849	391.658	289.7036	2645.31	970.45	150.335
2198	774.3652	87	150.143	93.304	293.578	125.472	86.5933	91.7055	936.381	309.191	117.107
2199	774.428	72	681.472	597.98	513.55	352.176	503.842	386.251	1585.22	951.654	768.104
2200	774.7827	84	1236.18	532.56	1503.95	1252.15	1263.16	782.87	1233.02	1608.6	474.065
2201	774.7975	82	1236.18	532.56	1503.95	1252.15	1263.16	782.87	3727.93	1230.09	474.065
2202	774.8397	84	1043.02	554.7	1304.28	1147.72	973.12	788.2384	3914.81	1650.18	510.321
2203	775.3528	84	910.012	419.85	896.144	569.908	549.136	651.3493	2175.1	1463.85	699.51
2204	775.4291	71	613.557	388.74	557.245	276.241	330.372	455.4305	1497.82	1098.25	559.718
2205	775.8419	83	501.965	301.06	683.853	433.392	578.003	305.0119	1686.36	806.019	403.941
2206	776.5438	1172	918.743	473.76	547.206	396.41	594.534	748.3233	1003.45	984.718	535.967
2207	776.5445	1196	0	0	66.748	64.6523	0	0	0	0	0
2208	776.5446	1091	532.754	388.4	501.434	308.934	269.83	530.395	657.79	725.446	421.222

2209	776.5452	1120	340.428	189.4	350.243	141.6	142.53	265.0577	285.759	337.137	233.577
2210	776.546	1149	1239.88	752.66	1082.83	468.429	636.616	906.7434	1160.52	1488.69	721.265
2211	776.546	1039	1084.86	777.7	1113.75	557.856	349.28	820.5632	871.08	1486.16	693.83
2212	780.4184	72	615.19	615.96	570.397	294.692	465.686	335.5886	1466.76	778.765	865.409
2213	780.5656	116	679.805	710.59	642.717	1437.88	1885.48	998.6712	1918.49	1075.91	435.934
2214	780.5659	240	619.154	333.74	655.693	1007.39	717.876	702.5612	1837.08	872.019	190.289
2215	780.5665	139	1250.54	1000.4	1340.93	1894.56	1613.79	3407.348	4784.4	1502.26	581.677
2216	780.5666	30	971.152	694.66	1105.18	1750.46	1030.37	1180.078	4584.63	1100.62	363.561
2217	780.5666	7	589.311	470.98	598.941	1217.11	696.539	699.3812	4779.9	821.794	226.842
2218	780.5668	1072	642.746	714.65	595.763	1228.82	1632.58	867.1488	1649.89	650.475	396.856
2219	780.5669	1156	503.514	374.76	292.053	795.784	678.382	726.7881	1074.11	637.114	232.756
2220	780.5669	1051	1213.32	1130.6	1019.46	2230.75	1045.41	2495.842	4286.76	2210.94	863.917
2221	780.567	1102	607.098	480.74	321.109	1059.38	952.178	823.5482	1532.97	686.745	308.55
2222	780.5671	1191	213.173	264.6	96.135	290.277	698.855	537.0605	1343.28	0	162.482
2223	780.5671	190	500.223	315.21	562.209	1356.43	658.572	746.0887	1939.57	950.072	181.621
2224	780.5672	263	397.137	252.96	453.093	974.658	600.969	748.5772	1525.15	456.363	148.363
2225	780.5676	302	343.083	225.33	344.629	484.493	363.192	407.6992	1363.12	399.447	125.064
2226	780.5676	216	649.492	497.37	692.5	896.135	956.345	632.3895	1967.28	857.071	277.409
2227	780.5677	1167	525.447	386.85	277.219	837.527	738.781	806.9455	1173.66	681.071	259.484
2228	780.5677	167	737.234	482.58	875.79	1298.49	1025.83	974.0755	2539.63	1037.24	275.189
2229	780.5678	321	198.521	118.13	186.246	419.997	188.354	227.9989	659.597	267.728	56.84
2230	780.5682	1127	149.422	51.683	122.386	243.413	105.576	189.97	209.443	134.27	41.448
2231	780.5683	45	457.302	542.53	427.173	748.066	1014.65	746.3147	3522.74	934.326	266.57
2232	780.5686	282	275.557	185.11	303.864	474.794	345.331	450.2663	1339.36	421.197	68.7259
2233	780.742	78	660.395	386.88	488.269	397.939	890.526	275.5864	329.635	511.276	394.499
2234	780.7535	79	204.949	247.9	352.42	380.88	787.583	55.4	934.849	399.165	286.225
2235	781.4173	72	635.391	542.15	478.648	232.817	500.094	383.6234	1638.67	854.313	766.917
2236	781.5669	114	302.841	428.97	526.397	802.075	813.896	710.8512	1326.56	578.398	305.739
2237	781.5684	1	0	5.268	0	0	39.746	0	436.185	0	5.548
2238	781.5694	328	212.497	184.41	247.013	462.894	310.021	341.6389	871.237	401.973	60.6288
2239	781.5694	239	367.31	221.89	389.691	607.157	483.056	457.3447	1135.6	513.221	125.663
2240	781.5696	1059	920.817	882.75	860.527	1412.73	1686.29	1529.867	2646.5	1434.04	617.741
2241	781.57	265	271.733	199.06	317.152	489.534	438.672	482.3757	966.642	448.875	94.4178
2242	781.5706	191	483.371	296.05	522.432	732.53	614.267	534.7114	1286.53	622.281	139.691
2243	781.5706	167	471.01	319.58	519.504	713.132	661.974	640.7444	1518.15	668	196.111
2244	781.5707	41	390.39	360.72	388.246	620.066	585.292	614.3636	2225.9	662.348	193.122
2245	781.571	217	409.922	342.26	482.192	711.17	623.861	482.6191	1224.54	565.488	188.286
2246	781.5714	13	389.894	398.35	440.305	634.029	615.826	482.0808	2687.64	629.104	219.201
2247	781.5716	286	230.513	173.29	265.776	434.127	342.854	365.2656	950.791	331.246	82.04
2248	781.5717	140	757.102	553.64	759.546	1186.55	986.155	889.8221	3068.38	1076.15	358.562
2249	781.8518	83	398.885	213.14	539.299	282.763	255.402	263.655	1817.01	597.44	178.972
2250	782.4149	72	703.398	613.09	581.886	436.967	527.317	398.225	1536.59	861.361	765.919
2251	782.568	37	201.167	225.41	160.994	316.241	305.883	294.7495	1064	335.738	100.07
2252	782.5706	13	178.156	232.45	193.673	290.496	321.159	257.085	1426.36	329.425	123.325
2253	782.5709	3	44.0233	14.825	44.4562	87.8389	23.1235	33.925	201.966	24.4807	2.3608
2254	782.8308	84	5216.15	2081.2	4030.73	3232.7	2543.68	2811.851	10752.2	5963.77	2332.3
2255	783.4127	72	681.6	386.9	415.03	291.03	284.058	314.4405	1414.67	745.799	545.249
2256	783.8277	84	838.243	428.13	998.767	785.09	826.249	534.4182	3571.61	1144.79	426.045
2257	784.4132	72	415.406	361.53	380.837	182.993	280.63	214.797	829.369	656.47	458.801
2258	784.8286	84	2475.95	1008.2	1400.74	1386.7	1235.28	1207.51	4499.06	2495.59	960.282
2259	786.8229	82	976.368	450.31	707.732	647.682	869.34	491.5925	1740.78	943.01	608.562
2260	787.5144	140	493.798	406.81	499.412	593.98	616.546	557.1148	14755.2	647.321	358.547
2261	787.8452	89	1470.45	322.06	852.735	694.762	427.689	767.7526	3964.25	1641.17	474.027
2262	788.4049	71	491.231	433.28	495.88	293.7	391.576	279.7988	1012	723.925	554.558
2263	788.5165	126	255.564	222.09	309.626	309.78	263.468	289.9671	3129.19	272.981	169.932
2264	788.5187	142	260.95	196.62	274.24	197.536	230.415	259.3584	4626.61	272.656	169.758
2265	788.7753	79	1163.19	534.81	1359.44	1038.03	764.319	669.9949	1537.27	1132.92	546.936
2266	788.8608	84	168.937	13.392	155.446	295.427	299.681	202.644	1451.64	614.434	85.3358
2267	789.407	72	407.66	416.57	497.33	166.944	366.129	273.0828	846.667	659.95	597.95
2268	789.5132	142	234.843	152.47	204.794	242.304	208.705	226.1336	4767.56	288.27	161.85
2269	790.4268	70	1479.3	1472.9	962.935	918.086	1237.24	853.0545	3563.88	1698.72	1623.43
2270	790.5156	142	401.735	238.68	442.856	271.927	143.147	386.8254	2121.74	391.116	216.911
2271	790.5187	126	348.502	243.27	424.249	288.462	135.24	294.6718	1118.62	288.201	174.291
2272	790.7753	80	1100.87	530.27	941.429	1027.55	948.205	562.2831	1627.42	818.276	468.06
2273	792.4256	70	3083.71	2836	1814.46	1862.67	2414.93	1775.764	8406.02	4239.55	3230.27

2274	792.8601	86	17102.6	6204.2	12341.5	9341.46	8043.76	9604.524	44400	24923.8	7322.68
2275	793.36	87	550.485	220.82	497.464	550.674	386.757	417.7122	3495.82	1657.13	306.346
2276	793.5155	41	79.4694	95.001	42.5615	67.555	57.2706	56.991	3126.53	64.9995	30.5345
2277	793.5173	13	32.9387	61.302	39.027	45.9842	34.4908	33.42843	12243.1	76.7866	24.5412
2278	793.5173	1	0	2.3566	0	0	0	0	2972.58	0	6.46631
2279	793.8614	86	3198.63	1247.7	2422.64	1558.95	1606.49	1894.41	8837.53	4738.99	1630.74
2280	794.423	70	3903.08	3628.2	2433.79	2179.23	3328.67	2361.952	9482.07	5649.06	4165.52
2281	794.5179	40	89.388	87.772	53.1974	71.669	46.9917	45.83543	1872.61	47.5447	29.2386
2282	794.5206	7	40.999	51.115	35.296	38.8804	42.0755	31.7008	5959.33	36.6983	40.8837
2283	794.8595	87	1582.1	796.4	1366.71	905.846	917.946	1019.113	4702.71	2897.96	949.958
2284	795.5142	2	0	7.9534	0	0	4.42607	0	547.31	0	1.76354
2285	795.5222	12	69.9947	68.308	79.828	86.6625	86.2271	70.37565	2137.43	96.9936	13.0887
2286	795.5937	116	1277.25	1049.5	1502.17	1692.3	1744.11	1697.528	3533.96	1847.41	1098.87
2287	795.5939	286	239.275	122.47	287.753	506.653	229.821	327.5127	706.805	378.697	87.6097
2288	795.5943	192	939.884	595.98	1143.77	1217.35	1077.08	932.3211	2101.27	1217.4	429.742
2289	795.5946	47	1457.65	1243.8	1396.34	1841.49	2153.05	1749.918	2649.15	1757.63	807.144
2290	795.5948	1071	731.2	1023.2	797.22	1236.86	1530	768.6656	1491.91	1247.74	896.467
2291	795.5948	1053	2646.93	2049.5	2761.86	3732.48	3014.54	4179.2	6020.01	2320.63	2883.13
2292	795.5949	1193	527.484	277.4	241.828	1187.96	554.405	1025.73	1307.18	0	425.489
2293	795.5949	238	693.929	400.96	838.902	847.506	677.174	666.7271	1561.27	787.788	306.682
2294	795.5951	1101	594.44	627.29	326.333	971.009	769.775	548.432	383.36	919.27	679.643
2295	795.5952	161	1225.24	449.95	1234.32	1206.87	803.9	1016.313	2398.31	1289.39	391.926
2296	795.5952	139	2168.33	1360.1	2697.62	4255.8	2049.4	1989.785	6724.46	3095.98	1244.38
2297	795.5956	264	408.642	293.99	572.804	823.834	574.372	710.9158	1227.62	573.627	236.508
2298	795.5956	17	531.293	716.17	649.268	701.225	883.159	625.95	3277.84	995.902	433.913
2299	795.5975	1171	398.675	459.32	252.928	615.791	893.723	690.1702	1108.4	649.212	558.5
2300	795.8353	84	1414.37	338.05	1311.3	619.415	753.371	768.5754	3463.58	1884.67	250.315
2301	796.4196	70	3334.52	3356	2559.87	1901.08	3253.87	2052.493	8526.79	5148.97	3863.06
2302	796.5951	243	253.493	144.13	244.728	258.62	283.502	313.0298	573.03	303.811	121.044
2303	796.596	157	710.773	276.52	744.813	753.711	429.134	588.2237	931.551	542.945	237.457
2304	796.5968	138	1663.33	1107.6	2105.45	2667.59	1564.1	1346.396	7420.29	1941.3	977.986
2305	796.5969	1055	1627.45	1393.4	1850.39	2762.61	1915.55	2551.062	3412.79	2096.02	1827.28
2306	796.5969	187	646.467	432.37	785.571	845.973	678.537	699.2457	1279.5	733.047	364.149
2307	796.5975	264	194.896	126.26	245.135	236.897	250.051	332.1412	575.339	331.626	92.5673
2308	796.5977	1093	461.843	400.34	331.598	697.737	776.284	699.7413	995.754	513.765	415.327
2309	796.5986	43	459.05	396.97	430.066	674.868	611.884	662.5943	1848.64	748.221	283.454
2310	796.6004	1	0	28.673	0	0	34.9359	0	410.04	0	15.6047
2311	796.6016	17	414.591	485.97	463.656	495.52	583.079	481.8848	2115.42	657.86	291.353
2312	796.8389	85	1244.62	459.7	966.565	798.686	829.745	698.2041	3001.94	1704.96	564.891
2313	797.4192	72	335.475	280.9	319.358	129.495	110.172	174.5411	920	511.627	332.469
2314	797.5427	139	354.716	282.48	289.667	417.705	448.229	303.7127	41064.9	364.453	201.004
2315	797.5888	116	863.209	655.81	995.684	1242.96	998.471	1109.567	1918.25	1158.17	762.461
2316	797.5925	1093	417.297	420.38	303.543	607.876	703.822	635.4738	859.569	519.592	414.342
2317	797.5926	207	429.942	312.99	455.138	552.621	485.678	433.8112	859.795	506.451	292.509
2318	797.5927	264	296.981	219.61	402.453	434.654	360.512	442.3299	762.452	449.714	152.717
2319	797.5934	138	1289.24	774.56	1648.49	2720.08	1119.55	1229.721	1585.9	2893.48	702.595
2320	797.5935	186	600.876	364.79	766.585	846.992	538.612	487.385	1279.98	652.092	288.364
2321	797.5938	1057	1527.65	1306	1788.99	2542.8	1788.24	2548.087	3215.56	808.689	1481.41
2322	797.5942	1110	155.631	135.92	99.3225	150.564	215.552	284.751	356.339	209.619	159.218
2323	797.5945	243	485.218	298.84	542.998	563.311	481.983	476.5659	944.204	614.472	226.356
2324	797.5951	164	805.278	280.11	847.404	728.167	536.559	680.2331	1384.74	570.256	283.694
2325	797.5956	43	607.683	405.78	496.125	745.811	579.945	699.4359	1718.61	726.796	292.04
2326	797.5976	17	382.902	468.67	462.096	495.99	576.581	424.9651	2061.24	661.506	304.56
2327	797.9213	88	1439.31	408.91	1055.65	455.939	353.368	417.2504	1853.07	1143.43	486.661
2328	798.4168	70	2300.86	2219	1469.61	1204.57	1774.87	1417.662	5716.07	3286.57	2439
2329	798.5469	139	235.931	211.27	217.891	301.192	314.05	263.8312	20640.2	265.387	152.275
2330	798.5928	1076	0	0	20.48	0	0	21.53333	7.92	6.26667	10.08
2331	798.5931	146	249.373	288.17	306.342	339.068	435.679	215.3719	583.478	379.889	258.305
2332	798.5936	1053	867.632	869.03	918.416	1052.7	1239.05	1300.053	1612.28	1129.28	988.62
2333	798.804	82	3701.2	1529.6	3313.92	2692.06	2546.04	2209.479	7104.26	3381.47	1848.53
2334	799.4217	72	278.318	211.47	271.69	126.825	115.565	114.7957	959.229	416.274	308.135
2335	799.5499	139	757.906	517.63	867.535	782.234	847.853	779.0625	5766.91	1004.72	521.619
2336	799.8064	82	1222.89	444.84	931.571	742.67	818.655	639.5625	2203.07	880.438	552.034
2337	799.869	87	873.098	197.24	598	426.927	398.537	492.6357	2856.7	1330.43	315.531
2338	800.3718	87	55.0154	23.038	111.781	150.753	71.195	38.522	1336.43	336.429	25.4258

2339	800.4127	70	1605.96	1337.2	922.223	905.608	1124.55	825.6907	3761.63	2266.28	1407.05
2340	800.5354	2	20.7345	14.681	32.8938	327.889	87.6649	46.99258	209.535	26.0313	4.75822
2341	800.5426	118	85.306	108.82	125.978	1681.2	351.582	205.4914	544.306	201.859	47.1852
2342	800.5469	335	67.1012	42.814	65.6429	773.32	172.759	103.6216	173.999	110.777	17.5746
2343	800.5469	93	102.052	179.95	154.693	1517.22	564.647	246.7083	720.223	253.258	169.912
2344	800.5476	147	210.636	125.33	204.265	1460.66	295.88	211.0197	1288.39	243.685	101.166
2345	800.8017	82	2020.39	800.41	1799.63	1298.95	1188.05	887.1985	2910.38	2163.25	1109.88
2346	800.8472	85	1967.91	962.34	2256.72	1580.71	1035.97	1218.417	6916.6	3139.23	1141.61
2347	801.3502	87	1014.25	513.38	866.422	569.742	956.395	618.425	2941.08	1786.2	574.362
2348	801.4067	71	660.965	497.25	427.085	343.212	452.729	415.4793	1050.84	754.053	621.068
2349	801.8415	87	464.874	285.14	511.527	321.244	634.167	211.2463	2161.75	763.018	259.765
2350	802.4088	71	885.909	789.25	748.714	552.699	741.27	599.5943	2600.77	1543.26	1146.24
2351	802.8382	84	800.667	259.51	823.408	511.008	425.673	384.7846	1235.7	1028.26	208.13
2352	803.4072	72	670.686	559.09	469.754	332.727	545.727	346.6263	1511.91	1046.54	847.146
2353	803.4492	71	761.908	623.42	523.36	467.039	510.19	439.7788	2168.52	1074.55	929.729
2354	803.8185	83	679.218	364.51	766.898	591.332	554.456	356.6325	2261.62	865.477	264.115
2355	803.8783	85	1157.05	572.27	1067.06	688.231	697.916	653.2602	3698.71	1708.96	676.482
2356	804.3265	82	227.681	88.187	392.519	178.229	105.107	310.1643	859.496	300.946	75.8535
2357	804.4057	71	1281.31	865.9	821.471	645.04	934.381	626.8539	2415.15	1546.93	1262.7
2358	804.4451	67	1462.14	1185.9	1019.81	732.3	1036.08	766.9667	3553.13	1669.41	1418.33
2359	804.5741	1139	1517.85	1179.3	1217.08	1271.03	808.744	1301.682	766.476	1230.38	1156.23
2360	804.5744	1151	662.35	443.07	466.171	576.84	286.867	600.2813	312.005	531.828	434.045
2361	804.5751	1113	1327.05	1007.5	1028.74	1237.45	798.954	1244.302	811.82	1118.83	1053.96
2362	804.5752	1168	1245.88	547.25	447.7	768.233	508.125	713.5849	457.295	558.329	597.429
2363	804.5752	1125	459.195	258.39	389.419	382.25	210.339	392.5339	223.166	337.617	312.067
2364	804.5752	1037	2369.98	1464.4	1288.49	1306.42	832.495	1368.961	838.904	1236.74	1318.02
2365	804.5758	1188	920.773	574.65	496	1052.15	0	0	0	0	686.122
2366	804.5762	1094	493.428	398.68	335.838	487.072	308.731	516.2533	339.546	446.152	372.88
2367	804.5764	1083	444.504	521.47	414.324	466.061	395.245	525.2445	413.073	439.326	476.552
2368	804.5771	1200	219.927	98.784	101.649	211.375	0	0	0	0	0
2369	804.58	1	0	746.39	0	0	661.36	0	1454.65	0	456.496
2370	804.7547	79	607.01	371.07	568.935	560.281	918.239	406.2211	991.939	638.208	279.808
2371	804.82	83	924.721	443.36	895.027	596.986	1117.63	361.5756	1870.73	912.605	339.597
2372	805.3071	82	86.3693	14.45	212.902	127.3	155.939	99.68	769.557	125.76	31.2867
2373	805.4282	67	513.772	208.76	307.309	252.072	213.305	257.8631	845.88	417.081	268.13
2374	805.6229	264	433.721	402.93	526.725	670.35	620.906	674.0295	894.834	722.949	274.344
2375	805.6232	1062	1577.89	1573.2	1513.89	2030.36	4663.06	1605.263	3281.72	1802.68	884.758
2376	805.6234	211	1374.77	678.73	1173.36	1684.5	1128.51	1254.229	4328.88	937.424	554.498
2377	805.6237	1096	596.8	629.24	436.872	1213.64	1059.05	750.8264	951.145	661.367	586.054
2378	805.6238	180	1003.09	690.46	1187.53	1438.22	1453.89	1063.928	1599.14	1039.21	592.594
2379	805.624	100	4762.24	3309.9	2730.79	8387.53	5818.64	4705.493	8390.47	6875.14	3854.29
2380	805.6241	148	1452.31	865.95	1474.77	1369.6	1589.62	1586.309	5498.64	1347.34	987.553
2381	805.6242	1191	434.588	358.61	217.131	700.77	883.071	867.783	1440.42	0	445.136
2382	805.6252	14	845.163	808.25	922.433	969.955	1064.89	988.31	2352.26	1129.88	514.88
2383	805.8204	82	261.58	86.549	255.673	257.209	169.108	202.8218	1086.47	318.42	141.52
2384	806.4024	71	2356.2	2014.1	1717.54	1133.81	1894.54	1211.023	5485.18	3175.03	2569.62
2385	806.6253	136	1028.04	691.32	1111.54	1065.62	994.152	971.0545	2983.46	1098.06	465.862
2386	806.6257	100	2532.35	2309.4	3195.19	4849.93	4107.44	3235.777	5443.96	2861.31	2321.72
2387	806.6258	116	4433.66	3396.6	4549.1	5200.27	5066.8	4129.092	8710.03	4346.86	3113.44
2388	806.6259	175	355.253	340.44	406.042	454.138	740.775	315.9326	588.132	423.275	302.328
2389	806.626	158	860.076	482.62	849.87	1027.67	711.948	735.9092	1352.48	884.67	379.371
2390	806.6261	211	1035.7	414.09	808.096	1135.45	663.247	669.3857	2940.81	575.85	354.904
2391	806.6266	1191	331.296	244.71	145.231	448.652	606.029	601.4806	1003.71	0	319.16
2392	806.6275	1164	320.301	260.17	192.822	398.722	467.024	482.8741	565.871	369.23	303.887
2393	806.6281	192	527.297	339.83	624.052	677.615	597.133	763.5467	1123.12	683.896	262.727
2394	806.6295	13	537.312	539.89	612.015	639.357	687.857	594.2344	1671.06	763.825	323.944
2395	806.8746	86	2657.75	1146.9	1610.81	1432.57	1053.45	1684.676	6609.63	3648.77	1026.93
2396	807.6269	116	2177.43	1740.9	2433.22	2793.12	2719.7	2174.709	4669.69	2422.01	1630.76
2397	807.8608	86	842.195	402.04	609.687	492.773	882.167	411.1038	3459.67	1497.15	424.932
2398	808.3997	71	4433.96	3999.1	3716.45	2342.95	3603.99	2446.877	9649.1	5639.97	4943.35
2399	808.8341	85	10170.3	4053.6	9517.05	5505.55	5272.25	6173.901	22394.6	11268.4	4788.84
2400	809.336	86	226.592	223.72	296.033	463.833	327.129	91.33529	2041.07	589.257	150.138
2401	809.4024	72	720.728	634.05	691.778	450.479	556.723	295.395	1780.07	861.604	719.584
2402	809.4982	1043	46.4847	37.433	42.8346	111.184	109.32	104.6154	5685.1	5152.64	4168.43
2403	809.8359	84	2411.01	932.9	2119.14	1546.5	1345.87	1297.538	5818.33	2763.46	1224.98

2404	809.9353	88	1392.59	447.27	1218.09	595.653	739.416	695.6654	1726.27	1272.21	755.654
2405	810.3965	71	6068.62	5682.4	5002.13	2872.19	4716.1	3105.258	13564.5	7751.72	6690.33
2406	810.5019	1044	255.783	245.75	143.585	339.789	102.34	281.6748	2713.13	2672.82	2275.21
2407	810.8325	85	1972.05	987.49	1866.35	1135.83	991.229	1178.219	5066.27	2281.5	940.414
2408	810.9262	87	1326.73	108.61	221.499	581.202	348.666	105.5896	801.818	621.731	269.37
2409	811.3955	70	838.19	527.13	603.95	406.98	285.209	337.7918	1695.69	739.834	652.975
2410	811.4978	1044	145.793	132.35	78.6703	177.289	90.189	172.811	2633.68	2358.95	1981.3
2411	811.8174	83	942.688	311.45	835.959	485.894	635.243	495.2755	2069.11	806.36	337.219
2412	812.3942	71	5379.89	5149	4519.39	2960.93	4369.37	2775.109	11864.6	7067.64	6333.19
2413	812.8155	83	995.483	511.25	1025.48	701.652	511.273	581.8289	2290.15	1061.01	511.631
2414	813.3923	74	318.468	364.72	393.105	209.791	321.558	238.7189	1077.33	652.719	532.779
2415	813.4893	89	42.0604	70.319	85.8821	57.2483	90.9704	58.0533	7141.49	137.714	124.456
2416	813.5297	193	47.6937	17.105	43.6505	52.9271	21.0343	35.40444	2094.92	39.1715	2.75
2417	813.5299	172	40.0937	27.519	41.7025	48.6827	40.6196	14.59172	857.324	39.6489	8.94157
2418	813.5304	144	88.037	52.063	90.6027	108.409	66.3397	85.45751	5028.32	113.266	34.7153
2419	814.3907	71	3359.08	3353.1	2865.4	1960.37	2891.23	1992.174	7868.41	4557.4	3757.29
2420	814.4954	92	263.929	358.89	306.549	337.484	468.674	401.3476	4619.55	481.926	232.224
2421	814.5333	140	472.352	373.67	455.686	586.02	598.079	482.4359	12143	505.478	331.262
2422	814.7777	82	1558.25	723.32	1868.87	1320.13	1044.02	833.4757	3258.35	1282.54	737.759
2423	815.4993	1045	179.721	131.24	193.251	231.466	227.9	280.7563	3290.02	2840.08	2619.86
2424	815.504	1088	155.576	136.28	171.967	280.305	281.654	231.4219	884.806	299.52	208.68
2425	815.5337	142	374.914	337.37	361.807	502.528	523.701	512.5875	6766.78	467.788	290.515
2426	815.8387	84	784.431	367.34	833.663	692.802	762.357	481.1502	1358.1	958.036	348.667
2427	816.3877	71	2221.31	2029.7	1571.02	938.543	1499.24	968.1698	3859.58	2473.51	2340.82
2428	816.5018	1047	200.403	158.24	212.018	247.021	296.645	286.0005	1850.24	1565.32	1217.51
2429	816.5369	141	722.125	594.8	708.761	863.041	919.916	779.8108	2949.64	752.665	500.683
2430	816.7763	80	1039.53	498.75	1077.31	640.019	965.177	425.8333	1680.76	1122.1	515.374
2431	816.823	84	1332.57	509.21	1367.51	785.557	1029.67	737.5438	2478.08	1124.98	532.473
2432	817.327	84	325.342	95.436	331.446	123.217	514.975	164.0526	1461.31	471.384	114.411
2433	817.8248	83	506.91	158.53	514.092	385.546	302.534	310.0657	1514.41	736.362	256.239
2434	818.3829	71	1132.57	1215	1018.74	613.215	857.95	583.8218	2569.5	1504.7	1406.64
2435	819.5279	1042	208.045	158.18	308.869	358.086	352.601	424.9097	16526.9	10723	12858
2436	819.5291	70	148.194	189.67	91.8812	144.735	201.622	69.38395	3002.67	289.654	221.844
2437	819.5327	41	41.0025	46.964	96.226	113.618	67.0533	39.254	11104	156.014	15.0871
2438	819.5329	18	52.0169	14.46	170.712	121.131	39.2094	38.72859	6332.59	141.975	31.944
2439	819.8547	83	849.226	338.36	906.42	566.739	583.176	640.4922	2014	1276.45	478.18
2440	820.3812	71	799.155	687.33	835.553	464	558.48	442.2609	1633.14	998.791	937.572
2441	820.5314	1042	114.912	70.626	123.807	178.934	202.978	192.0175	8129.21	8046.64	7000
2442	820.5352	41	28.1819	28.237	99.4686	49.8424	34.9592	14.1939	5702.96	114.634	8.29458
2443	820.5358	18	34.4115	26.941	82.4233	52.1158	30.532	23.46298	3222.36	78.9154	5.29292
2444	821.3964	72	543.522	444.76	470.998	299.436	305.215	241.344	1275.83	600.752	564.033
2445	821.5338	1042	79.6831	77.461	76.769	144.539	94.896	121.22	2647.05	2336.05	2070.95
2446	821.5365	41	27.0196	40.715	53.6645	164.323	66.4339	46.43711	1978.88	67.0962	22.0527
2447	821.5377	18	42.0585	68.251	62.238	654.144	71.2413	70.38895	1360.21	43.2156	38.8148
2448	821.8379	88	734.032	262.14	664.941	455.922	645.442	484.4349	3221.14	1489.06	200.578
2449	822.3311	83	169.64	122.05	222.164	303.616	126.488	83.9712	968.501	569.315	65.4134
2450	822.3771	72	1518.49	1527.7	1270.56	781.542	1154.89	856.1257	2932.81	1614.85	1489.5
2451	822.5354	2	0	11.86	0	0	0	0	997.253	0	3.5376
2452	822.613	1103	848.254	632.33	752.863	1809.08	728.297	1186.843	1495.24	805.755	705.675
2453	822.6136	220	1210.44	1050.5	1322.24	1572.06	1486.11	1185.747	3020.32	1188.37	716.668
2454	822.6137	1051	2659.48	2132.8	2536.42	3392.27	3673.02	3945.909	5710.66	3893.3	2984.29
2455	822.6139	1069	1463.53	4043.2	1266.61	2118.91	5218.2	4463.688	6808.89	5539.6	4824.26
2456	822.6139	173	1759.31	778.15	1818.44	2204.09	1637.2	1485.427	3697	1852.99	621.504
2457	822.614	1180	1420.1	689.62	624.061	2145.34	1406.07	1785.716	1756.37	1084.76	1080.66
2458	822.614	266	531.923	227.3	729.105	1316.04	1005.04	1254.885	1838.07	785.51	309.862
2459	822.614	198	1412.9	598.51	804.018	974.345	1173.53	1576.99	3739.61	1970.29	526.294
2460	822.6141	118	1397.93	1904.7	1668.1	1588.14	2746.13	1859.641	3287.86	1770.27	1934.94
2461	822.6142	1196	661.631	592.06	363.545	1028.36	1398.21	1505.541	2199.88	1268.98	868.989
2462	822.6142	1130	412.361	269.84	359.903	647.119	384.035	479.8683	481.77	420.012	255.534
2463	822.6142	287	520.442	298.5	798.851	756.413	632.138	755.43	1809.08	928.449	265.594
2464	822.6143	41	1137.54	1879.9	2450.43	1572.49	1994.34	2056.621	5406.81	1828.53	704.21
2465	822.6144	240	1154.55	609.85	1550.25	1586.47	1114.65	1215.074	3079.21	1634.66	509.031
2466	822.6145	1152	608.574	283.45	424.556	759.447	587.278	1280.132	565.079	392.109	274.083
2467	822.6145	141	2516.69	1039.8	2875.48	3299.05	2276.95	2296.237	7515.45	3025.52	1101.01
2468	822.6147	13	1151.81	1421.1	1407.62	1848.26	1903.1	1199.353	7340.19	1747.56	823.337

2469	822.6153	1169	515.541	621.6	322.354	733.736	1415	739.2151	1482.71	817.892	823.879
2470	822.8481	85	1924.36	746.04	1565.17	1237.69	1079.02	1129.708	4082.16	2108.71	990.147
2471	823.4017	72	622.44	386.61	288.939	214.452	253.247	307.4379	1449.36	648.407	409.301
2472	823.5388	1	0	0	77.14	124.12	0	0	110.376	17.751	0
2473	823.5591	193	2310.57	1790.3	2789.92	3229.89	2786.72	2273.94	11420.2	3077.8	1293.73
2474	823.5592	141	1625.74	1226	1893.07	2054.2	1658.46	1589.773	13935.4	2012.22	1149.92
2475	823.6146	2	0	108.27	595.513	1533	123.664	0	585.093	0	82.0096
2476	823.6159	1050	1750.09	1411.7	1625.71	2249.82	2130.84	3898.923	3735.39	1486.94	1213.64
2477	823.616	300	354.487	224.12	388.517	547.979	310.684	384.4577	1227.85	399.645	178.935
2478	823.6161	167	951.793	621.43	1215.92	1369.37	1081.5	1055.816	2576.07	1209.11	504.512
2479	823.6165	186	818.303	323.35	956.304	1399.29	551.636	882.3401	2727.86	1118.47	256.192
2480	823.6167	1192	723.1	487.61	355.924	1389.83	587.939	1207.955	1459.94	0	703.815
2481	823.6167	278	377.27	232.05	416.972	674.499	379.887	557.6502	1471.22	456.036	186.792
2482	823.6168	135	3538.89	1492.5	4070.05	2101.36	1991.58	1459.705	4876.97	2023.07	1373.39
2483	823.6168	114	912.63	866.7	1068.16	966.483	2154.99	1117.493	2333.2	1264.74	826.07
2484	823.617	1072	511.014	986	928.974	1532.05	3410.5	1107.132	6174.87	832.338	1217.43
2485	823.6171	1169	480.24	491	308.477	679.907	943.485	746.7671	1212.67	712.55	615.234
2486	823.6172	240	768.32	433.75	953.376	1073.92	805.119	843.178	1981.97	1063.52	378.675
2487	823.6172	206	739.57	616.29	888.552	1241.26	933.856	699.934	3393.86	1191.29	478.768
2488	823.6174	1101	597.093	566.25	512.94	1130.79	490.154	849.9195	983.914	976.582	855.62
2489	823.6176	257	640.423	484.44	919.983	867.659	845.785	866.2133	1958.85	524.732	382.905
2490	823.6176	43	739.354	703.88	1525.9	619.653	1085.82	956.6774	3448.84	1158.8	467.034
2491	823.62	30	858.686	713.84	1050.79	1309.15	939.352	684.2876	3118.55	740.466	478.208
2492	823.6216	13	823.022	800.33	992.636	1072.34	1033.81	834.0072	4702.82	1124.64	535.135
2493	823.853	83	482.263	327.97	540.236	296.815	461.095	286.9531	1268.18	852.252	332.058
2494	824.3737	71	2538.66	2428.2	2645.81	1104.87	1996.62	1215.518	5708.43	2849.74	2921.17
2495	824.5604	141	891.256	611.36	961.743	1136.59	934.89	938.5961	7439.14	1060.94	587.692
2496	824.5617	191	1136.3	892.31	1354.2	1496.31	1365.52	1257.437	6307.41	1613.76	710.979
2497	824.6122	2	0	63.992	432.107	737.03	74.223	0	433.788	0	35.343
2498	824.6151	140	889.897	586.57	923.792	1090.27	907.897	905.8373	2369.01	1030.25	583.362
2499	824.6159	193	948.585	634.93	1084.14	1230.47	1000.56	912.4126	2072.18	1197.71	543.923
2500	824.6159	40	352.642	273.08	315.971	319.726	337.045	514.0653	1801.75	500.577	190.603
2501	824.6172	114	400.31	481.55	520.804	772.812	881.504	608.1569	1245.5	636.876	496.57
2502	824.6174	167	540.83	381.31	629.558	569.579	536.296	564.9185	1283.12	640.033	288.73
2503	824.6193	52	302.306	462.27	294.58	388.279	524.81	153.3667	834.642	305.282	325.938
2504	824.8085	84	3757.74	1585.4	3817.08	2261.47	2570.45	1937.438	7347.07	3933.31	1796.48
2505	824.9304	90	368.877	85.745	266.18	491.4	354.493	211.2212	1136.1	529.526	408.377
2506	825.3987	74	352.45	364.38	353.932	201.333	340.075	258.7818	1134.45	532.598	424.773
2507	825.5486	402	35.344	19.775	38.9449	72.3249	54.2537	41.35371	1137.22	55.277	11.1999
2508	825.5497	443	44.7318	3.168	12.8495	84.9231	42.1846	32.43	1016.22	32.1858	8.32
2509	825.55	414	44.4387	21.307	34.8929	91.4632	55.8069	41.38413	1131.91	51.4653	11.0312
2510	825.5662	141	406.925	307.77	480.862	627.732	423.83	435.8642	2432.96	464.9	310.736
2511	825.8099	83	1360.61	462.29	1179.2	882.605	638.207	781.1786	2136.19	942.791	651.864
2512	825.8218	84	952.326	474.62	1151.33	757.196	651.8	517.8224	2653.93	1036.18	667.71
2513	826.3703	71	3429.02	3366	3207.26	1404.58	2571.37	1498.593	6721.3	3720.8	3846.01
2514	826.5615	1	54.4	40.891	47.8157	471.96	137.675	81.94242	160.741	43.9276	34.903
2515	826.5616	225	165.775	105.75	147.977	1655.36	281.545	198.0258	353.464	186.232	63.4494
2516	826.5619	113	582.843	560.66	471.646	4665.79	1340.34	731.2521	1864.02	757.791	478.059
2517	826.5645	335	56.5181	33.986	58.7438	932.048	155.031	141.6198	426.552	93.0561	21.7292
2518	826.803	83	1344.52	462.78	1300.75	713.351	860.208	638.88	2022.37	1118.28	466.735
2519	826.8546	86	1879.47	580.66	1445.85	1042.32	1104.11	1044.231	8231.04	3316.22	712.909
2520	827.3547	86	571.438	318.9	718.046	517.44	542.068	285.6977	3247.42	1546.01	515.921
2521	827.8549	89	915.057	378.78	927.198	668.542	667.798	490.7168	1825.89	1324.59	548.627
2522	828.3679	71	3119.01	3114	2953.8	1369.59	2326.79	1426.971	6683.72	3478.64	3745.04
2523	828.5039	71	399.3	277.11	262.841	381.524	511.797	384.9191	1391.37	820.978	415.946
2524	828.785	80	436.182	152.72	278.388	300.906	565.02	270.4683	762.603	522.72	217.201
2525	828.8488	88	320.529	98.695	299.814	391.114	341.858	230.0522	1477.2	880.593	172.901
2526	829.3938	71	458.645	372.68	337.822	209.646	466.581	300.006	1319.93	600.572	576.26
2527	829.8277	85	1028.96	335.14	867.853	625.022	692.167	809.8976	3368.92	1527.15	287.905
2528	830.3284	86	335.459	204.92	504.866	169.808	334.565	270.86	1430.65	792.977	203.87
2529	830.3664	71	2186.59	2024.4	1879.98	849.197	1941.73	1073.079	4867.62	2601.54	2504.22
2530	830.4557	65	2182.32	2028.9	1818.09	850.022	1749.68	1135.315	4980.64	2689.12	2522.44
2531	830.491	71	447.775	330.58	421.107	466.299	611.425	508.6228	1364.44	1057.34	572.533
2532	830.7488	79	545.726	336.67	544.32	426.288	651	400.1867	930.719	538.65	194.764
2533	830.8282	86	724.5	576	608.725	489.464	1000.35	459.6597	2226.51	1199.25	372.501

2534	831.3892	72	570.875	511.72	485.656	300.58	368.932	353.0841	1756.25	896.849	655.458
2535	831.5038	43	33.3771	42.411	27.7575	315.865	64.1431	61.21256	1901	60.8059	11.8745
2536	831.5059	1	0	2.6412	0	0	1.17508	0	803.846	0	1.17333
2537	831.5075	13	34.8965	37.718	45.8572	83.6449	70.4142	45.73902	3208.71	62.8432	13.6602
2538	832.3635	72	1376.91	1400.1	1070.98	687.469	1128.22	781.2496	3689.82	1687.72	1321.07
2539	832.3738	70	1391.55	1427.8	1069.85	631.061	1117.97	707.3979	2962.74	1558.49	1391.09
2540	832.4937	71	404.861	406.33	411.262	335.458	658.411	411.039	1762.92	840.416	545.958
2541	832.5094	2	19.0126	8.5996	25.9198	12.591	6.8195	14.17714	581.7	16.1151	7.07446
2542	832.8059	82	404.504	164.73	524.257	363.56	898.785	234.675	1450.33	516.072	210.789
2543	833.388	73	607.768	537.03	404.7	295.135	295.66	389.5118	1867.2	890.939	623.727
2544	833.5187	1062	44.3831	133.25	75.9733	155.125	153.036	161.3733	1465.01	90.4828	120.698
2545	833.5209	1043	101.417	161.02	85.2996	154.57	137.674	123	2127.33	383.568	353.173
2546	833.7995	82	402.343	241.31	422.232	226.455	301.839	222.345	1196.5	336.549	152.003
2547	833.8603	87	516.256	242.16	513.358	414.75	530.335	276.0102	2509.99	1081.6	310.122
2548	834.3784	74	843.11	733.85	736.625	451.365	659.126	477.5841	2055.16	1313.89	886.996
2549	834.4873	72	390.45	301.99	382.421	374.166	422.929	398.3381	837.52	636.768	441.369
2550	834.8421	85	2086.54	572.09	1772.08	1115.55	1912.27	1234.6	7345.75	3141.99	648.993
2551	834.8476	84	1941.17	570.29	1821.41	1231.77	2042.02	1172.453	6140.52	2842.68	660.843
2552	835.3398	86	583.198	260.86	663.636	623.578	413.442	495.3385	3004.11	1086.75	209.532
2553	835.383	74	884.995	341.82	728.174	457.578	458.416	376.7801	1883.24	991.515	453.849
2554	835.8336	84	796.367	311.31	758.365	396.855	508.174	519.4095	2501.18	1217.71	407.769
2555	835.9014	88	838.808	295.19	772.604	615.632	415.352	549.2698	1996.89	1161.35	495.467
2556	836.5166	1041	327.353	319.72	291.881	467.14	154.286	359.5694	3636.75	2894.03	2268
2557	836.8296	86	406.216	267.25	495.095	450.386	655.08	282.6703	2089.6	764.429	184.551
2558	837.3814	72	589.218	431.81	414.105	364.278	315.412	219.1337	1569.41	806.6	546.936
2559	837.5193	1041	180.747	165.26	165.564	257.437	92.8068	199.7608	1781.35	1909.48	1201.02
2560	837.8157	83	951.284	364.31	778.901	536.037	1055.77	522.8776	2561	1048.61	303.905
2561	838.368	72	616.819	518.08	599.638	345.909	558.48	382.3916	1635.26	805.68	645.786
2562	838.8192	83	1267.53	396.6	1166.04	505.67	533.691	523.6757	1878.89	978.462	460.483
2563	839.3747	72	704.852	656.37	485.64	383.275	494.966	319.0287	1632.6	797.884	801.935
2564	840.3531	72	1138.13	1152	1173.16	456.459	999.347	645.8223	3040.39	1449.34	1315.73
2565	840.3763	74	724.88	377.98	531.769	192.42	434.501	296.0648	1356	1052.26	604.524
2566	840.5479	191	67.7049	25.026	66.1978	65.6298	63.2026	42.56512	1871.36	93.1193	15.1561
2567	840.5512	141	113.946	72.825	151.9	90.7959	99.0998	110.8566	3194.79	142.716	70.873
2568	840.7877	82	1436.97	730.18	1662.32	1012.32	1203.49	670.0364	3455.96	1324.04	801.36
2569	841.3749	72	684.948	502.9	491.305	280.825	440.338	353.0956	1903.15	921.34	828.185
2570	842.3466	71	1435.73	1300.7	1403.01	727.355	1075.45	660.5649	3429.12	1316.79	1698.05
2571	842.7821	82	1067.84	608.21	1216.59	775.614	1118.64	691.8267	1446.03	1651.91	596.853
2572	842.8285	84	1048.26	583.12	1315.99	796.531	1085.68	701.8849	4417.46	1572.24	645.494
2573	843.3388	84	632.497	211.01	470.595	383.342	657.699	355.7432	2309.14	872.432	159.14
2574	843.8301	87	323.68	181.64	492.618	298.775	307.787	87.09256	1018.42	438.562	135.991
2575	844.3405	72	1382.07	1149.7	1421.61	585.494	900.695	497.5126	2328.88	1474.29	1543.15
2576	844.8261	87	583.138	261.3	545.938	558.793	580.224	471.7848	1702.87	851.153	233.708
2577	845.8085	82	515.651	253.01	660.052	417.914	450.72	273.7962	1665.21	543.689	328.681
2578	846.3368	70	933.875	763.7	944.558	400.852	726.318	289.1351	1880.5	986.393	794.58
2579	846.4709	70	357.77	307.17	403.17	419.58	540.967	417.9026	1478.64	710.348	458.471
2580	846.8061	83	638.368	175.76	747.583	384.703	363.486	396.905	1299.78	587.374	176.88
2581	848.3661	74	336.42	138.86	128.013	142.04	220.365	161.5048	968.24	665.438	341.168
2582	848.4656	72	839.908	529.68	567.53	429.892	524.433	458.2802	1060	1003.72	729.871
2583	849.3577	72	454.547	387.61	516.061	292.183	443.492	299.4277	1190.37	703.969	680.917
2584	849.8309	86	170.24	160.38	452.989	203.121	278.616	99.84333	1152.62	389.26	203.66
2585	850.3772	72	672.014	446.88	513.269	251.542	535.182	363.2956	1825.98	930.587	755.605
2586	850.3835	67	799.396	373.82	637.435	312.278	423.04	432.5021	1524.36	871.974	599.475
2587	850.8177	84	3105.69	1474.4	2686.07	1966.65	1722.33	1801.987	7692.32	4180.24	1721.63
2588	851.3221	84	446.122	240.01	482.132	245.241	233.925	228.0483	1662.01	556.227	226.35
2589	851.8228	82	916.046	398.4	793.573	468.895	836.312	489.665	3369.35	1258.34	428.556
2590	852.3793	70	866.719	786.43	676.037	610.364	569.242	509.0162	2366.33	1099.76	785.401
2591	852.8159	85	1521.36	552.05	1375.92	884.437	937.769	859.9631	3244.5	1300.64	741.386
2592	853.3751	72	283.29	214.49	184.114	231.768	214.796	179.6715	1032.79	571.969	285.714
2593	854.3761	70	774.226	638.65	666.146	496	652.362	435.0535	2379.02	1219.9	837.6
2594	855.8349	91	753.976	289.82	563.872	411.466	481.182	381.9888	1722.95	1608.05	277.935
2595	856.3708	70	574.194	557.31	482.689	378.594	480.452	350.78	2072.71	792.477	582.169
2596	856.7663	82	781.32	351.79	815.511	645.692	707.052	273.6	1946.33	888.659	475.53
2597	856.8322	91	1161.91	372.69	1006.12	985.228	769.146	510.2474	1507.11	1321.66	523.611
2598	856.8672	84	205.908	4.9311	245.687	175.778	188.441	130.6219	1089.73	89.4824	59.2423

2599	858.3665	67	370.814	350.44	377.277	300.188	456.691	345.5895	1330.91	772.778	646.34
2600	858.3765	72	371.69	325.03	326.162	244.407	458.988	295.7287	1305.63	714.661	597.95
2601	858.755	80	1097.72	317.11	909.651	803.257	870.406	593.9386	1290.78	988.264	413.09
2602	858.7993	83	863.28	293.22	672.653	662.46	729.026	421.0147	2097.03	919.409	400.467
2603	859.7974	84	351.348	190.92	589.624	356.913	518.027	211.6222	915.642	507.159	85.2
2604	860.3742	72	520.421	437.58	373.673	317.968	407.761	309.1364	1626.58	838.719	650.671
2605	860.8478	86	10491.8	3911.3	6754.85	4842.56	5298.12	6132.348	26493.2	14998.6	5011.11
2606	861.35	85	1248.87	643.64	978.221	1031.28	676.052	765.1989	3803.7	1890.59	634.611
2607	861.3674	72	458.196	553.22	429.516	642.954	456.981	469.0014	1415.81	1293.42	518.033
2608	861.8506	86	2090.14	915.33	1744.96	1278.39	1124.98	1466.13	7312.03	3466.61	1161.66
2609	862.3649	72	643.343	452.71	511.665	346.611	454.639	411.325	1597.43	1269.05	681.52
2610	862.4207	72	660.929	450.22	431.722	361.261	435.898	430.083	780.236	885.105	679.637
2611	862.8467	86	1193.96	489.42	892.576	640.421	677.613	727.6534	3868.88	2036.66	578.847
2612	863.3658	72	617.511	268.65	313.257	319.24	353.843	273.6491	1515.84	563.676	404.581
2613	863.8231	84	1211.2	448.62	1310.89	922.284	943.464	637.97	3351.98	1608.6	362.358
2614	863.8299	82	841.237	452.99	1166.96	802.395	1008.03	559.2625	2732.88	1473.87	371.204
2615	864.3194	84	539.535	231.24	432.478	237.982	405.882	228.0597	2557.03	959.469	240.341
2616	864.361	70	568.13	595.29	390.467	352.433	502.664	395.4368	1686.1	900.315	567.616
2617	864.8241	86	1230.72	363.61	831.562	728.67	944.949	628.111	3600.51	1701.47	517.477
2618	865.3198	86	327.265	159.65	306.073	167.58	361.63	120.8822	1400.27	275.775	280.2
2619	866.357	72	912.383	739.81	619.917	551.053	833.497	551.5966	2597.97	1143.16	931.245
2620	866.7919	83	2813.17	1149.2	2839.02	1877.65	1657.52	1553.939	4681.29	2363.03	1196.65
2621	867.358	72	303.525	233.2	239.855	212.265	180.523	221.1743	1188	572.283	325.819
2622	867.8528	87	1087.12	430.79	887.397	716.823	711.608	728.67	2378.74	1313.34	468.465
2623	868.3558	71	1384.47	959.98	895.291	781.389	1311.26	727.9213	3805.8	1635.46	1325.37
2624	868.7876	83	1126.27	724.34	1896.09	1421.68	1476.15	1105.865	1926.09	1211.93	749.944
2625	868.8342	85	1597.3	719.85	2138.63	894.805	939.362	1331.794	6391.42	3314.72	735.287
2626	869.3333	86	846.625	169.48	1065.75	373.22	433.804	475.3748	2577.65	1508.65	274.161
2627	869.3518	74	1675.66	648.54	1441.28	790.842	895.836	727.7472	3347.4	1113.73	738.529
2628	869.5578	97	142.386	261.54	1213.44	756.952	224.238	257.6659	383.049	153.986	2566.17
2629	869.8387	86	880.832	242.01	882.076	532.754	742.57	487.0517	2464.78	1236.84	310.317
2630	870.3373	84	322.361	207.21	349.969	138.497	231.426	101.4553	981.368	429.112	91.2459
2631	870.3524	71	1143.23	1091	957.664	782.192	932.524	727.1601	2357.2	1579.92	1473.85
2632	870.8197	84	499.921	296.49	608.361	419.703	620.448	321.1444	1863.3	745.306	263.397
2633	871.3445	72	888.582	494.96	519.418	358.979	521.591	254.7332	1369.02	911.364	559.311
2634	871.8131	82	846.189	399.32	854.986	572.871	610.127	399.735	1963.63	949.643	383.686
2635	872.3496	70	742.492	875.24	954.029	561.774	744.381	773.2427	2141.58	1044.73	914.774
2636	872.807	83	579.393	278.93	592.773	283.645	266.371	301.0086	1708.32	744.37	194.532
2637	874.3485	72	851.557	582.3	724.24	484.821	345.98	436.3252	1444.41	902.145	706.431
2638	874.7784	80	349.724	194.51	273.163	210.4	409.425	225.09	801.119	287.525	75.18
2639	874.8605	86	2080.39	885.47	1306.73	792.622	910.611	1097.535	4732.4	2450.99	1066.11
2640	875.8467	85	956.182	389.61	873.949	827.499	820.088	541.3751	3272.02	1351.03	454.592
2641	876.3353	74	664.839	574.52	756.904	294.013	713.172	406.5883	2413.61	940.772	520.495
2642	876.821	85	6806.84	2597.3	6115.02	3647.47	4036.99	3818.621	14669.8	8122.38	3062.57
2643	877.3216	84	300.673	128.21	586.774	409.328	409.149	267.0751	1631.98	888.34	149.34
2644	877.8233	85	2009.61	925.42	1787.67	991.964	1080.95	1224.207	4584.93	1948.08	652.347
2645	877.8305	85	2031.95	934.22	1570.97	1043.91	1218.58	1125.04	4623.19	2174.83	686.183
2646	878.8123	85	1358.62	616.86	1442.54	865.172	923.392	939.5789	3452.54	1559.5	864.854
2647	878.8203	84	1440.6	621.59	1279.48	890.18	851.262	874.5621	3648.61	1647.24	945.128
2648	878.9093	88	1090.02	100.44	211.433	314.466	247.664	156.735	506.481	405.685	163.036
2649	879.4642	71	284.76	266.93	208.8	312.596	295.804	217.2741	1234.57	434.831	307.254
2650	879.8096	86	841.212	273.65	761.813	574.331	536.904	397.509	1202.33	937.426	399.579
2651	880.2963	82	365.29	315.87	231.427	108.001	364.931	113.6429	1069.17	409.032	348.645
2652	880.803	83	580.356	274.26	632.14	489.173	670.742	297.1911	1262.84	746.816	303.408
2653	881.4746	88	439.238	415.2	316.211	707.246	526.066	411.9041	4518.44	663.711	553.979
2654	882.346	74	751.725	529	850.762	320.975	501.17	312.5521	1485.15	941.885	756.311
2655	882.4782	86	23.9558	79.854	15.6541	311.514	97.0103	62.755	1867.44	24.5759	72.4323
2656	882.7694	82	1051.15	516.46	1219.32	953.284	696.52	583.5889	2178.1	984.058	520.439
2657	883.4606	71	719.283	493.21	494.438	478.198	1067.37	1069.526	1444.3	988.632	472.419
2658	883.5356	1174	223.559	294.09	86.296	939.094	648.255	627.3987	90.6514	237.483	331.204
2659	883.5358	1055	1074.33	1521.1	546.365	3026.79	1885.26	3071.786	526.844	849.054	1462.91
2660	883.5366	1141	117.895	179.24	66.8615	538.274	515.436	227.8125	57.1846	148.862	165.262
2661	883.5367	1090	176.87	253.8	81.7964	2144.2	481.62	916.9246	122.337	192.557	238.925
2662	883.5371	1110	285.708	279	160.339	1277.6	656.847	1201.779	191.106	261.56	290.716
2663	883.5372	97	1110.71	773.87	1072.7	934.409	2816.4	2345.97	3554.99	1256.44	664.27

2664	883.5373	1189	174.342	149.82	38.3374	1334.93	478.132	288.5422	96.8369	230.259	249.59
2665	883.5375	1153	292.356	318.34	122.416	1001.63	679.636	673.4955	127.926	277.44	315.77
2666	883.5389	1129	147.154	111.01	69.4571	611.063	509.738	459.0617	72.3086	116.781	137.029
2667	883.5389	174	350.728	35.51	440.679	69.3343	180.964	130.1615	491.882	165.551	21.3966
2668	883.5392	145	547.111	175.1	621.467	250.087	425.646	301.0341	775.479	279.349	107.674
2669	883.5401	190	337.787	34.674	419.912	65.9035	141.602	88.19333	345.942	189.454	13.1011
2670	883.8223	86	519.166	225.86	667.925	422.846	681.892	223.457	1697.89	885.771	243.422
2671	884.3296	72	778.959	746.62	773.082	497.067	879.965	265.99	1656.99	975.184	736.785
2672	884.5412	96	880.533	533.68	1175.55	622.62	2446.6	1925.71	2511.64	596.394	426.189
2673	884.7601	82	898.564	457.4	1050.77	558.772	907.059	478.0141	1188.55	959.928	504.095
2674	884.8173	83	972.152	471.22	1148.42	629.464	900.499	601.591	3091.35	965.211	500.399
2675	885.33	79	818.563	336.01	703.72	256.227	464.699	384.8519	1571.62	703.166	564.524
2676	885.4582	71	436.063	410.57	368.946	264.219	417.353	371.8452	1179.75	768.651	395.78
2677	885.541	92	864.847	544.47	1055.89	524.639	1227.46	1107.421	1922.76	1121.47	652.386
2678	885.5485	1051	1988.27	1291.8	839.46	545.982	2232.67	1899.238	987.785	1175.63	631.379
2679	885.5486	1143	157.5	136.39	85.6152	114.92	280.162	197.7133	107.78	114.4	78.061
2680	885.5488	1118	351.844	217.65	157.878	306.142	613.258	469.5122	236.486	236.091	180.725
2681	885.5489	261	281.256	153.67	1047.66	138.561	242.331	293.8549	1119.19	1369.28	157.948
2682	885.549	281	347.348	169.75	1059.83	126.178	296.224	345.0321	1047.64	1182.55	283.288
2683	885.5491	1168	628.052	349.16	190.167	416.705	1098.23	842.1721	460.2	448.839	252.169
2684	885.5493	237	317.704	152.41	1255.35	127.122	216.159	279.0056	1913.28	2512.77	147.647
2685	885.5495	1070	670.918	545.71	392.915	398.594	1631.87	874.6427	497.149	407.344	315.132
2686	885.5495	299	342.135	171.2	707.067	120.648	296.593	304.3755	754.427	824.132	378.538
2687	885.55	1195	364.75	228.74	103.35	273.248	0	0	0	0	157.907
2688	885.5506	1095	787.41	466.13	320.45	516.049	902.509	1035.499	455.82	438.529	279.24
2689	885.5506	192	414.219	220.57	1348.63	202.173	226.104	344.1311	2227.98	1873.14	135.789
2690	885.5507	336	378.591	176.77	303.996	55.2216	456.091	286.62	377.18	341.021	218.512
2691	885.5509	314	330.939	161.49	436.767	68.7079	302.656	274.1909	557.77	628.057	370.544
2692	885.5509	217	476.16	282.33	2657.83	223.133	281.047	337.0478	3353.91	2887.81	194.814
2693	885.5509	139	929.035	667.06	2775.32	521.081	615.054	1005.184	3713.41	5340.13	395.086
2694	885.8215	84	609.705	251.44	504.598	342.538	541.079	322.6781	1940.3	647.364	137.633
2695	886.3261	71	606.48	544.3	362.522	306.667	420.518	426.2756	1374.92	823.542	700.851
2696	886.5513	294	128.628	71.793	290.528	31.0663	139.573	132.4273	278.207	293.687	175.412
2697	886.5518	314	154.299	101.49	201.313	39.2787	170.262	126.7424	236.969	273.849	184.036
2698	886.5528	183	464.963	298.72	1455.19	307.909	282.484	505.0509	1844.67	1824.23	208.864
2699	886.5529	264	178.267	101.91	626.679	104.963	202.228	235.9361	733.751	962.055	155.316
2700	886.5538	136	864.663	698.55	1811.52	574.785	625.801	1089.668	3620.16	3048.75	572.681
2701	886.5544	217	360.369	247.28	1054.38	215.046	238.94	315.3283	5227.55	1891	171.875
2702	886.5547	334	547.016	332.62	444.316	68.1696	764.154	596.9088	519.817	535.247	391.289
2703	886.5549	194	183.113	119.55	681.085	119.976	131.624	211.9628	1138.05	1067.5	79.9536
2704	888.3372	72	893.871	566.42	685.417	365.603	538.628	281.2154	1488.06	974.58	829.454
2705	888.8367	83	315.235	215.82	262.362	315.375	408.746	160.7148	1071.35	144.552	108.703
2706	889.8252	88	908.895	275.07	677.4	531.7	549.766	484.2779	3158.43	1634.98	190.109
2707	890.3424	68	474.798	276.93	396.973	196.47	147.339	229.6359	742.771	518.711	346.478
2708	890.8339	86	1637.58	531.93	1184.65	910.559	770.058	839.0893	2952.1	2234.61	633.275
2709	891.3505	76	583.499	383.3	433.48	477.123	699.544	357.1364	1659.82	973.918	524.151
2710	891.8223	83	554.965	198.79	400.246	503.668	431.728	340.1799	1869.98	781.757	98.2513
2711	892.3426	72	449.894	295.49	324.178	267.386	247.657	281.6097	1036.4	669.697	489.201
2712	892.7956	84	2827.53	1239.5	3175.35	1182.42	1849.2	1772.823	5668.75	2541.37	1241.78
2713	893.7985	83	908.093	330.85	1135.96	486.414	969.253	554.5785	1806.65	1415.69	411.32
2714	894.7904	84	1316.55	608.78	1687.61	1214.29	1164.79	737.505	1043.34	2719.79	582.271
2715	894.8403	86	1607.34	630.09	1562.75	1230.63	1193.07	771.7588	7232.31	3186.58	606.31
2716	895.3414	86	1047.46	469.03	929.851	548.895	534.121	577.2891	3810.76	1792.06	527.179
2717	895.8526	88	766.378	276.13	771.594	556.442	784.455	473.7468	1549.24	1269.49	320.259
2718	896.7755	80	525.256	261.97	651.372	350.09	428.252	434.8677	1226.54	776.737	302.958
2719	896.8223	88	595.277	281.74	653.069	418.224	458.722	450.0057	1865.09	882.557	314.294
2720	897.3327	71	642.937	460.52	414.722	362.505	246.938	345.5336	1125.72	756.728	564.054
2721	897.8095	84	1019.09	434.5	918.417	682.548	508.054	536.8087	3024.17	1291.44	366.368
2722	898.3319	72	839.894	495.49	609.757	300.406	598.115	511.885	1457.36	967.873	610.514
2723	898.8095	84	741.991	411.96	707.593	524.658	454.834	561.0758	2075.81	1053.08	252.938
2724	899.3294	74	899.488	440.26	694.408	393.962	563.073	420.7562	1859.66	1014.51	625.78
2725	899.4374	71	406.7	371.68	432.521	280.175	458.011	331.8657	1309.01	771.455	545.266
2726	899.813	87	468.057	70.321	528.851	325.645	377.476	269.1849	1148.36	726.609	248.938
2727	900.7777	80	445.7	259.79	369.103	231.44	235.613	92.232	650.661	389.55	56.385
2728	900.8521	84	637.561	317.87	612.386	468.679	410.626	211.5972	1213.88	792.279	112.348

2729	901.4308	71	385.7	308.57	405.648	260	285.209	363.6663	1110.05	627.209	385.668
2730	901.8487	88	347.357	215.93	520.468	371.792	434.532	218.5628	2186.61	1077.08	311.277
2731	902.8264	86	1809	591.45	1862.42	925.486	983.313	1052.213	6530.66	2580.1	605.503
2732	902.8352	86	1851.62	589.74	1824.35	921.954	1147.54	1027.896	6538.35	2583.46	613.598
2733	903.3334	86	926.889	479.71	981.171	490.035	653.099	590.3392	2174.19	1620.34	590.719
2734	903.8308	87	736.647	218.47	799.325	645.615	707.79	399.546	2780.09	1178.71	246.2
2735	904.8037	83	656.528	174.08	613.921	515.896	477.834	459.6993	1594.53	940.509	184.383
2736	905.8034	83	941.619	311.79	877.221	465.806	386.155	452.2958	2618.98	905.987	370.641
2737	906.328	72	771.524	461.85	703.283	254.949	577.94	334.2662	1161.52	862.38	628.41
2738	906.8054	83	707.702	292.72	845.733	569.97	700.425	467.8492	1824.12	731.123	253.697
2739	908.3272	72	661.917	446.06	547.436	340.771	540.243	417.5985	1028.48	679.735	565.634
2740	908.7726	82	1352.63	503.09	1524.74	891.9	867.534	654.72	2973.11	906.05	471.215
2741	909.3278	74	511.909	231.16	481.171	187.849	384.604	118.2781	1469.18	560.601	375.359
2742	909.8326	86	845.406	329	913.913	642.488	486.438	503.7108	2339.94	1059.96	324.374
2743	910.3365	71	719.62	603.6	475.28	350.416	586.014	358.1916	2137	893.176	598.877
2744	910.7678	83	1135.26	519.29	1481.32	880.118	1000.45	758.1673	1515.82	1576.44	469.7
2745	910.8148	85	1047.34	514.99	1421.08	823.106	999.664	642	3676.63	1552.18	465.85
2746	910.8225	84	670.142	527.14	1325.87	667.87	746.535	511.806	3476.53	1598.82	475.443
2747	911.3197	83	533.259	382.74	769.29	482.418	513.615	364.7875	3233.09	1142.7	397.211
2748	911.8274	86	537.315	274.66	537.229	279.795	366.784	375.5357	1073.74	773.467	140.525
2749	912.3306	68	586.157	559.96	402.017	322.605	203.268	492.3448	1482.3	791.154	529.003
2750	912.8142	83	487.862	229.3	605.07	325.742	158.755	390.6993	1390.83	763.842	160.448
2751	913.7833	83	442.667	190.07	650.684	343.446	305.345	226.4983	1472.08	665.752	245.299
2752	914.3362	71	777.245	475.32	615.323	395.914	491.783	357.371	1404.25	817.92	543.777
2753	914.7875	83	466.718	197.36	625.583	512.305	293.55	296.9832	1548.54	624.536	243.857
2754	916.3321	71	364.748	412.92	491.721	328.157	406.313	284.6429	1248.03	634.251	547.775
2755	917.81	83	579.764	236.24	664.382	434.489	283.515	420.9147	1236.32	628.95	279.227
2756	918.8038	84	2835.32	795.2	2130.53	1266.9	1398.01	1538.83	5729.03	3422.5	1133.52
2757	919.3038	83	740.616	507.38	926.297	347.722	690.414	494.669	1873.56	1085.78	536.038
2758	919.8039	83	859.427	276.24	902.012	588.691	697.805	644.144	2907.04	1173.46	403.947
2759	920.3146	72	695.415	391.35	578.115	359.147	296.497	361.422	1242.21	738.416	632.794
2760	920.8	84	913.157	475.59	877.336	897.112	692.985	575.6734	3142.2	1559.58	474.979
2761	921.7943	82	405.923	172.36	473.858	227.969	506.577	42.58333	1250.62	479.666	176.28
2762	922.313	72	732.551	457.9	544.853	394.745	287.006	293.8259	1310.82	770.445	385.556
2763	922.7794	82	757.243	357.3	575.211	522.086	513.427	457.6616	1281.21	626.551	212.884
2764	923.8184	85	718.237	207.49	713.031	378.818	436.899	451.5128	2128.74	1561.43	297.264
2765	924.3235	77	1001.35	557.16	671.834	450.869	641.633	447.0247	2085.53	1253.5	805.183
2766	924.7493	80	683.598	327.46	470.779	490.77	629.613	448.0533	1294.96	634.2	361.785
2767	924.8257	87	911.393	304.91	629.307	697.582	793.946	498.029	2032.51	1380.73	315.113
2768	925.8107	83	553.584	208.9	617.076	331.118	305.906	313.241	2074.35	732.107	253.548
2769	926.3139	72	934.88	865.76	694.09	560.326	651.19	603.5812	1942.36	1129.11	935.949
2770	928.3114	71	880.701	812.08	717.984	522.409	751.138	597.5788	2320.22	1149.84	1172.93
2771	928.8341	86	6412.21	2408.6	4489.34	3141.07	3168.94	3314.376	18621.1	9748.63	2920.55
2772	929.3381	87	833.621	336.41	435.863	362.036	467.802	344.8684	3461.58	1555.72	381.916
2773	929.8384	86	1445.2	568.17	1290.26	818.468	890.347	894.11	4093.73	2231.81	831.79
2774	930.3088	70	727.46	636.77	644.448	367.671	600.468	471.3047	1878.27	836.193	841.973
2775	930.7743	80	965.681	338.23	850.986	615.421	695.265	676.7323	640.732	1159.89	447.048
2776	930.8275	87	1065.58	353.33	804.97	705.469	751.146	678.9975	3572.15	1319.95	479.755
2777	931.8024	84	1173.91	371.18	755.671	547.866	608.566	329.411	2881.18	1313.78	359.194
2778	931.8175	83	1563.25	380.24	925.777	630.639	633.604	535.7028	3019.06	1191.82	385.761
2779	932.3106	82	707.192	560.24	861.076	350.075	625.487	496.026	2725.59	1285.44	580.072
2780	932.8181	84	1147.48	403.01	759.824	652.933	717.876	565.936	2573.58	1535.35	464.566
2781	934.3058	74	580.445	426.93	403.5	191.64	389.955	269.1526	1105.11	579.949	602.252
2782	934.7785	82	2049.08	913.58	1878.05	1365.44	1354.85	1166.495	3437.41	1938.3	802.545
2783	935.7812	81	951.521	345.03	896.08	598.307	689.281	602.4716	2185.43	1173.89	409.226
2784	935.8418	87	1044.22	429.77	981.856	719.456	859.265	632.9325	2397.76	1397.12	551.59
2785	936.7839	80	1727.43	672.65	1946.04	1200.73	1185.56	1003.101	1924.01	2615.69	804.404
2786	936.8197	85	1807.87	698.59	1461.14	1283.08	982.376	1025.832	5205.59	2675.5	833.708
2787	937.3289	85	894.05	301.25	791.633	542.81	570.446	556.0739	3325.93	1202.48	513.414
2788	937.5332	264	11.3191	4.4299	12.4607	62.3517	69.8605	4058.273	172.105	35.6469	1.46774
2789	937.5347	337	1.62968	0	3.618	32.3635	30.0889	729.3134	20.5376	2.96729	2.71351
2790	937.5347	91	171.477	120.82	397.836	16252.3	151.224	49.53252	885.512	167.564	560.426
2791	937.8093	86	759.535	163.25	681.169	662.605	434.551	422.8398	2697.41	1363.37	311.801
2792	937.8229	87	768.863	164.23	746.042	667.829	442.615	435.7313	2461.52	1146.62	314.036
2793	938.5348	284	8.81478	6.5893	5.06526	27.1667	22.5506	1133.72	30.411	7.00792	3.92484

2794	938.5353	337	1.65545	1.2604	1.85015	94.419	16.4805	559.7992	7.68667	1.496	0
2795	938.5366	305	6.31302	2.5205	5.60842	68.5475	19.838	1045.329	30.2858	10.5808	2.50235
2796	938.5377	264	3.71015	9.8408	4.99117	15.075	9.5016	900.1988	21.346	12.3573	2.69741
2797	938.5384	93	139.701	130.79	420.854	10905.8	331.966	152.7432	538.628	281.041	693.264
2798	938.7575	79	557.794	269.35	554.192	529.434	519.836	476.4	678.652	811.925	232.761
2799	938.8144	84	672.419	292.26	557.53	599.661	456.444	573.642	2055.27	795.105	243.164
2800	939.5382	91	536.084	377.63	636.563	7264.12	670.312	652.3582	1692.66	743.181	428.674
2801	939.7944	84	920.364	313.16	1075.45	468.695	642.87	602.35	2313.17	1247.53	258.712
2802	939.8042	84	975.954	329.68	1038.32	489.713	663.789	594.198	2564.84	1282.22	275.562
2803	939.8566	86	474.524	175.58	511.154	172.096	276.687	237.2095	1724.72	483.759	240.169
2804	940.5405	91	418.461	366.14	527.323	3445.84	459.683	363.0155	916.483	671.399	357.562
2805	940.7999	84	706.418	290.12	620.098	548.192	488.423	377.4188	1926.9	622.24	152.173
2806	941.4853	413	3.79662	1.3318	0	715.558	5.36784	11.30264	242.051	1.25396	0
2807	941.4856	282	8.81478	1.3179	6.33158	956.428	5.01125	123.7407	58.0574	12.6143	1.30879
2808	941.4857	322	8.912	2.7943	5.28662	4609.27	6.62702	86.12276	140.187	2.64702	4.16733
2809	941.4858	343	4.31175	0	12.0635	4960.87	15.138	97.23397	232.061	8.58942	1.42283
2810	941.4866	379	0	3.7704	1.41906	2017.52	2.50891	15.126	208.693	3.98242	0
2811	941.4868	396	3.7929	2.7276	1.2553	965.285	5.0376	5.0132	184.378	2.50771	0
2812	942.2894	72	731.308	616.87	736.392	451.804	806.922	350.1276	1206.91	1045.65	642.899
2813	942.4873	304	7.56272	0	1.43302	2061.13	3.77222	21.42	88.9246	0	1.25119
2814	942.488	361	1.33742	1.3357	1.25409	2033.8	1.3375	1.423289	110.859	0	1.2418
2815	942.488	282	10.0739	0	9.3395	699.261	0	26.63	64.2831	11.213	1.30737
2816	942.4881	343	4.4782	1.3268	4.76017	3182.77	5.33295	14.03538	152.166	2.65011	0
2817	942.4903	322	8.56139	1.3641	7.84416	4361.62	2.72433	13.06347	168.646	5.69925	0
2818	942.4905	397	5.05404	2.5179	1.33583	868.476	0	8.775429	138.522	1.25504	1.32298
2819	942.8451	86	1388.57	648.39	1236.23	691.054	546.588	918.1391	3546.06	1763.82	827.2
2820	942.8584	87	1376.36	644.43	1268.94	691.162	556.787	866.3916	3527.64	1797.86	824.75
2821	943.4847	322	3.23075	2.711	9.47979	2659.89	5.01307	2.717474	84.2467	4.07773	0
2822	943.4851	304	5.04565	0	6.96871	2135.77	12.5745	6.297273	115.52	9.20597	2.50238
2823	943.4856	362	9.84722	2.6557	14.1571	4696.19	19.9728	15.42221	289.269	10.1122	0
2824	943.4861	381	2.51846	1.2567	4.22712	1424.9	3.76589	7.569	175.395	3.98067	2.64667
2825	943.7771	82	674.645	215.5	668.72	353.432	436.602	312.095	1171.61	886.684	235.074
2826	943.8321	85	889.175	222.51	976.452	361.981	476.312	433.2508	2387.77	1390.01	309.411
2827	944.4863	322	8.18912	6.5783	23.797	6550.87	31.5931	29.5871	382.686	31.0712	4.00378
2828	944.8079	85	5068.51	1804.3	3890.55	2825.58	2593.83	2543.162	10255.4	5642.57	2048.08
2829	944.8128	85	4393.71	1616.4	3612.52	2628.55	2593.79	2143.69	9998.98	5587.69	2078.93
2830	945.3294	83	661.853	362.13	711.582	431.751	493.552	340.4894	2253.57	1113.76	395.973
2831	945.8075	85	1737.08	658.41	1527.02	781.886	741.641	745.4293	2770.13	1673.81	690.94
2832	946.295	75	820.346	487.11	683.594	354.848	578.735	398.535	1124.19	991.796	648.946
2833	946.809	85	1096.76	469.34	981.672	597.278	600.481	647.703	2946.2	1487.57	643.525
2834	947.784	82	646.112	367.75	850.986	411.509	611.421	434.8677	971.558	884.907	337.442
2835	950.7522	81	1373.7	549.51	1152.03	1070.21	786.065	872.4166	2681.04	1167.18	522.792
2836	950.7624	82	1319.71	537.66	1518.54	1025.29	766.179	804.9881	2171.11	1107.95	459.289
2837	951.5126	282	3.77387	1.3402	10.1127	1664.83	5.00978	35.32582	74.3033	37.832	0
2838	951.513	466	1.38613	3.7574	0	475.781	8.82339	3.753882	162.257	9.65813	2.49988
2839	951.5141	381	2.5152	1.2545	5.62752	3464.66	6.2469	8.851862	175.299	13.9708	0
2840	951.5146	304	6.30033	2.5168	21.1956	9274.16	13.8239	27.71851	250.697	41.0331	2.50243
2841	951.5147	411	3.80903	1.2533	0	1158.75	6.99929	5.016258	203.664	7.53213	0
2842	951.5147	363	2.64179	3.9666	9.25867	11452.7	17.2192	11.10057	360.856	36.0584	0
2843	951.515	395	1.26409	3.7797	3.75776	2884.2	7.56545	8.767907	270.983	20.0742	0
2844	951.5153	337	8.6064	6.5628	34.7624	34467.3	20.6232	32.76565	633.833	68.9463	4.08942
2845	951.5158	316	4.46976	4.2011	23.8143	18665	7.96071	19.88316	337.019	60.9303	0
2846	952.5174	413	2.53008	2.5075	4.01275	1360.18	6.72596	8.790902	231.843	10.0248	0
2847	952.5178	319	0	0	1.252	1952.53	0	1.271	26.42	1.242	0
2848	952.5179	394	1.26324	1.2599	3.75631	2188.11	7.56057	6.256098	187.407	5.0221	5.37389
2849	952.5185	465	2.93492	1.253	4.36769	481.107	7.58453	2.513043	170.376	8.04514	0
2850	952.5186	379	5.0368	2.5143	6.72	4255.57	7.50816	6.300408	258.451	10.7089	0
2851	952.5186	336	14.5299	6.5886	26.9398	36481.4	21.1021	28.95405	892.17	43.209	1.36819
2852	952.5196	304	7.5604	1.259	16.9529	7442.93	5.02243	22.6608	206.774	17.1992	1.25226
2853	952.7987	83	822.927	366.38	706.733	641.669	674.612	531.0514	3024.76	1099.72	409.731
2854	953.3057	82	468.614	300.84	435.25	291.229	355.01	338.6206	1073.68	679.096	303.174
2855	953.516	282	13.8624	2.648	64.6076	1211.33	30.1004	52.18965	116.222	96.0826	6.56
2856	953.5174	378	11.32	5.0271	11.3683	1363.58	10.0406	8.840462	101.082	21.2866	4.2275
2857	953.5178	395	15.168	5.3563	20.0336	1449.47	34.0308	20.07168	169.326	41.4014	1.3215
2858	953.5181	317	8.86709	2.9187	12.5709	2863.73	18.9663	15.08073	82.0484	31.5304	7.27789

2859	953.5181	304	21.4238	5.0403	50.1677	4231.78	15.0836	60.46189	133.398	73.2644	22.5253
2860	953.5184	338	60.2887	28.878	39.1795	14704.4	123.964	105.2092	395.078	80.29	34.0785
2861	953.5198	361	21.4698	12.116	21.4949	3888.69	52.5974	38.8692	170.869	34.4651	11.1232
2862	953.8029	84	767.74	202.4	575.174	299.845	495.961	345.4867	1902.89	719.291	132.431
2863	954.5177	336	53.9055	46.393	129.192	11407.5	174.107	1804.912	594.424	151.239	91.7787
2864	954.5184	379	0	5.0263	16.1526	978.015	12.5288	170.3204	122.14	17.3974	9.34187
2865	954.5248	52	118.255	74.729	74.424	1179.45	130.679	88.67118	372.479	127.343	46.5298
2866	955.3028	72	580.401	184.66	363.454	274.371	422.282	178.8072	932.209	530.022	237.226
2867	955.519	335	12.7685	16.001	10.2763	1702.3	20.0904	405.0977	134.656	19.006	17.1151
2868	955.5212	314	10.5093	3.9976	7.96145	916.541	10.6501	351.496	59.4139	3.97527	9.76554
2869	955.5274	52	32.2408	25.288	48.6182	782.659	40.8689	11.22133	110.369	49.0151	38.4746
2870	955.8037	83	522.782	146	477.328	404.73	547.838	312.7179	1312.35	628.387	216.755
2871	957.8133	84	473.715	152.4	417.743	334.563	310.488	225.504	1415.23	766.878	275.945
2872	957.8277	86	764.029	195.12	684.016	453.226	400.654	538.0445	2906.55	1432.21	391.773
2873	958.2823	72	730.578	446.34	697.3	234.463	390.012	327.4411	1386.95	1048.71	512.753
2874	958.8165	84	1022.52	477.09	939.622	511.353	592.428	538.8152	3021.99	1405.78	462.83
2875	959.844	87	621.657	234.82	376.532	315.586	533.136	326.9499	1240.02	717.494	138.299
2876	960.7829	84	2032.17	965.02	2003.78	1119.12	1420.41	1295.04	4292.97	2049.08	1033.23
2877	960.7883	84	1566.29	1005.9	1808.64	1058.54	1421.86	880.3398	4097.72	2115.78	984.687
2878	961.6113	38	31.4736	19.079	5.49658	40.0703	18.8923	54.82807	1242.21	26.4022	0
2879	961.6142	6	0	0	0	0	4.72114	0	1207.65	0	0
2880	961.7941	84	897.702	347.42	763.165	600.88	649.117	504.6986	1859.59	1007.62	335.443
2881	962.6158	1	0	1.1738	0	0	4.99408	0	933.11	0	1.17333
2882	962.6169	12	16.3996	10.575	12.3536	30.0439	18.7002	22.89502	2483.9	13.5204	1.63535
2883	962.8268	86	1772.13	466.18	1048.03	957.441	1166.88	792.4246	5032.77	2693.54	611.318
2884	963.3339	87	747.275	177.85	638.158	392.882	522.379	350.7136	2431.7	1298.35	343.355
2885	963.8311	91	400.117	176.29	535.807	422.85	462.068	232.0871	1058.44	888.34	332.004
2886	965.8041	85	1061	335.02	816.201	591.811	687.384	452.8842	3381.16	1414.93	165.235
2887	965.9995	87	3180.32	1846.1	2100.38	3015.1	2707.02	2453.198	5528.8	2897.42	1810.53
2888	966.0023	109	5302.8	4299.4	5237.41	7114.34	7259.4	5862.388	13127.1	6364.16	3873.23
2889	966.0026	37	529.276	415.23	633.747	881.552	731.864	691.8054	1552.06	772.064	345.423
2890	966.0056	6	1099.88	527.52	793.855	1050.78	964.987	801.1016	1114.24	1076.43	333.544
2891	966.7944	84	655.456	233.48	555.016	363.287	549.114	407.5833	1584.46	821.543	341.543
2892	967.5087	1059	25.4276	32.984	21.9582	87.912	85.2267	69.99934	2238.88	41.7845	25.1435
2893	968.2981	71	915.028	635.07	534.041	476.873	764.039	476.0354	1578.9	1191.89	732.326
2894	968.5033	279	57.8976	2.6333	88.0346	2109.88	16.2998	50.578	174.979	9.80259	0
2895	968.5052	343	85.196	7.8863	101.514	12957.5	41.3982	49.42745	530.492	10.0684	4.28762
2896	968.5052	318	59.2003	1.3328	71.7992	11512.8	21.3346	43.89508	349.143	9.31323	0
2897	968.5053	300	73.045	1.2584	83.0659	6514.44	20.1126	37.80453	287.051	7.99536	1.25133
2898	968.5054	378	50.3886	1.2561	39.536	5184	33.804	32.73957	439.703	5.64735	1.32491
2899	968.5057	396	39.1992	0	22.7297	2753.23	31.5408	31.33265	388.089	3.76368	0
2900	969.5063	411	20.2715	2.5115	6.97714	741.533	6.99857	11.27729	171.364	5.01058	0
2901	969.5067	332	32.696	1.257	32.6726	3827.3	8.51165	11.24585	148.703	11.8176	0
2902	969.508	395	7.58343	1.2582	10.8119	1955.15	10.0792	20.01219	234.554	3.76744	2.68062
2903	969.5081	343	34.8169	3.9939	48.547	7904.28	26.7639	32.57567	341.175	9.29656	6.6048
2904	969.5083	301	47.9198	1.2572	61.6507	5115.13	25.1333	40.968	248.38	14.4661	2.50158
2905	969.5089	361	36.0651	2.674	36.0467	5403.99	17.4388	15.662	266.526	4.60537	1.23631
2906	969.509	319	47.1905	1.3367	58.5804	8599.72	9.34528	26.66353	291.388	3.99247	0
2907	969.5112	279	20.093	1.3455	38.1748	1008.48	7.52514	41.64914	82.6264	7.33946	2.6742
2908	969.8348	86	423.878	148.47	548.395	297.378	448.349	254.8484	2195.06	1100	284.878
2909	970.2941	71	1005.88	782.69	661.902	601.282	842.231	516.5499	2615.3	1136.46	855.901
2910	970.5052	427	10.1054	2.8028	1.25628	261.651	10.0985	6.288966	71.6028	8.36954	0
2911	970.5062	342	17.075	13.082	16.3779	6144.6	35.5181	27.16822	295.547	12.8396	5.67021
2912	970.5066	301	16.3922	3.7725	21.0559	2791.47	11.3112	34.3899	170.71	18.4249	3.74991
2913	970.5075	396	6.32	1.3389	5.34026	1261.11	18.9432	6.2652	204.442	8.77882	0
2914	970.5083	319	10.6002	0	21.2689	4951.9	13.3255	22.65867	193.164	9.31538	0
2915	970.5104	378	8.81338	0	4.2409	2269.53	8.76659	13.852	227.617	12.7245	0
2916	970.8159	86	1541.26	475	1409.21	846.309	777.717	937.0362	4486.24	2488.9	491.791
2917	970.8241	84	937.131	391.56	1269.46	663.566	674.612	400.5413	3811.6	1773.56	402.28
2918	971.31	85	869.883	218.06	850.385	474.681	520.189	370.7409	2662.75	1255.11	293.918
2919	971.5073	395	1.2639	1.2591	0	441.75	6.29316	1.254737	180.175	146.772	59.3874
2920	971.5085	379	3.77858	1.257	5.61147	969.348	7.50468	6.296949	328.377	394.486	166.839
2921	971.5086	362	1.33867	0	1.34173	1176.95	1.34613	7.159048	114.561	46.4523	2.47298
2922	971.5087	342	1.41529	2.6142	5.91589	2642.89	13.6184	13.54737	175.141	7.36886	1.41046
2923	971.5097	298	1.26103	0	14.1608	1301.15	3.77736	15.1078	98.0278	5.29833	1.25146

2924	971.8174	84	984.298	326.97	736.386	396.513	461.743	404.6987	3506.67	1488.62	476.534
2925	972.2911	71	900.533	713.52	586.372	451.804	782.213	644.7448	1467.63	997.149	696.369
2926	972.8102	86	700.364	382.74	688.768	477.34	418.453	427.5594	2133.52	943.121	362.946
2927	973.7942	82	809.416	337.21	827.469	356.054	792.783	581.298	2630.87	908.724	446.527
2928	974.2773	76	883.997	576.99	576.427	528.35	582.864	701.937	1881.2	1163.05	528.178
2929	974.2922	67	888.72	600.36	499.14	454.132	542.949	682.8322	1795.69	1073.95	526.654
2930	974.797	83	1022.29	273.75	820.08	573.824	527.954	548.3656	2451.06	1037.04	340.104
2931	976.2789	71	492.1	340.35	340.96	206.667	236.127	239.78	1076.78	702.316	410.936
2932	976.7581	81	845.52	450.57	1002.64	706.629	773.933	526.88	1657.49	868.113	276.767
2933	977.281	76	496.975	325.02	275.233	215.028	254.903	250.1057	1489.23	529.293	316.531
2934	977.8287	87	781.676	282.99	760.905	542.188	859.131	432.8299	1947.54	1131.18	335.581
2935	978.2797	71	594.689	383.96	355.89	278.966	243.531	367.1718	1442.3	741.231	447.064
2936	978.3203	83	538.962	247.05	385.578	167.906	214.578	298.0466	1225.83	1107.68	353.206
2937	978.7835	80	1284.47	455.05	1382.54	681.675	955.13	657.9643	3405.87	1438.38	551.482
2938	978.8011	85	1326.97	468.7	1117.7	691.028	960.179	710.3298	3433.26	1786.23	604.522
2939	979.3116	83	666.623	231.52	750.749	333.212	436.249	432.7833	2496.82	913.928	332.479
2940	979.8018	84	821.036	160.37	640.086	456.364	586.095	505.5663	2327.83	1026	299.465
2941	980.2837	72	559.33	529.36	538.16	323.214	391.848	365.7061	1363.73	920.583	452.775
2942	980.7991	83	688.626	203.54	553.411	385.197	616.768	447.1863	1458.8	808.662	168.391
2943	981.5756	405	0	0	0	10.1883	2.54057	3.769714	140.816	151.352	64.9099
2944	981.5794	379	2.51802	5.0253	4.1997	132.579	68.8211	54.01766	2027.52	3859.27	1632.36
2945	981.772	82	440.421	261.6	547.162	247.474	434.393	272.5333	1271.48	729.249	124.865
2946	982.2732	74	892.549	792.72	673.369	519.262	775.833	551.0813	2011.68	1203.03	903.014
2947	982.5837	381	9.00088	7.855	8.01054	110.158	64.1627	49.68757	2706.58	2764.11	1145.64
2948	984.273	71	947.687	979.16	683.842	616.457	957.268	631.044	2596.74	1364.01	1078.22
2949	984.4984	1190	1.79373	0	0	16.48	15.3346	4.604211	820.176	0	1.78745
2950	984.4996	1060	8.352	17.776	12.948	57.1768	15.7373	20.19184	3151.5	5.63771	10.3811
2951	984.5001	1168	3.81053	0	1.32214	8.87793	14.544	3.412093	1190.18	1.5887	0
2952	985.2632	74	437.364	353.21	314.838	222.63	200.021	344.4198	1071	580.256	396.421
2953	985.5013	1062	2.20456	7.0074	3.91733	31.4708	18.9184	15.24099	2420.53	26.7864	11.8027
2954	986.2682	71	1381.69	1131.1	766.825	602.142	873.113	661.0937	2583.06	1620.36	1444.74
2955	986.7915	84	1970.47	875.22	1577.34	1097.74	890.598	1143.682	5111.05	2810.85	914.121
2956	987.2889	83	392.211	304.01	653.614	380.599	389.298	340.0515	1488.14	830.783	364.978
2957	987.6269	144	19.1393	9.0124	16.0659	39.9552	22.7269	23.584	1913.93	44.8478	14.3023
2958	987.6286	166	4.048	5.8741	14.0133	21.5207	30.1829	15.40218	1446.99	21.7964	3.07431
2959	987.7924	86	801.553	278.31	595.56	438.228	623.684	442.0225	2246.78	1147.77	297.932
2960	988.2662	70	1025.27	941.03	820.879	496.886	1090.08	611.0109	2317.4	1547.91	1163.52
2961	988.7956	84	1036.25	404.35	731.885	712.681	450.113	534.8822	3308.95	1206.82	468.033
2962	990.2628	71	823.971	714.31	518.428	466.56	562.622	523.1215	1698.94	1070.31	749.146
2963	990.7623	82	637.511	259.42	553.93	385.761	599.575	362.3232	1095.22	756.113	288.865
2964	991.5194	363	0	0	1.32815	1680.24	3.98689	2.794462	93.202	0	0
2965	991.5203	279	2.51492	0	0	324.925	0	29.01247	25.1837	0	0
2966	991.5208	398	0	1.393	0	325.748	1.25716	3.752903	55.2407	1.25871	0
2967	991.5209	317	0	0	0	2537.9	2.66667	10.65908	77.5153	0	0
2968	991.5211	378	2.51624	0	0	933.264	0	3.78375	91.2839	2.68142	0
2969	991.5219	300	0	1.2575	5.67446	1417.16	1.25614	21.42716	54.165	2.64989	0
2970	991.5231	343	0	0	0	2298.43	2.68261	9.961913	114.954	3.98424	1.32325
2971	991.5235	332	0	0	1.25748	1274.01	0	9.987429	42.4991	1.68822	0
2972	991.7981	86	761.802	189.3	387.311	255.424	255.83	337.2011	1572.74	1326.74	206.846
2973	991.8357	89	479.048	73.416	257.094	182.284	151.386	128.5491	828.24	882.212	150.557
2974	992.2612	72	634.138	378.1	287.077	173.622	359.497	331.6957	1258.67	811.739	507.593
2975	992.3015	84	919.598	517.55	624.157	461.082	580.783	493.4385	1590.48	1457.31	510.573
2976	992.5211	411	1.26642	1.2543	1.25755	204.978	0	2.508	53.6073	0	1.3772
2977	992.5236	302	1.26129	0	0	1046.43	0	31.8118	36.2444	1.31905	1.25041
2978	992.5243	332	1.25862	1.2582	1.25814	1108.94	2.81077	6.243448	51.2034	1.66442	0
2979	992.5249	362	1.33739	1.3392	1.33696	1207.09	2.68557	11.42474	55.8475	0	0
2980	992.5251	343	0	0	3.08117	1881.8	8.04784	9.961913	64.974	6.6404	0
2981	992.5257	320	0	0	1.32962	1926.04	0	21.28246	61.3557	0	0
2982	992.5261	376	1.25903	0	1.53129	607.147	0	2.521176	46.9378	0	0
2983	992.8129	87	480.821	102.48	470.519	492.998	267.697	300.7831	1749.03	1223.26	124.03
2984	993.523	342	2.82757	0	1.47617	2543.16	4.07984	13.53351	103.605	2.94186	0
2985	993.5236	320	1.51209	0	0	1762.73	7.99062	15.95864	58.32	2.656	0
2986	993.5246	376	0	0	0	569.422	0	2.521333	41.7327	0	0
2987	993.5248	302	0	0	2.90876	852.051	0	16.40708	30.7386	4.0216	0
2988	993.5274	281	1.25652	1.3386	3.79257	219.576	0	25.28356	15.0811	4.3569	0

2989	993.5562	112	1208.02	534.48	1313.5	1640.63	1157.46	289.4719	601.989	217.597	58.8012
2990	994.2567	71	503.194	339.31	504.395	392.406	219.584	310.6934	1142.29	1004.85	599.433
2991	994.5603	112	767.063	344.46	1005.2	1064.71	473.219	241.7792	415.976	94.1226	29.3811
2992	994.7506	83	582.832	305.6	664.469	478.766	609.858	341.6526	1364.43	989.871	303.457
2993	995.28	82	247.145	107.03	313.305	103.151	280.98	61.66364	1153.94	191.232	105.903
2994	995.7736	84	537.256	110.48	630.173	338.327	332.634	211.6162	1479.44	780.179	198.457
2995	996.2515	72	478.767	352.55	403.351	244.388	351.096	200.3204	991.162	634.429	587.82
2996	996.8214	86	4544.16	1587.5	3162.23	2346.82	1983.64	2409.035	12947.7	6308.97	2096.64
2997	997.2674	75	783.482	218.28	535.042	411.816	457.905	318.6435	967.198	1239.63	447.252
2998	997.322	86	803.412	218.04	533.687	433.029	458.298	339.2863	2925.05	1250.85	447.067
2999	997.8123	84	1429.64	547.29	1139.18	747.578	665.611	835.3158	3761.15	1914.76	547.958
3000	997.8233	86	1313.26	495.59	1101.89	737.291	646.363	801.2123	3041.45	1838.76	505.884
3001	997.8305	86	1345.56	518.72	1096.64	742.666	644.552	812.7042	4175.69	1884.93	520.165
3002	998.2514	72	707.086	509.68	507.363	317.649	310.905	337.057	1055	739.588	696.594
3003	998.8065	88	832.641	261.55	629.256	664.99	505.953	560.947	1307.14	1139.21	414.343
3004	998.8247	82	873.566	279.2	714.739	705.55	531.117	592.5404	1056.08	1187.82	454.94
3005	999.775	84	1004.29	330.81	824.618	696.46	710.462	502.9854	1930.03	1430.31	290.683
3006	1000.244	72	737.598	679.13	566.421	409.356	535.815	457.345	1077.65	952.219	898.275
3007	1000.791	86	973.854	285.57	885.902	575.968	660.86	619.5229	2677.55	1542.7	474.358
3008	1002.245	71	883.354	772.73	843.718	571.286	804.736	486.5261	1913.33	976.483	976.932
3009	1002.771	84	1704.16	680.07	1358.25	910.595	1150.01	818.976	3212.32	1731.82	768.964
3010	1003.761	82	806.826	228.93	819.341	677.569	632.532	404.077	1769.96	1336.43	330.951
3011	1003.829	86	748.287	203.96	717.823	669.035	618.407	379.7752	1675.02	1248.29	225.361
3012	1004.243	71	936.408	762.6	726.834	434.443	824.46	503.3327	2022.82	1030.45	884.97
3013	1004.773	82	1549.63	610.91	1462.22	963.96	965.43	946.3241	3769.22	1920.3	624.323
3014	1004.806	85	1302.08	575.02	1448.69	935.75	926.068	986.1938	4186.18	2046.97	592.692
3015	1004.814	87	1186.31	552.42	1102.96	844.01	907.034	842.0294	4107.33	2247.58	550.46
3016	1005.31	86	855.535	276.94	694.971	341.019	636.925	522.4978	2626.1	1231.15	295.104
3017	1005.765	80	255.889	147.63	270.3	186.973	257.5	212.686	861.84	474.715	141.225
3018	1006.812	86	565.728	240.75	485.934	383.785	548.8	279.873	1830.87	798.467	288.872
3019	1007.263	72	369.485	260.17	225.019	198.791	283.782	235.7993	1019.75	475.047	289.922
3020	1007.788	86	902.556	233.38	838.272	496.867	789.581	473.6069	2262.09	1050.37	390.901
3021	1007.848	83	911.692	293.52	846.803	503.31	794.55	499.281	2402.17	1076.19	433.136
3022	1008.251	74	846.418	439.23	730.902	303.9	616.987	476.8193	1271.55	973.241	526.744
3023	1008.789	84	453.281	106.47	491.954	328.573	474.011	175.8351	1962.74	738.446	102.926
3024	1009.765	80	404.003	137.97	409.107	344.569	300.433	259.7721	944.235	424.589	189.459
3025	1010.264	72	333.799	179.09	269.608	217.344	139.065	301.185	867.425	454.917	189.459
3026	1010.83	86	1064.39	494.06	782.403	582.297	450.113	633.6297	2652.66	1370.86	527.486
3027	1011.817	87	700.358	269.7	595.722	410.478	617.64	478.6005	1875.28	1320.59	296.756
3028	1012.797	85	3608.45	1429.4	3224.41	1573.3	2069.9	2066.136	7131.49	3962.7	1193.44
3029	1013.313	83	577.466	212.67	843.649	396.927	398.805	254.2393	1283.38	1205.09	285.218
3030	1013.796	85	1067.46	479.69	878.107	705.445	662.184	782.0155	3127.9	1615.2	612.694
3031	1014.248	72	544.291	321.73	309.063	215.236	263.616	282.9234	1193.72	635.529	563.805
3032	1014.792	84	814.938	465.6	886.47	554.656	535.333	536.1772	3107.39	1125.34	369.28
3033	1015.251	71	335.475	233.2	295.103	204.255	193.797	269.1398	1193.33	495.803	312.521
3034	1015.77	83	654.398	357.28	547.375	356.711	467.23	449.8894	1440.56	707.37	146.986
3035	1016.253	72	657.251	533.45	642.27	372.307	723.086	491.487	1492.87	918.254	633.588
3036	1016.773	83	876.035	321.34	840.5	350.403	450.353	526.3715	2231.21	801.925	404.233
3037	1018.23	72	541.717	350.06	408.747	187.04	262.132	164.4605	1219.27	583.399	634.736
3038	1018.779	86	566.438	286.25	367.88	280.375	326.765	345.5907	1363.05	755.731	173.316
3039	1019.501	342	2.888	1.3141	6.09876	2088.64	1.38055	17.8008	91.2483	7.25695	0
3040	1019.501	320	0	0	0	1059.64	1.39686	1.256129	19.5129	1.259	0
3041	1019.502	362	0	1.3203	1.3181	1275.36	1.3214	4.155103	66.5184	7.39373	1.30184
3042	1019.502	300	0	0	2.84576	1068.76	1.2586	11.32989	42.9944	3.97902	1.25165
3043	1019.78	83	594.164	177.15	523.503	378.335	375.444	371.7741	1056.61	618.32	250.26
3044	1020.501	342	2.83556	1.3078	1.48151	1815.04	5.45152	18.98475	94.0039	0	0
3045	1020.502	279	2.68618	0	4.06326	165.975	0	6.296522	9.74	2.8882	0
3046	1020.503	394	1.26311	0	0	189.228	1.25971	0	28.1274	0	0
3047	1020.508	301	0	0	2.87704	724.521	0	13.85831	19.2955	2.66531	1.25042
3048	1020.784	83	716.699	226.35	1028.23	380.876	638.032	371.7813	2043.64	1189.53	241.681
3049	1021.51	320	2.94593	1.3908	2.63738	1222.74	5.28987	18.48459	60.3212	13.2107	2.76365
3050	1021.511	298	2.52074	1.2584	7.15333	617.552	2.51011	18.89556	31.0161	9.31137	1.25076
3051	1021.785	83	692.965	249.22	537.306	326.847	386.773	257.7446	1345.06	618.549	240.171
3052	1024.249	70	764.706	523	563.684	414.96	632.455	444.1511	1084.84	875.992	671.444
3053	1025.8	87	602.212	246.18	412.927	428.016	378.16	329.6893	2435	1217.06	253.368

3054	1026.254	71	929.276	682.15	583.832	489.979	763.144	592.1645	1809.28	1075.72	784.881
3055	1026.805	85	1047.48	386.04	720.163	528.969	576.008	485.4572	3603.53	1718.04	405.527
3056	1027.801	84	345.903	144.41	543.473	241.405	367.098	335.037	1436.43	746.142	316.83
3057	1028.254	71	780.172	730.08	617.842	477.632	658.638	525.1087	2218.19	1368.24	908.333
3058	1028.769	83	1260.17	549.46	1773.37	759.518	1097	678.4919	5212.74	1810.73	735.289
3059	1028.778	84	1079.6	506.75	1536.42	739.153	1071.67	697.8462	3458.67	1611.01	644.36
3060	1029.769	83	766.415	375.69	885.059	391.573	585.814	488.7257	2677.71	939.051	333.03
3061	1030.25	71	897.206	871.58	625.652	532.261	835.662	529.1181	2039.09	1169.46	786.264
3062	1030.81	87	1080.55	381.67	1319.8	617.542	667.867	716.2393	3936.29	1582.54	415.754
3063	1030.816	86	1143.26	357.06	1055.48	658.5	624.13	660.0675	4307.89	1670.01	410.102
3064	1031.31	86	470.5	187.46	355.726	225.444	108.654	175.8546	2408.9	943.46	146.997
3065	1031.811	87	601.948	175.3	401.041	310.983	595.339	244.86	888.179	883.097	193.96
3066	1032.244	70	895.621	541.98	605.728	364.209	431.169	478.1755	1345.74	855.483	705.157
3067	1033.772	84	902.38	284.28	864.958	474.627	570.361	340.3972	2268.68	1314.84	291.288
3068	1033.791	84	738.768	233.58	648.757	354.801	400.706	260.4678	2124.44	1197.31	206.452
3069	1034.232	68	441.412	383.58	413.11	262.944	370.905	293.1286	878.651	630.244	476.832
3070	1034.252	72	558.562	415.72	459.42	324.515	472.527	403.2438	1206.76	798.646	513.345
3071	1034.311	87	358.524	207.07	453.756	271.149	404.804	299.3917	1368.72	582.505	175.548
3072	1034.787	84	624.762	138.33	531.127	259.337	405.418	414.5566	2586.97	971.43	365.133
3073	1035.288	82	222.698	112.95	356.046	122.041	354.948	142.3078	1497.98	334.522	243.99
3074	1036.241	72	515.194	270.3	295.103	197.58	205.743	261.1455	1185.33	660.631	365.716
3075	1037.78	83	777.581	117.95	701.578	408.477	318.143	303.4463	1137.19	835.744	141.808
3076	1037.82	89	422.575	55.093	349.633	286.241	315.867	110.8731	1261.43	686.299	66.069
3077	1038.236	71	340.736	381.86	334.279	229.448	279.765	289.0223	1252.76	613.754	470.431
3078	1038.796	86	1021.47	287.65	986.354	756.388	822.388	540.9101	4211.5	1763.12	376.594
3079	1038.806	85	1117.48	318.87	1021.93	774.315	852.022	598.0113	2676.42	1348.15	373.75
3080	1039.309	86	736.266	170.34	748.222	425.369	348.424	358.1308	2298.64	1155.42	346.289
3081	1039.793	84	666.407	193.38	441.115	310.557	565.565	345.4347	2037.46	997.46	284.141
3082	1040.231	72	696.113	540.19	436.644	452.226	570.137	331.3941	904.4	1045.37	815.945
3083	1040.804	84	384.272	204.05	308.955	392.663	380.142	196.5443	1440.83	718.48	119.372
3084	1041.763	83	977.524	208.49	844.009	470.901	426.697	394.9899	1712.11	1089.65	218.837
3085	1042.231	71	1162.48	960.89	729.809	535.001	807.789	587.3253	2370.51	1417.81	1162.35
3086	1042.788	84	527.116	309.23	698.679	260.252	482.329	280.6969	2415.63	832.242	295.687
3087	1043.237	72	280.864	206.79	241.252	171.093	173.648	132.6456	779.056	330.25	319.968
3088	1043.538	362	0	0	0	970.423	0	0	37.8439	0	0
3089	1043.54	343	0	0	0	1652.07	6.65464	6.981852	75.668	6.605	0
3090	1043.541	301	1.26041	1.2581	4.34819	812.469	13.8274	6.307755	41.9163	2.67635	3.75504
3091	1043.542	316	0	0	0	1261.07	5.4201	7.546133	50.0944	5.02509	0
3092	1043.779	83	333.364	129.43	555.858	130.976	327.733	295.6424	1681.71	443.272	171.058
3093	1044.227	71	1324.68	1223	922.716	636.398	1079.69	722.5889	2919.99	1977.21	1444.79
3094	1044.744	82	784.081	433.25	1260.91	442.015	895.772	575.975	1760.36	973.676	482.084
3095	1045.795	84	741.26	283.2	629.56	345.108	481.449	375.9923	2194.98	1005.67	246.475
3096	1046.225	71	1313.47	1074.8	789.558	760	849.972	615.734	3179.21	1664.31	1393.2
3097	1046.789	85	972.313	318.77	1063.83	483.605	607.347	716.4	3789.62	1259.92	354.489
3098	1047.294	84	609.438	207.18	557.652	243.008	417.749	296.948	1805.11	1257.15	142.907
3099	1047.805	84	552.912	249.47	661.14	292.656	614.626	303.7046	1589.58	759.404	80.6688
3100	1048.221	70	1042.06	814.88	663.455	604	694.706	470.0578	2164.63	1230.39	971.669
3101	1048.784	83	625.342	105.88	396.142	265.115	219.78	329.5474	1937.18	622.334	232.107
3102	1049.774	83	519.833	236.41	484.566	228.077	421.176	349.917	1826.79	506.883	222.833
3103	1050.217	71	552.469	483.63	493.185	295.035	354.409	405.042	1220	766.121	621.052
3104	1052.215	72	566.58	390.88	368.736	284	338.752	273.7837	1022.03	533.92	492.472
3105	1053.778	86	433.271	155.15	536.463	296.018	298.831	270.3006	1272.86	681.433	77.5775
3106	1054.768	85	1667.68	581.62	1523.66	916.709	909.325	861.5201	2827.6	1371.49	695.091
3107	1054.78	85	1865.49	515.81	1488.12	921.405	959.081	852.9878	3182.79	1887.98	639.921
3108	1055.282	83	350.029	122.47	423.919	123.977	190.365	281.5636	887.168	304.616	67.35
3109	1055.775	84	705.075	237.9	538.497	359.614	378.139	279.0971	1990.92	1118.6	357.61
3110	1056.21	73	565.211	541.01	522.063	260.414	512.125	228.7609	1336.25	778.497	696.872
3111	1058.205	71	883.12	690.7	610.318	356.21	823.635	370.3037	1690.01	1050.64	955.192
3112	1058.793	84	651.636	182	456.669	267.7	411.18	289.2365	1257.29	568.545	293.094
3113	1059.791	87	640.311	202.39	609.789	334.548	476.462	290.8801	1723.07	873.84	321.633
3114	1060.203	71	964.449	940.06	810.088	528.264	698.749	470.1607	1972	1088.59	1069.76
3115	1060.793	84	426.984	73.215	511.51	302.153	391.586	230.6243	1659.61	796.408	247.604
3116	1061.783	86	390.095	162.72	380.44	267.817	285.879	301.6137	1327.81	521.681	104.774
3117	1062.198	72	965.294	824.02	820.577	441.56	699.654	310.6909	1778.67	1179.92	1172.93
3118	1062.775	83	352.054	233.84	416.922	294.91	550.422	297.1376	2103.92	645.23	60.6914

3119	1063.263	82	273.951	73.667	439.261	151.565	247.122	109.296	946.992	464.543	84.9093
3120	1064.199	72	754.446	707.11	523.205	411.228	831.29	312.7653	1994.08	1040.07	860.741
3121	1064.808	86	3007.69	1275	2159.86	1620.31	1205.78	1671.726	8400.75	4335.31	1619.28
3122	1064.815	86	2912.23	1082.6	1976.45	1627.3	1277.31	1508.217	8214.44	4352.13	1446.85
3123	1065.816	87	1122.48	253.19	751.403	637.461	652.67	532.0475	1964.63	898.721	367.272
3124	1066.235	74	634.904	446.88	498.995	228.903	386.003	291.582	962.647	815.635	702.184
3125	1066.811	84	714.108	298.86	699.568	344.604	433.176	315.7195	1909.14	1016.7	254.388
3126	1067.782	84	750.988	273.41	569.315	309.055	461.294	360.7219	2669.39	1079.84	253.35
3127	1068.786	84	557.969	174.92	379.779	324.372	374.854	425.5724	2758.52	1146.42	181.396
3128	1070.753	84	1308.19	605.08	1073.73	684.794	842.12	697.5969	3401.93	1363.69	611.001
3129	1071.757	82	878.526	280.08	704.872	458.297	645.278	467.8281	2021.99	845.287	365.485
3130	1071.772	81	484.975	236.93	489.286	255.308	554.998	238.635	2220.38	756.394	347.851
3131	1071.818	88	621.657	262.45	472.797	309.3	642.098	400.8891	1716.65	843.804	362.092
3132	1072.795	85	1151.25	232.27	1075.89	648.122	627.036	727.6749	3876.05	1861.53	309.914
3133	1073.284	84	420.456	170.09	479.939	191.74	314.46	265.3051	1465.08	1204.89	184.026
3134	1073.797	87	468.148	155.39	354.544	146.6	400.985	174.69	1388.2	810.524	170.467
3135	1074.794	84	446.366	163.59	581.753	190.411	320.06	370.4268	1151.59	644.917	74.3058
3136	1075.768	84	851.649	270.44	721.706	493.693	628.635	465.5932	1588.9	1066.54	348.568
3137	1076.18	71	352.45	427.76	427.21	265.333	270.617	275.747	1255.13	694.41	527.966
3138	1076.75	82	721.156	192.35	388.278	310.696	487.5	280.952	1139.53	518.767	184.809
3139	1078.174	71	589.017	401.13	404.011	209.994	235.494	190.456	1144.25	645.819	586.466
3140	1078.819	88	833.463	238.48	479.853	439.888	437.282	524.331	1937.24	1135.74	454.713
3141	1080.176	72	428.663	310.05	343.613	273.675	215.035	195.8591	1072	541.955	442.848
3142	1080.784	86	2655.35	1083.5	2464.97	1368.9	1236.49	1510.04	5606.82	3104.95	1333.7
3143	1080.788	84	2422.9	1040.1	2441.81	1375.98	1220.01	1350.054	5068.92	2407.77	1113.81
3144	1081.306	86	523.413	195.32	500.026	221.118	252.866	213.8976	1800.29	648.872	114.33
3145	1081.783	85	926.475	324.17	825.924	673.331	417.123	485.1539	3477.97	1101.89	449.391
3146	1082.298	84	251.631	80.41	248.465	211.868	172.079	184.0094	1215.82	403.48	59.1066
3147	1082.781	84	861.91	400.99	775.198	359.893	549.364	538.187	2610.83	1232.6	473.586
3148	1083.758	83	466.429	267.82	611.5	422.846	461.052	185.3956	1378.96	633.437	189.053
3149	1084.194	74	232.728	233.64	246.364	125.725	173.729	196.2	856.547	379.916	342.785
3150	1084.75	83	727.77	179.12	728.973	325.928	541.871	444.2373	1354	660.883	237.319
3151	1085.439	89	71.6191	44.068	61.3446	11.1231	43.8381	79.02222	1697.66	86.8513	40.0455
3152	1086.744	80	729.537	351.85	784.818	444.564	674.975	377.0694	1240.26	805.474	436.449
3153	1088.774	83	478.523	261.63	668.93	473.542	422.121	371.5096	2457.47	929.633	240.348
3154	1089.27	83	243.728	150.82	243.827	134.704	157.246	215.2182	1823.59	422.793	58.4291
3155	1089.784	84	313.024	98.337	440.525	267.502	312.906	142.2056	1323.9	638.362	33.2274
3156	1091.806	82	407.174	79.876	422.805	147.566	229.38	166.6625	956.767	467.763	239.988
3157	1094.192	74	365.723	254.98	251.702	168.336	228.533	101.1507	986.824	415.391	296.568
3158	1094.795	87	766.015	366.14	681.089	453.573	488.712	423.5188	2045.88	1086.54	345.943
3159	1096.758	84	1307.63	557.06	1639.29	775.784	1162.04	752.8861	3171.5	1399.89	552.164
3160	1097.754	84	777.485	301.14	623.307	343.534	741.976	428.2569	2026.8	981.56	260.741
3161	1098.769	84	896.009	286.26	649.778	574.824	641.196	501.7579	2179.73	1533.45	379.883
3162	1098.802	89	872.695	254.8	602.376	559.196	619.942	503.7766	2072.58	1562.88	361.448
3163	1099.307	87	353.159	46.462	327.79	222.545	452.711	160.5867	1387.99	634.155	213.735
3164	1101.207	74	218.892	105.91	242.944	215.31	164.052	116.529	851.194	443.753	254.754
3165	1101.775	84	473.265	115.96	501.78	273.952	263.996	352.5673	1904.36	996.779	203.093
3166	1103.189	72	357.542	220.27	155.966	203.961	192.709	177.8956	850.317	530.523	378.809
3167	1104.185	72	351.384	328.85	276.545	232.464	219.767	239.8898	1121.78	609.857	516.553
3168	1105.78	88	298.235	13.578	297.338	87.9024	139.961	56.19412	1051.71	463.416	93.7128
3169	1106.167	72	385.433	184.92	243.107	203.834	148.155	242.3073	1090.73	549.015	395.074
3170	1106.783	86	702.603	159.89	723.601	358.411	427.238	460.52	2668.15	1392.02	203.204
3171	1106.793	86	820.074	168.71	687.2	385.545	453.758	470.6416	3441.51	1426.25	219.066
3172	1107.295	86	403.956	116.78	481.327	347.019	356.721	189.4692	1905.21	795.624	124.469
3173	1107.799	84	412.291	221.37	551.665	237.143	254.35	221.5067	1574.26	942.445	101.932
3174	1108.187	74	371.565	281.89	348.064	209.294	98.2897	229.5121	1362.4	485.973	393.166
3175	1108.799	83	360.987	80.352	295.93	153.442	185.807	171.5468	1014.73	729.298	17.5848
3176	1109.769	83	483.6	173.3	415.99	314.272	407.466	350.9403	2381.35	636.468	197.539
3177	1110.769	83	628.949	186.57	311.389	388.172	310.998	390.4629	2066.65	712.033	265.459
3178	1111.753	80	427.158	189.41	362.435	460.905	416.061	145.2647	1383.05	530.43	246.841
3179	1112.733	83	495.499	262.4	449.185	467.329	479.306	288.7351	1747.91	559.81	280.132
3180	1113.749	80	562.528	207.35	544.576	345.18	517.876	343.78	1048.27	913.086	328.681
3181	1113.811	86	457.932	110.8	476.094	206.641	325.132	350.5543	1400.61	921.008	119.899
3182	1114.777	85	752.159	239.22	877.764	472.562	501.501	477.5292	1788.35	1006.88	112.781
3183	1114.79	84	622.378	238.02	842.159	462.567	498.678	503.9221	1947.11	1043.15	112.157

3184	1115.787	83	467.112	129.93	427.761	186.154	401.431	164.404	1522.84	586.378	113.691
3185	1116.182	74	466.82	362.69	244.865	196.091	191.251	201.12	1038.92	521.705	352.837
3186	1116.761	82	580.737	234.78	525.466	273.321	444.99	291.9764	1137.63	641.603	266.864
3187	1117.743	82	457.209	222.71	543.086	367.514	637.94	293.9675	1370.16	708.9	205.205
3188	1121.799	86	49.2846	67.9	348.02	53.493	134.623	31.724	1058.76	342.874	39.9548
3189	1122.76	84	1025.98	451.84	926.19	560.885	731.612	578.4533	2762.64	1531.83	434.365
3190	1122.774	84	929.686	434.85	839.92	466.452	721.271	525.4264	2504.18	1427.16	389.274
3191	1123.29	83	192.596	101.52	185.18	92.61	393.529	178.4469	1379.72	442.507	129.703
3192	1125.745	80	389.082	217.22	359.551	304.498	361.609	92.08393	943.706	483.858	237.243
3193	1126.74	83	597.849	288.87	572.853	369.6	328.094	218.3279	935.246	652.156	220.393
3194	1128.766	86	312.304	88.197	270.578	214.447	198.811	111.7074	813.789	604.397	104.617
3195	1129.771	83	394.933	140.88	386.581	245.587	55.8498	240.0338	1266.13	382.023	12.5837
3196	1130.726	81	393.25	211.28	412.756	207.411	854.854	195.3464	1007.61	525.414	197.52
3197	1130.757	83	555.599	236.95	355.968	151.044	756.171	213.3476	1489.86	688.373	171.456
3198	1132.797	86	2151.54	686.95	1723.03	1077.5	1010.51	1014.981	5610.72	2793	1074.25
3199	1132.803	86	2003.83	681.38	1587.44	1001.91	989.573	996.8121	5769.01	2868.99	1044.38
3200	1133.298	86	291.477	111.76	213.246	203.685	180.306	157.1208	1395.84	574.256	183.56
3201	1133.791	86	711.727	235.7	501.605	305.502	300.469	306.5883	2462.49	952.872	334.292
3202	1135.769	84	552.161	194.8	474.006	356.091	578.003	395.6911	2252.84	903.837	189.941
3203	1136.781	84	462.774	169.81	484.216	246.847	348.087	440.263	1811.94	1109.56	80.5356
3204	1137.767	83	221.495	102.44	318.872	188.616	99.4425	81.03386	1372.43	464.347	61.7821
3205	1138.742	83	1022.08	459.56	1074.42	561.108	677.529	615.6515	2289.55	1113.8	365.335
3206	1140.782	86	816.145	214.34	672.449	377.317	509.346	453.2843	3036.74	1407.19	272.564
3207	1140.795	86	851.511	221.13	657.958	379.759	509.955	425.5546	3684.9	1376.06	274.495
3208	1141.281	86	296.506	104.58	467.695	117.315	170.224	115.1983	2014.16	862.307	110.92
3209	1142.756	83	481.863	160.95	415.431	309.825	496.872	257.8266	1265.27	711.359	253.139
3210	1143.787	84	499.941	112.14	565.514	226.096	460.751	413.5011	2323.43	770.576	228.336
3211	1144.763	83	489.315	214.31	670.952	358.914	366.885	287.9242	1217.97	345.42	138.038
3212	1146.813	87	690.634	260.07	341.292	178.839	254.384	315.43	1522.79	784.42	219.212
3213	1147.786	86	454.17	99.395	330.95	145.146	204.231	335.6891	1946.97	525.933	149.243
3214	1148.768	85	1896.66	766.24	1622.72	745.835	890.54	822.6164	3912.27	2249.27	738.914
3215	1149.286	83	363.299	102.44	321.497	196.013	71.0304	83.48943	1674.36	535.885	111.208
3216	1149.757	84	916.365	293.01	651.858	523.406	413.368	454.5859	2154.34	1370.7	333.076
3217	1149.776	85	850.894	243.17	604.821	500.261	387.236	420.8564	1898.84	1378.67	229.745
3218	1151.751	83	670.372	216.88	457.878	165.965	237.052	272.2458	1082.8	507.322	302.58
3219	1152.741	82	448.858	177.34	349.107	241.477	314.455	284.8	1464.39	525.31	156.433
3220	1153.764	83	161.451	57.941	152.901	12.2921	100.674	45.4844	929.396	346.258	18.5132
3221	1155.788	84	174.307	73.68	219.211	155.311	235.575	229.3361	1562.67	334.295	89.0023
3222	1156.749	83	554.417	294.1	807.893	392.746	344.541	361.1856	1654.72	846.253	163.251
3223	1160.723	82	356.244	174.09	406.113	216.351	165.657	181.2808	1417.62	318.168	133.889
3224	1162.152	72	477.47	248.91	264.748	149.184	149.827	165.0316	1195.16	470.49	366.776
3225	1164.746	85	877.007	417.51	1188.71	468.919	708.239	678.6383	2218.61	1211.07	399.932
3226	1165.756	84	535.148	187.83	539.176	300.52	406.889	399.6234	1548.35	665.172	210.364
3227	1166.784	86	456.98	225.39	479.806	197.842	368.014	287.4082	2300.93	911.194	244.173
3228	1169.769	87	431.717	127.87	412.645	332.019	281.422	287.455	1603.64	805.056	124.392
3229	1170.767	84	527.92	210.94	501.813	310.357	465.953	291.4951	1432.47	797.628	167.256
3230	1174.779	84	661.215	228.48	719.311	312.267	252.384	304.3773	2671.62	1072.74	242.551
3231	1175.265	84	410.105	100.86	260.388	59.7148	428.004	167.4784	1216.69	669.155	180.815
3232	1178.768	83	336.227	110.5	303.419	144.591	181.603	250.7001	1708.79	421.902	67.8922
3233	1179.737	82	256.256	148.39	272.667	194.167	130.179	120.9091	856.319	339.535	87.6436
3234	1181.758	83	486.211	206.42	464.753	288.544	509.23	280.9654	956.424	580.9	200.132
3235	1182.77	84	440.787	164.7	671.92	320.236	526.443	263.571	2080.71	904.136	223.541
3236	1183.276	83	314.56	83.97	442.907	124.24	172.423	146.3615	1320.23	582.651	117.96
3237	1183.764	84	309.193	141.42	467.878	121.33	45.241	128.0086	1941.37	408.883	129.214
3238	1189.782	338	0	0	4.48708	3110.87	0	0	118.841	13.4092	1.42012
3239	1190.749	85	814.884	411.02	764.789	417.109	648.9	610.6949	2975	1502.72	362.3
3240	1190.783	336	0	0	6.19894	2670.62	1.32432	0	70.0648	7.32582	1.37993
3241	1191.758	84	399.798	238.13	473.817	190.479	226.099	302.541	1375.97	623.935	187.807
3242	1191.787	338	0	0	0	2023.12	1.37488	0	49.8472	4.30914	0
3243	1197.745	83	324.072	163.96	309.723	106.942	335.58	113.1279	1432.55	465.568	65.4134
3244	1198.743	83	648.265	175.8	467.923	316.844	382.664	162.8973	1711.41	600.299	261.511
3245	1199.75	83	387.248	155.33	492.681	118.005	197.476	135.2869	2116.88	420.178	162.916
3246	1200.772	86	1026.07	427.6	1101.04	493.313	615.865	647.322	3252.41	1938.93	584.463
3247	1200.785	86	1271.66	410.19	1015.46	465.465	613.992	520.4089	3431.72	1911.75	544.208
3248	1201.769	84	675.052	219.29	423.669	348.217	248.523	303.4548	2115.75	809.173	374.836

3249	1206.729	82	596.649	280.12	607.171	315.726	212.677	374.2765	2106.6	683.46	219.141
3250	1208.767	86	597.382	235.57	489.501	315.948	424.58	244.9347	3245.53	1125.3	286.292
3251	1216.745	86	1242.81	518.17	882.304	526.753	743.204	608.968	2440.54	1280.73	544.881
3252	1216.758	85	1492.29	552.73	1125.73	648.343	935.794	708.6301	3226.66	1705.84	535.861
3253	1216.766	85	1339.5	545.37	1142.16	677.551	772.514	682.7336	2694.92	1523.34	554.68
3254	1217.752	84	534.641	226.18	588.958	297.478	284.898	376.1809	2241.33	799.34	282.085
3255	1218.771	84	389.359	72.52	288.791	88.7358	212.734	128.8161	1650.21	346.662	167.076
3256	1219.742	83	204.241	133.55	339.356	251.775	194.147	172.4018	1083.5	404.669	141.044
3257	1220.237	82	124.751	61.019	107.865	11.7885	43.86	56.63	817.821	206.455	49.5
3258	1223.758	83	232.998	168.07	340.136	190.189	153.899	157.2842	1149.03	382.964	129.474
3259	1224.731	82	379.488	187.74	363.666	222.873	445.671	256.665	1143.94	614.364	171.738
3260	1231.772	84	195.229	0	257.973	31.2338	112.66	84.58467	1024.83	255.4	67.4651
3261	1232.733	83	618.83	270.75	760.402	318.979	520.774	400.8724	2412.85	746.19	326.071
3262	1233.725	82	260.339	154.37	323.064	334.384	170.711	127.7195	1596.6	377.754	58.5121
3263	1234.77	87	587.048	130.55	486.069	262.12	349.5	219.8933	1622.94	973.896	220.967
