# Supplementary material for: Effects of Lycium Barbarum Polysaccharides on the Metabolism of Dendritic Cells: An In Vitro Study
Source: J Immunol Res. 2022 Oct 19;2022:5882136. doi: 10.1155/2022/5882136 (PMC9605842; doi:10.1155/2022/5882136)
Supplement: Supplementary Materials — Figure S1: the total ions chromatogram in negative ion and positive ion mode. Table Sl: the raw data of negative ion mode. Table S2: the raw data of positive ion mode. (Supplementary Materials). [file 5882136.f1.zip › Supplementary data (Table S2).pdf]

Supplementary data (Table S2): The Raw data of positive ion mode

ID	mz	RT	LBP1	LBP2	LBP3	Ctrl1	Ctrl2	Ctrl3	QC1	QC2	QC3
1	53.007	47	15005.6	14662.15	16712.06	11920.6	13560.83	15638.24	3533.487	11107	17567
2	55.053	1044	24694.5	22849.23	22576.78	20718.81	23967.85	31183.09	24131.25	22882	24720
3	56.049	1041	9887.03	13364.27	9579.772	7506.349	9879.738	6224.542	8995.669	5898	9713
4	57.069	104	4273.31	4947.246	5838.922	2364.51	3699.505	3930.556	3998.247	4017	2507
5	58.877	65	854.331	1507.627	2029.563	1264.634	1045.501	900.3683	1452.203	1099	1639
6	59.048	472	24307.4	22101.93	36270.03	23579.12	33240.19	27125.81	22137.31	38737	40727
7	59.049	1043	38122.1	35100.53	35281	28674.91	35173.92	36697.12	40479.31	38710	39071
8	59.06	232	66887.6	63274.82	75142.76	67099	56939.57	35664.77	55923.16	70630	69128
9	59.06	159	63826.1	58361.82	60754.68	54473.16	74957.59	57461.69	68639.6	63144	62008
10	59.06	61	239712	172730.5	283731.2	182612.2	253746.2	284862.2	199207.2	3E+05	3E+05
11	59.06	21	54258.2	83288.88	42153.53	48452.84	53388.9	49796.82	41784.9	52233	83338
12	59.06	1181	64553.4	95102.83	55773.37	60526.52	35978.34	61595.41	40334.22	66863	68509
13	59.06	1061	54911.4	51442.16	57233	44085.86	62572.64	58621.12	51276.49	52616	59816
14	59.06	252	41963.2	40624.94	45415.18	54785.87	45589.04	39758.3	36071.16	43390	48867
15	59.06	183	63056.4	61842.25	19148.81	55251.74	67890.8	61231.44	43047.25	64247	65680
16	60.044	1047	182123	297729.7	190507.8	162128.1	188293	199778.5	195484.8	2E+05	1E+05
17	60.044	224	67480.3	57764.89	67399.48	107192.4	76501.71	65091.71	56546.36	69339	65049
18	60.044	45	70449.8	87856.13	79443.82	65058.3	77913.55	71135.41	21005.93	83189	97313
19	60.044	16	50123.5	63029.56	764.8042	56922.25	59660.78	81382.22	46488.77	59770	71982
20	60.044	1179	105818	95775.42	93921.67	82532.33	93626.37	105863.8	80564.32	1E+05	1E+05
21	60.044	1096	3518.37	72623.96	85528.28	73572	78850.71	77593.64	85060.31	77802	80341
22	60.044	178	80046.5	76273.45	65704.92	71570.56	80223.62	81799.35	69759.42	83486	82395
23	60.063	61	5945.15	5723.959	5545.138	3107.683	4683.247	5456.452	4125.956	3670	7127
24	60.063	267	1372.78	1470.315	1379.413	674.7817	1281.502	1495.603	1144.864	1457	1307
25	60.078	117	609.073	1487.835	1242.715	1166.985	1215.515	1379.704	1443.773	1564	1554
26	60.08	1066	2337.19	699.2425	2172	2099.832	2148.956	2871.745	2329.458	1158	2476
27	61.046	167	27256.9	27734.34	25810.27	25136.58	27344.22	26765.25	24030.68	30002	28090
28	61.064	70	1067.3	1099.828	1050.908	1826.744	1587.422	2240.502	1209.289	1230	862
29	61.064	1045	1730.62	2064.876	2053.125	1795.213	2263.98	1502.687	1741.026	1603	1698
30	64.015	73	53966.2	30545.2	40118.04	22267.48	29091.53	17279.16	16690.07	44003	31772
31	69.043	1056	2919.38	3531.893	3158.008	2715.098	2167.185	3272.472	3220.56	2668	3426
32	70.064	1141	1416.05	1434.284	1265.687	1318.68	1205.962	1365.947	1171.655	1284	1513
33	71.951	1117	1892.8	2946.436	2224.195	862.6914	2418.442	863.958	1781.225	3574	2103
34	71.951	953	2580.99	1253.921	1295.645	1248.718	1184.978	841.4804	246.006	2008	3087
35	71.951	1000	1514.27	1236.728	1151.239	905.1554	1102.967	1150.514	1050.07	986.6	1300
36	73.064	1169	18963.1	15609.77	2837.688	13133.16	13635.6	13334.57	11621.35	12941	13427
37	73.064	1044	26309.3	21227.92	20851.58	8521.564	21047.49	27354.44	21052.96	16290	24637
38	74.095	5	23829.3	21952.41	3717.499	16271.27	13597.42	15276.72	7924.952	11167	26199
39	74.096	934	106323	100175.7	110557.7	91253.47	414.0024	169190.9	30979.43	96520	1E+05
40	75.099	1015	3271.14	3038.156	3289.817	1078.44	2859.238	10449.31	2299.077	3066	3473
41	75.099	986	2346.47	2433.831	2639.143	2143.474	2128.126	2098.162	1424.67	4758	2908
42	75.099	958	4852.57	2827.735	2085.988	2647.729	2510.86	2181.58	388.1448	1412	3265
43	75.099	931	6275.73	2254.749	1682.098	2002.922	1917.948	1531.916	1726.059	2557	2435
44	75.099	44	1897.93	1352.786	1535.298	1570.887	1740.665	1864.615	1351.543	1821	2017
45	80.947	69	101226	63666.66	80581.4	44060.69	49571.17	72113.8	77703.88	84252	77608
46	81.03	70	3988.38	1270.446	5313.446	2967.315	2237.608	1672.302	2704.685	2457	3319
47	82.944	69	34445.8	25776.61	29482.91	12395.66	20769.28	28591.77	26743.67	25750	35850
48	84.043	106	349.018	725.824	357.4613	779.4242	727.175	886.1067	681.338	824.5	747.8
49	84.043	249	3653.19	2487.009	6406.181	2970.665	2971.038	2651.92	3437.971	3711	3355
50	84.08	1043	2715.48	2899.285	2281.891	894.24	2450.5	3243.314	1801.691	2257	3461
51	84.08	72	3971.3	4654.434	1100.509	1653.144	3614.036	2825.894	3227.189	5634	4335
52	84.951	173	5543.67	5243.59	4969.369	1854.736	3163.272	5240.231	5163.589	5460	5776
53	84.958	1163	3552.44	2861.229	3505.189	2152.548	2475.414	3713.465	3092.219	4242	3368
54	84.958	12	1162.46	2029.759	1675.826	2840.297	2588.081	2802.509	1222.412	1819	2768
55	84.959	1179	3200.1	3653.132	2873.2	3615.525	3445.593	3365.28	2993.372	3769	3186
56	84.959	1125	4958.36	1501.297	6094.558	3237.979	6247.179	4868.571	1657.83	5779	22667
57	84.959	1085	5258.7	4698.955	6078.51	4968.867	4737.145	4916.63	5773.468	5033	4747
58	84.959	1072	3431.66	5476.544	5152.14	4812.81	5457.87	4778.071	4135.832	4371	5315
59	84.959	954	4801.12	1634.688	5394.643	515.458	10619.14	2647.26	4243.839	4703	35020
60	84.959	235	2327.11	2088.049	1897.124	5399.386	2412.162	1208.909	1824.637	1897	2165
61	84.959	214	2740.15	2295.399	2063.008	3058.438	3022.313	2278.154	2091.388	2196	2594
62	84.959	1109	6541.36	4580.84	7127.172	5094.509	5040.076	5728.417	5080.085	11649	5726
63	84.959	983	4609.79	4426.661	4648.14	4259.845	5073.368	576.7284	4417.034	4444	216.3

64	84.959	54	3720.26	2403.76	3087.605	2597.552	3881.028	3189.404	3554.181	1758	2836
65	84.959	346	408.111	504.0648	337.722	384.1933	379.4948	471.9189	450.468	399.3	528.2
66	86.095	62	3762.16	3185.417	6823.004	1532.434	3209.551	2565.607	3889.558	4055	3870
67	90.054	106	2626.92	2776.021	3467.084	2109.246	2783.511	1350.555	2335.868	1957	1650
68	90.054	70	2141.1	1717.67	2278.063	2480.169	1824.259	1275.412	2891.369	1342	2534
69	90.975	84	82943.6	68841	66444.02	55566.23	50542.72	35183.35	66446.95	44347	56773
70	96.079	1069	3394.38	5373.06	4316.555	3665.304	4744.359	3847.2	3139.988	4296	6051
71	96.921	71	25436.2	14503.36	22253.83	10385	9936.416	19034.87	18059.17	20692	19659
72	97.075	1066	1273	2292.801	869.9091	1628.913	1377.747	1686.853	1510.676	1367	1933
73	98.096	1070	3068.82	3823.496	3086.274	3079.411	2752.829	2935.871	2757.621	1670	3980
74	98.917	71	11555.8	7213.092	9349.051	4529.986	3764.362	4483.35	6665.605	7932	6500
75	98.974	1006	5637.62	3844.089	6781.378	5970.265	3877.143	3747.675	1091.402	12872	4041
76	98.974	967	8355.2	5222.788	10534.89	3253.022	27732.1	7164.616	7538.82	8278	8694
77	98.974	948	4735.75	8347.692	10980.21	3496.112	6723.298	5880.213	16921.87	4460	9186
78	100.11	49	4372.34	1928.181	5215.254	4607.847	4518.579	4913.532	4893.581	5378	4098
79	100.11	117	1602.1	5487.181	4987.998	5165.239	4022.974	4133.254	4790.27	4238	5264
80	101.07	1069	2331.88	2656.7	2309.566	2316.201	3996.79	1073.95	2610.875	2244	1336
81	101.07	147	980.04	1316.027	1105.255	1276.714	1250.809	1275.495	1069.843	1160	1554
82	102.05	245	2569.78	2123.283	2409.366	2621.569	2574.456	2120.863	1875.243	1762	1033
83	102.05	104	3041.49	1645.515	3347.689	2881.993	3077.027	2353.707	1837.658	1143	1587
84	102.13	904	3885.15	4181.863	3611.533	2923.106	3337.617	3203.005	3061.27	3388	3922
85	102.13	941	3340.69	2896.434	6668.011	2819.724	2896.906	2809.702	3019.008	1894	3609
86	102.13	989	3657.81	3455.674	1280.689	3458.768	2469.814	2896.916	2900.704	7257	3751
87	102.13	116	2566.44	1882.603	1097.19	2061.948	2239.828	2351.018	1740.065	1728	2152
88	104.07	69	79292.6	5256.547	97985.89	2115.578	6537.187	2201.512	2005.164	53162	4196
89	104.11	95	13570.7	1724.031	18138.22	13183.21	2321.88	11590.26	10633.92	6334	11762
90	104.99	84	3707.81	2771.662	2566.538	3064.271	4093.74	1689.822	2714.171	2032	3197
91	105.11	61	4910.52	1466.42	4344.16	1791.769	2448.482	2943.217	2743.965	1341	4145
92	106.09	70	4481.63	9757.979	1247.554	1356.305	4700.709	2363.274	6204.904	5306	4593
93	106.95	81	6371.84	4255.055	10017.05	2817.36	5035.255	4420.667	3923.621	4480	4901
94	107.96	62	848.75	812.3086	1252.354	1150.68	1736.657	943.2941	1415.21	987.7	1481
95	110.01	815	2560.55	2091.541	2191.31	2253.6	1801.323	3721.987	2739.196	2902	2744
96	110.01	885	20418.4	6736.686	16422.44	12750.05	17488.46	22834.95	8433.999	12047	16307
97	110.01	850	2433.66	2808.017	3328.612	2475.066	2811.79	2683.08	436.4138	3653	2867
98	111.01	879	919.329	287.5253	989.128	498.8327	1006.653	950.1323	993.5878	980.5	1150
99	111.09	1186	49321.3	51910.63	36080.04	42456.47	43035.17	47680.18	33482.75	48038	53064
100	111.09	1081	180979	63341.6	69088.85	56049.51	57884.88	168543.1	65530.18	59502	77750
101	111.09	102	104294	57856.51	61809.74	44162.98	48346.25	57611.95	30625.56	61132	64698
102	111.09	1063	43575.9	38881.04	49352.59	29631.18	39602.08	41314.55	44324.53	47807	40414
103	112	862	320.832	348.0588	505.2443	313.7188	358.5212	1303.986	363.5529	397.1	350.6
104	112.01	886	779.529	826.5693	1002.669	929.5573	4143.647	826.7474	957.4754	969.4	979.9
105	112.09	1185	3379.02	3555.236	1770.899	3240.625	3375.706	3406.313	2528.255	3547	4115
106	112.09	1061	2830.25	2590.088	2453.491	2345.506	2089.81	2362.939	2316.244	2893	2962
107	112.09	1079	5998.81	5201.197	14880.1	4948.058	4836.415	5566.844	4461.689	14358	5867
108	112.89	73	17075.1	5060.23	13921.76	4980.274	4110.872	9651.531	10508.75	10879	10383
109	113.11	1071	73891.5	26854.79	27569.85	25086.11	24696.21	18926.37	18517.55	20451	34064
110	114.08	118	4758.09	656.53	4352.577	3953.737	4344.44	3973.145	3462.529	4907	3368
111	114.08	2	226.115	1205.9	1242.714	865.6554	1199.489	998.7799	940.8849	1354	916.4
112	114.09	66	8018.66	14262.72	8299.08	9321.144	8271.568	9623.313	8155.998	11497	10029
113	114.11	100	941.755	1278.331	6806.917	5130.573	6384.458	6859.438	5181.425	6844	5881
114	114.11	1076	1968.96	4322.508	5918.126	1495.076	9889.605	1207.8	1727.769	1515	7675
115	114.89	72	11494.3	3628.871	9304.597	3504.461	1787.028	7854.536	4328.274	6002	6022
116	115.12	45	13756.6	5635.947	13863.57	11157.88	12081.8	14047.91	10429.74	14556	13138
117	116.07	65	13245.2	3995.983	14741.6	4913.127	4329.305	5776.968	4600.821	7022	4014
118	116.11	858	1340.72	1393.004	3191.16	1172.043	1402.583	994.7128	1072.827	1302	1475
119	116.11	837	1061.25	963.9002	1424.075	1139.677	1068.773	1142.513	660.2911	1054	1200
120	116.11	810	987.514	763.2126	697.7958	1049.913	921.1517	1354.819	991.3084	1055	1203
121	117.08	70	2955.02	3373.24	1914.952	1731.136	1195.174	1922.999	3096.586	3364	3750
122	117.11	107	4198.43	3047.99	5451.909	1461.6	4478.545	4993.117	3684.217	4131	2575
123	117.14	959	1086.53	691.077	797.5227	587.5388	603.9276	535.158	535.4821	688.1	848.2
124	117.14	933	1727.76	436.2593	167.5575	396.64	394.0132	483.472	363.7384	823.4	569.9
125	118.09	251	5599.28	4155.127	5467.707	4264.629	4330.172	3994.361	3635.104	4726	4513
126	118.09	60	546807	328050.1	548002	76378.12	119384.5	121792	241672.6	3E+05	3E+05
127	119.08	79	24347.4	27831.8	28431.78	29671.44	23219.31	17454.49	20238.82	20683	20027
128	119.09	60	23654.8	23318.27	35389.99	6477.938	7808.972	8827.932	16558.63	19175	18031

129	120	92	12337.5	4774.538	10631.67	4540.419	6477.768	7915.974	3671.244	8041	6105
130	120.06	108	6349.97	2520.749	5408.515	4470.611	2951.098	3214.816	4243.668	8487	5898
131	120.08	68	1489.39	2131.949	5821.724	4603.528	906.1	1655.351	3501.575	1185	4194
132	121.03	1072	1782.38	1426.194	1319.476	1478.654	1218.964	1416.976	1554.072	1414	1234
133	121.03	106	4696.76	3470.992	5622.918	2341.08	3422.728	4205.016	3463.952	4442	4127
134	121.04	63	1778.73	934.752	1796.7	1084.547	1618.889	1314.782	1067.691	1192	946.9
135	122.08	95	9091.26	14032.3	9817.249	33917.51	42989.81	15381.13	14333.74	17790	9202
136	122.08	72	12867.5	28566.01	15265.02	26528.33	81746.43	15676.5	31389.72	17492	18239
137	122.1	1193	5484.69	5542.097	5438.021	4380.073	5796.808	5366.584	4591.444	5688	10025
138	122.1	1046	12750.2	8391.88	11923.48	10374.74	12020.02	19694.02	12330.57	12401	10130
139	122.92	81	2916.49	2553.522	8311.274	2823.094	2872.123	4407.189	2274.521	3949	5130
140	123.05	64	14190.3	11800.08	16668.15	8978.246	12356.85	12497.91	9770.353	12979	14432
141	123.09	1070	3087.61	2876.319	2031.59	2837.868	2925.24	1314.198	1689.088	2923	3168
142	124.02	942	8339.27	3128.529	3491.397	1981.905	2699.059	2864.01	3289.394	2868	3696
143	124.02	1022	4103.8	3911.357	3737.646	4010.572	3596.397	3921.658	3720.527	3893	3758
144	124.02	797	3485.11	5057.287	7063.255	3430.651	6798.986	5261.227	5764.981	4957	6156
145	124.02	974	3000.56	2183.821	2176.563	1557.949	1928.325	2212.755	2543.166	1411	2327
146	124.02	914	7627.66	7077.011	6005.839	9869.836	13253.23	6983.392	10550.58	3549	7506
147	124.02	838	4688.59	2991.991	9149.484	3726.049	5371.247	4914.824	3806.03	8173	5390
148	124.02	820	4446.45	4046.586	3778.837	408.91	12878.01	4316.297	3904.355	3999	4856
149	124.02	777	4433.88	3497.083	4970.702	3421.465	5038.169	3669.767	4155.597	5126	4501
150	124.02	762	9117.93	2258.429	18509.21	6998.202	7725.59	7777.152	8910.721	9999	9600
151	124.02	884	53973.9	27364.41	4543.773	29183.64	5695.686	6768.909	20981.05	27970	1065
152	124.02	854	3375.57	1937.044	4160.608	3345.686	7943.091	8593.538	2428.71	6219	3949
153	124.06	65	17268.6	2811.466	1412.156	785.355	746.1415	858.7644	2593.633	1864	1241
154	124.09	1061	54061.3	25189.4	28861.54	39206.11	48023.19	58775.7	27091.98	31101	73621
155	124.09	1189	2121.15	15684.11	17048.71	24537.25	21294.67	35704.29	23675.86	40949	2877
156	124.11	1162	2540.1	27540.23	26889.25	19930.9	25087.49	2300.6	21205.51	2680	24965
157	124.11	1125	25172.6	3681.476	4399.616	17850.96	2851.988	16624.06	22041.79	25950	26744
158	124.11	1106	10039.8	2891.495	28313.31	25374.82	2808.696	32251.3	1911.937	27277	4188
159	124.11	1077	28117.4	3285.36	3707.319	2591.293	2947.764	3141.39	2014.823	2995	1822
160	124.11	471	29598.1	29546.96	34397.68	4187.246	30442.23	34003.38	23160.14	33021	33512
161	124.11	112	42738.1	4509.094	42656.45	39648.43	3118.707	3567.859	24681.98	7609	3273
162	125.02	867	927.502	1182.667	503.8841	1052.256	1316.532	1222.988	1383.39	1281	1990
163	125.02	849	2338.24	1287.713	1184.957	1150.244	1346.169	1260.398	1086.933	1348	1167
164	125.02	886	1995.99	1756.294	2010.879	1693.618	2047.419	1896.141	1905.657	2128	1271
165	125.06	72	2039.31	1495.728	895.0286	1596.51	679.9747	1500.629	1484.82	1229	2001
166	125.09	105	1611.51	2820.255	4021.947	3894.41	4130.808	2053.68	1130.92	2289	1749
167	125.11	122	5061.18	1406.592	4126.018	3450.763	3475.24	4625.93	1037.143	4670	2268
168	125.11	1066	3702.1	4694.692	2162.543	1014.617	1310.802	1801.067	1632.015	1981	2459
169	125.98	470	2636.99	2462.703	4782.549	4550.26	3472.325	2881.322	831.7329	2727	3301
170	125.98	216	4960.37	4030.685	4795.044	5347.44	5020.87	4620.033	4096.685	3935	4427
171	125.98	1073	13270.7	15584.4	12622.1	10388.56	12134.52	10890.85	9256.327	7637	12566
172	125.99	1106	36778.7	14047.07	38671.11	11013.07	11681.9	12090.26	13539.05	15571	14781
173	125.99	957	15387.5	14174.73	37824.01	11894.18	13859.2	8270.938	15942.73	14594	16367
174	125.99	22	5726.51	8485.568	4191.677	6255.931	7939.644	5658.969	1589.612	4923	9586
175	125.99	1185	6286.08	7762.367	5176.514	5899.014	7311.454	7504.393	4408.6	7509	7569
176	125.99	1153	8539.22	9345.047	7350.492	5506.7	5451.97	9253.173	6119.165	8663	7046
177	125.99	990	9593.73	9292.725	12401.44	9111.981	9052.064	5182.223	9442.074	9337	9476
178	125.99	1125	11172.8	14008.53	12573.33	9060.653	14368.37	11070.13	12199.36	11963	14452
179	125.99	109	4624.03	5348.332	3679.383	4909.023	4807.26	5192.035	1172.287	3925	6078
180	125.99	160	6144.12	3195.638	5038.881	5662.837	6662.619	5556.533	3907.033	3512	5892
181	125.99	123	7059.71	5800.903	6436.979	6082.187	5617.126	6664.884	5403.255	1349	6981
182	126.02	60	19786.7	5594.085	26268.54	10736.13	14495.03	14311.33	14415.7	18078	4026
183	126.02	917	5253.65	5268.564	683.5015	1146.035	983.4111	938.2725	11755.69	4720	5583
184	126.02	829	1573.43	1338.673	1401.786	1362.669	1525.096	1377.924	1200.416	1429	1626
185	126.02	850	2246.24	2062.486	2327.055	2105.659	1263.308	1012.067	1655.903	2175	1178
186	126.02	813	1062.52	801.456	882.0998	932.7267	887.3063	1066.64	1050.041	266.6	1046
187	126.02	871	1712.83	4399.808	4626.977	3611.844	594.3086	4318.923	4874.412	1841	971.6
188	126.02	79	15664.7	24350.06	15359.96	15000.07	13316.8	17058.43	6531.77	10999	28694
189	126.02	895	4283.04	9882.669	2762.686	10875.38	230.048	3625.733	4659.636	3802	10951
190	127.02	807	730.145	675.8981	883.1025	784.6926	655.3581	809.9004	767.401	798	791.4
191	127.02	873	1921.06	656.7835	1415.544	412.02	716.7816	852.1595	798.1146	822.6	602.2
192	127.02	838	532.955	471.2114	726.24	573.7793	1794.809	799.8877	382.497	185.8	445.1
193	127.07	1053	21189.6	26640.29	19836.17	12045.01	40168.48	38174	15364.38	20033	62536

194	128.11	478	2277.12	2125.731	4679.745	4083.236	2817.339	3017.941	3248.405	3702	2925
195	129.04	843	672.798	567.385	763.425	679.288	689.3174	969.8568	449.6214	741.4	697.3
196	129.05	65	1411.16	2354.1	4274.213	2100.717	2634.668	1489.947	2071.422	2425	1546
197	129.06	111	1657.23	611.534	659.0029	1197.864	1196.119	1273.771	1625.921	1510	749.8
198	130.05	283	3294.46	3164.348	3725.28	4113.241	3421.987	7866.365	5560.418	7854	6045
199	130.05	261	3755.25	32377.42	3240.567	9122.978	3367.406	4227.524	3540.725	3315	43716
200	130.09	477	2028.54	2176.596	3409.869	2603.257	2969.406	2613.668	2856.452	3166	1040
201	130.09	307	5764.16	2982.064	5275.417	3035.96	3898.251	3263.708	1137.554	3504	2331
202	130.1	236	3978.27	20671.83	4153.734	9979.841	5763.038	4127.54	4713.678	13615	4227
203	130.1	121	1344.41	888.5067	3969.889	5783.763	1370.88	4994.815	13923.55	11100	4535
204	130.1	10	3136.45	1601.82	2630.897	1650.862	428.2325	5830.844	2879.13	4692	2167
205	130.1	156	4592.46	5067.119	5011.02	5217.147	6261.773	6025.518	9071.371	5221	5089
206	130.1	1073	2306.86	11893.47	4460.728	17197.19	6505.683	5534.55	5333.283	6315	16077
207	130.1	191	4752.94	4489.684	4064.112	5235.147	5412.251	4982.441	3851.645	5465	3872
208	130.1	1188	3941.72	5979.352	2703.321	4027.967	3948.129	5737.036	4448.64	4745	4289
209	130.1	1108	3922.53	3804.002	4574.08	4784.67	4808.517	5505.984	4836.894	5177	3936
210	130.1	64	3800.92	5490.776	1595.571	3308.539	4126.116	5504.162	2624.317	3655	3534
211	130.1	1159	3857.36	3980.777	4572.479	4461.552	5091.334	5174.725	4479.423	4580	3792
212	131.06	126	1755.72	2487.464	1738.289	707.1833	2660.146	2190.648	2497.258	2734	1929
213	131.08	979	947.23	985.1073	720.01	833.9261	176.6688	916.4212	872.2611	907.1	964.4
214	132.08	97	76802.4	925.4196	120662.3	21041.09	37317.44	36894.8	37216.78	52194	46542
215	132.1	69	177720	69853.62	139001.9	38555.91	41144.57	87730.01	68981.08	1E+05	1E+05
216	132.15	1112	1733.55	1259.424	3631.137	1112.435	1334.667	1326.75	975.5419	1480	1652
217	133.08	71	10266.7	2018.54	5966.593	2847.149	2750.935	4291.746	3572.783	9367	3580
218	133.1	118	6987.04	2093.867	5513.75	2408.158	2847.977	3909.758	4999.02	4501	4907
219	134.02	95	11331.6	654.588	19510.2	3583.153	9798.943	9965.3	4417.6	8142	3278
220	134.08	68	768.056	935.3564	1367.045	1515.005	1296.974	912.0136	625.6219	1380	1238
221	135	63	16575.5	8342.615	15424.27	9133.456	8826.937	7594.375	9040.845	8530	12966
222	135.08	78	13736.1	10330.86	7677.683	12844.75	8415.115	10299.87	8160.3	9854	11185
223	136.05	98	13436.1	14149.67	24620.35	3195.836	7470.485	9008.168	1235.367	15527	7562
224	136.06	63	1510.32	2862.208	1289.871	1289.775	2466.289	2832.587	2052.094	1538	2400
225	136.11	2	523.914	543.9757	618.4629	423.6725	536.8278	553.3263	454.23	559.3	357.2
226	136.11	44	3907.27	1550.64	5364.188	4214.297	3634.363	3821.211	3253.637	3680	3643
227	136.11	1065	6058.6	5825.186	6736.688	5725.676	6217.2	6015.677	6065.47	6343	6165
228	137.05	62	44532.5	30564.61	57814.65	3203.676	9606.448	10313.13	17171.65	16530	13111
229	138.09	68	9193.12	9633.028	6701.383	9139.992	7579.413	10598.24	8661.299	5557	1662
230	138.91	70	13592.1	11050.97	11362.33	5479.711	6059.971	10628.06	11249.68	14417	11868
231	139.05	65	2079.54	1483.149	2936.421	2137.02	2134.781	2212.464	2255.932	999.6	2756
232	139.12	91	83087.8	84393.93	94156.24	62950.7	75747.21	76724.9	69664.38	2E+05	61065
233	139.12	850	19265.3	19874.61	20231.6	17529.37	20980.77	18055.27	1535.04	19287	39712
234	140	2	0	1380.423	0	1468.347	786.6	1145.197	527.9239	632.8	1706
235	140	993	9386.9	8637.333	8441.001	7956.511	11279.92	6107.788	5857.147	8776	9227
236	140	969	17263.8	16165.29	14316.65	10632.92	9628.224	12985.21	12232.49	15401	14528
237	140	1012	50938.4	9027.926	48674.11	44079.56	42498.13	29258.42	6801.081	34577	36586
238	140	927	6327.83	6133.191	6222.374	5625.404	5660.079	5169.602	5711.26	5534	6588
239	140	948	17278.6	37999.5	12275.96	13373.34	10672.72	6939.957	4243.786	16137	33772
240	140	1165	2560.02	1300.209	9379.971	1247.546	2054.164	1936.75	1677.657	2289	1433
241	140.07	92	6036.11	5545.61	13549.3	1627.615	6562.6	8382.739	8345.075	8069	8016
242	140.07	62	40583.6	25359.21	22158.13	4972.51	6329.986	6887.097	13207.4	16236	17944
243	140.12	124	3809.29	4188.097	6584.639	2307.419	4531.767	6962.267	4607.82	5674	10095
244	140.13	1074	9214.53	11123.69	7982.52	20510.85	9060.555	7965.184	7097.171	7973	13487
245	140.13	1186	5805.18	8550.642	7129.321	6841.929	0	0	0	12965	11487
246	140.9	70	10781.2	5325.276	7592.276	5533.14	4453.8	7835.98	8009.073	9267	7971
247	141	982	1478.78	840.141	1565.366	710.0617	1230.449	1199.96	1174.518	1518	1013
248	141.04	856	1294.01	1130.935	1138.449	1181.743	1307.494	2014.793	1339.746	1501	1468
249	141.04	911	1662.94	1833.967	1505.656	1687.034	1894.953	1588.107	1846.167	2355	1890
250	141.04	880	1942.16	1547.526	3440.753	1764.723	1376.55	2380.112	1230.839	2639	1807
251	141.04	817	578.972	633.3734	865.2137	509.9815	588.5741	921.9335	709.6327	748.2	771.7
252	141.04	836	1226.06	1075.654	654.3244	1324.624	1228.513	2516.129	970.6021	1229	1331
253	141.09	62	3589.72	2162.42	1554.45	1097.277	16337.59	5287.632	876.631	1285	1077
254	141.11	477	14679.7	13818.49	1147.5	13549.2	14508.78	16642.13	10300.22	16878	16750
255	141.14	110	1846.8	42928.38	97572.04	30635.76	36680.62	26665.92	24242.88	73612	35521
256	142.03	894	3077.79	1732.002	2133.896	1939.54	2138.923	2193.166	1903.539	2295	2258
257	142.03	914	1359.05	2066.616	2148.848	2174.636	2236.199	2088.231	2200.57	2101	2279
258	142.03	867	854.195	856.7562	938.1702	767.383	899.4375	851.1659	945.5659	928.9	871.6

259	142.03	843	591.51	497.6864	634.942	713.7651	550.188	1177.668	554.6556	637.6	581.2
260	142.09	66	6176.31	12177.04	6072.085	4344.904	7483.735	7062.448	6740.272	7642	8637
261	142.12	72	3372.64	2826.696	4111.83	1944.45	1673.25	3527.017	2669.006	2313	2404
262	142.14	114	5512.42	4230.278	3455.163	1346.94	3118.285	3003.14	2242.265	5735	4295
263	142.14	1108	3794.72	3381.408	4114.134	1875.805	2619.112	3410.545	2652.637	3376	4100
264	142.14	1082	3359.81	3502.515	4007.815	2903.57	3077.744	3205.629	3140.9	3262	3847
265	142.14	1165	2937.64	3554.557	3432.668	2660.972	2361.537	3151.716	2356.644	3109	3545
266	142.14	1066	2406.74	2511.903	2065.982	1990.264	3032.902	1766.309	2298.018	2249	2040
267	142.14	92	3761.15	2516.014	3327.738	3095.813	2745.252	3429.82	2363.478	3296	2177
268	142.14	2	1199.14	1880.313	909.4376	1113.915	805.9886	1143.378	1014.972	945.9	1966
269	142.9	69	2177	1335.654	1023.229	1226.428	741.729	1196.296	1449.633	1244	1360
270	143	1181	1319.3	1422.044	960.0429	1169.543	1421.966	1324.271	922.7667	1332	1370
271	143	1078	2325.86	2458.468	2563.05	2038.422	2326.56	2126.707	2201.182	1663	2584
272	143	1109	6819.78	2798.419	2952.309	2244.365	2158.152	2222.163	2314.268	3648	2980
273	143	1005	1461.78	1518.369	902.8846	1541.199	1137.61	1194.075	1196.707	1400	1495
274	143	975	1595.36	1448.008	1720.419	1039.275	694.4358	617.4575	1287.018	1540	1686
275	143	947	1270.97	1120.025	976.1518	2203.012	1796.842	815.6299	572.0145	1332	1395
276	143	1125	2267.41	2499.817	2221.463	1608.388	2581.093	2174.075	1900.67	2278	2691
277	143.02	915	539.235	1605.403	322.7918	255.3221	1408.651	1066.97	1200.743	1314	1501
278	143.05	830	832.298	625.3457	924.903	780.8724	805.3514	703.9586	587.4738	788.3	812.3
279	143.05	868	1058.27	675.8619	773.8924	774.3033	625.8501	745.8682	748.125	891.4	853.6
280	143.05	780	696.315	600.6113	712.5596	604.3718	648.1928	661.1529	258.1997	804.7	868.5
281	143.08	59	2301.35	1319.497	4800.77	2150.271	752.6716	1544.217	1562.915	1353	1477
282	143.12	142	1743.1	1813.614	1649.108	1410.894	1505.775	1232.817	3225.76	1141	1374
283	143.15	2	410.264	401.7323	337.194	471.0499	385.4119	5271.872	292.9702	4693	542.7
284	143.15	111	4904.44	9919.421	11944.95	1351.98	15987.38	6637.96	2028.07	6509	5809
285	143.15	1190	3265.48	3768.016	2430.563	2920.739	0	3090	2053.783	3036	4156
286	143.15	44	6107.88	2521.396	6322.058	4237.524	5146.924	5497.724	6313.5	5544	5871
287	143.15	1164	3938.42	3427.34	4065.384	1653.228	2011.392	3549.912	2466.188	4021	489
288	143.15	1110	5909.82	5615.98	17234.91	3515.494	5252.48	5169.839	4808.397	10862	6312
289	143.15	1079	4926.31	5043.233	5241.732	3975.128	4790.257	4471.647	4127.488	2618	5123
290	144	996	383.513	1564.29	1454.668	1379.052	1274.668	1313.747	1265.835	1643	1735
291	144	944	1351.06	895.2436	1274.14	894.9	816.5464	731.6122	902.4591	1177	1360
292	144	966	2718.7	2305.723	2678.354	1992.665	2096.448	1948.41	1962.033	2326	2690
293	144	915	1525.07	4283.426	1759.995	1713.733	1657.819	1238.76	1706.427	1677	1954
294	144	1078	2951.71	3128.936	3176.33	2794.568	2954.362	2897.183	3386.21	7367	3493
295	144	2	296.248	398.05	324.8972	316.7155	348.9432	359.9117	281.0769	131.5	359.4
296	144.06	86	2441	7909.876	2555.999	5865.287	13382.02	4230.493	7590.378	4494	3723
297	144.1	61	3339.17	5967.982	2104.135	1998.857	5623.834	3074.167	1568.292	4397	3931
298	144.98	1046	1678.61	2259.772	1430.352	1743.465	1434.625	2126.382	1530.672	1530	3620
299	144.98	51	1773.04	2209.788	2016.27	2624.898	877.7311	1932.83	1756.075	1781	1767
300	144.98	21	1595.55	1936.878	1576.625	1551.111	1995.2	1822.77	1070.776	1598	2368
301	144.98	108	1897.06	1802.249	1756.211	1158.241	1739.431	2171.091	2443.539	2023	1447
302	145.02	911	904.262	747.2547	581.5656	648.2207	513.8653	587.466	644.451	1073	673.2
303	145.02	890	503.745	551.6533	691.9557	655.4795	807.4423	775.4108	669.3988	1859	819.1
304	145.02	1110	1693.43	1842.299	1699.511	867.2365	1578.414	2280.96	1773.63	2578	2687
305	145.02	1077	3023.79	1819.08	1385.418	2637.634	1397.195	3050.093	3084.085	2892	2215
306	145.02	948	840.577	1224.612	1462.55	1138.065	1055.912	840.0798	1094.888	705.7	1457
307	145.02	995	1765.33	169.05	642.7025	1308.82	1223.289	1062.389	1161.667	5704	1344
308	145.02	965	1361	5750.758	1291.376	1809.908	1736.798	1672.518	1677.284	2003	2337
309	145.17	957	541.36	539.0585	436.84	406.1423	462.8684	486.2196	376.257	479.9	597.3
310	146.12	63	9260.13	5225.579	9305.521	3975.429	4741.837	5983.772	7297.675	10562	7138
311	146.16	345	1970.26	1452.656	1880.952	1802.854	1807.619	1380.651	1334.115	2427	1811
312	146.16	1119	6955.3	7190.229	6696.908	4031.306	6613.189	5057.333	4826.858	6881	5812
313	147.06	66	637.14	3497.26	3736.526	4070.458	3019.238	4453.136	855.0525	2207	4124
314	147.08	152	2061.62	1208.232	1235.168	5415.113	6097.825	6534.193	6482.246	7225	7507
315	148	64	7061.75	5075.844	8425.234	3489.601	2180.317	5607.227	4925.804	7485	1486
316	148	78	6289.88	9044.447	6200.725	6319.466	3615.715	8895.179	6407.749	6328	6891
317	148.06	286	32757.5	27841.88	36311.35	8123.168	14469.39	9796.448	24729.83	23823	21247
318	148.93	63	3018.65	2117.153	1724.075	905.6686	1807.539	1949.816	1686.012	2011	1985
319	149.02	106	197944	166218	332993.7	112479.8	255457.8	185360.8	158237.3	2E+05	2E+05
320	149.06	254	5022.57	2313.998	5403.124	2673.293	2611.55	2840.653	2743.887	6421	2741
321	150.03	106	19072	15814.46	32486.3	11537.82	28880.56	19366.32	16995.76	22551	23296
322	150.03	1072	4287.35	6845.884	4580.396	5795.416	7986.23	6692.464	5369.605	4930	5327
323	150.11	66	3560.58	2199.608	1571.664	1390.191	1837.302	3971.589	3021.903	10946	2486

324	150.13	121	1956.24	3032.847	4745.758	4161.378	4176.301	4368.044	3948.375	3378	2256
325	150.98	63	2194.06	698.9033	2561.114	3689.312	732.9664	777.267	1181.64	1238	1139
326	151.03	107	1791.59	5177.53	4074.614	1200.78	3628.373	2470.108	3733.626	2779	1684
327	151.03	827	1333.45	1510.32	1571.929	1100.826	1206.563	1158.93	1105.063	1247	1434
328	151.03	804	1190.9	1262.473	537.0145	1229.363	1173.929	1330.927	1161.211	1371	1416
329	151.03	877	1226.31	1101.289	1301.36	1150.553	3215.432	1062.176	1529.444	1288	1149
330	151.06	2	0	849.1519	0	702.9971	0	984.0877	1562.942	2245	333.2
331	151.09	471	3091.03	2822.018	6089.716	4761.652	630.1494	3799.069	3947.608	4714	4303
332	152.03	111	1178.97	412.1333	641.28	743.4	369.5844	1082.34	684.6629	906.7	1079
333	152.06	70	1218.92	3899.061	2981.602	1537.696	896.6636	819.704	1470.735	1167	1799
334	152.95	106	2198.82	4240.283	1041.032	4035.012	2067.993	4766.412	2318.626	3041	3468
335	152.95	1166	1722.88	1188.652	1613.127	1027.347	692.469	1845.521	1359.843	1877	1288
336	152.95	51	2150.38	950.7962	1269.945	1490.122	1793.053	1462.587	1405.111	1464	1199
337	152.95	1078	2767.7	2508.126	3140.669	2390.324	2792.196	2601.454	2816.917	2381	3120
338	152.95	1142	1417.89	1551.228	2091.547	1252.323	2194.89	1518.933	2200.807	1718	1135
339	152.95	1104	3324.23	1984.018	3545.192	2051.315	3142.596	3246.265	3005.775	6343	3884
340	152.95	205	1491.73	1000.648	1215.066	1055.661	1651.81	1338.355	1035.075	1008	1041
341	152.95	1120	3164.06	3300.065	3222.398	2455.792	4416.571	3202.914	3031.58	3452	2131
342	153.06	90	2711.58	6260.615	3329.419	6494.712	1125.493	6494.488	4540.471	3330	3781
343	153.09	119	1506.36	895.0575	1652.571	1825.254	1986.702	1117.8	1811.73	1308	2751
344	154.06	69	8546.58	4413.141	1434.998	1378.811	3296.774	4662.681	6277.7	5918	4427
345	154.06	98	23316	11873.22	21669.08	2553.144	5946.547	12950.42	3067.504	10332	9560
346	154.88	69	1418.3	585.57	1057.076	565.5435	690.831	1076.042	869.868	877.3	722.6
347	155.05	61	1743.58	1977.873	1469.476	1129.04	1291.045	844.6409	1559.258	1127	1513
348	155.05	895	1536.17	1321.335	1557.842	1440.257	1528.841	799.9475	1451.607	1444	1346
349	155.05	856	679.879	655.0306	532.245	572.0626	470.6509	408.7828	551.3867	585.1	703.4
350	155.05	913	769.438	748.9733	853.6891	956.2556	894.6891	1213.738	936.4011	648.7	834.1
351	155.15	67	1382.65	1710.965	1461.923	3641.989	2600.581	1077.373	1163.178	2270	2091
352	156.04	62	28062.7	13230.17	17139.74	4463.39	2166.416	5442.651	7239.131	7846	11265
353	156.09	470	1315.8	952.2968	1325.507	2586.69	1710.104	1230.34	1673.16	2044	896.8
354	156.15	95	3502.56	2739.947	1023.12	2907.622	1220.223	2301.446	5317.371	869.3	2246
355	156.15	1074	3324.38	1752.412	2569.097	4884.019	2286.287	6828.622	1838.25	1925	9942
356	157.02	54	1653.9	1213.445	995.8909	1126.533	1405.964	1329.675	1596.836	1019	1170
357	157.02	27	522.241	1167.676	547.6982	893.6098	982.219	675.3831	447.3576	477.6	982.2
358	157.02	1073	2533.75	2382.779	2170.193	2260.913	2277.72	2539.192	2528.25	2765	2948
359	157.02	1111	2404.81	2383.92	3047.405	1802.365	2898.277	2502.953	2156.467	4913	2787
360	157.02	959	4617.93	2669.957	3716.855	2314.233	3932.78	1614.971	3339.047	2865	6008
361	157.02	926	2164.19	2062.057	1424.955	2200.005	1976.476	1420.903	3468.3	2133	2301
362	157.08	91	3528.23	2649.988	4432.908	2876.809	1829.1	3201.534	4183.242	1693	3949
363	157.1	62	1842.12	981.3857	3082.14	2741.405	2216.161	1982.23	2402.015	2790	879.1
364	158.01	982	4399.21	2297.629	1953.553	2308.767	2027.152	2286.49	2338.334	2428	3597
365	158.01	1014	2744.48	5064.052	4260.596	240.1	2397.854	1237.2	942.1714	2692	12584
366	158.01	916	1167.25	1428.698	1142.943	994.734	1125.465	1033.032	770.4788	771.7	1194
367	158.01	950	2390.68	8176.849	2521.426	1105.313	1649.615	1221.462	1574.117	1580	2293
368	158.04	62	1580.38	1847.417	1371.375	615.66	237.4654	614.5313	642.872	723.4	910.4
369	158.15	60	3646.86	3667.85	3713.45	5264.186	4224.094	3658.78	4276.012	4597	4091
370	158.15	81	2834.44	6272.566	3747.943	3999.1	4632.206	4419.13	4306.982	5236	4670
371	158.15	467	1285.94	1229.635	2471.262	1276.542	1731.117	1335.233	1529.262	1103	780.1
372	158.16	136	1907.86	2872.501	3749.431	3217.312	2297.796	2474.377	927.275	3180	2629
373	158.16	1075	2981.26	5625.1	2206.397	3811.07	3078.906	4313.616	1554.498	1995	6443
374	158.96	84	158048	214842.5	192825.7	106537	187849.6	123098.3	162198.7	2E+05	2E+05
375	158.96	60	30481.3	20944.78	15093.37	12638.94	6432.136	17227.29	18255.88	18891	24580
376	159	965	3095.38	1251.914	1927.255	2183.649	7166.394	3070.46	3172.699	4493	4750
377	159	1008	1812.54	1895.842	2512.801	2984.936	1623.229	3165.981	3291.819	3512	8036
378	159	948	2537.89	3334.811	1619.68	1930.98	1841.506	487.2348	1965.877	3145	3336
379	159	925	1796.95	1055.529	1925.669	981.2818	1630.474	999.662	613.5882	991.7	983
380	159	987	2600.01	1907.876	2207.126	2194.729	3038.613	1790.55	927.6314	3845	2535
381	159.06	64	3268.25	2937.686	4843.025	1036.433	1760.328	1386.992	1433.695	1921	2138
382	159.07	94	10237.6	7396.2	5349.4	1547.181	462.8622	655.2748	2569.38	3424	3170
383	159.07	143	2730.01	1373.851	1951.85	1048.904	935.2024	886.1358	1742.206	1816	1958
384	159.97	85	5484.18	1678.451	2570.569	4327.553	8197.49	5184.052	4354.56	3269	4792
385	160.13	62	3184.32	1352.404	2625.367	1915.134	1976.499	1144.712	944.7886	1460	1762
386	160.18	2	624.456	1006.221	282.0966	410.684	312.6407	1191.996	195.9787	358.1	1451
387	160.18	1148	225.486	1149.737	495.52	642.1556	823.8959	198.7624	205.8667	288.1	572.7
388	160.18	1076	1113.62	1778.511	1709.25	1266.485	1719.297	1304.803	1203.033	1308	1617

389	160.18	971	890.239	412.5916	364.1062	310.8713	331.9527	362.4882	298.158	635.8	656.9
390	160.18	44	1053.27	980.4414	1073.702	305.3672	965.5176	685.1964	854.5241	940.9	329.9
391	160.18	1109	2346.87	1972.06	3456.05	1395.274	2708.45	1712.945	1667.045	3345	1895
392	160.18	1125	1586.01	2912.902	1597.197	1399.096	2567.737	1531.175	1316.773	1867	6845
393	160.18	944	1394.85	745.637	722.8653	398.4353	447.5468	534.8994	500.8978	612.9	597.9
394	161.09	467	2879.99	2571.123	3761.536	3411.596	3250.927	3001.528	3208.38	3430	3151
395	162.05	80	1104.64	2225.819	3505.197	1028.815	900.4091	1525.019	864.8982	987.6	3361
396	162.11	69	53406.4	43479.65	60061.81	12880.16	26566.03	26499.27	28820.18	37008	42473
397	163.07	1067	3409.94	2553.976	2123.963	1768.636	2131.855	3276.477	2165.277	2986	3975
398	163.07	75	1742.68	3173.218	2125.51	2731.299	2403.317	1863.875	2150.258	1292	1846
399	163.12	471	6330.48	5242.358	9167.335	5452.701	6461.67	5113.789	2837.57	7949	2506
400	164.03	91	24466	16046.08	24265.51	2056.774	8876.051	7781.337	10554.72	21011	13198
401	164.13	68	667.307	3213.336	1851.908	2039.242	1681.608	1408.543	1944.556	2158	1376
402	165.05	903	1194.29	2051.842	739.68	1170.527	1292.499	2771.144	1100.742	1092	1305
403	165.05	927	173.575	416.6827	817.08	589.2069	606.06	554.8832	560.2133	581.3	588.9
404	165.07	66	10355.4	2883.648	1337.464	1830.871	18712.02	2004.111	4276.062	6376	6894
405	165.11	119	855.872	891.5675	939.393	611.8272	741.312	1749.469	845.4071	1403	151.3
406	165.11	1076	1998.61	1542.113	5339.938	2060.431	2107.278	2038.23	2168.155	2023	1986
407	166.08	62	12191.6	9001.804	9332.178	4186.08	8166.241	7090.344	7179.118	8325	6374
408	166.1	1	408.834	348.4737	439.9868	313.2859	290.6838	366.9333	398.2735	340.3	452.6
409	166.1	1054	3017.56	2907.629	2035.255	2447.791	2850.351	3085.311	3086.202	2928	3407
410	166.96	970	1422.84	1019.717	1936.56	438.2262	1493.095	1553.63	1578.066	929.3	2982
411	166.96	994	137.855	646.4346	662.205	430.5311	545.4336	539.6625	527.9209	708.7	861.1
412	166.96	940	785.598	4435.242	1191.993	410.0688	419.8241	1226.651	1269.079	583.7	2382
413	167.01	1079	1883.74	1731.494	2372.231	1547.566	2142.848	1956.701	1879.837	1887	2440
414	167.01	1119	3995.05	2052.077	2760.911	1372.163	3197.992	1608.873	2269.098	2517	2139
415	167.01	2	380.041	571.8308	362.8269	669.305	318.3869	679.3205	296.7404	279.6	816.7
416	167.03	103	1306.62	1831.477	1115.372	1614.644	947.274	2526.536	1302.812	1140	1062
417	167.09	61	1748.66	2116.155	1939.949	634.9	2494.194	2519.304	2493.338	2498	2607
418	167.15	92	25765.1	19601.19	31650.01	13958.43	21750.39	22058.86	23406.33	19723	19534
419	167.15	1043	25599	29910.7	24759.04	18146.91	20606.99	28373.67	21012.42	22115	34818
420	167.15	110	28718.9	31986.65	35097.47	20309.39	32154.65	80281.53	21421.98	26177	27340
421	167.15	1109	40133.2	37994.09	40612.84	26747.68	32157.19	30512.76	15393.59	63267	44120
422	167.15	1075	25093	34518.51	47007.17	26827.37	31217.48	19979.56	22830.62	22944	44537
423	167.15	200	25410.9	25201.36	25623.56	23065.67	22335.31	23263.78	20384.83	26024	25707
424	167.15	217	26685.6	16726.23	27174.62	21246.21	17235.36	19834.12	14292.37	21279	21408
425	167.15	1186	18547.1	24477.56	11941.46	16366.3	15242.74	17263.2	11112.79	16181	24576
426	167.15	1165	21171.6	29082.25	21083.96	14134.04	13088.03	20270.41	14125.39	23348	22239
427	167.15	1123	47984.4	43957.26	51745.81	15076.1	38342.55	23660.89	34473.65	45804	53218
428	167.15	167	38205.6	32783.7	25047.2	25552.5	35637.1	33449.08	27061.6	37778	36916
429	168.06	66	2121.73	1189.709	2126.758	1267.906	1059.281	968.219	1533.268	1303	1649
430	168.15	118	5443.11	5923.294	4385.568	3285.152	6775.206	5111.174	4360.696	4423	4858
431	168.16	98	4773.37	2927.714	3485.736	2241.311	3459.848	4558.772	3978.452	5821	2891
432	168.16	1113	3708.25	5682.3	4096.262	2756.948	3070.744	2961.906	2770.714	3373	4479
433	168.16	1075	3061.39	4359.42	3126.533	3349.713	3589.401	3277.111	2923.875	2685	5771
434	168.16	40	3568.92	4350.75	3686.008	3052.245	3697.518	3243.378	2550.742	2674	4147
435	169.08	110	30198.6	655.2	18269.12	1518.064	4880.363	3570.46	5553.491	3952	15559
436	169.1	65	1413.36	7505.416	1258.091	1791.708	12008.38	795.1486	1123.679	1353	2214
437	169.17	1075	8374.95	15630.03	18384.47	10794.3	14736.38	12973.95	6698.287	7862	24147
438	169.98	78	24208	34806.92	22048.48	23829.97	16929.53	14575.7	23366.51	23902	25815
439	169.98	64	27703.9	15138.1	31598.29	9763.038	23200.58	20798.64	18328.29	28314	16385
440	170.03	108	2878.86	2581.895	4230.961	1601.107	2410.298	1901.88	1327.023	2113	2457
441	170.03	64	5911.46	4629.269	31333.46	15397.9	18393.7	559.436	18695.86	22169	19142
442	170.03	125	0	712.81	139.7293	304.4615	870.7856	105.07	0	0	610.8
443	170.07	849	747.244	536.13	557.7223	693.1727	736.8741	863.8207	515.6114	1345	770.5
444	170.12	1041	5508.16	4312.744	5298.947	5002.829	5185.217	1186.492	5880.488	4648	5200
445	170.12	588	543.664	443.0481	448.7927	514.4085	485.022	477.2018	232.4692	490.1	195.3
446	170.12	468	3022.19	2847.946	3732.754	3830.761	3859.611	3622.292	1372.18	1289	4088
447	170.13	116	2647.12	2303.926	1835.364	1281.084	1631.85	5347.278	1525.526	1948	2845
448	170.13	354	1383.71	1573.314	1428.045	1437.883	1594.033	1549.28	1384.344	880.2	1437
449	170.13	282	845.555	2277.48	622.017	652.9248	808.1241	957.996	889.014	1048	326.7
450	170.13	1191	1526.27	1711.522	1320.701	1369.111	887.995	1454.64	1138.733	1656	1577
451	170.13	1171	1284.12	1022.986	1227.003	1700.755	915.936	675.8589	1236.667	1548	2128
452	170.15	252	4856.31	4113.526	4745.713	4671.24	4803.36	4320.461	4472.936	4895	4408
453	170.15	229	1245.9	2718.637	1720.565	2254.987	3245.956	2710.322	2623.131	3035	3094

454	170.15	20	1407.92	2304.873	2221.032	2072.896	1296.114	2550.794	1934.138	2218	2235
455	170.15	4	919.224	913.5566	1002.989	331.356	862.0776	1395.29	977.6548	971.6	1155
456	170.17	1077	1920.1	2547.818	2273.205	1466.793	1665.494	1500.313	1288.641	784.4	1615
457	170.17	166	1861.35	1245.584	1736.842	1150.918	1150.904	1723.278	1255.078	1643	1714
458	170.17	55	1140.54	1058.9	1411.201	484.2067	1185.206	772.2141	911.8097	1099	585.4
459	170.17	1115	2217.15	2117.198	2567.313	1062.429	1752.774	4061.027	1530.243	3750	2442
460	171.03	992	2538.67	1469.213	1427.854	1172.55	1566.326	2052.043	2124.015	2775	882.5
461	171.03	903	1153.38	1203.192	983.3118	1181.898	984.5995	1001.847	1065.804	987.5	1247
462	171.03	1014	4878.48	2675.505	7070.407	5116.158	620.675	4052.875	4236.435	2185	2393
463	171.03	944	1317.73	6263.33	2031.667	672.175	1653.066	1420.351	700.0567	6257	1474
464	171.03	969	2652.95	1536.259	1494.294	1514.354	4419.878	1520.293	1372.002	2239	2158
465	171.1	118	12327.8	5336.425	3808.044	2523.344	5327.189	6401.124	4623.075	5930	6671
466	171.1	1064	5610.91	2871.333	1830.135	1913.687	1976.244	1412.225	2815.2	1042	2769
467	171.13	467	2607.74	2132.737	5518.9	666.9143	2819.934	2525.924	3121.417	3305	2047
468	171.13	140	1302.84	1274.62	1547.52	1348.587	953.304	907.378	1060.884	1211	1261
469	171.15	71	1973.72	1910.311	2001.453	2315.462	2587.987	2929.153	2761.168	2704	2250
470	171.15	1075	2055.75	2425.101	889.02	2077.373	1966.134	2135.88	2031.65	1972	2732
471	172.07	470	1216.25	1188.161	2123.32	2269.74	1779.473	1506.049	1728.198	1950	3055
472	172.07	1	469.739	336.644	579.616	366.5205	386.6685	461.4072	485.73	505	307
473	172.07	1050	2880.54	2842.639	2819.67	2395.934	2963.502	2727.993	7061.978	2858	3169
474	172.07	142	2176.05	873.5638	3282.668	2743.358	2195.712	2057.46	2761.647	2623	2587
475	172.08	100	1649.09	2514.072	793.36	1792.46	1508.51	1232	1491.246	2039	2285
476	172.09	64	1585	5976.397	2437.292	1422.236	2491.073	2507.176	2532.449	3586	3561
477	172.98	62	3499.65	2821.902	2128.965	2377.641	1013.769	2150.024	2166.054	1812	3063
478	172.98	84	16501.4	12263.65	17418.48	7954.693	18530.77	11272.22	12206.61	9636	18115
479	173.08	89	6786.8	7791.001	2822.367	7500.381	7617.102	5153.976	20861.18	3986	8575
480	173.08	1073	2692.16	2224.256	2763.074	2939.644	2934.375	2959.06	3033.69	2329	10012
481	173.13	63	6718.21	6911.415	1637.286	880.8447	4325.067	5595.931	1213.814	1872	8297
482	173.13	209	2400.55	1632.142	2463.986	2515.638	2291.235	1916.825	1361.66	1814	2220
483	173.13	195	1644.87	1802.125	2669.471	3300	1558.662	1502.868	1303.619	1366	1689
484	174.01	66	1966.65	2226.014	636.8971	950.3731	1058.821	1561.036	934.0763	1059	1387
485	174.15	69	2979.6	1178.589	1326.553	1775.8	3530.39	1327.203	1895.84	2098	1278
486	174.94	82	22700.5	22459.07	27643.47	10051.25	15968.73	18251.2	14579.09	15787	21193
487	175.03	968	1277.62	432.8862	271.5555	395.3971	394.6519	387.2136	342.1669	402.7	423.6
488	175.03	64	1644.21	890.188	892.413	903.8835	793.8418	958.3518	1074.689	784.1	844.6
489	176.04	85	13503.3	4165.25	22045.59	2815.404	2222.171	5040.633	6610.867	3646	9768
490	176.06	62	9706.28	2855.938	23841.91	732.998	6737.704	2134.025	2338.606	11533	3413
491	176.1	167	3306.53	1208.268	3677.778	865.6632	1165.033	1036.851	1578.385	2158	1824
492	176.14	60	17463.7	15978.68	23157.09	4689.465	6667.669	6362.917	12339.62	10275	12743
493	176.93	83	2250.31	1532.78	2825.331	1279.967	1281.611	1447.607	1771.953	2014	2254
494	177.05	111	7638.69	5284.308	9295.574	3117.855	2591.277	2246.876	1292.293	3296	4125
495	177.14	61	1775.59	3336.547	2236.86	2054.36	1513.263	1700.846	2591.304	2516	2612
496	179.03	250	298.813	612.5697	491.515	831.894	829.8356	556.1309	489.0606	638.2	647.4
497	179.03	13	1556.86	352.5522	1713.679	606.4155	1399.045	2239.893	603.2048	1340	1404
498	179.03	1114	1564.23	1547.186	1897.106	565.4306	685.8519	1215.467	880.1673	1610	1548
499	179.03	316	657.787	1004.537	1332.863	638.4462	1119.177	755.4806	1398.728	1285	1292
500	179.03	161	897.057	1185.007	855.0306	909.14	858.9861	1099.482	932.1085	1573	1020
501	179.03	42	1008.87	122.0508	2240.698	274.6155	1454.572	624.1449	1467.69	1985	302.9
502	179.03	222	656	2583.836	1150.742	961.0826	1004.703	887.1168	1118.278	1155	984.2
503	179.03	283	503.698	492.4387	826.0556	384.123	492.6264	657.5654	808.1732	1012	475.9
504	179.03	201	1303.98	1552.626	1179.335	783.3417	1291.387	1390.465	1995.928	2440	989.2
505	179.03	186	992.243	805.2592	672.2743	1036.065	1387.391	1173.958	985.7852	1677	1207
506	179.03	1085	800.119	679.4993	992.98	427.5531	780.7422	685.3114	562.9157	733.5	723.8
507	179.03	345	708.538	813.102	983.9195	1366.752	696.7743	916.8152	800.9722	1303	998.2
508	181.02	63	6725.49	828.172	643.005	2257.27	6459.626	882.8344	2312.8	3007	3179
509	181.03	954	900.654	2200.773	731.2717	738.6935	766.1735	632.3525	929.4297	1069	1026
510	181.03	970	1675.65	1725.53	501.16	1847.348	1841.803	1443.047	1399.63	1682	1378
511	181.03	1008	1032.53	2139.282	565.5	915.009	844.7428	817.0875	902.0976	959.2	754.6
512	181.03	985	823.775	351.7847	1229.362	742.7148	709.9601	608.7134	785.8572	841.8	966.7
513	181.03	1109	1074.79	1037.985	2164.025	704.0688	788.6982	869.924	663.0633	1068	834.4
514	181.03	13	889.606	305.9404	1007.788	242.3648	828.5373	1129.205	343.765	821.6	1354
515	181.06	102	1585.65	1720.811	1845.52	1758.75	1191.104	2015.982	1259.511	1150	1652
516	181.16	208	869.218	331.2	1336.747	769.6293	1322.654	500.0002	882.658	1013	543.3
517	182.06	816	329.738	332.9192	433.0793	314.6876	320.0093	511.04	302.9282	562.4	418.9
518	182.06	884	1884.28	1603.165	6244.354	1804.609	809.6001	1886.795	1873.428	2876	2239

519	182.07	852	545.998	493.2817	844.2065	517.1927	473.8847	527.3759	480.4614	996.7	825.8
520	182.08	69	4408.67	4434.987	3522.781	4490.72	2747.809	5555.737	1638.534	5176	4492
521	183.09	69	1070.67	1349.193	847.8611	2136.645	2480.037	1624.035	1257.013	1144	725.1
522	183.11	113	1023.61	2601.827	938.7467	1072.898	2528.232	2018.146	2349.618	2512	2187
523	183.11	1064	1962.53	1067.081	1918.454	1009.286	1511.631	2809.052	632.9945	1406	2339
524	184.07	255	20514.8	6074.949	19965.67	7947.027	9109.174	7694.67	13508.28	14089	8931
525	184.09	65	1568.25	1854.145	1603.184	814.8549	1496.354	1713.334	1726.464	2114	1502
526	185.11	73	4951.22	6421.959	3013.591	6403.919	1312.379	3620.931	5443.509	2032	4943
527	185.96	72	9412.37	3423.643	12759.59	6825.981	4363.077	7313.729	7218.549	6410	6815
528	186.01	987	664.697	849.7201	578.8216	600.2427	594.8263	503.815	600.4255	594.3	618.6
529	186.01	1008	626.536	474.89	447.0053	451.1382	543.4521	484.8907	530.2772	583.9	626.5
530	186.01	962	1182.22	577.7691	992.94	961.5857	971.618	845.7194	952.5749	638.8	5619
531	186.18	63	1855.4	1226.925	2307.435	2223.96	2542.284	3741.016	1700.798	2174	1097
532	186.22	470	1210.66	931.4576	1350.869	1839.323	1262.723	1321.778	1250.359	1664	1218
533	186.22	97	7174.13	6472.832	7201.084	5148.924	3557.453	10033.57	5818.796	7349	8939
534	187.09	64	2337.87	4203.47	2232.532	2033.862	1350.247	2322.951	2180.158	2806	2188
535	188.07	1	182.261	192.9223	532.8597	167.6135	254.7875	186.9054	276.7905	862.4	300.5
536	188.1	67	2274.16	1102.31	3592.576	2395.883	1961.346	1627.941	3090.485	2113	2272
537	188.17	345	6553.57	2247.48	4963.286	917.9453	629.0042	520.1967	1684.938	2687	2666
538	189.09	70	1437.95	1509.642	1123.433	1206.94	1111.944	1011.944	936.768	1195	720.8
539	189.11	118	9801.38	2790.545	629.256	3640.278	2684.94	3771.278	698.8314	4516	4349
540	190.91	80	1551.61	2306.652	3336.728	1794.564	256.592	934.308	1495.47	1741	2292
541	191.04	91	3533.2	1365.789	1048.79	3192.167	10624.28	13221.18	13131.88	10639	15441
542	191.04	125	1209.74	628.7478	369.702	2232.729	4853.16	5352.887	4669.968	3536	4549
543	191.14	196	1010.73	703.319	1597.988	2565.3	1354.658	807.0317	1197.375	1140	1345
544	192.02	295	1682.73	1393.767	3471.726	1134.231	922.355	1095.048	770.6308	1375	1505
545	192.02	261	8039.03	5024.888	5514.04	3259.567	8492.634	2959.929	3184.084	3687	13444
546	192.02	244	6749.12	2502.011	5246.271	2953.972	3272.686	2841.574	3831.786	4291	3626
547	192.02	220	3914.09	979.0457	2032.254	1634.544	1260.412	1884.773	1572.822	1921	1504
548	192.14	1044	77801.4	97453.44	48224.16	41048.82	72124	170188.4	69834.51	96120	80852
549	193.14	1045	13348.3	16717.67	12642.84	9655.5	11999.14	29337.58	11324.34	14286	13655
550	193.16	208	689.683	18.207	1154.253	251.615	1006.824	290.4	720.3385	385	46.54
551	194.11	87	2361.95	3026.176	2523.01	2286.25	1528.144	3657.891	3128.78	3148	2393
552	194.12	971	2455.02	1039.194	502.3732	495.3555	940.752	988.0348	859.3887	1016	1400
553	194.12	128	2696.37	2093.299	1282.61	2534.936	2292.896	2057.51	2612.088	2476	2956
554	194.12	931	800.899	617.7595	645.725	753.4429	619.2232	637.3551	639.5068	579.6	844.5
555	194.14	60	8601.23	8485.239	4068.19	3899.104	8002.876	2356.386	7805.192	7535	9389
556	195.08	113	1525.84	3873.261	3261.931	3653.314	4768.256	3213.557	4949.741	3826	3700
557	195.12	62	7938.52	6752.049	6591.738	299.367	9301.394	8331.282	8657.539	8962	1119
558	196.1	64	1578.47	1214.609	1142.369	2327.118	1821.396	1575.229	1841.323	1816	1456
559	196.86	70	41173.6	26842.05	26146.04	20516.24	18559.24	31748.49	28886.21	38083	31431
560	198.04	960	1488.49	754.9143	602.4851	727.6787	896.0845	828.4858	840.8066	1158	1173
561	198.04	990	848.09	971.208	688.7999	661.2863	677.2444	530.1429	658.5566	710.2	722.8
562	198.04	1003	187.198	386.88	500.1223	393.3455	385.9091	343.06	357.8073	508.9	482
563	198.05	142	903.905	1621.989	2642.597	1548.8	1307.033	1684.801	2375.285	1536	1893
564	198.08	221	5616.48	1455.679	1792.891	1200.229	1292.669	1175.823	1486.327	1922	2072
565	198.18	207	2428.89	608.8669	2870.625	1694.113	3039.326	912.5129	1683.5	2102	1063
566	198.86	70	40024.1	22430.94	26491.2	22755.99	17758.86	28926.06	29302.81	31605	32864
567	199.08	106	7811.68	597.872	5869.271	674.5115	710.64	469.4062	1451.47	1282	1919
568	199.09	70	1600.49	1155.669	1339.362	1488.073	1922.946	2199.682	1335.154	2337	1260
569	200.02	917	429.3	420.2332	416.4824	315.4764	411.3342	415.0588	369.642	388.8	604.8
570	200.02	1012	1430.96	2675.483	1403.482	1257.66	1698.703	1047.547	5748.614	1984	1398
571	200.02	993	2044.56	787.2475	388.3116	1016.643	1957.82	1638.047	1820.067	2231	2137
572	200.02	966	1616.06	1641.451	2065.837	1186.95	1706.156	1390.732	1260.063	2121	777.7
573	200.18	70	1788.45	1827.505	1200.589	1302.251	1073.859	1284.902	1171.84	1318	1139
574	200.2	99	1427.06	2208.091	1888.091	3102.032	1692.246	2697.231	2076.8	1699	3262
575	200.2	130	975.432	305.4333	1143.421	486.41	745.7914	733.7	698.1957	658.4	433.9
576	200.24	109	4227.23	1001.583	2492.646	1774.053	1660.05	4706.466	1350.916	3611	2326
577	200.86	71	12629.3	9738.384	7112.154	7450.597	6134.7	11890.39	11788.28	13469	13000
578	201.05	94	24483.8	4282.426	3641.012	8026.618	15538.96	4796.867	6814.176	4884	11102
579	201.11	64	2355.15	2547.036	1761.838	2234.51	1773.645	2253.798	2274.176	2274	2203
580	202.18	60	52043.2	33129.54	61634.31	66623.26	78003.95	89418.11	68064.82	61929	80357
581	202.21	74	7564.62	21009.34	1241.402	1753.02	20855.58	19163.78	1187.398	2232	1677
582	202.86	69	1915.87	559.4063	996.9867	584.64	962	876.075	717.9375	1142	1373
583	203.03	63	5576.08	1933.533	3014.031	1983.409	2974.727	657.3263	1818.598	2019	1681

584	203.1	99	1275.26	2539.017	2188.518	2668.28	1064.08	2619.42	2087.769	2751	2265
585	203.18	60	7018.75	4804.76	7904.933	7818.795	10311.09	10542.04	9429.759	8653	7976
586	204.12	64	23980.5	8838.245	25501.85	9257.14	16597.91	19158.81	15711.02	17473	13582
587	204.44	106	1671.88	1446.48	2217.193	1454.248	1515.759	1749.729	2224.602	2219	1330
588	205.09	1072	62553.6	68289.01	95457.86	96137.44	67503.18	90727.27	77504.78	70430	1E+05
589	205.09	106	305962	233444.6	541060.2	168564.2	379742.9	284552.1	244678.6	4E+05	3E+05
590	205.09	1048	43397.1	35491.96	41094.44	30227.72	43529.96	44892.63	48173.11	40153	45761
591	206.07	64	1453.28	1977.737	1841.651	2291.132	1043.166	2269.87	1975.289	1650	3131
592	206.09	1072	9367.95	9322.976	9439.224	10711.05	14727.56	13325.75	11106.91	10089	18287
593	206.09	1049	4108.54	5512.103	4990.429	4245.55	5675.854	6372.897	7101.162	6005	5700
594	206.09	106	43414.7	34792.98	73173.54	21552.46	66531.19	38457.77	25848.36	55863	40619
595	206.89	65	27915.6	10016.16	27318.85	10356.93	11696.36	23075.42	20335.43	20861	17626
596	207.09	106	5045.56	4724.44	9582.743	4378.5	7690.257	5061.889	4388.152	6128	3969
597	207.09	1071	1469.06	1717.806	1218.109	1200.789	1822.898	1942.932	2332.633	1757	2646
598	207.16	464	729.581	740.3396	988.5417	851.2013	1148.656	897.8436	957.4175	1044	766.1
599	207.16	126	1284.13	1422.145	2002.886	1446.451	1533.641	827.82	1122.24	1790	1379
600	208.17	197	1507.07	941.9312	2175.167	2991.28	1880.357	1738.874	822.87	1528	1971
601	208.89	65	17034.5	6131.313	19132.74	6619.746	6988.971	16207.46	11935.85	25007	11918
602	209.13	64	917.333	1268.349	1383.381	1873.628	1772.289	1432.414	1349.64	1554	1409
603	209.15	93	2975.4	3224.178	3273.459	1385.018	2455.154	4297.907	2860.258	2819	3740
604	209.15	196	1409.08	972.792	2245.166	908.1655	1863.098	1930.907	1593.53	4673	1848
605	209.15	209	1556.89	606.1176	3025.621	1632.89	1317.912	866.745	2073.841	2050	1150
606	209.19	1	63.8321	77	610.335	113.1767	156.6912	157.6368	303.4998	626.3	110.9
607	210.05	62	1481.12	1640.24	2305.827	1664.118	1418.769	1521.976	1654.868	2105	1286
608	210.09	1050	2490.6	2403.525	2852.929	2407.916	3341.423	3838.526	5766.469	2674	3110
609	210.89	65	4645.62	1296.171	2966.1	1292.929	1701.085	1929.25	2399.82	2090	2180
610	211.17	74	1142.57	954.408	1174.322	1245.019	1489.956	1378.467	1877.096	1467	1678
611	211.17	1044	2851.66	3225.899	1746.696	2265.207	2975.112	2514.49	2646.099	2990	3002
612	211.17	208	2067.36	740.2539	2728.96	1410.135	2895.942	1174.765	2038.018	2232	1362
613	212.04	717	1067.82	339.4437	431.5365	367.1554	315.3566	229.5506	140.895	505.7	165.8
614	212.16	66	1355.64	770.1185	1182.323	2186.739	3258.767	1073.3	1017.765	1466	1736
615	212.84	70	8270.47	2466.315	5255.923	3832.272	2689.848	5158.485	4611.743	4951	5286
616	213.15	70	2098.28	3280.47	712.58	1966.846	1840.529	2095.075	2231.082	1824	2051
617	214.01	230	1741.55	872.887	1340.443	2008.967	1273.607	996.82	947.1575	1421	1333
618	214.01	285	1762.86	1640.771	1822.81	1315.517	1095.733	1288.904	1349.386	1591	1780
619	214.01	246	9279.67	2211.402	1141.44	2323.737	2339.505	2280.179	2118.436	5873	2713
620	214.14	67	3568.24	1008.7	880.7682	2171.787	4284.633	1140.632	2254.945	2015	2980
621	214.18	1036	2381.16	2260.721	2378.969	2073.363	1119.638	2465.519	2351.287	2345	2548
622	214.23	122	5311.55	5766.125	3328.124	1296.42	3580.796	5753.274	2868.88	3585	1016
623	214.25	99	2095.59	1435.209	2030.233	2509.795	1484.008	2553.266	1479.175	2249	1524
624	214.83	70	7634.34	4236.933	5443.911	3191.358	2600.699	4490.341	5817.69	6048	4621
625	214.92	968	939.137	441.1	1909.663	712.056	725.0189	759.5625	777.4151	628	5179
626	214.92	997	664.19	500.7894	430.3853	511.0696	569.7413	531.3436	511.3231	607	926.2
627	214.92	1118	889.295	981.9833	1111.12	720.4127	944.384	759.2044	749.4982	844.3	1075
628	215.12	92	6978.65	7927.386	3070.491	7184.363	3366.246	8694.609	3544.523	4765	3001
629	215.12	472	1937.95	1747.032	2775.442	2773.247	2821.226	2353.71	2880.272	2764	2598
630	216.06	193	3130.28	3478.023	4788.128	889.1892	974.4643	831.2963	1379.203	1780	1644
631	216.06	140	3313.82	750.0867	4158.577	9650.853	899.16	937.876	1348.528	1665	1124
632	216.16	67	2523.48	2359.273	1370.591	3398.239	3144.678	1909.721	2243.139	2123	2642
633	216.16	1198	2639	3850.089	2957.9	0	0	0	0	2378	5043
634	216.16	1174	8784.72	7939.832	6828.594	6242.725	7172.319	7695.604	6324.884	9907	6537
635	216.16	1040	9910.69	10188.79	8906.205	8112.665	8982.268	10019.64	10541.96	10027	17598
636	216.16	1097	7148.35	6108.233	7344.914	6564.26	6562.763	7205.719	6716.236	6937	7737
637	216.83	71	2719.6	1266.923	2296.344	651.3455	913.458	2402.226	1012.154	2466	2395
638	216.92	62	74551.2	39056.66	37273.64	27204.74	17665.61	43478.63	43757.25	48702	53051
639	216.92	80	84471.9	67508.14	122977.2	37460.44	74120.51	43979.25	73123.8	85030	60712
640	217.1	65	19840.9	23007.14	14560.15	23739.68	15079.52	20299.6	16609.23	17359	19072
641	217.92	61	2271.86	1072.469	992.9143	870.9233	740.7501	1487.011	1231.366	1197	1283
642	217.93	78	2523.65	1804.079	1278.594	1031.155	2793.216	1284.224	2455.123	2916	1804
643	218.14	65	1438.97	2327.917	1503.648	2542.8	1938.318	2694.424	1805.748	2563	1585
644	218.21	69	75761.1	77359.15	30959.14	47466.55	56801.03	34649.59	45276.97	52222	58998
645	218.92	79	30374.2	20487.43	45450.32	14234.68	25426.67	16814.85	24213.57	49484	19607
646	218.92	62	26263.1	11878.05	13269.87	10332.33	5536.397	14862.59	13187.06	16380	19913
647	218.98	62	2424.71	213.76	273.8333	556.772	669.08	204.61	670.77	423.8	603.3
648	219.02	999	898.17	836.7618	789.1892	524.8907	702.1239	591.624	682.0181	736.9	1474

649	219.02	950	2145.71	991.1646	2820.596	435.591	926.9918	701.4684	973.1455	3889	486.8
650	219.02	1017	1558.24	1085.62	1525.022	542.874	1343.179	1187.196	1318.966	1399	4091
651	219.02	969	1168.58	1084.803	485.7255	1049.639	845.8229	1725.339	827.3538	1733	2626
652	219.14	67	1941.49	1364.322	895.0667	945.183	1714.304	2102.386	1593.55	1643	922
653	219.21	68	10049.9	10440.5	5798.548	8001.243	6863.257	5429.94	7159.706	8391	8498
654	220.12	61	3776.75	3499.476	4837.048	1264.3	2396.062	2420.863	3490.007	1983	2791
655	220.17	209	842.045	438.1385	1326.871	868.808	1269.746	622.0045	988.9038	1316	711.5
656	220.91	64	2367.53	916.7914	1307.025	1177.731	442.0378	1251.498	1279.399	1421	1676
657	220.93	1122	1743.55	1598.407	1996.734	1475.978	2284.498	1728.536	1740.317	1888	850.5
658	220.93	1108	1943.55	1773.371	4384.728	1591.358	2235.374	1806.252	1648.556	1593	1635
659	221.01	100	8119.04	3103.734	6681.656	86.64789	1412.835	2674.746	844.6175	2542	2293
660	221.04	63	1130.21	324.0557	505.7857	830.346	984.1541	429.1651	1487.676	1560	2260
661	221.11	99	4321.41	1560.266	3052.334	4504.195	5114.558	3376.578	3423.932	3977	5322
662	221.12	470	2117.62	1880.551	3167.058	1124.681	2689.357	2328.526	2815.015	2782	2675
663	221.17	207	3102.64	760.5495	4120.577	2478.246	4485.813	1377.054	2123.736	3568	1649
664	221.19	61	1825.33	955.4633	1763.325	865.4143	718.497	854.7733	646.8413	664.3	707.4
665	222.18	208	1048.49	354.6383	1457.664	785.5592	1294.465	595.4428	1234.404	1088	643.2
666	222.87	64	12670.7	925.9543	7949.656	4439.105	3541.2	4372.36	837.9077	1814	1110
667	223.09	105	5976.08	6522.865	10495.84	6900.941	6322.99	7494.651	5433.472	6562	5436
668	223.15	66	2038.99	1370.874	2093.28	2224.891	1734.637	1269.851	721.2488	1943	1007
669	223.15	2	171.511	97.02	424.581	96.8109	55.19455	108.9258	280.3111	458.3	119.8
670	224.13	106	1812.22	721.9784	1311.368	1130.22	2196.214	1896.484	1535.733	1381	1384
671	224.13	1009	2004.93	2169.718	10335.41	2192	1898.397	1623.36	2969.126	1944	2249
672	224.13	948	3732.59	1176.402	1238.291	1203.188	945.1543	1024.522	997.0139	1242	1327
673	224.13	139	2441.77	2141.839	2721.059	2480.792	2320.632	2071.999	1046.614	2816	1904
674	224.86	77	11990.3	3779.33	9145.996	3222.587	3298.115	2046.971	8470.958	6702	4880
675	224.86	63	7429.19	2381.156	5379.43	1869.178	3156.975	3054.93	3735.886	4463	3667
676	225.13	116	2844.09	1035.723	1313.348	2277.835	921.1716	1346.554	2790.538	910.2	3079
677	225.13	1067	1546.97	2059.23	1047.387	1465.27	1519.667	1276	1606.464	1550	1863
678	226.04	90	4250.34	2489.392	7080.031	2526.315	2376.147	3766.358	1549.922	2847	1851
679	226.08	61	1557.2	978.5098	873.68	927.2261	1983.907	669.8222	1145.564	835	1167
680	226.18	468	974.254	950.256	1130.808	1639.917	1405.745	1165.347	1339.718	1651	1518
681	226.18	209	6354.64	1874.195	10791.1	6502.93	10704.16	3552.714	7550.84	7900	4522
682	226.18	1075	1820.31	1759.658	2331.395	1668	1557.967	2063.102	1981.452	1667	1838
683	226.18	1179	4192.99	2867.192	2437.84	2107.446	4102.827	4960.167	1828.903	3995	2621
684	226.18	1050	4028	5691.167	3589	3144.309	3647.832	2572.328	3643.157	3741	5377
685	226.18	68	6875.63	5107.07	6350.372	19181.53	22872.93	9132.698	10553.56	10510	11733
686	226.18	195	6488.23	4814.88	9649.887	14284.8	9294.104	7790.915	6064.884	7213	13108
687	226.86	62	2735.47	825.5	1582.524	7710.839	2229.691	1073.718	681.2075	913.9	20104
688	226.95	84	383820	498007.4	474326.6	257351.8	480396.1	285166.6	338982.2	4E+05	4E+05
689	226.95	61	56789.6	38696.14	25524.15	25512.88	12008.07	31620.21	35125.35	33092	44835
690	227.18	206	2164.29	983.8007	3530.154	2144.969	3370.568	955.167	1235.592	2231	1061
691	227.18	68	1881.24	1522.56	1663.366	2535.697	3261.51	1751.668	2041.414	1591	1569
692	227.2	1052	1180.94	1926.775	1996.878	1749.863	1917.391	2009.848	1985.093	1933	1877
693	227.95	84	18025.3	24771.16	24047.23	12018.93	22696.07	15007.87	16705.94	20519	17004
694	228.19	74	14861.5	8349.333	12375.83	10726.74	14323.52	11938.88	456.576	10088	20935
695	228.19	47	5352.13	7357.256	5787.914	7647.172	5341.051	6575.12	6408.643	5737	7277
696	228.2	1180	12134.5	8134.873	5899.364	9829.708	12035.54	10551.31	8412.127	12235	8394
697	228.2	1041	30408.9	25208.87	17099.79	14394.38	36045.51	25591.02	28671.3	27412	27926
698	228.2	207	27665.1	7883.418	31129	18614.95	801.1233	16662.21	22196.55	25408	12409
699	228.2	1091	7071.05	6344.711	13902.07	8295.597	8207.106	7853.508	8486.314	7931	8556
700	228.23	95	4976.73	4967.741	1400.154	5153.835	5440.402	5324.378	2536.546	2506	4883
701	228.23	124	10160.7	9719.935	9505.634	3401.17	8147.936	12880.83	7777.643	6258	11125
702	228.23	149	7381.21	2836.286	7500.8	3405.505	8210.25	3943.517	6792.408	3092	3830
703	228.93	981	775.824	751.4518	699.7403	1125.382	586.5708	646.0275	779.3211	803.3	841.6
704	228.95	84	7698.91	10628.52	7996.056	4469.911	3937.042	3080.533	6287.773	5585	8358
705	229.14	75	1612.28	1179.264	2949.254	2264.112	2648.816	1599.383	2214.223	1758	3122
706	229.2	1190	1444.39	1399.631	1294.129	1205.338	1842.667	1745.449	1370.755	1457	1250
707	229.2	1042	4189.5	3974.17	2791.855	1571.839	4634.977	4108.335	4692.62	4023	6040
708	229.2	207	4164.92	1469.839	5668.089	3061.43	5256.139	2175.888	3705.846	3896	2239
709	229.23	124	4027.64	1839.2	3440.183	2106.227	2761.807	2192.999	1540.98	2539	1669
710	229.94	71	1332.5	672.0316	858.4229	965.9675	856.7	1203.869	1192.782	1858	1460
711	230.17	68	2423.89	1533.794	2488.002	1976.159	2708.821	2019.931	1929.466	1924	2105
712	230.25	90	80690.9	81789.58	61063.8	54569.64	65926.42	66570.57	65950.92	65252	82101
713	230.94	77	19143.9	6141.74	10833.65	4596.372	6092.936	3954.438	7835.352	12905	6776

714	230.94	62	7508.45	5714.038	3816.365	4641.391	1865.644	7075.368	4162.222	4919	7747
715	231.12	64	2926.35	3428.647	3002.125	2738.267	3271.404	2301.524	943.9554	3783	2694
716	231.25	90	13963.4	15910.61	12279.3	5981.859	13507.13	13169.13	13944.56	10598	13705
717	232.15	67	14692.4	13039.32	14836.88	7045.08	8515.643	10645.14	10223.54	9785	8136
718	232.17	130	822.433	323.4	826.1538	460.08	520.96	865.26	673.308	827.1	277
719	232.9	79	31879.6	16882.54	47064.27	13935.13	17659.99	13632.56	24118.7	30600	20199
720	232.93	62	2723.19	3296.267	12426.82	1418.308	3302.893	1309.91	10092.84	11240	13053
721	232.93	77	31749.8	16396.31	2570.583	13279.09	2131.64	15424.63	24596.11	33605	19914
722	233.08	63	2038.2	837.1256	2019.363	1487.617	1755.209	2280.674	1546.271	2049	1819
723	234.13	69	1040	2001.68	1153.259	1454.812	820.5084	1105.343	941.66	1071	1531
724	234.21	71	19870.4	18389.21	13039.53	17770.68	9407.526	7413.188	4533.963	5142	4719
725	234.89	78	22067.9	6852.186	22433.39	1090.805	6842.633	6368.052	13168.2	18154	5948
726	235.17	97	3445.91	1977.365	2744.776	3036.902	3895.178	5768.184	3426.898	3521	2973
727	235.21	70	3445.26	3026.34	1328.42	1116.124	1312.781	1541.735	1627.751	909.9	1540
728	236.16	207	1752.89	659.3288	1812.5	1754.992	2547.102	1351.239	1654.654	1929	1484
729	237.15	71	1543.55	1241.897	1024.884	636.022	1753.103	1768.258	1493.82	953.9	1060
730	237.23	149	1926.18	1606.4	1801.426	1862.496	1563.246	2163.696	1953.428	2204	1814
731	237.97	61	1880.25	1209.524	1229.288	958.9543	648.3258	915	804.9067	1022	1153
732	237.97	79	2214.86	4923.356	2165.711	2558.481	2813.773	3144.176	4550.244	3618	3798
733	238.14	75	2676.89	2841.719	2741.268	1919.613	3029.365	2840.425	2679.135	3980	2901
734	238.16	60	4488.01	2004.8	1738.491	1266.275	4884.464	1292.887	3461.464	2932	2801
735	238.17	139	3755.12	3975.064	5000.779	5388.143	4354.297	3687.853	3674.424	1413	4137
736	238.84	74	2002.45	1004.732	2215.485	780.3309	694.7385	544.0256	2112.138	2378	670.1
737	239.13	69	2251.64	8510.499	5586.429	5907.168	1356.313	5342.273	5622.553	1598	7032
738	240.09	65	974.252	1090.459	637.413	786.126	1635.153	894.7575	1480.453	1713	1566
739	240.18	468	1017.36	874.467	1793.685	2309.788	1304.385	973.3683	1215.331	1493	1413
740	240.23	129	4840.96	2848.207	4357.007	2871.352	5916.577	5840.885	4370.862	4443	5282
741	240.84	76	2657.97	718.1995	1834.982	671.7969	428.7158	1521.6	1346.153	1281	1081
742	240.97	85	45331.2	60716.89	55595.89	29072.69	52376.52	34473.63	40014.69	41891	45886
743	241.13	68	2119.98	14233.4	2327.012	2353.26	1282.278	1052.585	1723.075	1991	1190
744	241.23	131	804.978	2651.168	1649.833	2512.46	2504.54	1840.517	2523.925	2010	2298
745	241.97	86	2784.55	3563.375	2530.944	2552.29	3584.7	1679.683	2021.116	3505	3277
746	242.1	63	2034.96	1614.195	2206.108	1539.556	893.4033	963.9371	1251.663	1521	1551
747	242.25	163	6504.96	6822.66	6245.248	6996.445	8082.42	6662.816	4745.05	6927	7651
748	242.28	198	4595	2756.218	4106.596	4204.308	2858.299	2849.935	4050.989	4425	4338
749	242.92	82	73187.2	65374.88	120554.2	44712.68	70182.39	54077.24	64031.97	73040	62827
750	243.18	71	9848.87	1147.86	740.8233	951.6732	2903.304	1413.171	6320.102	5255	1434
751	243.24	142	8613.94	5986.923	8984.219	8166.812	8410.425	8097.45	5451.495	7640	7910
752	243.93	82	3397.89	3965.263	6748.728	4091.607	3872.175	4018.43	3282.675	4237	5270
753	244.09	63	908.919	926.2436	939.4085	999.4471	858.66	776.5867	989.6055	975.7	1161
754	244.19	1050	10600.2	1847.482	6327.598	6004.003	4810.537	4085.886	7204.722	9457	4958
755	244.19	1189	4656.74	3328.468	2195.199	2643.833	5663.138	3589.584	1950.624	6410	3334
756	244.19	1118	2177.7	1975.853	2044.973	1915.235	1926.844	2102.743	1782.308	1111	3385
757	244.19	207	19869.1	4246.848	761.2486	13525.53	23775.72	10546.13	16932.75	18601	9018
758	244.19	69	5796.2	4457.581	4426.741	17859.51	2202.2	7788.492	7663.755	8927	9770
759	244.23	92	3229.03	3744.328	4974.309	7072.823	5178.753	7830.589	6278.735	4631	4291
760	244.26	97	36228.6	23629.21	37448.51	74442.99	54056.69	52597.07	51674.97	44320	60845
761	244.92	82	7220.33	5731.519	11089.18	4959.612	6639.488	5746.179	4479.816	7537	6671
762	244.95	62	1524.01	817.5625	509.52	911.4617	316.3867	586.568	562.455	710.3	790.6
763	245.01	97	11083.4	654.3911	1238.911	2856.971	8453.765	0	4772.716	4744	5862
764	245.13	67	2796.14	1080.283	2440.319	3309.277	2545.956	1484.073	1998.763	2791	2040
765	245.17	475	2730.66	2359.068	4213.73	1904.317	1406.701	1278.263	1450.586	2435	2904
766	245.17	1060	4554.33	4176.731	3663.493	2129.75	2692.515	2784.828	2604.577	3018	4338
767	245.19	208	1580	1826.118	3870.825	3949.335	4206.726	1830.141	2492.203	3179	1337
768	245.27	96	6261.05	5491.966	8781.829	14279.23	9572.823	11830.45	8461.694	9248	12296
769	246.17	68	4967.3	5622.563	5148.708	40612.54	1622.929	1624.099	2932.16	2427	3147
770	246.18	140	1262.64	1730.767	847.6183	856.0289	1587.17	945.098	1641.54	1219	822.6
771	246.21	60	1270.08	2921.411	4045.4	2010.357	6983.159	4353.076	3324.687	3825	3181
772	246.24	74	93257.3	64094.51	43433.79	62459.44	46749.77	60532.04	65145.47	76595	75623
773	246.27	99	750.889	157.2222	827.4111	2269.322	1515.235	1113.226	1445.78	646.4	854
774	246.91	77	2241.04	2509.427	5236.678	1758.866	2290.383	2092.327	1911.197	1621	1945
775	246.91	62	1757.82	582.802	1105.247	736.4876	642.2823	1118.273	1401.225	1192	1717
776	247.06	125	5877.67	1034.828	4620.42	1267.216	1730.385	2152.085	1571.742	2426	4261
777	247.17	88	23982.5	6722.019	15635.47	10420.64	13625.28	12842.18	16968.05	11616	10225
778	247.17	298	6812.4	5827.565	8066.44	7451.53	6068.802	7156.841	7647.778	6691	7017

779	247.17	1183	8572.4	11033.8	5503.195	8815.625	7563.755	9728.914	6962.774	9063	8742
780	247.17	1114	7760.23	7632.19	8239.722	4670.832	7359.143	8944	8474.044	9191	6713
781	247.17	1064	8946.08	9488.917	8231.25	6489.544	12260.61	9783.745	10740.68	9139	9554
782	247.17	263	7013.36	5850.401	6414.536	7653.623	5382.46	6510.14	6416.768	6551	6780
783	247.17	139	11854.4	8038.553	11023.11	10508.77	10774.06	12148.27	17784.65	13601	9391
784	247.17	194	9079.19	7612.142	7721.817	8162.92	10355.3	8320.866	8674.014	9338	9533
785	247.17	4	5112.97	5450.266	4032.611	6057.706	3880.348	9999.056	4521.984	6560	9689
786	247.17	1085	8378.64	8917.333	9527.76	9654.916	8924.65	9364.312	9594.868	9247	36079
787	247.17	280	6074.15	6579.456	5386.82	5895.569	4297.834	4994.819	6215.017	5526	6166
788	247.17	239	8315.86	7007.266	8086.275	43617.01	9491.177	6713.675	7714.066	24154	15099
789	247.17	48	8071.19	6926.283	6837.512	8262.542	12766.63	8605.552	14461.43	7881	7202
790	247.17	157	9323.64	11273.62	7742.442	9872.874	11372.73	9569.661	9120.375	10102	9280
791	247.17	1154	6323.71	4906.371	6498.005	5558.431	5604.979	7277.121	5889.893	7771	6923
792	247.17	120	10835.4	9732.638	11361.1	11751.94	12195.82	12323.06	8491.833	10456	11000
793	247.17	214	9853.62	6779.196	8995.907	9391.523	9089.56	9678.869	7564.258	8419	7475
794	247.25	75	16233.6	11293.21	9015.058	12398.09	8123.672	12520	9817.936	11532	15673
795	248.17	2	965.071	942.9921	635.9364	1197.501	669.8103	1773.39	791.4062	1263	1820
796	248.17	1191	1432.37	1088.45	1197.362	888.5024	1102.423	1696.579	1128.414	1594	1220
797	248.17	26	1139.47	1904.177	1187.563	1277.21	1298.429	1543.576	981.76	1263	1412
798	248.17	1069	1670.92	1835.214	1481.296	4252.7	1609.997	1736.058	1580.67	1721	2199
799	248.17	211	2002.63	1201.047	1644.525	1127.203	1485.994	1339.658	1491.767	1593	1379
800	248.17	241	1464.26	1327.813	1615.842	1638.235	1579.225	1334.016	1327.97	1399	1396
801	248.17	146	2139.25	1962.891	1841.145	2711.988	1697.669	2641.424	979.5925	1600	2721
802	248.17	107	1968.55	2374.361	1642.446	1993.649	1879.273	2322.394	1421.723	1048	1499
803	248.17	1102	1357.22	923.82	1622.88	1445.217	1705.455	1570.293	1167.001	1392	1572
804	248.17	273	1817.37	2043.873	1044.817	2013.731	1722.209	2061.035	1751.549	1796	1968
805	248.17	41	1069.73	566.4813	1013.229	806.1072	1124.027	943.6161	1422.427	939.2	632.6
806	248.22	65	769.6	1480.593	1228.223	1851.125	2283.046	1430.396	2011.063	1941	1755
807	248.25	73	1656.91	1473.307	747.745	1266.322	1417.257	803.4883	1311.31	903.8	907.5
808	248.87	79	6577.84	2650.847	10341.38	3505.739	2159.105	3313.5	6015.903	5687	4476
809	248.91	71	688.94	1416.197	2456.202	500.7824	1923.939	1019.1	2405.746	1527	1335
810	249.15	69	1826.71	930.81	1754.378	1243.628	1285.152	988.3131	1326.964	1170	1305
811	249.18	98	1222.9	1663.011	968.5744	811.8938	1522.896	1522.315	822.3169	1177	885.8
812	250.18	76	8881.86	3571.633	9351.081	4913.279	10751.12	5299.422	5652.704	6630	4670
813	250.18	1178	3831.18	3029.038	3157.11	2789.242	2839.273	3476	2578.352	4430	3332
814	250.18	1097	5511.79	2357.923	2685.68	2822.048	2239.264	2888.076	2427.878	2550	2801
815	250.18	1037	4034.91	3414.419	4279.226	2824.802	3874.503	4116.436	4851.11	6351	5896
816	250.18	205	7490.9	3015.857	7582.943	5005.133	8138.966	5023.167	5776.799	6859	3707
817	250.18	1115	1762.87	1736.133	1684.046	1832.333	1769.762	1733.504	1487.46	1551	1998
818	250.18	467	651.292	631.1734	948.5714	1192.116	961.3058	1051.1	758.3969	1017	980
819	250.87	78	3365.53	1823.397	4493.816	1037.591	1215.872	2006.633	2983.611	2264	2128
820	250.92	63	1586.98	644.4229	1265.659	870.4286	486.2534	975.4486	931.7029	932.2	874.3
821	251.18	116	3903.42	3987.44	5170.597	2503.574	4455.149	2451.081	4226.756	2591	3767
822	251.18	1144	977.878	986.8385	2777.328	1032.948	1003.514	1051.637	811.2514	984.1	746.8
823	251.18	1171	3332.1	3248.917	3183.85	2821.508	3173.265	3552.239	2791.224	3426	3252
824	251.18	1193	2422.11	2324.564	2021.442	2025.387	0	0	0	2408	5514
825	251.18	1074	3312.32	2576.611	2978.953	2839.005	2924.423	3100.554	3437.613	3554	3246
826	251.18	1119	1781.33	1567.073	2039.407	1662.6	1784.16	1659.631	1624.431	1982	2035
827	251.18	1100	3090.6	2717.393	4038.757	3054.874	2903.122	3254.446	2941.579	3099	3734
828	252.16	65	851.021	1121.749	996.478	1932.109	2202.35	1282.056	1657.441	1172	1396
829	252.21	1108	1366.12	1146.678	1454.985	1366.985	2703.703	2043.647	2369.205	1665	1131
830	252.21	1074	1038.35	989.2647	1029.041	1512.208	1581.652	1415.082	1603.912	1029	1002
831	252.21	1125	475.956	550.036	428.5631	1382.88	2079.724	1147.022	1476.733	460.2	515.1
832	252.21	41	603.307	606.6456	606.9784	1292.868	1111.661	829.4679	1008.796	691.1	563.2
833	252.92	63	588.384	506.9967	773.9829	322.9167	323.7356	501.1667	641.0833	605.9	710.3
834	253.14	69	1551.01	1482.753	717.12	895.7689	927.5406	1142.044	843.8733	954	1001
835	253.18	1054	2494.98	1958.624	1843.789	1772.419	1689.443	2124.299	1788.968	1784	2310
836	254.25	106	7041.48	4510.224	8653.317	3257.1	7610.05	5546.301	4714.618	6277	5166
837	254.82	71	27558.6	19051.57	17130.07	16869.71	11709.77	21666.32	23043.5	27088	23255
838	254.98	84	2553	4407.813	3842.825	2547.505	2658.56	1948.35	2293.199	2979	3528
839	255.13	69	3124.96	3842.015	1175.244	2654.531	3842.755	2891.918	2396.52	1575	1033
840	255.21	68	1276.6	1694.014	971.6929	1770.742	1418.378	2075.602	956.8364	1527	1093
841	255.25	106	1486.24	1341.204	1087.305	1215.9	1531.008	1284.471	1679.93	1514	1341
842	255.98	90	14185.5	9483.606	8932.406	4782.995	3461.563	7788.631	6905.677	8502	10198
843	256.17	61	1236.48	1280.956	1551.734	1950.323	1520.393	1481.313	1805.938	1183	1564

844	256.26	128	27095.4	6359.374	20121.43	12172.19	1900.121	27360.7	12170.2	13996	16407
845	256.82	70	36484.9	22690.56	21824.99	16754.85	14757.6	26947.05	26189.73	33638	27235
846	256.94	81	10906.1	10033.64	13655.91	5627.034	7293.169	4943.267	7067.13	10232	7542
847	257.14	62	4869.57	3653.92	4505.387	1677.537	1106.208	1429.105	1595.571	1116	1491
848	257.27	179	2614.79	1081.791	1829.826	2003.014	1177.925	2307.03	1171.341	2257	2142
849	257.27	128	7586.83	1936.553	3854.153	3201.67	2699.844	698.36	2796.874	3589	2456
850	258.11	89	49284.9	15389.65	149291.7	10390	42127.95	23829.78	26922.24	31665	27151
851	258.24	111	34656.2	49703.66	47199.47	24117.84	57620.75	1030.135	36766.37	39117	41923
852	258.28	114	8129.68	11237.93	2620.845	5374.327	8077.52	3214.909	5599.157	10543	13494
853	258.82	70	17667.7	8651.181	11471.59	10057.34	7109.18	13782.67	12771.01	15913	14687
854	258.9	81	7480.12	7552.436	16996.82	4875.106	5614.743	6691.54	6344.575	7701	7875
855	259.11	98	6020.49	2895.286	3797.193	1267.138	2210.831	4179.007	4963.073	3744	2286
856	259.15	67	1819.76	1418.976	1214.335	808.158	2645.499	2063.463	2077.153	3421	2885
857	259.19	1070	1555.53	1625.912	1450.911	1458.114	1426.217	2782.597	1384.582	1343	1751
858	259.25	112	9493.41	2344.045	7504.164	5665.519	10610.11	7073.675	8988.646	1737	8837
859	260.15	64	826.892	1833	952.3864	1247.629	2174.547	1407.12	1669.363	2300	2063
860	260.24	242	1434.77	1258.799	1427.108	1120.701	1610.21	1007.16	1043.135	1231	1408
861	260.24	202	2504.43	2241.661	2440.778	2340.332	2202.775	2071.01	2128.02	2015	3604
862	260.25	110	981.41	1117.5	1138.86	1040.531	1333.275	1083.6	1387.444	865.1	917.3
863	260.26	79	3855.59	1531.116	2196.279	1645.855	4478.977	2524.467	2919.52	1269	3057
864	260.81	70	4987.3	1792.52	2496.498	2808.743	1466.4	3341.717	3142.385	3434	2869
865	260.95	63	3680.49	2102.488	2713.731	2408.907	1553.455	1952.308	1997.316	2337	2806
866	261.11	76	11323.2	3982.617	5703.163	2808.331	3012.632	5971.189	5583.76	7012	3223
867	261.13	62	10116.2	6924.926	8421.088	5707.941	9097.099	14228.95	8643.6	9795	12156
868	261.15	392	1615.46	1726.82	2074.378	1757.721	1684.526	2067.657	1897.51	2067	2229
869	261.15	99	5414.97	3463.703	4346.37	5179.802	4559.509	3243.638	4400.463	3734	4826
870	262.13	63	2086.3	1407.086	1759.25	1337.799	1318.176	1794.815	1714.702	1120	1585
871	262.24	68	49294.9	35875.37	682.8843	20618.13	27079.21	16598.84	21887.87	24621	29994
872	262.95	64	1564.79	621	1194.626	707.1429	388.7427	713.8686	774.5333	941.2	845.6
873	263.24	68	6869.62	7103.043	2451.324	2002.177	4127.5	2457.341	4022.421	4635	4066
874	264.23	1071	3284.32	2889.982	1753.014	2606.04	2844.946	2130.867	3462.251	2773	3613
875	264.85	64	7703.49	1613.826	4642.814	921.994	1776.037	2428.476	1892.533	6285	2697
876	265.11	85	7814.81	2304.076	7124.297	3022.422	2482.346	2661.778	7316.571	2652	4928
877	265.14	68	2422.5	1846.473	2060.73	877.2181	2263.047	1047.368	1811.803	1524	1601
878	266.17	68	4849.03	2780.949	3504.48	9328.152	11494	4593.497	6812.232	6872	8051
879	266.17	207	3446.86	993.4144	3426.159	2289.913	4091.435	3077.102	2891.693	3176	1500
880	266.85	64	5270.33	1284.84	3251.248	1249.903	1412.285	2031.46	2186.14	3215	4080
881	267.17	67	1155.68	702.2672	1015.387	1857.562	3045.503	1474.921	913.23	1452	1629
882	268.1	66	4754.21	2157.654	3100.948	1915.758	2120.213	1453.546	1993.962	2443	1696
883	268.84	65	1432.5	409.4609	1326.168	253.3405	558.57	563.0558	711.104	1358	992.4
884	269.06	138	1208.73	774.2308	1439.806	1338.325	1009.695	948.983	2075.226	978.8	930.1
885	269.09	60	7387.1	1538.901	7898.36	2670.365	2362.201	2131.1	3569.995	3113	2088
886	269.25	1073	2757.73	2305.367	2302.459	2193.956	2133.901	1880.198	1625.695	3585	1658
887	269.99	87	13182.4	10293.4	9872.866	6364.712	4575.711	5977.722	8061.021	9888	7757
888	270.28	238	2628.36	2365.105	3981.862	2748.859	3951.982	2320.832	2568.804	2811	2636
889	270.28	192	940.447	919.98	911.2963	1020.101	1052.725	809.3167	929.9832	1887	1112
890	270.31	313	2192.01	1049.817	792.4555	1552.715	1274.315	1153.989	760.9353	1383	1503
891	270.8	72	10050.4	6587.666	6055.572	3408.174	3178.343	6866.789	7628.349	7096	8050
892	270.98	62	7098.59	3297.64	3209.831	2465.984	1571.902	2643.483	2575.893	3140	4012
893	271.11	66	1179.02	1551.059	902.88	1465.061	1477.521	866.0308	1173.259	696.8	1647
894	271.27	98	997.506	234.2078	851.7891	757.7455	2863.484	3417.139	1382.842	1298	942.8
895	272.18	66	2438.3	3615.194	3770.242	3745.293	5017.785	6358.971	4376.264	4414	4395
896	272.26	79	7765.49	4328.466	4018.094	5248.909	3903.178	3358.454	4375.61	6008	7861
897	272.26	1050	1551.78	1721.353	3289.434	2761.092	2923.668	3664.546	3918.74	1641	3550
898	272.26	106	56584	52153.63	62454.35	16875.18	62549.53	39098.67	29600.45	54249	52252
899	272.29	131	13970.3	1222.615	11123.7	20065.43	24437.87	12789.92	10199.7	20603	13164
900	272.79	72	11611.8	7762.439	8143.552	5368.591	3881.8	8976.157	6177.429	10966	8691
901	273.06	61	1765.42	1235.568	760.3414	1141.771	1005.738	792.1129	825.3346	1045	974.5
902	273.17	79	10823.4	10164.79	8960.578	7711.3	11034.19	11372.92	1822.014	10713	8692
903	273.25	1041	5243.62	5149.652	4752.065	2417.82	3732.517	5793.09	5186.001	5445	6587
904	273.26	107	13071.3	10755.46	14895.23	1929.137	11519.23	8105.897	7478.284	10906	9723
905	273.3	131	1082.39	877.7511	2032.582	4269.235	6786.239	3289.458	4142.574	3374	3505
906	273.4	88	9193.39	1163.54	7304.749	12982.67	9167.914	2838.9	11197.17	7466	7931
907	274.27	121	76717.5	54457.59	70274.34	74555.48	72273.51	60193.73	61585.27	61441	86093
908	274.27	1038	2554.67	2737.875	2566.393	2413.249	2641.864	2335.23	2595.21	2514	3685

909	274.27	209	3437.17	2104.553	3178.051	3199.3	3052.973	2550.632	2894.731	2697	3489
910	274.27	766	404.035	282.5629	345.1175	333.6141	739.404	751.1661	320.1733	348.7	430.1
911	274.27	87	2555939	2559251	2491345	1971990	3358541	2102218	1978168	2E+06	3E+06
912	274.83	69	6304.16	3483.008	5311.449	2922.019	2347.745	4533.228	1827.209	5923	4196
913	275.15	63	677.833	1661.559	1622.531	1250.628	1870.026	1070.85	724.6255	1100	1606
914	275.28	87	484576	477315.6	469002.7	386809.7	859085.9	386348.3	390540.3	4E+05	5E+05
915	276.2	1038	1491.37	1558.486	1635.5	1620.799	2025.273	1653.796	1899.489	1610	1754
916	276.28	86	58210.4	51954.16	51939.86	46276.25	67022.28	50986.11	39814.91	49268	51529
917	276.79	72	2139.2	1365.15	878.9013	563.088	667.686	1957.031	1280.729	1571	966.8
918	276.88	63	2481.91	795.52	1135.715	966.8571	552.5607	1178.146	1178.058	1246	1269
919	277.1	63	3419.6	1800.941	2221.819	2841.89	1940.165	2795.278	2036.64	3004	2039
920	277.18	121	1114.07	2294.46	3875.163	3114.963	3201.637	2986.2	2648.612	2770	3055
921	277.28	87	3722.82	7561.211	2566.908	4339.749	7073.64	5674.307	5232.299	5378	4765
922	278.23	70	5520.79	3592.484	1997.146	3506.412	2527.058	1594.348	1645.431	1247	1431
923	278.27	105	1617.79	686.7	1472.825	1262.52	1655.363	1485.074	1238.091	951.7	1250
924	279.09	467	7020.49	6847.857	9273	5357.885	8889.042	8722.104	7855.978	9163	8841
925	279.09	77	13638.7	23849.03	1536.143	12059.75	10213.3	10554.95	10145.04	21310	12268
926	279.13	45	19565.3	19153.54	18058.01	24766.62	5677.252	20304.31	19621.59	19715	26841
927	279.16	1072	88070.9	156053.5	87997.76	121680.8	102594.2	119507	104062.8	1E+05	2E+05
928	280.09	90	31850.6	13609.04	88674.96	8633.076	12508.77	14237.42	18240.85	25103	17257
929	280.09	59	12296.6	3529.47	9272.357	1452.579	3688.627	6002.882	7386.975	5020	4838
930	280.09	111	11600.9	11192.12	11340.48	5418.935	4410.173	5700.25	5959.336	7056	13124
931	280.1	473	2502.95	2231.029	5626.773	2829.911	3134.393	3065.246	3165.377	3553	2804
932	280.16	1072	15385.2	28810.89	15293.06	20816	17174.45	20373.14	18489.58	26128	31350
933	280.26	144	3324.73	1568.865	3243.366	6268.416	5612.771	2747.875	3086.212	3604	4976
934	280.83	67	1440.05	480.1027	1155.344	166.2953	639.6	543.5954	974.0436	955.3	407.1
935	281.16	106	10476.2	6176.988	14094.42	6331.5	12091.11	6912.219	7101.254	9177	8941
936	281.16	1072	2183.49	2719.104	2677.883	2634.299	2549.66	3475.157	2988.857	4041	4799
937	281.23	112	1092.04	1015	1094.36	2071.266	7412.047	1807.968	1409.915	3383	2498
938	281.25	140	1712.62	1673.346	2118.278	2336.057	1150.944	1359.802	1784.35	1757	1853
939	282.14	79	5891.88	3184.436	4375.993	5430.618	7052.484	5682.486	3977.855	3603	2705
940	282.17	106	2183.97	968.86	1330.316	1926.54	2110.599	1677.202	2117.214	2168	1354
941	282.19	60	2516.01	1541.796	1404.953	1249.199	2990.498	1303.905	1921.024	1840	2088
942	282.28	180	16130.1	4800.012	13101.82	14115.68	15986.27	8369.237	16105.74	17656	16470
943	283.17	62	4174.48	2858.897	3813.744	1206.459	2141.93	5021.715	3318.02	3529	3389
944	283.28	181	3336.9	1042.871	2872.539	2355.85	3159.656	1675.523	3291.908	3650	3389
945	284.21	60	2102.1	999.8809	1846.71	1078.103	2454.879	1354.734	1073.614	1839	1059
946	284.29	111	1217.35	1233.496	1002.14	845.46	1400.264	807.315	990.5672	648.9	1040
947	284.29	249	8575.41	5839.762	7658.75	8213.612	7260.371	6343.254	5090.575	6597	6487
948	284.29	193	4527.44	2197.192	2815.402	2591.668	2558.08	3052.851	1546.544	4755	3826
949	284.33	173	2579.88	1619.477	2387.117	2525.053	5080.447	2586.744	1807.069	2855	2648
950	284.91	80	5319	3118.92	3518.152	2309.46	2320.71	3166.659	5628.196	7014	3552
951	284.91	62	2404.24	1555.088	1373.85	930.7054	546.8264	1665.227	1316.653	1470	849.7
952	285.13	74	1941.66	1370.944	915.0524	2124.06	1645.874	857.6462	1016.34	766	1770
953	285.3	252	1608.07	1296.046	1729.626	1788.228	1626.618	1138.722	1797.377	1483	1474
954	285.9	68	7937.39	4189.087	6039.988	3954.646	2939.447	5953.936	5329.62	6168	5341
955	285.98	89	2293.07	521.2815	3075.579	760.032	720.261	717.0825	657.825	2052	1247
956	286.24	120	6252.14	5835.838	5057.977	2910.083	5340.462	6919.433	4912.19	1376	5808
957	286.25	1034	1792.63	1723.92	1826.563	1728.85	1768.072	1623.016	1848.922	1926	1315
958	286.27	123	10695.4	694.69	10704.38	8820.459	8325.324	12935.87	9755.119	10115	11972
959	286.31	159	4407.98	1676.129	4965.05	3239.381	3155.551	4260.489	2587.024	6136	6405
960	286.31	97	7274.77	6042.824	7673.557	2538.9	5883.405	7687.727	5827.519	5374	7785
961	286.77	72	2368.86	1460.789	1859.213	1016.595	604.2467	1853.169	1484.786	1769	2049
962	287.22	1092	2156.16	1882.416	2394.638	1381.371	2085.16	1828.043	2141.046	2139	2066
963	287.28	123	2021.39	602.9333	2510.869	1864.094	2014.178	1295.769	2290.727	2671	2732
964	287.31	97	2202.48	1031.054	1061.247	1125	1712.317	1746.302	1106.116	1266	1469
965	287.9	69	4676.2	2075.013	3925.722	2548.7	2213.994	3787.866	2271.996	3550	2786
966	288.24	68	4712.22	3965.566	2021.413	3970.179	2510.3	1081.692	1261.44	1137	1426
967	288.29	1042	1962.98	2393.124	2358.565	2756.22	3690.932	3638.958	4869.648	3915	4601
968	288.29	92	256231	208009.9	364392.5	630589.8	474725.9	370439.2	418666.9	4E+05	5E+05
969	288.77	72	2385.39	1852.687	1520.96	1743.12	732.4968	1809.267	1154.686	2356	3452
970	288.92	1111	1399.54	1960.062	1546.193	1005.953	1598.94	2203.681	1160.181	1508	1415
971	288.92	93	3108.56	4106.886	3119.663	2284.9	4901.813	4502.401	3960.898	2000	2793
972	289.16	65	1350.27	2384.229	1250.388	1499.015	1931.243	1986.871	1533.107	1512	1209
973	289.29	93	51163.3	42025.74	81668.28	132235.7	102264.3	81064.82	83893.34	67322	96264

974	290.27	73	44316.4	24531.2	17605.7	22852.23	26918.66	20450.58	32095.53	34015	33193
975	290.27	101	112351	63793.26	78424.53	66824.34	117529.3	66158.52	37281.96	57020	87023
976	290.77	72	2144.96	1345.498	1599.935	998.325	311.43	1106.385	826.875	1111	2194
977	291.07	64	7399.54	7163.12	8661.184	2205.377	2164.562	2846.139	3588.76	3124	5835
978	291.27	105	24324.7	16294.73	17721.4	11009.33	24627.22	14164.46	8990.868	2908	10730
979	292.23	1054	2415.31	2573.657	2464.95	2295.294	2633.926	2501.785	1339.754	1893	2863
980	292.27	107	1958.36	3061.237	1984.638	3217.221	1540.14	2485.526	1037.797	1082	1806
981	293.17	469	1292.62	1085.456	1301.483	2304.166	1608.369	1553.206	1994.398	1737	1774
982	294.2	97	2421.06	991.6415	1722.749	3105.833	2766.425	2349.769	1748.87	2367	3761
983	294.21	1039	1855.63	1899.769	1890.681	1637.778	1992.381	1964.335	2222.983	1954	2211
984	294.24	119	1756.74	3309.113	1224.741	1603.988	1986.429	2902.043	2149.338	2101	3200
985	294.94	84	25546.8	29101.07	26927.66	18129.58	27969.75	12991.51	18633.49	23487	23196
986	295.12	79	10024.9	7607.372	8649.083	9909.559	8774.227	7582.605	8588.033	9796	9218
987	295.93	62	1620.02	811.0178	977.725	738.1386	631.5308	993.819	991.464	1048	1131
988	295.93	80	2653.67	1287.865	4071.176	1547.523	1983.771	1519.02	1794.283	3051	3370
989	296.06	101	5605.39	3300.882	12726.71	1624.493	3060.466	1706.04	2930.791	5053	7996
990	296.13	73	2577.05	1642.5	2403.51	2510.677	2307.783	2410.712	2225.973	1494	2209
991	296.18	106	1630.75	1075.193	2576.374	1461.6	1958.08	1576.278	1755.806	1396	1082
992	296.26	88	58579.3	76163.64	57531.14	23018.45	82036.12	77628.36	55385.96	61033	81853
993	297.13	74	3457.3	2289.778	1299.739	3190.84	2711.311	976.641	1743.257	2197	3223
994	297.26	87	13887.9	15291.64	12951.11	7151.69	16319.93	18313.14	13048.39	18510	18621
995	298.02	84	3024.61	2019.473	1639.936	510.98	512.14	1197.008	771.3014	1760	1033
996	298.1	64	3260.75	2496.695	2751.115	887.0133	1476.218	1212.68	1172.788	1361	1950
997	298.26	86	1709.85	1471.68	1959.494	2502.495	1513.26	2393.528	2033.64	2477	1727
998	298.31	402	2385.09	3805.508	1825.369	4052.694	2851.886	4847.803	4469.004	4296	4187
999	298.31	237	2422.95	1760.559	2802.116	1905.935	2831.045	1448.241	1875.374	2329	1923
1000	299.15	65	15489	4565.662	5577.845	4102.754	28767.11	2570.49	6212.827	8644	8032
1001	300	89	12942.7	6010.331	15380.67	5214.852	9462.536	6658.07	7207.2	8562	10587
1002	300.2	70	7745.29	4521.337	5074.688	1411.8	6170.116	12469.9	1816.56	9211	8821
1003	300.25	131	5586.47	3139.598	7393.448	4337.871	6033.844	4618.333	3826.62	5011	5514
1004	300.29	145	4389.77	4668.343	3677.114	2960.707	3351.04	3847.788	4616.568	3401	4118
1005	300.7	72	1831.64	739.0073	1290.251	2029.984	1315.902	2449.722	1755.049	1683	1563
1006	300.88	77	2758.35	1605.641	2461.436	543.3602	851.7706	993.3694	2256.018	2425	2154
1007	301.14	1117	8816.37	3889.3	4749.297	3835.445	4303.623	4243.027	4092.014	4366	3850
1008	301.14	1198	1879.13	19089.55	4596.238	0	0	0	0	4226	4210
1009	301.14	1150	1340.88	1336.995	4941.934	1045.209	1762.118	2010.027	253.184	2184	1448
1010	301.14	106	157750	118481.3	223233.4	107935.8	177838.3	147398.2	118552.6	2E+05	1E+05
1011	301.14	1072	25634.1	22955.99	26431.5	29414.22	29109.85	29467.53	25009.88	39489	27665
1012	301.28	135	1227.9	1154.629	1561.582	1927.196	1198.224	1179.662	896.0133	1004	1222
1013	301.29	86	9180.76	2154.149	1398.638	6532.5	13436.68	3314.176	4503.277	5978	4470
1014	301.35	107	1294.94	1101.012	685.4457	1062.18	632.7111	602.103	1364.332	1237	874.2
1015	301.88	68	2334.41	1364.4	1770.537	888.7442	856.86	1162.438	1206.144	1547	1144
1016	302.14	1102	1383.68	1173.571	1681.365	1542.745	1476.973	1538.498	1435.118	1464	1534
1017	302.14	1072	4634.63	5437.102	4668.583	4334.565	5918.94	5545.651	4881.655	4756	4672
1018	302.14	106	27010.7	19537.45	42475.23	23492.02	31737.89	26351.08	26181.2	40929	24817
1019	302.19	61	8379.31	6227.172	7206.889	4435.748	9952.828	9562.577	6775.112	7989	6041
1020	302.24	68	8443.1	6429.573	817.6	3623.353	4401.17	1547.409	1785.866	2016	1585
1021	302.27	111	2159.48	128266	2450.967	82315.19	1626.023	1324.392	110778.5	1E+05	1E+05
1022	302.3	106	136455	130304.6	139891.7	84824.46	147043.3	95964.35	121469.9	2E+05	1E+05
1023	303.15	107	4828.23	3377.205	7691.68	3529.26	6654.678	4553.408	2680.427	5589	4474
1024	303.2	62	1862.5	1707.43	2083.65	2483.223	1378.157	2014.348	1128.862	1402	1931
1025	303.3	470	584.361	299.7001	2635.701	4422.55	1472.652	860.0188	1111.765	1245	1835
1026	303.31	107	28565.9	25953.33	31551.02	12590.75	34157.77	22168.6	25433	33402	27556
1027	303.87	68	1735.25	629.9818	1470.254	1043.97	544.5936	1228.985	782.5714	838.2	1888
1028	304.17	73	1770.69	1882.841	1835.326	1298.113	2649.162	2463.99	1641.693	2267	2040
1029	304.28	120	10874.2	6084.94	16073.1	23688.57	14596.07	12268.02	11577.08	14362	25008
1030	305.16	63	8677.07	3956.353	6703.122	3057.508	4775.724	9978.375	6187.382	6770	6751
1031	305.29	121	1606.73	1842.953	1395.381	4643.131	2060.836	3021.945	3531.819	5223	5592
1032	305.3	105	1992.04	1221.81	2163.836	760.1771	1742.14	2304.123	1009.985	1309	2285
1033	305.96	82	9827.52	8350.213	12384.96	4862.551	7851.747	4882.931	5486.611	6396	4503
1034	306.16	62	2026.64	1637.366	1011.992	1293.981	999.648	1385.875	926.8852	1014	1264
1035	306.26	68	14680.1	11123.67	6211.27	7270.685	10828.52	3324.409	7433.364	9571	11114
1036	307.04	66	2686.83	3002.88	5584.013	736.1947	1178.102	935.6271	1857.138	1474	2590
1037	307.08	893	4028.49	1550.875	2178.513	1243.13	1791.833	1185.21	1598.538	2232	2932
1038	307.27	68	2624.77	1656.154	1171.563	1464.178	2033.3	812.0561	1840.155	1582	1805

1039	307.58	894	1059.97	492.5579	663.2141	331.256	526.3926	350.668	516.2204	669.8	846
1040	308.09	506	2061.95	1241.489	2653.41	993.9303	933.345	1750.163	1230.198	3769	1487
1041	308.09	438	8319.47	7261.474	8605.232	3956.832	2553.682	6334.659	3668.776	4350	6349
1042	308.25	116	1243.69	1832.805	1475.434	735.5517	2257.657	1484.633	1125.227	1184	1873
1043	308.95	86	5781.7	6700.958	6370.112	5089.173	6830.149	3848.763	4774.439	5366	6371
1044	309.09	390	1356.22	1713.712	2594.308	2396.359	1818.497	2555.644	2923.31	6107	2773
1045	309.13	77	4446.4	2032.39	4030.922	1875.651	2903.003	2913.756	3421.969	3769	2690
1046	310.27	94	6711.12	5476.749	7011.145	3526.18	10495.3	6725.193	11687.59	10365	14528
1047	310.31	267	2802.8	1338.801	13687.09	7238.438	3339.266	1916.754	5267.824	5192	5229
1048	310.87	63	1699.71	867.84	1130.077	811.2857	522.6574	653.3057	842.72	706	1138
1049	310.91	83	11735.5	11039.39	16069.16	4579.816	12274.49	6604.358	5351.684	9145	10288
1050	311.13	70	1102.4	1070.955	1051.543	945.1727	1643.904	1249.236	934.03	1452	824.5
1051	311.17	113	1445.62	999.3415	2491.938	1895.885	1077.757	1274.52	1185.877	1167	1423
1052	311.27	95	1300.2	1579.172	2036.204	5137.565	2054.349	1622.514	2076.84	3273	3406
1053	311.31	267	640.542	406.5116	3174.471	1808.136	765.5457	664.0343	1197.164	1332	1338
1054	312.01	87	8736.56	3492.976	8214.561	2736.25	4876.964	3637.197	3480.127	6199	4768
1055	312.23	86	4196.79	1416.158	4447.377	2453.489	3577.368	5132.104	3256.674	4465	3464
1056	312.25	102	6212.81	4799.925	2151.956	4999.757	4398.883	2767.965	2324.885	3887	2712
1057	312.33	427	5004.14	5682.299	7175.463	4828.688	5462.748	6473.342	7744.642	7038	6182
1058	312.33	450	1235.67	898.8398	3008.475	2441.856	2250.932	1403.769	1951.829	2084	1923
1059	312.33	310	1820.65	1984.057	2359.214	1767.224	1212.01	1629.36	1848.787	1176	1699
1060	312.78	71	14299.1	9298.003	10132.19	7936.499	5769.717	12287.65	9560.261	12661	10839
1061	313	96	7352.35	1108.38	1724.861	2518.36	6010.041	491.6682	1552.576	1753	2756
1062	313.16	66	1059.77	1375.474	1700.189	1645.015	1358.5	1277.772	1177.018	1377	1707
1063	313.23	111	1006.86	1063.444	1822.007	1432.892	1351.035	1427.58	909.72	1747	1017
1064	313.27	177	3260.66	2389.238	2517.986	2302.793	2915.528	2835.345	3011.142	2924	3011
1065	313.33	427	1431.79	1434.903	1740.554	874.043	1409.437	1580.915	1450.12	1832	1163
1066	314.27	140	3240.73	1765.808	2688.458	1788.16	2951.538	2558.718	1571.877	3356	1855
1067	314.28	178	1230.35	2306.9	806.6618	5363.738	3299.455	4650.068	3909.266	1266	896.4
1068	314.31	235	2141.72	1121.207	2582.672	2410.72	2455.947	2199.269	1541.94	2784	2472
1069	314.34	111	1938.83	2394.81	2771.168	2321.923	2705.02	1594.222	1358.948	1008	3411
1070	314.78	71	25516.7	15975.31	14301.96	10844.26	9924.2	17127.14	17150.02	20605	19575
1071	315.17	114	2922.54	1611.888	4170.553	830.5484	1297.676	954.6611	1653.674	1138	1604
1072	315.19	66	864.129	881.144	670.81	1367.696	3170.704	1743.99	1096.895	2413	632.7
1073	315.27	140	862.219	766.6486	1067.067	804.6357	886.6667	514.6388	912.24	888.8	785.6
1074	315.31	173	1227.75	639.1182	1109.784	1199.021	830.2624	1367.897	1093.84	1118	1455
1075	315.32	114	808.573	995.7824	2090.423	1864.967	829.555	1969.5	1285.676	1241	893.7
1076	316.21	65	179137	144241.9	206824.3	189251.6	233778	271848.8	191698.1	2E+05	2E+05
1077	316.28	98	37437.3	21268.88	4407.464	49258.69	2526.451	3493.223	42944.18	49433	1516
1078	316.28	83	8044.99	4560.214	5729.207	3847.098	7850.332	3353.355	6509.488	4242	8125
1079	316.32	1076	3438.88	1916.56	3191.966	1742.259	3601.36	4089.099	2903.994	1778	3728
1080	316.32	117	194333	153343.3	208969.6	409111.2	326040.2	270213.2	255855.8	2E+05	4E+05
1081	316.78	70	16176.5	10246.94	9095.854	6740.454	7748.727	11485.54	12217.4	16343	13071
1082	317.11	1071	2844.64	3011.943	2910.164	2882.798	4318.216	3917.506	3092.742	3216	3929
1083	317.11	106	22348.3	16209.94	36180.24	15992.93	16689	17287.96	15068	22641	26560
1084	317.16	2	137.076	104.5409	527.72	104.7338	83.44835	111.166	434.871	618.4	98.9
1085	317.21	65	34800.5	30121.5	41440.08	38265.91	48187.26	51440.97	35819.15	42729	40755
1086	317.29	88	13513.8	7235.248	717.3697	5611.578	4264.89	4204.976	5416.891	5698	5917
1087	317.32	116	40429.7	37353.17	53223.96	96926.6	80891.91	63513.82	48922.77	71748	91493
1088	318.12	106	5668.71	3258.579	7455.049	4452.84	4510.08	4405.05	2822.795	4865	5714
1089	318.22	65	7057.16	4796.867	5882.183	7246.99	6720.95	8182.535	6656.993	7316	5902
1090	318.24	99	1953.53	3816.986	1884.464	1435.14	2399.495	1465.926	1574.005	1824	1580
1091	318.3	143	16462	13941.13	12256.34	13235.97	14979.92	4361.76	6561.86	7534	6724
1092	318.33	117	6154.59	5956.07	5733.44	13871.47	12287.64	9894.899	8597.729	8898	13933
1093	318.77	71	5877.99	4604.873	3659.411	4040.047	2191.413	4445.535	4002.318	5132	5368
1094	318.91	63	1443.47	645.74	872.928	471.7283	351.9951	638.0363	577.915	622	626
1095	319.12	107	2073.45	2298.49	1098.611	2017.26	3503.102	2196.744	2191.11	2504	2131
1096	319.14	64	1269.36	1773.261	1005.164	1628.129	1200.677	1503.06	1395.591	1286	1307
1097	319.19	210	1148.42	673.9935	1044.108	838.0587	1412.013	738.4613	1204.573	1345	710.7
1098	319.29	140	5399.31	4079.675	1413.459	6607.059	799.632	5299.83	4981.463	4659	1124
1099	319.3	291	726.291	569.448	5084.668	3087.32	866.5155	791.0547	2785.133	1771	1375
1100	319.3	86	302981	85275.72	71508.72	169921.5	403759.7	51962.56	142624.6	1E+05	2E+05
1101	320.25	95	5411.82	2178.358	1937.037	2303.75	5426.246	2378.8	1794.297	1530	1203
1102	320.31	86	40595.3	11320.74	12201.22	19066.71	53170.43	8308.058	20754.95	22726	27016
1103	320.94	64	758.944	297.99	631.26	350.4156	360.4386	212.1249	308.3733	463.1	464.6

1104	321.13	64	86229.8	14626.43	22493.21	22142.04	94247.13	10469.09	32053.22	34995	40902
1105	321.31	86	5789.94	1491.84	1795.275	2548.874	7126.2	468.2417	2301.997	4560	3745
1106	321.31	469	4665.57	2121.525	60885.79	34489.31	15695.27	7565.267	8165.465	20108	20661
1107	321.93	82	2761.51	1008.134	3074.807	1282.192	1537.972	2361.067	2257.574	1467	1744
1108	322.13	64	15402.4	3008.686	4043.023	5465.839	16128.57	2000.483	5755.358	6675	6429
1109	322.23	131	4334.84	2282.19	6015.393	3263.068	3981.099	3966.04	1281.443	5120	4057
1110	322.24	97	992.293	884.4209	1026.206	1880.719	1671.464	1427.594	2376.192	2142	925.9
1111	322.26	69	2902.54	2901.73	1135.784	1547.407	2914.625	617.474	514.5277	663.5	625.6
1112	322.32	468	1925.5	731.6928	10148.58	8952.886	3946.933	2346.68	3897.32	5780	5391
1113	322.81	65	4238.05	1061.538	2697.937	968.2073	1365.478	1854.233	2437.425	4121	1980
1114	323.14	64	1444.57	1322.815	1102.626	1366.46	2866.772	1022.007	1196.679	871.5	1155
1115	324.22	1072	42303.3	43621.85	68029.25	45896.98	53848.93	91333.5	87569.56	44986	45107
1116	324.22	1043	19926.4	20897.56	18397.13	15418.76	19395.21	21754.12	1774.776	19195	23021
1117	324.22	107	44829.8	83948.9	54715.7	54549.18	79679.13	50996.37	34504.69	42810	72278
1118	324.22	49	9616.81	8186.458	8717.68	15276.85	10196.22	9280.815	9382.357	10553	10816
1119	324.29	106	7620.76	7926.596	6636.426	6149.084	6805.566	4747.665	5784.393	7937	9021
1120	324.81	65	6296.02	1152.883	3954.308	996.8778	1603.275	1691.379	2447.444	4556	2181
1121	325.04	343	1779.43	976.2984	1977.048	939.25	747.8058	750.3321	1001.664	1439	1290
1122	325.04	302	6504.63	2218.283	8754.998	2458.136	878.4927	1911.193	9359.642	3536	3079
1123	325.04	323	4419.56	2059.495	3228.226	1623.332	1006.764	649.4806	2377.926	2318	3258
1124	325.1	79	1062.16	1541.145	3856.698	1032.4	2532.862	1557.966	2575.62	1646	1190
1125	325.22	469	3684.77	3544.829	3220.325	4275.367	4242.908	4462.253	3850.023	4700	4819
1126	325.22	2	894.045	590.3857	632.0754	506.7789	570.6248	656.9876	217.296	751	681.7
1127	325.22	1072	9202.04	9675.368	14679.33	10842.24	11401.08	11945.15	11009.62	9445	9830
1128	325.22	50	1666.99	1205.592	1650.191	951.2721	1888.949	1592.021	1560.377	1474	1635
1129	325.22	107	9690.78	19243.87	11982.08	1055.88	19030.35	12291.69	7822.892	11392	17560
1130	326.21	58	2448.51	1442.064	1413.891	1045.123	2641.553	1095.05	1655.926	1661	1561
1131	326.22	1072	1721.09	1963.843	1810.366	1941.823	1887.007	1423.508	1979.364	3080	782.5
1132	326.22	107	2592.17	3189.932	3570.951	1960.395	1797.175	1780.723	1734.315	1971	2634
1133	326.3	133	802.604	442.2322	1376.813	1157.684	1142.4	808.3364	693.387	1244	951.3
1134	326.34	400	3176.72	3570.477	1932.751	3384.065	3120.878	4528.428	2746.231	4245	4200
1135	326.38	144	594.357	459	1225.402	610.9911	1188.72	355.52	779.0978	908.2	712.5
1136	326.8	65	2689.65	515.4236	1533.542	742.3571	638.7502	1011.944	1821.498	2463	667.1
1137	327.01	1042	9983.47	7516.425	5145.404	3098.244	6564.439	7772.32	7205.419	7796	9851
1138	327.08	120	2911.04	18442.34	18385.47	13234.29	18035.26	18974.93	13749.51	16879	19515
1139	327.2	61	3885.26	3395.637	2394.352	958.7348	3656.404	6163.799	3242.997	1651	3288
1140	327.2	122	2811.18	2096.738	2037.261	3272.391	2766.189	2615.488	2002.605	2334	2671
1141	327.34	148	17036.5	7581.125	25493.98	1065.13	1548.74	1823.957	4696.398	6843	6699
1142	327.34	210	773.194	356.662	793.9329	332.4062	493.8187	373.9636	394.8692	355.8	417.9
1143	327.34	401	707.934	660.1541	746.8416	571.3874	713.592	816.2322	546.4526	827.4	1096
1144	328.08	82	2203.18	4748.241	3405.949	2212.015	3501.45	3254.815	3444.688	2233	1856
1145	328.08	470	5142.98	4772.305	8237.662	8185.859	6257.742	6343.891	5035.918	6625	6453
1146	328.08	2	902.876	615.5469	787.8596	230.109	540.5999	663.299	674.1043	794.1	798.6
1147	328.22	103	837.747	1548.73	1304.522	875.005	1408.46	921.17	855.1087	820.3	1127
1148	328.23	61	1426.22	1259.604	871.1093	1683.347	1097.728	1017.544	2288.493	1647	2610
1149	328.32	213	2734.99	1293.324	2700.47	2002.226	2867.657	1758.939	2045.756	2440	1800
1150	328.34	147	4721.23	2105.678	6040.575	938.4556	766.1083	899.4737	1736.33	1760	1083
1151	328.76	71	4954.08	3070.168	4449.615	2712.063	1913.6	4536.693	2752.916	4055	4791
1152	329	111	1677.41	6455.541	5162.637	4836.351	4737.064	4625.453	4678.788	4785	4698
1153	329	468	1365.13	1185.043	2614.286	1842.763	2157.764	1597.248	1595.953	2283	2047
1154	329	1073	2390.95	2312.22	2545.757	2363.138	2461.157	2674.184	2236.14	2583	2301
1155	329	141	3202.77	3063.64	3092.176	3176.389	3117.943	2484.842	2644.4	3255	3316
1156	329	1042	8470.96	7639.615	9079.389	3353.4	7701.778	7216.839	7276.862	7877	8057
1157	329	1177	2430.06	2271.458	2096.317	1966.806	2424.171	2476.766	2077.483	2827	2175
1158	329.09	107	819.347	771.6138	607.776	735.84	664.7946	360.3444	549.88	847.8	745.9
1159	329.16	65	2376.06	3027.643	1904.148	1599.029	1864.443	2164.689	1342.889	1378	2239
1160	329.26	136	765.889	885.9644	1232.552	1485.57	1855.47	1106.634	1374.474	1082	1196
1161	330.07	377	12441.1	2962.418	4354.477	3309.589	2229.912	14496.14	3327.31	4675	3713
1162	330.19	70	4120.61	3824.625	2520.893	6358.338	3123.758	9351.355	3410.466	3243	6285
1163	330.23	71	5044.33	4382.43	4675.402	7727.967	4406.637	9056.724	5021.57	5590	6645
1164	330.3	110	2450.49	1993.838	2085.322	2251.588	1213.382	1116.766	1371.194	2693	1306
1165	330.34	140	108010	166100.7	91006.91	140185.3	150283.8	97624.28	1098.064	1E+05	2E+05
1166	330.75	71	9506.55	4510.329	6678.442	3079.915	2921.578	7663.6	6011.038	7361	9012
1167	331	1042	2618.29	2675.933	2263.472	1627.462	2800.057	2321.208	2712.243	2418	2493
1168	331.14	81	3862.79	814.7783	1867.889	2396.187	6368.921	1917.921	2445.782	3444	2283

1169	331.21	71	1231.83	2861.575	1471.806	2136.955	3997.896	1672.324	2693.088	2012	2974
1170	331.28	177	3025.68	2154.613	2145.751	2146.365	2818.328	2370.39	3082.737	3269	3239
1171	331.34	140	24964.8	40664.11	23629.16	34089.84	34776.1	22542.61	27316.43	31673	38035
1172	332.21	71	1329.97	963.9867	1231.729	1861.584	1848.315	1185.654	1217.462	2006	1726
1173	332.28	1091	2544.6	1631.112	2909.006	1815.554	1656.652	1247.355	2384.976	1907	1826
1174	332.31	92	8089.74	7632.123	9868.866	17021.98	16827.81	10094.05	9513.436	9282	14325
1175	332.32	177	7805.02	2836.397	4305.765	11386.37	6708.853	5775.77	4031.775	10418	7542
1176	332.33	133	49010.7	7963.847	63604.87	21952.44	33162.95	35030.2	28361.34	48117	48662
1177	332.75	71	5651.83	3482.074	3292.573	3337.871	1543.1	5294.442	3173.575	4807	4947
1178	332.84	63	7745.28	1579.763	3076.894	2139.534	1073.732	2773.451	3074.254	2850	2884
1179	333.16	118	2002.15	675.675	1307.922	915.4884	802.5	2311.676	977.2933	1514	990.6
1180	333.19	65	2314.44	2648.466	1922.785	2392.803	962.6059	2974.568	1087.35	2211	1505
1181	333.3	170	1745.66	1376.738	1592.186	863.4108	2476.375	1653.5	1941.116	1910	1719
1182	333.32	93	2286.84	1965.557	3415.276	6373.389	3647.176	4531.725	2575.581	2654	3035
1183	333.33	134	13856.9	2196.928	16291.25	5543.893	8045.035	8986.535	11185.9	15217	11951
1184	334.29	74	31426.4	716.8936	9893.657	17449.49	19539.09	4708.447	15812.21	17901	16719
1185	334.31	213	3212.37	1137.834	12050.92	9275.688	6183.501	2169.161	5459.804	6991	5587
1186	334.34	133	2268.03	1189.15	2793.857	599.72	830.3657	1138.091	1342.049	3169	1502
1187	334.83	71	2585.59	2017.513	1662.08	1311.092	1877.2	2678.236	1178.332	2370	1021
1188	335.07	465	5791.59	2839.702	12502.09	1481.545	2461.852	2917.22	2977.078	2730	6007
1189	335.07	443	6274.15	6968.685	7773.277	1396.461	1943.445	2050.911	1994.613	2272	1367
1190	335.09	64	1784.57	1340.683	1813.272	941.0613	1347.323	910.4436	1002.183	1141	1370
1191	335.1	117	2523.13	1204.058	3180.044	568.4774	714.7835	482.6684	2094.784	1626	1005
1192	335.28	113	3069.66	1115.535	6147.865	1976.713	7073.949	3767.52	2945.436	4978	4685
1193	335.29	147	1592.52	1067.037	2820.704	2318.14	1533.307	3185.873	1885.132	2515	2944
1194	335.3	72	5033.6	2041.636	1485.981	1019.231	3273.4	1283.287	2025.627	4420	1007
1195	335.3	98	7648.69	4559.566	5005.131	8084.447	11897.85	716.43	2050.727	4750	3793
1196	335.31	213	681.318	345.5325	3116.904	2634.246	1589.652	617.303	1133.943	1763	1474
1197	336.25	140	26171.5	13136.22	52306.7	977.9	920.7576	1380.769	4496.969	41994	46913
1198	336.3	95	1806.56	1302.863	599.412	1246.25	898.4607	643.28	778.5367	445.5	1094
1199	336.31	112	2497.3	1040.469	3096.746	3327.64	1800.376	4709.864	2380.821	969	3546
1200	336.31	1045	1153.4	1455.605	1336.871	936.572	1222.887	1233.024	976.3142	1287	1045
1201	336.32	140	25512.2	12462.11	50499.27	47963.89	86203.74	57468.38	31134.85	40156	46727
1202	336.32	310	1331.75	789.2867	4891.68	5082.787	2322.516	1081.903	2919.719	2530	2635
1203	336.32	193	2394.36	950.7863	3194.438	3000.162	3288.213	1693.232	2041.238	2855	2563
1204	336.33	290	6903.93	4938.572	58723.38	30405.88	6631.44	7311.727	28331.36	20677	15775
1205	336.83	63	3529.8	1305.066	1384.219	749.8533	964.0435	915.3475	1391.644	1371	779.7
1206	337.11	64	36501.7	6858.905	9034.14	8862.653	29859.2	4078.007	12060.7	12615	16758
1207	337.2	118	25690.1	7613.637	18932.51	5692.263	8184.107	14053.54	7193.16	9795	17481
1208	337.21	1071	1438.22	2460.945	1398.381	1493.289	2199.696	1484.608	1484.039	1445	1360
1209	337.27	469	1370.5	523.4843	7677.829	2947.604	1328.21	1465.11	2184.965	1240	2897
1210	337.31	111	1044.95	985.2159	911.25	900.9	1003.384	1132.364	1024.424	1259	1094
1211	337.33	140	6504.17	2814.96	13796	14202.94	20913.01	13911.45	7903.413	11608	12302
1212	337.33	310	224.839	178.5852	1234.025	1205.72	473.8992	212.9413	675.0861	512.6	530.4
1213	337.33	290	1719.46	1336.176	13455.97	7573.455	2031.492	1625.546	7241.866	5078	4011
1214	338.11	63	6748.25	1301.88	2137.5	1565.36	5475.542	888.5963	2434.373	1795	3152
1215	338.2	119	8105.7	1255.514	4220.964	2476.023	2387.783	4083.999	3270.389	3188	3738
1216	338.25	107	3352.25	1860.5	2599.942	2757.203	3031.407	1609.215	1569.246	1925	2803
1217	338.27	468	235189	109891.8	1758.446	1065.9	1584.865	324514.2	435122.8	1630	958
1218	338.34	1051	8120.92	7918.453	7392.553	1689.136	9611.756	9072.636	9229.937	13979	14342
1219	338.34	469	287355	99495.28	2345362	979015.7	672976.9	354923.2	438430.1	9E+05	9E+05
1220	338.78	67	1427.75	293.44	1360.767	457.312	569.4	637.318	1005.549	1352	476.5
1221	339.1	64	3384.22	685.8529	1237.486	1962.995	3332.689	972.7533	1236.17	1154	1696
1222	339.32	117	1271.33	2533.121	1158.1	2828.432	3401.089	3938.522	3420.8	2495	3404
1223	339.34	97	1501.2	1691.25	1206.022	2560.34	2399.898	3082.723	1540.673	833.6	2244
1224	339.34	1075	1770.71	5104.783	1648.309	1554.57	1674.218	2203.627	1393.655	1610	1798
1225	339.34	49	1446.5	1634.913	943.2215	2436.027	1385.924	1599.651	1560.566	1102	1703
1226	339.34	144	1499.66	5050.206	2174.331	4606.13	5499.921	5665.393	2046.728	2748	4738
1227	339.34	1053	2280.1	2573.456	2420.55	2026.983	2521.79	2213.224	2863.208	3636	2604
1228	339.34	1197	899.925	1924.165	1281.308	1240.725	2436	440.16	1171.95	1395	1450
1229	339.34	1179	2437.27	1632.348	2167.13	1928.281	2438.598	2586.793	1387.945	3126	1828
1230	339.34	1098	1574.06	1277.821	971.1429	1659.033	1496.25	1876.947	1647.425	1574	1987
1231	339.34	468	51581.6	12891.28	614401.3	294106.4	172355.6	87610.78	127861.1	2E+05	2E+05
1232	340.21	1044	2547.08	2450.249	1791.875	1765.007	2957.398	2354.48	2135.68	2117	2747
1233	340.28	87	166606	68665.05	43704.68	95110.4	195679.4	51875.72	112161	1E+05	1E+05

1234	340.32	148	1231.26	980.48	1278.757	1376.652	1362.068	946.5235	1162.951	1371	1350
1235	340.35	468	9208.29	2165.565	87511.15	39937.7	23084.98	13904.87	6258.522	28813	26658
1236	340.36	1147	5882.06	1898.947	17054.45	9162.533	2328.94	6763.81	3969.225	15814	10437
1237	340.36	618	2122.38	1188.206	2223.256	747.4412	1591.104	1599.431	1878.872	1390	1792
1238	341.09	49	12397.3	11819.39	12827.22	10395.78	17057.91	12686.05	12525.18	15067	13694
1239	341.19	76	2513.91	2724.437	1025.499	2828.702	1506.654	1766.246	1974.568	12010	1901
1240	341.28	87	38889.8	16899.71	12461.35	25655	42764.76	14103.64	27416.35	27626	29889
1241	341.35	468	1261.68	535.96	8436.208	3388.282	2531.263	1312.22	1313.024	3796	2235
1242	341.36	1146	1439.75	499.2586	4634.772	2413.927	1776.504	1272.36	2679.337	4076	2689
1243	342.09	90	4373.14	7146.921	7446.06	6692.893	6243.96	6620.497	5435.863	5726	6147
1244	342.1	466	4462.33	4046.727	7877.713	5216.797	5416.028	5420.899	5022.25	5567	6013
1245	342.2	65	1302.69	2224.141	1476.779	1659.999	1546.441	1766.698	1552.398	1929	1247
1246	342.29	88	5868.95	2769.772	2309.766	5870.913	9604.263	1638.12	4383.505	4548	5710
1247	342.31	264	938.228	1032.604	922.6633	983.4353	922.2061	860.0965	839.3715	637.6	1107
1248	342.37	144	6539.66	9033.262	7004.638	9729.844	8071.864	6645.419	4604.568	6762	6694
1249	342.87	78	10611.4	6437.456	12735.36	3662.536	3810.903	4840.458	13004.95	15348	4801
1250	342.87	62	8767.19	3859.68	4262.606	3132.17	1242.313	4425.53	4411.134	5082	8101
1251	343.15	1069	2731.99	3615.288	2376.192	2368.115	2809.016	3537.196	2857.559	3209	3420
1252	343.18	97	1113.79	3102.732	921.2181	1232.856	1457.148	902.79	725.76	2115	1731
1253	343.37	143	2096.77	2493.485	8189.521	2295.734	1973.747	1265.846	2144.945	1780	1687
1254	343.86	71	5806.62	5556.596	4906.655	5959.503	3206.549	4724.63	3691.483	5093	5207
1255	344.09	102	650.128	554.71	877.245	971.948	452.2346	653.9	703.6148	752.2	880.8
1256	344.21	84	1635.67	1909.154	1280.16	2762.208	2761.92	2131.872	3757.32	987.6	2232
1257	344.25	60	9980.75	7682.995	7986.259	3954.839	7799.613	12648.7	6568.409	7095	7567
1258	344.27	1	1083.3	738.5267	1484.375	448.3511	904.288	974.2412	622.9946	1290	685.6
1259	344.28	121	3384.37	2504.963	5723.337	1210.874	3770.203	3939.445	4411.673	1290	4731
1260	344.28	1166	4222.74	5932.667	4046.808	4024.413	5561.715	4009.167	2886.941	4472	4838
1261	344.28	1055	22831.6	8191.061	24322.56	5529.414	11611.01	9795.004	24242.45	10289	16588
1262	344.28	1119	3395.5	3447.36	3771.203	3264.555	4125.52	3419.728	2134.941	3765	3724
1263	344.28	1092	6177.96	6151.253	6065.808	6489.379	5676.57	6339.047	6476.8	5717	6848
1264	344.28	1182	17468.6	8292.473	19867.92	9453.402	16413.6	17593.43	7683.618	17912	12584
1265	344.28	1198	3055.52	7323.943	2400.35	3328.944	0	0	0	2481	5616
1266	344.28	1144	4600.92	4766.161	4282.051	3541.32	3677.292	1624.009	4039.7	6637	4329
1267	344.28	208	3843.41	2759.363	3645.16	3653.505	4381.329	3549.65	2910.884	4061	4175
1268	344.28	142	6608.31	9783.148	2950.35	5779.66	4421.927	6864.412	6712.784	7922	4814
1269	344.31	86	30435.1	6517.163	10196.04	13880	36605.33	4619.928	19320.9	19337	15779
1270	344.31	158	2829.36	3359.824	3818.1	2423.002	3676.124	3852.226	3955.15	5393	3276
1271	344.35	467	1878.11	1711.208	7651.461	3595.741	1766.535	2186.669	2426.434	2952	2574
1272	344.86	62	6315.26	3117.93	3048.417	2378.689	856.4325	3157.298	2960.393	3481	4365
1273	344.86	78	7400.36	4389.811	9206.038	1875.651	2760.603	2549.724	7487.051	11812	3535
1274	345.17	63	1544.22	2769.022	927.048	899.9404	976.7966	1212.629	1315.081	1993	1137
1275	345.28	2	346.971	243.5167	408.2622	201.4819	280.1544	286.5697	381.9596	778	208.2
1276	345.28	1051	2637.51	2242.134	1454.932	2164.979	2910.793	4763.483	3156.933	5790	2452
1277	345.28	1092	1326.57	1394.061	1485.216	1414.638	1469.236	1653.394	1325.363	1539	1719
1278	345.28	1077	1769.38	1657.879	1764.423	1334.667	1559.777	1997.94	1722.606	1736	2164
1279	345.28	1114	775.463	912.2263	1243.513	685.8446	732.8029	730.6761	735.1467	762.1	1001
1280	345.28	1183	3903.19	1974.892	5174.755	2377.275	3617.055	4039.058	2209.9	4028	4232
1281	345.31	133	4826.31	3228.34	1235.336	3487.67	1232.471	5460.412	1884.241	3567	4882
1282	345.32	86	5089.66	1842.161	3088.736	3821.214	12356.14	1098.927	4210.319	4250	6010
1283	345.86	70	6592.73	5255.615	5223.195	4561.687	3245.466	5907.652	3935.054	4621	5604
1284	346.01	109	223.983	97.8965	321.3013	6787.429	4040.583	1305.105	931.8142	796.2	1585
1285	346.22	61	4070.53	3194.517	4061.319	4426.948	4716.953	4458.186	3479.263	3552	4019
1286	346.26	1042	1609.41	1757.961	1426.774	1370.508	1578.536	1586.525	1677.127	1584	2210
1287	346.3	114	23896.1	30540.44	20691.04	22374.35	1035.296	22377.17	30327.04	32523	42437
1288	346.31	130	3212.82	1188.888	1427.304	2530.646	1390.383	2690.393	2477.025	3449	1603
1289	346.33	216	3515.18	2612.437	6662.221	3245.579	2897.568	920.2434	2616.876	2743	2388
1290	346.33	103	89144.5	68921.03	79968.94	52774.57	78311.4	51391.08	57603.94	85897	73482
1291	346.73	70	1229.56	379.08	778.488	497.0669	351.33	770.946	490.2878	948.7	497.4
1292	346.86	61	2226.82	844.6	738.5467	604.52	186.72	864.4717	618.57	804.9	1015
1293	347.02	309	2359.98	700.8066	2985.407	698.6283	486.7368	550.8196	1035.007	779.9	868.7
1294	347.02	286	1570.13	485.0837	1411.987	467.7998	308.0462	412.6593	913.7178	739.2	657.8
1295	347.22	64	2054.65	1739.567	1928.962	1995.142	1427.707	1444.992	1831.105	705.3	2007
1296	347.33	103	20753.7	17213.14	20312.78	10820.88	19787.21	13459.33	20377.95	21425	16371
1297	347.85	70	1859.85	1530.727	1852.199	1205.565	1110.023	2284.48	1637.848	1974	2092
1298	348.07	341	23207.7	3656.877	85410.47	9055.094	4292.27	7328.7	5554.934	14618	10548

1299	348.08	89	12056.1	1968.048	22374.5	2557.514	5198.638	7356.205	4482.307	7036	6105
1300	348.31	80	9171.06	1584.929	1502.238	4877.915	12056.93	2479.04	2977.56	5128	5510
1301	348.33	107	3061.92	2720.864	1621.46	1090.368	2254.985	2236.47	629.72	3723	1883
1302	348.81	64	3598.04	1361.073	1695.623	560.7711	1004.917	864.6314	1193.96	3447	1237
1303	349.07	343	3460.83	891.8661	2268.711	691.4117	1015.576	1283.096	1386.778	2106	1444
1304	349.08	93	1542.27	1235.576	3283.641	595.9067	1393.667	2242.953	954.1933	1743	1354
1305	349.18	62	14073.4	6293.347	9293.762	3161.418	6021.156	13594.39	6315.122	8175	7835
1306	349.29	126	2536.69	1186.782	2661.084	1821.625	2498.946	3477.197	2214.874	3378	3437
1307	349.32	146	2257.05	1228.462	2838.777	670.6469	609.1721	537.914	892.9572	938.7	1022
1308	350.19	62	3446.06	1668.507	1285.744	1511.121	781.3	2540.466	1386.792	1533	1281
1309	350.29	69	8084.54	4981.785	3582.462	7150.951	7765.982	1525.324	1784.803	3670	4389
1310	350.31	127	2395.24	1843.172	2774.401	1411.274	2779.352	3952.982	1100.944	3608	2565
1311	350.33	169	1551.63	1995.601	1541.037	1326.593	1443.23	1361.398	1166.613	1596	1604
1312	351.18	118	11297.1	2238.375	8713.786	2953.838	2492.572	6754.213	4009.725	7207	5789
1313	352.05	398	1553.25	942.118	1869.913	1045.651	902.1031	1957.781	1166.242	218.7	2048
1314	352.05	463	1345.16	815.3239	2728.427	1151.857	1239.272	1367.005	968.6024	1540	1957
1315	352.05	378	1627.35	1438.635	1906.332	1785.041	691.8895	3646.048	841.6657	1970	411
1316	352.05	436	1130.49	873.6844	1622.26	791.3097	740.8435	1280.985	640.3102	1050	930.7
1317	352.24	113	3102.94	3450.792	6576.61	1903.143	7682.725	3884.517	3700.487	2356	3170
1318	352.24	1072	3235.27	2529.956	2046.24	1169.818	2458.562	2363.15	2393.224	1190	2027
1319	352.32	148	28550.6	18520.77	46847.27	38568.57	24362.96	61357.09	31721.42	33727	45789
1320	352.9	81	33747.4	39055.71	38078.58	17561.06	15092.23	20060.11	23050.56	42620	25635
1321	352.9	61	14050.9	7503.93	5496.211	4736.077	2121.528	6574.12	5624.752	6620	11632
1322	353.12	138	4820.71	2061.902	6019.727	1033.198	1311.44	1049.001	1594.68	4812	3463
1323	353.13	84	2503.17	1458.656	3587.22	1789.423	3338.05	2595.9	3505.858	3237	2028
1324	353.19	73	2293.93	739.9667	1136.788	2525.148	2059.795	1638.942	2151.824	1763	1946
1325	353.19	103	4623.52	4417.341	7099.257	2848.85	5418.149	5152.572	3389.808	6133	4564
1326	353.27	177	1778.77	1576.518	1204.701	1471.635	2099.665	1700.119	1503.949	2582	2338
1327	353.31	111	1168.22	963.4625	2005.704	1459.181	1683.127	1142.783	1037.846	938.1	774.5
1328	353.32	148	6813.33	4504.221	11945.14	9683.406	6456.871	14841.3	8970.938	9362	11482
1329	353.9	61	1356.96	1526.051	707.8333	549.11	316.3867	612.535	597.69	711.6	1191
1330	353.9	78	3015.78	1805.431	2168.317	2061.641	1287.77	1710.69	1300.089	2059	2068
1331	354.2	99	1430.08	1215.616	1335.2	2502.762	2150.643	1197.57	1377.436	1672	2887
1332	354.28	466	2035.07	1958.942	2908.579	1410.541	2724.273	2225.575	1058.373	2587	2810
1333	354.29	93	7094.59	3987.883	1887.124	5519.807	6560.033	4424.789	2870.368	6168	4956
1334	354.33	147	3925.24	2596.954	1442.1	2641.19	2716.763	2372.229	3355.757	3455	3231
1335	354.33	119	4673.38	1597.211	5683.946	2763.346	4323.657	3523.089	1950.312	5032	6157
1336	354.34	172	3251.34	1939.747	5005.852	4294.433	3566.697	2720.358	2672.618	3450	3985
1337	354.34	193	5403.28	2267.326	8919.809	7696.857	8615.626	4520.754	5448.972	6727	6682
1338	354.89	81	17857.5	10841.56	17551.49	7663.818	11905.75	5097.716	11916.99	13301	11276
1339	354.9	60	5060.57	2623.925	1802.243	2267.133	840.6411	2472.356	2248.126	2371	3306
1340	355.11	471	5654.32	5017.741	5897.135	6887.031	7076.751	7070.272	6110.707	7247	7015
1341	355.11	79	3035.35	7731.919	1539.64	823.6294	4459.319	1215.326	3137.835	4529	2708
1342	355.28	103	918.305	1810.08	1343.302	2257.308	2504.81	1610.829	1990.426	1228	2106
1343	355.34	121	1532.39	1330.831	1825.945	1649.297	1530.157	1420.993	2025.134	1281	2765
1344	355.34	192	1433.99	1008.998	3580.368	2035.662	2211.3	1463.254	2634.465	1821	1899
1345	355.37	467	633.701	304.1169	5210.804	3040.073	1706.37	935.318	909.8083	1132	1911
1346	356.11	472	1233.79	961.4466	2322.969	892.4871	1106.672	1396.027	1531.954	1957	1836
1347	356.26	85	24269.6	9272.133	9715.363	13632.66	18323.93	8998.643	19349.67	19577	18852
1348	356.28	98	12023.8	8485.007	5423.501	9750.566	9082.624	4481.713	2247.383	3601	6781
1349	356.28	1073	1302.27	1192.473	1259.706	1080.831	1014.895	1560.004	1270.784	1225	1692
1350	356.35	146	39442.4	49939.43	29803.54	46495.86	39176.27	36785.96	32688.58	39533	47918
1351	356.88	62	1301.38	1028.851	586.3192	694.3035	284.622	1139.552	574.5027	881.6	1594
1352	356.91	93	1584.57	2463.741	482.1943	524.4857	1658.25	1124.23	2425.267	1011	1716
1353	357.2	64	1473.47	732.9764	1316.43	2236.88	3557.341	1133.699	1131.276	1563	1342
1354	357.21	105	3033.09	2447.414	4123.9	2668.014	3984.417	3893.111	2721.372	2107	1893
1355	357.26	86	5888.4	2274.803	2565.646	3790.728	5264.15	2459.204	5664.335	5507	4404
1356	357.28	97	2789.76	2114.029	1815.294	2485.369	3376.928	976.9439	1730.116	1537	887.1
1357	357.29	136	1226.68	1473.817	2887.662	621.4833	1340.308	866.0492	1164.4	1119	817.1
1358	357.31	208	2267.96	961.7411	2985.57	920.1807	1744.53	1095.899	1822.724	2531	1073
1359	357.35	146	11014.7	11848.35	7393.408	11980.65	10218.86	9858.792	9332.112	10169	12802
1360	357.71	103	1217.51	179.2324	1326.78	942.48	949.1405	1232.41	908.3897	1108	1384
1361	358.2	60	1602.61	1378.6	666.0578	1730.009	2186.675	1307.662	1313.059	1322	1325
1362	358.23	91	2678.08	2940.123	714.42	3011.655	7848.347	2592.989	2703.974	3891	4458
1363	358.25	86	4826.5	2948.218	2921.969	5295.483	7867.308	2936.352	3787.56	3328	2173

1364	358.26	69	2913.74	1792.187	2060.702	3334.518	4668.681	5880.112	2521.566	3169	3026
1365	358.26	110	790.328	854.0229	1137.5	1093.68	908.656	884.48	1086.44	1087	986.2
1366	358.29	1046	1563.22	1680.396	1741.551	1508.77	1578.302	1800.185	1881.74	1676	2287
1367	358.31	291	655.453	610.926	2756.704	1919.745	744.3223	632.6734	1402.933	1261	979.6
1368	358.36	144	1954.21	2274.951	844.7143	2524.039	1619.934	1932.753	1644.22	894.5	1647
1369	358.37	200	42474.6	39374.76	32068.71	43191.71	34646.61	42341.81	48826.67	56839	63267
1370	358.84	78	8122.97	1849.976	6785.259	2349.72	1731.05	2350.338	4222.845	7951	3198
1371	358.84	62	3400.71	1602.938	1886.973	995.9511	598.0487	1593.9	1727.314	960.3	2099
1372	359.09	127	243.889	297.18	473.877	828.313	846.1371	683.4971	274.68	308.6	491
1373	359.21	81	3655.44	5308.229	3846.389	5168.887	3002.511	4446.404	2030.565	3330	999.9
1374	359.31	263	2998.77	3046.671	2488.109	3120.935	3045.719	2764.263	3180.785	3580	4193
1375	359.32	140	640.427	1421.6	946.4457	1317.829	1045.288	1253.275	872.612	1130	1074
1376	359.37	200	10713.2	10213.09	8495.217	11274.44	9733.992	10794.45	12723.96	13661	16547
1377	359.83	71	2266.34	1472.008	1757.345	2026.451	1085.38	2712.87	1920.45	2030	2209
1378	360.15	109	3082.85	2118.116	3313.349	1446.48	1256.567	1549.102	2480.877	1881	2001
1379	360.24	63	10826.7	7560.968	9936.439	10408.15	11809.51	14419.25	9771.178	11432	12087
1380	360.27	1048	2151.76	1939.566	2265.6	2127.083	1539.012	2209.066	3069.541	3162	3097
1381	360.32	264	886.135	973.8828	854.2384	1053.756	940.9956	878.123	946.1705	1040	1343
1382	360.32	468	5574.39	3227.914	51507.98	34816.87	19696.08	10966.33	11661.93	24135	22805
1383	360.35	118	1911.94	3004.528	6368.738	3826.162	6010.271	3434.021	4824.222	4943	6592
1384	360.36	181	93130.2	32599.55	84028.68	13528.19	17783.58	24137.25	34138.09	52319	48905
1385	360.37	203	3094.14	2065.678	6610.03	1436.01	714.3139	1884.657	2509.701	2537	3270
1386	360.84	78	7754.51	2359.203	4207.53	1351.036	971.52	2645.575	3829.598	4319	1305
1387	360.84	63	2182.4	579.04	1463.35	548.25	318.99	1010.54	925.148	1246	942.3
1388	361.06	241	4992.35	785.1472	3091.302	1283.087	1417.732	1638.031	1955.536	2994	1989
1389	361.06	221	2324.47	346.183	931.4893	440.5713	473.8816	610.9732	476.5159	813.7	456.1
1390	361.06	259	5085.58	1321.809	4742.259	1467.268	2562.976	1582.13	1878.72	2220	6883
1391	361.06	286	2052.79	1422.263	1669.178	671.7857	755.5608	760.678	733.6105	1163	1415
1392	361.06	306	570.678	254.5738	412.4838	228.6509	124.215	261.1671	247.3313	338.3	376.9
1393	361.24	65	2942.5	3055.058	2572.82	3332.111	4159.443	3603.024	3120.357	3271	1693
1394	361.33	468	2183.51	984.9103	12186.85	11101.93	5761.541	3168.119	5179.28	7139	6600
1395	361.34	90	4495.39	844.592	576.81	1898.208	4014.409	870.7273	2380.557	2672	3106
1396	361.37	181	24726.1	9001.638	23106.81	3357.879	5259.006	6747.484	9806.173	13454	13244
1397	361.83	71	2763.45	998.998	2896.094	2047.209	1046.145	1638.681	1314.471	1585	2692
1398	362.02	87	1812.23	1974.093	3302.595	1014.561	3022.49	1420.41	3060.54	3860	2568
1399	362.24	64	1243.87	1069.9	1281.888	1461.717	1793.947	1365.3	1162.786	1631	1253
1400	362.3	207	4632.94	942.0165	5904.981	3289.643	8277.567	2544.674	3452.973	4327	2472
1401	362.33	86	675017	118830.7	101747.2	314736.8	894002	74965.19	289498.7	3E+05	3E+05
1402	362.37	182	3263.59	1309.516	3279.933	620.5536	886.425	947.0619	1411.102	1862	1877
1403	362.93	85	142642	175647.1	173774.3	87427.48	182603.8	104259.9	129600.9	1E+05	1E+05
1404	363.07	97	2572.67	3514.383	3367.791	3560.554	3681.825	4324.797	1168.935	2172	4642
1405	363.16	65	2034.09	1818.33	1550.208	2243.181	1204.908	1217.481	1504.285	1724	1556
1406	363.3	208	1389.33	567.1529	1903.616	1197.646	2462.052	750.4286	1139.925	1677	774
1407	363.31	1049	1549.4	1598.011	1549.575	1309.922	1475.69	1612.469	1346.629	1597	1955
1408	363.31	146	4229.92	3843.956	4838.343	4703.665	5210.334	4051.865	3607.301	4073	4723
1409	363.33	86	153482	34039.71	24301.7	78868.78	214293.1	20060.05	77102.21	69444	79162
1410	363.93	85	11263.1	15709	12341.33	4618.92	13238.35	8798.951	6522.411	11361	13170
1411	364.06	581	3714.06	947.4237	3734.144	612.1555	630.5902	910.6653	1376.207	796	1618
1412	364.31	144	3302.14	1133.718	4297.249	1418.264	1931.165	3294.734	906.525	3396	2117
1413	364.33	86	19238.9	6127.38	5863.172	16834.63	36585.83	4272.763	11600.25	12301	17753
1414	364.34	148	3238.8	2742.918	3523.022	2995.354	3040.518	2988.328	3643.104	3716	2790
1415	364.34	1046	1389.41	1392.461	1316.7	1312.605	1216.782	1485.848	1371.161	1529	1968
1416	364.35	189	1307.99	953.6097	1345.007	1624.845	2240.066	1490.683	958.1793	1294	2117
1417	364.93	85	4800.65	5031.968	4196.197	2817.36	5373.686	4057.738	6082.285	5946	6059
1418	365.1	82	10199.8	14544	9845.282	21635.09	15624.17	10143.76	9299.343	17655	15175
1419	365.13	98	1208.4	1773.193	1455.82	2400.723	1422.874	2587.884	2988.987	1129	1854
1420	365.13	1069	3600.89	3907.582	3142.8	2794.911	2787.71	3809.912	3461.898	2960	3995
1421	365.16	62	3763.55	3003.932	5672.974	1331.738	2380.703	5810.145	2925.776	3590	3778
1422	365.23	67	7465.92	3502.144	5863.431	2583.773	3065.067	1058.819	958.6593	4410	795.9
1423	365.28	96	5507.84	1702.698	1214.656	961.38	5872.663	2836.638	3877.974	3689	1512
1424	365.31	136	6298.22	3015.487	5625.408	2399.228	1110.611	1923.988	2996.218	3475	2441
1425	366.2	63	2239.26	1442.318	1507.02	1542.919	749.923	900.135	1420.916	1335	1184
1426	366.28	102	2096.27	1105.49	3000.556	1208.862	1683.321	1913.269	1566.949	1807	1570
1427	366.32	126	3121.57	923.8378	2212.497	2174.24	2053.59	3025.949	2007.18	2849	2492
1428	366.37	1170	6418.45	2699.293	28401.48	13018.76	7150.025	5951.34	11110.36	14339	13917

1429	366.91	62	2832.14	2434.343	734.4857	1553.259	351.6564	1655.076	1015.59	1197	2387
1430	366.91	81	6492.48	4017.603	8078.659	2823.094	8875.832	2673.639	7197.734	5173	5097
1431	367.16	61	1212.78	639.012	791.5787	1059.391	835.9305	1191.178	965.7014	879.5	1030
1432	367.23	1183	1276.02	720.3478	1447.938	1107.58	1386.865	1362.756	1011.432	1358	1208
1433	367.37	209	2886.66	1323.713	3984.118	367.8394	482.6375	575.1577	844.3607	1102	1258
1434	367.37	1170	1940.16	773.2758	7735.919	4128.351	1149.45	1807.406	2725.524	3846	3984
1435	367.99	88	4027.56	2152.552	2014.436	2035.919	2964.16	1618.922	1775.541	2920	2262
1436	368.31	104	14801.4	14382.98	13473.33	9641.52	16407.81	10892.27	7559.178	17248	18113
1437	368.31	196	737.582	544.0729	1031.072	1501.188	973.3391	549.8593	660.7023	885.8	1137
1438	368.32	209	1386.02	433.1493	1559.433	833.6873	2242.695	495.006	900.8652	889.7	681.3
1439	368.39	1077	1802.22	2060.889	1925.519	2030.287	2086.064	1168.743	1988.511	1913	2477
1440	368.42	473	2680.81	2313.269	2873.136	3489.01	2404.417	3142.769	1947.531	2587	3462
1441	368.42	2	558.895	418.9846	373.722	310.689	319.3337	361.8586	390.285	462.7	209.9
1442	368.42	115	1834.8	2239.989	2331.065	1546.164	826.25	2246.426	2675.893	2326	2185
1443	368.83	63	1114.71	795.3714	1167.801	464.5667	382.249	431.5917	520.7167	686.5	427.8
1444	368.87	80	2536.48	8982.545	3977.3	6413.052	8513.131	1241.372	1733.218	14908	2068
1445	369.01	307	1871.54	639.8723	1300.2	500.4159	411.6544	539.2971	869.3751	775.2	833.8
1446	369.11	83	3914.77	934.2654	5322.681	3836.9	2307.588	3528.621	3306.064	3660	3509
1447	369.13	106	1178.75	1026.293	661.1508	849.24	634.248	1049.644	962.8575	796.6	1454
1448	369.32	104	3748.23	3002.54	4550.363	3544.38	4156.425	3301.972	3846.301	5028	4254
1449	369.35	701	1143.1	5426.572	107.5917	1554.055	2434.99	1546.652	96.27426	94.88	6192
1450	369.35	667	802.125	1938.466	87.09167	1269.244	1392.376	1172.974	82.10171	111.8	2086
1451	370.05	470	1393.16	761.829	2000.427	572.957	1204.271	958.8513	936.4961	1440	978.2
1452	370.05	356	7158.77	1702.627	2329.319	797.1136	1592.384	2466.085	3418.715	2735	2951
1453	370.29	82	3907.86	1704.257	1282.827	2561.58	2307.153	2203.609	2514.948	3646	1737
1454	370.29	128	2099.07	2455.418	4236.986	3551.387	3983.258	4292.333	3521.798	662.8	1701
1455	370.33	109	2160.58	1243.697	6493.299	4802.727	3519	4129.701	3658.392	4587	2803
1456	370.35	661	339.426	781.9792	143.4375	466.0679	388.853	410.0409	137.5608	141.3	755.9
1457	370.35	688	519.369	1388.977	128.838	861.9692	613.9819	659.0838	123.0854	129.1	1167
1458	370.4	196	1517.26	1799.598	1574.064	1956.328	1695.453	1335.684	1304.3	1559	1631
1459	370.74	71	14999.3	9711.178	8477.44	8007.525	6266	11974.3	11742.96	11376	7624
1460	370.87	81	7353	4017.974	3593.227	3338.109	2175.344	3386.997	5183.03	5794	4326
1461	370.87	61	1877.39	1424.446	880.4357	811.6654	473.5093	915.975	782.5257	821.7	1384
1462	371.1	1068	3334.46	1564.974	1164.727	1013.451	1262.255	1307.667	1260.331	1135	1204
1463	371.1	185	2964.63	1958.241	1559.172	1207.128	2300.056	2498.174	883.6238	1330	2178
1464	371.16	93	2371.07	1049.679	1592.608	2725	4220.17	4586.32	1366.299	1600	815.6
1465	371.23	62	2840.06	2842.523	2841.751	906.1634	2456.702	4184.006	2152.85	2796	2764
1466	371.31	469	1819.82	1739.519	2594.028	2932.983	2505.252	2203.802	424.2217	2795	6727
1467	372.06	1	0	0	0	725.1922	0	863.4825	378.6475	539.9	0
1468	372.06	1042	4122.79	4510.068	3234.481	2047.131	4618.167	4074.583	4444.283	4659	5243
1469	372.07	130	1584.88	961.2162	939.7244	5122.233	5201.735	1486.578	4645.059	5287	4832
1470	372.13	121	4281.69	6466.236	1264.356	4692.2	5389.718	4333.596	4108.043	4703	4356
1471	372.13	1	1162.8	952.028	262.0382	725.1922	1710.107	863.4825	894.241	899.5	898.5
1472	372.14	472	5624.76	5034.517	14449.97	7975.609	1104.571	6448.542	742.0336	7663	2919
1473	372.26	67	3665.22	2691.114	5001.604	2153.953	5547.446	7188.192	3177.422	2693	3522
1474	372.31	2	1317.1	787.238	956.3408	591.2617	2779.659	1055.509	1222.655	912.4	919
1475	372.31	1175	4058.84	2449.58	2367.073	1836.55	2116.203	3300.817	1740.439	3816	3105
1476	372.31	133	3112.92	4403.648	3295.087	2255.65	3164.507	939.3773	1232.28	2408	2711
1477	372.31	1197	2425.81	3303.511	2487.627	1957.736	3790.048	2591.257	2283.145	2678	2179
1478	372.31	47	5868.9	8629.994	2551.325	11683	6640.613	6476.907	2372.854	1983	8453
1479	372.31	1099	2785.76	2104.131	2389.929	2283.272	1935.171	2574.569	2212.889	2483	2506
1480	372.31	1053	3954.55	5187.021	4405.231	1939.103	9704.775	7774.94	10887	4264	7731
1481	372.31	1076	3279.23	2787.519	3099.731	2621.754	3086.152	3187.976	3205.896	2910	3372
1482	372.31	102	4369.69	3402.036	1959.709	4132.715	5975.162	2799.146	2830.101	2357	2672
1483	372.35	232	3597.47	1793.899	3988.328	2164.334	2305.812	1801.764	3549.518	5130	4484
1484	372.74	70	25196.2	19921.53	16579.17	15060.33	11158.22	21650.38	19594.26	24058	23863
1485	373.13	470	1617.5	1304.434	2851.337	1029.668	2322.188	1817.93	2424.796	1649	2001
1486	373.22	71	2036.47	1401.691	1573.748	1022.238	1485.437	1108.701	1392.84	974.8	1704
1487	373.24	99	862.912	725.824	903.6232	1664.46	1247.5	1034.917	987.1974	1159	883.3
1488	373.31	49	1199.3	1712.487	438.1333	2963.033	2020.587	1688.1	422.8933	410.9	2021
1489	373.95	83	7013.81	3983	8868.606	2566.908	1279.668	1748.62	3657.442	3706	3235
1490	374.04	383	2876.56	2066.554	2992.068	1795.665	1336.379	8653.397	1536.31	2116	2890
1491	374.04	469	520.907	326.5053	1572.467	563.6746	801.152	750.4792	567.89	818.9	1061
1492	374.06	129	2354.55	2528.849	1936.508	2962.218	2161.44	2620.793	633.3567	3198	1435
1493	374.06	2	567.21	253.9665	394.3903	243.0955	231.3512	311.0523	302.0522	264.9	291.2

1494	374.06	1042	4322.17	4576.729	3448.038	1934.147	3688.897	4504.225	4621.203	4286	5322
1495	374.29	1048	2044.99	2306.325	2133.535	652.8615	1866.343	2337.975	1812.441	1840	2391
1496	374.3	117	1062.03	876.8467	1937.591	2328.281	821.6947	2939.899	2360.199	2176	1806
1497	374.3	148	12129.5	9605.419	19648.55	13337.21	13413.95	25765.73	11223.17	15057	19635
1498	374.36	134	43007.7	32732.98	41701.06	38950.76	48585.51	28263.9	38768	52763	47037
1499	374.73	71	22081.6	14310.63	12847.55	12289.61	9213.682	16535.6	16251.3	19412	17327
1500	374.82	77	750.652	1295.861	1940.739	777.084	202.884	559.8516	2283.633	1145	57.71
1501	375.17	111	3263	6312.001	2818.752	3731.264	4607.164	1702.32	1476.762	1205	944.1
1502	375.21	68	1683.46	1446.269	3204.469	996.903	2824.065	1886.192	1348.822	2819	1732
1503	375.3	148	2967.29	2401.036	5322.175	3993.982	3479.239	6795.896	2206.765	4276	5387
1504	375.32	119	2274.92	2118.735	3834.942	1330.586	5260.093	2771.646	2018.979	3663	4101
1505	375.32	1084	879.951	1630.726	1418.916	1498.467	2093.12	1540.737	1359.103	1413	1953
1506	375.36	134	10948	8760.599	10721.97	11468.49	13576.68	7638.025	10556.56	14459	12516
1507	376.06	1042	1174.5	1477.139	1232.781	918	1407.48	1488.233	1259.664	1188	1689
1508	376.17	112	635.322	1546.344	1497.5	633.0192	1178.614	405.398	331.9008	667.4	667.2
1509	376.26	79	98782.1	50762.36	99814.76	80637.33	77849.82	133515.7	107505.7	1E+05	79257
1510	376.29	470	885.636	465.5231	3161.718	2022.545	1438.107	1120.311	1197.911	1972	1630
1511	376.32	140	17287.6	7922.786	34516.95	28650.73	41010.17	37453.48	17142.4	26602	30912
1512	376.32	172	1801.72	844.6946	3726.76	2777.954	2480.927	2129.508	2080.64	2447	2708
1513	376.32	193	3439.47	1737.992	5338.343	4957.985	7356.808	2747.389	3266.709	4291	3905
1514	376.34	264	761.247	805.4582	664.104	823.7661	747.9846	736.3435	855.9828	866.6	1202
1515	376.34	94	3036.17	1816.538	1604.515	2245.253	2604.125	827.9213	2471.466	2130	1318
1516	376.37	135	2214.14	1445.614	1917.216	789.804	1806.261	1422.435	2416.141	3380	2460
1517	376.73	70	10285.3	6113.667	6677.042	5877.02	4093.937	8353.612	7485.269	8640	9275
1518	376.94	85	33646.6	42059.57	42755.13	24467.35	42439.55	19407.41	27740.52	30119	34928
1519	377.21	64	3430.63	1576.939	2145.325	965.6687	2282.897	3958.927	2083.934	2322	1550
1520	377.26	79	27188.7	15528.63	27929.39	16684.3	19460.89	36969.02	31304.67	29098	23638
1521	377.32	139	6796.04	3805.806	8912.597	8780.711	10964.98	9530.316	7999.701	8966	11513
1522	377.32	193	1365.21	885.548	1690.141	3675.369	2346.176	1237.098	1362.214	1571	1574
1523	377.32	169	3889.95	2726.46	3976.182	3566.152	4225.555	3414.899	2196.783	3841	4103
1524	377.94	85	978.399	3457.88	3850.362	2297.889	2090.932	3625.308	2298.14	2248	4041
1525	378.13	265	1505.79	708.1485	2710.362	644.5721	633.4836	746.7754	722.475	876.9	1014
1526	378.27	79	4864.01	3244.13	3503.803	3915.718	8917.76	7161.973	4867.84	5772	4026
1527	378.28	1076	1007.62	1037.138	1071.787	1025.769	888.632	1172.511	999.7224	1039	1106
1528	378.28	1098	1538.53	1377.03	1417.353	1404.858	1347.88	1830.941	1047.077	1659	1566
1529	378.32	93	17613.2	21611.94	13049.77	17640.02	15189.09	3362.089	7661.22	7911	9507
1530	378.32	72	9984.38	4599.724	2056.765	6397.753	9320.24	6210.06	4505.11	4722	5595
1531	378.32	140	2643.86	1978.76	3582.338	2441.342	2385.078	1995.012	1470.52	3025	2444
1532	378.33	169	633.428	890.2417	1168.434	1091.019	1778.301	943.2462	706.752	1465	1593
1533	378.36	1137	1690.92	1465.5	1783.98	1573.47	1546.232	1539.044	1322.719	1821	1085
1534	378.36	169	4865.15	2676.624	3313.514	2051.274	2985.92	3926.103	2209.099	5024	3968
1535	378.73	70	1707.88	1504.061	1428.62	848.3969	968.5931	2231.658	1256.063	2382	2653
1536	378.9	83	43579.6	37719.6	61099.64	26514.34	35388.28	31125.83	37764.34	35662	38913
1537	378.94	66	3413.32	2567.153	1658.563	1326.211	1119.954	1128.425	650.076	1180	2795
1538	379.09	64	7421.5	984.9982	1138.908	2254.322	7872.569	934.0055	2686.542	3341	3488
1539	379.28	133	1855.06	1086.644	1706.855	1470.277	1580.35	1698.038	770.76	1494	1915
1540	379.3	112	3693.38	2412.976	3604.587	3093.829	5201.038	2314.907	2343.636	2774	2686
1541	379.32	94	4220.66	4358.346	3243.124	4077.661	4741.403	877.2314	2282.43	1603	1915
1542	379.9	84	3790.5	3636.658	6529.184	3069.656	1374.327	2104.124	4340.545	2725	1726
1543	380	89	3206.66	1966.037	7848.696	1778.448	4013.888	2023.885	5174.201	3184	3046
1544	380.33	1047	1788.65	1471.32	1658.672	1348.616	2152.819	1990.689	1693.654	1915	2010
1545	380.34	113	6127.13	3001.056	1032.5	1100.358	4820.163	5126.549	3638.823	4522	5488
1546	380.34	144	976.349	4445.534	5702.801	2845.07	5151.613	4365.176	2672.468	5317	4412
1547	380.34	1104	1592.47	1732.151	2516.655	1633.667	1655.881	1796.07	1738.507	1464	1866
1548	380.34	1073	1548.4	1344.368	1444.18	1458.834	1187.967	1670.733	1589.179	1661	1683
1549	380.35	201	913.246	915.24	1392.468	1219.68	1347.648	1757.792	1221.157	1129	1764
1550	380.77	64	4323.07	948.328	2886.432	1114.211	1315.901	2324.923	2735.408	7524	3356
1551	380.9	83	5040.54	3908.693	7780.914	2821.832	4013.609	4495.8	3817.465	4602	4200
1552	380.94	67	558.145	701.2333	734.0677	613.7105	739.3061	479.3208	1039.004	1001	1309
1553	380.99	89	13796.7	606.3216	1303.731	2041.885	16678.57	377.1315	4945.25	5749	6940
1554	381.08	65	3441.63	1422.288	630.8003	2045.918	3906.389	1235.136	1791.863	1228	1503
1555	381.3	264	1359.84	2031.751	1265.396	2189.06	1670.085	1771.109	1532.176	1760	2385
1556	381.34	112	1397.41	1285.593	938.497	3032.961	2018.936	1751.666	1117.992	1305	1827
1557	381.34	144	1785.93	1275	1663.875	1714.732	1747.87	1676.648	988.52	1428	1477
1558	381.38	291	1067.74	701.7932	8778.699	3516.325	1081.396	1156.235	3958.407	3602	2218

1559	382.33	113	1088.95	944.5648	1032.75	492.18	1092.398	823.4455	1392.135	837.4	1356
1560	382.39	291	423.253	272.4765	2577.568	1828.518	614.0535	522.71	1173.983	990.9	774
1561	382.77	67	11496.1	4902.569	6469.028	1943.945	1973.085	2944.6	8863.442	7709	3151
1562	382.89	80	2013.05	1526.306	3789.831	1029.461	852.7871	1217.93	1629.546	2121	1254
1563	383.04	284	2533.68	1427.363	1830.202	783.2543	658.8663	798.4136	713.05	1500	1996
1564	383.04	257	5279.84	1791.024	2981.735	1826.946	2421.571	3994.477	1935.113	7222	3643
1565	383.04	305	692.733	843.0728	705.7823	325.0144	255.4265	356.0676	256.8574	488.7	738.9
1566	383.2	101	741.86	807.28	1315.476	1466.489	948.7833	1133.138	1002.893	950.3	1203
1567	383.4	467	20343.4	7902.071	104427.7	56365.96	41667.33	26277.94	8474.022	54505	55325
1568	384.29	106	3249.74	864.933	1404.967	3481.38	4839.282	1056.327	2064.6	1871	1009
1569	384.31	86	290695	56785.03	49094.51	132505	278202.5	46272.44	138488.9	1E+05	1E+05
1570	384.34	141	3042.39	2879.544	3916.383	2637.884	2621.299	2203.927	2519.918	908.1	2916
1571	384.34	52	1675.85	3273.823	1457.118	3083.476	1148.882	2028.061	1570.204	1389	1981
1572	384.35	122	4282.82	4049.684	5455.208	4062.457	4510.613	5146.31	1856.393	2272	4090
1573	384.35	1074	1314.73	2704.49	2945.222	2636.856	2978.758	3775.41	3657.761	3267	2939
1574	384.35	1050	4565.86	3563.114	4159.475	2921.681	10698.78	4598.152	4743.434	4326	4611
1575	384.35	1118	1919.32	1945.101	1454.056	1821.622	1676.603	1144.855	1813.083	2134	1975
1576	384.35	1091	3534.81	3755.43	3128.747	3349.432	3364.284	3485.539	3735.521	3449	3742
1577	384.35	1143	1659.88	1654.881	1246.623	1570.352	2278.441	1763.157	1577.25	647	2762
1578	384.35	1198	2551.06	4652.495	1976.456	2892.933	5405.918	2721.033	0	6965	3946
1579	384.35	1173	4985.48	4174.018	2891.089	2806.12	2902.821	3606.84	1511.07	3915	3420
1580	384.35	212	2258.7	1398.461	2031.674	1600.64	2285.389	2008.385	1738.506	1982	1738
1581	384.4	469	6215.44	1710.483	30702.97	19088.01	12409.16	7758.879	9453.966	16951	15281
1582	384.76	64	6222.18	1595.603	4944.265	1134.926	2081.131	2988.763	2579.35	6380	2936
1583	384.84	79	3098.15	2062.711	3584.42	2671.788	1549.716	2482.744	4860.76	2821	2877
1584	385.18	98	1496.8	1185.823	1256.455	2085.791	2311.292	1525.304	1053.444	769.9	2535
1585	385.18	71	1300.13	2531.348	1021.911	756.6565	1189.5	914.0004	620.94	1083	789.6
1586	385.29	147	1367.32	1297.95	1054.988	1267.494	1504.375	1377.485	1418.208	990.9	772.6
1587	385.31	86	69894.4	17549.11	12697.88	37433.75	68057.9	13185.61	37097.88	1286	37918
1588	385.34	113	1273.18	672.21	1596.3	1902.927	1068.099	999.8932	1637.493	1321	2286
1589	385.35	1198	627.002	1391.553	540.1	728.3222	0	854.32	0	711.4	893.3
1590	385.4	469	1241.47	500.8608	6299.765	3423.878	1027.337	1427.667	1031.356	2965	2625
1591	386.15	465	4909.06	4517.938	8659.533	7228.016	6774.947	6029.175	4856.383	6566	6638
1592	386.15	2	0	1665.839	392.214	1136.205	1094.256	1363.094	1343.31	1580	1629
1593	386.15	42	8298.93	5909.843	5804.948	10166.36	9794.487	9592.361	8256.988	10355	10229
1594	386.31	86	13837.7	3425.108	2566.23	6523.086	16525.43	1819.681	10651.01	9144	6283
1595	386.33	208	2658.2	1133.272	4669.808	1639.548	5264.562	928.5785	1988.754	2362	2679
1596	386.4	136	384.053	616.616	560.838	819.168	575.8425	523.4775	460.46	702.9	493.9
1597	386.71	70	12562.4	5832.539	7682.755	4697.768	3506.676	9005.868	6137.088	8565	9043
1598	386.84	63	1238.74	414.276	802.4571	369.2284	884.182	799.632	1250.336	2401	645.8
1599	387.15	469	2143.3	1936.867	1148.591	1482.938	1782.313	2533.674	2579.658	2851	4112
1600	387.18	1039	1615.11	2023.376	1667.157	979.9586	2074.287	1822.612	1820.91	1764	2308
1601	387.19	78	99907.3	10997.81	13024.61	33192.2	89485.23	24371.48	35774.46	37253	39198
1602	387.31	87	2491.49	502.74	256.0281	1276.096	2050.251	723.1344	1783.408	1008	1502
1603	388.2	79	23958.6	3877.026	3606.2	10116.58	21040	6117.705	10373.81	9715	10733
1604	388.21	1068	7972.18	7898.317	7345.062	5995.938	9466.177	8499.585	11916.09	7728	12642
1605	388.25	61	14055	10583.04	12442.57	5987.133	9477.506	17191.25	9226.53	8862	10745
1606	388.34	149	2694.99	2559.585	2217.2	1360.4	1980.468	2033.123	2601.586	4744	2716
1607	388.34	89	15235.4	4630.185	5668.215	9501.207	17445.68	2269.391	2012.606	8078	7865
1608	388.36	1067	2765.18	1292.851	2614.355	1516.327	2361.728	2355.578	2041.38	1909	2518
1609	388.71	71	19460.6	12146.67	14345.66	9425.626	6336.548	16374.15	14026.62	16137	17168
1610	389.12	63	3028.75	1262.418	771.606	1754.178	3314.156	800.685	913.9128	1327	1644
1611	389.2	78	4770.15	429.5175	1234.196	3177.661	3260.436	3008.618	2008.956	1993	3069
1612	389.21	1068	2136.69	2154.743	2010.528	1749.204	2365.982	2434.352	2968.54	2128	2313
1613	389.26	62	2264.24	1973.805	2360.971	1961.679	3720.96	4499.16	3539.154	2855	2272
1614	389.34	89	3096.87	753.8843	2471.834	1532.244	5689.97	1651.674	3037.364	2474	3936
1615	389.97	103	294.255	96.92375	57.845	1276.38	1080.077	371.48	0	198.3	403.7
1616	390.3	95	1355.49	3110.109	3675.258	2725.147	1728.135	4207.463	6376.623	3223	3078
1617	390.31	118	1600.28	1231.422	1673.756	1279.532	750.4533	1335.6	875.568	1611	1020
1618	390.33	149	3822.67	3289.37	1244.203	3303.009	2305.813	3374.415	2090.424	2873	3236
1619	390.36	103	24823.6	19706.43	22383.57	17715.56	24352.71	14552.73	9277.692	23660	19183
1620	390.71	71	17299.2	9845.929	11622.85	8858.197	5498.648	15669.37	10782.2	14078	14496
1621	390.8	78	6750.11	1615.605	3383.651	1589.033	1655.196	2934.94	6717.44	7578	2452
1622	390.8	63	8966.79	3202.534	4478.11	2003.713	1417.121	3606.658	4649.173	5540	4949
1623	390.96	85	4368.63	7238.7	6871.15	3559.436	4102.358	3819.644	3815.713	4235	4995

1624	391.12	100	1354.18	957.733	5208.851	380.52	1411.25	288.4857	325.8171	440.7	715.7
1625	391.21	72	1713.98	2087.641	1699.655	1316.922	1388.229	1043.04	1648.5	794.4	1165
1626	391.28	391	40826.5	25982.04	54790.9	37326.24	39750.05	47447.02	42548.48	49002	44477
1627	391.34	147	2107.64	31943.03	2221.118	1624.374	44905.33	1499.224	28643.3	36353	33412
1628	391.34	203	2536.38	34700.76	1212.863	2933.353	31434.67	2078.481	1959.352	3096	2778
1629	391.36	104	8543.14	5516.498	4695.329	4448.893	5927.139	4960.787	5116.32	6746	6225
1630	391.95	88	9565.79	1984.594	4059.595	2287.5	3177.949	4074.858	3557.594	5535	5204
1631	392.03	346	2082.66	616.6299	1833.014	893.6776	701.2977	1401.952	953.7848	1625	1205
1632	392.26	91	29349.4	38697.89	1670.845	4180.71	4869.284	5635.522	2538.376	4981	6744
1633	392.29	1052	9738.73	16513.17	7172.49	12454.53	12708.18	11716.39	32080.19	18111	11008
1634	392.29	49	11348.6	16496.92	6952.144	14978.93	11987.14	12408.2	6522.545	6865	12777
1635	392.29	1076	8452.08	8711.706	8288.404	7493.382	7450.698	8921.273	8897.431	7670	8162
1636	392.29	1194	9577.31	9727.85	8627.287	7450.469	10845.54	9640.779	8283.621	9652	7100
1637	392.29	1178	11334.9	9977.293	9400.538	8183.311	10608.73	1775.273	5761.551	10954	8271
1638	392.29	391	11085.9	7423.68	13416.93	10130.86	10130.03	12617.16	10632.48	12915	11576
1639	392.29	140	12505.3	11087.95	25789.16	13219.11	11750.21	13869.96	8260.725	17070	14460
1640	392.32	108	782.496	5490.549	9078.844	5778.36	8316.827	3267.369	4563.74	5609	2888
1641	392.34	81	7187.22	9344.226	6673.77	3584.373	5401.834	8785.179	2687.519	3461	6977
1642	392.37	203	2870.25	2462.718	2057.899	1976.244	1257.408	2188.162	949.666	2026	3075
1643	392.71	70	8701.67	4549.572	5460.661	3172.791	2233.523	8020.69	5197.579	6674	6901
1644	392.79	63	11557.1	3826.45	5235.456	2268.677	3356.6	4858.241	5708.545	6070	6105
1645	392.91	83	7753.94	10879.13	14300.34	3336.728	11408.24	5678.55	9362.306	8895	8546
1646	393.21	62	13038.8	5553.025	9167.217	3761.68	5930.501	11537.01	6429.735	7143	7257
1647	393.25	90	5894.28	5614.683	5042.286	5437.96	2300.018	2290.133	3564.528	1478	2046
1648	393.29	79	135008	70475.61	147220.7	102162.3	117538.4	175364.2	146754.9	2E+05	91258
1649	393.29	392	3906.37	1800.754	2260.994	2230.767	2573.914	2614.917	2578.466	3103	2593
1650	393.29	1072	3641.67	2522.623	2429.85	3195.667	5597.179	7533.395	2909.662	2274	5115
1651	393.29	1197	1978.85	2451.609	2116.992	1690.169	3058.432	0	0	2053	1845
1652	393.29	472	2883.84	2325.256	3416.178	810.5236	1704.185	3303.892	981.3773	4570	3217
1653	393.32	124	3654.65	908.16	4300.42	4124.221	3448.235	4130.215	2007.042	2843	2719
1654	394.21	62	3249.69	2143.797	1953.75	1330.994	1333.465	2454.285	1475.302	1752	1482
1655	394.26	1077	1354.39	1715.22	1425.436	1721.802	2111.627	1860.48	1463.671	1456	1951
1656	394.29	79	37080.5	22808.69	38263.01	26421.47	29854.61	34645.27	39268.68	43823	29130
1657	394.32	108	1594.54	2306.749	4898.124	2939.977	5807.377	3789.845	1811.931	3149	3175
1658	394.35	126	3568.39	2821.754	1370.169	1575.22	5232.542	5728.272	2014.812	3981	1705
1659	394.35	1076	1728.18	1784.611	2015.706	1730.667	1717.888	1747.008	1728.06	1377	2087
1660	394.35	1138	2100.5	1977.965	3055.931	1431.36	1895.853	2640.475	1859.135	2470	2246
1661	394.35	168	4978.85	3991.417	4366.5	3932.611	5062.72	4970.126	5231.378	9029	4849
1662	394.38	291	463.583	557.2014	2194.675	1416.274	750.9228	508.5052	1313.12	1196	619.7
1663	394.79	69	2257.37	1593.266	1702.587	1299.799	2029.3	987.017	829.23	2153	3236
1664	394.88	84	6278.59	5813.589	9245.72	2546.366	2041.729	6109.639	2264.114	4413	5842
1665	395.29	79	5623.04	4508.635	6035.956	4380.513	5035.995	8675.345	7252.733	7349	4087
1666	395.33	104	1581.21	1855.572	1994.287	923.58	2144.492	1181.785	805.7769	1461	990.5
1667	395.35	125	1296.79	542.7644	957.5253	964.2413	1331.837	1139.155	1064.7	894.4	798.7
1668	395.36	168	1416.11	1231.791	1290.677	1242.052	1409.137	1245.793	871.5333	1484	1460
1669	396.33	112	8430.55	6351.162	11189.14	7389.297	12901.34	10550.43	4426.704	8188	8621
1670	396.34	134	2204.03	1729.442	2125.518	2567.946	1544.048	2655.667	2020.849	1604	3404
1671	396.39	469	3935.09	1622.718	3912.992	8044.897	5749.298	7676.612	4405.756	11179	10091
1672	396.76	64	2937.7	668.7643	1551.849	590.2594	326.0176	727.425	1311.423	864.4	844.7
1673	396.87	84	1238.93	0	2234.749	0	511.56	921.4725	1747.883	1246	256.8
1674	396.92	62	1358.25	1033.193	868.224	829.5696	391.3	533.232	827.86	640	744
1675	397.02	89	3302.17	0	1707.414	473.2763	1024.38	0	255.5516	500	255.8
1676	397.2	140	2833.67	3202.926	3260.084	3826.064	5036.625	2420.746	2906.073	3942	3039
1677	397.2	79	22154.9	18698.83	21146.08	22491.42	22014.6	18579.41	18672.4	16424	16772
1678	397.26	75	5334.15	3355.412	1343.767	2692.312	3980.691	990.0916	1588.153	417.7	1079
1679	397.29	125	1531.25	909.2386	2711.508	2149.467	2224.701	2629.855	2924.754	1604	1671
1680	397.33	112	3468.66	2287.296	3449.542	1705.364	3471.619	2243.608	2291.207	2779	1971
1681	397.38	148	7388.54	3907.583	12367.81	8561.006	6033.732	14592.18	8091.466	8733	10346
1682	397.4	470	2027.55	1113.829	4094.371	1191.45	3171.123	2127.106	1165.581	3632	3708
1683	398.2	139	1858.15	1527.374	1831.856	1068.48	2352.2	1298.574	1886.667	1402	1189
1684	398.2	78	6400.41	5240.552	76450.09	5279.809	4072.67	4734.27	84617.88	2518	3282
1685	398.23	1104	3104.24	2744.233	3322.853	2568.175	2913.826	3246.94	3349.271	3284	2938
1686	398.23	1049	2173.13	3766.488	2181.122	2268.125	2657.564	2823.905	4044.943	2613	4679
1687	398.23	1076	2306.59	1236.006	2556.843	2845.642	2016.615	2690.827	2421.252	2298	2695
1688	398.24	80	91469.1	43855.68	78140.37	49868.78	61710	75060.14	85158.03	86774	50388

1689	398.36	119	1305.01	1876.005	1717.765	1367.761	1765.186	1760.573	1068.679	1904	1907
1690	398.38	148	2121.1	1334.302	3596.923	2647.965	2229.424	3881.494	2372.199	2807	3342
1691	398.74	70	5144.52	978.2725	4407.594	958.6415	1225.094	3647.669	2045.364	6516	2342
1692	399.14	639	2090.91	834.0157	1964.052	1137.199	1047.127	1112.927	1640.089	1287	1165
1693	399.14	616	1642.69	642.0438	718.1211	919.7257	2215.502	843.5028	4279.133	982.4	840.9
1694	399.14	678	1329.66	608.2863	808.3897	776.08	722.2623	844.2887	986.4983	867.2	787.2
1695	399.14	570	1414.24	556.332	828.223	738.7263	547.5565	796.8948	1116.376	904.5	829.6
1696	399.14	654	1110.6	317.7	976.5099	646.2468	559.1041	503.4918	908.6171	749.9	696.9
1697	399.14	600	1552.51	557.634	1160.096	793.9563	738.3767	808.6187	872.352	924.6	917
1698	399.15	64	659.076	1896.806	1610.793	945.2197	1817.585	1747.476	1422.528	1532	1523
1699	399.24	80	25533	13883.15	21880.85	2335.622	16963.41	19689.5	22625.26	25007	14521
1700	399.31	135	3534.17	1998.787	3922.789	2317.676	2095.562	3219.385	3062.194	3223	2163
1701	399.36	118	898.207	503.44	382.3856	615.1728	793.6527	278.2544	744.48	74.82	750.1
1702	399.39	140	7559.59	4539.371	14532.06	15990.4	21974.51	18025.07	10143.84	12126	16019
1703	399.39	193	3061.67	1171.177	4699.255	4210.691	3797.148	3138.531	2765.21	3983	4159
1704	400.16	468	2138.01	1812.222	4122.834	1771.545	2697.747	2335.457	2221.977	948.8	2494
1705	400.16	2	394.151	557.2357	493.608	368.3371	489.2094	455.3031	519.8655	721.2	484.1
1706	400.24	79	6600.54	2875.577	4276.895	4000.008	4166.682	3689.838	4350.258	7236	2872
1707	400.29	85	27886.1	9003.181	7699.556	8317.899	16216.33	3408.598	15681.78	17854	14053
1708	400.3	1074	1429.69	1448.418	1131.745	1426.891	1331.844	1703.508	1923.515	1261	1695
1709	400.38	141	16348	17517.94	12832.01	18051.56	20610.37	12172.47	12952.26	14666	17236
1710	400.4	193	910.598	519.9055	1463.471	1496.298	1200.638	990.0133	957.7353	1096	1257
1711	400.83	63	12165.6	5079.263	4798.267	3219.353	1761.275	753.675	6023.653	2812	7822
1712	400.83	77	14620.5	5023.685	7565.26	3676.092	5083.515	5974.044	11062.14	20229	5019
1713	401.28	85	7556.62	1752.243	1284.427	4080.708	8049.942	2147.21	3603.459	11940	3057
1714	401.28	111	3565.42	2150.387	3720.454	2347.309	3574.188	4774.219	2997.713	3722	3354
1715	401.34	412	2867.57	1491.394	2978.953	1038.578	1153.051	1604.713	1851.045	1477	2078
1716	401.38	141	4837.47	5430.716	4700.074	5748.137	6133.949	3698.237	3717.011	4366	6028
1717	401.82	69	4880.28	2832.952	3392.197	3718.795	2114.115	5658.941	3302.019	2925	3673
1718	402.23	61	1263.02	1291.4	1720.873	1142.962	1717.839	1706.022	1566.591	1463	1436
1719	402.29	113	1484.19	1125.468	1513.11	1684.307	1200.214	1582.2	692.655	1134	1066
1720	402.34	411	840.253	408.6219	1047.595	305.9974	366.2195	476.5913	577.715	566.6	522.9
1721	402.38	141	1482.67	1060.181	1666.933	1682.267	1579.301	1015.154	1759.685	1314	930.3
1722	402.39	187	10510.6	10251.83	7581.666	9621.978	9538.728	8658.311	10827.17	14423	13261
1723	402.69	71	3655.43	2025.515	2670.405	2582.022	1098.56	3811.023	2249.149	1419	2782
1724	402.82	77	13756.9	5510.075	7915.148	3389.127	3370.24	4963.455	10017.56	12485	4047
1725	402.82	63	1916.25	5105.273	5775.103	3004.82	4488.84	5715.502	5928.461	6674	7370
1726	403.19	67	1434.31	1980.587	1148.579	931.9628	680.3922	891.7388	1230.677	1536	1312
1727	403.23	1082	3223.01	3281.702	2920.8	2365.72	3318.945	4034.625	3358.801	2614	2817
1728	403.23	1050	2560.36	2217.066	1535.49	1899.701	2528.106	2321.705	2994.47	2423	3232
1729	403.23	116	2198.87	1349.92	1495.109	2378.671	2550.893	1719.24	1985.5	2108	1540
1730	403.35	149	1593.21	1221.317	2109.153	2404.063	3553.619	1253.461	1872.59	2172	1965
1731	403.4	188	3030.29	2809.511	2188.891	2744.271	2941.809	2524.759	3122.301	4187	3512
1732	403.82	70	5856.34	4453.278	3989.215	3807.365	2102.1	6014.547	3853.665	4904	5013
1733	404.22	78	14227.6	1731.896	3236.861	4829.46	14108.2	3303.949	5555.324	7683	3777
1734	404.34	105	2464	2557.796	1330.56	2593.64	2237.064	1615.767	2666.577	2767	3122
1735	404.35	188	1325.73	748.9349	1217.795	1002.376	1475.504	1305.517	1053.911	1312	1225
1736	404.68	71	8442.94	3555.416	3775.553	4233.353	1800.673	6221.946	4864.74	5610	6405
1737	404.82	77	5211.71	2073.089	4077.115	673.7051	1811.461	1789.458	4571.029	6842	2090
1738	404.82	62	4830.45	1938.87	2225.15	1400.383	623.7501	2214.287	2027.254	2660	3226
1739	405.01	915	1609.81	805.9602	2653.49	570.7566	1008.61	1005.493	759.6033	1232	1551
1740	405.01	899	2265.19	891.8184	1551.878	1434.931	1473.541	1520.269	1266.285	1994	1686
1741	405.02	247	8050.29	1353.25	2613.24	1397.576	1567.531	1304.744	1485.769	2152	2951
1742	405.02	275	2170.38	1094.494	1965.141	826.0328	980.5628	1363.837	971.544	1550	1017
1743	405.22	69	2153.16	1808.069	1316.378	2448.93	1390.706	704.79	1612.352	1620	828.9
1744	405.22	1072	1667.66	1276.692	1254.331	1185.882	775.1224	2511.104	1015.163	1236	1715
1745	405.22	1052	1405.92	1501.02	1290.047	695.0163	1278.021	1313.932	1102.474	1534	1512
1746	405.3	107	1540.44	981.684	840.96	1417.177	1090.394	528.75	1691.29	1084	1689
1747	405.81	69	2438.76	2135.869	1838.52	2490.25	1310.746	2621.415	1571.664	2494	2735
1748	405.96	85	5942.91	2472.258	7059.728	2686.432	4550.994	3517.217	4337.652	4846	3163
1749	406.21	109	1021.57	2531.754	1528.126	1263.7	1739.839	1585.388	646.8337	986.9	1515
1750	406.29	197	1156.79	1954.51	1200.088	2071.973	1533.88	1728.198	1028.915	1372	1128
1751	406.29	92	11892.3	7191.8	2598.288	14549.04	3005.035	9558.776	20739.72	707	22433
1752	406.33	477	1460.17	1420.563	3053.052	3067.079	2235.18	1959.68	1958.043	2505	1822
1753	406.33	2	1080.13	547.4986	742.6945	447.2954	543.6894	701.927	437.777	701.5	454.3

1754	406.33	266	1287.26	1599.303	1318.889	1552.075	1234.973	1320.355	1117.722	1366	1325
1755	406.33	1098	3745.44	3716.188	4905.993	4130.261	3157.15	4238.362	3718.056	4117	4700
1756	406.33	1142	972.939	2000.1	1806.7	1865.594	2242.33	1799.893	1515.482	3271	3151
1757	406.33	109	2951.35	4707.225	3935.185	3928.699	5458.127	2422.75	2987.775	1916	2878
1758	406.33	1173	5589.88	5141.88	3763.209	4098.612	2741.965	5387.549	3927.063	5295	4658
1759	406.33	1074	3300.08	2393.224	3008.47	2697	2679.828	3759.318	3382.759	3183	1165
1760	406.33	1054	2440.01	2973.769	2363.86	2405.481	2792.452	2523.15	2599.272	2710	2944
1761	406.33	223	1944	2015.7	2163.715	2093.619	2811.222	1763.187	1628.468	2093	1789
1762	406.33	1198	8747.78	5302.951	1948.1	2804.922	0	2686.36	1314.367	1777	3706
1763	406.33	1121	863.784	1641.695	1604.397	1533.102	1262.831	1925.657	1567.73	2596	2404
1764	406.33	208	2490.37	2137.942	2224.536	2027.019	3190.99	2296.398	2428.986	2359	2726
1765	406.33	135	2994.88	2375.068	3085.194	2701.218	3009.346	2187.918	2714.594	2713	2156
1766	406.35	86	269344	35031.45	25701.16	136620.6	397472.8	18753.71	115484	1E+05	1E+05
1767	406.68	72	6110.54	3306.768	5125.665	3614.8	1285.7	5952.368	4389.923	5012	5735
1768	406.79	63	4379.73	1618.481	1074.496	1167.721	646.8398	829.84	1669.678	738.5	1181
1769	407.19	66	1919.29	925.6949	1716.345	1849.45	1296.309	1608.533	1359.868	641	1507
1770	407.27	1112	3304.39	2015.095	1964.282	1839.987	1900.944	1924.53	1667.952	2298	2382
1771	407.27	140	3297.37	1446.419	3633.42	1512.649	1736.931	2897.326	3467.195	3652	3311
1772	407.3	1076	1448.39	1618.295	1638.35	1673.231	1809.334	1870.243	1562.436	1477	1704
1773	407.33	1198	646.998	1868.996	873.6127	0	0	0	0	809.5	0
1774	407.33	144	4356.59	2382.042	951.9275	2676.791	2898.998	2519.491	5497.929	2443	5288
1775	407.35	86	74851.7	10662.17	6347.973	37658.75	101728.3	6211.705	33945.9	33632	33267
1776	408.27	1113	4111.9	3225.249	4598.282	3888.256	3747.697	4430.427	4017.655	4504	3901
1777	408.31	1149	2057.94	688.7356	1998.75	1868.502	2244.372	2894.918	1687.296	817.9	1943
1778	408.31	195	13153.9	12862.46	21071.36	36432.46	21345.74	11392.76	13163.37	16194	21661
1779	408.31	209	33228.7	5189.769	52984.83	15937.43	34535.12	10518.92	19331.21	27894	12390
1780	408.31	1107	2892.39	2114.474	2871.33	3261.16	2650.575	3693.089	2456.153	2907	2864
1781	408.31	1076	2928.77	2851.611	2697.635	2547.088	3062.908	2961.671	1827.812	3006	2340
1782	408.31	42	2731.5	2978.129	2922.024	3360.195	3064.515	2995.613	1904.44	2199	3096
1783	408.31	1177	3173.42	4449.789	2120.967	3365.98	5133.873	6915.204	5631.937	2994	5013
1784	408.35	86	15559.5	2302.483	1024.744	8696.698	23771.6	4410.464	5390.32	9202	6303
1785	408.37	1048	2344.04	2232.34	2426.528	1790.92	2319.771	4734.975	2169.05	4680	2997
1786	408.37	144	6952.27	3519.545	9363.66	5501.68	5661.963	5924.052	3748.512	6765	5788
1787	408.37	203	2981.69	2430.475	4581.274	3339.572	2395.061	3031.903	3615.442	3332	3589
1788	408.68	72	3056.37	1892.757	2546.643	1368.719	766.2046	3508.751	1318.513	2712	2713
1789	408.77	63	5191.4	1595.73	2879.28	1575.005	870.2463	2207.843	2477.713	2772	2379
1790	408.77	78	7128.8	1590.575	2958.199	1610.136	1455.36	3288.324	4746.887	8131	2095
1791	409.17	78	59649.6	5043.382	7469.254	13471.95	41486.12	11742.53	19349.56	22647	14919
1792	409.28	91	2060.95	2869.76	2020.267	1539.052	5621.415	3917.994	2067.45	1637	2636
1793	409.31	209	8768.68	1498.577	14165.34	4085.355	8906.84	2844.16	5183.754	6798	3413
1794	409.31	1177	1859.65	2435.197	1252.369	986.598	1407.546	2531.998	1053.029	1748	2521
1795	409.31	195	3636.18	4014.258	5389.29	9049.456	5706.288	3154.048	3456.496	4780	5443
1796	409.37	142	1751.71	1177.602	2026.355	1997.395	1647.699	1715.502	1626.685	1835	1283
1797	410.18	78	14925.4	1000.46	4322.498	3783.891	11151.02	1746.428	5494.66	6394	3426
1798	410.24	63	2204.16	852.7016	2350.134	1955.488	3857.041	2997.553	2217.366	2758	1266
1799	410.31	105	2097.83	1612.79	2663.799	2122.552	2139.286	2205.425	2391.206	1830	2548
1800	410.31	208	1302.76	392.0248	2566.162	1755.429	1713.225	803.1879	1012.499	1466	1028
1801	410.34	124	5296.73	2499.86	5703.496	2840.26	4663.731	4142.009	4494.709	3176	8204
1802	410.37	148	8411.65	6389.488	18450.07	7996.16	15372.1	15561.7	11804.8	13279	11720
1803	410.86	62	13374.9	5202.951	5820.48	4766.491	2206.789	6924.508	1170.147	8082	11590
1804	410.86	79	20898.5	13611.03	17184.5	6934.725	8612.998	16450.29	17358.26	19432	8610
1805	411.09	101	1105.87	1474.289	2059.922	1692.18	937.32	1118.205	404.3244	752.3	2164
1806	411.18	140	1283.7	1868.541	842.688	1106.473	1689.482	1207.91	1714.86	1686	1456
1807	411.35	125	2378.62	1588.187	2129.108	1810.189	1809.879	2997.655	3121.02	2784	1872
1808	411.38	148	2669.95	1922.778	5469.282	3449.827	3241.963	5008.868	3703.64	3583	3760
1809	412.24	64	802.715	1388.163	1733.105	1681.818	1829.44	1384.256	1502.63	1566	1329
1810	412.31	116	1941.33	966.735	2492.769	1069.504	2077.938	2055.129	1683.134	2396	1697
1811	412.34	104	8368.69	8440.136	8135.027	7813.907	9413.411	6368.26	7379.184	8549	8519
1812	412.39	140	7877.37	2390.593	16962.8	9682.31	9117.525	15497.72	9824.943	12919	10269
1813	412.39	192	3198.65	1191.821	5474.95	3218.459	4246.327	2705.003	3387.492	5639	3033
1814	412.85	62	10697.6	5037.863	4165.522	3374.586	1554.837	4593.703	4479.018	5376	6657
1815	412.85	80	15853.4	10668.74	12260.33	5952.548	5490.459	5877.606	12789.26	13658	7758
1816	413.26	470	6130.58	5265.012	15686.15	6841.833	6094.189	7932.141	953.6047	8646	14645
1817	413.26	1122	4807.21	5248.464	6008.179	4828.244	7754.114	5520.547	4057.988	6727	5090
1818	413.26	2	6960.26	0	3316.647	2674.993	16231.4	34832.76	2873.394	13541	4232

1819	413.26	1036	3446.88	2926.16	3928.824	2255.773	2720.464	3665.636	3250.935	4575	3166
1820	413.26	1183	21406.5	11723.51	8066.346	14832.42	8868.15	22184.28	14938.8	20302	8489
1821	413.26	1146	7442.08	7062.807	11234.86	7221.13	7509.922	9917.77	7535.65	9543	9573
1822	413.26	1072	11964.1	14243.75	37413.87	7409.892	6609.68	9675.273	10840.05	10449	9157
1823	413.26	1057	6051.54	7777.829	5820.373	5489.753	8434.963	6448.869	5390.114	6790	7054
1824	413.26	98	73714.6	84917.89	50938.26	66963.8	72068.72	79714.17	12288.8	18038	90242
1825	413.27	1094	11406.5	10412.68	13356	11573.85	13757.24	13631.07	10623.25	11586	12568
1826	413.27	264	8841.17	7200.75	7933.502	8107.753	7305.738	6584.536	6593.69	7010	6485
1827	413.27	200	1644.77	12212.13	1520.133	12543.05	14672.52	11743.23	5558.043	7711	13987
1828	413.27	148	11600.4	20039.37	12535.04	13195.44	17574.36	12740.45	10009.51	14122	13520
1829	413.27	217	11986.7	7844.686	8205.114	12351.2	11778.27	9120.062	6788.321	9678	8999
1830	413.27	46	15767.2	34534.9	9333.36	14927.92	24494.73	17465.91	7034.509	10145	22682
1831	413.27	178	16060.5	13744.88	10923.37	12857.77	11353.59	13733.48	21589.59	20051	15183
1832	413.27	390	13584.4	9420.171	19646.67	15571.88	15872.4	19956	17032.14	19642	16822
1833	413.27	241	9689.02	8607.629	9291.645	9356.595	10329.93	7555.236	6582.737	9096	6120
1834	413.27	309	4910.03	6642.693	5409.514	5589.727	5174.718	4884.921	4842.453	4926	4931
1835	413.34	104	2842.98	15983.46	2321.504	1929.731	905.196	37148.16	1945.018	1450	3074
1836	413.39	140	2458.78	1157.104	4954.217	3950.116	2922.3	5854.798	2655.664	5142	3037
1837	413.39	193	975.065	525.6635	1666.513	1020.942	1430.02	917.181	945.0107	1328	794.9
1838	413.88	78	1276.91	5230.573	4031.189	3668.388	5978.205	4519.93	2088.943	1021	1938
1839	414.21	80	94793.4	49799.9	110964.3	43422.68	46106.68	52579.56	63484	3597	43402
1840	414.27	266	1859.85	2133.274	1833.53	1976.939	1762.045	1829.408	1663.19	1907	1782
1841	414.27	470	2307.31	2177.721	5981.792	2773.122	3520.684	2725.723	2049.317	4131	2100
1842	414.27	97	18427.2	22336.33	5536.22	22132.11	22528.98	22892.96	7153.14	4879	25355
1843	414.27	1057	2897.54	3501.313	2071.37	3010.745	3280.67	3689.85	3116.989	3146	3853
1844	414.27	1098	3419.95	3067.382	2968.1	3687.808	2471.909	3599.689	3352.349	3252	3504
1845	414.27	1115	2089.43	2311.579	2274.78	1904.056	1825.592	1982.295	1930	1904	2028
1846	414.27	1174	4199.14	3701.163	3451.784	3223.062	2228.597	4929.558	2818.15	5762	3693
1847	414.27	1076	3365.34	2454.284	3551.635	2497	2457.113	3493.854	3818.635	3085	2846
1848	414.27	179	4451.87	3653.311	3303.062	3270.036	3560.699	3956.764	2799.548	3629	4079
1849	414.27	1196	3040.16	3194.555	3347.368	2263.839	0	3573.616	2568.816	3585	2746
1850	414.27	1142	2197.49	2111.272	1637.271	1923.446	1084.994	2181.183	1889.923	2346	2247
1851	414.27	46	5504.13	6184.031	3699.974	6510.878	7457.887	5581.622	2650.253	3622	6696
1852	414.27	391	4017.76	2666.863	5221.868	4443.487	4324.243	5469.183	4299.926	5400	4869
1853	414.27	243	2561.22	2772.252	2705.702	2808.126	2979.284	2495.684	2379.255	2581	2137
1854	414.3	80	4218.32	8606.813	3919.881	4765.626	3580.093	5401.929	1535.271	3050	1888
1855	414.32	126	2192.79	763.1606	1687.31	1133.684	1451.072	1846.043	1175.673	987	1848
1856	414.39	141	1397.75	971.7125	2191.157	1255.341	1298.4	1583.343	1150.152	1353	701.5
1857	414.71	73	2648.31	88.689	1366.909	346.0936	298.1154	565.915	1913.743	1671	770.6
1858	414.85	62	3018.42	1613.228	1430.361	1818.921	360.5042	1697.84	1727.868	1934	1948
1859	414.86	78	3535.55	3069.174	4606.886	3256.773	2763.406	4371.033	5046.588	6302	5560
1860	415.21	78	1540161	1252894	1305391	1489360	1341556	4919.121	1078204	1E+06	8E+05
1861	415.25	60	1783.59	1853.771	1677.371	1220.539	763.7178	1959.665	1275.96	1443	1276
1862	415.27	98	2245.37	7900.499	1509.631	3797.112	3498	5826.683	3257.796	1780	8185
1863	415.32	128	1523.62	808.13	3385.337	1489.696	1571.356	2402.239	966.544	652.5	1164
1864	416.06	90	2304.31	2380.08	6177.859	506.3927	1862.38	1491.988	3441.166	2784	2537
1865	416.21	78	481701	368517.2	372585.8	404552	419963.7	299067.7	371698.2	3E+05	2E+05
1866	416.28	65	5571.68	3968.375	4891.417	1423.118	1569.592	6179.643	3054.022	3806	2937
1867	416.32	101	2402.43	4664.342	1723.088	1320.786	3963.134	686.4	1548.887	2169	2740
1868	416.36	129	5689.7	1878.614	4881.086	2117.867	1191.149	3428.46	3235.105	3231	3032
1869	416.37	50	1856.24	1229.146	1247.662	2119.536	1101.141	1755.533	1187.609	1219	1971
1870	416.37	471	1862.23	1538.148	2977.221	1716.071	2427.451	1981.287	2081.287	2508	2422
1871	416.37	213	3877.19	2514.957	3740.486	3288.891	3150.158	2328.226	3405.086	3981	3907
1872	416.37	1045	2162.46	2266.344	2027.774	1856.552	1997.773	2587.333	2506.75	2187	3705
1873	416.37	1190	2339.33	2486.725	1944.712	2125	2458.77	2540.925	2190.155	2590	2585
1874	416.8	78	12581.6	3276.766	1699.213	1918.42	1527.746	1260.333	1644.256	8986	2415
1875	416.84	63	3538.78	1235.664	2792.334	640.9226	665.6816	2354.625	912.635	2653	1235
1876	417.22	78	73337.4	67347.87	61474.76	75170.19	65752.06	42787.99	52038.38	57629	48841
1877	417.32	136	958.017	1129.03	1502.452	1328.639	1455.336	1675.995	1347.831	1635	777.1
1878	417.36	129	1141.67	1086.412	1998.159	784.4418	1123.005	1397.433	2583.724	1487	1363
1879	418.22	79	12689	11583.37	10298.64	12222.85	10913.08	9144.871	6078.672	8336	5466
1880	418.31	1046	1459.32	1420.844	1165.588	1098.448	995.4861	1608.923	1442.323	1249	1802
1881	418.39	132	11884.6	5836.172	12423.15	11234.03	12428.51	6904.924	8991.806	14411	12022
1882	418.8	76	13219.7	3885.063	5426.642	2209.493	1927.716	3848.771	6579.712	8413	2871
1883	418.8	62	1275.42	2165.726	2315.264	1474.714	767.0843	2318.14	2284.766	2607	3050

1884	419.18	95	2614.22	3973.31	1181.58	3860.996	1951.565	997.985	1599.95	2636	2775
1885	419.24	76	10198.5	4655.836	4537.819	3313.237	8950.148	1829.349	2649.2	1665	1630
1886	419.31	1	824.874	643.797	894.2931	423.054	480.5447	537.0067	771.12	764.6	494
1887	419.31	120	3836.56	4393.312	6212.489	892.8859	6916.48	4362.853	3487.227	3752	5664
1888	419.31	90	3917.69	5356.913	4705.492	6035.603	5345.491	5242.753	4075.302	4446	4109
1889	419.39	132	3345.58	1838.896	3878.381	4133.242	3586.933	1906.857	2459.35	3920	3125
1890	419.79	73	3536.14	1830.661	2620.704	2382.694	1076.219	2206.721	2076.401	2116	1890
1891	420.24	76	3365.61	1700.543	1508.392	1579.013	2057.643	986.1615	1259.062	1583	943.1
1892	420.32	106	1566.72	1937.082	1738.136	1792.98	1235.471	1526.344	1557.292	1748	2286
1893	420.32	2	355.586	242.8833	246.015	168.7885	273.0729	241.615	324.4431	244.1	226.5
1894	420.79	74	1260.14	421.1516	2210.555	1166.1	99.24444	2026.347	4016.056	4415	1344
1895	420.88	61	12712.9	8299.86	4243.189	5019.676	1856.986	7031.878	5814.38	7596	10335
1896	420.88	81	43014.7	50788	56156.68	26350.1	43117.23	27806.48	41628.22	46577	39586
1897	421.13	101	2230.3	1482.003	4406.386	0	1900.03	0	608.58	173.1	1845
1898	421.24	65	3769.33	1330.791	2547.249	1359.874	2238.901	1855.802	2093.238	2227	2704
1899	421.32	80	27106.5	13748.51	22557.88	24342.48	27348.38	40025.7	33940.85	37190	15159
1900	421.35	166	1601.3	1164.953	1544.64	1421.738	1391.273	1482.38	1470.27	1540	1621
1901	421.88	80	1101.27	4391.004	6955.404	2565.646	3009.122	2718.261	5502.242	4465	3573
1902	422.03	899	1462.13	785.8408	886.3833	950.1943	858.2655	1078.363	958.7336	1204	1179
1903	422.29	468	1421.27	936.6101	6289.295	3296.105	2923.869	1778.244	2541.551	2257	3220
1904	422.32	80	9347.07	5576.099	8182.951	8394.466	9444.15	12352.68	11610.23	11702	5848
1905	422.34	94	5316.09	5571.326	1722.599	1011.776	8616.876	2205.368	3527.743	2028	1483
1906	422.38	1136	1866.32	1797.188	1931.927	1451.029	1902.504	1726.326	1588.642	2373	1449
1907	422.38	167	7233.71	3444.085	4645.92	3481.408	6194.601	4867.422	3528.352	4995	5903
1908	422.88	81	23795.7	19820.99	20152.56	9706.665	16595.99	10833.76	14308.06	17474	18599
1909	423.32	79	1171.37	978.724	2240.187	2574.834	1796.46	2790.19	2806.82	2346	2055
1910	423.33	112	1256.51	715.9626	2346.13	1300.547	1684.108	1319.781	752.1908	1083	1541
1911	423.39	167	1415.8	1075.495	1733.742	1149.907	1559.45	1345.632	1304.759	1462	1435
1912	424.28	1149	978.625	850.9729	893.2185	813.2361	832.9549	947.0577	863.4375	1133	824
1913	424.28	1190	1534.33	1296.798	1242.017	1293.549	1540.59	1698.117	1191.164	1760	1661
1914	424.28	1177	1850.53	3537.35	1117.667	1733.65	1791.004	1882.489	1381.213	1703	1742
1915	424.28	208	6014.6	2492.326	7832.462	7406.549	10368.63	3389.871	6132.372	6733	5461
1916	424.28	98	5461.47	1118.081	1060.369	4310.298	4671.04	748.696	2568.731	2445	2056
1917	424.28	195	5119.61	2203.514	9258.355	8099.104	6924.729	4346.068	5638.57	14655	5201
1918	424.36	1073	2569.38	2086.301	2216.005	2349.35	2202.083	2903.833	2534.288	2537	2538
1919	424.36	1048	2903.13	3050.848	3909.419	1888.73	3441.592	3116.181	3334.277	3030	3731
1920	424.36	111	901.38	4688.765	4835.624	3642.66	3085.253	7020.08	5322.862	5973	8387
1921	424.36	1106	3075.12	2325.443	3728.938	3032.702	2765.594	3230.902	3139.68	3355	4514
1922	424.36	145	10720.5	7895.657	7646.477	10091.49	11312.51	12075.78	6713.13	13741	10830
1923	424.87	62	1992.06	2007.54	1196.831	1292.443	511.0091	1546.29	1467.48	1334	2199
1924	424.89	87	3758.61	4211.443	4881.97	4840.373	1660.031	2578.83	3104.508	6481	2973
1925	425.15	79	35719.2	3443.405	7147.415	6774.384	17082.13	5337.332	13861.56	14557	11752
1926	425.21	1082	3200.49	2477.958	2961.6	3583.716	3469.316	2286.929	2616.784	2987	2019
1927	425.21	111	1872.31	2299.85	2342.152	2599.689	4019.52	933.065	723.5712	3212	3246
1928	425.28	195	1655.65	672.014	2334.926	2165.342	1786.339	1033.589	1619.975	1948	1604
1929	425.29	208	1858.63	1555.589	1986.947	2387.871	2962.298	945.9815	1787.729	1912	1522
1930	425.36	112	1667.75	1521.529	1338.56	1419.274	1616.936	1637.515	1528.426	1622	1788
1931	425.37	145	3397.56	2316.396	3110.358	2915.035	6466.559	2351.984	2065.935	3585	6277
1932	425.41	148	859.95	2027.262	1438.201	2388.531	2359.199	1318.903	953.5167	1121	2654
1933	426.15	79	11366	1730.294	1869.136	1024.744	8892.843	1774.309	2138.88	3776	2435
1934	426.28	208	753.438	447.5695	977.5738	1147.84	2597.363	554.2486	855.8875	963.2	778.2
1935	426.34	99	947.67	428.485	612.4025	438.0875	907.365	468.115	608.58	696.5	1014
1936	426.36	115	1176.77	1118.426	1214.312	1622.677	1913.597	866.2958	797.4311	830.4	1175
1937	426.39	353	1982.26	840.657	2539.967	1475.801	1130.953	1133.742	1143.325	2099	1751
1938	426.83	62	1711.46	2412.844	933.18	1764.47	961.1675	2514.839	2123.38	925.8	3669
1939	426.83	79	9102.3	4648.932	11944.65	3380.397	3547.68	2034.462	10261.55	3488	6462
1940	427.15	77	6660	1013.442	1710.898	1188.786	3290.336	1330.503	1676.605	2112	1201
1941	427.25	475	1705.65	1212.655	1005.893	884.1405	2403.455	1799.9	2101.906	2612	2237
1942	427.3	152	1342.25	1431.421	1129.079	1225.572	2284.214	1703.773	1350.189	2119	1646
1943	427.31	108	1728.95	809.5026	1825.116	798.84	1634.425	1111.044	1139.659	1489	1886
1944	428.04	903	12133.6	5938.69	2425.801	6351.818	7991.965	7646.376	6833.916	10219	8528
1945	428.33	86	219770	21565.62	19503.82	88697.5	238137.7	15966.78	86444.85	95080	92305
1946	428.38	131	1319.89	892.32	1376.13	1378.445	2635.21	1389.948	1344.414	1511	1032
1947	428.7	70	16695.8	11313.33	9899.792	10014.09	7354.504	13981.92	12167.96	13836	15097
1948	428.83	78	7655.56	5016.206	9127.742	2882.58	2187.424	3013.08	6067.2	9504	3723

1949	428.83	62	4085.43	1698.503	2299.993	1501.608	701.8316	1758.206	1676.28	1916	950
1950	429.04	903	1722.04	776.058	714.8471	1111.109	1287.848	840.9733	892.664	1559	943.3
1951	429.19	72	1259.39	2374.082	1432.138	1833.25	1456.833	923.1014	563.3	609.4	1095
1952	429.24	1	936.695	609.7	715.3478	457.3219	657.8473	548.4726	489.8939	453.5	611.3
1953	429.24	97	21709.1	24386.92	9424.983	25315.53	19534.86	25886.45	10775.89	5661	24638
1954	429.24	1064	2219.8	2428.8	2050.376	2177.107	2025.65	1936.944	2321.437	2282	1763
1955	429.24	391	2945.29	1717.265	3442.658	2712.373	1830.826	3314.752	3268.277	3471	2899
1956	429.26	137	3214.1	2124.136	4280.248	2697.582	1534.683	1655.065	3108.747	3164	3252
1957	429.26	1101	863.197	287.2255	731.8033	301.7367	498.0714	691.1233	759.3833	781.6	326.7
1958	429.28	62	1503.95	1508.709	1516.178	1479.566	1068.238	1389.911	1272.075	1717	1187
1959	429.32	144	2491.57	5535.84	1039.013	1740.114	2980.687	1436.07	1164.108	1501	1500
1960	429.34	85	61951.9	7274.813	6409.131	22634.91	64988.93	6946.183	25808.89	25415	31194
1961	429.38	108	1624.56	1768.542	1811.835	2288.11	2417.215	2604.761	1816.742	1347	2001
1962	429.4	2	550.198	283.0273	1130.091	317.3567	396.97	478.4071	522.7629	372.3	292.3
1963	429.4	50	1889.04	1914.662	1416.572	2112.429	1130.899	1862.384	1872.547	1427	1893
1964	429.4	1076	2454.18	2300.941	2349.976	2224.492	2804.002	2723.774	2896.844	2395	2485
1965	429.4	1151	1029.77	653.7365	990.1418	657.286	854.8361	743.1627	5245.095	1831	787.2
1966	429.4	1050	3845.64	4340.459	5886.946	2218.897	1632.321	4204.72	4250.944	3566	4465
1967	429.4	1114	1556.11	2124.306	1754.771	2264.905	2344.541	2378.922	2577.795	2402	4362
1968	429.4	1097	1357.05	1882.72	2497.32	2805.568	2059.98	2382.792	2849.122	3039	2485
1969	429.4	211	4496.89	1829.699	1816.788	2662.597	2423.161	2028.759	2098.827	2413	2644
1970	429.4	1198	7408.87	4362.471	1834	2919.845	3871.77	2833.253	1097.647	1880	3794
1971	429.4	1175	3367.98	3204.544	2116.063	2110.964	2224.6	3685.012	18699.36	4006	2927
1972	430.24	78	61990.2	30848.97	54870.06	57243.17	52194.68	102276.7	53980.26	55361	75851
1973	430.34	85	13698.5	3045.743	3337.731	7046.549	15223.19	2298.036	9635.451	11141	5581
1974	430.41	1197	672.534	1325.632	635.6277	753.61	0	0	0	743	1065
1975	430.7	70	34604	24182.71	22244.15	20380.44	15306.28	27829.99	22735.75	31673	29558
1976	430.82	63	805.747	796.1745	931.3033	837.6722	408.5838	452.844	674.24	765.7	1105
1977	430.84	78	2327.76	2591.14	2617.961	1295.586	3508.283	2000.853	3238.112	2350	1623
1978	430.91	84	209076	256627.9	2603.84	117586.9	241017.2	150823.1	173913	2E+05	2E+05
1979	431.18	587	1593.65	497.4743	763.7309	589.7345	925.6512	1320.217	827.1836	1046	1515
1980	431.21	73	6413.35	3758.288	1963.449	4861.521	5342.011	2965.408	3645.841	2261	3441
1981	431.23	111	8133.07	6156.713	5574.942	6820.163	7999.053	2149.47	1833.104	2238	2375
1982	431.25	79	17009.6	6938.723	16853.67	16850.98	15788.68	24673.14	17457.79	19544	21397
1983	431.38	127	14363	4584.524	4670.66	4864.657	5080.31	5495.957	4622.94	5016	5347
1984	431.38	469	3802.7	3332.77	5683.131	5060.838	4432.086	4169.672	3626.269	4720	4235
1985	431.38	2	0	4172.901	1705.184	927.2158	4492.824	1209.556	1931.419	2067	1104
1986	431.38	1050	11662.9	14378.88	37491.58	5197.082	28787.75	18108.94	12489.1	10835	18742
1987	431.38	1175	20601.4	11491.44	11618.94	10309.81	12181.9	17594.36	33240.12	24208	12560
1988	431.38	50	5477.44	7363.611	4052.923	5671.907	5217.371	6546.979	3430.644	4274	6041
1989	431.38	208	121629	22368.57	131894.6	75581.62	159230.5	36926.09	81256.01	98439	48472
1990	431.38	195	42761.1	19691.93	74687.45	96369.9	46635.54	30447.5	48487.52	58331	62351
1991	431.92	85	20435	23883.5	26561.88	11817.63	26341.9	14197.94	18221.72	19615	18456
1992	432.24	78	460542	398316.8	433244.1	417274.7	432353	283944	353444.2	3E+05	2E+05
1993	432.24	1038	6138.7	7536.33	5996.445	3847.363	6959.626	6919.952	6850.852	7142	8503
1994	432.28	470	2192.89	1801.136	3076.511	4210.382	2744.753	2361.833	2181.603	6433	2928
1995	432.28	61	14727.9	10298.71	14552.24	7255.514	9535.926	17684.75	9038.121	9892	9761
1996	432.33	139	3955.15	2157.745	4401.729	3766.11	3787.93	2501.036	3576.211	3797	3844
1997	432.37	87	1496.06	255.78	255.78	1018.248	3616.004	0	2171.74	1526	1469
1998	432.39	1050	3182.56	3604.763	2802.252	1243.543	1441.44	9531.6	3613.341	3231	5254
1999	432.39	51	1391.16	1036.603	1069.511	2004.656	1030.4	1528.8	1302.827	1433	1672
2000	432.39	1088	2559.48	2588.962	2767.814	3449.686	2585.735	2874.408	2656.5	2597	2838
2001	432.39	1115	1915.36	1718.17	1907.951	1861.009	1783.88	1972.768	1957	2065	2099
2002	432.39	1150	1548.23	513.8036	2345.138	1049.094	984.3965	1243.651	3182.12	1832	1088
2003	432.39	1190	2468.91	4147.171	2590.465	3624.826	7628.021	3189.42	5784.646	2937	3981
2004	432.39	2	389.903	396.0968	993.5617	349.2833	385.7283	386.2387	502.6797	853	279.5
2005	432.39	207	23093.6	6377.687	34005.7	19540.77	55231.22	9587.976	21273.04	26368	16150
2006	432.39	1175	5567.4	3257.4	3310.688	2911.662	3601.25	4756.244	8789.4	6590	3623
2007	432.69	70	32839.4	23353.97	20688.16	20401.48	14640.1	31083.14	25445.32	32178	31175
2008	432.78	75	2323.98	1054.693	831.8886	525.03	369.72	1204.814	2521.366	1964	1605
2009	432.92	85	10782.7	12364.24	9948.551	8242.058	10931.62	8716.411	8646.253	10565	9953
2010	433.09	95	2762.09	2514.429	2790.896	880.308	3409.335	822.1886	1471.451	1613	1484
2011	433.14	100	2056.45	1426.837	11808.18	2275.196	2454.598	94.07143	398.8917	569	2229
2012	433.2	62	4684.26	1782.483	1954.911	5872.797	1842.337	3635.839	1171.003	1054	1948
2013	433.24	78	130381	109669	120456.6	112326.7	115837.7	86200.92	90055.51	1E+05	66458

2014	433.24	1039	1760.9	2226.952	1784.67	1441.59	2517.251	2255.84	2225.25	2396	2285
2015	433.28	61	1583.3	2742.722	3071.345	2510.5	3244.813	3920.482	3036.662	3595	2980
2016	433.39	196	2127.89	868.527	3356.467	4027.669	2148.995	3002.698	2143.697	2506	2826
2017	433.39	208	4113.57	1249.167	5956.684	3642.863	9407.477	1575.9	3841.424	4083	2466
2018	434.24	79	24577.7	24989.69	22738.5	23502.26	20867.31	19102.46	21601.41	22133	7329
2019	434.31	79	71274.8	52898.74	38155.99	197264.5	30399.3	231337.7	99513.88	85740	2E+05
2020	434.38	103	6207.78	4459.399	5771.52	4156.74	5072.32	3114.415	4035.587	5799	3774
2021	434.69	70	18083.5	13965.9	11538.29	11795.44	8610.699	11814.9	13443.15	16928	16874
2022	434.82	64	1387.43	685.1	1054.13	443.989	279.5	1610.714	845.48	823.8	650.2
2023	434.82	116	781.2	675.4364	1187.23	1174.771	1421.949	1710.45	1193.505	506.3	1139
2024	434.9	81	13155.4	14887.62	14148.4	6564.264	13042.43	5522.16	10949.72	9829	10424
2025	435.16	93	1365.21	2214.442	1867.32	2051.822	1756.522	2652.988	2633.133	832.9	1948
2026	435.24	76	7634.55	2350.964	4768.243	4771.629	5216.309	4005.004	4092.039	1301	2145
2027	435.31	79	20588.8	19712.09	14730.12	58295.73	11121.58	69030.26	27951.8	30092	51440
2028	435.39	102	2383.38	1626.743	1514.417	1829.569	1454.545	1056.506	2315.837	1775	1424
2029	435.79	79	3449.37	2308.656	2423.241	3145.576	4127.667	2033.196	4474.884	3213	2575
2030	435.98	87	6517.25	2501.136	9348.147	2293.75	5700.609	2539.686	4448.208	3662	2183
2031	436.31	77	4154.02	3958.505	4651.579	11366.5	1968.849	11954.82	979.5157	6176	9488
2032	436.34	2	9083.82	9112.457	6674.503	5568.051	25164.47	8255.81	10278.7	73238	8201
2033	436.34	1096	26222.6	25773.94	25546.13	30787.09	21764.72	27623.65	23455.47	27282	26297
2034	436.34	98	33853.9	25600.17	9181.028	23617.93	35532.99	45084.09	9058.245	8921	30792
2035	436.34	1171	39858.7	20179.78	23751.01	20212.99	21802.32	28223.91	23759.71	39338	25114
2036	436.34	1050	29849.4	39097.06	37224.5	34440.37	81902.01	38102.23	33105.96	43285	50470
2037	436.34	146	21794.4	24341.03	23103.28	21444.83	24428.5	24376.16	23172.57	1E+05	20944
2038	436.34	1192	27134	34396.62	23145.81	23491.99	39196.18	22466.82	0	30305	29340
2039	436.34	1114	20840	17771.83	22790.45	19754.32	18550.14	20054.37	20142.43	21732	50075
2040	436.34	1076	24037	22176.12	98739.41	23468.69	23800.15	24623.35	27529.34	22092	22979
2041	436.34	49	39675.8	54430.14	17585.66	48381.46	17072.25	42955.92	17232.54	18904	42476
2042	436.34	391	39830.3	24211.57	55255.28	38222.74	39086.7	49924.22	40145.26	51250	46850
2043	436.34	17	27255.6	29186.37	26696.32	28672.01	33176.86	40186.72	20575.02	27107	39621
2044	436.34	201	3824.61	24136.78	3979.87	3594.049	3218.447	25562.72	1779.06	2522	2603
2045	436.34	238	17235.1	15402.94	16933.01	18142.37	17962.01	15925.46	13886.6	17448	14994
2046	436.69	71	6238.93	4520.794	3610.776	4683.462	2788.191	6313.586	2921.17	5758	6048
2047	436.82	63	1054.07	2669.866	1042.427	806.679	396.5436	856.9	817.8571	784	1249
2048	436.86	80	25981.4	5592.986	6202.179	2302.98	11716.91	10247.78	11854.88	21690	15107
2049	437.19	80	827372	653135.8	621337.3	541763.1	854455	375079.3	575342.6	6E+05	4E+05
2050	437.23	62	10703.3	5101.443	5639.731	3350.901	4150.059	967.8064	5748.84	6790	6913
2051	437.34	470	4280.68	4609.565	4237.802	6360.74	5998.169	5795.759	4899.92	11120	6351
2052	437.34	97	13314	2090.504	2773.299	6487.84	5245.589	10802.78	4191.211	4750	10198
2053	437.34	1076	7078.33	8730.16	29748.63	8845.467	7431.375	9016.435	8421.992	8218	9335
2054	437.34	1188	12019.5	9560.353	7830.218	7627.369	9629.167	6857.024	3897.995	14601	8325
2055	437.34	1096	7951.93	8532.048	7291.828	8103.78	6704.408	8102.408	6848.811	7934	8940
2056	437.34	1048	9776.68	14327.22	10609.12	9914.139	12297.31	12719.1	9436.175	12368	17090
2057	437.34	20	9622.78	11657.55	8649.636	9837.667	11340.41	12343.45	5149.867	8870	11974
2058	437.34	209	8623.4	5949.03	7045.271	7032.907	8961.918	8273.218	5402.57	6593	7150
2059	437.34	1	2935.68	10665.71	6045.315	423.1863	10380.12	3663.551	3519.238	4494	3975
2060	437.34	1114	4956.84	5871.055	5375.808	4514.016	5030.817	4408.2	4590.113	4884	5555
2061	437.34	50	11189.2	15853.6	6372.004	12306.08	11226.39	12488.16	4527.431	6253	12683
2062	437.34	392	12646.3	9057.196	16358.36	11424.63	12340.3	14201.25	11906.59	14653	13428
2063	437.36	1061	639.36	321.1792	468.45	632.2705	10889.21	1389.029	1166.283	714.2	763.3
2064	437.86	80	2411.68	2363.07	2947.345	1349.755	1545.142	2114.149	3043.502	3400	1341
2065	437.87	61	819.027	440.572	425.8722	843.4702	442.2012	461.6367	357.0233	369.2	540
2066	438.2	80	236029	179085.2	189554.7	152663	238795.5	98825.35	171340.9	2E+05	1E+05
2067	438.24	62	2115.96	1402.017	1605.084	2430.022	3600.177	2534.706	2206.912	2230	1757
2068	438.3	166	7784.66	3036.664	7803.625	3256.734	5363.546	5909.071	3073.184	5152	5657
2069	438.35	98	5292.47	2283.467	3339.745	1139.036	1750.848	4815.131	3935.304	4227	2038
2070	438.35	1049	3793.24	3904.904	3690.763	3175.78	4093.998	2506.417	2293.044	3724	1723
2071	438.35	391	2379.08	1706.984	2953.509	2208.458	2253.825	2551.296	2008.593	2855	2572
2072	438.35	48	2299.2	2239.755	1662.129	2423.341	2264.066	2505.925	1540.872	1661	3016
2073	438.35	2	634.66	545.5543	335.5601	415.9561	262.332	517.3154	895.8331	675.2	484.1
2074	438.37	126	6041.9	4731.984	5396.08	4684.7	5571.52	2589.398	4086.796	3649	4254
2075	438.38	1073	2631.98	3237.487	1651.407	2400.116	2430.944	2960.853	1803.515	2523	3638
2076	438.38	1054	3965.25	2921.342	4489.6	3841.001	8652.46	3761.076	4665.943	9748	3700
2077	438.38	1137	3361.58	3575.264	3324.247	2067.748	3890.116	5262.49	3217.767	2688	2192
2078	438.38	1093	2565.5	2435.142	2734.288	3077.354	2514.376	2880.842	2751.536	2977	2983

2079	438.38	167	9914.52	11952.48	8443.38	8447.391	10599.44	9598.755	7508.865	9708	10406
2080	438.73	65	3352.11	3068.927	3126.134	1755.797	1260.8	1539.667	3699.675	2063	1441
2081	438.85	61	832.05	1242.988	1477.98	1056.45	533.2906	780.444	484.4856	895.1	1407
2082	438.86	81	11058.5	6423.688	11829.6	3867.539	2744.862	4525.413	8564.133	9796	7714
2083	439.2	80	43056.8	36226.44	36310.34	28208.63	42616.9	19612.07	32961.61	32777	20732
2084	439.28	218	831.85	517.5981	693	767.811	738.7707	659.7855	554.5638	556.5	760.7
2085	439.3	136	4902.38	2017.253	5538.63	5875.978	4286.46	5564.457	3539.146	5772	6414
2086	439.38	167	4693.31	3244.046	2346.19	2428.376	3316.107	2893.057	2164.698	2861	2998
2087	440.2	79	8353.38	4880.871	6168.32	5349.469	4633.086	3937.527	4710.708	6516	3123
2088	440.3	136	2040.3	1326.576	2432.999	1319.743	1737.946	1440.545	1563.618	2678	2362
2089	440.36	111	11754.7	7393.542	14231.1	9477.51	16567.53	12565.43	9155.277	11049	10866
2090	440.36	1073	2106.95	2391.943	1707.449	1656.766	2044.169	3738.281	2033.384	2477	1872
2091	440.41	411	2158.72	1341.543	2968.541	1963.987	3171.226	3535.631	2650.336	2349	2101
2092	440.72	65	10196.4	3324.301	5416.647	1175.936	1999.4	3215.707	2805.895	6388	3792
2093	440.85	79	3499.28	762.4973	3080.642	1539.64	425.76	1261.11	3065.916	2939	1559
2094	441.21	80	2890.55	2883.908	2728.783	1007.597	4928.453	2350.178	2921.123	4296	2398
2095	441.23	100	2086.62	684.5563	1268.372	862.3389	1117.935	1094.829	1199.827	960.3	1884
2096	441.3	217	1383.64	1027.429	1117.885	1405.693	1427.476	1248.594	873.1432	917.6	1132
2097	441.33	142	1817.46	1040.774	1873.351	1529.547	1066.791	1615.883	1307.364	1594	1842
2098	441.33	60	909.296	744.9446	1099.922	808.6286	1234.722	1077.406	927.5049	1423	1067
2099	441.36	112	3746.89	2178.842	4761.146	2882.495	3917.743	4410.543	2839.315	3497	3696
2100	441.78	99	2122.56	1681.467	2702.113	2283.013	4794.168	3779.536	2549.706	725.4	1818
2101	441.93	83	5746.87	8497.602	9694.273	4085.695	5395.74	3561.867	4291.471	4476	5435
2102	442.26	93	5480.93	4889.317	6578.478	4526.457	2204.777	8169.56	6020.457	6464	7420
2103	442.36	122	977.825	1793.656	818.8857	1334.83	1879.344	1738.512	2857.75	3448	2648
2104	442.4	1098	3240.91	2798.98	2805.667	2545.192	2873.927	3158.637	2997.8	3280	3331
2105	442.4	1198	1536.69	3189.262	1891.423	1482.542	3675.662	1750.98	0	2078	2889
2106	442.4	1152	2122.7	626.9893	2594.173	704.3591	779.355	924.4125	864.108	2585	1021
2107	442.4	1060	1972.41	9169.533	2166.445	2262.833	3800.64	2647.519	7944.013	2278	3189
2108	442.4	1	585.446	352.9215	412.6166	333.7413	383.0098	486.7198	613.12	512.2	364.2
2109	442.4	1173	4210.06	3669.375	3311.558	3056.175	3636.058	4226.04	2630.188	3669	3637
2110	442.4	1117	2504.84	2267.176	2424.898	2082.336	2169.601	1950.78	2269.039	2325	2266
2111	442.73	75	5655.36	789.6789	3886.182	523.6733	1447.546	1498.776	2501.284	2798	1078
2112	443.25	92	2614.7	970.8543	1704.028	3244.584	1901.34	2297.993	1649.826	2408	1895
2113	443.33	167	2067.8	1215.528	2486.232	1511.643	1845.643	1579.655	1426.074	1842	1540
2114	444.03	916	1291.33	867.4481	1035.231	794.246	843.6099	1231.604	794.6114	644.2	1084
2115	444.22	73	1449.48	1498.959	819.945	1652.455	1418.906	2635.227	1484.73	1136	1737
2116	444.31	85	25741.3	3207.657	3078.56	8610.215	18932.43	1924.065	11369.8	7785	10508
2117	444.38	1	799.84	710.0056	1013.327	589.2169	730.0938	1054.913	788.3351	2778	752
2118	444.38	1061	3179	19257.58	3654.054	3547.122	4390.024	4252.402	3230.135	3975	5014
2119	444.38	1143	3109.07	3421.028	3767.454	2748.051	4072.992	1591.556	2594.576	3134	6196
2120	444.38	1111	4679.56	3724.562	5078.8	3847.761	4059.096	4008.646	3038.369	4307	6001
2121	444.38	1038	2143.38	2187.216	2677.425	1687.658	2232.095	2266.116	3022.538	2368	1991
2122	444.38	1187	17077.8	9882.536	7298.804	5170.071	7350.676	16201.24	6473.025	9271	14417
2123	444.38	206	29363.7	20788.16	28060.37	65105.16	37229.93	16291.8	21601.98	27910	27007
2124	444.4	140	4801.64	4505.378	4399.408	3777.536	5223.711	2573.436	4022.957	4307	4102
2125	444.67	70	9813.83	5977.469	858.4229	5397.078	3420.267	8160.607	6509.269	7988	7974
2126	444.85	63	1091.37	561.4567	1065.717	1565.615	703.1289	1195.92	951.3314	872.6	1023
2127	444.93	85	48534.3	64164.47	66046.89	22123.99	58518.83	36061.37	44164.5	46201	53953
2128	445.12	131	2502.13	1553.968	2437.055	1566.83	1676.493	2847.152	1832.04	1119	2323
2129	445.23	1036	2126.73	1734.605	1554.531	1715.825	2634.717	2049.454	2053.79	2281	1824
2130	445.24	77	16716.4	6154.003	4820.885	25936.81	8696.179	13537.07	10115.22	12391	33141
2131	445.31	86	10624.8	2231.692	3062.864	5045.189	5901.135	2307.642	3227.251	4463	5092
2132	445.31	111	3047.29	1254.579	5067.118	1239.84	2548.505	2310.789	1442.347	2858	2364
2133	445.38	1152	83.3	481.1056	1892.295	376.781	396.2063	535.4105	605.068	546.2	525.8
2134	445.38	1177	2598.59	2872.137	1604.433	1808.73	2280.075	2407.161	3073.712	2184	4357
2135	445.38	1190	2307.99	1878.543	1934.305	1641.178	2053.23	5120.395	2698.626	2482	1994
2136	445.38	206	8494.63	6045.543	10645.92	20366.88	20536.54	4980.344	6879.938	19297	8089
2137	445.41	140	1651.17	942.2241	1480.05	1358.28	2147.452	1290.642	1325.61	1298	1049
2138	445.93	85	5265.38	6661.363	6674.507	3759.231	7621.074	3891.672	5305.12	6035	7146
2139	446.25	78	8220.7	5875.814	3870.303	9061.235	8789.776	9359.614	8454.4	10343	12161
2140	446.26	98	5417.83	694.2933	1250.533	3573.912	4140.554	1055.623	2611.204	2558	1089
2141	446.38	209	3345.47	892.0032	1117.092	2225.563	2273.429	1705.728	1582.433	1821	1671
2142	446.42	183	2546.43	2266.903	2161.857	1867.097	2120	1725.929	2851.987	3348	2808
2143	446.67	71	23684.7	12987.34	14159.86	12063.12	6672.948	18414.06	13794.67	15482	19190

2144	446.85	62	1574.4	1687.32	1129.34	1677.94	528.1334	1461.592	734.152	714.4	1269
2145	446.89	83	55324.8	46141.33	82342.89	36267.45	49709.59	38239.2	49293.8	56854	51479
2146	446.93	85	2061.94	2869.202	66099.19	35080.46	2445.363	38402.97	46835.29	2022	3391
2147	447.25	78	1810.2	1778.663	2615.566	4103.324	3344.815	2736.948	3500.612	2565	3117
2148	447.89	84	5814.7	7053.988	9077.228	5569.927	5036.078	4868.761	5461.644	6334	5145
2149	447.99	88	5913.06	2518.716	9370.307	2535.938	4088.742	2708.881	4093.184	3183	3984
2150	448.14	111	364.157	92.5	62.35714	189.25	0	536.1514	779.71	1108	2215
2151	448.23	69	2718.21	2140.019	836.7542	2783.919	3057.84	896.1237	2347.591	1820	957.9
2152	448.29	2	310.249	367.3777	408.0912	232.4182	390.2364	213.3743	373.0058	445.6	292.3
2153	448.29	115	880.256	3244.965	1961.703	3263.384	2817.888	1880.804	1749.368	2372	2648
2154	448.29	1046	3995.15	2652.878	3318.671	1545.017	3964.995	3700.305	3947.348	3239	4007
2155	448.29	1082	6301.14	6498.9	7440.107	5212.659	6600.223	5931.1	7567.14	5333	6418
2156	448.29	1190	1468.3	1524.938	1025.316	1276.398	1641.335	1123.407	1587.949	1653	1557
2157	448.37	105	1077.85	1622.245	553.384	926.1	1638.107	606.8236	1478.229	1826	891.5
2158	448.67	72	23307.1	14142.54	15422.55	11203.92	7681.166	20514.39	15344.28	16895	17565
2159	448.76	66	4264.54	6782.118	2010.32	5598.17	1363.783	8997.75	9403.211	3382	1948
2160	448.89	82	7984.27	7669.042	10702.07	5602.501	5170.595	4995.174	4816.628	6255	6848
2161	448.98	90	6545.28	277.9075	1867.511	2295.633	11241.29	0	2793.444	4585	3985
2162	449.15	74	993.885	1476.511	936.2595	1879.981	1471.613	1315.735	1539.555	1101	1314
2163	449.21	67	717.367	783.492	1064.254	1100.717	1828.251	1077.373	837.056	1437	1645
2164	449.29	1082	2376.4	2630.375	1774.586	1686.549	2069.74	2466.75	1499.755	2086	1735
2165	449.3	115	1728.88	974.8629	1634.15	918.5214	2550.324	2442.23	2415.221	1455	3772
2166	449.33	99	11701.7	4513.152	2581.6	4684.114	5378.576	6403.432	4366.514	1245	5251
2167	449.34	1181	5281.43	3157.672	2556.988	3567.473	2915.153	4892.815	3793.003	4552	4145
2168	449.34	1097	3560.68	2937.63	3760.971	586.715	3330.214	3806.378	2598.424	3567	3431
2169	449.34	1154	818.4	696.9857	739.97	589.8	639.4015	894.4	759.416	879.4	820.4
2170	449.34	1119	2579.85	2285.603	1942.92	1693.251	1731.026	1677.568	1826.009	2088	2103
2171	449.34	1058	2238.41	3214.493	2259.6	2226.814	3480.073	3680.373	3053.986	7081	3455
2172	449.34	391	6021.9	3385.8	7511.918	5833.084	6214.014	7086.886	5635.024	6838	6839
2173	449.34	49	3027.89	3604.156	1844.239	4009.535	4774.794	3547.1	1868.745	1699	4269
2174	449.35	80	2366.36	3434.483	4090.966	5714.007	3086.688	10865.38	5006.359	4188	5864
2175	450.32	99	2347.35	1638.66	735.0951	2282.813	2750.322	1486.159	1313.069	1426	1357
2176	450.32	1047	4867.82	5423.078	4613.702	1132.449	4801.333	5936.009	5355.312	5174	8133
2177	450.32	1089	3933.01	3902.08	3622.526	3351.325	3565.673	3777.265	3734.953	3629	3843
2178	450.32	1109	3903.75	3382.641	4391.695	3431.835	4322.433	3557.594	3462.489	5968	4173
2179	450.32	1129	2714.58	2622.491	2759.651	1523.977	3146.598	2348.339	2460.967	2486	2714
2180	450.32	1073	4013.06	7364.398	3650.79	6428.155	7106.4	14549.77	16567.88	5815	7849
2181	450.32	140	2669.36	2485.193	3077.913	2211.143	1499.411	2824.377	2459.258	2549	3276
2182	450.32	123	3416.63	1452.287	3150.063	2542.932	2723.57	2929.647	3410.329	1264	3336
2183	450.34	392	1900.76	1047.674	2139.773	2087.784	2120.487	2055.703	2402.831	2215	2527
2184	450.34	48	1262.88	1583.415	1340.782	2324.785	1563.713	1579.311	1432.847	1252	1623
2185	450.38	86	112513	11276.44	6981.234	56485.18	174639.6	6844.817	46215.66	50239	50028
2186	450.66	71	14553.2	7887.843	7956.96	6315.754	4194.471	10805.35	8710	10973	11658
2187	450.75	62	5930.22	3174.863	6015.283	2682.132	3017.602	1967.794	3117.08	6872	2899
2188	450.87	80	3385.96	3022.294	8269.838	1539.64	3734.186	1432.56	4323.219	4798	3196
2189	451.16	77	5633.5	4324.374	2726.322	2823.458	6001.246	6401.627	3174.813	3884	3379
2190	451.31	103	1941.71	1512.304	894.1714	1626.66	1833.366	1432.363	1763.619	1268	1607
2191	451.32	1110	1891.33	1013.308	1138.151	1054.011	1280.664	1061.255	1020.394	1167	1453
2192	451.38	86	36917.8	3391.108	1537.444	18129.45	55309.33	3076.368	14649.53	17863	18698
2193	451.71	80	8214.58	5377.366	6160.008	4166.781	5383.777	4907.28	5942.831	5381	4077
2194	452.22	79	4985.1	2051.444	5663.825	2834.172	4161.336	2246.63	4572.416	3775	3360
2195	452.32	100	1007.85	1683.539	1670.911	917.28	2325.937	2357.231	2033.736	1819	2204
2196	452.33	139	1780.73	1251.608	1602.368	2234.216	2308.554	2592.19	2386.286	1555	2182
2197	452.33	1050	2077.32	2519.649	3580.055	3441.403	3696.709	3209.376	2721.097	2700	3229
2198	452.34	1106	3504.84	2241.9	4347.973	1030.08	2251.397	2748.653	2534.583	2686	1984
2199	452.34	1	240.852	241.472	279.063	179.7033	240.0963	200.772	270.084	487.9	223.7
2200	452.39	85	11814.1	2554.02	1293.615	3579.111	13527.32	1862.504	4074.84	3746	3050
2201	452.39	1049	2774.95	2835.56	2636.321	2518.297	5228.559	3386.994	2392.471	2813	4141
2202	452.39	1146	617.307	495.575	609.5833	686.3933	1414.589	752.76	686.93	644.2	804.9
2203	452.39	145	4444.26	5518.217	4289.1	5951.856	3459.997	4100.837	3748.988	6457	7323
2204	452.39	1173	4599.13	3370.889	2206.268	2890.033	1970.048	4919.301	3789.448	4714	3515
2205	452.39	202	7539.6	5579.343	2654.202	4851.602	3153.812	5512.926	6145.25	7133	8656
2206	452.66	71	6110.1	3512.219	3329.112	4371.219	2017.851	5266.632	3111.217	3891	6298
2207	452.75	76	3736.48	1804.079	3353.11	5607.63	1764.806	1630.599	5442.619	2865	1990
2208	452.83	80	4418.65	3551.108	9317.041	3580.61	937.3933	2833.457	3916.961	5390	3914

2209	453.17	80	2403689	1745109	2358301	1071002	1642446	762621.7	1473173	2E+06	2555
2210	453.21	62	5472.81	2778.3	4643.047	2961.045	2247.397	5793.633	2755.445	3517	4014
2211	453.34	464	3801.43	3325.832	6710.256	5520.223	4624.456	5025.55	1233.597	4780	4400
2212	453.34	52	3599.37	5273.407	4270.874	3604.872	6617.265	2546.831	3038.28	3898	3503
2213	453.34	273	10204.5	6426.685	4294.316	5920.067	5715.713	7014.75	4818.764	3779	6017
2214	453.34	112	7089.3	6955.496	9846.618	6458.037	10768.67	3466.825	6749.111	8989	14524
2215	453.34	1188	6033.56	8549.327	3462.648	6400.452	5774.805	7388.589	5170.789	6125	5681
2216	453.34	1073	17673.9	21387.57	18764.04	20476.41	24546.75	16998.16	17097.98	15700	20933
2217	453.34	31	9108.35	6489.768	7494.217	8688.101	9127.17	9317.751	11306.06	8957	9090
2218	453.34	174	8873.66	6996.374	6959.57	6778.776	7194.188	8277.576	7784.858	8524	7886
2219	453.34	1024	31730.2	19202.77	9325.452	26640.89	22958.67	32408.81	31425	28482	15350
2220	453.34	1108	61478.2	24303.38	70409.37	16218.69	20233.69	20199.01	18465.84	20985	23607
2221	453.34	148	9538.35	7956.986	2290.775	8962.786	1420.32	7758.552	9275.442	8540	1537
2222	453.37	71	1006.31	379.6365	740.6792	3251.527	1627.069	845.7098	1085.341	1179	1843
2223	453.39	205	2433.3	2143.746	1278.352	2796.634	1834.774	1404.788	2206.81	2621	2500
2224	453.84	81	3357.08	509.1233	2283.734	2385.805	0	1903.73	2058.426	1513	1304
2225	453.84	1160	4433.11	4143.494	4220.88	3029.153	3841.583	4788.36	3991.439	5059	3154
2226	453.84	1188	3256.81	5587.935	2157.095	3270.055	2837.333	4081.748	2654.4	3244	3254
2227	453.84	5	2763.5	3304.03	2515.805	3066.587	2179.553	4722.83	1688.267	2505	1750
2228	453.84	1023	20814.8	11090.23	15234.68	14657.2	10248.93	18621.35	18744.77	19211	16313
2229	453.84	41	2729.15	2652.288	3532.984	2822.097	3481.799	3490.764	3387.05	3570	2262
2230	453.84	1076	9445.51	12213.83	9384.983	12031.99	13762.15	9957.646	11189.91	10620	11832
2231	453.84	1057	7638.49	7392.894	6534.847	6443.252	8421.829	9049.04	9769.6	8170	7821
2232	453.84	1108	32008.8	16363.77	40426.58	11993.05	11245	12364.21	10774.66	13359	14152
2233	453.84	246	4926.87	3636.165	4686.701	4877.884	23213.38	4966.229	4902.574	5165	4420
2234	453.84	116	13567.2	3422.015	6914.584	6022.562	6607.521	7859.319	7331.251	7016	5233
2235	454.17	79	607790	563120.4	620328.8	308698.3	559038.3	222997.7	433018.8	5E+05	2E+05
2236	454.21	62	990.472	1278.545	1394.974	1097.284	1323.018	1626.08	1008.41	642.3	829.6
2237	454.29	142	4717.11	934.096	4819.966	3117.537	3334.981	2685.183	1336.271	3601	3324
2238	454.34	105	3563.52	1461.6	3015.2	2472.261	3117.838	2133.244	2773.368	2106	1761
2239	454.34	465	1127.54	1119.858	1658.029	2380.397	1557.979	1510.374	1484.237	1006	1461
2240	454.35	1075	3349.41	4332.449	3718.668	4255.611	4518.217	3477.225	3351.566	3307	4018
2241	454.35	1062	3709.68	4759.855	3134.378	3560.36	4645.327	3814.767	3451.787	3755	2971
2242	454.35	1108	4123.15	5276.395	4335.824	3313.975	16657.27	7548.379	3483.863	7681	4577
2243	454.37	123	10084	1268.003	7718.801	8330.833	8804.722	11950.61	3280.116	9634	11545
2244	454.74	65	1941.06	696.141	1450.881	885.168	642.95	1052.98	886.485	4084	746.5
2245	454.88	62	1887.67	523.872	1064.073	896.1	477.4011	877.1625	641.568	975	1365
2246	455.17	80	235669	180677.1	248393.8	121272.4	173049.8	95189.26	176660.4	2E+05	99702
2247	455.25	92	7632.63	2737.746	753.5033	4979.52	4544.355	2843.138	2928.798	5265	4252
2248	455.27	136	1432.23	1821.727	1260.555	2524.405	2214.206	2319.596	2596.134	2261	1738
2249	455.33	217	1467.73	1310.296	1247.822	1606.903	1827.539	1675.021	1342.017	1730	2600
2250	455.37	123	4001.88	1968.101	3786.888	2812.636	3187.938	5436.762	2258.708	1503	3806
2251	456.17	80	59529.7	43909.07	57874.69	28823.7	44631.61	22856.61	40519.22	44649	26440
2252	456.33	116	1896.31	2185.388	3828.889	2981.482	2415.702	743.622	2020.149	1614	2252
2253	456.36	103	3764.53	2661.649	2466.712	2595.6	3842.657	777.444	1759.294	2447	2240
2254	456.44	629	6553.84	2483.583	8155.25	3634.719	8660.709	4858.362	8053.168	2194	6387
2255	456.83	116	1340.47	973.9222	591.77	1165.063	1121.408	1116.36	1178.19	606	1162
2256	456.88	62	1262.77	885.4245	860.78	684.0167	161.2207	615.8233	702.575	615	753.6
2257	457.17	80	12303	9997.296	14219.98	5373.604	9980.93	5373.374	7102.193	10176	5596
2258	457.27	96	1528.69	1310.639	1432.037	1504.5	1297.812	1694.628	1224.322	1796	1850
2259	457.28	256	879.074	668.9813	880.1415	894.6286	766.7054	867.7251	936.6522	855.6	829.6
2260	457.36	103	1739.46	1204.779	1253.845	1492.751	1525.21	834.8308	1013.107	1064	787.7
2261	457.44	629	2053.47	1347.889	689.273	795.6993	2888.613	729.5858	2902.103	857.1	729.6
2262	458.34	125	2113.85	1996.621	3905.291	1216.863	1206.573	2775.916	981.54	2069	2208
2263	458.78	77	6720.9	2583.587	3527.223	2074.107	2921.353	1848	3005.933	8603	1897
2264	458.79	62	4988.94	1933.71	2431.963	1699.643	627.1891	1878.39	2602.88	2705	2632
2265	458.94	85	6451.6	9525.821	12707.5	3074.4	4876.728	3834.044	6409.979	7508	8081
2266	459.24	77	1411.18	1889.818	1589.591	2118.748	844.4333	1439.146	2016.933	1618	1278
2267	459.3	60	1267.49	1634.896	1309.777	1265.913	2150.325	2259.965	1383.624	1434	1725
2268	459.33	123	1415	1045.44	1986.092	2009.64	2052.697	861.9657	632.6622	1918	1368
2269	459.35	97	5133.18	3507.838	5121.882	5726.049	12554.25	9056.259	7654.197	7823	6335
2270	459.41	318	1048.38	660.6659	3080.791	3439.485	1482.302	745.2	1332.22	1960	1409
2271	459.41	209	1032.45	878.4132	1710.154	2044.888	3163.798	631.4831	1167.497	1106	1191
2272	459.41	293	802.1	524.5828	1508.597	1675.752	794.5945	659.9065	1117.238	914.3	757.8
2273	459.41	196	1550.28	689.5157	2641.209	4414.295	1602.5	1483.927	1493.26	2152	1563

2274	459.78	71	3896.49	3357.113	3192.064	1412.285	1497.6	3052.884	2263.615	2585	2881
2275	459.94	89	12149.9	8578.679	8747.344	2043.064	5145.84	6115.331	6946	8509	5821
2276	460.2	78	7762.88	2569.103	6980.654	5263.255	7222.168	5745.686	4637.056	3781	3533
2277	460.27	1043	3420.38	4558.075	3281.238	2280.378	3550.501	4484.361	3063.107	3900	5045
2278	460.27	79	243438	222529.6	247623.4	196626.5	326635.5	172666.4	210924.6	2E+05	1E+05
2279	460.31	64	4430.06	1684.222	3759.223	33179.37	2851.098	5055.921	1280.275	2966	2612
2280	460.36	98	1937.91	7813.975	1957.275	2259.258	2698.608	3929.805	1318.757	3269	2385
2281	460.64	70	1349.4	880.32	1238.72	904.3494	704.6	1231.802	1050.667	1611	987.3
2282	460.78	62	6552.21	2383.088	3218.922	1834.805	841.6088	2443.959	3221.057	3771	3598
2283	460.9	82	11031.9	12299.09	19306.36	7933.901	14508.07	8067.426	6853.438	10764	12999
2284	461.2	77	3078.93	1094.493	2570.583	1886.951	2058.322	3012.207	2229.426	2178	1345
2285	461.25	65	1034.42	997.4462	1470.368	1070.527	705.446	1312.875	1595.841	1742	1687
2286	461.27	79	72168.4	68518.17	68128.33	64245.47	101319	57822.06	64966.19	71192	39532
2287	461.35	99	4679.32	3643.034	5025.635	4674.344	1081.44	999.2206	1978.953	3974	1171
2288	461.78	69	5574.39	3846.791	3669.09	4147.6	2946.987	3974.287	2880.216	4975	6068
2289	462.23	97	1868.13	462.0897	698.19	1778.335	1443.556	583.8667	1958.746	982.8	1202
2290	462.27	79	14207.5	15444.04	15825.52	14834.98	21199.92	13914.66	14856.62	13823	6217
2291	462.4	135	1907.95	1287.073	2041.813	2252.965	2918.757	1893.584	1878.751	2359	1454
2292	462.64	72	4704.84	2748.318	3773.971	3334.734	968.4515	5398.277	3075.535	4155	6347
2293	462.78	62	3725.54	1524.78	1873.289	1063.077	930.215	1572.951	1824.953	2410	2239
2294	462.86	82	11063.3	6280.899	20357.3	8701.739	5162.505	7337.298	8284.495	7167	7508
2295	462.98	85	8825.47	5261.063	1978.505	2541.25	3558.486	3463.163	2544.03	1500	2775
2296	463.13	132	3169.33	1441.17	2129.311	1076.253	2700.403	1905.643	1071.759	1422	1343
2297	463.2	100	1432.17	671.58	1017.419	1622.715	1401.377	1296.415	810.4608	1247	1338
2298	463.27	79	3175.22	2667.95	2100.043	3165.302	4615.903	1778.34	2836.118	4144	1732
2299	463.34	134	1437.83	904.8556	1587.032	1082.653	1379.947	1443.279	1255.624	1764	1190
2300	463.77	71	2192.58	2410.254	2472.027	2854.087	1546.941	4291.25	1757.968	3831	2208
2301	464.13	132	2037.57	1032.51	1155.645	857.4706	986.6013	2292.975	536.76	782.3	1190
2302	464.31	171	3954.72	2348.145	6678.401	2224.764	3885.522	3084.835	2432.501	3082	3519
2303	464.33	1108	2358	1906.542	2271.713	1732.693	2384.969	2172	1878.038	2363	2382
2304	464.33	1080	1517.61	1604.444	1822.829	1881.4	1764.736	1681.231	1703.564	1864	2005
2305	464.33	97	1641.23	2167.011	3434.067	1843.163	3433.342	2939.272	1616.532	3618	1453
2306	464.36	123	2987.5	3689.873	4470.033	2841.486	2859.545	3535.003	1856.689	3660	3166
2307	464.37	473	1409.06	1378.872	4150.122	1496.967	1778.556	1615.771	1832.014	1991	2031
2308	464.37	2	603.073	677.2811	1111.148	482.733	557.6929	538.2328	824.6141	567.9	523.9
2309	464.37	211	2965.91	2431.029	2092.284	3161.505	3011.251	2864.752	1622.602	2022	2940
2310	464.37	392	2151.74	1710.297	2584.037	2137.536	2157.608	2605.537	2075.093	4020	2419
2311	464.37	50	2075.05	1097.618	1490.475	2491.76	1886.861	2284.147	2074.966	2087	3696
2312	464.37	1046	3268.45	3704.019	3959.334	2938.889	2066.822	5819.339	3703.888	3431	6272
2313	464.37	141	2293.8	1918.93	2166.637	2914.488	2333.688	2344.766	1954.601	1082	2592
2314	464.64	72	5147.18	3976.532	4810.344	2909.795	971.1533	5919.454	4151.557	4380	4726
2315	464.73	69	3984.51	1302.672	1092.776	1000.727	619.3856	478.3836	1507.275	2689	1356
2316	464.83	1107	1121.96	1339.524	3588.749	1145.253	1306.434	1235.054	1289.143	1455	1356
2317	464.86	83	2017.48	1285.488	2817.36	1791.72	1281.853	2107.733	3051.582	2051	1775
2318	464.91	62	2050.71	934.8967	1154.947	1098.533	349.7448	708.5833	881.8167	856.8	1366
2319	465.13	134	786.736	241.3	691.06	119.1114	634.74	349.3371	236.1543	326.4	221.8
2320	465.27	63	3286.23	1750.022	1900.854	729	1956.804	2464.782	1838	1323	1405
2321	465.77	67	987.852	1250.694	886.0909	2009.913	810.0144	658.368	740.328	832.1	657
2322	466.41	167	3901.92	2324.864	3363.865	2822.79	3592.153	3604.462	2555.401	5234	4560
2323	466.41	245	1411	881.6898	1363.642	1448.174	1557.027	1386.374	1111.868	1126	1254
2324	466.64	73	3100.5	2079.879	2549.32	1684.985	940.476	3986.94	1958.507	2460	2999
2325	466.73	63	2839.85	516.2063	1651.2	878.1862	215.4058	1062.928	681.7657	1424	1075
2326	466.73	76	3058.59	1052.474	2158.882	783.58	664.8967	1095.293	2274.412	2992	846
2327	467.14	75	3746.05	392.2208	1138.99	1384.152	554.688	560.5618	1260.33	1188	628.2
2328	467.18	82	3136.44	10018.64	4102.846	3069.656	2453.932	2192.18	2132.443	3971	2721
2329	467.28	209	5963.07	2120.936	5434.703	3844.427	4339.34	3021.529	4450.336	5293	2289
2330	467.46	300	1653.9	1920.106	1980.807	1097.595	1108.224	728.6467	1392.308	1534	1275
2331	468.2	80	2016.89	3684.943	4437.419	1731.84	2411.621	1698.891	2765.365	2455	943.7
2332	468.28	210	2020.63	820.44	3076.488	1019.69	1316.913	1030.244	1500.109	2069	972.7
2333	468.31	121	4537.7	847.53	4455.21	3680.24	3344.982	4454.286	3113.266	3309	5504
2334	468.39	92	2745.14	1183.58	1526.08	1422.72	2411.442	3892.28	2215.304	2710	1760
2335	468.39	1050	3090.97	5403.6	4914.294	4199.02	5452.148	6007.253	3379.874	3106	3621
2336	468.39	144	12883.5	11260.22	15798.19	13071.36	16182.75	12353.85	11727.85	15087	13975
2337	468.39	1104	3871.49	2324.501	3950.542	1305.656	3556.469	4101.831	3611.518	3814	4777
2338	468.39	1076	2479.02	2463.684	2518.156	2793.835	2820.563	2814.873	3012.243	3009	2921

2339	468.39	2	313.242	243.7077	323.6103	225.475	287.8327	394.1333	255.78	292.5	262.5
2340	468.72	74	4875.47	426.7677	1556.975	715	337.589	1383.839	1888.045	863.7	1421
2341	468.81	78	14847.2	4491.44	5877.923	2060.846	2600	4203.459	11297.7	11409	3109
2342	468.81	61	6375.51	2859.538	2502.6	2243.171	978.4093	2962.492	3189.424	3962	4150
2343	469.16	76	7781.07	1431.773	1747.85	1897.221	3384.32	1035.342	4803.694	3585	4133
2344	469.39	113	1156.37	1216.869	1571.714	1527.971	1787.932	2175.059	823.99	1465	2007
2345	469.39	144	4772.81	3810.079	6591.3	6360.642	4660.869	4351.813	3767.658	3548	4323
2346	470.36	100	878.882	647.0854	1006.679	1091.229	833.4667	556.5188	986.6583	788.3	798.8
2347	470.81	79	13112.2	4201.094	5581.289	2364.931	2420.56	3834.714	5633.722	8596	3416
2348	470.81	62	5553.87	482.784	2369.683	864.348	950.9104	2486.588	2855.303	3486	3684
2349	471.13	73	962.576	367.1137	668.4393	649.9792	989.3567	583.84	1354.769	1167	1485
2350	471.3	71	954.858	1164.401	797.3715	1350.483	1618.923	1994.72	882.4215	1195	1440
2351	471.75	77	4430.96	4486.171	2488.943	2052.012	1704.185	4672.148	3115.403	3763	4002
2352	472.27	61	2851.14	1622.575	1840.375	1074.824	806.6522	2806.956	1151.707	940	1024
2353	472.29	98	1864.7	1108.536	1655.382	2243.663	2614.59	2296.87	1769.534	2113	2626
2354	472.36	86	108201	10671.49	7692.976	46618.22	131444.5	6322.698	51032.24	50202	45515
2355	472.74	78	3408.43	2311.763	1711.301	2199.668	2103.04	2829.595	2668.247	2901	3948
2356	472.81	62	2571.25	1345.993	1226.675	1245.883	637.3004	1431.843	1300.122	1919	1776
2357	472.86	81	6134.42	1581.649	1559.242	1743.75	3083.22	2059.329	3488.111	3114	2036
2358	472.96	84	516.128	2261.421	512.0675	1801.463	512.0675	448.1875	766.2437	0	2060
2359	473.26	77	667118	1303173	509489.4	2052020	460914.8	3271.251	718556.8	7E+05	1E+06
2360	473.29	98	2570.58	3571.235	637.5	3567.965	1831.25	783.5433	1181.575	2063	1906
2361	473.3	61	1366.53	1243.394	1081.591	1220.893	1020.877	1735.044	1091.136	687.1	1001
2362	473.33	104	3891.48	4914.761	2056.86	4037.04	2498.633	2709.731	3195.029	1504	3141
2363	473.36	86	31911.7	2735.049	1878.772	1531.064	41010.47	1981.216	13930.92	16451	14180
2364	473.39	208	1251.77	339.6926	1800.448	1564.852	1237.141	564.6989	934.4114	1297	694.6
2365	473.96	86	10407.8	8503.333	16705.62	4184.713	255.78	8291.301	7284.399	7849	7932
2366	474.27	77	224813	429088.4	165389.2	664863.4	163439	397025.2	233421.1	2E+05	4E+05
2367	474.29	59	691.653	1059.08	931.645	654.1989	1831.808	1418.225	1090.805	1822	1547
2368	474.36	85	6755.89	255.78	0	3934.163	9552.015	1169.346	3303.635	2518	2499
2369	474.76	62	1124.38	650.16	1964.726	640.0299	347.7635	1585.637	1177.875	1348	1690
2370	474.76	76	3294.66	1314.121	2065.255	2851.984	736.1618	2212.353	2279.036	4373	2105
2371	474.93	84	4103.95	3040.82	2758.443	2609.968	1793.458	2409.19	1491.997	3410	2019
2372	475.27	78	44541.3	84452.73	36147.07	125069.8	29279.79	64005.67	49273.67	51436	66947
2373	475.3	122	4862.02	2293.434	4001.55	3108.162	3223.579	5180.011	3478.924	4015	4307
2374	475.32	97	3172.05	2893.651	3924.72	1802.4	5262.908	4098.86	4032.666	3778	4235
2375	475.33	95	3487.28	4126.833	2924.85	1297.8	4548.982	5831.229	24311.42	2110	7559
2376	475.75	69	1268.8	962.8218	1524.243	1513.952	991.3278	1087.191	1006.749	1610	1070
2377	475.83	97	239.411	1529.775	1766.972	389.4818	1636.903	1129.303	1566.18	1381	873.7
2378	475.87	22	1242.78	2104.625	961.0759	1824.508	1520.946	1699.783	718.5523	1325	2385
2379	475.87	42	1483.7	1273.873	1469.045	1170.693	1312.254	2223.313	1256.502	1596	1356
2380	475.87	160	1657.92	2139.082	1883.555	1555.083	2505.987	1668.212	1942.51	1694	1611
2381	475.87	1077	3427.19	11040.21	4459.964	4483.661	4527.69	3773.714	3392.34	3799	4338
2382	475.87	1021	9844.92	7234.626	7000.349	7291.588	5872.455	7453.284	7561.697	7697	8411
2383	475.87	234	1740.4	1488.426	1477.728	1682.86	3494.171	1243.862	1387.238	1718	1728
2384	475.87	1109	4449.4	5125.526	5336.568	4372.395	5745.788	4573.487	3887.094	13166	5516
2385	475.87	214	1923.03	1236.289	1648.015	1557.487	1250.239	1436.021	1291.489	1656	1432
2386	475.87	125	966.507	832.5257	845.288	1268.312	1670.823	1602.207	901.269	1485	1357
2387	476.27	78	9730.7	15744.98	8191.63	15655.05	7354.207	11363.94	10481.08	9794	12350
2388	476.3	469	1690.5	1473.027	2917.709	2258.064	2582.411	2137.267	2293.527	2666	1375
2389	476.3	61	11597.1	8227.352	9659.393	5669.669	9054.113	15497.95	8506.236	8952	8797
2390	476.3	122	5599.85	3367.97	6272.49	3419.327	2756.29	5104.575	1045.8	4605	4454
2391	476.32	97	1092.28	2084.059	2049.288	3118.725	2105.425	2153.048	1890.543	903.4	3369
2392	476.37	1073	2401.35	4356.684	2787.161	2566.251	3040.29	2137.266	2712.672	2314	3152
2393	476.37	1109	7470.62	4144.585	3150.223	2920.395	3104.916	7089.133	2256.811	7889	3534
2394	476.37	1022	5672.81	3790.877	4251.759	888.515	3615.314	4514.1	4319.936	4640	4659
2395	476.4	27	489.036	1132.661	597.6322	958.902	831.947	767.4265	4776.87	567.3	1126
2396	476.75	64	5433.78	919.4963	3830.359	900.6537	1190.037	2051.436	2117.026	4489	1829
2397	476.87	82	1240.88	1717.691	3341.355	256.1183	1537.972	2318.745	1507.356	1491	2024
2398	477.23	62	3593.27	1191.404	1838.48	569.3494	812.1959	2725.666	1031.248	1689	1685
2399	477.27	77	3907.88	2342.747	1751.956	6630.387	5408.166	3192.703	1885.953	2511	4048
2400	477.31	61	3575.79	2381.236	2725.811	3016.762	2946.663	3929.595	2104.443	2607	3145
2401	477.35	120	1847.59	684.4735	1664.276	1775.821	1360.124	1643.595	2450.647	1646	2355
2402	477.75	72	1749.15	1900.547	2160.749	1966.763	1011.22	3116.722	1713.465	1721	2202
2403	478.23	91	2886.58	2682.743	932.3015	1765.28	1969.38	3274.125	2700.933	1910	2558

2404	478.35	116	2077.92	3152.767	2530.484	1998.359	2669.424	4552.954	1878.465	1941	2823
2405	478.41	103	2526.12	2050.76	2085.171	2194.92	1738.373	1344.105	1132.603	1733	1597
2406	478.75	76	2373.4	7888.122	2307.688	4445.678	5285.46	2521.48	7186.876	9634	6647
2407	478.84	78	16817.1	7838.556	11933.33	6071.594	5487.387	7947.824	12148.1	18265	8837
2408	478.84	61	7442.33	4106.363	3051.926	2278.826	1041.313	3371.697	3581.12	4035	5637
2409	478.85	115	715.68	351.9525	1070.112	832.86	1461.931	1019.126	653.716	545.2	969.6
2410	479.24	76	1550.57	1595.932	2424.78	1033.78	863.6355	1422.392	2115.551	1525	2486
2411	479.74	72	2590.09	1830.245	1761.838	1347.323	527.5346	3045.69	1277.218	1252	2622
2412	480.31	147	5232.6	3851.305	8899.96	3315.983	4601.16	6339.93	3468.755	5987	3076
2413	480.42	202	1612.41	1590.446	2620.8	1615.795	2671.26	1935.349	2107.295	2276	1548
2414	480.61	72	1002.3	448.074	953.1675	294.5784	277.986	2012.149	728.2833	1081	1547
2415	480.84	61	5371.32	2529.804	2032.935	1842.816	520.04	2225.25	2459.402	2465	3785
2416	480.84	80	6709.18	4079.894	11509.91	2821.832	5275.109	4459.35	8586.24	11258	3897
2417	481.26	103	1833.1	2806.325	1706.286	1536.183	2029.08	1749.444	1058.101	919.7	1910
2418	481.26	62	7612.93	4144.847	5905.779	2474.782	3847.803	6683.458	3734.029	5222	4974
2419	481.31	142	1787.5	1407.454	2237.488	1097.628	1377.599	1571.076	1578.736	1403	1926
2420	482.18	77	2812.87	550.407	1781.003	694.2643	1068.969	1431.828	1984.613	1589	899.7
2421	482.26	62	2041.04	1528.626	1247.262	967.4359	874.3964	2018.437	1073.766	1474	1119
2422	482.32	210	17159	4167.24	22444.84	3506.777	5415.781	6322.161	5146.449	10025	3509
2423	482.36	259	2275.56	1387.51	7412.389	1588.212	3709.184	3973.09	1893.392	2259	2712
2424	482.4	1	740.207	494.6627	455.6917	472.851	636.585	523.1448	765.802	685.6	494.1
2425	482.4	1172	2034.21	1793.677	1706.425	1433.263	1718.36	1796.464	1533.844	1995	1670
2426	482.4	1194	2727.21	2619.155	2622.71	2176.64	0	0	0	3036	0
2427	482.4	1073	3322.01	3225.974	3680.933	3153.613	2900.525	3484.404	3267.398	3084	2935
2428	482.4	1135	4036.45	3790.065	3470.886	2395.68	4122.257	1506.99	4017.22	2955	3728
2429	482.4	1100	2787.33	1994.12	2807.585	2570.859	2535.33	3175.518	2900.979	2791	3017
2430	482.4	166	11469.8	12712.37	9622.688	9925.25	12968.27	11272.53	8855.901	12242	15043
2431	482.4	1050	3654.13	4918.613	4410.456	3723.672	8519.517	3699.628	4774.56	9276	3502
2432	482.4	121	4535.52	3371.853	8395.484	3060.568	4706.142	5740.344	2184.943	6182	4775
2433	482.43	97	635.144	1141.022	970.7495	1075	1218.007	838.75	681.36	905.4	1487
2434	482.83	62	1558.71	1462.457	863.6286	850.0149	351.5644	768.936	844.335	1213	1991
2435	482.84	80	2932.93	2079.306	2166.711	1553.06	1317.555	1843.344	2863.39	2867	1041
2436	483.25	79	2401.12	5324.148	3008.416	4073.625	2581.644	2176.292	2973.089	2883	1054
2437	483.27	107	1319.49	1103.836	1589.18	964.4464	1748.565	1190.695	895.4538	1150	1118
2438	483.27	139	2480.81	2889.316	2494.562	760.326	3111.263	1700.254	2556.217	3236	3617
2439	483.33	210	3978.43	2112.07	6839.471	1590.531	2324.132	2281.325	2362.563	2721	2466
2440	483.41	166	3866.28	2937.487	2913.887	3347.53	4010.696	3814.319	2675.917	3802	5201
2441	483.73	118	2074.17	1066.02	1640.285	635.544	497.952	2577.968	776.16	841.3	1268
2442	484.05	92	4375.65	831.5382	8087.66	253.344	754.2	1694.809	1524.825	2130	1766
2443	484.34	218	1428.8	974.9693	1398.026	1350.505	1557.697	1345.257	1016.846	1230	1253
2444	484.38	112	12549.7	7804.507	15083.42	9574.695	17535.9	11777.95	7402.828	11610	12565
2445	484.38	1072	2160.73	1771.364	1720.142	2663.223	2139.531	1975.05	2262.994	3619	2174
2446	484.47	92	5415.68	7877.792	70.43556	5180.555	3293.093	4719.608	0	31.49	4784
2447	484.47	52	5380.77	3211.946	192.1053	4822.183	2338.402	4650.888	100.8755	186.6	3664
2448	484.75	65	1010.41	947.44	937.878	933.39	478.86	1035.726	1232.298	1762	1424
2449	484.79	78	4688.8	2128.683	3354.018	1079.977	1207.14	2880.188	4110.204	5340	2600
2450	484.79	63	4929.28	1116.252	1455	1122.786	501.8027	944.5229	1132.693	1597	1229
2451	485.11	131	3095.35	616.4288	1249.107	812.63	1619.7	1999.648	398.475	656.7	1357
2452	485.29	119	454345	129193.3	402941.8	110280.4	133240.5	241536	142898.6	2E+05	3E+05
2453	485.36	81	6948.75	4427.736	5821.643	9398.9	10488.74	9476.758	7086.99	7319	6845
2454	485.36	58	5828.05	1683.423	3545.542	3751.607	2983.24	4483.025	2693.348	2901	3817
2455	485.39	112	4178.89	3395.603	6363.761	2888.96	7159.633	4257.74	3948.216	4081	4074
2456	485.47	53	1802.56	970.5157	60.73071	910.4764	811.9138	1325.442	89.4948	66.54	1089
2457	485.47	69	780	1336.404	0	1389.797	643.995	1338.167	27.594	35.05	1883
2458	486.11	131	1294.19	313.158	960.6933	609.1209	600.71	964.7711	53.03455	318.4	528.8
2459	486.29	119	164120	47993.29	163771.3	35118.46	46665.4	87897.55	49453.03	59532	1E+05
2460	486.36	79	1077.99	1091.306	2677.4	4442.212	2846.461	2652.476	4276.86	4404	2867
2461	486.38	122	2532.23	921.5344	2981.589	1814.617	1002.091	1698.577	1853.232	2688	1307
2462	486.66	69	9592.26	6950.626	5468.976	5090.198	3918.2	8126.736	4117.905	8145	7683
2463	487.3	115	67405	51346.85	65049.98	47086.76	57900.16	31187.69	20641.88	28606	33287
2464	488.3	78	26476.3	12855.82	15055.93	15395.55	11890.16	9060.48	17175.38	11508	1407
2465	488.3	111	29433.3	26027.32	20137.87	14910.84	18774.21	6786.8	5965.522	4129	5604
2466	488.32	1046	1062.29	872.5294	857.7563	925.739	782.351	1134.407	856.7333	1011	1478
2467	488.33	80	20114.1	11728.82	13794.78	11713.35	11433.87	2757.882	8588.811	11087	7141
2468	488.65	70	21137.1	13762.78	12944.38	13625.11	8719.938	16113.59	16224	19115	17010

2469	488.87	81	39633.3	33572.02	42243.15	17883.62	32014.98	20235.52	25074.38	37530	29844
2470	489.26	69	3303.87	3551.042	1234.754	4884.012	5156.427	2444.386	1588.067	2104	3040
2471	489.3	80	8800.64	5434.378	3952.841	4144.939	5043.608	3563.536	3767.913	4674	6310
2472	489.34	111	2567.26	4411.307	3120.108	987.84	5325.788	1987.838	1746.59	1279	2020
2473	489.87	81	3259.79	2526.005	5577.483	2560.806	4201.117	3170.749	5088.24	3598	3179
2474	490.26	68	2070.84	1592.303	1211.112	2232.457	1876.452	2177.01	522.972	1192	1617
2475	490.65	71	24177.6	16337.02	13855.81	13596.19	9695.8	19383.76	17357.81	20595	22689
2476	490.87	81	13996.4	13431.89	16524.24	9445.342	14167.49	9148.445	6736.796	15005	11627
2477	492.32	122	2469.24	1416.482	3126.406	2742.04	3364.212	4285.233	2741.569	2309	2678
2478	492.4	1169	1912	1196.936	528.1983	1010.43	1010.133	1312.215	442.5862	484.2	1172
2479	492.46	1	0	143.84	487.229	86.74754	0	168.966	884.225	1197	85.86
2480	492.46	182	3840.35	1928.833	220.9716	2885.955	4596.875	4228.879	169.786	130.8	3545
2481	492.65	70	17751.9	9921.37	10025.45	9726.42	6965.4	11755.25	10289.71	15542	12764
2482	492.86	61	1107.6	884.6853	916.4667	925.76	287.8847	1027.333	901.0589	1006	1606
2483	492.86	79	3838.89	2904.637	3920.56	2390.365	1551.118	1526.953	2961.306	4554	1776
2484	492.88	94	4093.23	2472.027	2650.31	2281.953	3646.706	2747.138	1069.208	2867	1614
2485	493.24	68	1634.94	1354.699	880.27	1064.782	1288.596	1054.078	896.3733	1196	1308
2486	493.28	1046	2464.22	2379.73	2383.166	1702.566	2126.935	2916.826	2718.267	2333	2952
2487	493.35	1074	1372.69	1289.57	1299.22	1574.049	1354.154	1417.243	1633.125	2423	1126
2488	493.35	109	2091.22	1015.295	2846.34	1064.096	2387.823	1986.85	1409.639	1752	797.6
2489	494.32	130	5789.93	2031.772	6511.734	3067.392	4861.287	10796.19	3880.006	7833	7538
2490	494.4	87	58035.4	6386.813	2566.23	31180	101996.4	4093.042	28759.25	26480	31273
2491	494.64	70	6703.62	4988.935	4385.825	4129.308	2738.941	5164.528	5260.846	6364	6207
2492	494.82	78	7670.93	4848.613	8624.458	3976.2	1834.483	3274.556	8878.005	3317	4138
2493	494.85	62	1422.99	1240.907	924.888	916.3901	1223.394	1527.151	1561.976	1780	1196
2494	495	65	1651.26	269.1	414.4	766.1576	2140.446	91.68763	853.3956	973	833.9
2495	495.15	77	12280.7	9318.311	4928.931	18049.25	2601.585	12326.4	11870.81	11048	9868
2496	495.34	103	922.278	948.7017	1113.84	854.28	1916.845	1348.528	1156.778	1037	881.5
2497	495.41	86	20029.3	1290.635	1871.011	13856.25	36081.45	1815.494	11926.95	10354	13076
2498	496.15	77	3997.26	3241.904	1509.084	5458.04	721.7875	3917.593	3059.539	3861	4300
2499	496.34	177	20549	13338.18	27455.56	6897.54	22808.68	19007.04	9830.438	15234	23560
2500	496.41	88	7264.26	1949.62	1972.457	3457.931	9960.825	8098.492	3560.801	4626	3558
2501	496.42	142	7330	2653.04	3805.217	3871.793	4147.671	4027.786	4307.632	5343	6621
2502	496.42	115	2909.11	2355.269	1420.371	2238.183	2781.21	3574.441	2460.005	2931	2669
2503	496.42	201	8612.51	7221.451	9117.638	8563.194	4119.538	6190.184	4973.834	9977	9648
2504	496.42	1106	2457.07	2731.133	3067.193	2241.734	1897.5	2802.07	2677.675	2815	3538
2505	496.42	1050	2566.3	2026.551	2350.334	2387.291	5140.001	4723.651	3045.625	2431	3236
2506	496.42	1171	4717.63	3643.383	3252.037	2796.281	4576.82	3965.821	977.9	4362	3447
2507	496.65	68	3151.23	1355.192	1170.992	614.9473	1066.103	1592.85	1036.549	1383	1627
2508	496.81	79	6329.35	3866.79	4209.054	1562.956	1926.531	2579.5	4102.039	5729	3221
2509	496.85	66	1139.8	1254.863	2109.794	724.5609	1722.882	988.425	1854.524	2069	1383
2510	497.15	78	5287.78	3223.808	2707.758	6800.88	1804.603	5266.715	1298.222	5131	3233
2511	497.23	62	5301.28	2316.137	3332.694	1532.558	1749.732	3740.403	2322.298	2996	2825
2512	497.34	179	6388.74	3889.455	11513.23	2014.41	7515.88	12095.8	2551.456	4531	6854
2513	497.42	200	4230.24	1968.305	2407.844	2157.893	1834.473	2531.915	2069.268	4326	3815
2514	497.42	143	1903.25	1452.714	2376.096	2101.762	1627.812	2925.967	1961.387	902.7	2049
2515	497.43	1176	3111.79	2347.437	3977.961	1610.952	2609.56	2836.305	1095.375	3282	2434
2516	497.43	1048	1119.12	1433.114	1427.09	1340.726	1435.503	1412.978	1909.029	1484	1677
2517	497.43	1072	1309.58	1421.7	1350.773	1183.379	1088.278	1467.871	1418.75	1249	1641
2518	497.43	114	2290.15	877.92	1063.208	1666.28	653.7745	686.4709	1565.933	1457	1311
2519	498.15	77	3199.33	1944.197	1348.259	1984.02	1108.56	1521.6	2282.16	3086	1995
2520	498.26	78	991.97	1032.646	451.7748	1364.311	1071.036	1798.672	1936.541	1398	1336
2521	498.4	122	15210.3	6292	13638.75	10582.8	10260.81	16876.88	7987.951	10484	15370
2522	498.4	1075	2598.47	2786.974	1634.753	2038.047	4359.318	3375.988	1953.402	1294	2335
2523	498.68	67	3957.72	889.386	2831.816	481.9965	1277.9	2111.839	2764.604	3894	1584
2524	498.8	161	1263.55	595.9347	1142.672	1058.243	1205.428	1222.977	835.8924	1284	1089
2525	498.81	77	1624.62	1443.276	1930.047	1892.616	713.5789	902.652	2410.66	2240	1113
2526	498.85	66	3838.61	3354.317	3807.352	1961.957	7861.886	2030.691	2245.331	2833	3181
2527	498.9	85	132576	154320.7	153646.7	74003.34	146890.4	87772.55	120765.9	1E+05	1E+05
2528	499.11	79	6542.24	8352.953	6074.549	4094.714	4024.504	3279.355	6027.385	6214	4328
2529	499.26	78	2074.6	4018.16	2680.129	5474.912	3830.609	7360.184	3732.466	4688	1558
2530	499.29	122	646.04	1387.156	780.7744	602.3567	240.4478	1728.48	2422.335	339.4	503.3
2531	499.4	123	5855	2511.317	3911.396	4007.164	3349.513	6298.818	681.7229	5604	3899
2532	499.9	85	16076.7	20603.22	17385.75	5599.854	17951.63	12303.52	8612.721	13901	16168
2533	500.12	79	2872.56	3045.525	2650.014	1803.144	1113.819	515.5333	2495.004	1455	259.3

2534	500.27	78	1425.91	2583.519	1814.738	2708.483	1630.2	4443.073	1753.931	2423	1075
2535	500.36	115	3032.51	1808.8	1671.736	630.8698	1442.065	2812.464	1576.666	2318	2637
2536	500.68	65	887.49	1269.432	2852.284	897.1386	1077.7	1979.904	2670.087	4464	1507
2537	500.7	120	2236.19	295.68	1417.02	693.6166	302.016	431.635	421.68	0	572.9
2538	500.9	85	5524.2	5721.653	6802.934	4860.053	7668.141	4827.389	6070.761	5021	3648
2539	501.11	78	3618.07	2566.031	2246.399	1551.143	3735.685	776.5511	3676.434	1769	1562
2540	501.28	108	3564.06	3828.358	5380.011	3189.06	5301.831	4169.233	3334.789	5507	2931
2541	501.29	77	1524.4	2428.214	2016.422	2408.936	3234.838	2609.21	1457.539	1896	2536
2542	501.36	114	915.212	528.99	945.4278	902.1747	1418.677	1174.764	631.516	1006	708.8
2543	502.28	78	7060.63	10768.01	7010.734	7896.853	11214.27	5231.621	7243.894	6728	4302
2544	502.31	118	291919	84765.31	258896.3	66782.4	83883.36	141230.9	85582.77	1E+05	2E+05
2545	502.34	254	1358.33	865.8072	1305.983	1068.838	937.7732	983.0854	1351.93	1099	935.8
2546	502.37	125	2817.49	1295.132	2279.975	2290.093	2869.32	2003.029	2887.92	842.1	2740
2547	502.63	72	7709.46	4654.501	4187.669	4927.21	2652	5655.652	2298.323	6417	5548
2548	502.89	82	8876.66	9599.253	10797.86	5107.093	10347.29	4275.564	5552.838	9188	7693
2549	503.26	154	6490.07	2308.435	6454.53	1209.27	2794.251	3778.656	3282.975	4035	3039
2550	503.31	78	3717.78	9329.096	3139.877	3189.121	11228.8	4626.354	7465.142	5932	5800
2551	503.32	119	100850	27710.99	93926.76	23100.08	27590.24	50103.46	31660.46	41219	62110
2552	503.96	87	7179.15	7512.438	12497.65	2556.06	3800.418	3302.331	7140.44	6283	5930
2553	504.26	156	2470.47	902.2403	2258.815	913.0652	892.5714	1335.4	1430.056	1423	1374
2554	504.3	77	3218.59	3829.369	1518.834	3349.263	5037.773	3857.875	1518.56	1118	4639
2555	504.32	1049	1475.8	1296.05	1509.023	1240.059	1506.297	1083.022	1060.025	1176	1056
2556	504.32	111	90078.2	114415.8	119612.4	95728.18	124197	45531.57	33081.59	44187	48495
2557	504.34	63	2062.36	4497.198	2163.397	893.9433	2990.128	3120.987	2211.338	1866	2257
2558	504.36	141	2399.41	1602.047	2184.115	1336.588	1420.561	1968.846	1586.679	1910	2604
2559	504.36	164	1482.36	1349.364	853.0284	1614.496	1290.55	1595.253	668.0463	811.9	2876
2560	504.63	71	17955.5	9648.13	10417.86	10470.81	5577.38	13803.05	10819.9	13041	13997
2561	504.85	81	16220	10172.8	18976.28	6916.14	10020.18	11024.5	11664.47	13603	8939
2562	504.88	83	4002.98	3056.143	2177.62	3343.395	4805.475	1637.137	6495.275	3277	3038
2563	505.18	80	21073.8	21259.73	24539.35	16592.56	26477.77	9780.517	16364.5	16382	13064
2564	505.25	140	3181.72	3841.015	2120.292	1965.779	4852.162	2474.375	3187.885	2565	3185
2565	505.25	154	3893.81	1686.183	3148.7	1354.993	1887.204	2697.113	2059.99	2657	2393
2566	505.33	111	33916.3	36707.02	40591.32	36445.05	44107.46	14681.4	13775.07	14128	15076
2567	505.65	119	2151.94	639.18	2056.009	711.711	339.6373	680.3014	291.5467	184.8	1413
2568	506.18	80	5508.44	7456.383	8766.606	4105.463	8887.418	3508.719	3749.147	5822	4536
2569	506.26	156	1276.08	858.2964	1008.608	891.48	743.6381	1010.792	868.1136	1136	1764
2570	506.33	113	8292.88	8884.432	8794.048	5246.113	9399.282	1832.373	1950.738	1535	1499
2571	506.62	71	19488.9	12545.39	12544.82	10756.38	6547.933	17855.71	12394.2	15140	17069
2572	506.84	81	8788.31	2482.354	10633.28	3344.596	2840.376	3860.984	5322.053	7896	5364
2573	507.23	1048	1872.37	1930.762	1801.222	1496.629	1763.521	2329.691	2223.647	1873	2496
2574	507.27	118	255266	110122	229634	71157.33	111706.5	177423.9	88497.44	1E+05	2E+05
2575	507.33	112	680	2539.788	2239.047	1475.46	1816.52	93737.03	54107.64	52655	1E+05
2576	507.36	141	1113.36	767.08	999.8729	1522.533	1443.575	959.094	1200.167	1225	930.8
2577	508.19	1098	1523.87	1168.295	1411.5	1027.85	1080.782	1482.42	1296.035	1677	1627
2578	508.19	134	3802.18	5418.131	2608.479	1748.859	3729.276	2861.209	1294.546	1308	2398
2579	508.27	118	92470.5	36659.65	78246.38	24955.91	35588.94	59529.59	28179.62	48895	67314
2580	508.37	262	1708.57	870.5269	4218.91	1057.944	1726.416	2226.183	1302.691	1404	2114
2581	508.62	71	12676.2	7995.697	8744.275	7062.232	4215.492	12309.92	7638.165	10772	11838
2582	508.71	63	4173.69	957.2942	4329.173	794.1017	1392.033	1729.583	2931.224	2506	1900
2583	509.19	132	2053.11	695.058	1296.656	1007.814	1451.378	1602.752	599.45	791	1138
2584	509.28	115	37275	25997.69	35870.2	20797.93	25993.94	19084.72	9682.808	18667	20969
2585	509.29	63	2141.02	1799.903	2110.278	1027.204	1552.259	1330.526	1725.304	1880	1788
2586	509.31	138	1439.55	1905.494	1861.106	1629.948	1631.95	2760.833	1822.424	2606	839.1
2587	509.92	84	7558.16	9621.408	14769.01	3406.065	7530.781	7133.756	5756.061	6226	8026
2588	510.18	132	1144.9	858.9163	918.511	737.87	1598.886	818.455	185.955	615.4	706.2
2589	510.28	113	10823.4	8489.68	11727.95	6754.018	8731.088	6528	2674.26	4183	5848
2590	510.31	1046	1177.65	1145.088	1065.475	859.9875	880.8765	1283.211	1340.859	1110	1422
2591	510.4	1166	1219.75	1287.428	1228.717	1154.888	1169.447	1327.014	1153.974	1392	1362
2592	510.43	242	1148.87	885.7647	1465.332	1468.778	2023.714	1498.332	1211.153	1360	1439
2593	510.44	165	2614.26	2376.713	2233.947	1973.017	2293.652	2318.396	1987.401	2883	2902
2594	510.62	71	6103.07	3601.34	4146.877	3403.018	2181.021	5961.398	3175.934	4327	5042
2595	510.83	63	1355.92	899.5956	2076.84	423.82	891.1442	282.8675	809.1	846	719.8
2596	511.13	77	2882.05	2246.592	1139.574	2749.793	1906.788	3053.531	2256.361	1663	2318
2597	511.28	115	5777.99	3173.118	3889.877	3228.365	3583.043	2511.261	1439.09	1092	2241
2598	512.29	78	820.286	129.583	3013.674	839.2186	550.4486	1102.097	1645.838	2467	338.5

2599	512.34	177	7375.51	4810.124	4686.358	2512.492	4090.489	7152.055	4251.133	4909	3597
2600	512.34	209	2830.2	1576.211	2744.599	1680.992	2618.014	1736.285	2138.14	2713	1803
2601	512.34	157	1759.91	936.2168	1513.89	941.2	1194.244	1426.425	822.5864	1209	1257
2602	512.41	110	2376.35	1789.427	2992.164	2836.26	1571.324	2632.158	2817.634	1120	3918
2603	512.41	1052	3773.54	5177.467	3099.113	2679.318	4399.645	3072.074	2793.768	4989	2782
2604	512.41	144	13826.3	11756.06	21963.1	13815.72	15975.51	13427.58	12867.55	12914	14505
2605	512.41	1104	3883.33	4512.25	3548.138	3273.502	3248.263	3878.006	3783.24	3689	5897
2606	512.5	2	442.74	798.2912	391.0836	271.7155	825.5342	1183.071	210.156	230.7	131.5
2607	512.5	850	67.2705	54.69975	1658.076	52.35263	51.03779	48.84547	1740.19	1390	70.11
2608	512.5	775	152.355	70.29176	963.2209	82.53167	117.8728	101.9328	1707.058	1582	129.6
2609	512.5	743	80.3895	56.42	240.7731	74.32229	62.70147	51.633	534.3901	576	62.67
2610	512.5	1198	18710.5	28439.76	527.156	13342.58	24759.31	0	0	419.3	21108
2611	512.5	1045	1075.9	1090.695	14502.18	877.944	927.9867	927.525	15126.5	11541	1064
2612	512.5	1174	38572.4	15248.04	637.7725	9211.179	7933.924	32807.13	678.16	723.2	16577
2613	512.62	70	1793.43	778.2588	1139.773	716.9061	677.3521	2207.304	1039.374	1714	1373
2614	512.71	69	2179.85	881.55	1143.608	397.5705	628.7967	810.4688	1092.18	3456	686.9
2615	512.91	85	31513.6	41462.5	40191.44	24554.22	45179.52	21106.54	24710.52	30663	34406
2616	513.34	210	1153.79	701.9022	1071.063	697.7778	1423.316	879.5541	856.2906	1175	736.3
2617	513.34	179	2280.77	1598.77	1637.165	1422.153	2312.104	1609.475	1473.86	2261	1458
2618	513.35	1048	1008.61	1344.262	1488.96	1203.76	1379.696	1438.067	1530.528	1124	1494
2619	513.42	144	4767.84	3133.468	4707.36	6354.766	5665.332	4340.622	2526.376	4547	4515
2620	513.5	1045	474.696	481.935	5226.65	429.552	373.9333	419.6833	5618.557	4515	484.2
2621	513.5	1195	6779.91	9866.57	214.802	4612.567	9244.871	0	0	230.5	7876
2622	513.51	1175	14024.7	5572.5	327.7325	4343.949	2549.533	9082.846	253.37	297.5	5715
2623	513.51	1	821.453	256.003	0	33.075	255.84	478.72	91.306	78.28	41.55
2624	513.92	85	2066.29	4822.667	6157.673	3830.8	4101.762	2390.858	2248.018	5040	4458
2625	514.32	79	2995.81	4109.326	4134.664	4841.184	5148.177	3383.304	5431.506	5121	1603
2626	514.35	156	3474.78	1171.052	1211.934	1300.221	1447.232	2122.068	1664.15	1453	1444
2627	514.35	143	2290.52	1039.248	778.68	2212.801	2496.65	2392.726	1146.737	2395	2342
2628	514.51	1198	690.078	1997.888	191.352	1106.243	1797.333	0	0	172.3	1631
2629	514.51	1175	2349.1	1242.247	295.7126	940.2133	625.1331	2062.421	279.9409	332	1238
2630	514.66	66	3488.67	1188.46	2465.864	433.9796	863.5281	1086.677	1530.491	2965	1092
2631	514.83	63	1203.43	426.36	620.922	487.198	330.2254	448.3934	541.99	702.9	1002
2632	514.87	84	35741.2	34537.06	56316.95	22943.73	34365.53	27723.42	33305.05	29776	32472
2633	515.27	79	1891.88	2638.555	2408.324	1694.672	2255.134	1465.942	2370.902	2336	1831
2634	515.88	83	5625.62	7868.205	4884.096	4937.355	3551.451	3500.298	4039.56	4411	3771
2635	515.97	87	7887.35	3920.45	7441.375	1211.25	2749.705	2399.79	1981.016	3575	3916
2636	516.3	61	2215.48	1129.198	1499.178	859.2996	1080.085	1783.657	1234.685	1131	1214
2637	516.32	119	5021.17	2753.28	5606.858	1782.289	1781.035	3527.432	2696.807	2805	4103
2638	516.38	86	46837.6	4168.866	3424.933	19925.46	59700.17	2901.725	21781.23	21271	15385
2639	516.74	64	4736.84	1368	1401.633	735.2354	945.672	1276.63	1480.187	3916	693.1
2640	516.87	83	3724.03	6565.085	8909.111	2554.803	3330.18	5417.17	3782.88	4735	3507
2641	516.96	87	8496.44	501.942	1661.818	1774.22	9209.621	974.1338	2203.076	3096	2460
2642	517.35	1198	498.111	1305.906	1138.992	542.035	0	0	0	802.3	888.7
2643	517.35	2	692.449	231.472	312.4763	150.1	249.1065	447.3313	637.856	1216	168.1
2644	517.35	1174	2358.19	1707.48	1732.463	1406.875	1112.611	2210.728	827.1467	2100	2033
2645	517.35	1053	5124.75	2839.916	1749.15	2856.286	3916.08	3466.232	1380.089	3770	3181
2646	517.35	48	1248.42	2704.955	1697.879	3014.751	2088.931	1702.871	642.1322	629.9	4771
2647	517.35	199	964.586	970.4814	825.4309	1174.162	1087.571	965.2742	655.9195	937.9	1188
2648	517.35	103	1437.28	1379.161	2558.296	1131.616	1497.188	1343.504	1808.235	1312	1475
2649	517.35	143	1281.61	1619.268	1959.143	1475.339	3399.632	1651.101	1728.268	1443	1824
2650	517.39	85	18022.3	1291.905	1053.976	8809.575	21981.55	1117.846	8119.291	8380	9657
2651	517.73	69	1786.8	1500.124	1673.727	983.664	1213.553	1658.24	1059.188	1643	1257
2652	518.26	77	2985.07	11726.69	2925.21	3250.985	1004.366	4229.867	2204.164	4299	3215
2653	518.31	107	2149.68	1664.742	3151.642	1829.52	1657.283	1719.303	1304.72	1938	2436
2654	518.32	173	5232.34	1737.552	3325.769	1123.173	2149.325	2288.627	1682.411	2070	3942
2655	518.33	71	3370.46	5040.599	2175.307	5403.299	4365.727	4781.031	3508.544	3796	5067
2656	518.37	460	1354.13	1294.529	2118.538	1480.485	2234.467	1376.181	1492.527	1942	1599
2657	518.65	70	1750.23	1830.595	1927.823	1054.991	679.4642	1188.052	1210.023	1774	2243
2658	518.86	80	4176.67	2324.405	4359.015	2055.987	2136.911	2471.628	2264.592	3269	3368
2659	519.26	76	1139.07	5593.968	1425.395	1841.374	1644.338	2524.42	1231.888	2202	1928
2660	519.33	70	766.541	1941.988	935.2548	1062.082	1171.848	1759.515	637.97	1719	1505
2661	519.73	70	4046.06	3221.396	2067.199	3546.245	1689.053	3834.934	2088.311	3475	3701
2662	520.28	154	3490.74	1089.44	3600.811	1597.539	1398.896	2051.592	1681.978	2962	1702
2663	520.29	77	1563.01	7391.532	2386.015	5908.892	3259.153	4277.004	2565.309	1287	4910

2664	520.33	61	7766.86	3447.529	6657.908	4858.805	6217.406	9128.59	5823.228	7114	6680
2665	520.35	122	2458.93	2010.948	3626.151	2460.309	2140.785	3766.982	1759.362	3490	2164
2666	520.51	140	3446.34	3701.155	39.37119	439.2615	3588.853	2813.773	84.66023	151.3	639
2667	520.6	71	6348.68	3811.19	4988.042	3103.55	1210.416	6469.18	3022.38	4191	5906
2668	520.74	64	4782.06	1006.023	2786.759	818.7847	980.1959	1656.902	3035.554	4964	1965
2669	520.82	81	3546.82	2061.443	2301.659	1795.826	977.416	1941.308	2291.995	2594	2844
2670	521.25	115	2490.51	3285.2	3724.76	1997.125	2310.4	2564.771	2961.679	1103	2940
2671	521.26	62	2364.8	2937.135	1748.753	3445.03	4950.362	2370.46	3997.878	4312	3835
2672	521.29	155	1986.49	631.4635	1248.821	574.6	709.8692	753.35	977.2422	895.8	803.8
2673	521.33	60	5342.98	2306.739	4479.009	2747.778	5136.869	5362.54	3822.729	4343	3598
2674	521.35	122	3200.34	2497.311	3319.895	1850.663	867.7463	2296.956	2526.3	2032	2401
2675	521.73	72	2978.39	3217.986	2666.124	3441.835	1656.225	3652.84	2045.231	2988	2574
2676	522.28	156	1604.54	959.3673	1532.441	664.508	650.1249	785.8575	1204.321	967.1	1059
2677	522.35	183	14627.3	8658.979	35924.33	6451.805	17130.94	24849.06	12251.31	23369	17239
2678	522.41	140	1797.21	2174.184	988.3627	1903.138	1640.611	1173.653	1340.051	1890	1768
2679	522.6	72	6651.8	3126.346	5495.599	4330.561	1801.186	7064.68	5309.496	6173	4434
2680	522.82	80	1760.67	1533.126	3299.266	1027.268	0	1031.745	1518.186	760.7	1811
2681	522.87	62	1170	674.67	1337.252	601.744	473.3239	534.1926	533.5733	556.4	928.1
2682	522.88	114	863.217	608.75	884.224	662.76	1299.102	1146.09	571.535	498	717
2683	523.24	118	168520	42497.07	212427.1	37396.96	78681.67	96766.93	79129.79	1E+05	93329
2684	523.29	71	3140.79	3984.827	2551.036	3632.039	2912.913	4638.01	2627.626	3376	4261
2685	523.36	184	9840.03	4370.938	6372.411	2071.52	5572.601	11021.39	2749.793	4591	5410
2686	523.72	70	1193.46	1070.729	1217.998	767.1675	768.3422	1341.102	978.2771	1108	790.4
2687	524.25	118	58922.7	17290.03	78613.8	8990.258	26101.31	39139.01	29091.83	38012	32316
2688	524.29	70	1142.7	1200.075	716.2908	1359.777	1081.683	1345.185	636.1583	1675	815.3
2689	524.37	258	22448.3	21920.61	68689.35	8932.442	10622.29	19723.33	25036.69	25806	13900
2690	524.59	72	5948.37	2568.49	3763.868	2420.928	961.9693	4024.86	2291.935	3956	3909
2691	524.68	64	985.266	480.8533	1787.784	359.823	639.9633	1177.209	805.9333	3139	1208
2692	524.86	62	775.008	371.808	506.867	501.861	186.7723	277.936	421.2243	387.2	698.5
2693	525.25	118	31628.1	7252.309	36447.75	6370.69	13829.13	16148.71	12625.79	16071	16326
2694	525.29	62	5032.89	3068.827	3655.213	2006.316	4575.996	5133.058	3254.399	3464	3155
2695	525.33	211	1001.72	783.299	925.704	1004.442	783.394	824.681	801.6884	904	920.1
2696	525.33	183	2195.39	1869.903	1687.179	1591.52	1940.665	1965.609	706.5792	2060	3247
2697	525.37	257	15953.4	7116.231	15752.04	1979.998	3622.63	6904.672	7165.987	8583	3642
2698	525.9	82	2292.72	2320.074	4981.36	1796.274	1537.681	662.7157	1752.144	1469	1514
2699	526.25	118	8973.99	2453.047	9582.335	3167.714	3877.284	4666.717	2919.536	4677	5164
2700	526.29	98	3583.69	877.5927	1380.344	2268.508	2124.42	3213.095	1275.096	2536	2388
2701	526.42	120	3059.41	2985.046	3878.005	2394.525	3170.16	4345.553	2505.816	3442	3515
2702	526.43	1073	2536.48	2601.558	2619.771	2344.563	2418.905	2748.065	2817.443	2568	7690
2703	526.43	1134	4214.12	3456.122	5242.704	1998.953	3483.866	1662.581	4476.383	2870	2695
2704	526.43	166	11052.4	7872.506	9179.767	9080.127	11864.24	10957.11	6369.107	11376	16312
2705	526.43	1055	2957.87	7179.167	7986.57	7065.888	7704.442	3220.617	9166.878	8741	8594
2706	526.59	72	1182.84	1188.572	1465.818	1640.161	392.4153	1930.5	1168.86	1290	2492
2707	526.68	76	2705.23	1216.353	1173.685	714.096	501.8489	723.576	2583.158	1969	1388
2708	526.77	62	4404.98	2015.663	2205.856	1403.192	851.983	1599.117	2045.793	2800	3659
2709	526.93	85	3531.33	7005.728	7962.922	5207.516	8577.075	2493.513	5577.034	5976	3276
2710	527.25	118	2196.36	627.15	2261.513	565.4557	1652.64	1325.52	1199.721	1273	901.2
2711	527.31	79	4332.22	3194.443	3199.731	8476.95	11025.54	1850.34	5299.525	3030	3980
2712	527.43	165	3758.11	2723.541	3265.65	4930.627	8351.492	3596.83	2569.478	3695	4870
2713	527.93	88	8763.33	3014.13	4189.53	3045.564	2052.917	5467.404	4805.156	4002	6336
2714	528.31	117	3673.59	3544.626	5247.701	4293.892	3228.251	4528.789	3431.949	4336	4400
2715	528.33	140	3585.32	5639.548	1585.531	6755.997	3293.365	3726.192	2092.86	2695	4251
2716	528.41	112	11667.5	6002.102	14292.78	6522.083	14869.38	12471.25	1531.558	10639	12480
2717	528.41	1072	1801.44	1859.757	1798.507	2098.931	1943.704	1787.364	1070.873	1921	1915
2718	528.65	118	5038.2	1465.98	6238.722	1416.227	1040.001	2655.3	2447.458	2436	2504
2719	528.77	78	5070.45	4009.495	2497.564	1500.996	1237.358	2891.882	3686.952	9571	2415
2720	528.77	62	6167.59	1852.44	2375.306	1635.294	722.8556	2230.598	2205.834	3034	3477
2721	528.89	83	9940.37	8489.886	15675.57	6396.968	11735.89	6776.571	7291.102	8652	8332
2722	529.33	81	3089.92	2972.369	1948.756	4410.484	3144.232	1966.159	2713.751	2277	2105
2723	529.33	140	1857.92	1627.662	1806.92	1825.547	1408.803	1538.526	1364.336	1411	1895
2724	529.41	112	3178.02	2729.48	5022.107	3628.602	5623.516	3617.708	2768.411	4094	3974
2725	529.53	259	4578.78	857.0958	1199.47	2983.754	4462.087	2591.082	2341.144	2234	2857
2726	529.65	119	2593.74	795.6929	2782.828	1176.502	1108.816	2937.955	1964.944	1859	2019
2727	530.28	78	3191.65	4579.058	3483.27	3811.393	3376.64	3323.335	3623.68	2668	3706
2728	530.28	104	3264.79	2193.506	4650.266	3888.36	2225.854	6248.216	4162.18	4387	5598

2729	530.35	119	1264098	423356.3	1317038	375996.1	449701.2	850447.6	567777.1	7E+05	9E+05
2730	530.41	112	1285.57	53579.64	1302.737	132591.2	908.9131	1291.791	1157.942	1189	805.2
2731	530.65	113	813.703	1616.846	1031.101	877.9543	763.1413	690.7829	563.968	511.8	883.1
2732	530.76	77	3904.54	2369.037	1322.009	3181.927	2983.708	1614.658	3371.521	4708	3210
2733	530.77	62	3335.34	1214.72	1270.463	1062.014	298.4538	1253.88	1383.349	1485	1614
2734	530.85	82	7024.16	5002.075	13700.58	4866.12	5186.601	1640.333	2737.884	5542	3816
2735	530.98	87	1977.02	530.665	0	2296.65	2765.665	5323.901	3910.429	6342	5214
2736	531.26	113	2953.53	31639.8	4987.968	3781.221	5182.268	195389.4	892.9294	3E+05	2E+05
2737	531.27	169	2245.32	1526.558	1677.037	1670.941	1558.361	1874.681	1385.994	1907	1798
2738	531.35	118	489609	145892.1	480954.6	127031.7	155234.7	320677.7	195767.3	3E+05	3E+05
2739	532.26	111	2361.91	17307.81	3161.365	1326.78	2738.84	15777.6	19502.6	31706	821.7
2740	532.28	139	8131.16	11621.39	11648.39	13188.53	20958.83	10931.41	10205.85	14027	13887
2741	532.29	75	2108.57	3541.08	2101.603	2499.631	782.1485	2160.572	2454.209	429.2	2152
2742	532.35	114	264553	265760.2	250511	247417.2	221649.6	148421.9	93849.87	1E+05	1E+05
2743	532.76	64	1905.33	638.3	744.9244	521.6538	348.4298	677.9483	2135.098	789.2	895.2
2744	532.89	62	949.969	798.9055	487.2736	595.0154	197.847	517.235	337.4064	475.5	776.9
2745	533.28	139	2434.6	3876.869	4382.381	4139.756	7142.757	2686.78	5129.342	3573	3046
2746	533.36	112	74433.5	90604.16	72219.58	80732.67	74800.94	42486.34	29736.89	35591	40245
2747	533.38	1046	1030.58	989.6747	801.3241	962.7891	709.665	906.6167	1050.24	873.8	1567
2748	534.28	140	1190.59	1303.085	1479.305	824.8414	1439.763	1150.232	1383.317	1438	1194
2749	534.31	64	1566.46	2167.045	1725.008	1661.034	1425.436	1473.111	1501.393	1462	1109
2750	534.36	112	17917.2	20924.1	15227.28	15372.24	17714.69	8760.209	4448.407	7769	9764
2751	534.45	241	3056.55	2772.928	2331.275	2904.93	3915.219	2018.086	1974.319	2331	2580
2752	534.72	73	4039.2	1571.131	3057.421	734.4273	1142.072	2939.781	3275.033	2491	1403
2753	535.36	112	3150.5	3563.126	2627.745	3093.3	3255.016	1825.622	1136.551	1025	1174
2754	535.45	241	1251.9	1164.339	1020.321	1203.326	1555.036	894.6	1055.715	946.9	842.4
2755	535.71	73	1852.29	1763.868	1787.805	1586.304	829.4	2482.85	1664.692	1745	1793
2756	536.16	120	3665.96	5131.316	7123.951	3935.799	4269.086	5268.667	4737.628	3604	6460
2757	536.16	1076	4072.3	62161.58	6674.013	2488.095	2667.599	3367.56	2556.02	2819	4450
2758	536.16	470	3685.52	3385.594	7527.553	3718.193	3546.79	3319.671	3523.979	3958	5694
2759	536.17	84	3267.73	7464.54	9789.871	34204.71	15995.06	13012.8	7757.248	5247	13153
2760	536.49	98	6133.49	9305.931	889.66	7404.882	5556.096	9193.328	814.9905	1186	9544
2761	536.71	76	2500.02	1314.318	1651.767	1484.585	729.5916	2130.333	2472.711	4130	1084
2762	536.8	79	9144.72	3938.526	7389.532	2340.57	1720.048	2754.816	5840.885	10697	3714
2763	537.16	1	727.126	496.0886	0	260.7985	0	421.7933	344.6465	509	557.7
2764	537.16	1070	2984.27	26997.21	8397.892	2351.412	2635.923	3393.519	2336.75	3151	4565
2765	537.16	470	1904.7	1511.806	4907.551	2010.168	1978.98	1593.191	1615.344	2358	2912
2766	537.17	95	3990.32	4380.728	1867.32	2762.063	1473.899	4976.405	3054.551	3644	6858
2767	537.29	77	1107.68	5036.873	1022.078	14501.3	8763.584	4692.211	2095.395	3158	4128
2768	537.49	98	2844.88	3232.643	356.0828	2222.507	5101.435	3495.317	485.4359	826	2367
2769	537.7	72	1634.78	739.5098	2214.393	1452.442	880.4367	2768.123	1358.05	1131	2369
2770	538.16	472	1907.97	1793.07	3302.3	1600.261	1848.483	2006.095	1449.277	1983	3665
2771	538.16	95	5009.27	1337.009	1066.704	1729.92	2108.371	1021.255	1469.978	1540	3387
2772	538.16	1	463.472	340.8501	491.1764	238.0313	312.4975	258.6504	377.3807	384.3	438.1
2773	538.34	1046	2242.8	2604.252	1876.013	1621.862	1934.5	2638.736	2082.282	2011	3255
2774	538.43	87	30605.7	2201.797	2161.09	16032.5	52051.28	1210.455	13182.4	11549	17924
2775	538.52	141	2647.41	2130.443	257.9765	548.8067	2225.588	2558.637	354.33	251.9	215.1
2776	538.57	75	1387.94	911.0883	1287.682	719.603	108.647	1853.486	932.7336	1490	1707
2777	538.8	78	7125.69	3692.822	5164.27	3132.96	1980.523	3682.512	5659.888	6693	3702
2778	538.8	62	4124.57	1000.654	1718.1	1673.147	622.6971	2137.494	1909.206	2532	3601
2779	539.24	106	5255.59	10814.67	9358.035	7122.413	7776.222	11331.76	4977.481	8130	11163
2780	539.43	87	11424.8	322.1943	733.7888	8368.222	18795.49	254.6345	3921.434	6804	6281
2781	540.24	105	3289.36	5004.866	3906.068	4195.006	2795.583	4217.225	2394.993	1136	3628
2782	540.42	724	602.023	338.793	506.3266	646.935	874.7209	688.22	615.5453	757.3	1045
2783	540.44	89	3432.45	1632.491	1096.299	3836.588	7466.856	787.9725	1268.805	2697	5457
2784	540.44	142	2331.81	2079.182	2765.428	2351.221	2401.74	2107.034	1829.52	2683	2493
2785	540.44	1	214.159	130.592	223.189	169.7651	400.56	267.3748	269.01	218.1	212
2786	540.44	1072	1883.97	1720.879	1765.442	1619.73	1590	1581.202	1735.077	1585	5820
2787	540.44	1173	3546.77	2725.632	631.0109	2235.078	3509.429	3069.675	1130.588	3427	2769
2788	540.45	205	5504.46	5129.251	6125.642	2311.347	5083.964	6364.068	4033.199	5231	7631
2789	540.53	1045	4639.03	4227.301	1385.566	954.8428	1389.481	4844.525	1354.103	1259	1555
2790	540.53	466	390.664	316.905	3762.864	3463.992	4675.76	449.0552	544.2165	2299	7476
2791	540.53	410	204.505	190.0926	1438.298	2379.434	3101.799	163.5469	215.7038	1788	3461
2792	540.53	124	1918.4	1362.755	1640.76	1146.389	2009.131	1396.541	2381.383	700.4	1233
2793	540.53	382	413.232	624.1381	1396.815	3415.58	7022.63	491.024	568.23	2359	1669

2794	540.57	71	1119.74	714.7238	1369.295	441.1143	174.3638	710.4846	482.4011	303.7	695
2795	540.79	62	2280.65	1265.6	1276.974	909	439.4563	905.464	1419.542	1168	1660
2796	540.8	79	2898.96	1807.495	2315.795	802.7613	493.9088	1346.725	3741.169	4106	1326
2797	540.9	89	1261.66	2294.849	1536.02	1780.344	2152.401	1154.43	1766.801	1990	1493
2798	541.24	106	1346.33	2709.728	4867.848	1722.373	3700.66	2341.508	3567.531	4317	2747
2799	541.28	78	7881.84	7246.171	3721.675	7149.81	3458.624	2531.176	2030.558	4145	6207
2800	541.33	141	699.133	1125.855	2620.671	1745.562	1585.422	1903.45	1862.057	2213	1430
2801	541.44	99	2895.5	3474.317	991.2733	975.2683	3791.119	1177.867	1148.874	1619	3856
2802	541.45	209	1887.95	1233.181	1539.143	2592.65	1890.322	1748.605	1609.863	2130	3054
2803	541.54	468	265.7	224.0428	3267.002	1663.445	4315.22	271.5192	388.9903	1560	980.4
2804	541.94	86	5969.57	6587.28	8873.263	2031.455	4259.095	2707.082	5310.462	4628	4820
2805	542.28	77	2191.8	2291.844	1224.305	2478.228	1921.28	3984.931	1888	2410	1743
2806	542.42	121	17023.4	7742.836	12076.61	12394.25	11488.06	20058.76	9012.565	12853	14574
2807	542.76	62	1330.97	1063.316	1185.987	698.7445	471.9363	1107.525	1076.144	653.6	1404
2808	543.34	118	267227	102643.3	322575.3	71116.64	204678.4	193431.5	136553.9	2E+05	2E+05
2809	543.43	121	5715.87	3096.679	6475.021	3356.683	3504.429	6763.067	1951.158	5239	7203
2810	544.3	79	1568.51	5460.7	2723.416	3258.64	3015.253	2823.46	3144.22	2287	2544
2811	544.34	118	112202	37897.79	130307.8	30341.23	79664.11	81123.67	65329.07	69676	68013
2812	544.43	121	1597.62	845.3456	2467.318	2963.866	2549.05	1687.599	1291.25	2958	2684
2813	544.61	70	3529.2	2257.021	1645.093	1807.286	1474.2	2308.175	2058.683	2824	3140
2814	544.75	63	3414.99	846.4585	2651.616	741.052	877.6324	1130.638	1382.748	1220	1484
2815	544.86	83	1785.82	725.3034	3247.213	1539.64	767.296	1849.697	764.841	998	2004
2816	545.31	79	1486.62	1798.26	2325.828	1096.134	1969.098	2194.159	1658.556	3080	1154
2817	545.35	114	74328.3	52161.75	76468.14	52220.71	62537.49	42812.94	34047.2	39558	33581
2818	546.28	77	4845.65	7078.48	4515.929	6948.091	2415.513	11862.08	2168.266	2969	7510
2819	546.35	112	26055.3	21071.1	28180.63	25129.21	25133.8	14927.89	8585.406	16025	12701
2820	546.35	256	8861.87	2870.144	9826.452	1188.083	2297.759	2291.685	2087.804	6373	3166
2821	546.39	123	1604.07	1041.495	2506.384	1509.026	3138.318	3158.28	2706.48	2269	3523
2822	546.61	70	9283.29	7515.288	4713.884	5706.201	2439.239	7888.86	6434.484	7752	7555
2823	546.75	62	1707.2	2741.76	1046.307	2235.388	937.0286	2597.59	813.696	2634	3875
2824	546.83	79	18403.1	9526.875	11179.5	5387.317	5630.964	6394.575	12889.08	13645	6386
2825	547.23	111	2001.05	1707.775	1913.75	2530.59	1973.684	961.9875	1769.071	1922	829.6
2826	547.29	75	1920.49	2198.312	990.6967	2253.416	1905.5	4087.349	1263.844	2261	2898
2827	547.35	249	2928.4	1265.86	5776.323	778.8664	1176.448	1359.878	1570.832	1673	1699
2828	547.36	114	6732.66	6119.997	5808.908	4111.828	6621.71	3117.717	3037.762	3490	4457
2829	548.3	139	10138.9	10783.59	11473.93	14486.1	24047.15	11019.76	9743.72	11837	19906
2830	548.36	62	1625.34	2064.252	2013.143	1544.215	1771.367	2405.13	1513.696	1515	1757
2831	548.36	113	1862.56	2355.783	1590.04	1559.796	2951.734	1412.504	799.7457	1078	933.4
2832	548.61	72	11097.2	8096.099	6957.768	9101.065	4980.366	10369.14	1478.689	12030	10901
2833	548.83	80	10042.2	4581.98	10703.29	3622.958	4008.368	4686.492	5534.266	9792	3910
2834	548.83	62	3771.26	2178.291	1374.307	1523.37	465.7256	1747.286	1439.415	1818	2596
2835	549.3	139	5140.88	8741.904	5671.123	5918.48	11253.3	4212.22	5867.612	5990	4783
2836	549.32	156	5603.16	2267.649	5864.558	1335.51	3259.505	3252.923	2473.67	3602	2945
2837	549.34	112	1470.24	570.1605	724.3933	694.26	1013.351	926.409	862.524	1145	747.3
2838	550.3	139	1602.22	1876.74	1725.403	1714.818	3187.7	1355.333	1514.261	1341	1790
2839	550.31	155	5994.52	2296.63	6167.649	1318.05	3206.134	3169.242	2558.875	3720	2846
2840	550.44	259	1377.74	1318.009	7607.711	1410.433	1047.524	2185.604	9796.451	1332	1495
2841	550.44	197	5702.33	6185.822	4691.974	9602.824	5120.616	5317.081	5795.075	5791	7614
2842	550.61	72	9883.93	6450.612	5204.264	5275.324	3853.562	8324.702	6002.22	7498	7333
2843	550.82	61	1841.62	1269.757	733.589	735.552	344.8104	852.2564	842.4486	914.3	1431
2844	550.82	80	2989.97	1032.763	1640.852	1018.248	2469.6	2255.52	2829.339	4002	2133
2845	551.31	154	2630.8	1009.318	2086.08	573.3	1165.371	1085.747	966.6124	1493	1091
2846	551.45	197	2175.46	2390.027	1795.985	3873.658	2110.981	2045.218	2763.184	2592	2794
2847	552.07	248	2104.41	325.7283	1535.94	378.1696	467.2733	682.5456	759.4772	978.1	918.1
2848	552.07	267	1713.14	227.6688	960.8857	219.5171	310.6031	363.35	498.6107	663.4	514.6
2849	552.27	102	1052.46	848.7205	649.9016	853.15	659.2218	1487.583	1014.381	1214	889.6
2850	552.28	1048	1410.46	1336.821	1277.46	1019.343	1102.915	1381.744	1193.373	1199	1527
2851	552.35	116	1504.25	344.65	791.1163	1047.751	1756.755	1175.185	858.06	727.9	1349
2852	552.6	70	2771.65	2926.22	2632.776	2411.799	2125.5	2602.95	3302.482	3818	3537
2853	552.77	79	4184.64	2113.202	3758.066	1824.302	713.6567	2295.883	3653.364	5412	2580
2854	552.81	62	2849.85	1349.381	1137.78	637.7458	452.8711	955.0564	1028.678	1313	1301
2855	553.11	77	1131.9	2959.967	188.3692	2731.916	1064.512	2387.53	2529.234	1032	1338
2856	553.25	140	1374.31	1161.981	1528.282	1623.06	1679.485	1179.184	1713.99	1784	1861
2857	553.32	63	939.284	1381.728	1106.034	1853.856	1717.242	1391.911	1213.336	1657	1135
2858	553.33	118	1415.88	911.24	1927.684	1032.965	1248.32	1579.853	781.9429	775	1001

2859	554.17	185	3656.32	1706.27	1691.088	1509.875	2655.228	2388.03	683.279	1036	1263
2860	554.26	140	512.968	777.4975	828.6688	683.824	762.3939	757.4811	643.5669	627.3	714.6
2861	554.27	79	5114.34	9930.508	5886.022	1899.212	1097.538	1358.86	2354.823	2463	4950
2862	554.29	96	2499.09	2396.604	2317.571	4097.869	4528.213	5873.29	3525.429	4214	4021
2863	554.42	1161	1473.19	1406.817	1600.241	1233.913	1437.289	1413.198	1052.333	1620	1890
2864	554.46	239	1563.59	757.119	1624.965	1408.759	2073.368	1223.096	1306.464	1523	1622
2865	554.6	67	1849.81	1167.388	635.6	727.3149	1040	815.813	916.9182	1373	749.6
2866	554.77	62	931.063	1133.225	1509.544	878.6913	366.882	975.46	1431.464	1454	1523
2867	555.1	77	805.933	1000.247	23.2236	1735.36	0	1798.672	1557.76	1652	883.3
2868	555.18	185	1879.92	1166.109	909.048	870.2016	1305.807	1314.116	406.0891	682.5	720.3
2869	555.28	98	1042.14	1186.072	1211.454	1278.402	1197.331	2563.183	1368.469	1033	2309
2870	555.31	78	4389.35	1372.572	8798.637	3530.6	3283.148	3625.44	3295.095	3708	3228
2871	555.34	118	3051.06	1958.768	2691.708	648.1996	2206.939	4602.581	2341.292	2751	3494
2872	556.3	108	4514.31	5248.119	4459.748	5090.657	6109.591	6428.177	5602.512	3866	3992
2873	556.34	118	9830.76	3173.675	9119.5	3730.542	1800.223	6245.258	1666.665	4940	7014
2874	556.44	1059	2396.85	1705.334	2343.403	2456.151	1669.7	2703.492	1263.388	2384	2724
2875	556.44	97	2283.08	1250.031	2272.248	1683.375	1537.43	2605.2	1250.368	2946	1446
2876	556.44	1104	2475.97	2574.072	2323.056	2462.335	2526.7	2542.703	2300.088	2582	3522
2877	556.44	145	10954.7	8315.787	7942.777	11238.49	13619.9	12005.37	11195.46	14486	12666
2878	556.53	417	5894.36	1861.673	398.1453	1341.682	5381.292	2803.562	439.9915	354.1	1499
2879	556.53	209	1240.09	1010.808	8333.669	1238.676	1388.357	982.1037	2977.622	10167	1333
2880	556.64	69	3809.89	1100.877	1489.8	873.6611	530.4	805.6064	1490.977	4051	875
2881	556.77	77	4149.22	1412.677	1693.119	777.0894	2047.617	1379.396	3383.746	3289	1831
2882	556.86	82	26797.3	33296.74	31951.32	15835.21	27686.36	17562.81	25283.75	27402	26756
2883	557.34	117	4286.04	1859.406	4288.258	1230.324	1561.89	3293.5	2486.943	2595	3462
2884	557.44	143	4180.69	3648.456	4627.534	4551.749	5313.608	4299.581	4331.65	4072	4596
2885	557.86	82	4488.95	4568.271	2866.875	3085.335	3214.889	2083.324	2306.553	4280	3611
2886	558.32	128	3220.1	1963.007	976.25	4228.903	2755.604	4633.72	1902.418	1804	3448
2887	558.38	119	33971.9	8841.778	36993.73	8170.073	12861.28	20510.27	14397.91	20011	19252
2888	558.4	1047	1434.94	1464.015	1777.246	1540.169	1285.207	1771.973	2148.789	1457	2096
2889	558.44	393	12972.4	8242.287	13277.3	6990.109	10951.92	10802.4	11999.81	13174	12963
2890	558.64	70	4568.26	1579.154	2236.536	733.1365	701.0228	913.5971	1973.03	4180	1999
2891	558.85	82	12378.7	11372.05	14122.32	7390.804	12138.5	7425.538	11655.01	13243	11331
2892	559.13	187	1763.52	685.5111	799.1573	560.9021	972.4796	1236.788	237.2314	527.7	449
2893	559.38	118	12630.9	4159.845	15599.85	2319.751	4733.482	9455.464	6353.626	9823	8705
2894	559.44	393	5750.64	3788.356	6053.34	3230.277	4347.202	4754.074	6862.956	5720	5546
2895	559.91	78	0	1006.23	1169.502	1124.624	343.8	0	2005.77	3268	521.8
2896	560.32	118	2646.03	498.236	3494.702	906.8191	848.8254	2005.911	906.5111	2415	1638
2897	560.38	113	11917.3	12275.82	13471.29	11543.88	8932.377	7022.253	5729.638	7299	6453
2898	560.41	87	24662.4	1413.558	1539.884	11387.3	32789.02	763.7266	9190.805	12216	10825
2899	560.44	393	2206.38	1439.38	2092.65	1197.711	1540.633	1758.314	2737.446	1964	2375
2900	560.5	143	4653.47	1380.715	134.5361	642.6	2602.831	3138.363	712.4033	244.1	669.7
2901	560.59	71	4169.84	2475.493	1801.735	2103.919	1262.421	3050.529	3036.642	3213	2336
2902	560.63	68	2573.78	1928.32	1267.488	1567.921	1446.712	815.4886	2019.618	2092	1929
2903	560.85	80	3249.11	2270.835	4144.462	1536.42	1739.787	3600.774	2226.674	3762	2508
2904	561.31	155	3293.6	2300.724	2408.438	1512.185	1601.611	2257.288	1268.913	1774	3042
2905	561.38	112	4644.34	4597.266	4521.491	4688.46	4067.763	2762.053	2498.796	1931	3346
2906	561.42	87	8528.2	569.6475	0	4324.367	13010.45	861.0973	5226.771	2511	4590
2907	562.31	153	1255.65	1135.443	920.6229	890.1	999.3921	936.5951	932.6728	949.7	1116
2908	562.33	263	1920.72	1286.783	5117.487	611.246	1058.915	1177.74	1246.497	1460	1565
2909	562.36	70	5029.27	7015.758	5652.745	7281.916	5949.444	7777.764	4169.9	6721	5512
2910	562.38	112	1175.55	949.1398	1260.518	2002.34	1294.125	692.5769	796.9932	602.4	1062
2911	562.59	72	9972.21	5607.303	6674.966	7267.686	3454.1	8114.293	6167.967	8120	7811
2912	562.8	62	1446.52	816.1778	1293.1	953.6677	465.7256	885.085	1182.733	1064	1295
2913	562.8	80	8565.22	3670.73	9702.814	3110.919	768.936	3720.312	5956.35	6821	3620
2914	563.21	111	1854.07	878.8379	748.995	1431.36	516.5	901.9875	504.722	784.5	383.9
2915	563.31	154	1309.24	892.1501	908.791	673.4	644.64	827.6718	558.9175	690.9	1230
2916	563.34	120	578.879	703.7385	907.5383	956.1977	975.1664	795.3375	709.075	494.7	437.8
2917	563.36	70	1526.02	2321.749	2021.344	2753.134	2210.817	2831.476	1656.468	1239	2038
2918	564.29	109	2038.99	1626.65	3102.36	1358.366	2511.195	1756.422	1474.742	2232	1990
2919	564.36	61	5153.53	4185.628	4612.008	3070.217	4936.264	6229.555	4154.766	3981	4483
2920	564.58	71	12821.9	8436.517	7969.17	6628.768	4332.9	10807.3	8561.952	10484	11409
2921	564.71	64	1160.12	296.9573	1187.956	252.0413	529.1	526.7296	796.8255	1463	703
2922	564.8	78	6711.9	2436.1	7167.697	2821.832	1736.62	3461.64	5002.5	4226	2604
2923	565.36	61	1613.65	1469.359	1412.964	1750.399	1569.914	1800.746	1358.465	1436	1451

2924	566.4	113	747.18	708.4433	914.9333	626.22	1151.638	536.784	677.515	638.8	735.6
2925	566.58	71	9287.69	7342.928	6381.989	4238.296	3656.835	9609.768	6525.361	8301	9745
2926	566.66	77	2425.43	1066.416	823.4021	1372.329	502.0575	1798.672	2060.622	3675	844.5
2927	566.67	63	4935.51	614.544	2305.573	515.0518	902.1795	1156.923	1832.508	2031	1352
2928	566.79	79	3784.48	2173.253	2276.638	1215.953	661.7344	1416.681	2302.758	2076	2641
2929	566.89	85	115484	134921.1	132130.5	61015.08	115867.8	82067.79	99984.39	1E+05	1E+05
2930	567.31	71	4026.04	5018.276	1914.267	4442.183	3135.465	4577.045	3338.781	4238	5178
2931	567.36	124	2038.97	680.68	1508.328	1373.95	1064.509	1941.136	1471.142	1517	1606
2932	567.89	85	15990.4	20728.26	15782.82	5359.389	17292.19	10881.14	12936.13	14857	15267
2933	568.18	141	2063.27	892.185	1778.788	684.336	642.348	963.8231	655.2975	744.6	557.4
2934	568.32	70	1545.93	917.0673	943.8883	1855.649	920.4506	1186.755	767.1717	1077	1341
2935	568.34	115	1876.2	1362.424	2584.165	2023.312	3278.533	1765.149	1884.991	3060	2198
2936	568.45	139	3796.27	2831.931	1221.896	2051.534	4059.869	3225.222	1058.34	809.9	2051
2937	568.58	71	5243.89	3213.07	3561.506	3483.648	1782.3	4980.133	4347.378	4625	5027
2938	568.67	65	3379.8	860.654	1991.65	773.1648	1168.026	1787.583	3422.958	4302	1502
2939	568.89	85	5561.11	6032.406	5748.676	4085.768	6977.013	3380.073	6069.42	7126	6381
2940	569.19	118	1562.67	900.1333	2457.67	553.941	861.58	1082.331	1295.254	1207	731.6
2941	569.29	112	2084.13	1483.82	1668.2	2198.738	2319.738	1233.523	1081.744	2672	1376
2942	569.31	62	3603.74	1923.999	2395.6	2227.339	1838.626	2681.603	2342.625	2616	2943
2943	569.46	137	1471.11	1719.902	1456.541	1184.15	2036.444	1640.778	679.4624	373.6	1237
2944	570.36	119	2528.01	1316.706	2476.382	1877.933	1479.867	3113.088	1872.433	2422	1549
2945	570.46	164	13947.2	5647.011	7329.922	7465.522	9136.064	8574.151	6456.223	8743	8723
2946	570.58	70	1778.85	1349.193	1143.333	953.7063	713.7549	859.2024	1626.732	1728	1025
2947	570.74	64	1705.38	581.7044	1023.851	422.2297	343.2293	733.441	896.5889	2485	882
2948	570.87	82	7700.06	8145.593	13496.45	2814.876	9783.167	4312.1	8386.386	10854	6047
2949	571.37	119	6902.79	2281.246	9661.844	3067.978	3213.608	6071.1	4924.945	2107	5005
2950	571.46	164	3286.8	3076.242	2547.866	2630.995	3213.994	3016.466	2703.669	3380	4759
2951	571.95	88	5867.82	4736.149	6859.589	1274.84	3562.02	3478.889	5212.017	5079	4557
2952	572.36	119	12472.2	5699.1	12643.43	5083.312	3613.914	10260.23	6396.482	7410	11488
2953	572.43	111	10332.2	5417.052	12097.9	7783.621	12980.53	9943.708	8130.861	7516	9359
2954	572.62	67	851.5	668.202	1361.376	376.0179	679.9523	757.448	980.5756	1433	692.4
2955	572.79	63	1265.66	1393.367	861.9943	754.6519	486.2	869.2276	1054.224	869.4	1516
2956	572.83	81	13705.7	3408.134	19324.78	7935.16	8711.928	8252.396	11910.94	13993	9020
2957	573.17	81	1528.8	3088.95	4985.58	2814.565	4622.448	514.855	2783.491	2749	1226
2958	573.39	119	38485.4	12739.64	43299.62	11388.77	15465.94	28775.42	21433.71	25555	23060
2959	573.44	111	2578.56	2293.186	3655.973	3354.272	4572.492	2870.801	2807.676	3901	2846
2960	573.55	250	4767.35	348.4804	217.7047	2482.108	4075.6	882.9842	1662.729	1487	1368
2961	574.06	260	2040.3	596.9801	1944.186	501.528	502.1496	528.1421	565.7312	1352	1291
2962	574.29	75	1686.29	983.7664	531.9277	2073.699	528.2917	2047.347	1513.838	1601	1988
2963	574.39	118	3202.45	4683.132	22338.81	5926	6828.35	2678.373	7213.438	10010	11115
2964	574.44	111	1215.53	6796.199	23292.07	1441.081	1716.397	1337.67	11220.3	1618	11046
2965	574.61	73	1770.57	1131.507	1378.533	791.3918	644.3379	647.55	1167.45	3083	1008
2966	574.81	61	1464.9	583.7257	270.6	396	326.3359	394.3029	482.8629	1060	987.8
2967	574.83	81	5334.47	4480.874	8548.079	2308.85	4721.5	5038.859	4223.87	4506	5048
2968	575.3	118	1181.88	817.6583	908.544	756.93	325.8324	537.2	711.5429	151.8	1020
2969	575.33	143	1194.79	1984.309	1328.818	2229.406	1784.045	1456.57	1800.878	1642	2042
2970	575.39	115	9604.07	6798.3	4061.581	5792.702	5532.942	4611.066	4661.742	4694	3697
2971	575.69	75	1221.19	818.2977	703.134	827.4286	405.6595	945.8172	872.2182	1016	1259
2972	576.26	97	642.781	670.32	830.6567	807.3222	1018.96	634.5746	909.72	1130	803.1
2973	576.33	140	55806.5	55228.68	43258.1	63883.07	58602.51	61612.49	47756.53	62284	62915
2974	576.33	169	3001.88	2085.247	2721.183	2417.529	2471.8	2759.222	2422.019	2625	2596
2975	576.34	68	630.72	1721.78	415.0544	1396.211	988	1095.589	732.84	1248	1105
2976	576.39	86	11233.8	1947.01	1137.749	3834.286	13933.61	1002.499	5622.462	6124	4959
2977	576.4	113	3401.11	3905.122	3193.35	3393.197	1759.112	1659.502	1228.784	3491	3235
2978	576.56	71	2169.62	1297.098	882.2925	1692.731	565.5435	1357.16	5967.863	934.7	937.8
2979	576.7	63	4431.09	1025.55	2468.237	768.1695	516.1176	1687.496	1654.339	2916	1909
2980	577.33	140	23125	23718.75	22708.97	29745.55	23779.31	24711.41	20263.87	25850	26488
2981	577.39	86	3230.83	1037.85	626.484	1657.787	4755.651	937.8229	2159.795	2775	1710
2982	577.39	111	1922.22	1647.398	2356.392	1755.569	2082.972	1089.846	1029.764	2296	1384
2983	577.51	85	655.67	2345.828	1023.858	2387.985	2140.087	1675.393	5580.714	707.2	733.8
2984	577.52	1180	664.759	930.784	1756.599	874.0534	943.9872	739.0512	26519.55	1998	930.9
2985	577.52	46	3094.44	1946.346	1440.614	5708.639	2737.342	2606.971	2892.274	918.9	821.6
2986	577.52	2	518.968	1492.092	428.3895	637.2147	311.7713	380.1403	406.9557	391	216
2987	577.52	1073	1514.16	1406.004	7057.617	1981.908	2410.306	1588.251	23268.66	9489	1839
2988	577.52	122	2718.35	1517.67	1496.632	14736.67	2554.284	3819.01	5408.837	628.8	220.9

2989	577.69	71	2103.79	2498.408	1348.336	1569.81	1043.9	1516.512	1601.323	2718	2285
2990	577.91	83	4706.22	6448.68	6907.37	3569.103	5105.789	6053.21	4275.463	5509	5144
2991	578.34	140	5623.84	6412.781	6442.749	7698.074	6708.559	6220.406	5671.82	7162	6733
2992	578.48	299	1038.85	1416.687	928.6875	2282.188	766.681	1139.355	1170.818	1016	1481
2993	578.51	53	896.436	584.3797	335.3082	1353.884	563.6107	1243.502	912.8246	293.4	352.6
2994	578.56	72	4119.35	2006.714	3051.067	2514.291	1080.627	4243.96	2581.415	2894	5086
2995	578.77	77	3231.45	1293.188	2729.386	654.7163	610.0475	1558.626	2422.189	2048	1216
2996	579.29	1072	1616.37	1571.627	1140.368	1446.776	1081.94	1759.478	1583.898	2143	2008
2997	579.29	106	21309	16711.37	31262.84	12091.13	22112.33	17997.98	13679.44	25941	21148
2998	579.34	140	1930.73	1404.054	1650.673	2295.302	1305.87	1413.336	1605.083	2138	1879
2999	579.69	69	2234.47	1765.712	2118.88	2372.976	1330.176	3808.581	1361.298	1866	2413
3000	580.29	106	8877.65	6109.05	11991.07	4910.22	9442.3	7738.283	6689.511	9768	9099
3001	580.51	26	2956.75	6032.645	282.7709	4248.192	7146.844	5670.374	863.4389	389.8	6290
3002	580.56	71	6463.76	3742.938	4493.876	2721.349	1441.083	6222.328	2897.328	4047	5124
3003	580.69	65	641.08	561.8667	947.7236	310.461	465.0022	854.1589	766.3378	692.1	727.8
3004	580.72	78	1793.1	1160.894	2622.976	515.62	351.21	1244.522	1733.76	1839	893.8
3005	580.9	85	29995.3	39658.45	39330.73	18214.39	40839.21	23200.45	25376.06	27334	32931
3006	581.04	915	637.67	249.6841	581.4259	250.0897	309.9495	393.792	297.5409	394.5	491
3007	581.24	137	5640.78	3588.497	6699.29	2324.803	4558.929	2098.412	4197.506	6465	5243
3008	581.3	68	1500.74	1063.53	963.3393	1263.57	1167.464	1607.12	1171.223	1176	1233
3009	581.3	106	2613.3	1911.673	3235.32	1392.3	2669.814	2691.975	1968.691	2338	2218
3010	581.52	27	1224.17	2481.073	227.832	1807.286	2870.2	2196.035	774.6401	237.7	2884
3011	581.69	68	1620.13	1591.472	939.7108	884.412	665.4709	838.0004	826.1593	1424	861.3
3012	581.91	86	2792.04	4366.474	4779.653	2609.888	5567.441	2544.977	4807.247	4229	5780
3013	582.2	1173	1428.63	1312.745	1713.04	926.035	1263.515	1292.73	729.6333	1563	1197
3014	582.21	185	5556.74	3940.831	2219.112	3114.17	4087.915	3532.543	948.376	1614	2360
3015	582.24	137	2355.78	1747.778	2575.593	1185.007	1648.367	543.95	2108.578	4131	2673
3016	582.3	107	675.435	1064.846	1295.602	812.7	1054.125	966.2836	915.04	959.7	1380
3017	582.46	89	11057.7	576.164	1385.581	10391.1	23190.91	673.0758	8140.339	6680	8964
3018	582.55	72	6245.73	2344.115	3796.577	2061.913	1276.6	4870.002	2456.5	3069	6182
3019	582.64	66	1054.45	2004.328	1758.488	1278.395	1069.9	2732.056	2395.727	3046	2786
3020	582.82	63	950.355	635.3238	354.6462	496.1882	198.0794	452.9857	639.2992	413.8	786
3021	582.86	83	27676.1	25027	48659.28	19530.44	32087.88	27129.39	25947.96	34137	27593
3022	582.91	86	28020.7	23944.53	46323.49	18859.1	26595.75	25744.62	23341.66	30346	27701
3023	583.21	186	2997.41	2207.724	1493.893	1487.754	2224.666	1983.891	542.5616	825.9	1496
3024	583.29	70	2304.39	1045.172	1396.369	986.5414	924.8511	1293.049	1619.304	2056	1265
3025	583.46	88	3538.3	0	255.4417	2671.074	12799.12	466.271	2870.068	3107	4832
3026	583.87	85	3795.5	8602.419	8255.77	2821.087	6221.286	1231.319	3561.337	4225	1767
3027	584.2	186	2108.64	1453.867	1091.99	1072.491	1477.804	1481.861	392.7	625.7	873.3
3028	584.39	118	6577.19	2707.873	6630.294	3202.487	2903.941	5263.47	4714.345	3908	4540
3029	584.47	1169	1763.64	1558.643	1535.039	1320.964	1772.396	1688.735	1546.165	1889	1521
3030	584.47	190	1991.14	2071.499	2365.968	4920.086	2545.912	2563.224	1391.706	2183	2953
3031	584.47	209	4178.83	3045.183	4514.421	2746.92	4061.77	3749.466	2881.154	3661	1749
3032	584.55	73	2587.41	1582.71	1722.584	1295.624	3862.995	2288.385	1748.888	2089	2778
3033	584.73	62	1292.58	1007.49	1061.444	532.312	522.6014	1109.48	666.344	1257	2411
3034	584.73	77	3085.52	806.6014	1296.112	1096.5	691.9413	1070.951	2722.597	2115	951.1
3035	584.86	83	8073.94	3023.622	8385.601	2461.179	2310.112	4754.575	5831.467	6016	5557
3036	584.95	89	8112.06	1726.235	4259.588	3292.08	6648.584	3744.243	2797.453	2274	4211
3037	585.23	105	1133.51	427.1965	1407.119	375.48	471.099	736.685	728.7579	613.2	685.2
3038	585.29	62	2342.69	1326	1421.443	1050.436	1061.941	1472.54	1028.231	1284	1669
3039	585.39	118	1559.81	1112.068	1255.254	2527.676	2012.8	3219.683	1758.96	2650	1591
3040	585.47	191	1116.41	903.2747	1143.715	1000.792	1069.34	1043.699	1019.011	958.9	1017
3041	585.47	210	1549.51	751.9238	1699.688	1337.953	1537.056	1061.918	1283.99	1528	1295
3042	586.45	120	14117.2	8840.043	15825.79	12281.38	11417.97	19892.83	2411.217	13498	16564
3043	586.73	76	6854.7	3083.675	1981.354	952.9904	2372.931	1514.848	5171.167	4456	1861
3044	587.36	112	1680.21	1979.858	1656.204	1509.541	1522.437	3848.987	4186.213	1414	3143
3045	587.45	120	6551.78	4794.924	2661.974	4256.856	4622.437	1436.593	5312.233	6184	6500
3046	587.71	71	1727.6	591.6318	754.9369	541.8245	335.7038	674.5347	1135.654	1019	357.9
3047	588.41	116	1587.25	623.5152	1829.085	1835.178	1272.67	1955.571	915.624	1837	1302
3048	588.73	64	4922.83	714.16	2143.858	994.9145	1019.846	1155.214	1656.096	5793	1589
3049	588.81	79	4078.08	3816.726	4909.846	2153.92	1725.42	2244.22	2813.667	4271	3670
3050	589.32	139	17124.3	8042.683	22230.17	11314.35	14686.83	14138.99	17172.49	22620	11819
3051	590.33	140	7646.18	3913.815	11165.09	5046.403	5916.072	5925.889	7401.725	11686	5140
3052	590.39	105	3479.04	3006.619	3195.05	3172.095	3090.5	4018.804	1349.657	2453	3439
3053	590.43	122	2727.5	1753.504	1558.696	1830.743	4217.355	3285.59	2062.618	2515	3558

3054	590.68	74	1591.31	1456.917	1462.801	422.5	428.1301	498.8317	1420.444	1205	497.8
3055	590.8	81	1502.05	1018.969	3208.611	1026.006	512.372	980.164	1498.077	2467	742.7
3056	591.33	139	2017.15	1411.511	3293.032	1767.932	1959.575	1791.712	2222.003	3098	1513
3057	591.39	103	1979.58	1389.873	993.9636	1735.02	1703.184	821.315	1736.916	906.6	1388
3058	591.42	123	2350.41	1020.27	1279.562	1194.064	3345.463	1863.003	39673.71	1683	961.4
3059	592.32	110	2567	4311.252	5262.507	4139.279	3471.302	3442.189	3911.987	3808	3598
3060	592.39	61	948.847	1740	1832.933	1531.082	1226.551	1800.261	1195.518	1322	1461
3061	592.67	76	5277.41	678.1416	1857.484	976.7004	702.4093	703.0154	1128.96	2524	1922
3062	593.33	110	1994.95	2129.342	1655.61	1818.237	1630.881	1583.1	1045.345	1058	1584
3063	593.67	73	1746.98	1400.524	601.29	1140.234	456.3351	693.0727	974.988	1369	2196
3064	594.76	62	3449.34	1231.543	947.036	868.806	483.6567	1642.449	1201.905	1434	2068
3065	594.76	77	3182.1	2187.525	2263.058	1963.453	885.2478	3207.432	3261.162	6504	1375
3066	594.92	84	6813.94	8384.04	7137.839	2038.598	10147.3	3128.216	5302.155	1537	6767
3067	595.37	119	10467.9	2559.808	9913.037	4620.421	3555.617	7645.839	4927.973	6749	6891
3068	595.66	73	1861.84	1630.29	1660.568	1410.585	785.2461	2579.394	1382.543	1461	1236
3069	595.92	86	5893.16	5284.495	4341.308	2559.934	3031.263	3872.227	4394.279	5028	4376
3070	596.04	287	624.389	189.7056	559.9838	95.42933	78.52852	177.528	169.1882	263.2	345.7
3071	596.04	247	3082.49	537.1604	1740.88	635.1664	632.5012	927.189	720.9772	1168	1036
3072	596.37	118	5412.47	1746.456	4086.991	2492.944	1433.703	4370.744	3286.854	3337	3453
3073	596.53	70	1132.12	464.2833	1151.622	652.722	102.6408	853.4599	750.1525	870.9	1569
3074	596.66	73	1895.61	835.6267	788.0004	391.2913	465.2889	175.7573	1414.578	1541	642.7
3075	596.75	77	8716.97	3381.272	2642.502	1702.626	1257.399	2280.984	5599.222	6991	2510
3076	596.76	62	3695.14	1594.591	1356.147	1203.334	411.2396	1534.91	1546.773	1603	2487
3077	596.87	83	8079.28	9606.829	15075.11	5618.458	8476.749	8284.842	7390.925	9509	8048
3078	597.34	63	1772.43	1371.92	1116.513	1088.109	1374.232	1646.453	1151.462	1400	1226
3079	597.38	117	7954.24	6213.379	7110.283	3995.627	4444.854	7465.689	4408.74	5067	5575
3080	598.07	915	1079.48	581.3429	1225.704	586.3163	624.8177	738.6505	691.8422	896.3	906.8
3081	598.39	118	6685.55	3664.516	5059.087	3072.685	2044.017	3472.144	1717.393	2735	2387
3082	598.49	235	1484.13	744.8502	1787.912	1430.07	1931.061	1449.17	2804.97	1647	1335
3083	598.75	77	4089.98	6339.043	788.6322	4438.788	664.3	3019.968	3115.593	5518	4263
3084	598.84	82	6470.21	5815.957	2306.166	4350.24	1421.464	4634.871	5794.867	1519	5541
3085	598.96	88	2658.05	0	0	2729.429	2252.211	2510.577	3792.108	2875	2248
3086	599.39	74	16630.2	11848.33	19128.75	19606.2	16675.73	23293.01	18593.14	22352	15359
3087	599.4	118	16602.3	5998.967	21588.18	7609.787	5730.089	11603.88	7722.24	11844	12819
3088	600.39	75	5969.88	3134.852	5421.553	6756.278	7941.564	4594.59	6944.528	7137	7184
3089	600.46	144	6637.38	4641.895	6519.997	9771.56	11792.39	10284.74	11117.34	10833	10201
3090	600.71	63	1138.51	648.2971	856.1867	264.3667	169.0217	738.3514	728.2775	2540	914.1
3091	600.86	83	1717.87	1745.237	3100.558	1533.133	1537.628	2024.133	487.487	1766	1481
3092	601.41	117	7666.65	4218.024	7331.486	6603.366	5065.78	3376.308	5065.206	3110	2972
3093	601.47	144	3776.17	2364.135	2192.321	4651.057	3795.859	3577.525	3260.94	2395	3824
3094	602.41	115	3791.41	2126.714	4803.618	1372.167	3577.493	1987.787	1338.923	2019	1387
3095	602.57	73	3085.49	2724.831	1733.843	1054.673	1004.737	2401.931	2607.435	3493	2734
3096	602.7	77	3242.16	1598.256	1637.175	1047.734	496.64	1087.554	3245.48	4113	1072
3097	603.4	112	16298.6	14298.36	17147.41	13826.45	13829.14	8360.69	8833.66	5572	4607
3098	603.52	115	3198.94	1794.493	1212.273	1429.607	1782.674	1129.099	1215.45	592.9	1059
3099	604.36	140	4711	3538.955	3792.898	5326.488	3249.402	4712.063	3079.716	5500	4066
3100	604.4	112	6353.27	6264.72	8140.574	8025.428	5382.311	4231.918	1746.549	3294	3492
3101	604.44	87	11581.4	611.2385	235.3467	6092.5	15041.2	402.2624	4503.845	4243	5447
3102	604.57	72	9295.05	6429.754	4716.589	5517.072	3603.426	7667.292	6813.366	6271	7476
3103	604.79	78	6049.63	3386.674	4801.229	1817.38	1137.139	3536.452	4212.291	7151	3134
3104	604.79	62	5278.6	1900.352	777.784	1208.194	361.3843	1368.734	1581.421	1689	1978
3105	605.31	271	1987.51	1770.836	81.31423	83.01462	924.1364	1158.244	81.92518	100.3	123.2
3106	605.33	110	1663.93	860.4182	2744.299	1673.063	1691.002	1288.249	1275.928	2661	1207
3107	605.37	140	2224.04	2154.343	3278.045	3492.2	1133.398	2515.92	2590.202	2237	2992
3108	605.44	88	4533.3	758.6844	371.0805	1771.812	5786.091	0	1279.168	1613	2321
3109	606.36	116	1249.31	2834.91	1037.294	2498.839	3380.245	2648.553	1208.513	2180	3180
3110	606.36	140	1008.14	957.7219	947.485	729.905	823.875	834.5336	1019.34	857.8	1217
3111	606.38	70	3782.2	6078.07	3460.78	6179.014	3360.006	7782.595	3092.3	5665	3913
3112	606.57	71	11894.1	8868.981	6980.349	8329.954	5159.7	10125.73	9558.203	9245	10102
3113	606.78	79	6622.85	2629.624	5984.694	2073.521	1124.376	2835.43	6052.915	5632	2612
3114	606.78	61	4114.29	1538.853	1487.387	1145.469	435.68	1163.385	1430.28	1684	2228
3115	607.39	70	1064.94	3858.391	1025.993	2475.647	2149.982	1701.336	1207.751	2398	2214
3116	607.39	1076	1048.59	771.236	1709.015	564.6455	1745.64	1925.033	625.742	1333	627.4
3117	607.39	1053	816.873	833.3695	1616.632	776.416	2091.39	1008.947	882.0438	1337	691.8
3118	608.09	706	1559.98	598.578	635.5316	764.5896	844.9699	778.3353	1933.664	1080	5327

3119	608.09	727	633.55	324.6851	761.5468	342.9779	414	396.4136	313.56	505.7	580.4
3120	608.37	111	1762.53	1542.137	1560.615	1930.707	3224.805	1200.783	742.9867	1082	1528
3121	608.38	61	4039.51	2406.501	2176.411	2824.555	3563.263	3797.164	2804.634	2615	2836
3122	608.57	70	11064.5	8996.182	5601.724	4727.198	4059.375	9152.528	6954.816	9228	9810
3123	608.78	81	3176.25	1689.559	1768.663	1061.612	2075.71	1016.009	3223.538	4741	1081
3124	609.38	61	1285.88	714.6248	1153.938	1023.825	887.4154	1144.167	946.9403	722.5	864
3125	609.93	87	3879.78	1636.275	7304.43	1321.703	5235.806	3203.03	4937.7	5233	4475
3126	610.18	8	1433.11	1658.897	1740.379	6714.975	2264.694	1865.386	761.5394	1880	1649
3127	610.18	472	2534.28	2459.71	4694.24	4352.346	3599.873	3286.877	2783.708	3637	3350
3128	610.18	140	4810.08	3573.936	5573.129	28905.13	7070.18	3631.382	2965.263	5594	5238
3129	610.18	1076	2047.2	2056.54	2230.841	18073.38	3396.166	2701.516	2773.687	2475	2290
3130	610.18	204	2885.96	2265.983	3131.703	15054.65	4375.821	4345.353	2585.474	2707	2988
3131	610.18	1052	2667.51	2833.208	2570.613	7930.288	4653.519	3939.772	3598.31	3229	3035
3132	610.18	82	3339.76	4969.548	3578.099	17873.44	10238.25	6257.384	4168.194	12335	5507
3133	610.18	233	1467.43	1201.565	1700.259	12935.12	3260.708	2295.58	1092.77	1668	1680
3134	610.18	123	5921.12	3558.75	10177.94	18635.16	5599.322	4759.002	3476.147	4266	6521
3135	610.18	39	2796.1	2349.892	3010.893	19989.83	3785.893	2814.445	2005.272	3050	2968
3136	610.18	98	4211.61	14802.02	5560.083	28608.5	8200.402	4748.69	1194.679	5188	6497
3137	610.56	71	4929	3910.361	3343.456	3947.759	2756.478	5367.877	3808.558	4573	5025
3138	610.73	78	3175.98	1312.774	1844.843	1090.116	389.12	1587.024	3046.972	3866	606.4
3139	610.75	63	1338.63	700.264	918.393	678.2309	231.4178	733.5244	604.9867	833.5	1288
3140	610.89	83	2521.3	2921.469	1797.249	1271.121	5331.949	2124.692	2548.939	1527	2211
3141	611.07	77	1633.28	1962.268	0	2651.514	42.108	3342.016	1883.783	1145	1329
3142	611.18	85	2970.02	1539.597	2579.605	23882.76	5963.368	2013.253	2772.546	2428	1670
3143	611.18	1053	1720.4	2052.155	1771.403	6038.235	2427.647	2324.948	2471.95	2433	2180
3144	611.18	1176	1374.94	1260.3	1161.729	1658.063	1453.32	1930.926	1798.52	1653	1247
3145	611.18	1088	1269.69	1224.108	1292.047	3541.316	1793.103	1458.726	1176.484	1335	1389
3146	611.34	70	3482.62	3242.203	1888.17	3246.233	1638.249	2995.353	1617.766	2785	2359
3147	611.35	119	6299.32	4457.3	7165.292	2874.937	3684.277	3666.136	2334.78	2721	5665
3148	612.18	95	3140.3	6364.054	3164.813	14614.56	4401.691	3291.974	2385.344	3440	5930
3149	612.18	118	3328.75	6149.219	2074.17	10711.16	3296.885	3412.6	1737.176	2588	2815
3150	612.18	1051	1804.88	2100.226	1874.079	3134.626	5469.304	2382.998	1933.838	2145	2110
3151	612.34	70	701.374	650.43	529.5375	694.9654	761.8733	723.4734	711.375	989.1	705.5
3152	612.36	119	2598.75	1614.756	1719.483	1634.463	1613.435	2216.63	1734.554	1774	2497
3153	612.56	71	2519.86	1759.072	1313.128	1731.707	1076.4	1622.368	1822.508	2185	1368
3154	612.73	64	896.55	349.8	2340.188	398.9877	358.8307	448.572	422.7711	843.5	1361
3155	612.85	82	1814.33	2046.77	3846.813	1536.018	256.186	1129.867	2507.56	1732	2258
3156	613.06	78	1551.55	1144.65	47.7374	1956.093	2317.604	1842.388	1814.115	1473	1206
3157	613.16	893	13443.6	4519.281	6798.064	3780.976	6056.248	3785.84	5281.651	7355	10486
3158	613.34	63	2312.98	1108.918	879.3862	1325.408	1091.418	1563.79	1199.305	872.3	1117
3159	613.36	115	2756.75	2395.05	2750.021	2233.752	3523.235	2597.173	2293.206	1670	861
3160	614.16	893	3583.67	1296.141	1871.708	1081.115	1624.335	1135.303	1614.846	2139	2965
3161	614.38	118	4950.61	2645.36	6616.846	2791.532	7223.905	4043.241	4077.335	4325	969.1
3162	614.48	121	2923.16	1473.347	1894.676	1475.817	2546.278	2645.985	1855.4	1929	1594
3163	614.48	163	6517.41	6307.238	10181.81	5578.524	8028.168	6913.763	4927.325	7094	8456
3164	614.73	76	2219.95	1162.17	1446.842	802.2408	830.4191	2562.651	2627.278	3944	1932
3165	614.82	80	11662.6	6169.122	14177.52	6418.443	5531.487	7039.413	10416.43	12128	6414
3166	614.98	73	885.168	1372.827	103.4011	1910.52	321.811	740.05	179.8125	0	1308
3167	615.16	893	1795.52	666.3905	1027.689	597.1324	914.1567	589.9826	772.6587	1005	1530
3168	615.39	119	2954.54	1933.2	4915	2238.799	2062.06	2664.289	2299.884	2811	4465
3169	615.48	163	2556.92	2133.781	2076.518	2430.652	2929.392	2701.91	3503.466	2573	2668
3170	615.82	79	2821.71	1793.513	1718.587	770.125	256.2875	1001.163	1736.625	1777	1294
3171	616.36	118	1480.23	1396.448	3517.069	3206.559	2019.164	2692.255	1181.433	2716	920.9
3172	616.46	112	7142.54	4887.855	8671.813	6115.36	11316.01	8063.595	6581.707	7207	6964
3173	616.6	75	1822.89	1141.501	1919.586	630.3994	713.4154	944.0269	2351.347	2275	1426
3174	616.82	80	8535.79	4586.531	9227.641	3335.466	2615.746	4167.743	3876.59	6650	4914
3175	616.96	73	31.2343	1534.752	0	1163.082	157.1444	702.6914	103.7157	103.5	1628
3176	617.33	105	1678.35	878.2061	847.125	671.384	950.223	862.185	900.2488	1020	1103
3177	617.36	141	966.749	806.9489	1522.41	1672.59	767.8	1213.023	1370.554	1317	1030
3178	617.46	112	3027.14	1696.596	2376.192	3212.138	3494.653	3140.136	3316.693	3922	3443
3179	617.93	72	162.656	1476.116	60.19429	1057.794	320.7824	749.3048	199.5543	29.77	1127
3180	618.34	140	787.925	754.776	434.7074	2141.868	1536.243	890.3435	696.01	1799	1153
3181	618.44	112	619.476	1505.696	1046.25	1210.674	1155.695	1154.385	913.8003	850.8	681.7
3182	618.55	71	2752	1955.952	1555.672	1526.307	1218.217	1796.442	971.046	2363	2813
3183	618.59	67	1887.72	910.45	1060.152	847.6652	1072.024	907.302	2330.9	2390	679.9

3184	618.8	80	2068.27	2024.334	1539.64	1026.006	256.592	3052.009	1799.68	1470	2070
3185	619.37	140	2074	1048.461	2051.287	1706.283	1204.29	1552.563	1700.759	2505	617.6
3186	619.93	72	69.0767	1547.616	124.64	2268.722	550.885	503.8095	170.3	153.7	1545
3187	620.37	67	872.546	1181.828	706.9147	1096.185	908.1445	1088.179	1069.075	1130	1012
3188	620.41	89	6689.59	1113.185	412.4873	2698.338	6969.06	843.0703	2252.422	3282	2165
3189	620.54	72	8641.95	4283.864	4249.135	5528.933	2277.819	7083.224	6537.594	5870	8155
3190	620.76	62	1956.85	816.536	934.0875	437.3751	276.3941	846.1125	650.36	761.7	1074
3191	620.77	79	2715.49	2582.317	1936.683	1296.67	622.81	2073.495	5839.36	4677	2350
3192	621.27	106	999.713	255.0441	3049.744	216.72	554.9867	345.125	437.262	694	719.3
3193	621.31	365	4058.01	5392.516	5161.221	6260.449	5526.366	4737.033	5455.035	6598	5875
3194	621.42	110	1770.66	1174.255	1356.533	1324.26	1668.164	1124.815	2250.035	1871	801.5
3195	621.92	73	0	2021.376	227.9649	2535.244	597.9829	1464.904	161.13	411.1	2457
3196	622.31	365	1489.41	2070.352	1912.896	2434.02	2147.302	1975.229	2168.171	2345	2141
3197	622.54	72	10662.4	7795.616	6708.269	7506.99	3393.54	7802.39	6685.769	8696	10351
3198	622.76	78	6661.98	2140.447	3293.882	1824.302	1059.633	2807.204	3430.4	4820	2097
3199	622.76	62	1631.95	1054.164	665.112	504.3988	179.4	719.0229	611.576	928.2	1419
3200	622.89	73	25.9714	1327.171	91.82833	601.1433	796.4457	669.1423	1075.2	349.1	1299
3201	623.4	119	2544.42	998.9308	2245.253	1060.402	1353.916	1399.973	1645.56	982.8	1329
3202	624.41	118	1428.93	539.0333	2178.985	713.0689	731.914	360.185	1139.797	836.7	929.4
3203	624.54	71	10666.1	6179.53	5180.825	6301.405	3489.94	8782.004	6162.366	6183	8791
3204	624.54	158	1134.24	813.4811	282.1429	1857.709	1669.68	1620.062	485.7932	334.3	2035
3205	624.62	72	2652.19	1176.504	1400.496	26.1	294.5609	127.0448	1292.813	1612	801.4
3206	624.84	82	25731.5	22449.97	29344.99	8427.583	25858.17	17424.83	20871.84	25416	26049
3207	624.88	72	248.573	2063.48	238.16	2579.921	1027.158	1541.612	536.9586	796	2358
3208	625.4	118	1643.47	1403.375	3151.037	1546.585	1444.229	1689.978	1684.073	947.5	1469
3209	625.52	1092	1289.77	2087.422	826.4274	2079.125	1605.022	1627.857	266.1584	1470	2129
3210	625.52	1145	975.204	1198.736	566.8914	1064.177	961.0596	1177.024	189.4086	1175	1483
3211	625.52	1173	1153.04	1841.917	626.3832	1305.692	1178.667	1867.96	277.8975	1305	1806
3212	625.52	1052	2273.63	5789.082	1527.181	1710.073	3100.833	3465.995	476.8866	2448	4961
3213	625.52	1195	662.86	1637.672	547.155	849.2587	1370.586	834	214.802	868	1360
3214	625.85	83	4368.67	3223.628	4088.897	2903.924	1842.54	2244.173	4480.437	3547	2154
3215	626.48	88	5151.17	125.8733	0	3935.995	12135.06	260.8459	4185.029	2057	3312
3216	626.52	1049	1462.01	2976.789	881.3497	1611.569	1648.421	1795.035	562.9352	1352	3250
3217	626.53	73	6090.9	4475.933	3054.065	3699.326	1906.084	6344.323	3695.485	5360	5778
3218	626.85	82	13285.1	11217.16	13110.28	5957.468	8497.431	6835.566	8889.802	10167	11164
3219	627.31	73	1083.91	749.7314	1445.118	1619.427	1151.917	1053.82	1034.003	1465	1113
3220	627.53	2	0	464.831	0	160.965	0	652.1867	686.2598	1025	259.7
3221	627.53	94	4055.24	2866.055	2104.218	1799.546	4355.82	4301.222	2945.268	2153	1616
3222	627.53	122	2009.86	4942.609	1747.587	944.7229	755.28	3909.972	2402.958	4640	2582
3223	627.53	1098	3472.49	2174.371	1599.455	539.6	3576.516	3132.489	1539.917	2142	1417
3224	627.53	1051	6342.42	3707.348	3550.563	789.549	2370.871	5911.034	3444.687	7150	3985
3225	627.53	1073	3737.86	2949.354	2130.98	543.48	3012.014	3823.403	1884.231	2477	1517
3226	627.53	469	1919.54	1432.031	3213.593	271.9621	1768.998	1890.869	1565.619	2575	2547
3227	627.53	1185	4372.82	1645.204	1489.873	455.2083	2624.14	2786.608	1210.572	1834	1094
3228	627.53	208	1238.86	1094.751	5302.424	797.6067	574.8126	1126.389	4209.604	6023	1252
3229	627.53	232	1060.65	813.1656	6100.349	491.1175	502.3849	707.0556	1983.636	6543	767.8
3230	627.85	75	1589.28	1664.386	569.64	1121.583	1494.552	1353.35	1754.71	1306	1806
3231	628.19	42	782.302	906.8027	269.7887	1603.029	1376.431	879.6314	155.6613	201.6	1043
3232	628.19	311	5196.26	3035.293	2837.281	3658.943	2201.692	2694.833	925.2312	741.8	1109
3233	628.5	208	2590.15	1143.824	3195.21	1701.507	2681.741	2663.92	2313.247	3155	2064
3234	628.5	186	1880.01	1518.005	2286.157	2181.818	1832.17	1943.443	1934.02	2331	2377
3235	628.53	129	860.795	1205.755	3698.266	2457.239	1101.406	898.7022	1672.02	3471	2369
3236	628.53	71	2905.43	2136.362	1571.993	1626.828	908.7	2568.39	1721.767	2088	2840
3237	628.54	1051	3646.54	3677.416	1981.2	1015.441	2749.823	2772.528	1972.438	2354	1443
3238	628.54	1076	1892.69	1201.089	1059.548	804	2061.868	2114.145	901.7393	1250	1041
3239	628.54	468	740.734	494.6423	1093.393	152.608	786.6127	592.5018	684.1058	887	747.8
3240	628.7	76	1822.4	445.1895	853.7379	709.5367	498.972	893.2771	1962.386	2168	777
3241	628.85	81	4829.8	941.7111	4775.146	1593.8	1292.588	2482.882	2524.362	2854	2513
3242	629.19	311	3040.74	1918.756	1678.015	6807.774	1510.607	1678.538	618.8889	509.9	745.4
3243	629.5	210	1292.72	497.8824	1018.705	1004.436	1295.661	928.3163	1122.773	1096	857.2
3244	629.54	1078	2067.85	1608.777	1061.174	1663.951	7433.813	1534.694	866.427	1229	2133
3245	629.54	1050	2277.84	2119.981	1347.364	1509.681	2468.909	1598.673	994.3103	1397	3766
3246	629.55	139	2554.2	1556.649	4639.933	1809.935	1951.052	2520.718	2368.271	5493	2589
3247	629.84	74	2899.92	2078.056	1023.282	1452.615	1184.681	1928.102	1391.326	1107	3606
3248	630.19	311	2356.36	1485.683	1738.071	2032.891	1246.855	1294.203	457.5957	393.1	569.1

3249	630.38	140	816.272	1043.311	1152.751	982.614	973.4258	939.2793	947.4154	968.9	878.7
3250	630.48	120	13898.8	8879.103	13866.9	11089.18	12621.23	14921.29	10450.44	12652	16185
3251	630.79	78	7716.43	5117.694	9654.162	2927.35	2204.2	4236.348	3049.877	6450	5051
3252	630.83	79	2778.75	4080.185	821.8574	2920.764	1794.274	3484.88	3229.623	2265	3894
3253	631.48	120	6467.42	3073.54	4994.016	5845.828	5108.145	7799.701	6534.892	7059	6468
3254	631.83	72	1032.86	1765.712	1062.672	1298.498	1009.323	1081.662	738.468	675	2058
3255	632.48	120	2302.89	1577.587	1396.084	1230.064	2035.999	1695.902	1786.98	686.5	2921
3256	632.57	74	2670.96	493.1537	1484.866	260.7701	92.04827	376.8741	1170.514	1972	672.3
3257	632.79	79	5761.56	2062.711	5944.555	2250.006	1861.641	3587.86	5026.101	4819	2516
3258	633.83	74	410.098	1009.853	1610.262	1063.502	989.4564	2264.258	325.194	199.1	1762
3259	634.41	105	3348.28	4478.729	5513.389	3944.775	4688.706	4845.039	1993.387	4956	4512
3260	634.45	124	2781.13	2026.75	3015.151	2228.798	2469.814	1788.739	2013.799	2798	3211
3261	634.54	100	438.55	330.644	98.033	1127.7	770.1314	1378.356	432.8088	204.4	700.6
3262	634.66	64	3413.16	527.31	1339.29	667.494	790.8373	890.4686	1305.454	2740	1032
3263	634.79	73	2201.88	2254.496	2750.645	3351.248	1117.14	1746.619	1950.362	1826	937.3
3264	634.87	85	89605.9	97225.66	106643.4	48053.07	90824.3	58278.56	73727.52	82800	79166
3265	635.14	893	1903.23	698.8519	891.4048	560.3344	1006.02	686.9475	629.8956	1101	1503
3266	635.37	71	1266.91	932.008	1053.173	1337.117	823.9241	3066.05	1573.725	1471	2084
3267	635.42	104	1428.07	1410.061	2097.261	1733.558	2071.819	2064.312	1843.307	1982	2365
3268	635.65	71	1180.4	1301.56	1211.334	1757.376	749.3581	1115.898	1183.914	2805	1079
3269	635.88	85	13672.5	17088.27	19157.3	10817.1	13760.45	11139.97	11619.23	12775	13622
3270	636.41	62	1955.79	1482.728	923.7579	1171.818	1843.193	1085.035	1521.058	872.3	1535
3271	636.52	71	3708.88	2290.026	1993.328	2922.417	605.6212	2472.346	1546.426	2465	3421
3272	636.78	73	2511.95	1210.194	972.6835	1242.8	964.5622	1401.993	1393.214	1525	2221
3273	636.88	86	6001.16	6011.887	6755.621	3328.92	8602.083	5429.743	6086.069	5913	7324
3274	637.3	136	168035	110517.2	194691.6	60142.56	158147.7	47980.5	136783.5	2E+05	2E+05
3275	637.65	72	1674.62	2031.663	678.6	1473.552	673.452	1684.649	1112.712	1765	1210
3276	637.86	81	2049.26	1509.116	1882.839	1609.658	415.9636	1004.256	1479.23	1480	1668
3277	638.31	136	65691.4	43022.11	70299.63	23636.12	57093.2	19143.71	51352.04	92413	65629
3278	638.33	365	3406.08	4128.493	4182.125	4879.654	4458.173	3862.37	3939.886	5095	4195
3279	638.51	73	4739.58	2787.561	3486.269	3858.134	1522.981	4513.152	3598.524	3195	4538
3280	638.65	71	2126.8	1939.312	1679.145	593.4542	751.5557	882.8616	1760.304	1220	761.4
3281	638.76	76	1660.66	2652.164	864.2205	459.24	1169.117	976.7065	2007.18	2684	1028
3282	638.86	82	8640.62	8143.701	8900.459	4969.134	9398.693	5192.255	6327.916	7563	8196
3283	639.31	136	15898	10328.31	16198.43	6080.271	13355.98	4554.077	13472.81	24501	14677
3284	639.34	365	1333.38	1489.257	1671.261	2116.296	1486.09	1694.707	1832.221	1954	1788
3285	639.37	103	652.68	823.8683	832.4333	1158.364	624.402	1018.384	1136.636	1229	1173
3286	639.86	78	1268.9	774.822	1295.361	345.44	2151.798	257.404	780.1452	254.6	1126
3287	639.94	86	6790.59	1998.593	6872.857	1521.216	2378.417	2070.644	4741.394	5196	2242
3288	640.31	136	3051.52	2045.376	3756.982	1186.046	2431.641	863.412	3654.709	5359	2273
3289	640.43	120	951.18	99.17143	1334.647	704.5004	590.6193	480	775.3633	1474	1136
3290	640.51	71	5164.65	2580.248	2139.013	2843.988	1122.008	5793.424	1930.145	3614	4198
3291	640.6	74	2387.88	568.1751	1271.678	186.6572	357.7385	468.6786	840.96	2138	659.6
3292	640.82	81	13895.5	10561.29	17674.38	6909.279	3591.022	8458.614	2701.306	11225	8817
3293	641.15	80	4463.93	3474.247	3180.828	2300.882	5974.452	1450.02	1514.873	3292	1517
3294	641.35	140	2745.28	2137.111	777.48	3288.813	1934.308	2442.398	2501.03	2603	3776
3295	641.78	76	2795.19	1417.538	2591.971	2165.76	645.9933	764.8613	2049.141	2290	1547
3296	641.88	85	1807.21	1711.516	2707.634	1886.525	1023.888	1405.163	2545.444	2240	1960
3297	642.36	140	1525.4	1569.924	1347.653	1496.48	1193.201	1347.782	1723.5	1450	1448
3298	642.44	115	934.373	1515.15	1802.309	1816.643	1346.691	1240.763	979.4156	622.9	1256
3299	642.51	71	3286	1107.308	1843.281	1318.546	874.9038	3189.161	1993.002	1723	2286
3300	642.51	233	1743.43	669.6291	1585.479	1666.065	1765.658	1531.437	1465.138	1613	1525
3301	642.69	63	1527.87	439.89	788.3167	612.0302	437.2996	768.306	753.84	1081	981.2
3302	642.82	82	7512.34	3168.929	8771.824	2820.554	3131.629	4056.118	5274.937	6713	5033
3303	643.29	364	1937.65	2863.72	2514.882	2528.069	3099.803	2652.866	2794.215	3232	2849
3304	643.37	139	1819.24	1559.262	1958.189	2325.777	1310.811	1482.105	878.7044	2248	2341
3305	643.8	79	2194.44	1589.179	1762.813	1318.096	243.9454	1356.162	928.6715	1329	1582
3306	644.29	363	832.163	1241.479	880.1566	1209.493	1320.581	1097.271	1099.482	1134	1134
3307	644.37	141	1153.3	968.375	804.9433	979.17	766.3106	817.1046	1474.068	1248	1259
3308	644.45	85	1005.84	2687.965	1281.611	1785.626	4866.12	512.2367	1021.427	515.2	1248
3309	644.49	144	8520.64	5957.205	6407.15	6198.01	9513.72	9223.183	5726.534	9670	7308
3310	644.69	76	2585.16	2198.924	3255.061	1098.111	1468.16	2375.962	3626.285	4224	2396
3311	645.39	140	2639.35	2931.485	1192.893	2730.933	2156.04	2353.537	3221.299	3732	2480
3312	645.49	143	4240.76	2291.119	3131.754	3141.797	4471.269	3359.523	3618.898	3841	3380
3313	645.89	82	1988.51	4750.54	6189.609	2817.36	2310.592	3074.2	2768.207	3550	3247

3314	646.39	140	1522.44	1521.874	1872.072	1597.96	1267.962	1249.837	1422.513	1543	1566
3315	646.68	72	4218.97	854.3467	1849.548	1239.197	995.4462	1111.11	3533.853	2960	1117
3316	648.76	76	1924.81	1891.021	2414.17	891.1641	528.5925	1035.268	1159.412	2439	1056
3317	648.89	85	24717.2	27148.37	31283.18	16009.31	29296.22	16477.83	22103.03	24282	24080
3318	649.89	85	4448.72	4017.273	6942.803	3929.678	4163.8	2368.148	3800.551	3709	5255
3319	650.41	69	1608.72	2898.392	1251.244	3328.721	2160.271	3372.304	1329.093	2913	2143
3320	650.63	75	2119.73	1242.25	1284.358	679.1625	525.19	740.4388	1806.251	2201	723.6
3321	650.85	84	26667	1500.66	37591.56	17579.25	25207.28	18036.96	25332.33	24221	24160
3322	651.1	893	676.701	356.2721	435.6575	246.3188	466.6781	238.672	310.9871	384.9	721
3323	651.85	85	4027.17	3702.2	5132.196	3561.913	3898.219	3078.485	3001.075	4250	4929
3324	651.95	86	2526.08	2902.998	2309.349	2170.569	2784.6	2415.751	1529.44	4286	2127
3325	652.22	136	561.771	1035.05	597.552	281.385	797.9314	230.685	848.064	871.1	764
3326	652.41	61	1945.11	1137.109	1809.6	1545.546	1899.844	2068.456	1675.925	1719	1090
3327	652.71	79	1742.46	550.9913	731.2361	1037.591	234.83	624.0296	2930.543	2279	521.7
3328	652.85	83	5008.6	6443.8	7699.462	2291.87	5508.192	4125.567	4311.12	5256	3016
3329	652.93	89	5845.99	1535.631	2052.344	1520.267	6386.209	1016.25	2292.411	1543	1020
3330	653.62	72	1528.46	1556.401	654.08	806.0564	700.7	1096.195	1222.995	1119	2894
3331	653.85	84	1521.97	1996.725	1245.304	1278.709	1024.28	1168.2	983.7254	2032	2772
3332	654.33	136	176711	117229.4	210024.7	66748.99	147886.6	50612.31	138099.5	2E+05	2E+05
3333	654.49	74	1060.67	577.3983	951.4464	892.0772	482.3164	1465.326	901.8215	871.8	1102
3334	654.67	77	1701.23	952.0754	603.3956	60.63	237.4938	640.5088	1131.987	5726	1059
3335	654.71	63	3845.38	956.97	1511.911	661.3098	683.2315	1211.31	1164.282	1315	1877
3336	654.84	81	4029.61	2101.05	7105.918	1544.825	1793.273	2644.475	3046.079	3553	3040
3337	655.31	97	8879.66	8789.103	5799.665	7133.42	8203.623	6319.374	5436.774	13566	12920
3338	655.33	136	63364.6	45355.66	82941.26	28343.59	51841.17	18241.64	52194.72	1E+05	67690
3339	655.37	70	1092.19	968.584	784.8045	1090.425	1973.535	974.2079	998.985	1551	1214
3340	655.43	112	1691.69	1386.45	2908.876	1723.571	2464.157	739.4215	1530.717	960	831.9
3341	656.22	310	4958.3	3009.114	2994.574	3548.438	2254.295	2881.01	796.995	740.4	1266
3342	656.31	98	4733.06	4809.862	3058.435	2409.716	3503.167	1953.888	4664.53	5544	5437
3343	656.34	136	16089.6	11354.54	19964.97	6672.111	12027.75	4208.342	14603.85	22610	18136
3344	656.44	114	1001.19	1206.55	857.6653	1099.232	697.2627	1122.489	948.2056	728.1	194
3345	656.71	77	5375.18	1790.517	2283.014	495.68	590.733	1080.87	1876.93	3615	1626
3346	656.71	62	2289.67	971.6667	1063.876	615.913	260.0222	1051.597	934.8625	859.4	1181
3347	656.8	81	4623.31	2049.197	2767.792	3110.189	2019.303	3037.84	2755.33	4737	2647
3348	657.23	311	2996.39	2048.077	1891.807	2319.814	1688.216	1847.947	554.2939	541.9	846.2
3349	657.34	137	3979.03	2137.41	3172.328	2235.373	2988.888	1098.726	3339.001	5344	3800
3350	658.22	312	2225.51	1353.355	1397.627	1427.31	1170.689	969.6422	456.3925	405.6	644.8
3351	658.49	123	1434.35	913.425	1240.066	1738.002	1719.453	1862.336	1737.54	1709	2127
3352	658.51	161	5086.86	3382.404	4761.739	4812.699	5759.061	5778.516	3249.428	5563	5538
3353	658.79	81	1863.68	1548.13	3642.169	822.358	1517.416	1387.991	2267.044	1860	1554
3354	659.26	365	1450.46	1812.95	1604.448	1752.18	1936.292	1846.691	1991.229	2294	1847
3355	659.29	136	39062.3	23738.42	35564.92	16714.45	20647.58	14605.08	24828.7	46609	39780
3356	659.46	112	1568.4	1113.117	1222.421	1632.289	2905.275	782.6743	652.7533	428.5	1015
3357	659.51	161	2193.45	1505.792	1915.758	1822.66	2216.059	2454.49	1585.242	2189	2193
3358	660.29	136	16394.9	9573.018	14624.07	6863.161	8383.473	5759.572	9891.556	18588	16177
3359	660.36	140	1005.35	9840.96	14542.15	625	8252.333	947.8935	1103.476	1664	15616
3360	660.49	111	6294.07	4159.878	8388.424	5369.87	7836.726	5548.625	3960.979	5568	5354
3361	660.68	63	891.633	616.17	2019.124	305.5235	749.523	881.28	1128.32	1440	723.7
3362	660.72	77	1216.53	1145.719	2482.162	892.4429	452.1086	679.6638	1586.585	2280	1534
3363	661.29	136	3388.26	2603.5	3781.31	1873.495	2266.95	934.1475	1062.576	6069	4842
3364	661.35	105	1500.28	1038.343	1672.762	1067.22	1402.511	1652.236	1627.43	1571	759.7
3365	661.49	111	2345.6	1252.68	3279.864	2266.176	3736.6	2028.208	1716.988	3045	2441
3366	662.29	137	882.646	696.1429	638.81	461.2688	864.1928	374.44	401.5413	1362	909.3
3367	662.53	71	3980.15	3612.21	2557.984	2752.872	2336.155	4004.16	3187.674	4252	4749
3368	662.74	62	1023.41	1073.177	1343.166	898.3333	219.7282	1006.374	896.2083	1342	1488
3369	662.75	79	4986.67	1890.136	2685.28	1310.897	1095.177	2454.94	2031.82	3077	2604
3370	662.9	84	3534.1	6914.325	6864.868	3584.92	9609.909	3279.64	4587.574	3403	4304
3371	663.45	1076	1837.23	1517.505	25406.18	1394.745	21579.44	16747.25	1485.711	41968	4911
3372	663.45	1053	2741.06	1614.022	21038.58	1524.978	30535.88	13880.68	2436.062	13452	1949
3373	663.45	1185	1082.55	838.7167	9041.721	885.4764	10555.24	6490.638	1059.419	6783	941.1
3374	663.45	1152	529.383	357.7108	6648.704	365.5409	6227.512	2848.047	487.013	4248	513.7
3375	663.45	112	21307	18106.7	2219.683	2657.672	3164.664	2662.611	2320.031	3169	1960
3376	663.45	2	2944.6	1163.57	645.6751	319.5966	442.308	566.6723	475.8724	726	421.6
3377	663.45	48	21959.3	19151.45	684.8917	3541.072	2705.648	928.761	725.2595	663.9	1078
3378	663.45	1100	1071	781.0265	16324.55	729.528	11456.19	11452.89	768.6476	10540	1181

3379	663.45	209	1764.74	1882.158	1282.389	3103.08	744.9609	1674.533	2974.302	2275	1653
3380	663.46	142	6897.84	11034.54	2353.2	3083.401	1746.133	2022.163	1340.774	1915	2358
3381	663.85	85	2018.23	2252.67	1280.16	2554.803	1796.46	3440.608	2566.685	1464	4269
3382	663.9	88	6721.85	3495.24	5601.303	3054.067	2911.264	3963.691	2806.868	4070	5844
3383	664.45	1155	356.59	236.0633	2776.183	264.55	1773.391	1901.596	271.4	2147	298.1
3384	664.45	1054	1594.39	1172.453	9761.991	986.653	14541.5	7062.073	1330.446	6902	1135
3385	664.46	106	13015.8	26090.72	1674.253	1847.109	1340.039	1597.248	1267.996	1890	1395
3386	664.46	1181	445.262	313.5295	1830.772	402.62	5067.578	3377.613	554.1474	3532	481.3
3387	664.46	50	9349.09	9503.994	1311.161	2499.301	1873.098	664.8965	1527.258	1684	1231
3388	664.46	1	1694.35	621.452	501.6073	134.19	326.4538	320.2414	361.92	381.8	327.3
3389	664.53	71	5632.73	5179.778	3594.32	4455.82	2468.856	5143.349	5079.102	6218	6730
3390	664.74	77	4307.47	2187.832	2710.631	2326.082	2603.579	2110.27	4152.425	4632	2302
3391	664.74	61	3011.14	1467.063	963.8514	1023.535	232.2141	1062.875	1214.354	1416	1780
3392	664.86	83	11332.4	8349.726	12818.33	4079.453	10021.6	4536.63	7839.544	4800	8025
3393	665.45	110	2803.67	3582.247	1501.883	977.3001	2540.541	1376.78	2101.63	1692	922.7
3394	665.45	90	4672.51	4112.336	928.1435	1704.904	3335.28	888.3734	1145.184	1141	1933
3395	665.46	1101	289.97	232.29	2243.949	299.2082	1591.814	1449.701	248.4221	1453	342.1
3396	665.46	1075	865.788	713.8889	3508.812	536.127	2948.168	2777.173	575.5282	2108	569.7
3397	665.46	48	2599.12	2953.092	330.6775	770.9808	565.3435	353.0803	449.7029	271.1	431
3398	665.46	1054	539.311	365.0972	2685.521	449.504	4130.116	1982.634	595.836	2360	517.6
3399	665.86	82	2529.84	1517.284	2990.043	1278.89	1026.413	1965.713	1759.314	2273	2027
3400	666.37	364	8805.59	11493.84	9419.963	11243.99	10166.25	10247.6	9935.439	13405	11691
3401	666.52	72	6289.28	5835.396	3767.763	3984.494	2909.4	5999.089	5535.872	6275	5658
3402	666.74	63	2216.22	1006.2	851.7444	538.7409	186.736	958.4423	768.9733	1179	1758
3403	666.82	81	6302.89	1240.484	3848.545	4617.658	1794.564	4896.933	2500.805	8238	5068
3404	666.95	86	2711.19	1656.844	2562.555	851.512	1503.18	2204.416	2795.822	2786	2338
3405	667.37	365	3418.12	4640.246	4432.858	4957.575	4443.885	4118.285	4138.043	5217	4595
3406	667.83	84	1971.42	0	2048.76	767.075	256.2875	1885.845	2005.185	1978	2033
3407	667.97	73	68.8243	2664.794	1630.999	1640.157	354.12	671.994	216.15	324.5	1793
3408	668.34	137	752.25	711.8667	1996.598	465.7829	1345.813	372.9643	1193.126	1156	1458
3409	668.37	364	935.907	1083.292	967.8551	1539.356	975.1714	874.4455	1103.829	1161	1134
3410	668.52	71	4553.56	3800.763	2514.112	2598.864	1892.643	4258.039	1495.252	3391	4535
3411	668.56	94	796.938	1231.442	0	3839.097	2551.858	1934.211	57.83514	0	3009
3412	668.56	1167	3512.49	1371.238	1127.455	2536.995	3971.165	4396.544	1096.338	3882	3651
3413	668.69	77	1880.16	1553.487	1336.304	857.8943	517.0394	1434.832	2105.564	3113	2270
3414	668.87	85	5337.63	2904.3	4874.16	3579.636	4488.151	2158.912	5568.336	3723	3681
3415	669.37	85	1031.24	1246.14	2051.28	509.936	769.82	447.8917	1029.258	1784	960.6
3416	669.38	140	1790.02	1465.452	2365.028	2249.179	963.4929	1733.895	2302.264	2304	2273
3417	669.57	1167	1334.85	626.9755	1580.071	1357.611	927.4013	1788.091	1216.88	1591	1307
3418	669.97	73	68.8243	2121.392	61.57671	2271.318	607.0629	909.7752	286.89	678.6	2037
3419	670.46	139	2356.38	1868.417	1203.285	1319.867	1693.811	1618.798	1380.123	1681	956.9
3420	670.52	71	1812.35	1315.949	1030.513	1779.408	1063.502	1647.367	1501.967	1393	2009
3421	670.68	63	1397.48	448.324	826.88	473.43	334.1428	586.568	877.8917	647.5	815.7
3422	670.69	75	4904.78	1582.805	1528.713	523.8671	227.84	649.8508	2974.385	1800	1052
3423	670.93	74	2566.58	1897.948	654.0171	734.8567	646.3969	739.8813	215.3473	614.5	685.1
3424	671.47	139	702.555	1286.505	504.5332	1236.281	1480.388	880.0872	1618.581	757.9	473.5
3425	671.97	75	68.9883	1486.327	1614.511	2145.206	280.24	653.832	0	43.86	1539
3426	672.34	98	4248.78	2641.672	3284.086	3604.36	3227.841	3503.096	4447.539	5939	6032
3427	672.52	208	2156.79	926.9018	2366.482	1263.75	2147.938	2039.623	1617.098	3019	3613
3428	672.64	74	1107.55	625.2718	1097.319	408.5454	513.5395	755.0433	555.822	921.6	197.5
3429	672.77	62	2337.52	1158.327	636.2767	797.3846	237.6581	832.2	738.1468	899.8	1584
3430	672.77	79	6845.05	3098.8	3827.102	2324.1	1838.393	2493.031	3295.208	5441	1744
3431	672.93	73	174.647	2169.414	193.9011	1148.707	651.1657	1586.049	244.97	550.8	2154
3432	673.34	99	1592.49	888.7756	1478.549	1430.7	756.7047	1277.26	1913.68	2447	2847
3433	674.46	136	4289	3046.713	6792.86	4168.175	5604.09	2062.551	4971.931	4201	3586
3434	674.5	119	12390.3	8421.748	11553.67	11569.77	8899.092	14925.16	7044.355	9558	13934
3435	674.77	78	5912.2	3099.288	5161.062	1816.104	1506.48	1762.535	3226.602	5404	2879
3436	674.77	61	2801.4	962.54	813.96	795.02	384.8	1045.856	1167.529	1039	1672
3437	674.93	73	192.647	2393.907	244.001	2195.941	763.687	1516.759	330.12	243.8	2383
3438	675.26	137	17934.2	9358.417	11604.21	9265.837	5391.945	9270.733	7888.4	16142	19990
3439	675.46	137	1876.37	1750.239	2021.19	2900.037	2200.795	1262.854	2341.01	2757	2233
3440	675.5	119	5700.11	3500.161	6180.57	4042.853	2714.346	6884.945	4265.127	5106	6934
3441	675.67	468	4036.32	1129.603	253641.1	76360.5	23441.08	7381.659	16305.9	37389	36406
3442	675.88	84	2049.17	2709.106	2308.85	1393.336	945.0203	1216.247	2040.792	1760	2253
3443	676.26	137	8093.92	4030.706	5609.708	3887.345	1963.358	3076.733	3890.932	7501	8068

3444	676.51	119	1235.62	1693.938	1981.105	2071.566	1633.127	2694.846	2527.19	1927	2069
3445	676.68	468	2787.4	700.3317	130940.1	39949.69	11675.2	3936.018	7107.498	20229	18550
3446	676.76	77	3332.82	1662.286	1685.638	1811.953	1191.84	776.5765	1188.017	2542	1042
3447	676.77	63	1033.24	1004.917	376.36	419.7917	132.617	636.7917	871.72	538.3	1158
3448	676.86	84	2044.97	4635.54	3076.189	2807.719	3240.341	2580.017	2810.46	5019	3722
3449	676.93	73	181.3	1992.424	176.5294	2215.413	1039.17	1115.535	200.585	310.3	2019
3450	677.26	137	3083.8	1629.564	2960.392	1200.8	1178.564	1640.628	2211.624	3064	3111
3451	677.29	96	1788.99	1530.9	1315.032	2195.278	1809.164	2885.76	5138.638	1462	6454
3452	677.68	467	848.482	203.9247	36619.88	14283.64	3862.95	1328.793	4073.33	5560	5687
3453	677.89	82	6922.33	1716.605	2051.828	2567.679	4959.765	1979.502	4316.76	1481	1252
3454	678.3	97	1148.45	1231.174	203.31	1552.435	632.5125	1119.326	1268.484	1323	2161
3455	678.44	104	4259.63	4691.071	5189.577	5003.46	5427.062	4432.1	4335.683	4228	4243
3456	678.48	123	2198.44	1345.68	2225.689	1382.248	2652.207	2223.75	2703.127	2441	2452
3457	678.5	472	1045.03	1210.511	1077.393	869.5128	1313.496	1129.955	260.3224	4313	1824
3458	678.5	71	4565.64	3472.556	1723.972	3101.023	2157.65	4999.663	1663.524	2622	4378
3459	678.68	467	231.353	106.6838	7811.122	3149.575	911.7658	410.32	1426.706	1419	1251
3460	678.72	78	1820.47	1584.628	1504.253	1050.06	399.2463	786.1375	2348.772	2344	1583
3461	678.74	62	1428.92	759.81	602.527	390.4875	238.1773	480.4945	532.5444	466.6	683.5
3462	678.88	83	1784.62	2346.669	3179.099	2193.033	2384.182	1219.296	1765.94	1480	2880
3463	679.36	365	6744.02	8061.596	7712.064	8041.927	8541.597	7685.014	9188.523	9764	8592
3464	679.42	136	4209.49	3832.4	3328.332	4996.801	4529.008	2496.512	1573.283	5803	4942
3465	679.45	103	2949.49	1756.029	1543.771	1202.04	2392.136	1497.807	1603.595	2178	2112
3466	679.51	472	1158.66	958.9103	1245.509	1500.024	1840.163	1467.916	944.202	1889	2452
3467	679.88	73	194.786	4145.421	279.8318	1935.238	1406.72	1655.07	483.39	1054	3377
3468	680.19	137	806.546	634.6	1009.374	184.2033	572.77	249.402	704.99	842.4	884.5
3469	680.36	364	2699.47	3622.31	3177.421	3513.078	2920.886	3328.683	3844.563	4122	3765
3470	680.42	136	2427.57	2149.449	1421.718	2411.016	2073.258	1704.352	1903.85	3535	3287
3471	680.44	61	870.234	1591.488	1483.627	1358.525	1515.251	1738.741	1155.152	1294	1066
3472	680.48	2	1179.81	381.495	395.9654	111.5115	167.0729	231.073	277.2782	494.4	163.7
3473	680.48	1181	499.111	534.5485	3849.874	535.6032	4023.18	2632.988	676.62	2543	408
3474	680.48	1075	1046.1	1009.053	9076.375	976.469	8743.666	7876.806	969.22	15715	917.2
3475	680.48	1153	383.512	361.307	2639.4	361.8045	2583.916	1429.235	415.0593	2322	335.7
3476	680.5	72	9692.28	1705.136	4202.269	3951.345	2638.894	6930.728	4464.033	6433	6610
3477	680.72	79	4057.9	1293.455	2027.182	1553.467	406.1933	777.8517	3955.826	2912	1303
3478	680.72	62	2087.49	704.34	763.7467	590.8467	326.6068	571.446	863.7067	798.3	1347
3479	680.84	80	2789.12	2331.297	2319.967	1053.827	993.9375	1490.178	1495.146	916.5	1552
3480	681.43	137	1154.11	997.5107	700.7284	981.8987	1025.737	522.1795	1205.346	663.1	966.2
3481	681.47	277	190.826	169.3888	1625.847	716.4102	167.2847	205.9992	2771.195	715.2	186.6
3482	681.47	256	477.723	337.8879	3135.096	1266.025	323.8043	516.8954	5259.247	2844	397.6
3483	681.48	1053	1555.14	1655.988	3802.067	1209.756	6469.615	3952.929	1051.889	2876	2223
3484	681.48	1076	749.218	800.2735	4645.08	599.2381	4390.97	4674.367	600.1105	3002	712.1
3485	681.48	48	3899.9	4501.458	475.5253	1143.902	679.4865	608.5066	581.233	567.1	503.4
3486	681.68	467	151.917	50.42833	2750.769	1513.398	780.4143	242.0924	632.427	853	1201
3487	681.88	73	174.009	1761.141	204.646	1623.875	491.6171	1487.811	195.19	601.2	2552
3488	682.36	1104	1241.8	870.8185	1149.664	981.6433	912.5475	1017.839	1166.536	935.8	1199
3489	682.36	137	145266	130033.1	126753.1	83646.72	114712.6	59173.31	101087.5	2E+05	2E+05
3490	682.5	72	7052.52	4620.93	3738.822	4357.74	2414.1	4901.271	3815.023	5703	6328
3491	682.71	79	1218.24	1835.828	1773.946	869.8744	354.9133	782.8333	2399.729	2457	543.9
3492	682.81	76	9842.52	6656.417	11594.81	1739.765	3842.176	1879.744	7850.688	10270	2973
3493	683.36	137	63980.7	51843.72	48509.29	37708.83	50147.75	26540.34	51592.04	60115	75765
3494	683.39	102	1807.21	1368.453	923.4703	1123.92	1197.872	1257.762	1110.432	1108	1845
3495	683.86	80	3103.58	1206.127	3844.535	2213.747	1418.122	2657.488	1259.458	2877	2914
3496	684.2	139	4028.38	3377.884	3725.046	3204.829	3354.484	2411.579	2485.014	2952	9063
3497	684.2	80	2855.72	4830.493	3497.868	2412.804	1840.484	5208.048	2761.842	6036	3604
3498	684.2	472	1622.79	1417.327	1378.331	901.46	2193.01	1862.868	2038.587	2815	3072
3499	684.2	95	2483.48	4299.743	3975.28	1531.113	2034.543	3657.287	2749.383	2719	5902
3500	684.37	137	15251.9	11984.04	11681.04	8013.868	10625.45	5910.271	12030.03	16043	17620
3501	684.49	71	3965.29	3697.56	2172.112	3126.735	1406.832	3838.739	1833.03	3480	4659
3502	684.58	67	2249.45	817.782	1159.98	558.8875	495.3722	1086.797	2244.714	1745	1259
3503	684.82	78	2271.79	6303.896	9665.041	3335.466	2126.488	4573.425	8206.436	7688	4027
3504	685.2	1077	2105.5	2156.701	2012.963	1457.082	1410.252	1755.6	1416.959	1460	3943
3505	685.2	118	2852.02	3222.337	2646.132	1978.113	2639.91	2940.269	2744.368	1863	5188
3506	685.2	472	978.75	872.0579	2883.759	1989.425	2102.283	1238.963	1375.855	1610	1481
3507	685.37	136	2864.43	3303.733	2772.142	1675.18	2315.21	1315.525	2973.609	4728	4115
3508	685.43	138	2904.81	3364.134	3528.224	1932.298	2805.43	1655.851	1036.157	4503	4437

3509	685.43	48	2889.84	4488.23	350.9034	551.6886	584.4	270.0026	373.9103	396.8	556.4
3510	685.43	92	15475	16139.34	899.7711	1610.984	1309.468	545.0355	1242.633	1122	2301
3511	685.43	1155	129.789	92.092	1927.687	117.6931	2244.629	422.28	149.2822	1233	132.3
3512	685.43	1100	234.038	182.5065	4323.913	189.8867	3169.859	3109.558	242.7778	2670	288.1
3513	685.43	1076	426.115	415.7689	5317.547	249.623	5273.542	3700.023	468.6659	3249	277.1
3514	685.8	73	1102.05	2480.995	1483.092	1060.902	846.622	1611.16	835.84	1069	1434
3515	686.2	143	4146.52	2067.771	5002.398	1749.889	1534.2	1414.402	1626.262	1546	3222
3516	686.2	120	2110.77	2422.394	3874.354	1104.354	1619.031	1883.133	1890.374	1498	3468
3517	686.2	95	1316.11	2215.65	2840.085	1056.71	743.2291	1585.064	1115.372	1920	3230
3518	686.43	138	2227.36	1588.028	1126.904	1064.916	1182.37	939.6039	1366.025	1209	780.6
3519	686.44	1075	1344.78	1253.991	2782.144	1374.483	2603.201	4961.105	1658.824	2990	1378
3520	686.49	74	2990.88	1274.966	1266.16	2316.708	1149.288	1779.467	2746.821	1883	3449
3521	686.53	92	4432.05	2471.446	1787.928	3561.068	6812.815	3355.272	2087.924	1747	4619
3522	686.54	231	1441.59	603.2288	1494.692	1287.884	1303.155	1140.917	1189.812	1279	1254
3523	686.83	75	3727.12	1795.295	2417.427	2052.012	1160.403	2381.158	1973.854	3826	2766
3524	687.18	181	1871.89	583.1228	2167.231	339.822	447.373	402.363	437.4562	474.3	880.7
3525	687.5	94	1852.97	3135.279	420.425	1452.5	1909.842	2880.904	976.186	193.9	2395
3526	687.8	73	1085.53	2243.066	847.6	1952.57	1815.036	2284.8	830.7917	1445	2783
3527	688.18	181	1758.35	480.4	1340.688	292.92	217.2537	244.37	168.9557	303.6	491.2
3528	688.48	82	3245.01	3312.195	3074.4	2987.586	7083.129	558.8	1237.077	1494	1364
3529	688.52	144	3926.36	5116.575	8730.632	5248.272	8146.036	3904.274	7008.92	6888	7505
3530	688.52	129	4957.96	1276.184	4715.967	2081.131	2947.636	4104.48	3163.378	3061	2964
3531	688.61	75	1062.62	95.2723	376.3069	84.24	234.7982	204.1518	1065.281	815	458.3
3532	688.78	76	2358.52	2412.351	2807.326	1160.986	1562.661	2445.728	5930.167	3503	1140
3533	689.52	140	3542.94	2350.747	3166.38	3571.425	3181.956	3306.87	3535.291	2641	1794
3534	689.79	72	1306.14	2398.976	1977.237	1774.793	1511.666	2515.028	990.2936	1539	2498
3535	690.46	112	1335.69	2372.172	1638.868	1903.958	1400.53	688.2255	2064.935	2385	1910
3536	690.52	132	758.119	1480.031	1663.437	1462.921	2111.082	2229.433	1332.557	757.7	1744
3537	690.75	77	3346.26	2885.119	3241.214	1897.555	1642.227	2990.79	1331.737	4880	3488
3538	691.42	105	2920.39	2123.994	1043.367	1739.784	2524.459	3025.403	1003.787	1909	3387
3539	691.79	73	1549.4	2570.876	838.1505	1944.8	757.9584	2122.92	647.5556	1128	3327
3540	692.42	105	1108.91	718.861	1350.827	1531.523	1528.853	1080.954	1022.981	1144	1781
3541	692.61	63	1505.07	694.1945	627.805	236.245	180.0398	559.0009	726.1761	638	683.7
3542	692.75	74	1362.24	3153.251	2138.064	5897.666	11411.06	3685.242	3907.84	1511	4298
3543	692.83	82	19132.4	26733.62	28010.6	12747.35	20886.42	11148.33	17105.54	25812	19955
3544	693.79	73	841	1993.825	1442.19	1684.686	1807.621	1061.648	640.755	711.1	1694
3545	693.84	82	5597.56	3468.04	2543.523	2808.416	5340.11	4189.576	3515.111	3494	1851
3546	694.4	364	616.902	965.4387	653.5407	1120.958	730.3304	771.0847	969.1281	821.7	1027
3547	694.47	71	2558.03	1534.084	628.186	2098.465	787.8758	1361.405	1781.776	1106	1518
3548	694.75	73	2450.14	3091.171	2130.965	2177.19	4093.729	2371.02	1132.12	1910	2234
3549	694.83	82	10987.4	9739.325	10383.61	4608.792	11131.48	5241.537	7269.009	11206	10350
3550	695.36	137	63351.3	28534.01	45138.2	25502	19553.85	25784.21	50856.97	64366	54766
3551	695.41	111	2250.69	2635.734	2031.555	1344.42	1650.107	1407.681	2547.765	1711	2584
3552	695.7	72	770.9	677.2213	608.1683	445.778	433.866	634.6677	399	662.4	998.3
3553	695.84	79	1988.89	1793.937	1976.226	513.634	1083.696	2377.584	2467.169	1228	1520
3554	696.36	137	26998.6	13522.08	19887.89	12016.38	9302.595	11339.2	20006.9	28185	24095
3555	696.41	112	2090.43	1663.082	820.9333	1623.277	1149.787	1922.966	1225.705	1966	1723
3556	696.47	71	3978.28	1049.922	2000.732	2825.28	1169.073	3571.624	2737.696	2409	3904
3557	696.81	77	2811.05	4058.587	3459.669	2321.683	1537.433	2491.883	3009.832	3595	2107
3558	697.36	137	8257.14	3044.843	7829.658	3504.249	3398.025	3122.115	5546.287	10224	6359
3559	697.65	469	786.378	208.4122	11507.39	5778.714	2592.889	1137.257	3033.246	3769	4019
3560	697.71	71	983.505	1951.121	935.8836	1773.768	588.384	2159.687	1030.847	1338	2370
3561	697.85	84	3306.52	2143.306	4657.993	1356.294	2243.15	3736.152	3787.929	3570	3866
3562	698.36	137	1841.28	1521.808	1466.236	865.62	846.1371	1043.625	1985.211	2763	1821
3563	698.47	72	3525.74	2385.166	1859.504	1054.374	1022.224	3419.295	644.52	2646	3752
3564	698.49	1	520.502	319.576	0	478.1005	505.1593	413.952	348.15	153.9	523.2
3565	698.5	1131	3163.61	4671.062	3170.772	3901.851	3761.205	1971.667	2856.756	3873	6203
3566	698.5	1045	2075.64	2073.185	692.181	1973.149	1873.433	3239.473	1113.819	717.6	3305
3567	698.5	256	478.707	381.4409	6203.035	384.6199	441.4661	438.8792	10671.6	7024	417.3
3568	698.5	125	882.633	1573.8	4257.681	1437.444	1809.549	1235.006	960.12	3104	1131
3569	698.65	470	500.574	207.13	4375.178	3476.496	1779.611	759.6645	1180.222	2205	2502
3570	698.75	75	3966.88	4028.533	4268.639	3021.95	2783.766	1865.957	1311.615	1913	1325
3571	698.79	80	6297.55	2912.18	7346.157	3400.861	2683.428	1850.503	2866.062	1459	4004
3572	699.27	95	834.279	2903.542	1141.028	1498.5	2161.032	1037.5	882.6412	1678	2183
3573	699.5	1129	1104.48	2452.112	1491.919	1273.628	1721.132	946.1185	1602.663	2029	1433

3574	699.5	275	160.253	132.2421	1163.994	177.42	153.2608	116.2215	1618.283	2078	141
3575	699.5	256	273.244	189.5455	3052.98	248.0786	193.4568	218.6045	3181.817	3133	203.5
3576	699.7	71	1384.19	2249.831	1034.383	939.195	878.559	1762.631	1026.039	1117	2743
3577	700.37	100	831.465	1088.736	2098.344	1378.6	1031.153	949.6144	1271.34	2221	994.8
3578	700.47	71	1617.2	1622.028	1031.781	880.824	1291.429	2858.827	1983.676	1821	2685
3579	700.54	97	661.373	1986.669	230.1378	1699.763	1699.228	2109.909	1657.495	272.2	1325
3580	700.66	73	1401.68	930.5363	1265.074	1463.426	943.8189	1284.189	1449.465	1780	1296
3581	700.78	79	1542.43	3866.79	5743.414	1451.106	1913.52	2193.64	3697.666	4836	3833
3582	701.4	92	9455.03	10518.63	1037.763	590.94	850.3368	950.4514	1649.567	1361	552.3
3583	701.41	1076	952.136	1022.628	6819.38	882.1867	6542.386	4474.336	822.7796	4298	842.4
3584	701.54	96	447.218	1589.534	500.9292	1422.242	1147.318	1150.871	2059.094	822.2	2873
3585	701.55	1	0	0	0	327.1543	0	366.8158	720.7976	126.8	487.4
3586	701.56	1114	4041.43	1840.602	2616.776	2035.8	3324.782	1979.679	4625.161	1812	1843
3587	701.56	1040	5516.75	3520.61	5274.642	1592.036	4268.246	6221.452	3920.356	4813	4625
3588	701.56	1148	1526.14	710.2629	2174.467	807.88	1156.879	978.2933	1289.05	1288	1205
3589	701.56	1075	5331.65	2608.451	5669.687	2827.261	3330.443	4332.412	3990.94	3511	4014
3590	701.7	72	495.751	1288.056	808.8954	1868.1	613.0927	2504.78	667.5171	1773	1469
3591	702.21	1067	10256.6	4626.403	5099.758	3586.8	4164.318	5545	1246.875	1452	2843
3592	702.41	92	4923.28	5895.54	598.9931	1304.142	870.3822	381.368	541.585	364.6	563.2
3593	702.47	73	1810.4	1046.779	1196.05	912.0182	556.2357	1769.971	2124.845	1210	2303
3594	702.49	137	2689.97	2068.557	814.66	2070.805	2468.689	1855.87	2827.614	3350	2964
3595	702.53	118	2665.11	1798.607	2093.305	2011.9	1906.424	1835.949	1586.318	3348	1745
3596	702.53	160	4016.6	4712.709	5618.722	2755.359	4908.583	4038.244	2750.5	4826	4368
3597	702.56	1041	2755.86	1794.18	3712.771	1552.595	2113.117	2346.105	2246.096	2454	2668
3598	702.56	1076	4959.9	1725.835	3076.23	1684.946	2103.436	2472.007	1293.415	2205	3104
3599	702.65	75	2112.69	2415.559	1394.188	1371.261	1578.157	2684.583	1968.553	5095	3028
3600	702.86	85	71878.4	84670.98	85689.67	36628.61	73846.83	48378.75	68847.68	72526	69790
3601	703.21	1067	7054.02	3472.323	2699.014	2556.025	2734.511	3779.027	863.65	1182	1585
3602	703.37	86	2329.05	2866.367	2545.146	254.33	862.576	1126.549	1014.95	1763	1213
3603	703.5	136	2027.12	1038.174	957.1437	1286.872	1603.867	1293.192	1753.035	1585	1226
3604	703.54	159	2381.08	1337.556	1225.933	1243.176	2173.519	1751.876	1487.576	15682	2158
3605	703.57	468	10650.8	7580.736	6970.035	19713.6	23469.34	14643.76	771.1251	5805	23294
3606	703.87	85	11088	12901.71	14612.76	6908.02	15816.58	8294.292	11818.66	11865	11028
3607	704.21	1067	5605.67	2667.734	2343.731	1701.995	2426.631	3203.665	726.88	965.1	1772
3608	704.51	110	5571.94	4268.842	6316.093	19109.74	7481.261	5251.874	4315.04	4736	7397
3609	704.52	634	984.929	832.182	949.6241	989.9481	745.9375	821.4219	207.1949	1192	1206
3610	704.52	1040	1724.78	2032.084	5222.437	2037.458	1855.271	1517.465	1723.883	2363	4224
3611	704.55	294	2075.93	6699.781	3867.694	5003.142	1163.444	1709.044	3405.84	8798	4788
3612	704.57	436	1440.63	1655.627	730.5822	1916.111	6093.95	2007.644	80.53467	1047	2863
3613	704.57	467	2160	3808.88	2946.317	14304.96	3054.2	6757.625	586.5043	3672	9433
3614	704.58	320	5427.93	4448.528	2779.562	3619.204	1396.679	1408.488	1989.766	3118	4834
3615	704.58	410	1063.55	7630.945	1295.579	871.1858	1307.9	1663.83	96.2742	993.5	2559
3616	704.58	372	4422.85	9951.192	1205.799	5347.215	4696.881	6844.409	362.6865	4390	5575
3617	704.66	71	1028.57	2079.834	1617.638	1763.788	989.6276	1626.504	1347.427	2587	882.5
3618	704.86	85	5855.28	8604.54	8460.751	4604.724	9860.801	4301.027	4323.634	6021	2800
3619	705.21	1068	2649.85	1175.293	912.2176	891.7558	1165.842	1466.312	323.8553	330.7	673.4
3620	705.38	105	1033.69	734.4287	775.1265	1357.2	1057.248	1316.616	1275.346	1072	886.5
3621	705.52	110	4212.4	2427.444	3675.662	34960.6	3951.153	4585.56	2465.31	3026	2554
3622	705.57	470	1189.4	1564.252	1856.509	2208.458	2583.912	2947.811	190.425	2044	2926
3623	705.58	438	386.591	494.9776	231.4471	345.8373	325.5525	539.5573	32.17475	256.2	1186
3624	705.58	355	1218.66	2379.14	962.3449	2027.238	1095.592	1444.287	135.3995	1334	2825
3625	705.58	414	344.406	1269.439	192.424	455.9895	299.1873	1245.255	33.768	280.6	2169
3626	705.58	336	795.024	1593.382	757.2579	8102.346	655.0838	772.4684	212.91	769.7	1873
3627	705.58	376	691.118	4615.003	519.9321	807.696	756.2667	1108.279	95.52576	765.1	1855
3628	705.84	84	2741.73	760.928	2212.69	766.16	512.2367	1856.352	3032.937	3205	1732
3629	706.53	1039	4804.39	4239.512	4119.321	976.3016	4423.632	11027.03	4096.776	5097	2706
3630	706.72	75	2012.3	2100.306	937.7875	1612.79	229.1114	1610.714	1752.66	1347	1433
3631	706.85	83	9068.58	8460.457	8825.136	3079.28	3880.242	4838.883	5261.782	8259	7665
3632	707.54	1039	2173.5	1730.655	2086.627	455.3739	1870.587	1707.987	1918.266	3599	1119
3633	707.62	71	1080.14	962.997	674.497	786.5756	698.1	989.38	776.574	1121	1126
3634	707.85	83	3332.85	2048.388	3342.016	2045.469	2305.891	1659.304	2536.444	2661	2513
3635	707.92	87	5429.96	2925.689	5388.74	1014.323	3451.694	1608.516	2797.22	4376	3802
3636	708.51	1053	4382.81	6912.754	34602.43	1514.173	54285.07	25295.65	3604.703	25636	2022
3637	708.51	1198	861.536	1480.605	8581.925	1207.008	19361.39	5401.04	0	5214	1169
3638	708.51	206	3731.84	2707.216	3542.955	6272.62	1653.6	3445.87	6074.122	8801	6366

3639	708.51	1	5756.78	2325.888	1669.85	522.5687	786.1284	981.9028	466.175	2120	743.2
3640	708.51	1151	395.596	270.48	5714.54	246.4047	3771.004	3075.28	408.4373	8758	255.1
3641	708.51	125	58106.2	23996.9	5606.822	6177.322	7286.174	6186.463	3258.54	1665	6270
3642	708.51	1076	2914.23	2327.163	53057.66	1701.54	49892.13	34791.36	2675.613	31198	1877
3643	708.51	140	22985.2	18322.56	3806.419	3405.761	3675.368	3412.206	1464.31	3476	3172
3644	708.51	1181	1647.8	1256.474	44177.79	1308.15	19396.11	12992.58	1366.848	13951	1353
3645	708.51	19	27422.6	19798.27	2352.999	1712.731	2201.028	1949.123	795.4722	2382	2817
3646	708.51	47	31639.9	42629.07	1754.067	4127.626	2554.772	1797.638	937.8947	1649	1881
3647	708.51	1099	1849.46	1439.263	38782.2	1715.04	28730.44	27945.79	1507.868	26448	1605
3648	708.67	73	1541.55	1713.103	1169.748	1161.888	602.95	1640.725	1791.02	1491	1305
3649	708.81	81	12209.3	8012.424	17145.98	1584.681	3339.665	5674.523	8301.327	11111	7243
3650	709.14	81	2826.71	3493.703	4633.606	1535.94	5388.916	1171.632	1026.514	1456	481.8
3651	709.36	136	4774.72	3150.428	5370.993	2075.865	4341.488	1340.9	3854.584	6685	4512
3652	709.51	1	2751.48	1276.551	973.2206	281.0453	491.892	534.1013	427.5103	1392	389.3
3653	709.51	110	8827.1	12065.38	1339.33	5604.964	1780.756	1251.243	918.495	1300	1930
3654	709.51	1052	2592	2038.433	17936.58	1366.2	25681.32	12233.27	2015.707	12250	1359
3655	709.51	1149	354.321	254.1677	4874.26	268.4416	4126.095	3946.052	371.5294	3824	263.9
3656	709.51	1074	1728.18	1630.077	25623.4	1101.24	24568.66	17863.88	1490.943	16647	1140
3657	709.51	1185	1007.34	805.6273	10188.56	907.7558	10366.27	2531.587	1018.615	7408	814.4
3658	709.51	20	14728.1	10509.52	1616.829	1576.953	1353.865	1252.338	1002.152	1544	1340
3659	709.51	249	906.265	811.4418	1411.482	15129.43	475.2273	1324.91	8900.47	1372	1038
3660	709.51	42	14478	20881.14	1555.562	3161.367	2303.516	1193.446	1096.236	1368	1417
3661	709.51	1100	950.765	718.8209	20248.01	760.4876	13658.04	13664.88	707.8889	12977	835.5
3662	709.51	209	1708.56	1519.481	1856.4	2819.731	683.6797	1512.191	2364.437	4291	1790
3663	709.51	133	29579	8808.603	753.4089	1997.063	2422.245	2022.986	2058.739	2450	2778
3664	709.61	72	1033.5	1101.42	839.6647	1141.817	636.51	1073.354	948.7367	851.5	1064
3665	709.81	81	2277.99	2553.119	3530.961	1797.088	1140.489	1403.676	1498.077	1742	2296
3666	709.87	85	3606.2	1932.509	3304.863	1020.8	2573.567	1855.755	3068.486	3012	3508
3667	710.39	136	5650.09	5858.852	3763.129	4260.377	2857.555	2499.258	5379.906	7134	9330
3668	710.46	85	11431.7	16146.9	13047.97	11760.98	25370.9	8891.172	10330.36	9129	8455
3669	710.51	112	2831.17	5152.557	699.5326	6043.951	1115.125	1585.477	1637.307	983.3	761.5
3670	710.51	1181	413.422	418.251	3092.06	508.3672	3025.784	1830.956	502.7237	2149	414.4
3671	710.51	1074	781.976	596.39	16888.87	603.1	6593.25	4599.815	816.8895	4358	553.1
3672	710.51	1052	870.455	750.3632	4802.292	653.058	6885.543	3644.242	766.541	3336	458.1
3673	710.52	1098	365.976	376.6392	5592.518	354.3767	3485.429	3762.813	414.72	3614	302.4
3674	710.52	48	4354.45	5489.331	345.981	1005.783	435.6655	338.6858	659.3959	300.5	372.6
3675	710.72	72	2774.23	1903.908	1658.258	1567.143	581.9731	1767.156	1555.977	893.4	2123
3676	710.83	82	4042.03	2232.871	5654.618	4607.025	3111.212	3972.56	3809.848	6255	4308
3677	711.07	73	57.2733	1532.363	31.5408	1608.277	1278.128	421.9583	27.51	0	852.4
3678	711.39	110	2500.07	2309.49	772.44	2051.275	1779.194	1528.3	2136.96	2716	3406
3679	711.39	136	3621.64	2744.862	2480.004	2257.355	1784.917	1173.323	1714.554	2839	3641
3680	711.46	85	7295.24	8967.321	5066.698	4512.5	13433.19	3664.117	5592.568	5144	6725
3681	712.37	136	1205.53	883.2539	1678.934	825.1425	497.4389	563.1722	1035.022	1619	1554
3682	712.38	112	671.95	985.5058	1024.837	769.4067	873.08	683.637	1344.478	826.3	846.8
3683	712.46	85	863.394	2479.969	2825.186	2554.514	3846.05	1903.584	1246.239	1006	1513
3684	712.58	73	1872.71	1065.696	1037.837	496.3135	487.1875	627.627	964.6875	1507	502.4
3685	712.59	1111	2737.64	1095.828	2310.819	1644.576	2093.089	2000.946	1614.768	2655	2998
3686	712.67	64	3967.95	1339.563	1689.397	754.3076	899.1209	702.7971	1158.903	2336	1105
3687	712.83	84	2551.46	735.648	1794.09	766.16	800.8	1218.548	1238.148	2722	1219
3688	713.42	103	12079.9	4658.789	11857.44	9253.596	10795.45	11643.71	8140.328	8912	8609
3689	713.88	84	3565.86	4490.987	6864.868	1786.98	2411.388	2656.403	2530.278	3001	4465
3690	714.42	104	4186.15	2520.285	5876.88	3083.22	5390.993	6134.182	3367.057	3483	4285
3691	714.72	78	1873.4	2545.437	3960.939	807.636	770.56	1239.06	1991.905	2419	1519
3692	715.42	101	978.336	1238.689	1638.523	1854.72	1115.92	1307.71	1458.557	1810	1069
3693	715.67	73	469.514	1208.684	791.4966	821.3841	331.5456	690.2883	452.7167	1027	1376
3694	716.55	207	625.371	1061.539	2607.589	2015.659	1361.524	1494.317	1488.628	866.8	1765
3695	716.67	76	793.462	1332.63	3141.538	260.6013	456.0675	1937.504	2382.872	1998	1552
3696	716.88	85	20260.7	27393.66	27540.08	12739.08	28338.78	19167.95	16592.78	21728	26513
3697	717.24	137	1010.4	1204.28	1478.673	214.2	1738.698	282.095	638.81	2544	1216
3698	717.52	133	2067.52	809.445	628.241	1315.947	1059.854	686.9943	2303.572	750.2	670.2
3699	717.88	84	5108.09	7376.161	6135.307	3562.835	2317.245	2677.271	5857.973	2474	5387
3700	718.53	119	10358	8196.995	12904.73	9645.876	10637.47	13984.67	9758.2	8869	12339
3701	718.55	468	1019.52	775.0224	973.09	2282.298	1124.259	964.9672	1075.113	1587	2244
3702	718.58	118	2151.41	1495.83	930.6075	1193.539	477.7994	1155.793	1278.317	1201	924.9
3703	718.63	75	3130.44	1098.504	1899.412	1183.94	850.0569	1176.708	2436.686	3574	2187

3704	718.84	83	26675	18755.42	36380.19	14846.08	21193.02	16386.21	23124.32	24079	20103
3705	719.53	119	6165.08	3706.438	2626.925	5038.164	4705.161	5839.589	4058.264	5743	5832
3706	719.84	84	6536.21	5703.739	9394.709	4346.88	6789.242	3050.71	2266.42	4732	4775
3707	719.93	86	2232.72	1490.58	6443.388	1271.129	2482.853	1653.067	3044.322	1794	993.8
3708	720.44	144	1006.57	1330.007	964.5708	1314.45	1003.864	905.773	658.544	1410	1456
3709	720.49	71	1245.35	724.1429	920.7538	1496.354	1050.192	1660.102	952.6733	1348	2050
3710	720.53	118	2265.85	2110.277	1543.97	1859.1	2383.09	2817.497	1348.678	1695	2737
3711	720.64	76	1957.61	1703.834	1221.087	754.078	512.9778	2130.882	1455.239	1730	1688
3712	720.84	83	4307.88	3181.487	4872.89	3834.964	4670.044	3241.4	4818.777	3786	5036
3713	721.5	1	0	0	0	423.1247	0	801.5445	0	568.9	585.7
3714	721.5	1062	1772.94	1213.984	18618.42	1291.444	23750.14	32009.58	1540.598	12793	1471
3715	721.5	1151	593.683	384.7357	7048.09	443.7651	11006.06	12702.53	572.968	4475	473.1
3716	721.5	119	9064.81	10454.92	838.9173	1811.87	1748.99	1131.509	945.3695	1362	1739
3717	721.5	43	16894.9	13136.63	1356.857	1572.175	1765.469	1044.114	682.587	1354	938.2
3718	721.51	70	23582.1	21712.52	481.1907	1743.017	1120.002	1607.303	538.7431	949.3	1425
3719	721.84	83	982.061	1274.574	2321.285	1279.031	768.0167	1194.16	2757.096	1474	1772
3720	722.01	75	31.24	1225.891	30.13383	1489.964	134.3086	201.2417	2048.76	0	1498
3721	722.47	104	3276.78	4075.005	3320.388	3178.755	3482.072	3079.303	1914.401	3793	3132
3722	722.49	70	13297.7	14938.43	1582.714	2310.809	1487.378	3533.291	1849.945	2994	4123
3723	722.51	123	18233.1	7320.929	2067.313	2357.921	2951.865	2473.503	1656.208	2510	2983
3724	722.51	1185	669.207	696.0536	4218.268	693.1262	2381.159	3732.69	535.0829	2861	781.4
3725	722.51	1157	416.405	414.7623	4900.336	344.729	3956.918	2432.615	323.945	2547	433.8
3726	722.51	1081	1176.15	1444.953	10354.35	1197.493	10325.59	17217.36	869.13	5673	1327
3727	722.55	2	224.128	357.678	259.0118	266.7047	400.2841	519.992	192.7485	344	400.8
3728	722.69	73	2688.36	1121.942	1502.22	698.9483	317.6833	1212.875	3504.468	1725	1502
3729	722.83	82	3253.25	1017.864	9322.218	1797.088	1266.024	2814.32	3254.639	3682	1485
3730	723.47	102	2486.29	1101.625	1222.463	1296.54	1464.154	1468.35	811.1805	1444	1172
3731	723.51	69	1878.5	5229.137	221.1308	422.6349	319.2512	473.6858	438.5923	223.3	325.8
3732	723.51	123	3340.39	2594.107	1700.065	1437.898	1828.706	1365.693	1568.242	1972	2282
3733	723.51	1080	1007.94	661.1738	3165.024	751.2008	2907.66	5269.945	1392.832	2345	1026
3734	723.94	75	52	1298.892	1539.597	1674.4	443.0417	309.0033	53.36833	59.62	1081
3735	724.37	137	2295.8	1987.55	2650.81	1228.205	2066.025	766.2533	1776.096	3420	1324
3736	724.48	72	4156.59	3935.129	2139.013	3741.535	1963.254	4338.319	2524.791	3941	4237
3737	724.52	469	1361.13	630.4258	2845.34	81.216	1155.409	876.803	949.0883	1750	595.9
3738	724.69	76	1520.8	953.9775	1297.415	550.5558	605.4068	1827.743	1989.738	3801	908.7
3739	724.79	81	2790.09	2311.663	3610.484	2054.536	2112.197	2925.489	2463.054	3511	2550
3740	724.92	77	156.2	2275.527	153.1196	1634.28	501.0743	845.215	161.3771	264.5	1559
3741	725.4	137	2352.05	2778.071	3028.567	1580.769	1490.98	1280.859	2691.846	4302	4242
3742	725.55	345	1782.62	2275.945	1233.457	698.3367	1117.042	1518.472	452.0836	2022	4069
3743	725.55	301	2470.79	4586.535	3765.14	1662.998	975.8923	1457.713	1171.122	4104	3972
3744	725.55	419	1690.33	2140.869	888.9253	1527.403	891.8588	1899.133	398.0335	1234	3666
3745	725.55	371	1992.32	2860.235	1436.823	1695.338	1472.746	2294.391	496.8325	1731	3205
3746	725.55	267	1184.59	2822.996	3434.837	341.5523	495.022	491.2443	777.8006	19838	1687
3747	725.55	331	1583.69	2166.841	1545.462	4062.809	986.7914	1098.996	732.875	2359	2118
3748	725.93	73	121.21	1831.872	163.9792	854.669	760.149	1053.183	2038.911	219.6	1803
3749	726.41	136	964.692	1176.252	1564.55	764.5	1075.125	653.5742	1335.853	1971	1876
3750	726.48	72	3846.55	3090.035	1493.809	1354.448	2563.573	1700.492	1699.525	1878	3738
3751	726.53	1043	2826.81	4014.138	1260.63	2471.158	3645.708	3724.184	923.4858	881.9	5724
3752	726.53	125	1556.34	811.35	5701.378	415.2231	510.1943	844.7885	4647.498	6948	1249
3753	726.53	256	827.119	641.6506	17610.03	526.56	513.3944	584.3118	23846.72	19091	861.7
3754	726.53	337	1774.78	942.4117	516.1681	1937.005	2256.927	2960.58	653.7068	456.3	1803
3755	726.82	83	1529.92	1789.975	3853.992	2046.728	1024.28	1388.11	2588.268	1970	1548
3756	726.97	75	82.88	2080.479	60.15075	1879.226	188.705	1171.911	729.4	302.6	1231
3757	727.27	137	1494.6	846.72	2032.881	415.0883	623.152	374.736	634.786	1846	778.7
3758	727.4	102	1675.5	1028.962	899.2973	1072.26	2436.011	1670.917	2169.259	2535	945.9
3759	727.52	130	575.234	589.4769	3099.789	905.1976	736.0861	851.2192	2749.587	1606	583.6
3760	727.53	1044	1776.16	2203.579	702.7971	1563.787	1678.312	2164.145	765.9115	615.9	3099
3761	727.53	256	557.555	259.425	8334.423	301.4296	331.3694	304.3211	10511.78	9788	427.3
3762	727.93	73	1757.11	2385.908	500.4746	2136.135	958.5886	1350.823	179.47	153.9	2899
3763	728.41	103	1014.72	677.1467	865.4529	1053.36	315.2752	441.5644	612.8952	776.6	1273
3764	728.48	71	1003.19	1118.911	882	629.7324	794.9046	949.404	922.74	1162	1528
3765	728.51	97	2706.14	1935.284	4612.702	4199.596	1799.581	3241.542	6772.495	1655	2704
3766	728.52	118	1292.85	1690.413	2607.896	3032.086	1137.932	1771.753	3672.106	2162	1823
3767	728.53	254	1595.16	850.7628	2751.066	864.6584	467.9271	974.3734	24538.11	3262	1015
3768	728.89	74	62.3314	1417.332	2349.167	1880.797	507.9525	1338.799	1028.665	383.7	1686

3769	729.92	73	3274.64	1135.708	369.4603	1891.5	1000.097	1033.211	138.86	355.5	1247
3770	730.24	1067	6733.71	2573.058	2791.39	1784.444	2755.971	3019.68	802.8125	1001	1339
3771	730.52	93	3190.08	1391.315	1470.3	1101.03	3883.16	1324.466	1300.18	1215	761
3772	730.53	469	1115.79	681.5877	1751.987	1494.476	1716.179	1246.52	2519.775	3931	1020
3773	730.53	1150	2132.39	3179.578	1771.44	2220.891	1543.915	4776.345	1398.352	3199	1210
3774	730.54	1042	3289.26	3472.517	2117.409	2715.224	1386.087	3270.312	2356.041	7051	5130
3775	730.54	113	4233.29	3228.607	1433.813	5611.658	4389.989	1948.89	2687.672	2599	2925
3776	730.56	228	881.79	515.3738	1046.506	850.4913	657.8325	817.2459	359.72	827.3	1103
3777	730.69	79	2585.98	2062.711	1288.788	1026.006	256.186	1773.392	1509.158	2878	1813
3778	730.73	62	1392.94	1042.875	831.8813	382.2223	193.3461	557.6063	634.7438	933.4	959.8
3779	730.89	84	3923.33	7810.923	6072.21	5281.712	6478.152	4692.717	2963.866	4014	8696
3780	731.24	1068	4223.12	1451.288	2254.102	1147.809	2041.933	2425.859	465.2236	580.5	937.1
3781	731.88	84	4768.48	3740.793	3075.737	2046.766	2368.95	4643.247	2257.005	3034	1649
3782	732.24	1067	3663.03	1476.413	1790.559	1092	1472.631	2125.152	686.2344	590.5	844.6
3783	732.55	141	28893	25050.36	20795	25302.26	10394.24	24654.39	7552.314	28198	27491
3784	732.55	467	6643	6638.794	8925.896	12943.07	2800.358	8847.519	2304.463	7747	7806
3785	732.55	1	0	1454.021	2261.718	1522.042	0	1400.978	650.727	1398	2410
3786	732.55	1147	2968.75	4667.002	8925.84	1229.444	3004.515	8723.714	5206.365	9464	11034
3787	732.55	278	5463.75	6489.055	3558.482	3207.445	899.1129	5179.553	1312.269	3734	5200
3788	732.55	1114	24876.5	7044.583	17796.05	2631.428	5312.88	7512.219	10600.15	22098	7955
3789	732.55	1041	15242.7	10049.83	13532.38	3529.719	3179.48	9810.466	12463.74	32810	10438
3790	732.55	1192	8302.78	9882.968	8591.34	1753.488	0	0	0	9069	5700
3791	732.55	1078	13509.9	8863.056	16657.77	3332.619	9098.418	7743.75	10893.44	15925	6474
3792	732.55	235	10557.4	8204.87	6695.314	10198.16	1967.25	9698.707	1519.539	7532	6890
3793	732.55	1166	19031.8	7678.309	13289.24	2130.625	6588.086	10301.8	8358.028	17091	7499
3794	732.55	93	12608.5	14321.9	9371.474	1622.797	3989.321	18317.49	3437.368	12602	6009
3795	732.85	83	10871.6	7982.788	13578.5	6137.956	8622.821	5922.347	7834.613	8175	7579
3796	732.89	73	230.955	2777.796	825.39	2519.4	1972.39	2178.874	525.9783	740.7	3513
3797	733.55	122	9626.04	1135.273	7670.635	7637.23	3260.705	16246.32	2905.606	10418	7817
3798	733.55	1	1408.28	1949.919	0	584.1558	0	955.5627	250.4282	778.4	1040
3799	733.55	92	10274.2	8638.73	5061.581	5174.314	1914.516	7643.823	1185.264	5067	5893
3800	733.55	47	5649.64	4877.991	3745.531	4910.52	1034.611	6814.065	823.7046	4405	3816
3801	733.55	1039	4514.62	5262.61	9613.021	1322.815	1824.12	2936.025	4554.422	7785	4217
3802	733.55	467	2680.52	2428.011	3596.481	4122.943	1113.489	3578.639	1615.648	3297	3095
3803	733.55	1111	7031.03	3142.236	8249.173	1303.21	2308.513	3886.297	5180.474	7490	6663
3804	733.55	1136	2600	2232.676	4549.313	608.31	3559.792	1829.835	2917.439	4635	1966
3805	733.55	1077	5848.07	3860.59	7190.26	1565.958	4234.853	3256.111	4730.44	6442	4323
3806	733.85	80	2767.19	1596.187	4395.271	2347.554	2137.456	2680.934	2053.452	2677	3017
3807	734.57	1040	4715.67	3352.337	6091.881	1396.068	1319.017	2879.27	2632.409	9706	4118
3808	734.84	82	6002.49	1813.699	12628	4857.538	2048.56	2329.316	5537.036	6408	4550
3809	734.94	85	976.798	1014.895	2051.511	1274.233	2036.38	2072.608	2761.066	2019	2226
3810	735.4	103	1845.9	1636.884	2900.915	2956.523	1880.096	1955.29	1208.833	2835	1534
3811	735.57	2	523.318	2241.044	527.3643	317.5318	145.8374	291.5048	236.3243	231.9	486
3812	735.57	470	2596.84	1971.778	3452.276	1708.474	1215.565	2198.13	915.8113	2732	2209
3813	735.84	75	837.425	4201.734	1792.98	2859.096	1605.924	2623.437	819	2189	6137
3814	736.42	105	1367.6	2863.386	1189.413	1162.98	1664.915	1378.905	943.5677	1191	1222
3815	736.46	71	1704.28	1781.26	1292.965	981.0958	891.5316	1780.274	1319.939	2017	2436
3816	736.54	1099	350.602	378.63	4173.866	304.9518	2918.41	2601.403	313.0556	2551	447.1
3817	736.54	1052	1243.27	1154.965	9633.517	790.685	4901.76	4330.456	864.5868	4380	1031
3818	736.54	1074	631.092	557.38	3902.094	475.1111	4324.703	2812.361	609.9224	2817	610.4
3819	736.67	77	1571.14	1043.079	1021.957	567.6857	333.3343	1326.622	2092.251	2881	1309
3820	736.81	80	4605.44	1252.44	2363.286	2696.749	1529.773	2060.762	1259.458	2250	3872
3821	736.86	84	5880.66	4446.678	6253.736	1098.867	5185.631	3158.829	4335.879	690.2	5773
3822	737.36	85	2272.03	990.67	2562.84	509.936	769.37	1387.872	2297.79	3262	2216
3823	737.54	1054	1462.6	1291.361	7642.56	1286.547	6847.509	3572.87	1232.069	3183	1290
3824	737.84	72	808.187	4361.406	813.696	2911.57	1282.918	3206.686	1041.463	1355	5948
3825	738.46	71	2702.7	3283.719	1990.345	2360.484	1571.79	3076.558	2755.836	3514	3871
3826	738.67	78	2286.59	1035.272	1343.57	868.15	439.3371	1340.2	3165.201	2291	261.3
3827	738.83	78	1503.13	2156.166	3863.009	4787.218	2813.092	2581.165	2494.58	1461	4492
3828	739.52	258	267.572	295.2784	4001.321	314.7941	256.6202	239.0332	6110.589	4513	438.7
3829	739.52	279	241.46	323.0803	2508.863	213.0392	208.2916	208.495	3946.472	1950	295.9
3830	739.84	73	2856.78	3109.854	3555.557	2668.685	1918.09	5477.071	922.9386	2172	4298
3831	740.45	71	3746.7	2928.711	2263.298	2980.658	1908.474	4102.255	3617.305	2932	5325
3832	740.53	258	549.973	472.466	2007.71	346.2897	479.113	349.353	3298.715	2476	397.3
3833	740.68	61	1130.94	621.0789	490.105	384.0923	84.73617	306.6473	586.5981	386.9	548.1

3834	740.68	78	963.63	1303.158	2544.548	778.5125	722.3575	1531.243	2239.576	2087	783.9
3835	740.8	74	1751.17	4266.164	5065.476	4439.006	2190.5	4520.357	1762.9	2242	5361
3836	741.83	74	1624.11	2754.418	1213.969	1494.225	734.4558	2013.54	1773.11	665.6	3449
3837	742.45	72	3486.93	3694.674	1719.976	1998.49	1359.076	3014.532	1779.245	1667	3255
3838	742.55	118	1309.91	2076.508	1107.534	1560.901	1756.845	1206.873	1903.098	2138	1528
3839	742.8	73	5297.63	5498.958	1919.864	4839.266	1326.542	4381.719	2047.483	3207	8573
3840	743.76	71	2349.09	1677.9	1580.161	3277.973	1799.373	3422.482	1463.438	3508	4849
3841	743.86	83	3815.55	3220.427	2822.348	794.5547	1024.744	2404.319	4586.178	3779	2251
3842	744.43	379	1523.75	1089.065	2003.013	1853.081	2035.976	1113.685	1276.787	2919	1763
3843	744.45	72	1469.5	1190.484	988.0317	1388.825	678.078	2145.808	1183.624	1648	1866
3844	744.55	1043	2624.51	2828.221	2097.225	1960.75	1348.947	1813.65	1211.942	5455	5143
3845	744.8	75	2152.61	4388.381	1826.158	3405.172	6224.903	3236.2	1516.131	1765	4935
3846	744.91	83	1549.4	2017.543	3340.694	2044.597	2305.065	938.6	1281.394	1962	2406
3847	745.34	84	1807.97	1936.886	2821.816	1019.872	0	450.14	765.93	1725	1184
3848	745.56	114	1142.17	2486.266	1514.348	985.5939	1866.306	913.2938	899.4759	1028	1491
3849	745.76	71	3461.78	5174.477	2376.996	5098.464	2768.434	5495.49	2957.563	5246	5995
3850	745.9	86	4239.14	3277.191	6785.995	1531.369	2562.86	1388.46	2326.016	2491	4304
3851	746.56	118	2338.82	3954.733	4825.503	2985.678	3076.886	2571.953	1337.093	7515	3306
3852	746.56	469	2270.77	2144.714	5598.111	1901.397	2744.091	1346.03	1877.009	2312	2700
3853	746.56	158	7229.3	3679.867	7733.792	2419.514	10968.43	3397.696	2318.646	5160	4998
3854	746.57	1041	2832.81	3385.997	3190.949	2637.575	1867.704	1858.78	1823.687	2858	3532
3855	746.57	1	534.84	296.5789	500.6221	103.3013	412.8685	693.4303	633.1545	555.8	505.8
3856	746.79	73	1847.56	2686.71	963.8671	2818.676	1474.266	3011.31	1700.967	2012	3325
3857	746.85	82	2018.23	2631.156	1793.142	1934.959	1406.72	4121.349	3102.717	2445	1829
3858	747.39	137	2704.8	2393.502	1617.151	2384.544	1772.58	990.5333	3799.327	1595	4227
3859	747.55	121	2677.17	1160.65	595.344	1108.407	1359.748	1268.588	3178.98	3233	1753
3860	747.57	157	1697.17	1200.913	1727.679	1162.2	2188.96	1731.327	1070.538	1394	1627
3861	747.62	103	3111	794.429	1612.176	238.14	856.6965	734.76	1506.345	1066	1267
3862	747.75	70	2716.39	7190.867	2459.147	4513.968	3102.098	7320.456	2933.438	4705	6894
3863	747.83	83	2458.57	955.584	2048.76	1665.084	512.372	1455.064	1237.077	1946	1260
3864	748.39	137	1625.25	1747.52	906.1714	787.2455	891.54	644.1626	1378.394	1504	1870
3865	748.53	110	10915.9	2711.937	6483.33	3002.58	8975.736	7768.936	3250.796	6847	3512
3866	748.71	73	3388.83	4657.258	3872.553	4699.11	2501.056	4218.792	2726.778	6782	5028
3867	748.82	76	2480.8	2128.274	5587.507	1288.442	1953.9	1526.676	2473.764	2468	1575
3868	749.54	110	2547.7	2625.279	3261.689	1778.067	4365.479	1616.128	1277.19	2471	2182
3869	749.75	71	3111.89	4874.939	3001.836	4667.181	2814.3	5824.651	2545.218	3936	5608
3870	750.53	2	577.921	408.221	322.8888	139.7032	81.3778	463.3856	394.88	315.1	322.9
3871	750.53	116	3577.5	9631.623	3946.827	3017.067	3542.716	4929.989	2814.331	2380	4043
3872	750.54	1084	1837.69	1651.48	1766.786	297.6831	2052.623	2553.201	2021.026	1488	849.6
3873	750.54	1121	3281.37	1213.341	2130.197	244.9814	2445.072	1631.323	1639.506	1569	876.7
3874	750.54	1044	7784.57	3527.668	3179.108	737.8614	5506.413	3470.667	8598.845	3897	2148
3875	750.54	467	480.13	376.6667	5822.831	327.6206	538.189	514.1861	1410.387	1728	1603
3876	750.71	72	7287.24	7001.389	3644.523	6816.036	3182.825	8298.384	4693.908	9517	8669
3877	750.79	80	9874.07	7528.986	9594.141	4609.677	4903.451	5066.977	4696.965	7569	3134
3878	751.42	137	1900.97	2417.653	1690.08	1359.466	1216.921	714.3618	1397.785	2136	2966
3879	751.75	71	1633.48	3739.541	1480.334	2034.525	1484.186	3831.312	1534.73	1928	3437
3880	751.85	81	3509.01	2312.3	2327.098	1867.811	2656.422	1897.547	2375.605	3363	2438
3881	752.52	106	2063.17	2030.504	1162.52	1215.9	1538.184	1125.76	2579.077	1408	1737
3882	752.71	70	6573.19	6816.753	3794.209	6359.825	3156.021	8228.543	4901.58	8228	8137
3883	752.81	81	6019.34	3808.867	4393.985	3060.647	4060.561	3294.977	5957.205	6995	3798
3884	753.67	71	2911.83	5304.651	2493.895	3427.174	2334.8	4640.664	2343.527	3285	4904
3885	753.79	74	1268.9	1992.054	1895.678	949.3581	1813.377	2634.95	1337.113	1232	2323
3886	754.49	85	6913.24	2480.984	2049.531	3828.673	6784.637	3896.878	3388.96	3648	2219
3887	754.54	138	1667.94	1584.649	912.7205	2392.419	412.75	1461.38	634.6714	559.6	1595
3888	754.7	71	5320.67	7720.679	2874.415	5337.487	3269.682	7846.269	3584.674	4913	1497
3889	754.79	79	3441.49	2718.121	2056.853	2671.778	2403.364	1289.443	1259.869	1712	1356
3890	755.34	276	1055.42	1226.947	5.37504	7.0844	575.883	965.6428	3.374	22.11	27.29
3891	755.52	88	3791.77	1998.593	2667.434	2041	7933.815	2139.008	2235.005	2053	1507
3892	755.67	70	5338.01	6876.682	4479.584	6869.336	4525.651	6977.971	4894.475	7309	9761
3893	755.81	74	816.86	2384.693	941.4404	1569.313	1059.785	1593.328	330.3954	705.3	1866
3894	756.43	72	1496.3	1856.994	967.3825	1413.44	507.039	1262.846	766.062	1606	2585
3895	756.55	91	4688.15	3963.095	4464.786	25700.31	7327.147	4578.991	5519.045	4083	4460
3896	756.56	119	6806.75	2486.814	4536.976	28671.17	9761.198	4853.983	4779.758	9080	3078
3897	756.62	1077	2036.29	1199.25	2479.846	1548.296	2282.155	2645.71	2621.338	2591	2609
3898	756.63	70	2790.54	1032.811	1633.766	6097.834	611.8064	1247.156	1400.877	1409	831.5

3899	756.74	73	2506.18	3018.407	3358.13	3065.67	1045.301	4802.236	1356.871	3036	3657
3900	757.55	99	604.26	1183.179	845.5404	6546.759	1669.666	1980.203	1290.23	533.5	619.6
3901	757.56	117	3226.56	1968.024	1968.688	56724.17	3893.055	2561.012	1355.674	1792	2434
3902	757.62	1075	1178.55	744.12	1286.626	2172.348	1318.873	1518.5	1494	1289	1313
3903	757.66	71	6212.12	7127.17	3895.901	8213.311	3831.658	8883.341	6240.438	7507	10397
3904	758.21	118	1153.86	1667.53	989.2556	1749.122	1677.457	1223.88	970.5584	1011	2396
3905	758.22	139	1873.25	1430.24	1274.623	2181.494	1891.131	1748.657	1748.594	2099	2048
3906	758.42	73	1538.75	1022.954	690.37	1640.726	518.5818	1076.467	901.3373	1430	1731
3907	758.57	1041	5868	8172.964	5862.64	5401.268	11862	11793.88	12982.45	21153	5722
3908	758.62	70	4623.47	4298.652	3029.734	3829.68	2634.053	4633.495	2515.302	4515	4479
3909	758.73	74	4157.34	3910.831	3048.254	2495.425	962.217	2428.823	1528.155	4105	2399
3910	758.86	84	1525.13	1494.523	769.8978	254.968	768.558	685.988	0	773.3	993.8
3911	759.56	122	6089.81	5000.476	6764.088	5546.807	4418.096	6497.919	1953.329	5653	7116
3912	759.57	93	5108.45	8394.097	7200.501	6648.99	4236.798	9539.816	7707.329	7732	7909
3913	759.57	1142	1508.05	2584.448	2402.632	1131.756	2097.253	1839.03	2249.205	1938	1027
3914	759.57	470	4151.86	1937.666	8254.175	4623.576	8227.695	5667.652	1685.366	3269	5299
3915	759.57	326	1986.64	2297.533	2141.958	4908.109	2239.256	2037.15	3822.243	9135	2421
3916	759.57	140	5174.58	5219.652	4482.288	7581.611	3803.713	4974.15	6853.437	5760	6488
3917	759.57	1040	3052.48	3588.779	3437.794	2531.262	5507.702	3697.322	5641.553	4791	4031
3918	759.57	1073	3074.52	3154.93	3662.811	2709.623	4768.506	4124.387	6296.608	3952	2218
3919	759.57	275	2014.47	2188.125	1554.636	2203.177	1705.089	2020.68	2586.48	939.3	1796
3920	759.57	1174	3775.47	2227.558	3422.631	1880.47	3859.898	3289.065	4236.657	6053	3219
3921	759.57	380	3462.72	4126.537	2778.245	3592.383	3964.839	4476.742	4899.47	3650	3512
3922	759.66	70	5440.42	6421.246	3502.591	5320.269	3828.868	7702.073	4600.1	6547	8214
3923	759.73	73	1346.27	6932.691	4241.62	1512.152	606.633	2787.583	4334.614	1228	2889
3924	760.58	1039	17579.9	22673.99	25015.13	8110.768	6884.097	12765.98	7375.296	14969	20117
3925	760.82	82	15771.9	19406.5	23683.22	11353.86	15147.54	8772.115	15652.59	18506	17785
3926	761.58	121	18535.6	11491.46	9396.623	4136.946	6701.184	7683.079	6350.484	7638	12180
3927	761.59	1186	9319.01	7836.866	6697.883	4044.483	3013.189	5857.389	3171.345	6750	7105
3928	761.59	1114	10592.3	6018.943	10360.47	4685.047	3056.072	6234.967	3582.234	6526	8602
3929	761.59	212	9390.94	7304.712	7168.986	4564.568	4652.721	2732.548	3179.411	3031	6878
3930	761.59	1082	7836.32	7002.052	7454.208	4439.106	2692.2	5296.228	2835.801	8513	7369
3931	761.59	1040	15825	11905.94	8451.285	5657.217	3974.091	14880.27	5585.58	7430	8023
3932	761.59	463	4075.14	4403.395	695.7516	3389.007	1841.478	2851.505	1285.496	2761	5159
3933	761.59	1165	11736.4	9435.029	8933.706	4836.949	3340.001	6941.639	3074.504	8345	7965
3934	761.66	73	3184.58	5207.87	2577.622	4033.152	2782.268	4548.544	2719.98	4753	5552
3935	761.73	74	1607.95	3499.035	2130.377	2767.184	901.7825	3736.033	2472.136	2570	3537
3936	761.83	83	1728.09	1738.249	6247.408	2555.771	2307.24	3531.213	3989.071	4196	2509
3937	762.56	118	7929.52	6594.158	7261.613	7137.332	7034.444	10185.21	6787.125	8328	8373
3938	762.59	469	4562.13	4127.883	7555.299	3797.367	2431.173	2870.222	1956.456	2531	5750
3939	762.59	1041	2642.57	2518.83	2705.968	1070.45	988.59	2462.45	984.1114	2193	3035
3940	762.62	71	1004.08	9821.871	5429.63	7925.279	4330.593	9742.193	6310.134	9819	10335
3941	762.76	73	748.658	1373.375	1558.986	1382.939	3322.859	1589.283	1598.72	1553	1479
3942	762.82	83	9831.15	8004.25	10026.11	5614.035	9545.488	6054.788	8281.056	9986	8675
3943	763.37	137	1154.22	1405.422	3570.19	1154.032	2145.031	718.5768	1732.726	2270	1150
3944	763.52	117	1908.32	1263.885	1743.767	1943.013	1137.985	1275.612	1229.244	1054	1377
3945	763.56	118	3865.93	3862.909	5316.757	5177.93	4547.658	6113.397	3822.056	3445	4941
3946	763.58	70	4509.67	4602.208	2549.927	4385.421	1985.342	4827.429	3543.295	4445	5003
3947	763.73	73	1623.7	2523.439	1852.569	2642.544	1651.251	5393.769	1884.276	1911	4426
3948	764.37	136	518.322	781.088	1795.352	243.952	1214.869	193.698	674.576	1670	599
3949	764.56	117	2196.55	1485.408	1163.447	1951.188	1413.952	1623.321	72591.74	1263	2126
3950	764.62	71	5055.7	6703.803	4043.768	5858.884	3873.077	7510.38	5478.816	6524	8614
3951	764.73	76	2564.49	1811.116	5109.213	2591.769	2103.67	3708.824	2016.103	3602	3515
3952	765.58	71	8159.17	7427.392	4775.069	6552.426	3929.106	7862.55	6423.021	7417	9224
3953	765.72	72	1710.74	2841.099	1402.156	2116.368	1284.217	2612.444	1387.918	2161	4042
3954	765.84	84	1240.88	3763.57	2821.816	2553.956	2460.954	1679.125	1005.039	3042	3108
3955	766.5	103	2276.72	1302.446	1941.931	1576.457	1566.214	1915.717	7058.249	1725	2381
3956	766.53	1	0	460.05	863.9792	232.1968	191.7493	282.2158	698.427	1030	244.3
3957	766.53	121	23013.3	6020.829	6112.799	1090.721	7122.085	5485.929	3484.964	5908	3924
3958	766.54	1075	2267.7	2102.511	5297.713	1717.683	1679.813	2070.216	11714.14	17632	1994
3959	766.61	72	4287.49	4167.229	2572.297	3545.875	2062.358	5034.255	3001.688	3459	4920
3960	766.69	73	3559.73	3084.772	2166.225	3414.62	1736.475	5000.64	1757.438	3123	4979
3961	766.83	83	2563.22	3836.745	2919.944	1795.826	1538.556	3152.38	4740.062	5500	2250
3962	767.5	103	1359.36	1117.992	1091.653	802.62	2130.439	2424.642	3461.484	1025	1153
3963	767.54	2	353.113	197.6737	784.0842	134.6575	219.3116	129.9073	576	351.2	128.6

3964	767.54	117	5621.12	5485.444	983.9291	1025.223	6718.846	2807.475	1779.803	4566	2983
3965	767.55	1124	315.19	311.7614	932.9261	213.5021	215.3077	234.7059	2537.345	1432	301.8
3966	767.56	1151	242.582	184.075	664.4115	138.7167	156.2736	204.2039	1377.902	1157	199.1
3967	767.58	71	7895.05	7753.765	4856.096	8095.26	4553.388	7006.452	6494	8315	10322
3968	767.71	72	1189.1	1735.168	1060.669	1189.422	858	1697.122	969.732	2109	1141
3969	768.54	74	8784.12	5516.304	4531.659	5569.452	3173.701	6860.337	6014.204	6989	5639
3970	768.55	291	996.036	1004.446	1352.504	3848.944	456.62	472.2209	664.509	1290	1960
3971	768.55	336	1039.96	1030.354	1601.234	2731.241	538.1157	612.0869	561.0188	2595	2746
3972	768.55	468	2723.28	2455.619	2543.406	3246.941	962.9674	2485.086	1455.722	3393	4211
3973	768.55	373	926.541	943.7628	1042.331	979.0671	364.5513	599.32	387.9092	1276	1480
3974	768.55	305	812.684	877.4675	1144.412	2183.271	457.5047	473.6125	591.2352	1276	1435
3975	768.68	73	3634.41	2870.849	2402.204	4022.669	2113.473	4850.359	3077.62	3681	5610
3976	768.82	83	2019.29	1273.855	3843.61	1795.826	0	2395.22	3543.553	3733	3072
3977	769.57	71	7405.18	7680.801	4362.96	7054.957	3512.986	6199.367	5227.83	8012	10092
3978	769.64	71	1866.8	1920.778	1790.823	2113.636	910.2485	4014.512	4678.072	2660	3206
3979	769.81	83	1970.14	0	1280.16	255.2725	256.2875	1154.158	773.175	1241	1798
3980	770.5	104	3329.77	2049.797	1261.356	1777.417	1475.552	1880.632	13558.18	3030	2053
3981	770.53	70	7387.71	7138.302	4193.058	6203.018	3695.231	7163.611	7060.746	1229	8573
3982	770.67	72	2809.68	3111.088	2426.713	3662.728	1577.419	4508.536	2246.408	2778	4281
3983	770.85	85	54707.6	56738.81	63167.18	24422.97	52969.28	35188.28	46832.39	55888	49208
3984	771.34	84	2997.43	1670.856	2561.58	1020.8	2050.88	916.575	3067.2	2277	1479
3985	771.56	71	2856.54	4589.734	2686.252	4158.864	2177.709	4976.832	3007.858	2127	5626
3986	771.64	71	4291.72	3670.883	2862.372	3338.621	2029.429	6872.185	2670.623	4586	5903
3987	771.85	84	12151.7	9492.84	13455.7	6747.675	12261.47	6497.235	9409.867	8383	11560
3988	772.53	71	9571.47	9071.78	4545.893	6935.055	4682.839	8618.54	8005.706	9275	8957
3989	772.58	469	2097.64	1631.234	3186.287	870.3899	1814.662	1136.613	1617.436	3228	2410
3990	772.58	127	2082.5	2572.676	3547.817	2348.129	1814.506	2421.125	19120.55	2959	4028
3991	772.68	72	3204.17	2809.355	1535.557	1412.01	946.7627	2875.177	1196.723	2667	2908
3992	772.85	84	3924.48	4031.516	1258.952	4088.207	5634.72	3872.259	4095.51	6975	3972
3993	773.37	83	1500.77	1250.096	1282.192	1282.192	1793.302	1484.63	1285.409	1970	1258
3994	773.54	1075	1583.51	1462.118	6667.789	1160.036	11063.47	8779.508	2597.832	8500	1195
3995	773.54	122	8221.33	7583.183	4420.977	3248.105	3083.266	3552.977	5180.448	2813	3376
3996	773.56	75	19137.8	2278.5	19316.33	2100.432	12199.27	2485.958	2867.545	2561	2898
3997	773.64	71	4883.95	4630.192	3372.144	4586.822	2468.1	7630.744	2325.136	5450	6441
3998	773.83	83	2766.6	2450.411	2309.46	1021.67	2016.65	2107.564	2279.83	2210	1994
3999	774.33	83	1007.42	1018.9	2192.628	255.78	0	1126.49	256.4905	979.1	1248
4000	774.53	76	9487.91	8315.763	7985.067	6574.786	9011.92	6114.758	14731.5	9420	8470
4001	774.54	1076	1329.27	1377.2	3354.538	953.2757	4853.027	3224.415	1331.052	5520	2003
4002	774.55	119	3937.79	1823.118	2964.373	1643.486	2077.698	2339.609	4982.344	3705	5135
4003	774.59	226	2088.19	1421.977	1433.264	1264.997	1858.486	1446.13	1320.109	1643	1921
4004	774.6	72	3708.26	4178.992	2048.584	3793.591	816.4897	1948.379	3715.112	1855	4441
4005	774.83	82	8292.73	9311.531	9923.677	3569.697	8301.535	5801.612	7632.204	7968	6027
4006	775.42	135	837.59	644.5238	414.1706	211.3041	447.9155	137.8169	106603.4	388.7	736.6
4007	775.49	72	10472.2	6030.321	4613.584	4553.935	3470.998	8351.392	5900.028	8416	8496
4008	775.54	115	1415.86	1210.71	1471.88	366.7967	868.392	224.287	50228.12	1906	1497
4009	775.63	70	2525.9	4660.76	2764.055	4174.416	1814.993	4281.106	2899.371	3951	6533
4010	775.9	86	3904.75	2496.78	3945.543	1016.624	2026.264	2171.948	2811.725	2053	1765
4011	776.23	154	119.605	347.2074	2865.158	221.229	141.232	143.4108	2513.166	1348	219.4
4012	776.45	72	1770.54	5178.566	943.48	927.0003	919.1	2285.84	1447.381	1419	1047
4013	776.55	2	391.666	629.6855	360.3024	215.3889	142.0184	430.3316	248.4186	373.6	259.7
4014	776.56	112	4019.8	4490.656	3299.327	3058.398	2641.911	3946.598	19229.47	2573	3389
4015	776.56	1045	1515.84	1383.06	7926.771	921.1714	864.744	1088.718	1900.541	2392	1985
4016	776.56	127	10250.9	2036.115	3818.907	1954.678	2015.115	3521.849	58421.9	2900	2798
4017	776.57	143	3147.81	3819.553	2670.719	3317.24	3237.344	3537.272	1332.616	5625	8019
4018	776.59	70	4513.62	5695.869	2890.347	5162.887	2236.924	6632.233	3996.872	4582	8202
4019	776.79	81	10643.4	5720.904	12739.36	4355.82	2306.572	5695.147	7545.89	8058	6798
4020	776.83	83	2536.8	2499.75	4984.138	4604.28	4186.287	5685.53	7800.288	8520	4003
4021	777.12	81	1759.79	513.9148	1024.516	768.355	3336.869	0	747.992	721.1	1292
4022	777.48	71	7655.72	8524.616	4679.193	6067.205	3974.1	7782.096	7287.676	7950	9013
4023	777.56	2	410.943	454.4296	208.0131	162.3563	105.0033	209.3833	183.8865	161.4	134.9
4024	777.56	1098	929.652	683.2921	2168.93	575.7954	1991.816	1196.03	13716.22	1920	880.8
4025	777.56	1076	746.733	578.011	2695.211	648.1438	1785.203	2301.771	1432.108	2139	902.4
4026	777.57	144	2447.69	3010.314	1409.8	1481.606	2099.581	1817.691	2722.88	1199	1977
4027	777.57	127	4014.46	2423.889	1688.221	1849.735	1596.994	4573.707	20890	1990	1509
4028	777.63	71	2427.98	2335.896	1537.29	2715.337	1471.201	3611.42	1927.105	2392	3844

4029	777.85	84	3002.52	2985.156	4616.829	1789.689	3397.25	2149.383	3243.259	3078	2731
4030	778.45	70	4761.19	6314.44	2933.235	6552.788	3014.394	4016.688	4151.923	5549	6092
4031	778.53	94	694.377	487.8269	745.8669	8905.739	2382.496	1373.363	14203.57	1222	336.9
4032	778.53	117	2948.22	1560.756	1695.321	5056.351	2169.544	674.2933	18977.76	3444	1775
4033	778.59	72	6983.02	6285.501	4115.926	5399.387	2453.1	8599.94	4624.704	4879	7141
4034	778.82	83	3465.99	4458.169	6695.288	3582.798	4165.205	3579.6	5559.735	4424	4698
4035	779.48	71	6529.88	5617.935	3638.631	4814.79	3002.7	6665.774	5272.52	5920	7153
4036	779.56	72	5416.3	3143.207	1135.493	1505.527	1490.306	5996.401	1094.625	1257	4067
4037	779.84	83	1498.42	1208.996	2318	1024.744	1429.524	2361.37	1768.602	1771	1481
4038	780.44	70	11282.5	9333.213	6027.765	8475.196	5817.001	9472.772	7584.15	10162	12637
4039	780.55	94	4562.91	9773.34	5503.502	10950.51	14512.94	6568.226	7732.091	4812	6983
4040	780.55	1	499.573	402.5667	0	1001.149	691.8617	432.6272	992.8605	254.4	337
4041	780.59	73	8179.57	5501.327	4388.797	4554.12	2480.683	7275.893	3365.908	6177	7798
4042	780.67	76	1922.94	1865.019	4011.933	305.0529	1729.28	2042.18	2376.057	1695	2850
4043	780.83	81	2742.76	1965.787	2306.826	2823.094	768.558	1664.97	2259.81	2010	2554
4044	781.48	71	4851.58	4128.236	2258.135	4434.101	1700.4	4602.867	3366.9	3714	3872
4045	781.55	99	2576.61	2736.249	3331.952	5013.694	2660.213	2993.475	3437.215	2523	3529
4046	781.55	70	8696.81	6443.687	4768.758	12609.59	4577.939	9461.261	5909.516	7957	9243
4047	781.63	465	652.795	473.1564	613.8333	1147.548	940.9961	709.2565	792.9482	1199	500.3
4048	781.81	83	1763.43	769.37	2741.666	1534.39	512.2367	1406	1252.074	2791	3292
4049	781.87	84	2990.04	3959.505	6274.19	3582.403	3475.17	2322.394	3020.82	4936	3500
4050	782.44	70	12338.8	12810.5	6186.24	9902.307	6514.729	11085.94	8674.25	10730	13546
4051	782.56	1	0	1189.066	0	4633.506	3298.574	389.4088	866.3665	376.4	732
4052	782.56	94	9416.28	13434.41	7682.606	23398.2	9183.317	3227.092	7549.143	7946	19232
4053	782.57	1041	4146.5	3722.737	3176.296	7064.155	3516.423	3629.765	7846.297	7637	2944
4054	782.57	123	4143.35	4308.725	3762.072	14706.59	6317.442	3841.32	4774.291	3286	3366
4055	782.66	74	2593.84	5729.59	1580.076	1647.409	3612.374	3213.466	2599.925	4049	2366
4056	782.79	81	3380.98	1792.603	1471.459	1803.14	938.63	1668.78	2551.646	2519	2626
4057	783.54	71	12185.7	8429.81	1165.553	1008.765	1166.212	1840.206	1850.142	1475	1997
4058	783.57	1040	1425.82	1336.041	1377.5	1334.978	1320	1706.933	2512.094	1284	1178
4059	783.57	466	1746.28	1343.789	2028.359	13674.56	1873.858	879.9491	2451.168	1628	1571
4060	783.57	253	4280.65	2290.752	2774.831	13159.91	2370.282	1496.336	3097.035	1343	2381
4061	783.57	100	6426.99	3683.671	4171.213	19413.6	15324.77	2599.827	3915.184	2928	5108
4062	784.44	71	10888.3	9226.838	5024.55	7696.836	6163.989	9206.676	8718.806	9347	11442
4063	784.51	72	3919.97	4441.567	2749.335	3305.258	2978.632	2879.072	3135.814	3769	6165
4064	784.58	1040	3472.38	4023.149	2758.602	2962.388	3175.738	2025.085	3382.5	3508	2863
4065	784.66	75	1240.62	1611.4	3338.239	3862.429	2795.943	3038.885	2012.606	3061	1865
4066	784.87	85	19326.7	20919.44	21228.57	9447.632	19125.17	11234.51	15417.12	15708	13823
4067	785.55	70	5606.42	4382.252	4372.787	5191.237	2383.58	8109.14	5106.544	949.8	6051
4068	785.58	125	3083.9	2806.44	2471.788	3650.933	2073.334	4454.715	1253.537	2039	2011
4069	785.58	474	1520.47	1352.508	1525.056	2798.07	2194.367	1301.376	1600.774	2187	2610
4070	785.62	72	1995.07	2659.67	934.0013	2295.042	1516.133	1874.04	1845.741	1660	1470
4071	785.86	85	5495.71	4843.44	6796.169	4339.48	5506.978	3276.188	2992.729	3843	3017
4072	786.43	71	5371.68	6525.74	3153.004	5659.325	3124.2	5058.351	3523.288	5910	6419
4073	786.51	72	7273.36	5770.526	4309.72	6589.95	3322.983	1804.855	2841.7	2029	9677
4074	786.6	1041	15526.5	18974.29	12290.2	2603.419	6396.459	6172.932	6402.406	15192	12795
4075	786.65	74	2269.14	1770.04	3847.141	3075.625	3054.073	3464.9	2841.856	3478	2018
4076	786.82	83	20969.4	17727.64	29380.62	11766.07	18588.44	14944.01	17416.87	21197	15765
4077	786.88	84	1284.22	2753.029	1027.226	1276.773	1025.735	1431.171	1252.272	1523	1226
4078	787.32	85	1268.18	477.225	1285.494	509.53	0	894.855	1259.67	1762	256.8
4079	787.42	108	1074.97	735.2169	1061.422	1277.64	1077.703	764.295	533.3451	627.7	374
4080	787.46	72	861.9	1105.746	2546.895	3289.321	1126.737	4114.533	2368.561	3539	1763
4081	787.54	73	3348.23	2990.317	2455.485	2843.722	1283.223	5503.14	3469.069	3317	5735
4082	787.6	466	5801.52	6358.547	9582.442	5986.196	4235.995	2862.628	745.6696	3139	7411
4083	787.6	1163	6299.81	6047.58	5224.479	1552.329	2062.426	2662.523	1560.16	3417	4519
4084	787.6	1	0	1517.988	0	532.5285	0	572.1092	606.7633	227	1326
4085	787.6	1147	5124.53	2419.16	5150.204	1954.86	1804.018	3323.914	1566.468	10657	2597
4086	787.6	1041	6437.62	9001.378	6320.6	3382.402	3300.107	4898.857	3785.117	4014	6358
4087	787.6	1094	7172.53	6210.88	6872.931	3339.63	2533.987	2653.783	2584.056	4864	5167
4088	787.6	1118	2410.23	2058.435	2371.265	1019.846	778.22	2032.87	831.2477	1503	5725
4089	787.6	120	9922.82	10349.59	10786.39	4333.733	5595.32	4856.773	4664.134	5727	11363
4090	787.82	84	5346.03	5528.023	8765.339	2303.513	3741.868	3057.377	5588.593	4500	4873
4091	787.93	88	1991.67	1278.636	2717.701	0	255.8138	930.1122	1278.467	2259	1023
4092	788.35	112	1251.95	125	1323.52	256.1183	449.3497	278.8283	475.6483	495.8	781.9
4093	788.43	71	3066.93	3573.235	1573.1	2788.014	1553.649	3897.667	1452.38	3030	3719

4094	788.5	71	7242.74	7940.028	5893.785	5999.671	3528.129	7711.316	5373.657	7521	9891
4095	788.6	117	7518.32	6207.33	4728.208	2801.1	4789.773	2848.262	771.0178	5096	5670
4096	788.61	1145	3789.84	5834.639	2068.883	1114.301	1351.695	1917.76	844.8227	2698	4046
4097	788.61	471	2788.27	2614.186	4771.454	1095.668	1550.499	1334.868	1585.734	3876	3896
4098	788.61	1171	3956.74	3902.556	3106.547	1203.323	1314.898	2168.495	877.3531	2834	3790
4099	788.61	1040	3892.77	4663.222	4221.202	1154.397	1472.266	2054.276	1176.843	2595	3329
4100	788.82	84	5080.08	5462.005	2662.315	3579.636	2819.844	2814.625	5060.794	4684	3244
4101	789.47	76	8284.38	5160.937	7100.931	3392.753	5920.729	6597.22	8843.089	10230	5452
4102	789.52	1057	632.335	702.2143	2001.443	549.8889	2735.957	1415.444	507.3378	1971	611
4103	789.54	71	3537.19	1743.183	1488.551	1038.78	1019.957	2272.687	1685.669	2201	2280
4104	789.62	114	2362.07	3719.86	4739.719	1247.604	1953.339	986.821	2433.62	2941	4565
4105	790.35	137	1544.32	700.4667	1502.734	440.3217	323.85	536.0571	869.6133	1797	1283
4106	790.43	72	1125.23	1209.911	6176.814	1196.726	1190.589	2228.274	1176.988	2238	2164
4107	790.5	73	7072.85	6618.338	1227.064	5237.89	2670.077	5467.932	4901.345	8096	8295
4108	790.54	1046	3477.6	6319.688	1125.881	2735.175	3953.739	4349.45	711.2733	2154	10655
4109	790.68	76	1834.38	2426.759	2067.439	989.7967	600.7377	970.7539	1296.611	2119	1794
4110	790.69	62	1900.5	685.0457	488.7	633.6707	216.6467	421.9371	607.1771	666.4	983.7
4111	790.81	81	4009.5	2834.801	4288.161	3078.467	2048.947	1678.287	2726.633	3978	2776
4112	791.46	72	8883.66	5685.003	4233.816	5390.583	2842.983	7312.259	14567.19	7826	8249
4113	791.54	1045	2156.74	2591.713	1190.805	1981.7	2316.569	2438.383	899.9774	1293	3258
4114	791.54	1117	716.522	829.1844	442.294	678.9512	485.6009	663.1772	286.1222	328.7	814.6
4115	791.57	138	624.74	672.448	560.176	706.299	817.215	321.818	1677.505	262.2	726.2
4116	791.6	71	1355.9	1125.327	1331.671	1161.882	537.5949	2801.175	1250.653	1728	1732
4117	792.42	73	2005.16	2035.404	2567.389	1949.949	1087.521	2767.189	1343.658	2287	2510
4118	792.5	71	2817.53	3355.079	2259.535	2017.164	1883.794	6320.201	7686.852	2117	5733
4119	792.54	1	433.941	561.1	786.6346	272.3327	130.7367	504.7439	1057.019	296.8	468.8
4120	792.55	1044	1345.04	1548.553	3257.331	1070.052	1602.643	1285.92	4343.75	3361	4010
4121	792.56	109	4617.07	6947.16	1888.069	2369.339	4925.736	5675.793	6774.227	3986	5851
4122	792.6	121	6528.03	6869.747	7578.403	1343.094	5428.8	4205.178	5060.897	2340	6336
4123	792.62	140	3692.47	5900.222	2673.904	1536.039	1901.414	3063.443	6457.102	4317	3908
4124	792.78	81	3340.57	1745.237	5978.81	2052.012	1795.826	2806.084	2975.927	2954	4040
4125	793.46	73	8063.97	7356.646	4653.307	6158.042	2756.242	9209.786	6428.515	6618	8515
4126	793.56	1046	1021.78	1167.529	2801.91	1093.201	1444.828	1158.123	3557.663	2701	2177
4127	793.57	110	1368.86	3078.064	6225.683	1368.632	2693.049	2214.655	11861.22	2459	2388
4128	793.6	74	1327.99	913.6951	795.6471	1105.01	443.7892	1652.49	1014.991	1139	1536
4129	793.63	209	1269.13	487.8187	1940.34	950.9415	1358.851	584.2504	931.2463	1135	680.1
4130	793.63	139	1633.05	1932.501	2774.642	551.9382	922.0294	1949.885	3257.882	2441	2036
4131	793.83	84	3285.6	1238.72	1687.304	1020.413	1023.212	1620.504	1492.897	2233	1955
4132	794.47	71	2130.82	1948.634	3702.371	3924.469	2368.077	3960.539	2975.386	2695	6394
4133	794.56	112	3651.59	3140.301	5104.373	2763.149	2716.817	2478.986	1493.91	2306	3337
4134	794.57	72	2642.28	2561.547	2224.807	1932.158	1628.19	4303.208	1203.624	2456	5493
4135	794.58	1047	2096.64	2246.3	1577.423	1096.886	737.5343	1693.416	1873.941	1883	2696
4136	794.58	467	865.978	728.539	2022.765	862.3913	705.166	733.1598	363.682	1201	1470
4137	794.81	82	1993.81	2208.307	5763.199	1791.72	256.1183	1877.2	2517.682	2750	1514
4138	795.46	71	6872.4	5642.386	4256.256	6603.264	2557.1	6415.17	4581.738	5977	9172
4139	795.53	73	2215.86	1367.781	1101.242	1686.165	803.985	1636.427	1770.158	2005	2620
4140	795.82	83	3314.42	1248.585	1889.726	1535.675	256.2875	1387.598	2007.034	2491	1478
4141	796.42	72	12015.1	8454.149	6746.796	7733.778	4518.8	11873.66	7039.914	8937	13189
4142	796.53	106	3288.33	2207.261	2784.99	2870.619	2151.172	2954.219	2580.976	2888	3189
4143	796.56	72	4611.92	1566.912	1099.808	1616.11	1022.375	2048.581	1682.747	1952	2253
4144	796.63	77	3067.23	1679.12	2417.427	1134.835	338.5375	2364.4	2015.546	2755	3786
4145	797.45	72	3755.74	3038.813	2116.449	3292.572	1831.59	3839.446	2342.214	4211	3872
4146	797.52	1041	400.664	514.299	424.2563	681.3806	821.7517	506.4729	2032.947	1381	2172
4147	797.52	107	2334.34	1056.116	2097.524	1490.229	907.6308	1216.348	1719.802	1577	885.7
4148	797.53	70	2752.58	2578.5	2718.533	2619.033	1298.047	3979.634	2861.291	3818	4994
4149	797.78	79	1113.95	1239.904	1803.7	1282.192	768.558	231.312	234.0252	1241	1554
4150	798.42	71	13280.2	6959.614	8700.132	10084.8	5454.786	12150.99	9656.763	11880	14747
4151	798.5	72	1655.14	1450.376	1347.382	1881.663	730.8679	3175.255	3270.994	2341	1260
4152	798.54	100	5016.11	6678.145	3327.643	4024.648	653.544	3498.512	1560.684	2347	5784
4153	798.54	245	2385.97	830.83	1692.966	624.8007	544.7398	596.0864	711.8097	534.8	985.4
4154	798.88	84	4025.9	5747.638	5390.002	2815.824	5361.792	2604.635	2040.654	3749	4556
4155	799.45	72	2306.03	1743.697	1684.293	2925.555	917.4203	2100.546	1641.154	2555	2851
4156	799.52	71	2530.92	3206.225	2286.365	2830.525	1050.16	3838.097	1716.402	3075	3800
4157	799.87	85	3287.75	2985.892	3160.085	2552.192	1678.899	2513.652	2809.732	2475	2029
4158	800.41	71	11315.1	10202.1	6437.848	8406.618	4277	8857.507	8769.685	8585	12927

4159	800.49	73	3523.49	1378.31	930.2014	1269.221	850.2818	3016.288	8982.015	1492	2625
4160	800.6	118	1344.86	765.1345	2222.457	2866.569	2310.681	2281.825	4250.947	1576	3568
4161	800.64	1052	1348.13	747.2912	522.45	1424.201	1345.992	1580.364	1626.902	1188	1459
4162	800.71	78	3349.45	1811.091	3886.923	1293.707	329.7933	1296.845	1010.755	3565	838.2
4163	800.84	83	7348.6	6994.25	12720.8	3318.352	5765.096	3348.086	5561.351	5063	7465
4164	801.52	72	2317.34	2208.988	1984.194	2629.916	1048.428	2863.273	2110.5	1889	3023
4165	801.6	118	1722.61	1101.047	489.608	1190.547	1090.334	1219.271	1838.878	780.7	2543
4166	801.83	84	3044.06	513.9829	3354.581	1020.8	2465.357	1915.213	2012.089	2237	2753
4167	802.41	71	7258.04	6601.488	4706.613	6122.748	3123.9	6267.032	5101.061	5787	8136
4168	802.48	71	3421.84	1895.437	2551.767	2332.355	1210.955	4678.764	2878.333	2285	4047
4169	802.61	115	996.117	948.588	966.7156	2039.315	1066.684	609.84	1061.126	238.1	396.1
4170	802.8	82	6562.53	4487.733	10024.84	2809.229	5933.35	3990.763	2106.63	4765	4816
4171	803.52	71	1494.63	1461.148	1044.396	2305.771	585.045	1730.198	1127.412	1435	1698
4172	803.54	98	2597.79	4806.239	198.606	3940.7	693.312	4359.569	1471.771	483	3926
4173	803.82	81	2789.12	1251.085	3009.243	1278.89	1393.363	1830.455	2257.281	1738	2544
4174	804.41	72	3717.75	3098.889	2232.707	2812.629	1274.621	3416.642	2128.091	3493	5415
4175	804.48	72	4765.72	3609.048	3609.635	2831.819	1485.109	3941.854	3555.762	4359	4800
4176	804.54	97	1754.44	3407.868	563.6677	9765.919	6055.449	3737.733	2335.37	684.8	3106
4177	804.74	79	1787.68	774.822	1128.161	774.7	41.778	2120.337	1584.872	1797	1741
4178	804.84	85	4097.94	3678.776	5128.487	2043.512	4243.222	2607.632	4601.464	6266	5819
4179	805.34	85	2039.62	512.14	3334.985	764.73	513.4014	231.1029	1540.99	2256	735
4180	805.44	71	1857.7	1145.896	905.1743	1813.244	802.4968	1727.12	1103.682	1334	1937
4181	805.55	99	1360.92	1002.687	396.1511	4913.245	674.472	767.6229	1221.288	1228	971.1
4182	805.84	84	2041.91	1247.321	1965.055	766.5788	512.575	744.3488	1738.589	2240	1770
4183	806.4	72	1673.13	1605.12	1068.228	1334.727	845.649	1811.257	1364.434	1218	2739
4184	806.47	71	3791.11	2728.697	2714.928	3220.094	1200.276	1753.644	1320.57	3377	4705
4185	806.57	471	2002.91	1388.725	3671.604	1426.93	2528.609	1086.3	1893.101	2366	1643
4186	806.58	118	10143.5	5721.322	8911.606	8961.832	5233.817	10759.84	8507.674	6146	9224
4187	806.83	83	2320.54	2564.916	3074.318	2297.96	1536.463	931.52	2010.825	1267	3286
4188	807.44	72	3591.48	3250.459	2375.728	2977.163	1340.886	3876.717	3954.224	3183	4485
4189	807.58	118	4999.14	3735.895	2139.88	5020.062	4442.751	2597.954	2928.24	4231	3212
4190	807.82	83	3760.37	2955.04	2827.568	1282.192	1796.46	1388.46	1796.454	2732	2997
4191	808.4	72	1372.8	520.344	753.912	1594.71	665.6816	1257.311	773.2729	1403	1554
4192	808.47	72	2997.41	2062.973	1728.72	2968.26	894.5099	3212.24	1330.788	2485	1605
4193	808.58	112	8765.91	7072.252	8541.953	2635.51	2983.295	5643.512	3366.614	5919	6795
4194	808.58	93	12348	20079.03	6754.942	7428.187	3397.637	5659.761	7655.392	9584	12982
4195	808.58	1041	3676.84	3413.666	4464.249	1298.8	1229.399	2488.786	2394.211	8173	3482
4196	808.58	1077	3589.23	3111.6	3640.32	1411.76	1147.208	2256.308	1730.309	3978	3089
4197	808.58	1115	2506.65	1692.181	2141.254	780.6876	609.2667	1238.95	608.685	5258	1948
4198	808.58	1169	3675.39	2412.878	2521.68	789.7931	1134.039	1988.68	1615.627	3558	2061
4199	808.58	466	3178.16	3108.038	4273.931	2795.618	1775.556	2717.331	1113.06	2867	3349
4200	808.81	81	5205.18	3312.831	1536.143	2565.746	1537.681	1824.686	2513.116	5066	3330
4201	809.43	71	4927.64	3659.379	2862.28	3171.495	1694.852	5281.147	2093.202	4436	6039
4202	809.55	78	3025.07	1192.286	818.7503	2213.067	582.9056	3752.056	2370.655	1025	2061
4203	809.58	94	6259.6	8039.952	6519.101	3320.201	2026.15	5503.728	2246.533	4474	8489
4204	809.58	408	483.551	445.3182	450.4631	260.6743	184.853	344.1122	275.5971	329.3	461.1
4205	809.59	297	1177.09	1377.397	1096.546	1260.534	326.9154	800.8	468.124	790.7	998.7
4206	809.59	336	1880.35	1759.198	1773.169	1586.23	671.3065	1081.175	811.9007	1356	1839
4207	809.59	378	1513.19	1765.69	1438.369	751.3575	474.504	1190.581	508.5143	1002	1388
4208	809.59	314	1147.8	1211.435	1107.726	965.7522	282.285	552.42	425.5612	575.3	989.1
4209	809.59	273	1857.74	1870.098	1651.7	809.477	557.4397	929.995	650.863	814.6	1464
4210	809.82	79	1502.05	773.175	1465.09	770.125	770.125	462.075	468.0504	251.8	776.2
4211	810.4	72	3514.87	2987.138	2793.702	2672.626	1112.701	4110.068	2402.318	3570	3927
4212	810.47	72	1030.98	1396.239	700.248	1006.574	785.6158	1109.118	1916.229	2006	2441
4213	810.52	102	3388.45	1319.966	2566.017	1620.36	1663.823	1475.017	1489.468	1570	7177
4214	810.58	122	3890.77	4821.954	1265.6	4502.786	1694.757	3728.835	3929.16	5600	6013
4215	811.43	73	3908.04	3151.792	2427.276	2362.608	1133.171	4169.28	2497.892	2034	4575
4216	811.6	109	1709.43	1982.333	2027.649	990.884	605.9284	1010.059	1132.897	1011	2011
4217	811.85	84	1238.93	2470.789	2564.121	1797.088	2048.27	2872.188	3050.693	3567	3686
4218	812.39	77	16422.3	9159.095	18151.16	4772.789	10436.4	6382.92	5465.861	13834	6956
4219	812.53	62	1934.63	1428.466	1707.685	1077.643	959.035	1379.377	3338.616	2807	1343
4220	812.61	135	2502.56	1243.983	2309.398	759.2	536.928	822.4734	2165.94	1013	1983
4221	812.61	100	2369.5	1338.705	1022.67	1307.462	891.419	793.0887	1076.637	1214	2317
4222	812.74	79	1753.63	1031.465	2215.766	257.4717	515.5333	0	1760.898	2451	1127
4223	812.83	85	7856.52	3452.041	5652.245	2298.938	2567.389	2858.937	4027.76	4518	5402

4224	813.32	85	2018.23	256.2875	2561.58	1020.315	767.34	1196.52	1281.192	1248	993.8
4225	813.44	78	11387.5	8129.825	10434.26	4656.051	7590.002	3613.259	10681.6	9760	6413
4226	813.52	68	621.6	393	805.4364	1132.693	544.3196	323.8528	2393.652	647.4	375.2
4227	813.56	144	2216.35	2927.273	2075.272	687.6789	459.792	2430.292	1242.657	2412	2700
4228	813.56	124	2792.22	3002.311	2071.36	647.6511	449.3195	3505.427	1352.414	1410	2521
4229	813.89	86	2960.06	2751.656	2307.833	1780.325	2050.02	2382.338	1755.942	3560	2826
4230	814.39	72	10594.9	5469.855	5103.748	5119.996	2384.723	7609.44	4352.906	5465	8484
4231	814.45	78	4317.42	5755.186	2798.533	5810.1	3260.16	6699	3361.893	4307	8058
4232	814.84	82	3057.45	1200.8	1467.769	2048.76	2874.648	1130.291	2553.699	2225	2257
4233	815.47	71	959.743	1193.865	821.0011	1089.099	963.1645	1244.046	1211.325	1087	1851
4234	815.84	83	1762.16	2276.3	2307.06	1539.64	2234.714	2154.295	1537.024	2250	2511
4235	816.39	72	7106.93	4091.816	2942.109	6493.252	1879.559	6173.016	4049.749	5157	6817
4236	816.46	75	7318.59	4151.383	4248.968	5922.381	2021.568	6080.568	4432.408	2097	2141
4237	816.82	83	4348.43	1753.168	5070.453	1278.128	256.186	2095.5	1238.344	2410	2638
4238	818.38	72	4355.08	2887.445	2813.926	3057.019	1805.826	4589.662	2311.778	3508	5242
4239	818.46	74	2596.82	3182.302	1587.75	3296.315	2263.085	4102.88	3041.692	1281	4984
4240	818.68	80	1292.35	773.175	1501.754	1026.413	0	770.125	1514.873	1779	776.2
4241	818.78	82	7637.41	2832.669	8860.005	3590.703	4387.921	4775.333	2244.406	7998	5602
4242	819.83	84	4321.69	3256.11	4616.928	1790.171	1315.686	2788.389	3537.072	3032	1732
4243	820.38	73	875.044	2059.057	1374.613	859.7453	1197.924	3425.416	2026.374	2068	2633
4244	820.51	98	858.941	812.478	652.366	2220.12	1129.695	1983.836	1113.053	1024	357.3
4245	820.59	125	3015.13	1362.906	3721.967	1784.473	1754.445	2295.818	1475.46	1870	1851
4246	820.6	142	1743.04	4381.162	2356.028	1267.096	1954.202	1828.499	1774.674	3659	1947
4247	820.81	82	5047.87	1524.549	6559.143	2043.34	1874.64	2828.29	5207.93	5528	3017
4248	821.6	127	2416.21	896.274	1360.304	1292.222	1012.232	1078.904	1155.974	889.3	1163
4249	821.81	81	1528	982.2608	1626.968	1024.38	1490.612	1171.94	1538.19	1761	1216
4250	822.38	73	933.4	567.2841	560.9711	659.1634	414.8925	840.051	837.09	1244	1966
4251	822.52	96	3050.94	3627.225	2224.144	2062.647	410.989	1537.115	1070.85	1617	2701
4252	822.57	119	1112.5	338.7429	912.08	632.043	1286.323	1612.779	1136.186	1358	602.9
4253	822.8	82	2745.75	994.3258	3627.048	2058.895	1359.491	1499.581	3023.223	3041	1769
4254	823.38	78	8515.14	1342.015	2277.493	1763.938	8771.072	2096.116	5199.514	4694	3153
4255	823.79	84	1790.1	514.855	1205.42	765.8175	256.2875	1201.24	1001.564	1500	773.2
4256	824.38	78	4505.86	462.536	993.1508	1941.131	5290.92	1881.158	2031.044	3287	1126
4257	824.55	98	2516.21	2521.531	3970.155	1095.72	1163.644	1599.585	1250.735	943.6	1716
4258	824.74	81	1759.75	2361.744	2429.765	2722.947	1483.786	2351.544	2839.784	3949	2679
4259	825.4	74	3048.27	788.1389	1438.458	1285.67	1631.916	1703.009	1474.907	895	1968
4260	825.55	99	2136.93	1178.284	2402.55	430.3331	859.923	971.3998	664.6923	1269	1671
4261	826.73	80	3103.68	982.2608	2468.226	1026.006	0	1237.08	2009.7	2766	1292
4262	827.41	75	1436.95	673.4043	870.8783	739.7711	550.605	1122.371	1043.139	1143	1833
4263	827.82	82	3532.59	1202.064	1544.52	1534.76	0	1132.84	1281.192	1713	997
4264	828.42	78	9378.15	5857.947	13080.82	1994.493	7467.273	6207.665	7997.389	10534	5061
4265	828.75	80	1786.15	8323.95	998.6148	6414.746	6163.264	5280.486	6201.468	1824	9844
4266	828.81	83	17261.3	13873.08	21540.06	8010.877	14268.45	7710.728	12329.24	15813	15259
4267	829.42	79	6237.94	4366.892	6503.209	2523.06	3541.714	2840.761	4628.249	5516	3387
4268	829.81	83	2254.2	4223.233	6531.857	3921.92	4525.245	2375.441	3503.053	5496	3805
4269	829.85	84	2264.38	3534.862	2749.791	3579.636	3330.594	2574.407	4024.98	5250	4052
4270	830.37	75	3770.04	3048.735	5005.436	1415.75	2379.52	3735.372	1228.59	2067	3784
4271	830.8	82	7475.39	6784.65	1150.554	5862.531	6060.896	3973.458	5546.033	8010	5075
4272	832.36	73	2873.89	1286.747	1993.351	1201.776	725.256	3567.355	894.834	2261	4105
4273	832.58	97	2965.49	2651.319	1733.973	2189.449	804.2624	1640.377	1625.367	2071	2704
4274	832.58	467	1919.53	1114.241	2387.77	446.1711	890.7513	1204.654	1924.063	5899	1536
4275	832.79	81	2018.23	2268.942	5057.586	2307.588	512.2367	1941.308	3234.669	2761	1316
4276	833.82	83	3311.97	1738.954	2917.398	2303.186	929.28	2361.686	3046.008	1235	2760
4277	834.6	468	1700.9	1401.437	3190.421	1890.318	981.1999	1063.914	995.5442	4921	1186
4278	834.6	309	905.199	1039.155	970.4716	900.0625	336.4111	465.7793	566.5616	944.6	920.6
4279	834.6	114	2576.03	2766.06	3013.944	1743.733	2840.186	2584.179	1592.465	1905	3325
4280	834.79	81	4807.58	3324.844	3993.613	2052.344	1281.43	2775.655	3228.705	2494	3291
4281	835.6	113	1246.72	2459.719	2561.032	1428.965	1061.484	1434.358	1718.99	875	4441
4282	836.41	71	1198.6	1947.668	1055.466	1460.592	876.2674	1576.729	1353.462	1249	1945
4283	836.58	109	2694.64	1323.938	3112.993	2291.277	1819.111	2045.08	3698.37	2378	1124
4284	836.61	471	1684.57	1206.678	3368.392	1223.406	889.0854	1130.654	989.3203	1653	2096
4285	836.77	81	2795.16	2938.342	6998.509	2139.792	2043.812	2267.057	1777.465	1835	3046
4286	838.4	71	2863.07	2740.598	1355.6	2268.5	1527.042	2654.82	2494.917	2536	3084
4287	838.56	1043	1537.28	1106.471	759.5175	956.7231	363.6826	616.1786	483.5106	1345	2283
4288	838.56	1073	2104.57	1272.765	1707.558	1511.879	735.0737	1630.049	933.8941	2192	1936

4289	838.56	1150	416.914	780.6188	1242.233	784.7329	182.8171	763.9655	175.14	554.7	829
4290	838.56	109	1364.17	1561.698	927.1143	876.2346	811.1885	597.7692	1758.96	1474	1273
4291	838.68	77	2409.51	1886.135	3708.453	554.7269	354.5781	921.0541	1039.05	1316	1169
4292	838.84	85	42306.9	40503.23	51749.13	21624.11	39326.51	29322.49	35891.53	40442	35563
4293	839.35	81	7929.72	1999.087	4377.941	766.6633	5408.092	1235.356	2289.104	6273	2509
4294	839.84	85	7761.44	10137.96	9962.851	3063.943	10450.06	6400.005	8347.5	9780	7281
4295	840.34	80	4203.97	2055.317	1551.787	1552.25	2873.88	2439.67	2006.292	1804	2525
4296	840.4	71	3967.61	3251.937	1946.872	1660.442	1814.153	2633.464	2870.154	3500	4987
4297	840.56	106	1803.39	1050.757	718.08	1173.776	925.7231	952.6554	835.7873	1639	1176
4298	840.84	86	6040.01	3471.533	4617.894	1787.288	3472.636	2851.988	4558.917	4830	1540
4299	841.81	83	3751.11	2476.736	5128.939	1278.128	512.2367	1856.352	2293.992	3469	2283
4300	842.4	71	2992.75	2992.933	1744.752	2204.108	1688.7	4098.173	2230.45	3242	3923
4301	842.69	79	2508.47	1041.758	1768.012	1347.614	246.4886	1536.961	1323.863	630.6	617.8
4302	842.82	83	8457.5	5775.058	8996.151	2808.416	6504.726	2781.295	4785.507	7982	5591
4303	843.86	84	3069.11	1015.45	3849.1	509.82	1025.541	1979.656	2236.815	1504	1762
4304	844.39	72	1406.6	2050.038	1327.573	1582.382	1172.713	1179.23	2168.271	1972	3422
4305	844.79	82	5536.93	3226.082	2875.439	3848.083	3354.064	3526.79	7254.643	8278	6558
4306	845.84	83	1528.8	3167.404	5911.291	2299.218	1793.3	2419.171	1746.09	2501	3232
4307	846.38	75	2481.35	1693.99	2051.47	4408.3	702.0675	3382.986	1279.168	1017	2022
4308	846.44	78	7104.8	7233.903	10025.59	8032	7810.396	5735.327	8415.444	7159	2161
4309	846.82	82	3571.7	4776.666	6017.218	2046.437	4006.131	2130.295	6069.587	3996	4696
4310	847.32	84	2589.96	503.072	2312.205	1021.67	512.2367	899.012	3067.221	1999	1768
4311	847.44	78	3098.8	4230.786	4238.26	5860.378	5569.566	2826.304	3864.067	4939	1088
4312	847.82	83	2022.46	760.928	3693.738	1021.128	768.558	2089.664	1510.577	1780	1991
4313	848.45	79	2418.07	1792.669	2832.042	1541.513	1543.254	2236.016	2659.817	1528	2020
4314	848.53	96	1901.02	2233.02	1720.374	1945.09	1667.378	1499.849	1373.281	1327	1524
4315	848.81	83	3299.4	2523.514	2453.653	2050.02	2728.088	1198.26	2270.914	3290	1998
4316	849.85	83	3820.16	2403.407	3335.217	2043.512	2143.634	1882.98	2023.086	1719	2543
4317	850.55	96	3662.18	6882.12	2141.978	1067.005	1992.438	1220.886	3731.811	1481	3826
4318	850.6	118	2504.75	2413.092	3561.374	2794.116	3781.639	3638.6	3286.474	1753	3820
4319	850.8	82	2535.83	2403.963	2744.588	1280.16	1454.017	1406.529	1492.423	2494	1809
4320	851.4	80	33668.2	26449.9	34658.99	18008.54	40642.3	15164.7	29069.6	33117	11929
4321	851.55	96	3975.94	1909.29	1649.825	500.75	1630.88	471.7448	3926.443	677	1726
4322	851.61	116	1478.54	1711.24	1500	1009.788	1262.518	1934.523	1027.812	1101	1963
4323	852.4	80	18540	17497.34	19324.99	11168.7	21722.19	9793.79	17811.89	22960	10312
4324	852.61	64	780.167	355.3412	757.375	824.9346	148.6117	487.5512	653.0579	561.6	585.4
4325	852.71	79	1574.61	795.7339	1472.118	1559.857	526.1829	771.3878	1022.694	836.4	1046
4326	852.85	84	14000.3	17694.18	20689.44	4083.256	18522.99	9613.393	12659.22	14218	14577
4327	853.4	80	8521.13	5045.508	6622.475	4960.221	10307.32	2995.667	8302.876	8766	2805
4328	853.84	84	8256.66	5498.64	3590.067	2813.306	4618.784	1619.25	2788.586	6947	6471
4329	854.38	74	3971.51	3499.168	2020.686	3101.763	940.2691	2793.192	2844.162	3096	4752
4330	854.81	84	16365.3	11942.94	21932.74	8406.885	11671.63	11899.23	13137.63	17645	14298
4331	854.86	86	15714.9	8201.809	15904.09	6891.36	4875.421	11668.8	2267.226	16671	12272
4332	855.81	83	2059.6	4546.263	6615.517	3326.404	2694.164	4954.089	2776.777	4308	5544
4333	856.37	71	3596.04	3661.653	2950.785	2846.016	1539.348	5304.678	2381.44	4546	4196
4334	856.81	83	4081.54	3790.77	3790.277	3070.091	2508.475	3304.706	3539.136	4465	5572
4335	858.37	72	3547.37	3779.573	2487.775	2989.872	1229.637	3080.805	2762.304	3641	3869
4336	858.68	76	2778.36	1595.573	1523.73	784.1315	322.9477	992.7253	1571.333	2276	1315
4337	858.79	81	2740.15	3524.829	6461.316	2303.186	2310.637	2832.267	3067.504	2705	2256
4338	860.37	73	1549.4	2391.207	1877.4	3283.069	1356.952	2461.142	1797.405	1958	2668
4339	860.65	116	414.247	596.8105	532.1671	1994.858	924.935	324.564	748.143	1080	433.2
4340	860.75	82	2796.43	1625.437	5997.461	2052.012	1217.28	1637.03	3778.694	4762	3033
4341	861.82	84	3571.7	733.9967	2921.78	766.5667	512.9133	1874.73	2259.29	2748	2357
4342	862.36	71	1561.59	914.3619	667.4171	876.8925	672.1569	575.0244	528.93	1185	1531
4343	862.8	82	2251.25	1029.454	3331.881	2308.85	767.832	1361.667	2262.118	1984	2550
4344	864.63	143	2138.72	1112.165	1491.525	2299.995	1941.288	656.1333	760.5	1819	1997
4345	864.8	82	1708.56	514.7533	1537.505	1280.16	511.3125	919.48	1005.039	1023	1485
4346	865.82	83	1736.64	1238.067	4107.319	766.122	256.186	1829.067	1500.447	1219	1998
4347	866.7	80	1503.13	1557.173	1470.71	1036.123	341.8489	518.9759	2361.6	3131	1566
4348	866.86	84	4083.83	2013.48	3083.273	1278.9	3804.775	2091.045	3321.968	3534	3862
4349	867.37	79	89240.4	63391.75	96614.5	39788.58	76423.52	18282.42	61200.93	69099	18431
4350	867.84	86	2035.03	2212.56	3200.802	1019.872	768.21	3773.625	2562.384	2514	2498
4351	868.37	79	53633	36225.88	53206.01	23648.7	38513.79	11785.5	39006.48	35823	11946
4352	868.82	83	7145.11	6782.108	9985.61	2052.012	5795.591	4234.093	5072.693	2740	6293
4353	869.37	80	25281.5	18346.15	28666.53	9286.998	17883.1	5783.583	17468.17	17716	7257

4354	869.48	73	1381.83	1158.504	821.3193	1633.683	913.9879	1122.498	1013.345	1005	1506
4355	869.81	84	2530.78	1276.128	4211.189	1790.947	256.07	1360.643	1495.146	2237	2859
4356	870.37	79	10008.9	6467.122	10294.19	5584.785	7909.257	939.6716	7418.014	9730	2809
4357	870.78	82	4797.67	3802.206	9968.275	3074.4	3150.045	4742.336	6064.533	6721	5324
4358	870.86	85	1035.82	246.1875	256.8458	764.295	2317.194	1219.76	1528.875	2258	993
4359	871.37	80	3259.79	1977.007	3022.102	1323.765	4879.491	515.5333	3053.814	1773	1296
4360	871.48	71	1142.5	1729.224	925.7117	1680.912	737.051	1024.812	601.2886	899.2	1773
4361	871.57	125	911.015	1594.93	713.5767	7783.71	1399.874	2606.515	913.9223	1195	915.2
4362	871.79	83	1526.73	1248.173	3678.901	1022.927	768.355	1668.78	2499.775	2486	3025
4363	872.35	73	2872.16	2979.357	1749.968	2285.112	1268.842	2788.606	1385.365	1489	3176
4364	872.83	84	3724.38	2956.305	1981.512	3833.029	2052.143	3335.133	2253.262	7651	2658
4365	873.33	84	2817.49	1459.369	2307.153	1020.8	256.36	680.57	2301.26	3030	992.2
4366	873.82	84	2553.99	477.792	2455.843	509.936	256.186	1450.592	1025.408	1269	1480
4367	874.35	72	1713.4	1777.708	1180.379	1632.865	1891.127	2547.236	1817.131	1667	2932
4368	874.57	98	0	364.1721	120.7573	1288.656	1185.689	30.10667	225.7588	318.8	0
4369	874.82	83	2538.53	1237.736	1280.16	1534.199	768.21	1728.966	1486.968	1527	1773
4370	875.8	82	3281.56	982.2608	2821.106	1021.67	256.1183	1129.867	3276.134	4506	1996
4371	876.34	74	1341.9	2011.933	932.1813	722.1082	962.559	970.0299	829.3244	2231	2918
4372	876.56	98	1804.17	3106.076	1443.663	254.04	1267.016	485.5432	710.7296	1116	2067
4373	876.66	77	2412.9	2087.441	1008.255	1043.277	524.8	777.8517	1597.255	1918	1309
4374	876.8	82	3054.73	981.64	3786.161	2045.469	1280.169	1821.6	3036.602	3759	1737
4375	878.34	82	1184.4	630.5685	908.9433	645.0227	300.5782	862.1685	694.5957	905.6	782.8
4376	878.73	80	2304.73	2317.695	3725.962	1283.115	0	1236.267	3554.226	5247	1552
4377	879.78	80	1527.53	2261.34	1980.242	1539.233	1540.003	1151.833	1771.56	977.8	2002
4378	879.83	85	3877.86	968.7771	3845.551	1786.881	1590.04	1173.194	2301.997	3032	3507
4379	880.81	83	2563.22	2695.579	5898.217	2300.476	5014.76	2084.388	4089.04	5522	4228
4380	881.86	84	3080.31	2223.339	3787.936	1282.192	2295.202	2681.67	2790.9	3795	4045
4381	882.68	77	2530.01	1352.414	835.8768	717.2849	354.9279	132.9972	1188.2	1257	986.3
4382	882.83	84	2781.38	1494.523	1026.206	2815.424	1329.912	1640.333	2511.025	3928	2292
4383	883.79	83	3002.52	1531.968	2150.955	1534.76	745.6167	1898.765	1770.148	1990	1999
4384	884.59	104	762.613	530.1855	1099.164	691.74	1239.808	1107.792	537.6963	2630	892.1
4385	884.8	80	1757.44	1999.253	5495.653	1795.826	2392.716	703	768.6	2509	2226
4386	885.45	73	1463.35	1041.248	827.6022	1095.526	491.4378	822.0364	957.906	872.3	1002
4387	885.55	131	6216.63	2892.272	9859.125	1483.508	1948.832	1534.975	1937.88	475.6	1510
4388	885.55	1051	7912.15	1305.189	7124.859	2146.242	1226.567	1300.8	1288.875	1465	1638
4389	885.55	1074	2923.48	689.6029	3305.337	2426.32	713.7833	1162.214	918.215	1710	1143
4390	885.55	208	16120.5	688.2118	17362.61	667.3875	4798.598	549.7215	2290.343	650	1005
4391	885.55	182	12092.5	570.78	11761.92	419.87	3308.472	549.9651	1129.586	314.3	573
4392	885.55	1096	1826.21	548.4848	1984.867	2282.116	682.5543	616.4867	660.2165	2000	816.6
4393	885.55	276	6356.1	260.078	8597.353	374.0563	468.6596	188.1429	1359.206	244.6	273.5
4394	885.55	232	12322.1	427.7903	13783.98	433.8843	2392.027	419.3143	1764.235	361.5	546.9
4395	885.55	155	10406.5	1115.897	9831.135	736.1575	1289.303	696.6189	1011.411	471.6	1136
4396	885.55	253	10293.3	342.6562	10366.32	420.7035	1231.641	380.5637	1963.448	319.5	412
4397	885.8	83	1996.94	512.9133	1542.848	1533.133	256.186	449.6667	1252.272	1965	515.6
4398	886.55	1054	7324.49	1584.15	7458.786	1242.498	1354.156	1609.832	1852.228	2399	1767
4399	886.55	276	4118.87	204.7772	5502.366	214.6573	360.541	171.847	874.4356	167.3	251.2
4400	886.55	136	5183.33	1320.26	16184.72	955.2063	1049.815	955.459	1530.9	1032	1326
4401	886.55	231	7624.42	314.7715	8802.218	326.2629	1541.449	304.8764	1172.21	260.8	391.1
4402	886.55	209	10063.7	252.4721	10939.7	353.43	3032.011	329.1083	960.9732	327.5	561.2
4403	886.56	253	6855.06	283.187	6788.053	329.8575	823.1824	267.6157	1188.376	206.5	250
4404	886.77	81	5567.35	4511.995	7886.24	3331.44	1414.4	3594.629	4034.289	1867	2296
4405	887.45	74	1621.34	626.2398	529.5881	1251.936	587.2238	1089.939	900.8333	1238	1424
4406	887.56	209	4803.12	610.9916	4450.752	1450.669	3691.053	861.4554	1401.81	1585	624
4407	887.56	1052	4408.6	1357.767	3869.58	1337.079	1370.752	1858.421	1831.055	2955	811.8
4408	887.82	83	4049.85	1498.707	2663.373	1535.457	1280.35	1620.504	1793.534	1220	2254
4409	888.77	80	3831.46	4365.599	2583.193	2308.85	1228.8	1881	3210.699	2976	2284
4410	888.8	85	5375.6	1482.179	3592.549	1786.881	512.14	1875.343	4050.319	4727	2531
4411	889.81	82	1990.19	1286.383	1893.251	1024.38	1022.874	467.7075	2041.665	2494	999.2
4412	890.78	81	1498.42	1983.52	3180.367	511.56	1791.567	914.4683	2259.588	1694	2033
4413	891.79	79	1215.24	1792.656	677.4042	512.575	256.2875	1192.718	1765.94	974.1	1811
4414	894.63	116	1998.04	1772.43	2592.63	1016.698	2131.274	1817.589	2469.35	1225	2098
4415	895.64	115	762.693	642.0333	727.224	1748.87	987.0357	497.7	824.3643	892.7	1293
4416	896.36	72	2541.53	1834.262	1139.741	1518.379	1452.029	2994.998	1644.554	2331	3533
4417	896.79	84	11366.9	11876.68	10542.56	6371.167	14011.68	7337.65	9283.196	2836	10306
4418	897.8	83	2561.54	2266.864	6169.584	1790.947	1795.013	2084.631	1515.67	3474	3470

4419	898.36	71	2944.01	3097.604	1983.296	2805.84	1710.488	2682.049	2381.167	2930	3824
4420	898.79	83	4812.6	6018.338	8349.542	2052.012	5238.342	5757.448	3790.757	5019	5321
4421	899.78	81	3002.52	1017.864	3011.337	2048.76	769.6167	1220.137	1492.194	1707	1516
4422	900.35	71	2979.84	3114.588	1289.409	2578.135	1200.012	2330.008	1882.962	2207	2663
4423	900.78	83	2253.71	3296.59	4383.365	2048.212	256.2311	1455.052	4301.65	2266	1289
4424	901.81	84	1498.42	1752.243	2308.088	1538.378	1537.972	1425.655	2531.386	2768	1252
4425	902.35	71	2477.07	2953.894	1082.113	2124.695	1014.098	1130.208	939.25	1487	2745
4426	902.57	1048	1249.17	2266.196	620.5693	1640.118	1681.291	3645.228	403.5745	1375	2024
4427	902.74	81	3286.47	2836.069	3075.539	1794.564	769.776	3274.06	2011.492	2700	3362
4428	903.57	1070	1221.7	1508.42	682.3031	2429.197	1594.343	2931.185	918.9619	1285	1818
4429	903.79	83	1501.26	1017.703	1974.44	1282.192	1588.931	1386.84	1273.596	997.8	1296
4430	904.58	115	1537.23	1292.248	1759.932	1325.857	1008.95	1960.002	2107.988	3138	484.4
4431	904.59	133	1176.66	1392.846	2518.471	542.5588	999.3694	1706.865	1631.7	2649	455.7
4432	904.59	1051	3232.81	2135.913	1589.878	736.23	3538.167	2614.278	1532.317	2457	1337
4433	904.59	1098	1582.4	865.1575	704.2667	1660.157	1622.885	1489.241	620.0195	927.7	539.4
4434	904.59	1072	1872.34	1276.675	987.9938	1175.308	1282.583	1982.624	877.7459	977.1	747.7
4435	904.76	79	2911.61	1802.812	4314.371	1795.352	876.5167	1963.566	2753.711	2486	2073
4436	905.68	1076	942.972	1231.543	807.9106	1153.423	1184.095	763.952	855.5222	767.9	1139
4437	905.68	1101	980.632	1033.344	1135.184	919.665	1091.491	999.5995	914.8218	1124	1332
4438	906.82	85	28818.6	27509.93	35336.52	18219.55	27303.41	16500.64	25512.96	27894	25619
4439	907.32	85	1268.18	770.6333	1535.94	1019.872	512.372	680.57	1281.192	2256	1222
4440	907.53	180	5071.11	258.3762	4744.882	225.7067	603.9534	268.6421	285.3635	196.8	314.6
4441	907.53	128	3927.74	562.9264	2406.594	437.4429	514.0607	278.3021	526.68	114.4	323.8
4442	907.83	85	5804.61	6936.3	7708.037	3324.116	5827.009	4852.235	5598.889	7051	6777
4443	908.53	182	2986.32	109.2618	2580.995	159.896	462.0683	122.7395	308.8945	126.8	147.6
4444	908.53	274	1277.31	92.45115	1482.754	10321.21	93.89797	254.528	221.6638	67.59	57.62
4445	908.54	253	2444.59	78.17875	2342.48	734.1002	216.63	581.7526	429.4717	102.2	102.6
4446	908.54	208	3040.42	202.0774	3009.255	194.5361	922.2608	344.3231	437.4413	279.2	275.9
4447	908.65	126	3927.08	1255.414	2074.81	1612.56	1384.277	1385.887	1672.648	2045	2017
4448	908.82	84	3566.36	3400.74	5072.345	2039.727	3419.646	2542.34	3814.003	3734	2540
4449	909.81	83	3519.49	1974.665	3009.611	1023.364	1801.2	1635.72	2531.773	2736	2573
4450	910.34	75	773.168	2163.679	2170.843	1399.966	1302.72	2049.689	1515.47	1114	1628
4451	910.81	83	5312.03	4740.039	6423.064	2814.166	3613.066	3321.848	3568.936	4912	3807
4452	911.8	85	1240.88	1226.234	2057.792	510.4	256.1183	1355.314	2029.408	1226	2023
4453	912.33	73	4059.15	2491.931	1439.931	2872.595	1125.985	2234.727	1436.532	2520	3986
4454	912.77	82	6330.13	6001.946	9148.445	4091.016	5615.518	5002.476	4093.691	5909	6808
4455	913.82	83	3554.34	1740.921	3270.213	2046.437	1796.46	2102.351	2009.459	2722	2799
4456	914.33	74	4140.89	3405.35	2385.097	2840.879	1284.217	3384.231	2296.638	3246	4516
4457	914.59	76	929.778	519.0268	872.4342	259.3325	96	21.80675	304.545	556.4	260.3
4458	914.8	82	2511.62	2878.443	6274.145	2555.771	2126.104	3036.86	2598.564	3443	3017
4459	915.81	84	1781.81	757.26	2915.626	765.55	256.186	931.11	2038.832	1456	992.2
4460	916.33	72	5439.56	3725.055	1882.907	1530.944	699.4	2796.872	1728.108	2636	4586
4461	916.8	82	2277.99	1752.243	2927.173	1534.76	256.186	2110.74	1765.94	1978	997
4462	917.82	83	3266.55	1533.126	3854.091	1790.947	1537.681	2092.2	2006.648	2465	1959
4463	918.32	72	2978.63	3460.605	1628.107	2572.18	1441.839	2462.61	902.718	2925	4685
4464	918.73	80	1810.06	1289.048	2923.264	1027.675	0	975.9125	1773.11	2255	517.9
4465	920.32	73	1132.3	1734.425	776.1629	1631.664	583.251	1180.352	894.9356	2063	1440
4466	920.74	80	1760.67	4487.21	1954.739	3078.467	6664.123	4603.067	4526.518	5392	5257
4467	920.84	85	9616.54	10329.98	11931.1	2310.722	12291.07	8165.61	7374.795	11061	8577
4468	921.83	84	3566.36	5041.316	4871.243	2559.039	3790.56	2563.392	3308.058	2448	3785
4469	922.01	115	1877.08	1905.917	2727.513	2075.236	2567.928	2503.24	705.6267	3112	2003
4470	922.8	83	12610.4	7972.688	19281.85	5885.915	9899.54	7060.287	12427.84	13185	7775
4471	923.8	82	4054.35	3622.578	5129.504	1282.192	3450.138	2516.98	3835.861	1235	3606
4472	924.67	79	778.767	626.2086	1126.885	0	84.822	257.404	1568.895	2549	1042
4473	924.8	84	3572.97	2984.54	6668.229	2301.733	2702.97	3540.05	4304.699	2793	2768
4474	925.8	83	1242.82	1495.65	3078.711	1278.03	768.355	2092.96	1259.67	1461	2249
4475	926.79	81	3779.25	2541.554	5589.278	2050.02	1764.18	1449.611	2267.764	2854	1810
4476	927.66	1072	957.246	1295.587	899.6329	1117.629	1100.038	747.967	916.92	754.6	1317
4477	927.66	1114	1176.21	858.8	1152.152	1075.953	1237.808	964.5729	1387.035	1289	1195
4478	927.66	108	2603.74	2611.903	3128.341	2819.823	3277.194	2310.912	2081.983	2239	2573
4479	927.66	1100	1113.14	929.88	1096.474	1068.468	1175.614	984.6759	1436.119	1074	1361
4480	927.77	82	1786.49	1073.322	2149.293	780.709	1609.509	2098.743	502.802	720.8	1395
4481	928.31	73	1666.68	1234.846	1535.94	1782.193	398.5936	1611.09	1530.827	2363	2056
4482	928.66	107	1421.92	1092.252	1064.846	1118.88	1122.924	2542.074	631.9544	1576	1518
4483	928.76	81	3257.6	2048.064	5083.34	2565.646	1627.75	2356.629	2753.003	2730	2258

4484	929.81	84	2301.05	734.384	1472.735	1277.352	0	1407.48	1794.799	1741	1509
4485	930.3	73	3001.52	2246.008	2122.113	1996.108	849.42	2526.93	1426.303	2872	3051
4486	930.79	82	2509.74	1468.768	2899.765	1539.64	1025.735	1390.65	1744.665	2421	1999
4487	931.79	84	1526.73	1237.736	2150.955	255.78	256.1183	1148.003	1274.023	1739	1810
4488	932.3	72	2598.09	1539.384	976.08	2138.4	603.0275	2932.402	1261.97	2446	3128
4489	932.72	79	1427.18	514.7268	1554.944	514.9433	211.5333	0	1294.096	1235	1039
4490	934.3	71	1891.42	769.692	926.9663	1197.3	849.5856	1061.151	1870.184	1580	2666
4491	934.64	1070	1204.14	920.9906	1012.489	776.5429	686.668	1148.106	1475.172	1326	1452
4492	934.72	80	1810.06	1029.454	2567.94	1282.7	0	978.618	1513.615	2507	1035
4493	934.85	84	2553.99	2448.18	4100.772	1529.168	1789.2	1866.245	1774.684	2515	2253
4494	935.8	85	2840.99	2464.763	3180.828	1276.773	256.4567	1388.46	2042.058	2520	2542
4495	936.82	83	4390.39	4220.186	8860.805	2874.184	3935.505	2581.648	3274.601	3972	4032
4496	937.7	79	1810.06	514.7268	1548.138	769.82	0	231.312	255.4146	254.6	517.2
4497	937.8	84	1756.63	980.3467	2564.968	511.4633	1027.09	2410.794	1281.192	2233	1744
4498	938.66	115	1391.04	811.5714	1000.16	924.84	1634.544	1419.405	683.8233	820.3	925.2
4499	938.77	82	2457.55	3764.251	5325.899	2304.54	1410.079	3928.264	1986.109	1272	4370
4500	939.59	1072	1189.96	842.5697	1154.974	956.6327	882.3282	1214.414	1275.51	1439	1459
4501	939.78	82	1266.97	1274.862	2307.426	769.82	512.372	1365.25	1744.665	1198	1485
4502	940.81	84	2018.23	1717.574	5654.618	2304.444	2550.692	2366.887	3280.451	5479	3908
4503	941.31	86	2272.03	477.036	1282.192	0	256.186	444.928	1531.86	2513	1696
4504	941.81	84	2248.9	760.5267	2818.583	766.5667	256.1183	698.28	1516.935	732.4	773.7
4505	942.78	82	1266.91	2255.769	4557.558	766.5788	1281.43	1868.47	1524.233	2008	2545
4506	943.79	83	1781.81	1745.237	2819.596	1021.496	512.72	1634.721	3262.753	2213	2031
4507	944.78	82	2013.94	1272.596	2920.832	1534.416	770.125	1914.491	2011.492	2698	1292
4508	945.79	83	3306.52	512.9133	1537.444	1022.754	256.186	2103.933	1225.682	1246	774.7
4509	946.72	80	3223.33	1287.127	2472.151	515.3638	168.245	721.0043	1497.065	1985	1298
4510	947.83	85	2794.97	1494.523	2314.75	509.936	1026.006	1595.165	2286.396	3225	2021
4511	948.81	84	3848.1	3966.749	3693.738	1532.457	1025.44	3071.269	1777.465	3283	4286
4512	949.3	85	2042.83	0	2562.578	0	512.9133	1363.67	1537.178	1269	1510
4513	949.85	86	1503.13	1750.98	4254.028	1020.413	1025.827	1476.352	2054.718	2736	2247
4514	950.8	85	1493.32	1679.92	2663.193	1278.89	1334.853	913.6913	2259.29	1984	2029
4515	951.8	84	2509.74	982.2608	1792.98	0	256.186	2069.54	1531.86	2236	2228
4516	952.79	84	2255.97	982.128	1796.538	767.075	256.2875	1171.632	2269.709	2754	2223
4517	954.32	71	2230.43	2555.782	825.5	1702.8	1102.506	2534.666	1731.762	1980	2935
4518	954.76	81	4559.39	3795.701	4468.231	2052.012	768.355	2357.832	4080.926	4491	3038
4519	955.57	1070	1695.05	1153.713	1246.747	1318.195	1144.529	1285.342	1482.918	1650	1576
4520	955.81	84	2251.25	1226.697	3168.848	1279.967	1544.52	1407.48	2564.149	2751	3219
4521	956.31	72	2822.64	3126.359	1776.522	3054.084	1942.309	2776.249	1961.56	3684	4563
4522	956.79	82	3286.47	2038.26	4888.647	1534.199	1681.564	2136.14	2291.826	3267	3805
4523	958.31	71	4089.4	3656.395	1170	2429.307	2239.38	1862.884	2619.668	3740	5805
4524	958.72	86	1013.73	6054.3	1281.43	255.0188	0	1416.365	2009.085	1750	1285
4525	960.31	71	2380.34	3060.954	1022.336	2810.53	1298.101	1960.92	1243.987	2562	3349
4526	962.31	71	1201.02	2242.083	748.66	922.5147	809.9779	789.1164	864.7544	1212	2736
4527	964.3	72	1727.23	1166.745	2267.901	1535.751	707.2419	1264.334	1504.126	911	1168
4528	964.78	83	8835.75	8925.424	9624.904	6129.28	6068.466	5300.601	7293.624	11236	7835
4529	965.79	82	3746.15	3402.406	5244.769	1794.09	1709.29	2153.03	2254.53	2507	2258
4530	966.3	73	1375.2	651.0135	523.3675	871.9021	1530.912	501.2607	911.588	887	1415
4531	966.78	82	3633.47	2245.767	5788.663	2557.514	3856.439	2860.228	1776.201	2956	3269
4532	967.78	84	1503.13	749.552	2235.077	509.936	256.186	914.228	1017.864	1955	1252
4533	968.3	71	1376.7	1536.872	701.0284	1289.107	1112.1	1013.651	839.8222	1685	2130
4534	968.76	81	3227.35	1532.314	2658.894	2050.02	1281.611	1873.25	1765.142	1751	1807
4535	969.8	84	2044.1	1445.597	2049.531	1020.896	769.08	2172.114	2279.765	2231	2250
4536	970.3	72	3799.53	1926.831	1235.512	2329.391	1046.581	3055.656	2105.04	2406	2752
4537	970.78	81	2511.05	982.2608	2738.927	1539.64	0	1690.37	2011.492	1962	1484
4538	971.79	81	2611.17	1249.82	1280.16	255.78	512.2367	943.64	1986.312	1467	1448
4539	972.29	72	3527.51	3916.713	1684.445	3259.44	1527.76	3841.908	2428.358	3289	5668
4540	972.48	137	1262.94	594.72	1722.12	111.8433	1010.592	192.66	1529.64	2208	1471
4541	972.74	81	2536.53	1755.665	2677.141	1282.192	512.372	1643.328	1455.086	1998	1775
4542	972.97	137	1374.19	786.24	2123.063	109.185	1356.272	57.0375	1573.74	2756	2020
4543	974.29	72	4454.83	3691.458	2804.107	3623.405	1613.753	3688.489	3039.754	3390	7259
4544	974.81	85	21713.7	15760.85	22782.8	8658.866	16511.9	11987.89	17976.98	19721	20631
4545	975.31	84	1825.96	477.792	1798.957	254.968	512.372	1175.436	1001.406	1526	1028
4546	975.81	85	2509.74	6995.383	5906.507	2555.771	4746.421	1915.934	5108.92	5316	3965
4547	976.29	72	3296.31	3538.411	1997.728	3120.969	1458.456	2732.464	2435.342	3032	3543
4548	976.81	84	2764.09	4134.654	3333.484	1535.675	2354.854	1616.7	4013.296	2751	3509

4549	977.43	136	677.611	272.16	873.76	185.055	348.16	29.21	284.25	1335	1056
4550	977.79	83	2563.53	1788.71	2311.191	1279.031	1280.028	1404.15	2034.881	3202	2307
4551	978.28	73	2654.82	2082.495	3090.868	1128.918	1743.933	2939.833	1020.345	1258	2708
4552	978.79	84	4874.31	1925.364	6363.195	3358.649	5519.36	3029.341	3557.065	2553	2982
4553	979.79	83	1500.77	515.417	1792.98	510.748	0	878.84	1281.655	1246	1481
4554	980.27	72	854.921	1254.022	811.509	984.1	478.5744	629.694	1013.111	1905	1629
4555	980.77	83	2435.88	3069.996	4349.078	4453.338	1501.888	2829.782	4779.54	4586	3005
4556	981.28	74	481.77	299.6956	216.4525	77.10317	452.5367	350.2671	208.29	459	319.1
4557	981.79	82	4110.53	1017.864	2828.527	2050.02	512.2367	1430.304	1273.049	3529	2038
4558	982.29	74	470.546	1149.478	1330.503	507.3078	460.4175	691.3748	635.1894	1251	1548
4559	982.79	83	3029.57	2553.522	2057.225	2305.8	1402.88	1154.43	2213.231	4271	3763
4560	983.81	84	1810.06	1249.721	1890.81	512.6673	811.8382	701.52	1023.295	2789	1225
4561	984.27	74	1366.7	504.3981	624.6398	1259.27	628.614	767.5074	489.5833	886.2	913.4
4562	984.77	81	2046.04	1769.906	2923.804	1290.46	724.176	963.93	1012.388	732.8	2002
4563	985.79	82	2536.53	1769.868	2819.88	1790.134	512.372	1390.65	1782.735	1221	2033
4564	986.27	73	2218.89	1645.413	1594.279	1070.486	768.393	1134.083	906.2367	2124	2164
4565	986.5	136	884.94	583.492	787.1175	185.055	574.2	44.3625	379.26	911.8	1053
4566	986.6	118	4663.41	753.2571	4070.925	733.7385	728.9488	1698.3	1013.948	2501	2291
4567	986.74	81	1524.88	1254.76	1317.066	2064.482	1503.68	1668.393	2293.611	1777	1899
4568	987.5	136	606.816	445.77	730.482	195.195	405.76	13.9425	473.76	272.9	1510
4569	987.59	119	5626.12	711.77	5345.611	805.563	608.1007	1064.929	1028.869	946.2	2083
4570	988.26	72	2249.47	2632.756	1480.489	2546.445	1081.409	2709.445	1292.618	2504	4282
4571	988.6	120	2399.12	0	2145	217.6065	406.848	419.16	97.89	37.32	726.6
4572	988.82	85	8860.73	8910.569	9197.666	3579.636	7839.354	4436.716	6861.706	6318	7822
4573	989.81	85	2529.84	978.8686	3417.405	509.82	2871.285	2361.543	2824.373	2480	1989
4574	990.26	72	3400.91	2183.219	2081.807	2330.944	1603.075	3100.6	1208.557	2630	4407
4575	990.78	83	10621.3	7506.914	13847.39	5117.351	8470.544	5362.091	7101.011	9485	8161
4576	990.83	86	8286.75	2673.72	7944.3	1529.88	1790.134	4678.502	5882.656	3805	1000
4577	991.55	118	20465.3	3734.773	18061.49	1945.337	3950.791	7155.533	5015.541	6910	8384
4578	991.79	83	3532.59	1996.836	6941.539	1789.943	3083.98	2698.181	1771.064	3240	2991
4579	992.26	72	2252.66	2134.819	903.4988	1658.951	613.8774	2257.477	988.642	2114	2767
4580	992.56	118	12474.3	1610.143	12829.12	1728.601	2903.593	6772.48	3381.142	4560	5969
4581	992.79	83	3824.46	2770.398	4882.832	1535.94	3693.387	3278.168	2008.82	1765	3206
4582	993.55	118	4546.58	1203.848	6572.269	1316.745	1512.366	1672.465	2104.635	2470	2860
4583	993.77	80	490.992	1286.256	1208.077	768.558	512.575	955.83	0	252.1	1294
4584	994.26	73	1383.9	2211.37	964.8371	1744.416	713.2213	1613.47	1242.33	845.5	2566
4585	994.56	118	1575.8	111.3	915.42	89.2416	887.328	484.8	554.778	329.5	1584
4586	994.77	83	2798.63	2774.902	2817.36	1538.378	1875.129	1407.48	2041.714	3215	1512
4587	996.73	80	778.186	1032.733	2302.231	1538.378	1605.523	2649.22	2257.206	2034	1300
4588	997.79	83	1812.9	2012.486	3845.551	255.374	256.186	427.99	1761.112	1722	1741
4589	998.76	82	1294.98	1974.514	3761.169	1795.826	256.1183	1131.353	2507.946	3015	2740
4590	1002.2	75	625.328	406.2552	484.1913	1004.739	914.0282	782.4837	992.4136	638.1	1544
4591	1002.8	84	2785.21	3533.633	2052.825	1022.588	769.3625	1407.688	1272.596	1995	1027
4592	1003.3	75	247.68	462.1851	193.4169	117.6721	380.656	140.2235	219.98	420.8	353.3
4593	1003.8	84	1526.73	1447.78	1282.192	1533.133	1282.192	1219.76	2004.078	2505	2242
4594	1004.2	74	1174.49	683.8853	1102.92	1123.2	272.9168	1221.814	577.5	856	1855
4595	1004.8	84	4042.19	2727.962	4874.404	1786.388	3082.805	2177.637	2784.265	4747	2513
4596	1005.8	84	1791.5	0	1541.06	254.968	512.372	1146.272	1529.44	2021	1514
4597	1006.2	75	1280.21	672.4183	485.7703	1614.401	420.1778	1931.072	848.7467	1596	1975
4598	1006.8	83	3276.6	2492.472	8076.016	2047.696	2615.546	2369.933	2505.03	3954	4319
4599	1007.5	118	8071.05	682.2917	10511.45	1433.779	2565.153	3417.12	2688.84	3278	3195
4600	1007.8	83	3311.97	971.52	2065.345	1280.149	1025.396	492.3629	1016.156	2012	2029
4601	1008.5	118	4903.75	525.0917	7242.5	489.888	1113.662	1936.062	1573.74	1866	1362
4602	1008.8	84	3070.71	1970.8	2821.416	1275.517	1536.71	2070.432	3551.323	1974	2769
4603	1009.5	118	3610.5	501.075	3598.542	189.1755	490.5426	769.315	1335.092	1384	1178
4604	1010.3	74	1203.8	1736.747	428.3909	1432.004	486.3677	901.8858	538.614	510.2	1312
4605	1010.5	119	1400.5	56.074	2234.014	44.5284	248.3918	335.085	764.864	656.3	0
4606	1010.7	80	258.318	1029.258	768.558	769.82	0	720.354	1002.785	1253	258.2
4607	1011.8	84	2281.49	733.9967	2663.373	766.122	0	703	1745.814	1464	1807
4608	1012.3	71	1562.89	1348.168	903.6574	1968.521	786.961	436.7298	667.1389	668	2040
4609	1012.6	137	675.504	402.8	1856.393	362.0983	1566.595	532.565	771.12	566.1	1088
4610	1014.3	71	1866.78	2610.296	745.047	2031.792	937.3721	1568.862	1372.51	1733	1605
4611	1014.7	80	1293.62	772.26	1567.424	1282.192	256.2875	1653.396	1273.596	2248	776.2
4612	1016.3	71	2546.81	3953.193	1276.459	2575.544	1214.298	1331.958	1271.6	2783	4258
4613	1016.8	84	4091.22	1224.847	3083.273	1786.881	769.37	1895.549	3302.915	2518	1476

4614	1017.8	86	2482.31	256.4567	1794.759	1533.133	1027.09	1638.175	1531.915	1508	1989
4615	1018.3	72	1630.33	3077.656	1112.342	2783.204	1306.826	1326.909	1713.74	1846	2840
4616	1020.8	84	1531.2	503.072	2308.088	1021.128	2456.316	1388.46	2001.494	987.6	1029
4617	1022.8	83	3057.45	1248.173	6773.179	2044.597	767.0933	2091.69	2758.063	2446	2037
4618	1023.8	81	1731.87	1752.52	2051.766	255.5263	1282.192	1840.677	1501.484	1979	1255
4619	1024.8	83	2828.95	1988.721	4364.895	1794.09	1898.625	2573.197	2002.812	2035	1481
4620	1028.2	71	1740.57	1450.683	892.0663	1365.354	1106.3	1292.51	808.414	1750	2659
4621	1028.7	78	2306.01	1288.085	1902.609	770.125	1105.524	975.9125	1518.727	2464	776.2
4622	1030.2	71	2606.22	4019.804	1197.072	1721.602	1138.287	2294.976	1227.276	2851	3564
4623	1031.8	85	1526.73	502.8067	2049.531	510.2067	256.186	941.16	1281.192	1739	997
4624	1032.2	72	2610.57	2140.711	1239.51	3052.851	1271.441	1923.292	1242.773	2532	3017
4625	1032.8	83	6346.27	5422.127	9446.09	3079.28	6231.528	5494.4	7055.074	6193	6212
4626	1033.8	83	2534.26	2775.627	2752.152	1022.057	767.6	1386.84	1520.442	1974	1804
4627	1034.2	72	3116	1379.379	1684.9	1874.654	1129.49	2230.169	1676.615	2415	3987
4628	1034.8	82	2536.48	3896.721	3095.439	1282.192	1794.403	2827.378	1792.828	2207	3502
4629	1036.2	72	1151.52	1798.638	882.1818	1539.252	827.463	1664.958	774.2111	1382	2332
4630	1037.8	84	1530	1225.888	1799.186	0	0	1173.705	2261.076	2193	1250
4631	1038.7	81	1500.77	772.138	2563.247	768.558	0	721.492	2233.267	2997	777.1
4632	1042.8	85	12866.3	15507.71	16354.05	8946.573	14148.52	10653.08	12442.14	9432	12604
4633	1043.8	84	3825.73	4784.24	4104.689	2555.771	1759.158	1880.832	3559.366	4512	3017
4634	1044.8	84	3023.46	3038.055	2307.588	1020.413	512.9133	2126.651	3068.486	2516	1992
4635	1045.8	84	1758.47	467.4333	1879.563	766.8571	512.72	941.16	1286.675	1766	1736
4636	1046.2	74	1086.24	998.0803	892.0663	793.7501	599.6313	891.0288	1159.728	1552	2574
4637	1046.8	83	4009.5	2488.255	5397.992	2050.02	3983.203	1916.657	2283.822	1998	3397
4638	1047.8	82	1731.73	0	1794.561	255.49	256.0338	727.7857	501.7584	1461	480.9
4639	1048.2	72	1485.9	1276.706	1388.139	727.0667	643.5495	1920.954	1022.598	1341	1219
4640	1048.8	82	4294.62	2216.96	8272.115	1797.249	2321.04	2752.09	4032.915	4989	4111
4641	1049.8	84	1763.43	1456.623	1539.64	511.192	513.634	920.568	1533.126	1764	2180
4642	1050.2	73	904.887	835.8759	1154.102	1373.149	382.4541	1574.174	1081.866	1054	1756
4643	1050.7	83	1733.91	1276.128	6167.809	1790.171	1538.16	2365.479	1237.077	3706	3281
4644	1051.8	85	1007.42	1017.864	1024.744	766.16	256.186	214.2075	1511.604	1297	1476
4645	1052.2	72	1199.9	1457.223	600.321	654.3291	413.082	485.4104	475.125	552.7	1802
4646	1056.7	81	1760.67	771.65	1020.08	768.8625	0	699.66	1003.29	767.5	1038
4647	1056.8	85	5617.95	5700.196	5903.413	3582.403	7680	4085.325	5563.301	4866	6373
4648	1057.8	85	2503.17	1457.887	2822.348	510.2067	1283.547	1149.885	2279.53	3760	1994
4649	1058.8	83	7917.87	6419.7	1535.94	4606.371	6549.363	4408.65	6576.414	6517	6806
4650	1059.8	84	2784.91	502.6171	2563.839	1533.714	1026.703	1472.893	2774.145	2020	2223
4651	1060.8	82	2522.88	1467.606	1208.077	1277.772	1846.473	1125.704	1778.299	2029	1222
4652	1061.8	82	2017.48	2106.88	1791.72	1024.38	256.1183	683.26	1273.596	1496	1517
4653	1062.8	83	1785.82	2013.751	2669.943	1281.42	512.14	2393.633	1767.336	1504	1734
4654	1064.8	82	1407.45	503.47	2085.102	1024.38	1421.04	232.105	1492.897	1498	998.3
4655	1070.8	84	1008.1	1494.573	1792.828	0	512.2367	914.0786	514.9228	1001	1030
4656	1071.8	85	1260.81	758.462	1537.681	509.936	256.186	1197.008	2311.716	1505	2025
4657	1072.8	84	2533.82	2247.909	6923.5	1277.631	3076.624	2671.68	3289.86	3797	2060
4658	1074.2	71	339.3	293.4848	546.3678	484.0092	357.5275	304.795	314.1522	606.1	431.6
4659	1074.8	81	3300.38	1534.392	4369.648	2304.771	1894.753	2364.354	3264.988	2735	3026
4660	1075.8	84	1786.49	1160.673	2056.853	511.192	256.186	1363.1	1282.458	1242	995.1
4661	1076.2	75	1045.99	2105.798	376.4991	697.27	443.502	925.6325	698.5033	709.8	943.8
4662	1076.8	84	3594.67	1237.25	2564.968	0	512.372	1662.348	3034.735	2494	2032
4663	1078.2	74	598.148	369.4553	94.239	217.935	272.5013	330.5756	292.6875	405.3	889.5
4664	1079.8	82	1294.67	991.2	1539.597	766.47	0	1360.643	764.9793	472.8	997
4665	1082.7	82	2081.67	1029.095	2563.247	768.558	0	699.936	1279.37	1722	993.8
4666	1083.8	84	1266.91	246.48	2315.169	765.31	768.355	899.012	509.0124	1722	1033
4667	1084.8	85	2065.02	220.85	2311.029	1020.315	1582.339	1406.68	2299.056	2242	1801
4668	1088.8	84	1467.89	1521.795	2053.028	511.0525	256.0338	1338.37	1535.608	1264	1770
4669	1090.2	74	827.505	1109.335	712.1939	1517.084	633.3039	1379.963	595.536	951.6	1246
4670	1090.7	82	1759.39	1287.231	2836.852	1875.862	1281.853	1215.19	1526.501	1990	1258
4671	1092.2	71	1113.7	1283.571	555.1328	1340.504	565.4108	1002.333	759.2862	1083	1343
4672	1092.8	84	2039.62	1253.888	1798.957	2052.012	256.186	1127.252	2268.145	2229	2504
4673	1099.6	80	1986.93	1763.271	769.6238	255.78	0	953.16	1026.514	764.4	775.5
4674	1100.8	83	3830.03	4584.082	5676.789	2300.476	2265.387	1402.67	2004.078	4720	3800
4675	1101.8	82	3316.97	1469.1	3081.72	1023.219	512.14	1408.74	2269.41	2243	2029
4676	1102.8	84	2736.07	1431.357	2311.29	1276.773	1024.473	2134.333	2738.725	2994	1512
4677	1110.8	85	11527.6	11453.4	10511.7	5881.369	10655.28	7705.664	6674.708	13128	8789
4678	1111.8	85	3301.65	2177.348	2563.707	1019.863	2305.8	1610.422	2295.424	6301	2244

4679	1112.8	83	1036.58	1988.58	1539.64	255.374	0	685.2667	767.3172	1508	2031
4680	1113.8	82	518.634	256.65	1538.378	0	256.07	0	508.3003	2253	1654
4681	1114.8	83	1503.13	1711.272	3331.856	1277.257	513.9829	1355.314	2497.946	2994	1803
4682	1116.7	82	3307.79	1264	4555.695	1539.64	1280.592	944.88	1998.064	1952	2781
4683	1117.8	83	2040.89	477.9	1461.05	510.98	256.07	235.4229	1515.67	1481	1739
4684	1122.7	82	1988.14	515.1457	2150.395	511.56	256.07	677.9214	513.358	961.5	774.7
4685	1124.8	85	5586.32	5005.633	4362.24	1531.877	3942.532	2836.377	4079.303	3026	4302
4686	1125.8	84	1553.47	723.89	3075.85	510.4	770.3429	1146.09	1525.53	1763	1953
4687	1126.8	83	6027.43	4003.769	7133.959	3323.049	4551.895	3543.265	3592.908	2294	4511
4688	1127.8	83	2818.36	476.7525	4368.96	1276.644	512.0111	1588.225	2035.728	1006	1737
4689	1136.2	74	412.946	643.8874	310.5039	24.81129	273.6971	24.64843	149.34	394.3	841
4690	1139.8	86	1285.67	476.28	1794.564	0	0	233.988	1530.704	1267	221.8
4691	1140.8	85	3863.33	942.2871	2821.416	1275.806	1026.703	1357.571	2034.466	2749	3542
4692	1141.8	85	1266.97	0	1538.378	254.765	0	661.072	1017.803	1009	960.1
4693	1142.7	83	3765.7	512.9133	4623.843	1795.826	1280.93	2304.83	2531.386	2923	3067
4694	1144.8	84	2789.51	749.552	2396.57	1276.096	256.186	961.144	2791.53	1499	1769
4695	1148.2	73	946.08	1044.582	435.157	624	350.3588	1482.243	459.375	1742	721.8
4696	1151.8	81	258.487	772.6667	511.56	514.855	255.78	231.19	0	511.4	780.8
4697	1155.8	86	1007.42	477.036	2049.531	0	0	911.344	771.65	1248	770.4
4698	1156.7	82	1009.01	736.0845	511.6615	1282.192	256.2875	1407.48	251.7848	988.5	740.6
4699	1158.7	81	2280.98	1027.83	2819.067	1027.268	512.2367	2105.66	490.918	489.4	1034
4700	1159.8	81	258.487	1254.88	768.6	255.78	0	450.2583	508.597	1007	1483
4701	1160.7	83	2564.56	256.592	2494.692	510.748	256.186	1365.25	1273.596	1461	1290
4702	1168.7	83	3774.02	1759.444	2308.85	1276.35	2841.336	1620.504	2794.387	4751	2286
4703	1169.8	85	2556.51	724.272	1797.961	0	0	1598.948	1254.825	1028	773.2
4704	1170.7	84	2061.77	1465.282	1538.649	509.3044	1277.64	1148.299	2297.244	1484	2244
4705	1172.7	79	1008.1	1498.27	1090.211	769.4714	256.07	1149.299	485.705	728	258.4
4706	1173.7	81	751.66	256.9574	0	769.82	768.558	471.696	486.717	234.7	1259
4707	1178.8	84	5575.4	8077.475	7708.888	3832.819	7494.002	5004.703	5869.503	7577	7769
4708	1179.8	85	2066.29	2520.658	1537.972	510.2067	1541.267	918.39	2043.317	2984	1512
4709	1180.8	84	1757.9	733.9967	1795.352	510.2067	0	727.375	492.085	257.2	1250
4710	1182.8	84	2018.23	1762.35	2053.028	510.2067	512.2367	938.6	1758.82	2510	2546
4711	1184.7	82	984.3	1029.507	3180.828	1538.378	256.186	1611.63	960.7625	1983	2003
4712	1186.7	83	2800.82	735.648	3597.573	1022.754	0	933.45	2806.722	990.5	1520
4713	1192.8	84	1964.75	3959.743	3078.018	1021.331	1794.52	3310.885	3068.396	4234	2536
4714	1194.7	84	3303.27	2970.22	3940.018	2554.514	2305.891	2984.135	3326.512	3291	3014
4715	1196.8	84	1989.41	955.584	1027.795	511.192	256.186	935.784	1790.124	1503	1255
4716	1208.8	85	2558.11	1989.845	2051.083	1021.67	256.4567	896.885	1510.338	1487	703.9
4717	1236.7	82	1990.68	1496.495	2312.553	1534.199	1208.834	909.4667	2006.195	2708	2103
4718	1246.8	85	5085.06	4514.71	6664.385	2046.766	4689.18	2127.068	4044.65	3736	3791
4719	1247.8	85	1291.59	220.85	1795.826	509.936	256.186	466.416	2272.929	2024	1509
4720	1248.8	84	1528.73	0	1025.962	510.2067	0	257.1333	766.691	514.9	0
