# Supplementary material for: Effects of Lycium Barbarum Polysaccharides on the Metabolism of Dendritic Cells: An In Vitro Study
Source: J Immunol Res. 2022 Oct 19;2022:5882136. doi: 10.1155/2022/5882136 (PMC9605842; doi:10.1155/2022/5882136)
Supplement: Supplementary Materials — Figure S1: the total ions chromatogram in negative ion and positive ion mode. Table Sl: the raw data of negative ion mode. Table S2: the raw data of positive ion mode. (Supplementary Materials). [file 5882136.f1.zip › Supplementary data-Figure S1.pdf]

Supplementary data (Figure S1)

Supplementary data (Figure S1) for manuscript entitled **Effects of Lycium barbarum polysaccharides on the metabolism of dendritic cells：an in vitro study.**

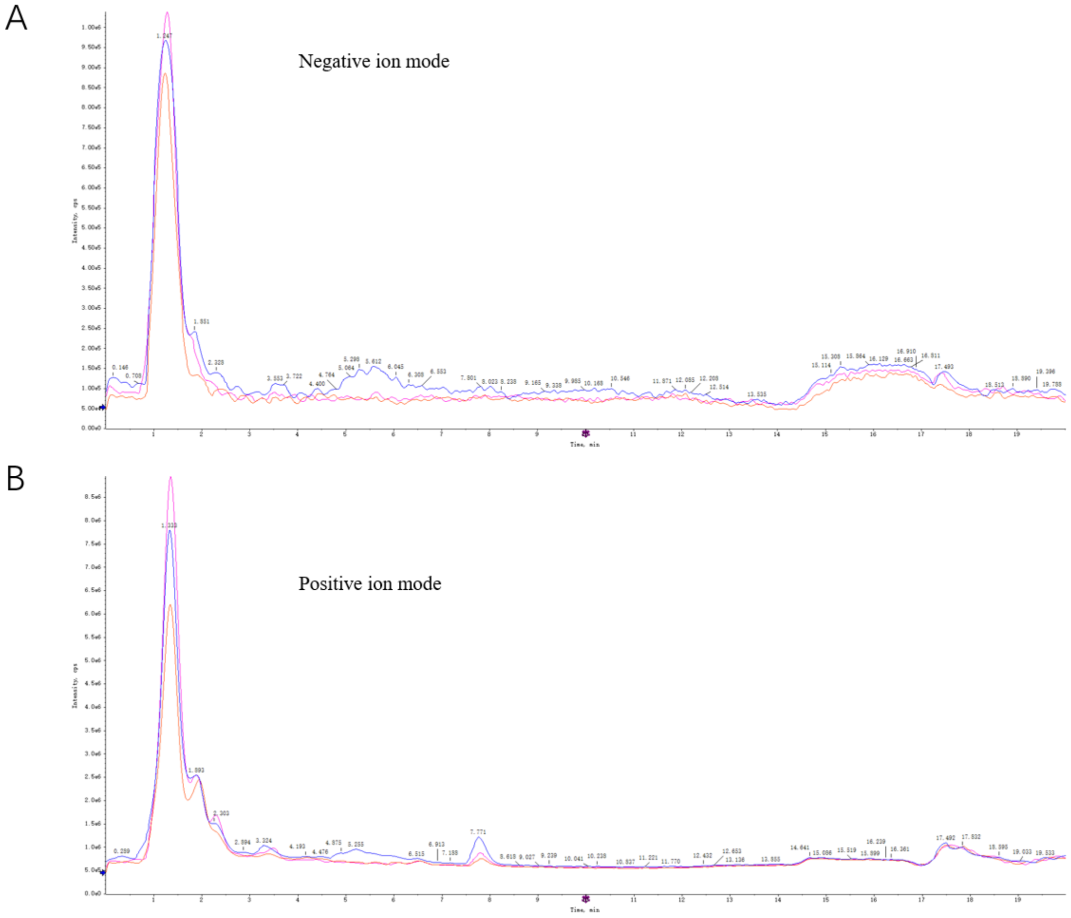

Figure S1. The total ions chromatogram in negative ion (A) and positive ion mode (B).
